# Supplementary material for: Fine-mapping a genome-wide meta-analysis of 98,374 migraine cases identifies 181 sets of candidate causal variants
Source: Nat Commun. 2026 Jan 12;17:355. doi: 10.1038/s41467-025-64880-3 (PMC12796328; doi:10.1038/s41467-025-64880-3)
Supplement: Supplementary file 1 — Supplementary Figs. [file 41467_2025_64880_MOESM1_ESM.pdf]

Supplementary Figure 1.

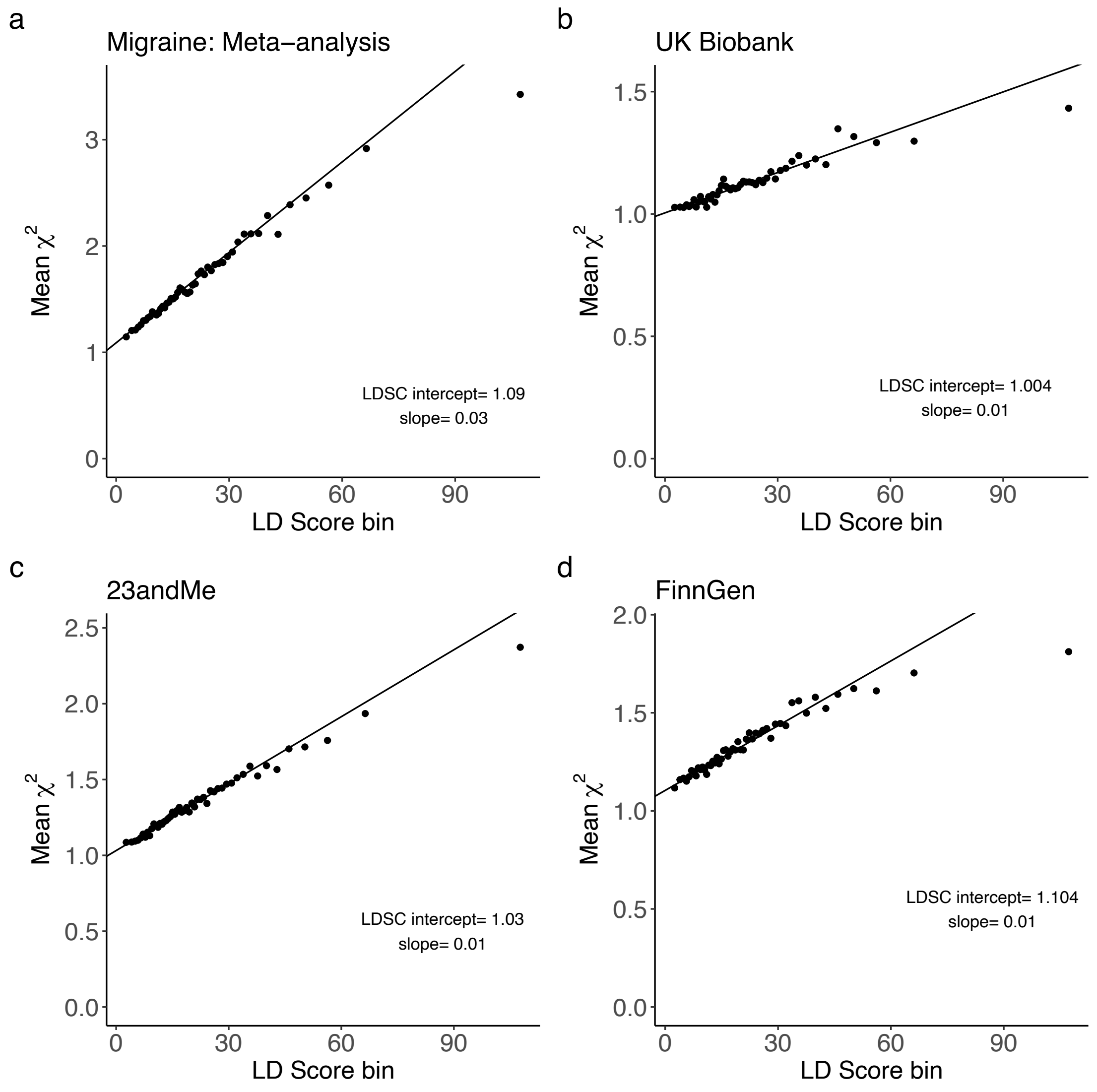

Supplementary Figure 1. LD Score plots of GWAS results from a) the meta-analysis (N=967,534; 98,374 migraine cases and 869,160 controls), and separately from b) UK Biobank (N=341,050; 10,881 migraine cases and 341,050 controls) c) 23andMe (N=283,985; 53,109 migraine cases and 230,876 controls), and d) FinnGen (N=342,499; 34,385 migraine cases and 869,160 controls). X-axes show LD Score bins and y-axes corresponding mean χ^2-statistics. LDSC intercept measures confounding inflation of the GWAS summary statistics due to cryptic relatedness, population stratification and model misspecification, and values close to 1 indicate only small inflation. Heritability can be estimated by scaling the regression slope.

Supplementary Figure 2.

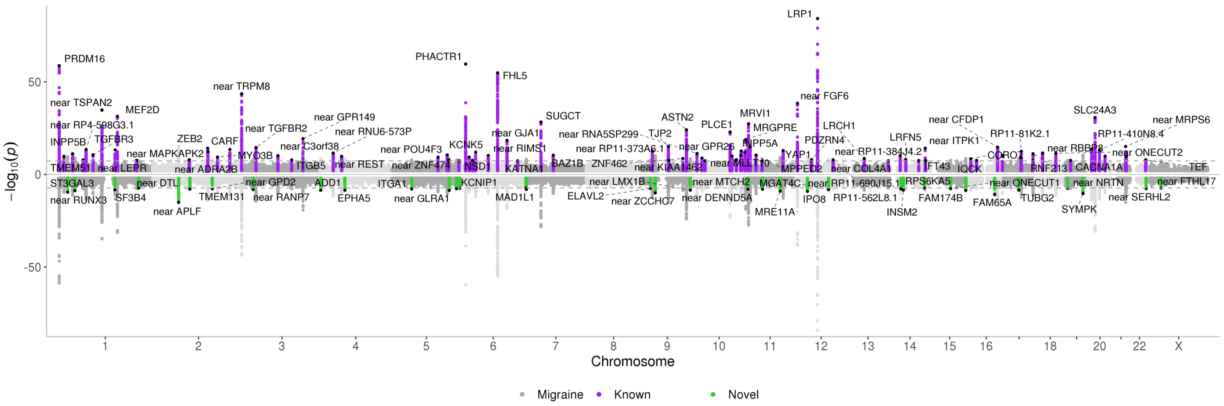

Supplementary Figure 2. Miami plot of the inverse-variance weighted fixed effects meta-analysis including 98,374 migraine cases and 869,160 controls. X-axis presents the chromosomal location and y-axis the -$\log_{10}$(*P*-value). The upper panel shows the previously known loci highlighted in purple, and the lower panel shows the new loci in green.

Supplementary Figure 3. Forest plots of the 122 lead migraine variants from the inverse-variance weighted fixed-effect meta-analysis of the discovery (98,374 cases and 869,160 controls) and replication data (34,807 cases and 193,475 controls). For each variant, the log-odds-ratio estimate (BETA) with its 95%-confidence intervals (green) are shown from each of the three studies included in the discovery meta-analysis and from the two studies included in the replication meta-analysis, and the combined estimates of the inverse-variance weighted fixed-effect meta-analyses (blue diamonds). The sample sizes of each study are presented as grey squares. Additionally, the lead variant and effect allele, two-sided *P*-value by the inverse-variance weighted fixed-effect meta-analysis and heterogeneity index (I^2) are displayed. Figure 3 is divided into 122 parts on the following pages.

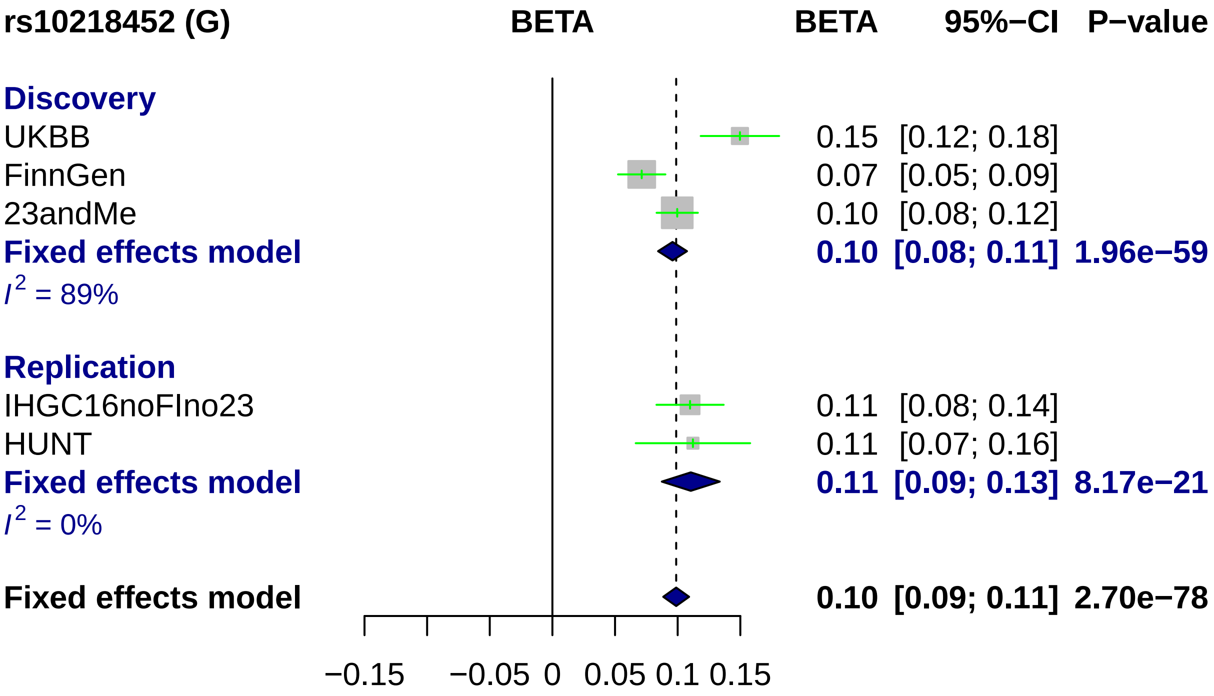
rs10218452 (G)
BETA
BETA
95%−CI
P−value
Discovery
UKBB
0.15 [0.12; 0.18]
FinnGen
0.07 [0.05; 0.09]
23andMe
0.10 [0.08; 0.12]
Fixed effects model
0.10 [0.08; 0.11] 1.96e−59
I2 = 89%
Replication
IHGC16noFIno23
0.11 [0.08; 0.14]
HUNT
0.11 [0.07; 0.16]
Fixed effects model
0.11 [0.09; 0.13] 8.17e−21
I2 = 0%
Fixed effects model
0.10 [0.09; 0.11] 2.70e−78
−0.15
−0.05
0
0.05
0.1
0.15

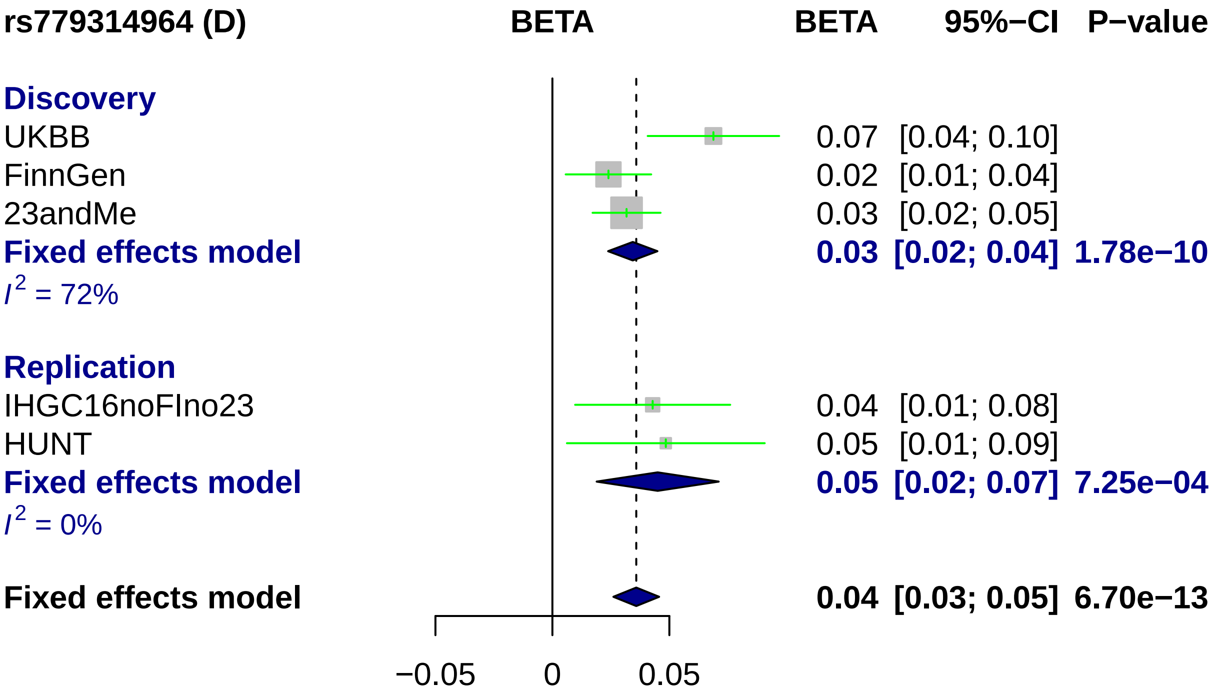
rs779314964 (D)
BETA
BETA
95%−CI
P−value
Discovery
UKBB
0.07 [0.04; 0.10]
FinnGen
0.02 [0.01; 0.04]
23andMe
0.03 [0.02; 0.05]
Fixed effects model
0.03 [0.02; 0.04] 1.78e−10
I^2 = 72%
Replication
IHGC16noFIno23
0.04 [0.01; 0.08]
HUNT
0.05 [0.01; 0.09]
Fixed effects model
0.05 [0.02; 0.07] 7.25e−04
I^2 = 0%
Fixed effects model
0.04 [0.03; 0.05] 6.70e−13
−0.05
0
0.05

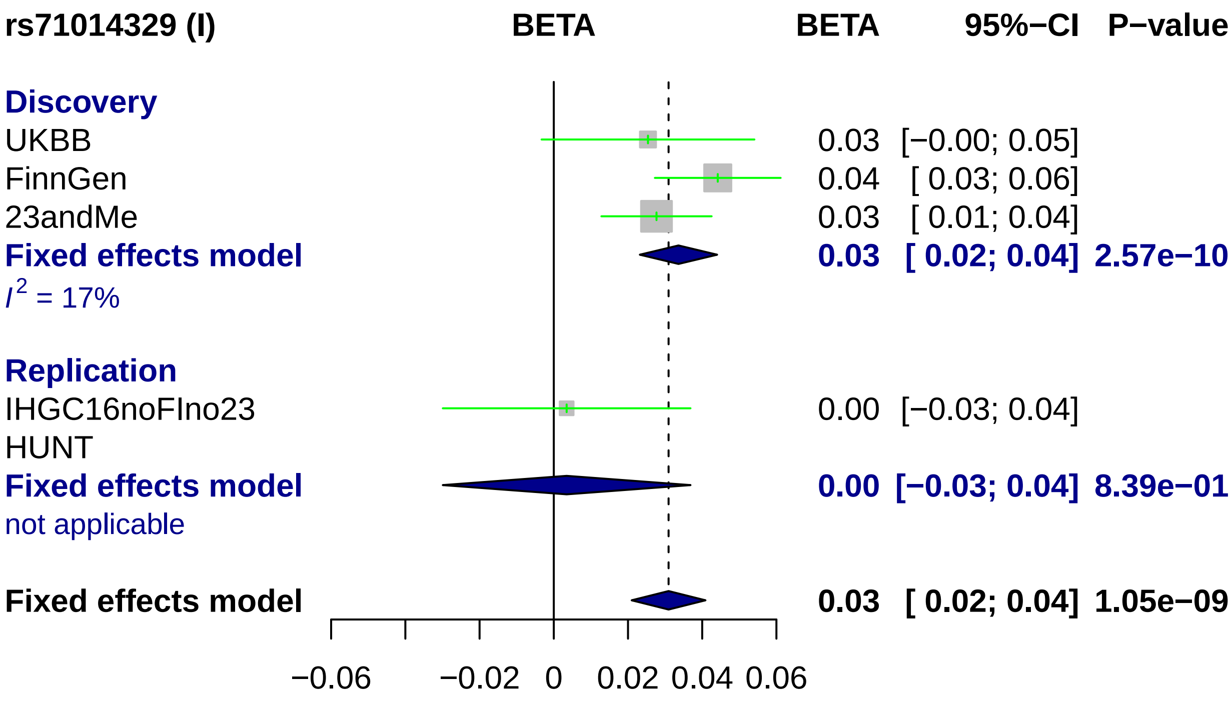

rs71014329 (I)
BETA
BETA
95%−CI
P−value
Discovery
UKBB
0.03
[−0.00; 0.05]
FinnGen
0.04
[0.03; 0.06]
23andMe
0.03
[0.01; 0.04]
Fixed effects model
0.03
[0.02; 0.04]
2.57e−10
I^2 = 17%
Replication
IHGC16noFIno23
0.00
[−0.03; 0.04]
HUNT
Fixed effects model
0.00
[−0.03; 0.04]
8.39e−01
not applicable
Fixed effects model
0.03
[0.02; 0.04]
1.05e−09
−0.06
−0.02
0
0.02
0.04
0.06

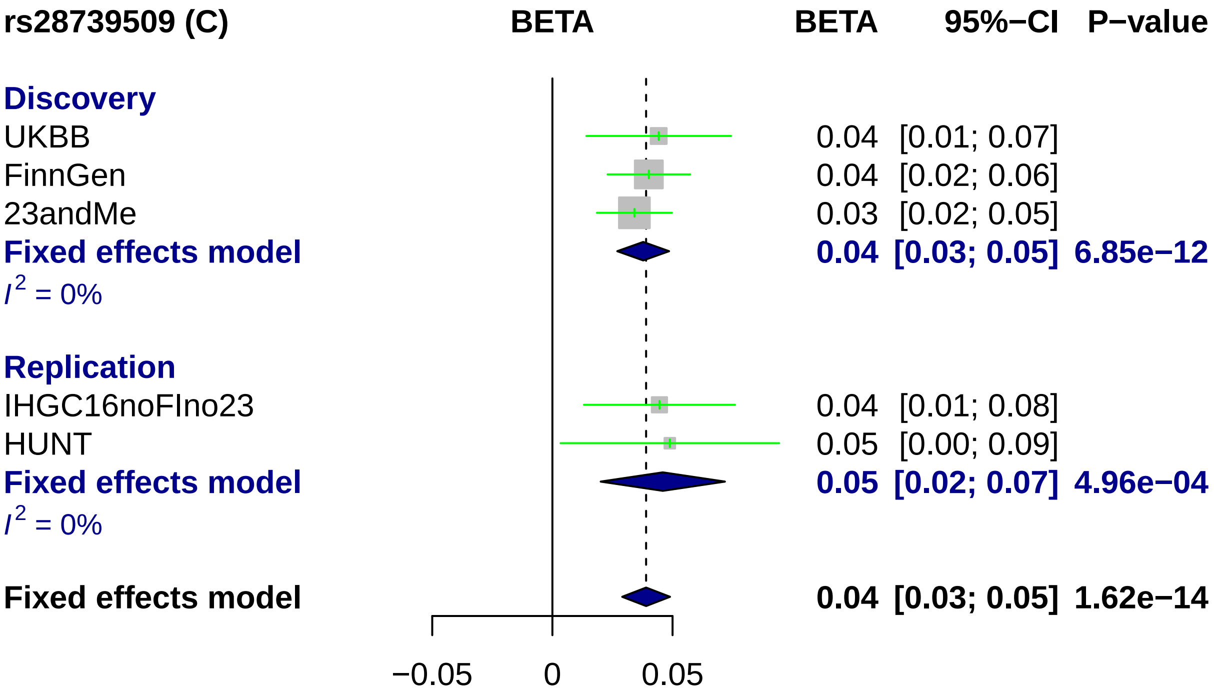
rs28739509 (C)
BETA
BETA
95%−CI
P−value
Discovery
UKBB
0.04
[0.01; 0.07]
FinnGen
0.04
[0.02; 0.06]
23andMe
0.03
[0.02; 0.05]
Fixed effects model
0.04
[0.03; 0.05]
6.85e−12
I2 = 0%
Replication
IHGC16noFIno23
0.04
[0.01; 0.08]
HUNT
0.05
[0.00; 0.09]
Fixed effects model
0.05
[0.02; 0.07]
4.96e−04
I2 = 0%
Fixed effects model
0.04
[0.03; 0.05]
1.62e−14
−0.05
0
0.05

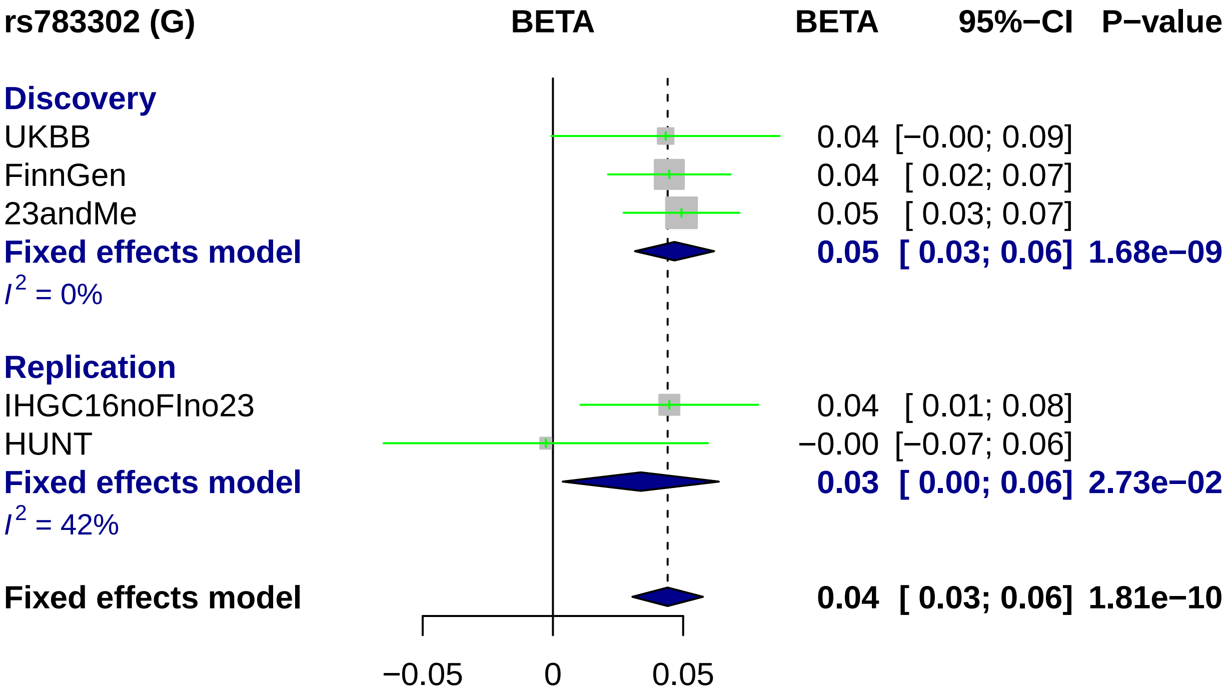
rs783302 (G)
BETA
BETA
95%−CI
P−value
Discovery
UKBB
0.04 [−0.00; 0.09]
FinnGen
0.04 [0.02; 0.07]
23andMe
0.05 [0.03; 0.07]
Fixed effects model
0.05 [0.03; 0.06] 1.68e−09
I2 = 0%
Replication
IHGC16noFIno23
0.04 [0.01; 0.08]
HUNT
−0.00 [−0.07; 0.06]
Fixed effects model
0.03 [0.00; 0.06] 2.73e−02
I2 = 42%
Fixed effects model
0.04 [0.03; 0.06] 1.81e−10
−0.05
0
0.05

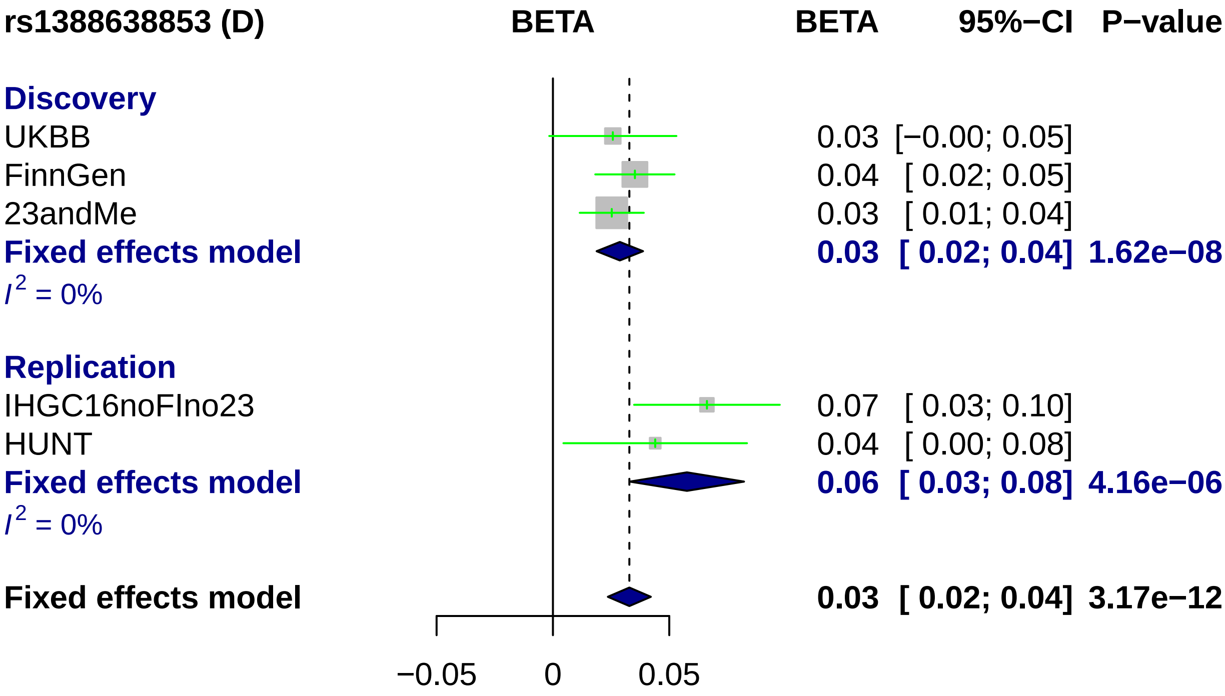
rs1388638853 (D)
BETA
BETA
95%−CI
P−value
Discovery
UKBB
0.03 [−0.00; 0.05]
FinnGen
0.04 [0.02; 0.05]
23andMe
0.03 [0.01; 0.04]
Fixed effects model
0.03 [0.02; 0.04] 1.62e−08
I² = 0%
Replication
IHGC16noFIno23
0.07 [0.03; 0.10]
HUNT
0.04 [0.00; 0.08]
Fixed effects model
0.06 [0.03; 0.08] 4.16e−06
I² = 0%
Fixed effects model
0.03 [0.02; 0.04] 3.17e−12
−0.05
0
0.05

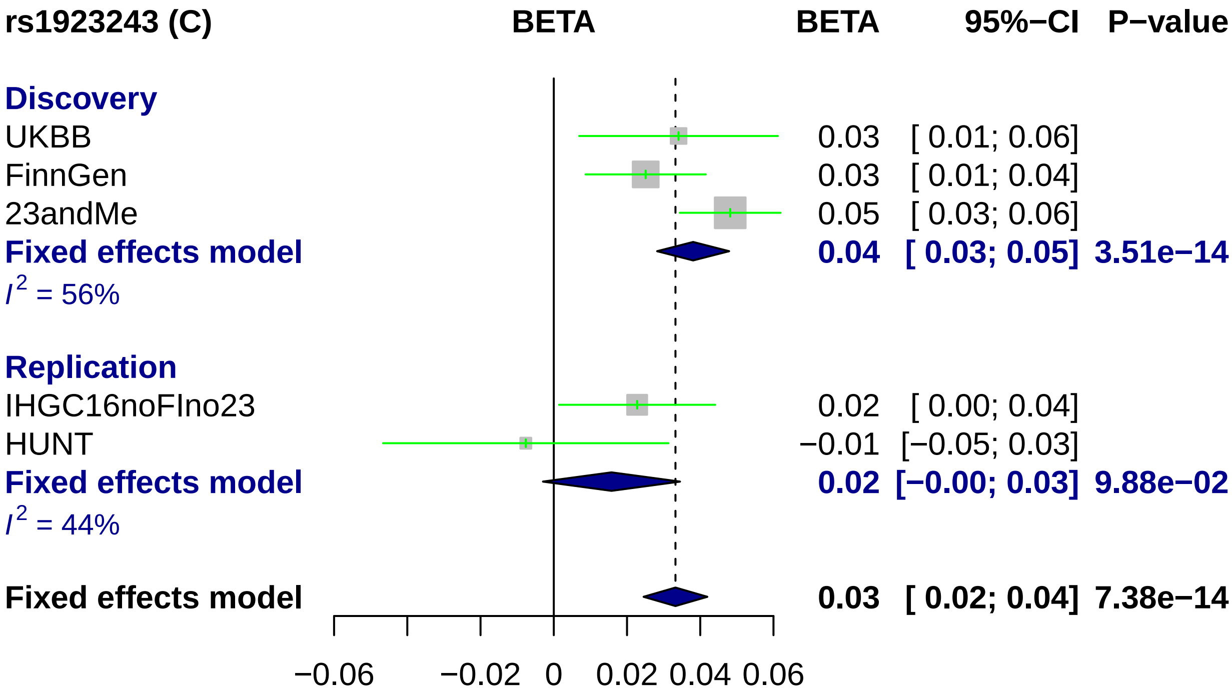
rs1923243 (C)
BETA
BETA
95%−CI
P−value
Discovery
UKBB
0.03 [0.01; 0.06]
FinnGen
0.03 [0.01; 0.04]
23andMe
0.05 [0.03; 0.06]
Fixed effects model
0.04 [0.03; 0.05] 3.51e−14
I2 = 56%
Replication
IHGC16noFIno23
0.02 [0.00; 0.04]
HUNT
−0.01 [−0.05; 0.03]
Fixed effects model
0.02 [−0.00; 0.03] 9.88e−02
I2 = 44%
Fixed effects model
0.03 [0.02; 0.04] 7.38e−14
−0.06
−0.02
0
0.02
0.04
0.06

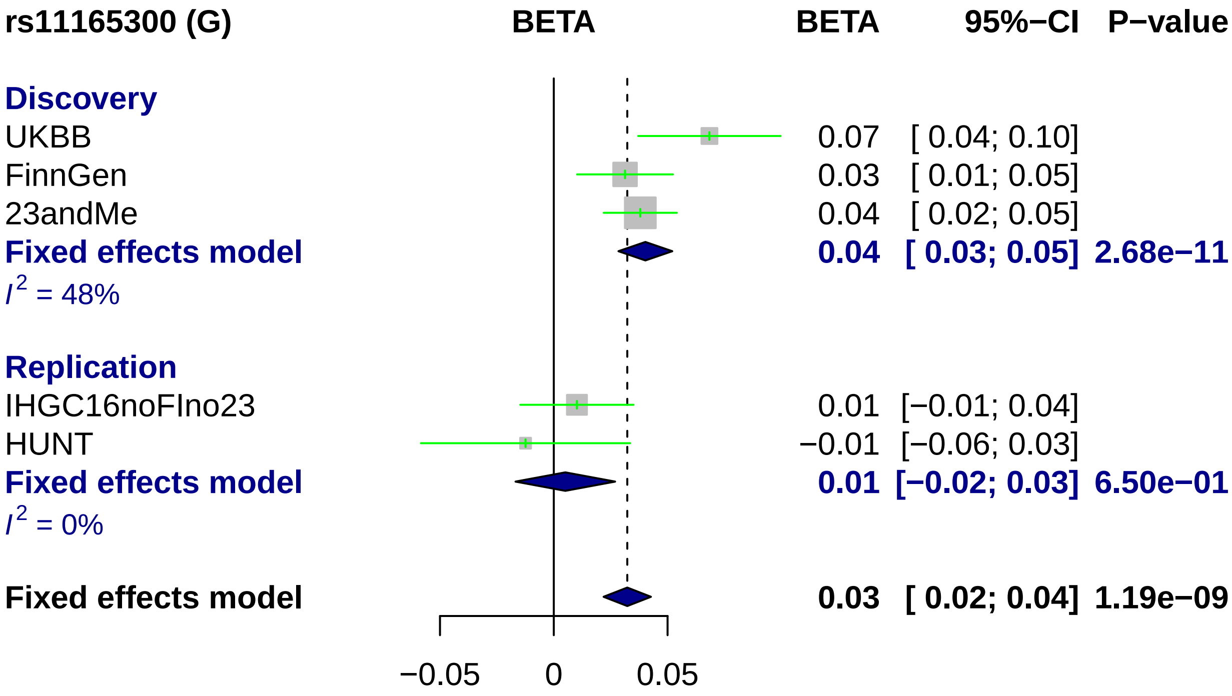
rs11165300 (G)
BETA
BETA
95%−CI
P−value
Discovery
UKBB 0.07 [0.04; 0.10]
FinnGen 0.03 [0.01; 0.05]
23andMe 0.04 [0.02; 0.05]
Fixed effects model 0.04 [0.03; 0.05] 2.68e−11
I^2 = 48%
Replication
IHGC16noFIno23 0.01 [−0.01; 0.04]
HUNT −0.01 [−0.06; 0.03]
Fixed effects model 0.01 [−0.02; 0.03] 6.50e−01
I^2 = 0%
Fixed effects model 0.03 [0.02; 0.04] 1.19e−09
−0.05
0
0.05

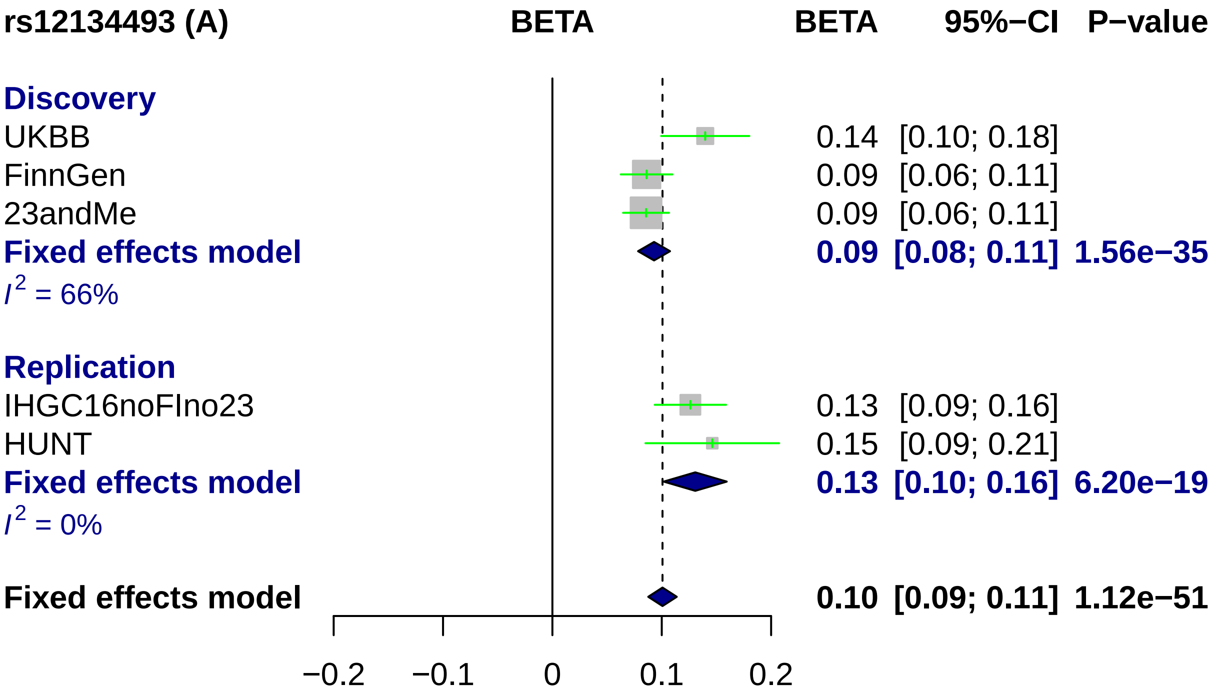
rs12134493 (A)
BETA
BETA
95%−CI
P−value
Discovery
UKBB
0.14 [0.10; 0.18]
FinnGen
0.09 [0.06; 0.11]
23andMe
0.09 [0.06; 0.11]
Fixed effects model
0.09 [0.08; 0.11] 1.56e−35
I2 = 66%
Replication
IHGC16noFIno23
0.13 [0.09; 0.16]
HUNT
0.15 [0.09; 0.21]
Fixed effects model
0.13 [0.10; 0.16] 6.20e−19
I2 = 0%
Fixed effects model
0.10 [0.09; 0.11] 1.12e−51
−0.2
−0.1
0
0.1
0.2

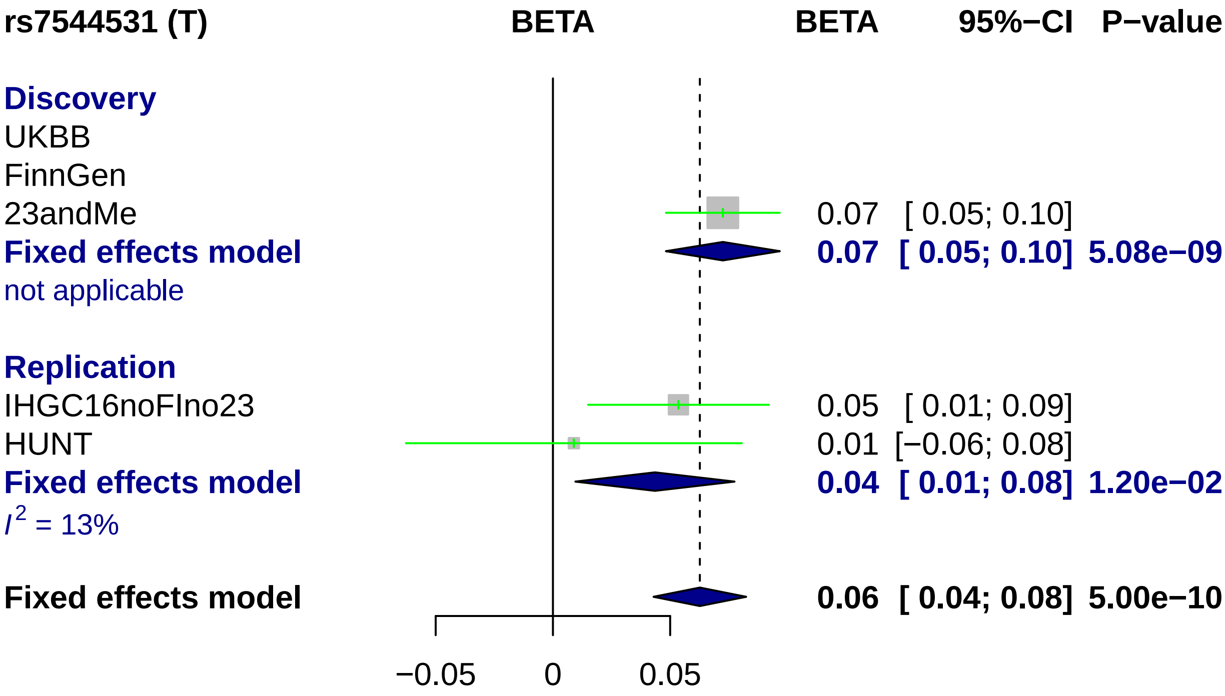
rs7544531 (T)
BETA
BETA
95%−CI
P−value
Discovery
UKBB
FinnGen
23andMe
0.07 [0.05; 0.10]
Fixed effects model
0.07 [0.05; 0.10] 5.08e−09
not applicable
Replication
IHGC16noFIno23
0.05 [0.01; 0.09]
HUNT
0.01 [−0.06; 0.08]
Fixed effects model
0.04 [0.01; 0.08] 1.20e−02
I2 = 13%
Fixed effects model
0.06 [0.04; 0.08] 5.00e−10
−0.05
0
0.05

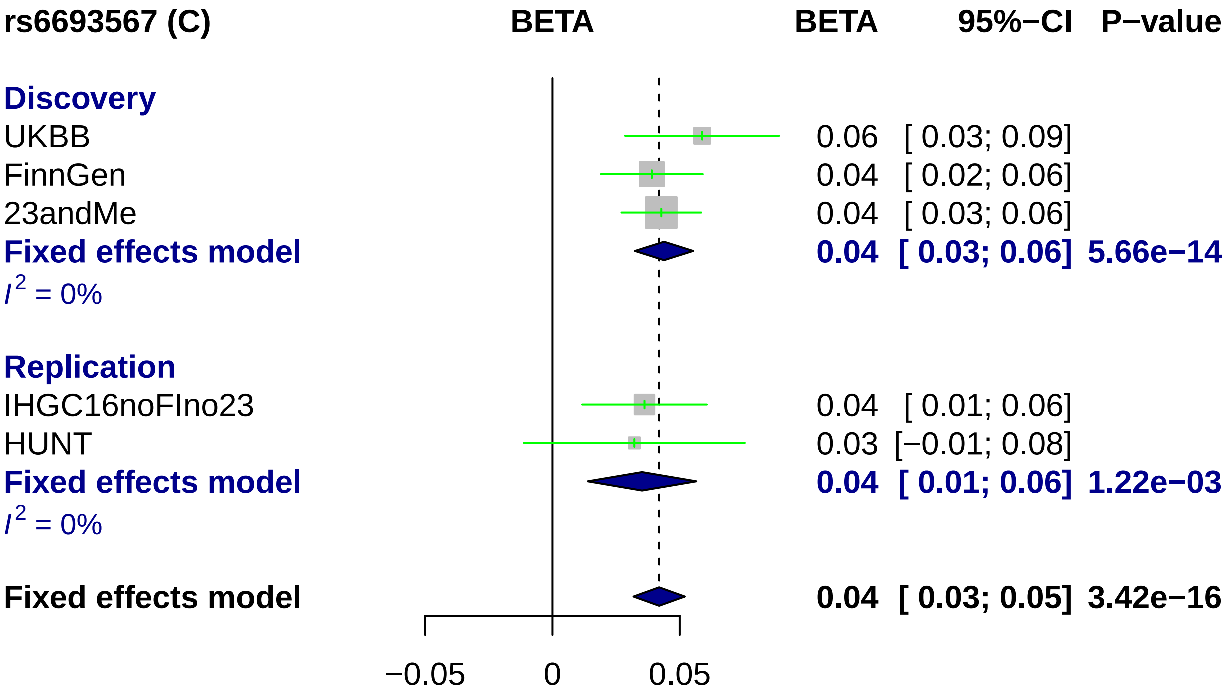
rs6693567 (C)
BETA
BETA
95%−CI
P−value
Discovery
UKBB
0.06 [0.03; 0.09]
FinnGen
0.04 [0.02; 0.06]
23andMe
0.04 [0.03; 0.06]
Fixed effects model
0.04 [0.03; 0.06] 5.66e−14
I2 = 0%
Replication
IHGC16noFIno23
0.04 [0.01; 0.06]
HUNT
0.03 [−0.01; 0.08]
Fixed effects model
0.04 [0.01; 0.06] 1.22e−03
I2 = 0%
Fixed effects model
0.04 [0.03; 0.05] 3.42e−16
−0.05
0
0.05

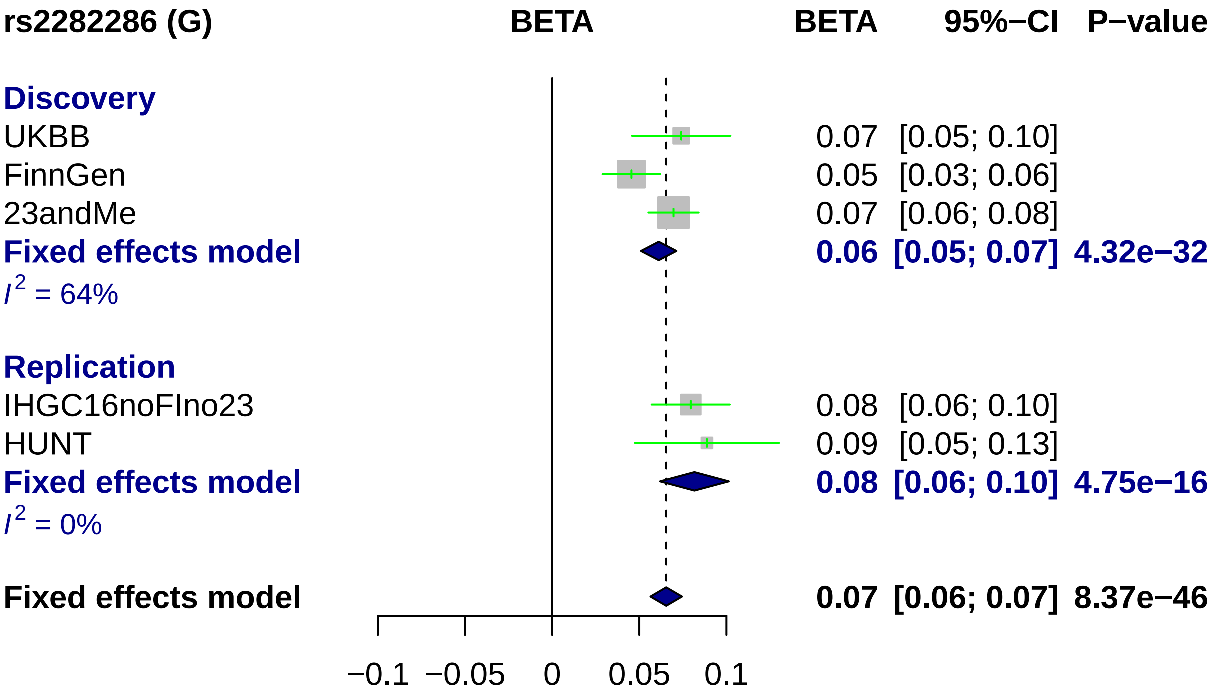
rs2282286 (G)
BETA
BETA
95%−CI
P−value
Discovery
UKBB
0.07 [0.05; 0.10]
FinnGen
0.05 [0.03; 0.06]
23andMe
0.07 [0.06; 0.08]
Fixed effects model
0.06 [0.05; 0.07] 4.32e−32
I2 = 64%
Replication
IHGC16noFIno23
0.08 [0.06; 0.10]
HUNT
0.09 [0.05; 0.13]
Fixed effects model
0.08 [0.06; 0.10] 4.75e−16
I2 = 0%
Fixed effects model
0.07 [0.06; 0.07] 8.37e−46
−0.1
−0.05
0
0.05
0.1

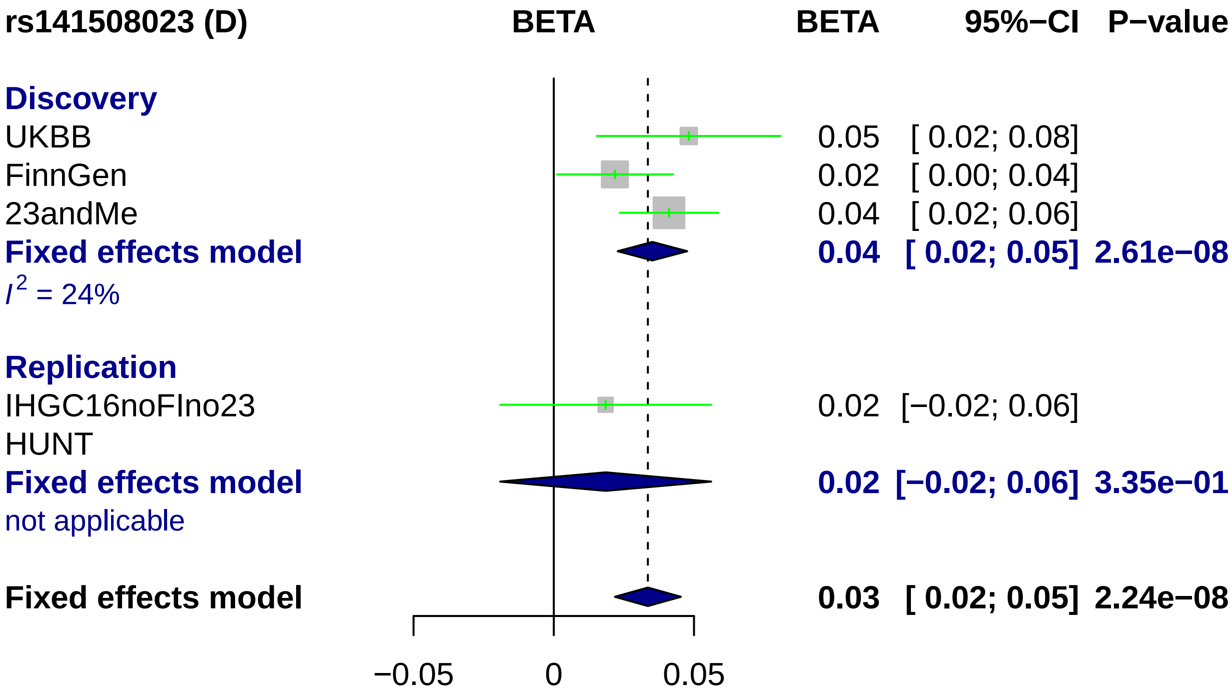
rs141508023 (D)
BETA
BETA
95%−CI
P−value
Discovery
UKBB
0.05 [0.02; 0.08]
FinnGen
0.02 [0.00; 0.04]
23andMe
0.04 [0.02; 0.06]
Fixed effects model
0.04 [0.02; 0.05] 2.61e−08
I^2 = 24%
Replication
IHGC16noFIno23
0.02 [−0.02; 0.06]
HUNT
Fixed effects model
0.02 [−0.02; 0.06] 3.35e−01
not applicable
Fixed effects model
0.03 [0.02; 0.05] 2.24e−08
−0.05
0
0.05

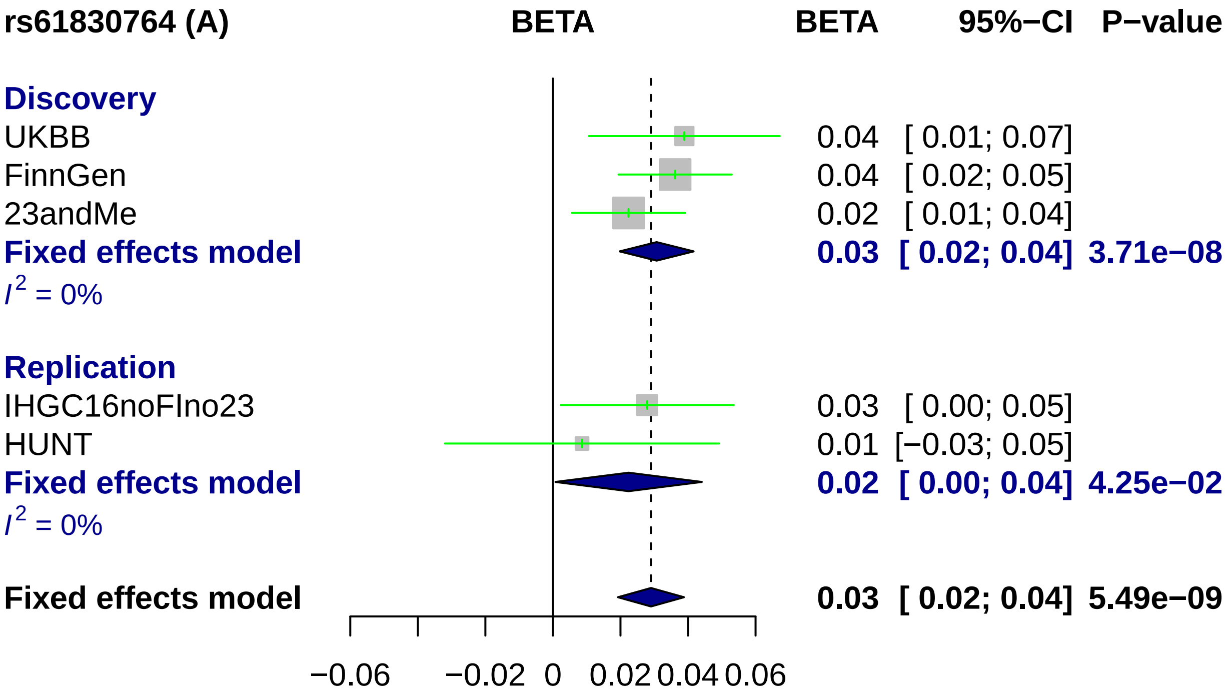
rs61830764 (A)
BETA
BETA
95%−CI
P−value
Discovery
UKBB 0.04 [0.01; 0.07]
FinnGen 0.04 [0.02; 0.05]
23andMe 0.02 [0.01; 0.04]
Fixed effects model 0.03 [0.02; 0.04] 3.71e−08
I2 = 0%
Replication
IHGC16noFIno23 0.03 [0.00; 0.05]
HUNT 0.01 [−0.03; 0.05]
Fixed effects model 0.02 [0.00; 0.04] 4.25e−02
I2 = 0%
Fixed effects model 0.03 [0.02; 0.04] 5.49e−09
−0.06
−0.02
0
0.02
0.04
0.06

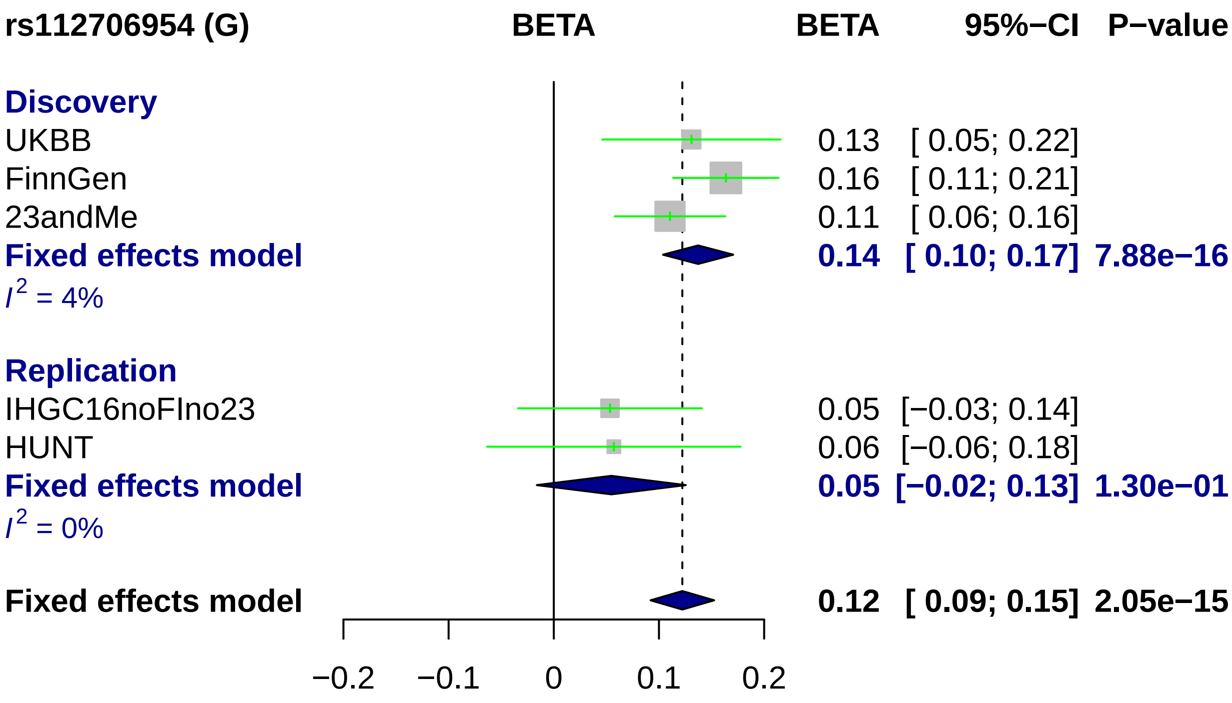
rs112706954 (G)
BETA
BETA
95%−CI
P−value
Discovery
UKBB
0.13
[0.05; 0.22]
FinnGen
0.16
[0.11; 0.21]
23andMe
0.11
[0.06; 0.16]
Fixed effects model
0.14
[0.10; 0.17]
7.88e−16
I2 = 4%
Replication
IHGC16noFIno23
0.05
[−0.03; 0.14]
HUNT
0.06
[−0.06; 0.18]
Fixed effects model
0.05
[−0.02; 0.13]
1.30e−01
I2 = 0%
Fixed effects model
0.12
[0.09; 0.15]
2.05e−15
−0.2
−0.1
0
0.1
0.2

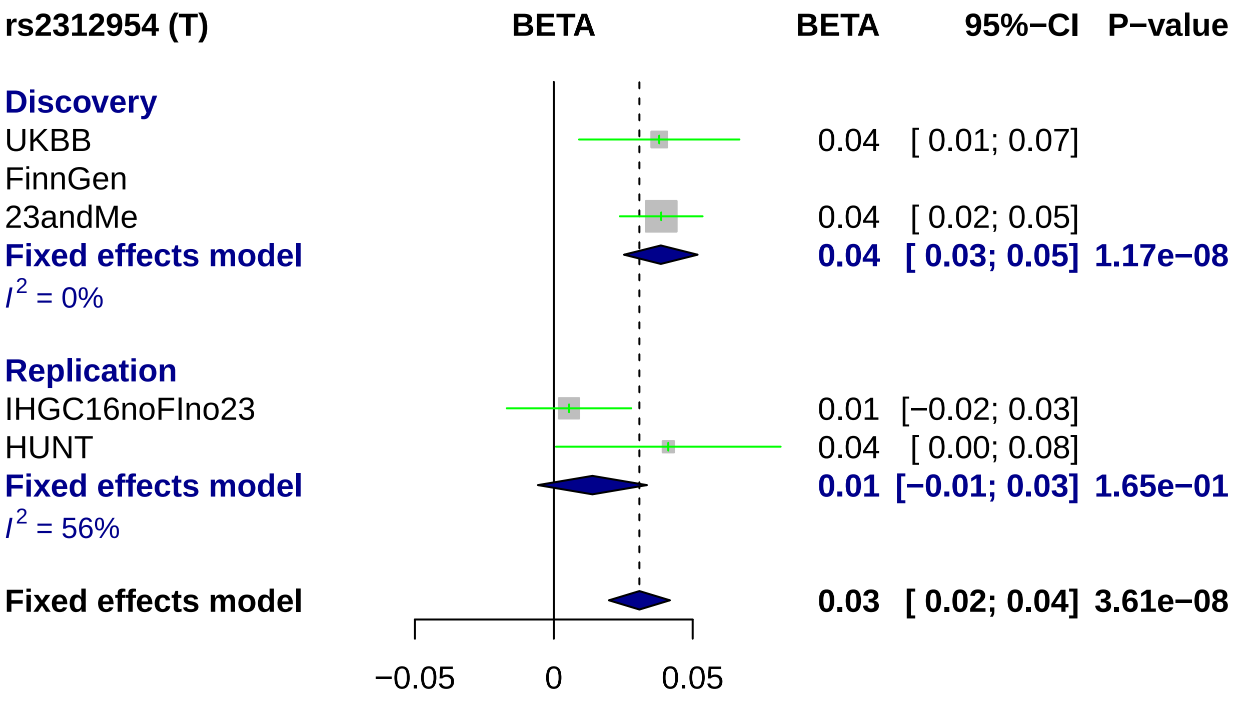
rs2312954 (T)
BETA
BETA
95%−CI
P−value
Discovery
UKBB
0.04 [0.01; 0.07]
FinnGen
23andMe
0.04 [0.02; 0.05]
Fixed effects model
0.04 [0.03; 0.05] 1.17e−08
I2 = 0%
Replication
IHGC16noFIno23
0.01 [−0.02; 0.03]
HUNT
0.04 [0.00; 0.08]
Fixed effects model
0.01 [−0.01; 0.03] 1.65e−01
I2 = 56%
Fixed effects model
0.03 [0.02; 0.04] 3.61e−08
−0.05
0
0.05

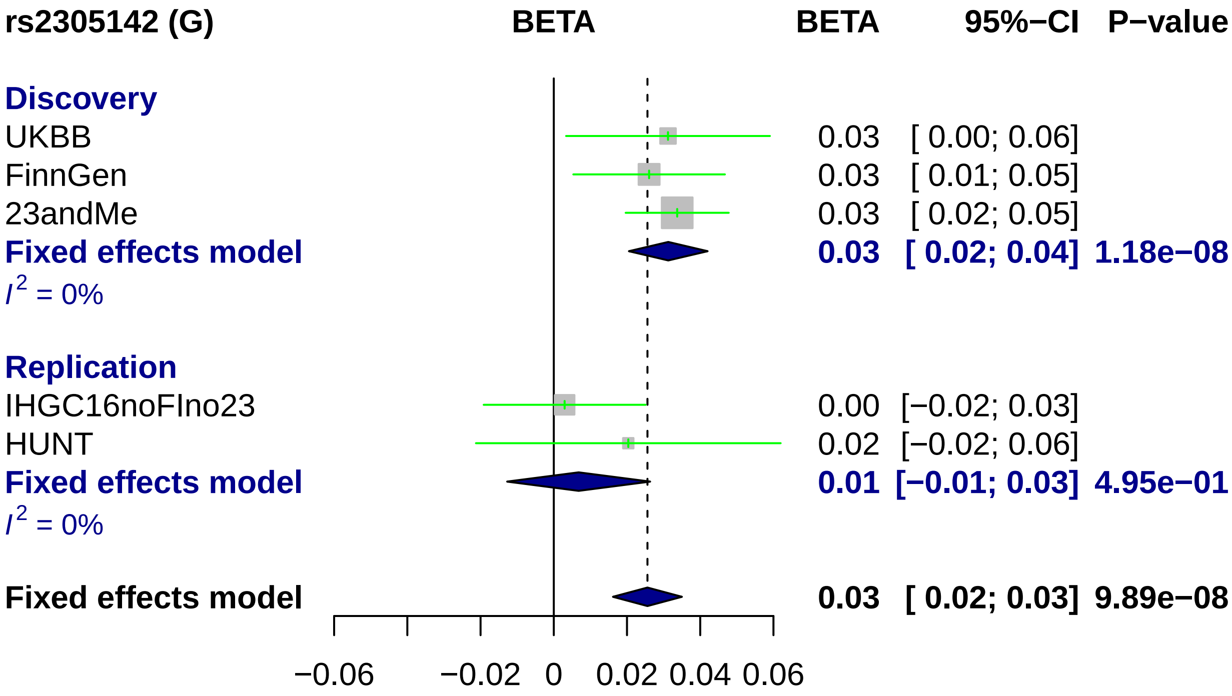
rs2305142 (G)
BETA
BETA
95%−CI
P−value
Discovery
UKBB
0.03
[0.00; 0.06]
FinnGen
0.03
[0.01; 0.05]
23andMe
0.03
[0.02; 0.05]
Fixed effects model
0.03
[0.02; 0.04]
1.18e−08
I2 = 0%
Replication
IHGC16noFIno23
0.00
[−0.02; 0.03]
HUNT
0.02
[−0.02; 0.06]
Fixed effects model
0.01
[−0.01; 0.03]
4.95e−01
I2 = 0%
Fixed effects model
0.03
[0.02; 0.03]
9.89e−08
−0.06
−0.02
0
0.02
0.04
0.06

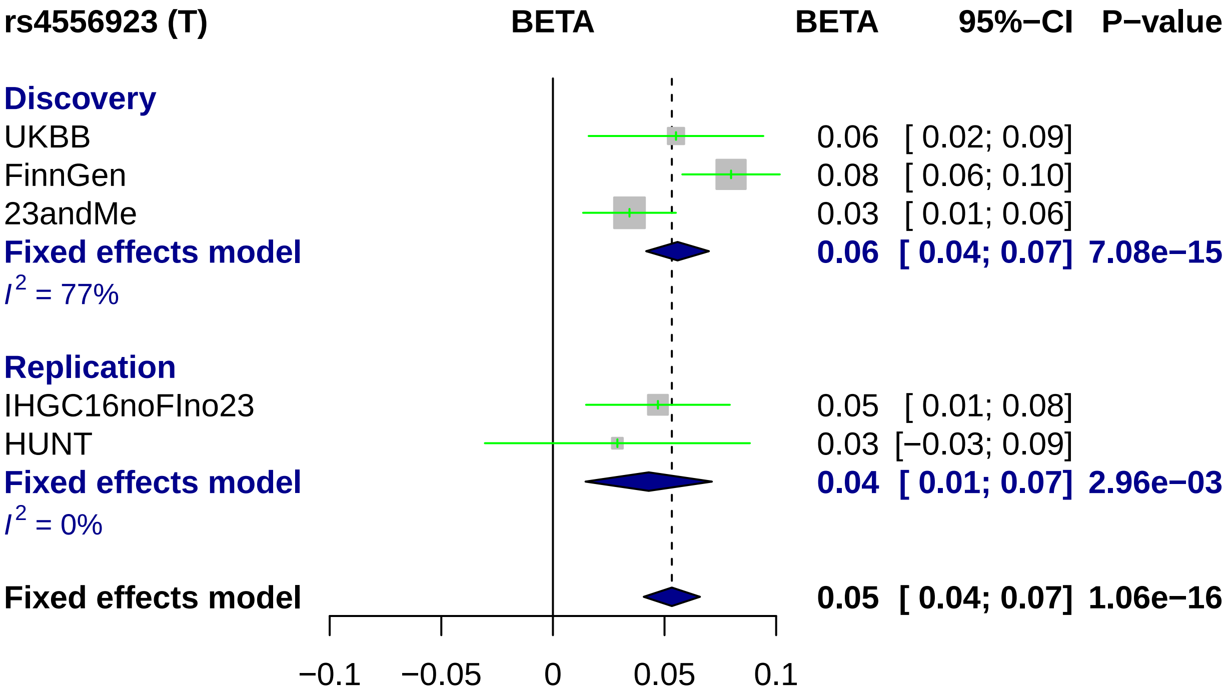
rs4556923 (T)
BETA
BETA
95%−CI
P−value
Discovery
UKBB
0.06 [0.02; 0.09]
FinnGen
0.08 [0.06; 0.10]
23andMe
0.03 [0.01; 0.06]
Fixed effects model
0.06 [0.04; 0.07] 7.08e−15
I2 = 77%
Replication
IHGC16noFIno23
0.05 [0.01; 0.08]
HUNT
0.03 [−0.03; 0.09]
Fixed effects model
0.04 [0.01; 0.07] 2.96e−03
I2 = 0%
Fixed effects model
0.05 [0.04; 0.07] 1.06e−16
−0.1
−0.05
0
0.05
0.1

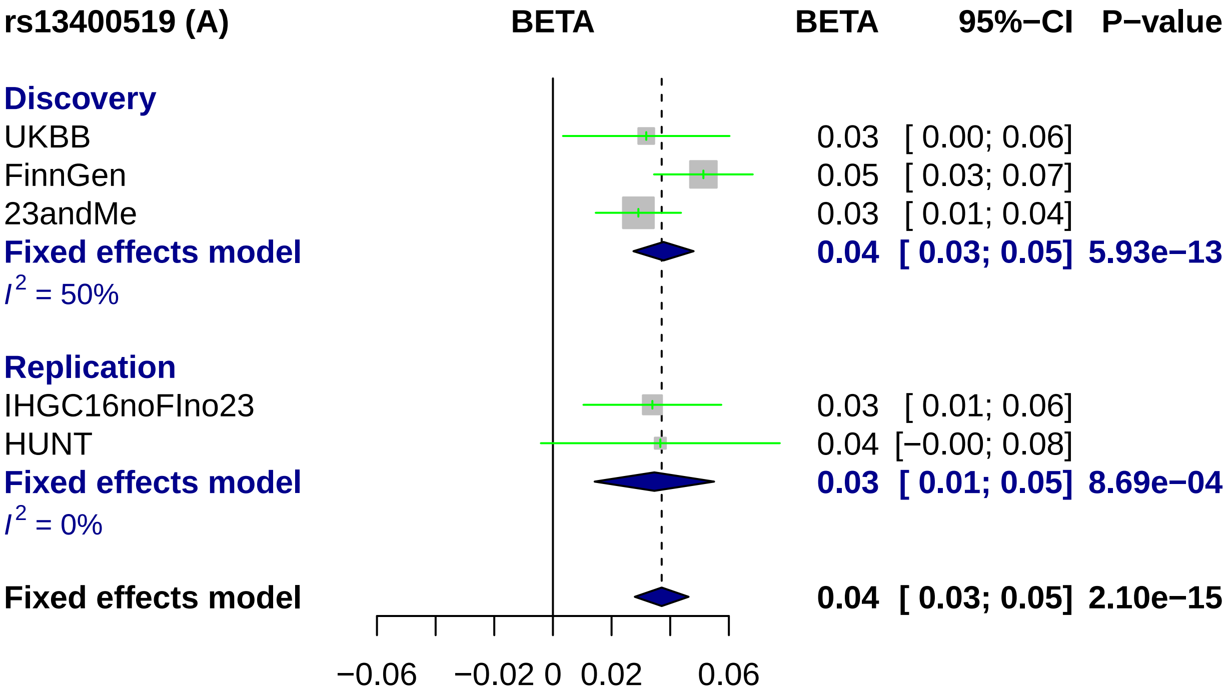
rs13400519 (A)
BETA
BETA
95%−CI
P−value
Discovery
UKBB
0.03
[0.00; 0.06]
FinnGen
0.05
[0.03; 0.07]
23andMe
0.03
[0.01; 0.04]
Fixed effects model
0.04
[0.03; 0.05]
5.93e−13
I^2 = 50%
Replication
IHGC16noFIno23
0.03
[0.01; 0.06]
HUNT
0.04
[−0.00; 0.08]
Fixed effects model
0.03
[0.01; 0.05]
8.69e−04
I^2 = 0%
Fixed effects model
0.04
[0.03; 0.05]
2.10e−15
−0.06
−0.02
0
0.02
0.06

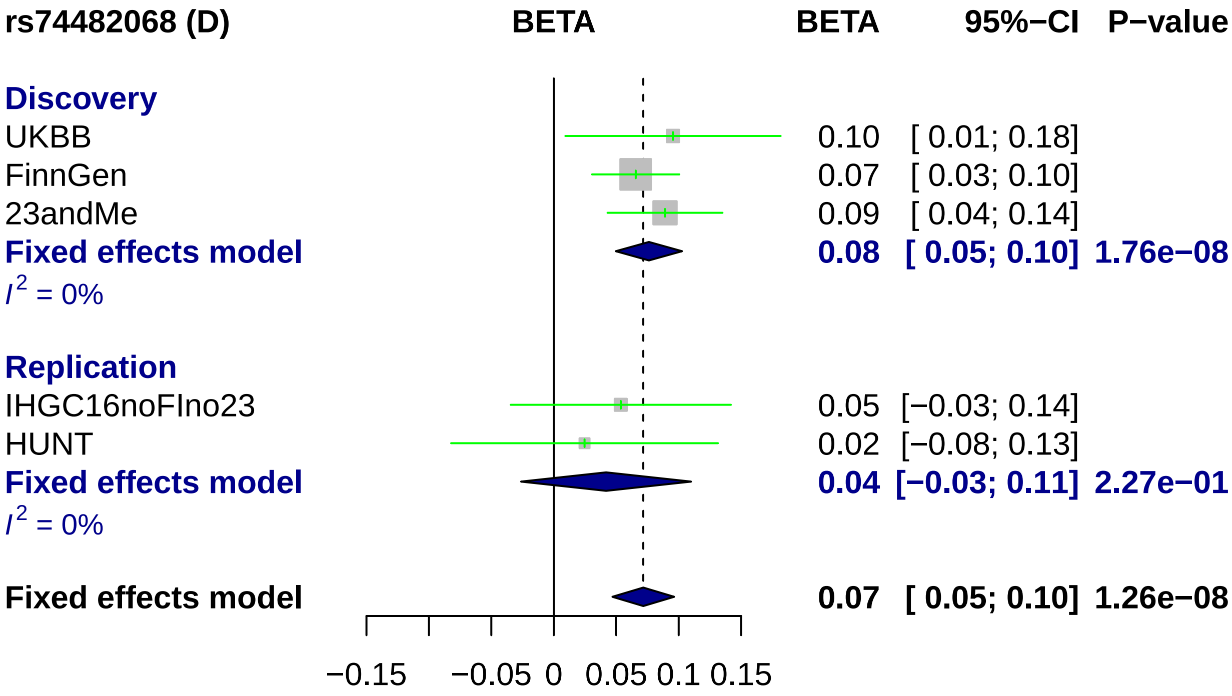
rs74482068 (D)
BETA
BETA
95%−CI
P−value
Discovery
UKBB
0.10
[0.01; 0.18]
FinnGen
0.07
[0.03; 0.10]
23andMe
0.09
[0.04; 0.14]
Fixed effects model
0.08
[0.05; 0.10]
1.76e−08
I² = 0%
Replication
IHGC16noFIno23
0.05
[−0.03; 0.14]
HUNT
0.02
[−0.08; 0.13]
Fixed effects model
0.04
[−0.03; 0.11]
2.27e−01
I² = 0%
Fixed effects model
0.07
[0.05; 0.10]
1.26e−08
−0.15
−0.05
0
0.05
0.1
0.15

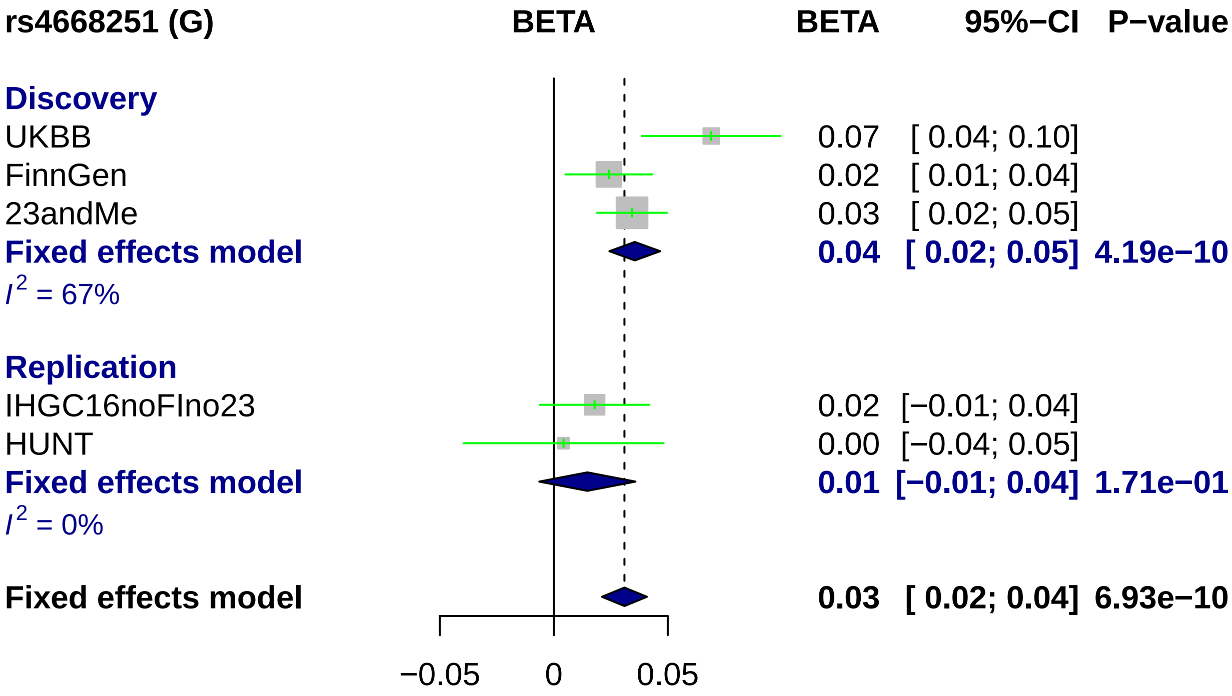
rs4668251 (G)
BETA
BETA
95%−CI
P−value
Discovery
UKBB
0.07
[0.04; 0.10]
FinnGen
0.02
[0.01; 0.04]
23andMe
0.03
[0.02; 0.05]
Fixed effects model
0.04
[0.02; 0.05]
4.19e−10
I^2 = 67%
Replication
IHGC16noFIno23
0.02
[−0.01; 0.04]
HUNT
0.00
[−0.04; 0.05]
Fixed effects model
0.01
[−0.01; 0.04]
1.71e−01
I^2 = 0%
Fixed effects model
0.03
[0.02; 0.04]
6.93e−10
−0.05
0
0.05

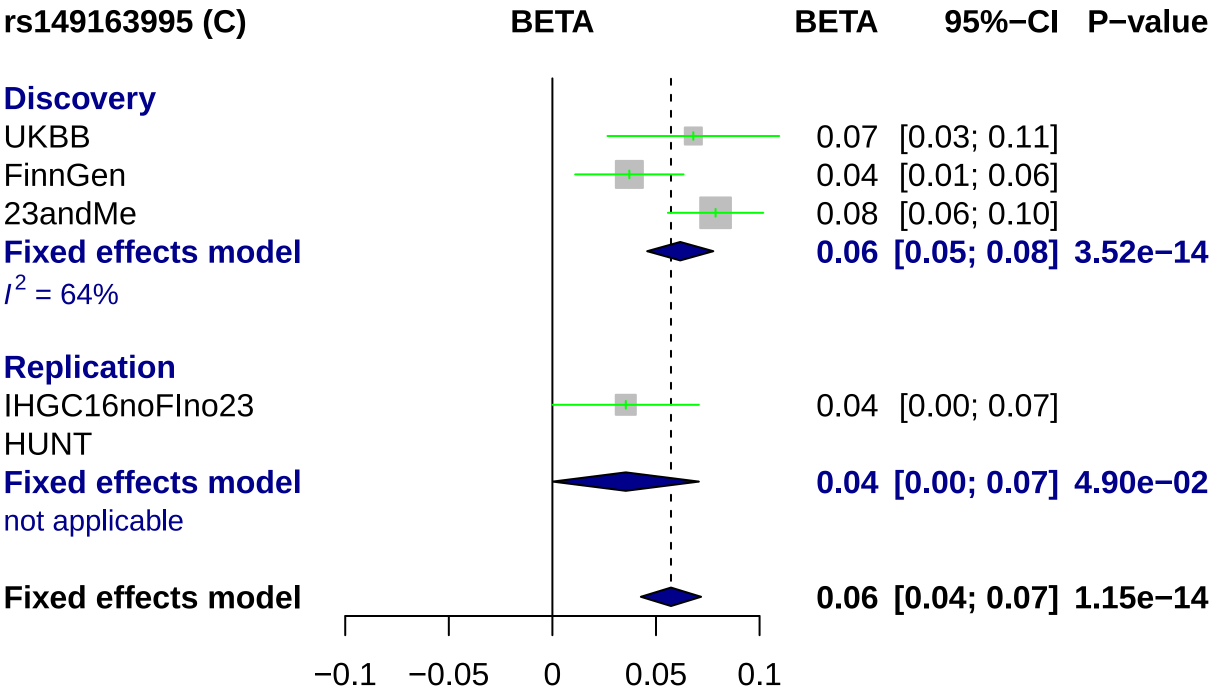
rs149163995 (C)
BETA
BETA
95%−CI
P−value
Discovery
UKBB
0.07 [0.03; 0.11]
FinnGen
0.04 [0.01; 0.06]
23andMe
0.08 [0.06; 0.10]
Fixed effects model
0.06 [0.05; 0.08] 3.52e−14
I^2 = 64%
Replication
IHGC16noFIno23
0.04 [0.00; 0.07]
HUNT
Fixed effects model
0.04 [0.00; 0.07] 4.90e−02
not applicable
Fixed effects model
0.06 [0.04; 0.07] 1.15e−14
−0.1
−0.05
0
0.05
0.1

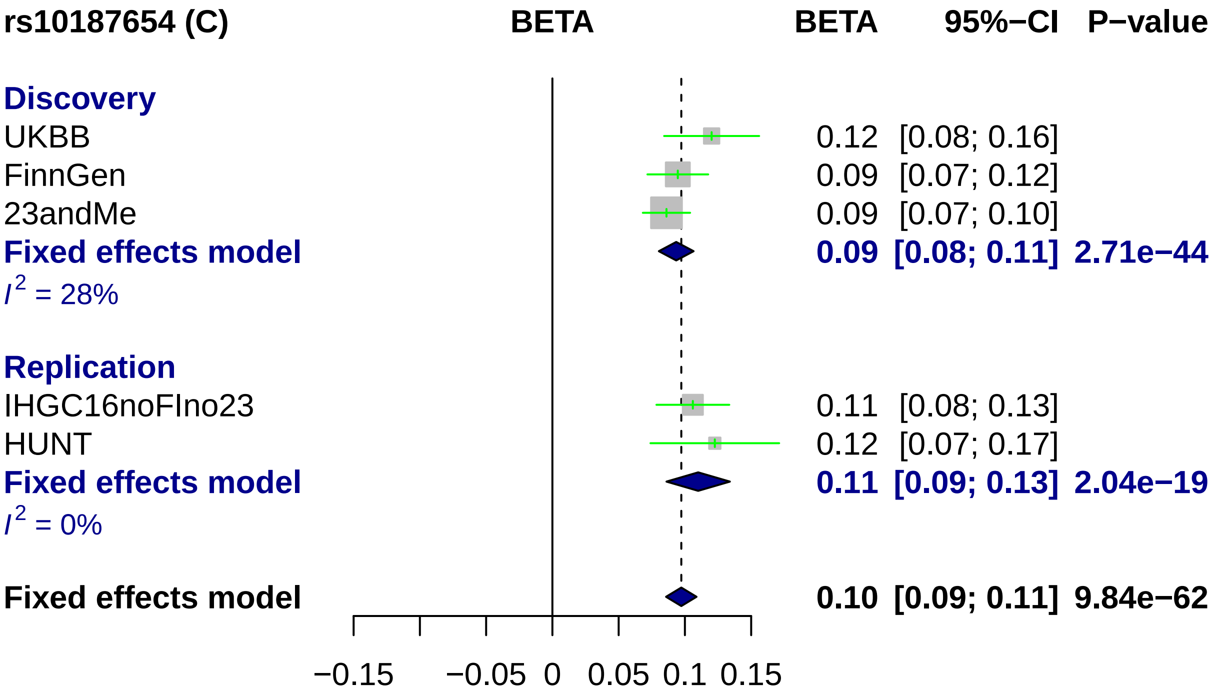
rs10187654 (C)
BETA
BETA
95%−CI
P−value
Discovery
UKBB
0.12 [0.08; 0.16]
FinnGen
0.09 [0.07; 0.12]
23andMe
0.09 [0.07; 0.10]
Fixed effects model
0.09 [0.08; 0.11] 2.71e−44
I^2 = 28%
Replication
IHGC16noFIno23
0.11 [0.08; 0.13]
HUNT
0.12 [0.07; 0.17]
Fixed effects model
0.11 [0.09; 0.13] 2.04e−19
I^2 = 0%
Fixed effects model
0.10 [0.09; 0.11] 9.84e−62
−0.15
−0.05
0
0.05
0.1
0.15

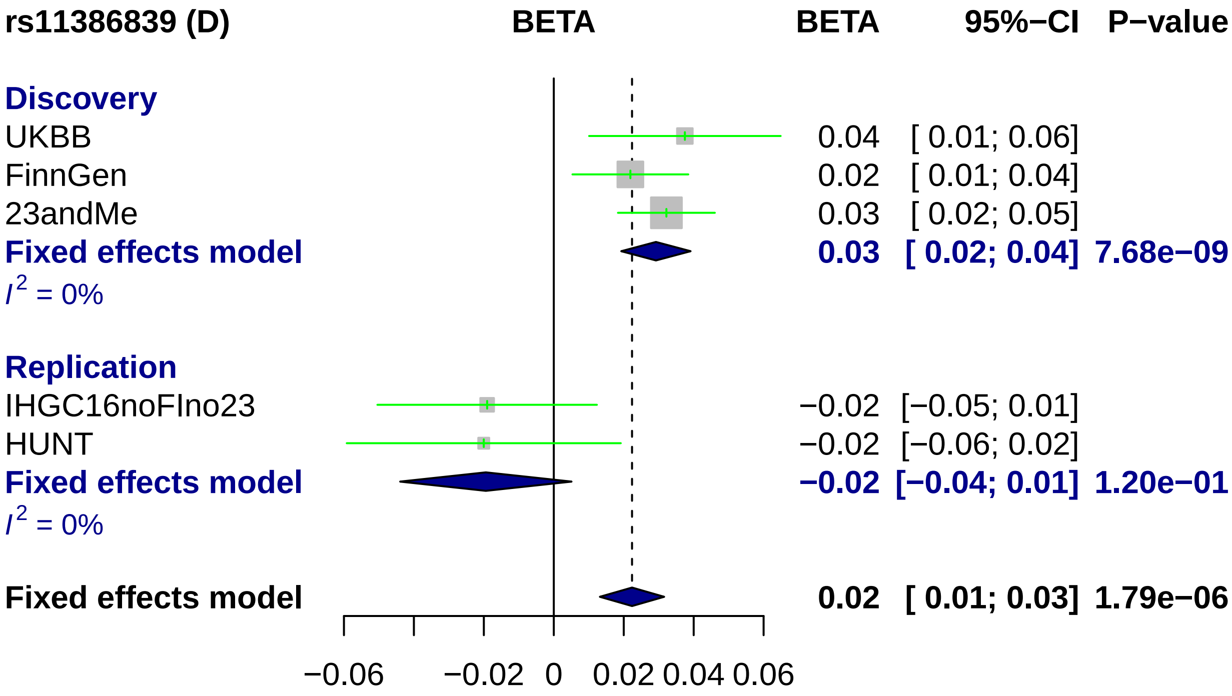
rs11386839 (D)
BETA
BETA
95%−CI
P−value
Discovery
UKBB
0.04
[0.01; 0.06]
FinnGen
0.02
[0.01; 0.04]
23andMe
0.03
[0.02; 0.05]
Fixed effects model
0.03
[0.02; 0.04]
7.68e−09
I2 = 0%
Replication
IHGC16noFIno23
−0.02
[−0.05; 0.01]
HUNT
−0.02
[−0.06; 0.02]
Fixed effects model
−0.02
[−0.04; 0.01]
1.20e−01
I2 = 0%
Fixed effects model
0.02
[0.01; 0.03]
1.79e−06
−0.06
−0.02
0
0.02
0.04
0.06

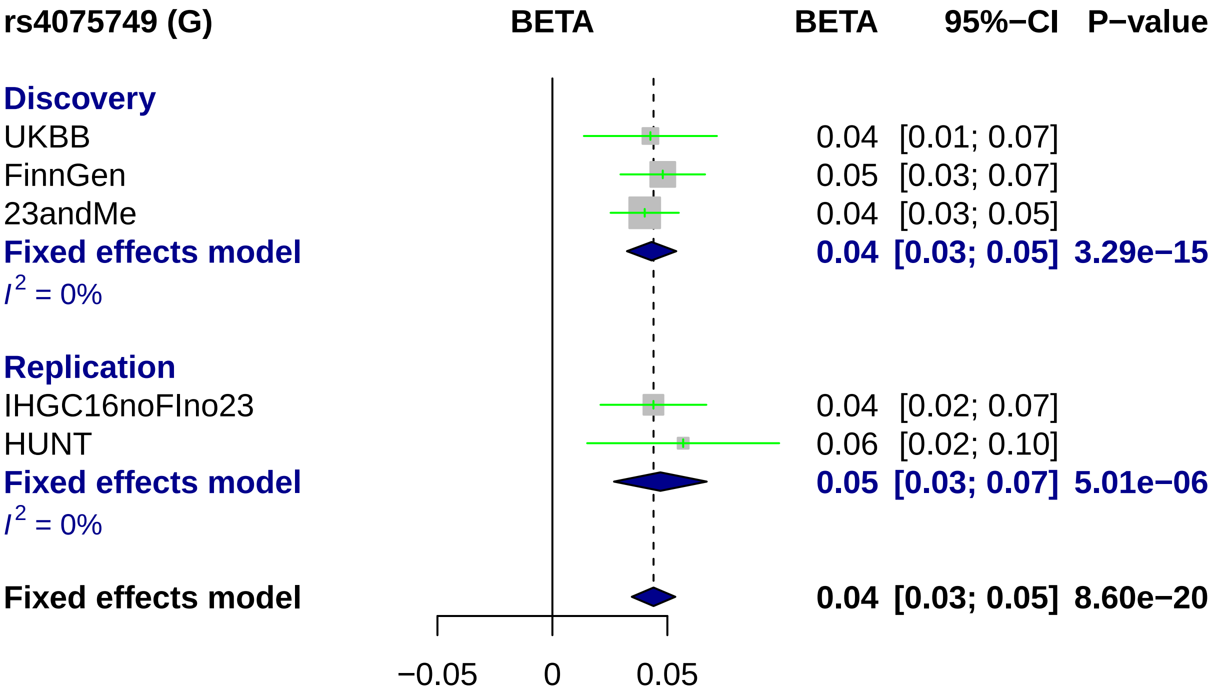
rs4075749 (G)
BETA
BETA 95%−CI P−value
Discovery
UKBB 0.04 [0.01; 0.07]
FinnGen 0.05 [0.03; 0.07]
23andMe 0.04 [0.03; 0.05]
Fixed effects model 0.04 [0.03; 0.05] 3.29e−15
I^2 = 0%
Replication
IHGC16noFIno23 0.04 [0.02; 0.07]
HUNT 0.06 [0.02; 0.10]
Fixed effects model 0.05 [0.03; 0.07] 5.01e−06
I^2 = 0%
Fixed effects model 0.04 [0.03; 0.05] 8.60e−20
−0.05
0
0.05

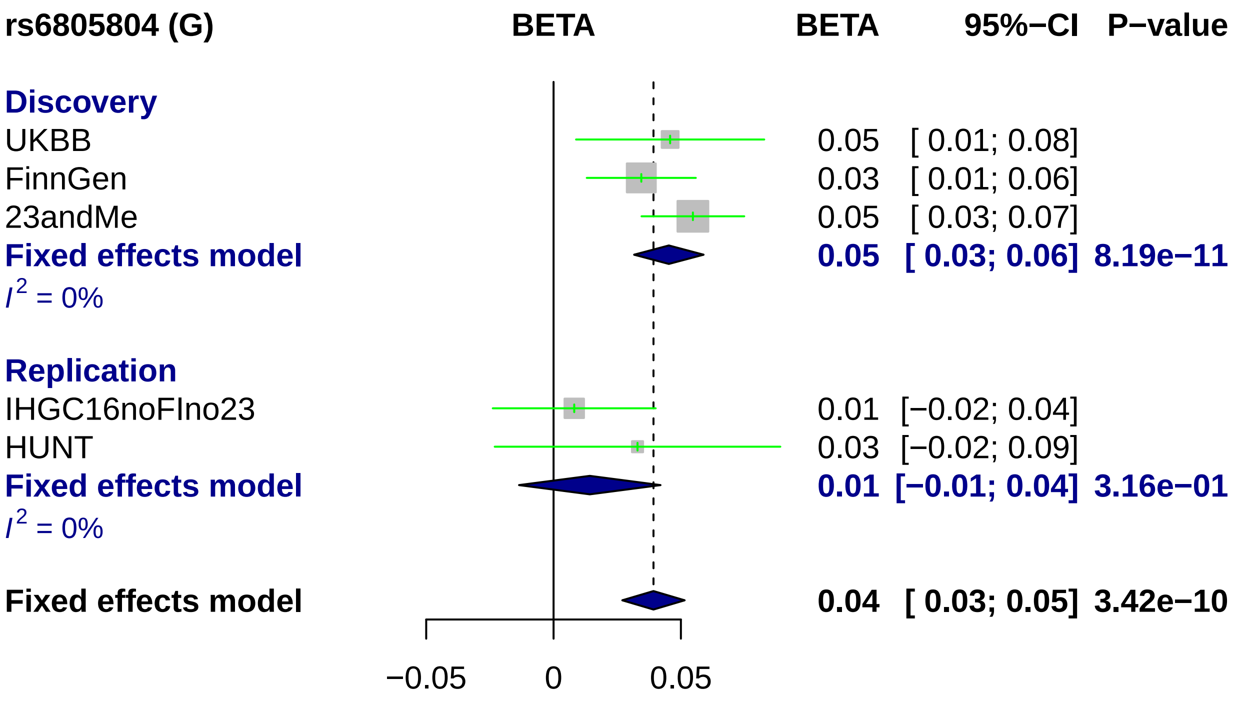
rs6805804 (G)
BETA
BETA
95%−CI
P−value
Discovery
UKBB
0.05
[0.01; 0.08]
FinnGen
0.03
[0.01; 0.06]
23andMe
0.05
[0.03; 0.07]
Fixed effects model
0.05
[0.03; 0.06]
8.19e−11
I^2 = 0%
Replication
IHGC16noFIno23
0.01
[−0.02; 0.04]
HUNT
0.03
[−0.02; 0.09]
Fixed effects model
0.01
[−0.01; 0.04]
3.16e−01
I^2 = 0%
Fixed effects model
0.04
[0.03; 0.05]
3.42e−10
−0.05
0
0.05

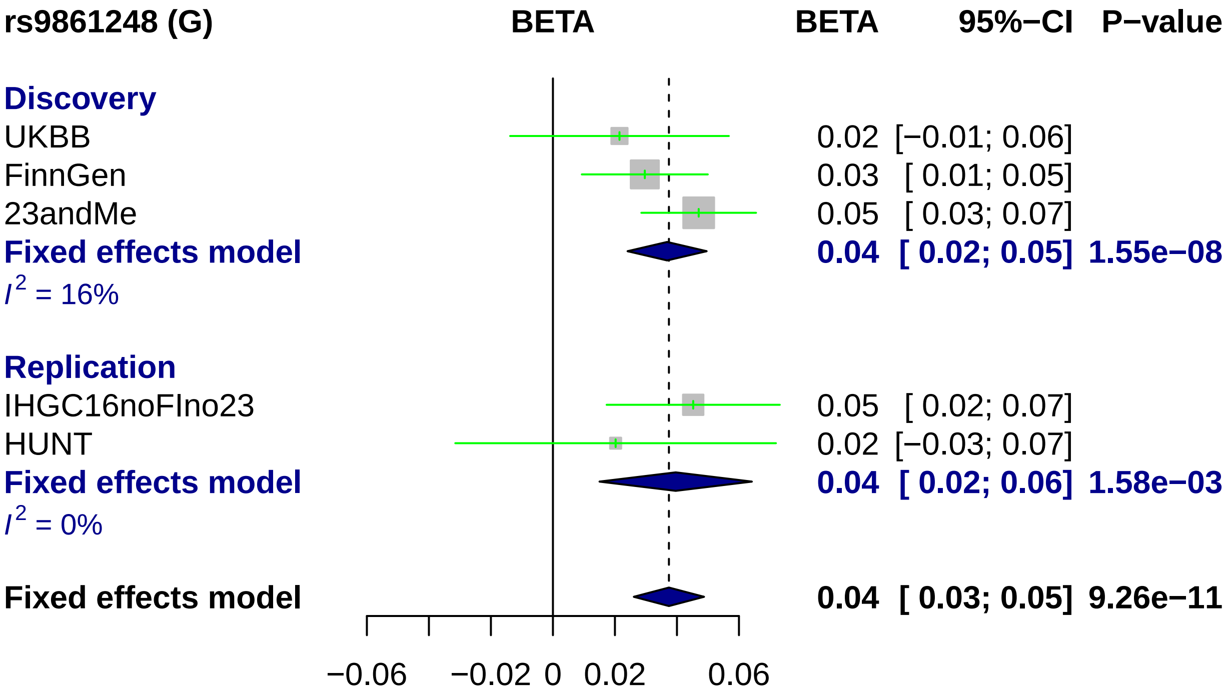
rs9861248 (G)
BETA
BETA
95%−CI
P−value
Discovery
UKBB
0.02 [−0.01; 0.06]
FinnGen
0.03 [0.01; 0.05]
23andMe
0.05 [0.03; 0.07]
Fixed effects model
0.04 [0.02; 0.05] 1.55e−08
I2 = 16%
Replication
IHGC16noFIno23
0.05 [0.02; 0.07]
HUNT
0.02 [−0.03; 0.07]
Fixed effects model
0.04 [0.02; 0.06] 1.58e−03
I2 = 0%
Fixed effects model
0.04 [0.03; 0.05] 9.26e−11
−0.06
−0.02
0
0.02
0.06

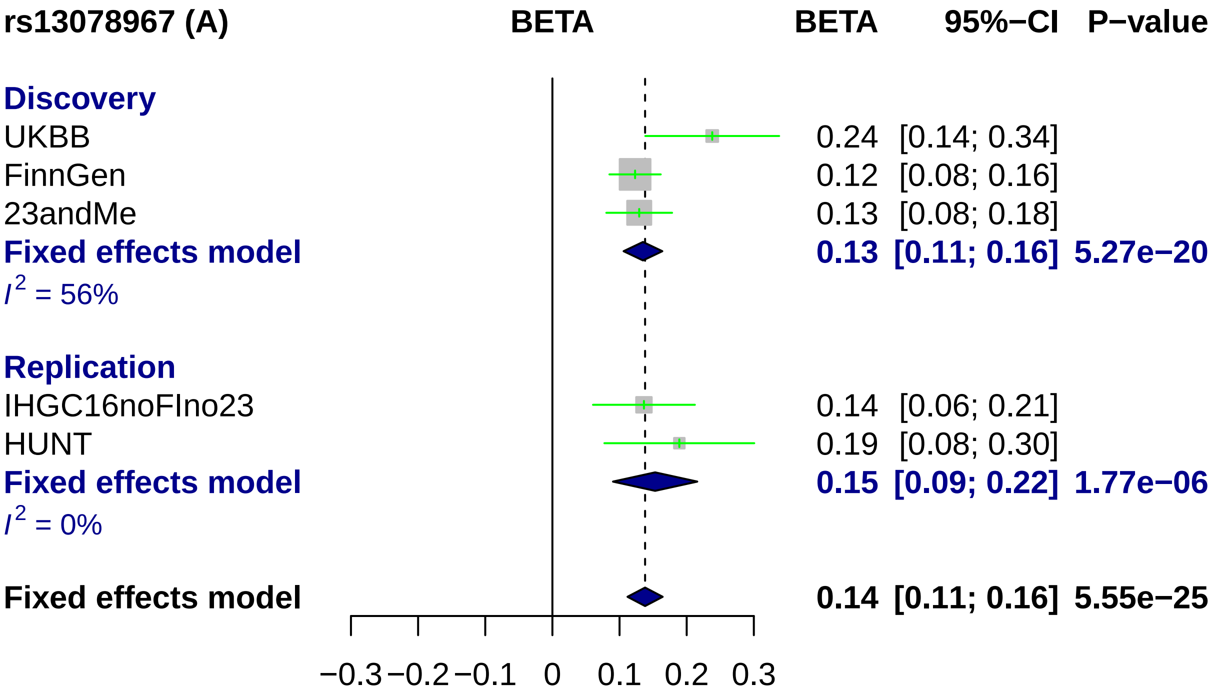
rs13078967 (A)
BETA
BETA
95%−CI
P−value
Discovery
UKBB
0.24
[0.14; 0.34]
FinnGen
0.12
[0.08; 0.16]
23andMe
0.13
[0.08; 0.18]
Fixed effects model
0.13
[0.11; 0.16]
5.27e−20
I^2 = 56%
Replication
IHGC16noFIno23
0.14
[0.06; 0.21]
HUNT
0.19
[0.08; 0.30]
Fixed effects model
0.15
[0.09; 0.22]
1.77e−06
I^2 = 0%
Fixed effects model
0.14
[0.11; 0.16]
5.55e−25
−0.3
−0.2
−0.1
0
0.1
0.2
0.3

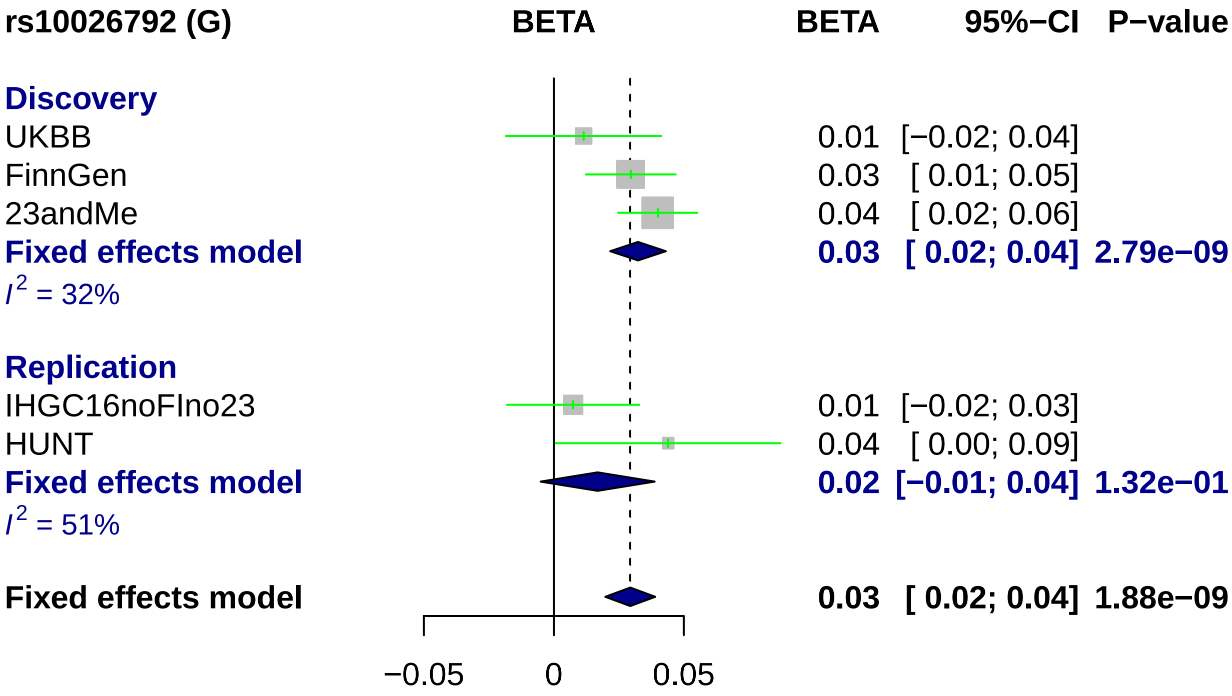
rs10026792 (G)
BETA
BETA
95%−CI
P−value
Discovery
UKBB
0.01 [−0.02; 0.04]
FinnGen
0.03 [0.01; 0.05]
23andMe
0.04 [0.02; 0.06]
Fixed effects model
0.03 [0.02; 0.04] 2.79e−09
I^2 = 32%
Replication
IHGC16noFIno23
0.01 [−0.02; 0.03]
HUNT
0.04 [0.00; 0.09]
Fixed effects model
0.02 [−0.01; 0.04] 1.32e−01
I^2 = 51%
Fixed effects model
0.03 [0.02; 0.04] 1.88e−09
−0.05
0
0.05

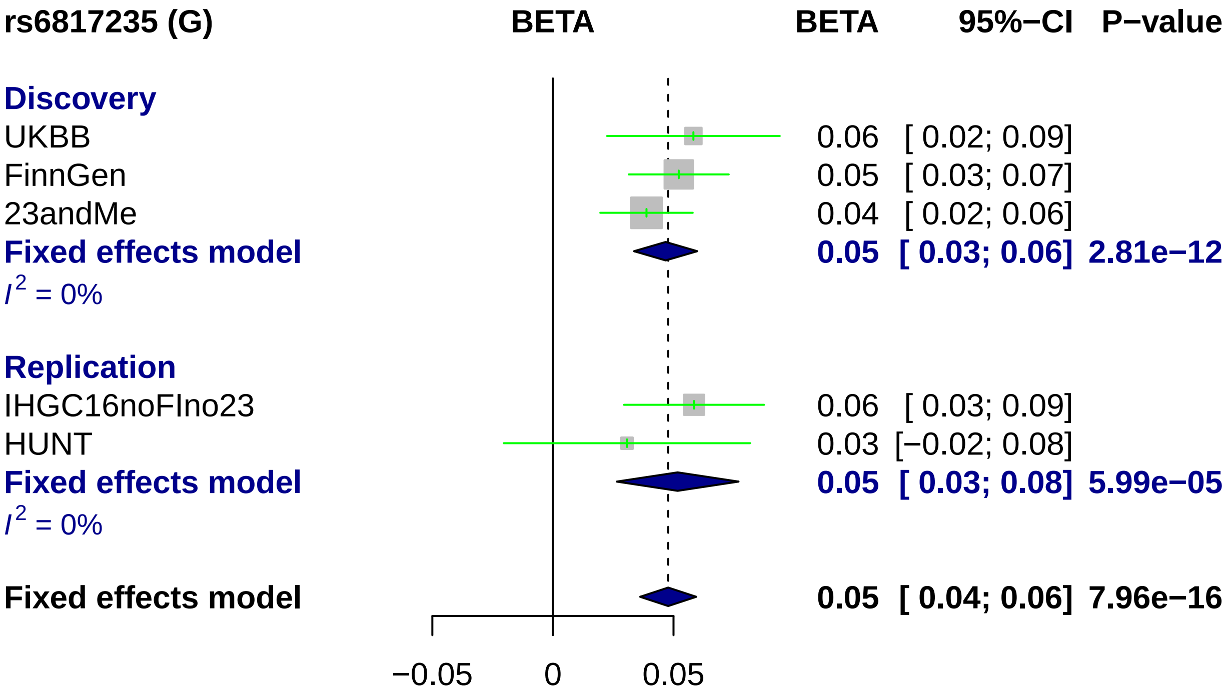
rs6817235 (G)
BETA
BETA
95%−CI
P−value
Discovery
UKBB
0.06 [0.02; 0.09]
FinnGen
0.05 [0.03; 0.07]
23andMe
0.04 [0.02; 0.06]
Fixed effects model
0.05 [0.03; 0.06] 2.81e−12
I^2 = 0%
Replication
IHGC16noFIno23
0.06 [0.03; 0.09]
HUNT
0.03 [−0.02; 0.08]
Fixed effects model
0.05 [0.03; 0.08] 5.99e−05
I^2 = 0%
Fixed effects model
0.05 [0.04; 0.06] 7.96e−16
−0.05
0
0.05

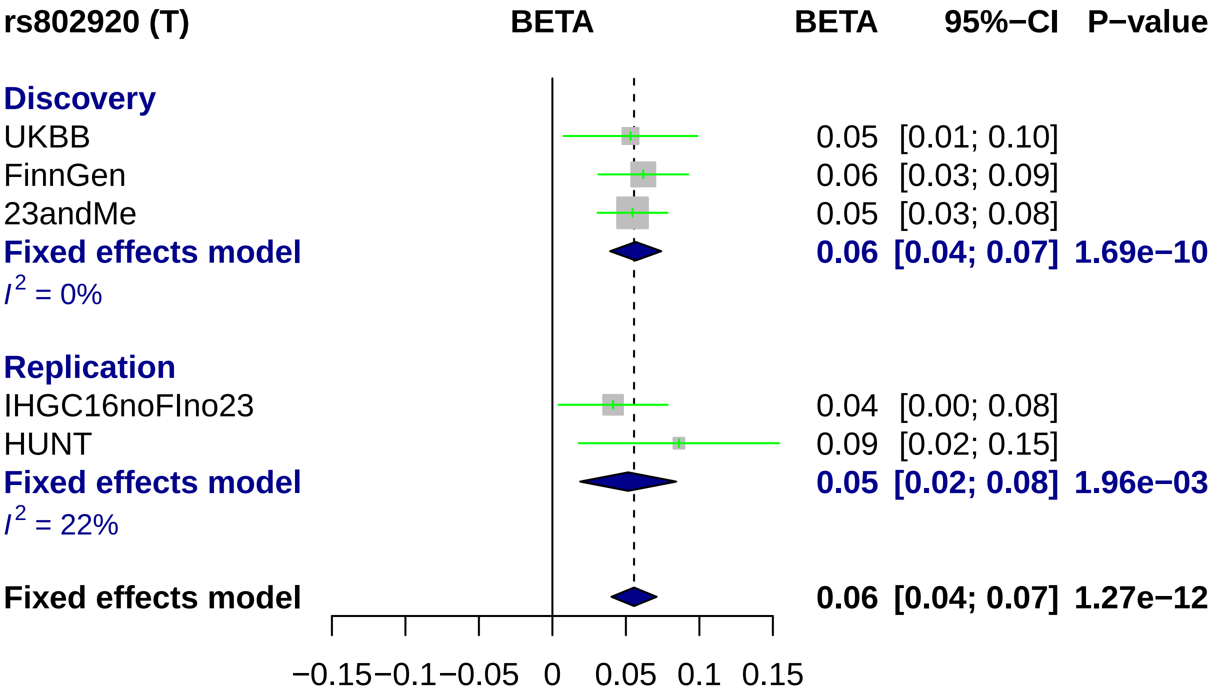
rs802920 (T)
BETA
BETA
95%−CI
P−value
Discovery
UKBB
0.05 [0.01; 0.10]
FinnGen
0.06 [0.03; 0.09]
23andMe
0.05 [0.03; 0.08]
Fixed effects model
0.06 [0.04; 0.07] 1.69e−10
I2 = 0%
Replication
IHGC16noFIno23
0.04 [0.00; 0.08]
HUNT
0.09 [0.02; 0.15]
Fixed effects model
0.05 [0.02; 0.08] 1.96e−03
I2 = 22%
Fixed effects model
0.06 [0.04; 0.07] 1.27e−12
−0.15
−0.1
−0.05
0
0.05
0.1
0.15

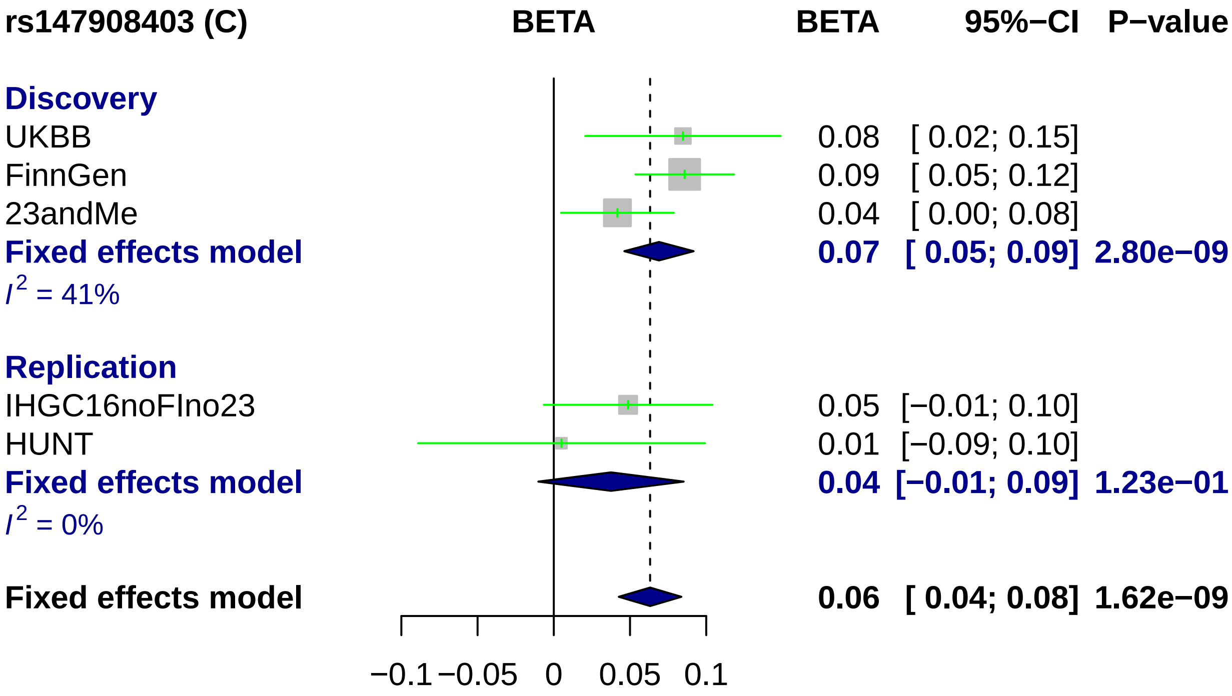

rs147908403 (C)
BETA
BETA
95%−CI
P−value
Discovery
UKBB
0.08
[0.02; 0.15]
FinnGen
0.09
[0.05; 0.12]
23andMe
0.04
[0.00; 0.08]
Fixed effects model
0.07
[0.05; 0.09]
2.80e−09
I^2 = 41%
Replication
IHGC16noFIno23
0.05
[−0.01; 0.10]
HUNT
0.01
[−0.09; 0.10]
Fixed effects model
0.04
[−0.01; 0.09]
1.23e−01
I^2 = 0%
Fixed effects model
0.06
[0.04; 0.08]
1.62e−09
−0.1
−0.05
0
0.05
0.1

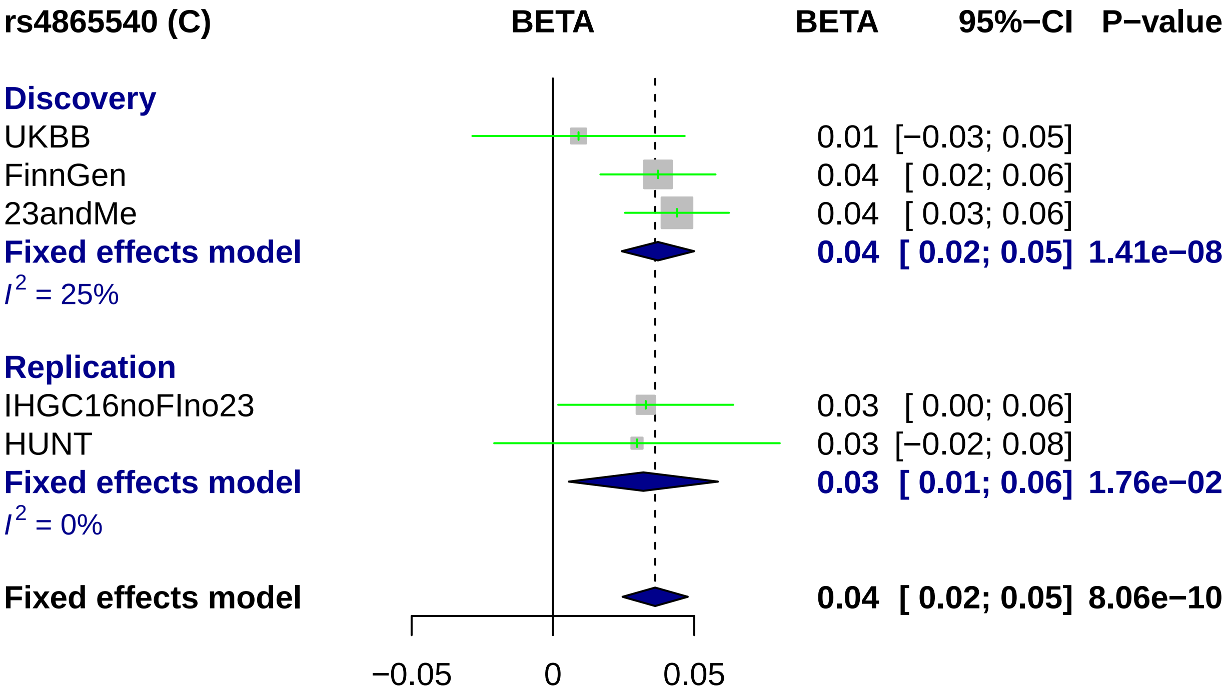
rs4865540 (C)
BETA
BETA
95%−CI
P−value
Discovery
UKBB
0.01 [−0.03; 0.05]
FinnGen
0.04 [0.02; 0.06]
23andMe
0.04 [0.03; 0.06]
Fixed effects model
0.04 [0.02; 0.05] 1.41e−08
I2 = 25%
Replication
IHGC16noFIno23
0.03 [0.00; 0.06]
HUNT
0.03 [−0.02; 0.08]
Fixed effects model
0.03 [0.01; 0.06] 1.76e−02
I2 = 0%
Fixed effects model
0.04 [0.02; 0.05] 8.06e−10
−0.05
0
0.05

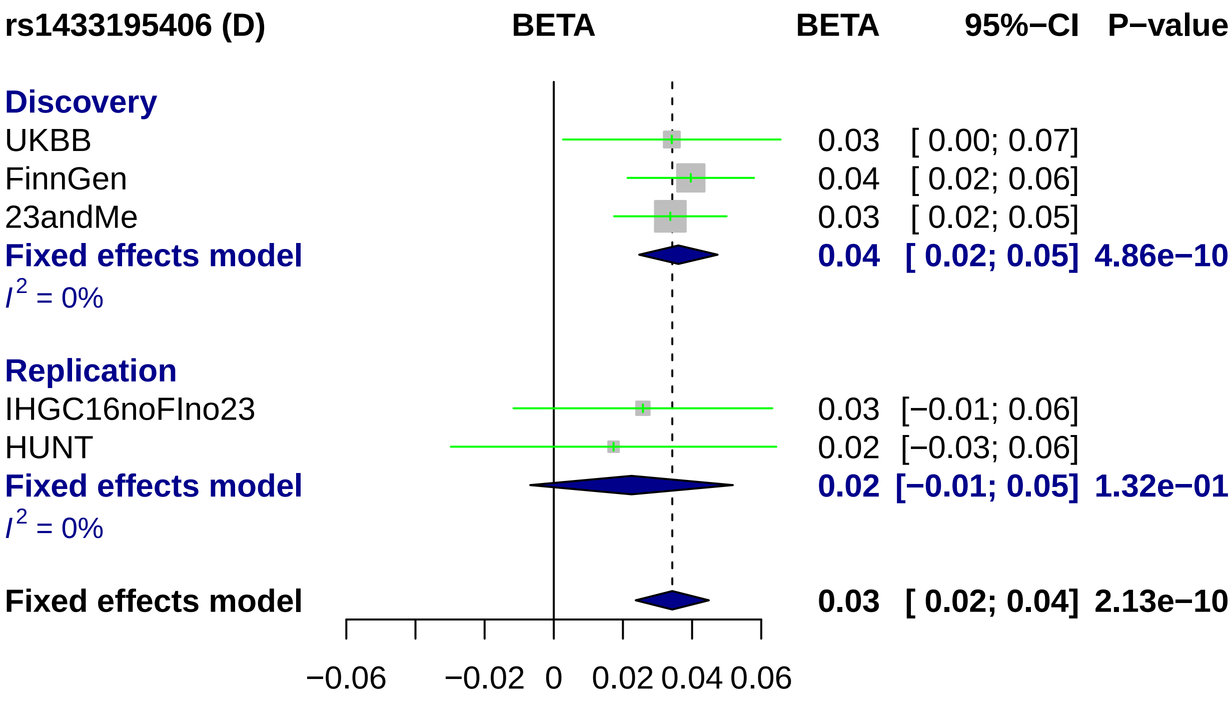
rs1433195406 (D)
BETA
BETA
95%−CI
P−value
Discovery
UKBB
0.03 [0.00; 0.07]
FinnGen
0.04 [0.02; 0.06]
23andMe
0.03 [0.02; 0.05]
Fixed effects model
0.04 [0.02; 0.05] 4.86e−10
I2 = 0%
Replication
IHGC16noFIno23
0.03 [−0.01; 0.06]
HUNT
0.02 [−0.03; 0.06]
Fixed effects model
0.02 [−0.01; 0.05] 1.32e−01
I2 = 0%
Fixed effects model
0.03 [0.02; 0.04] 2.13e−10
−0.06
−0.02
0
0.02
0.04
0.06

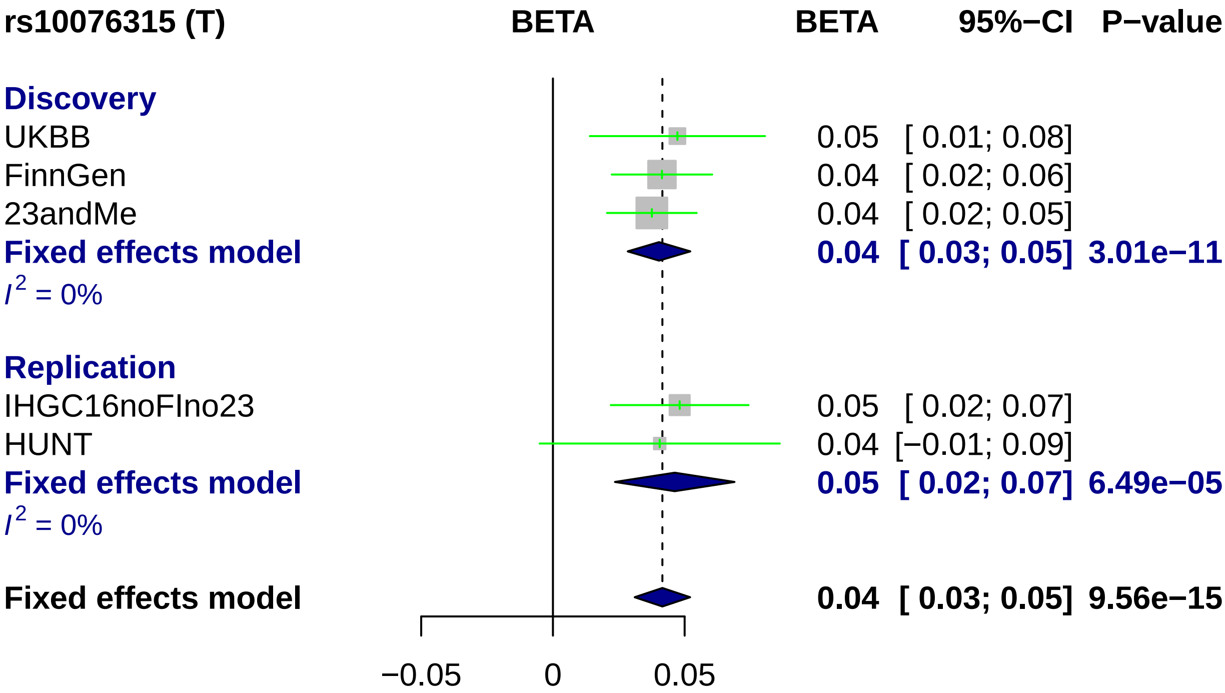
rs10076315 (T)
BETA
BETA
95%−CI
P−value
Discovery
UKBB
0.05 [0.01; 0.08]
FinnGen
0.04 [0.02; 0.06]
23andMe
0.04 [0.02; 0.05]
Fixed effects model
0.04 [0.03; 0.05] 3.01e−11
I2 = 0%
Replication
IHGC16noFIno23
0.05 [0.02; 0.07]
HUNT
0.04 [−0.01; 0.09]
Fixed effects model
0.05 [0.02; 0.07] 6.49e−05
I2 = 0%
Fixed effects model
0.04 [0.03; 0.05] 9.56e−15
−0.05
0
0.05

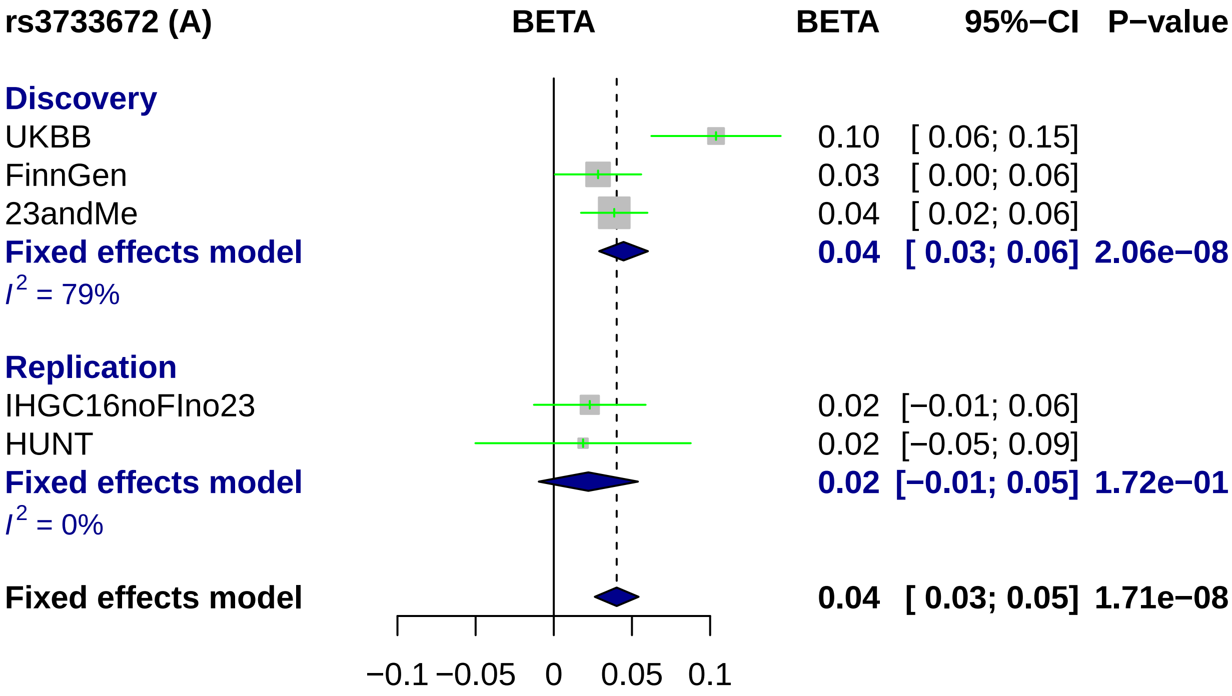
rs3733672 (A)
BETA
BETA
95%−CI
P−value
Discovery
UKBB 0.10 [0.06; 0.15]
FinnGen 0.03 [0.00; 0.06]
23andMe 0.04 [0.02; 0.06]
Fixed effects model 0.04 [0.03; 0.06] 2.06e−08
I2 = 79%
Replication
IHGC16noFIno23 0.02 [−0.01; 0.06]
HUNT 0.02 [−0.05; 0.09]
Fixed effects model 0.02 [−0.01; 0.05] 1.72e−01
I2 = 0%
Fixed effects model 0.04 [0.03; 0.05] 1.71e−08
−0.1
−0.05
0
0.05
0.1

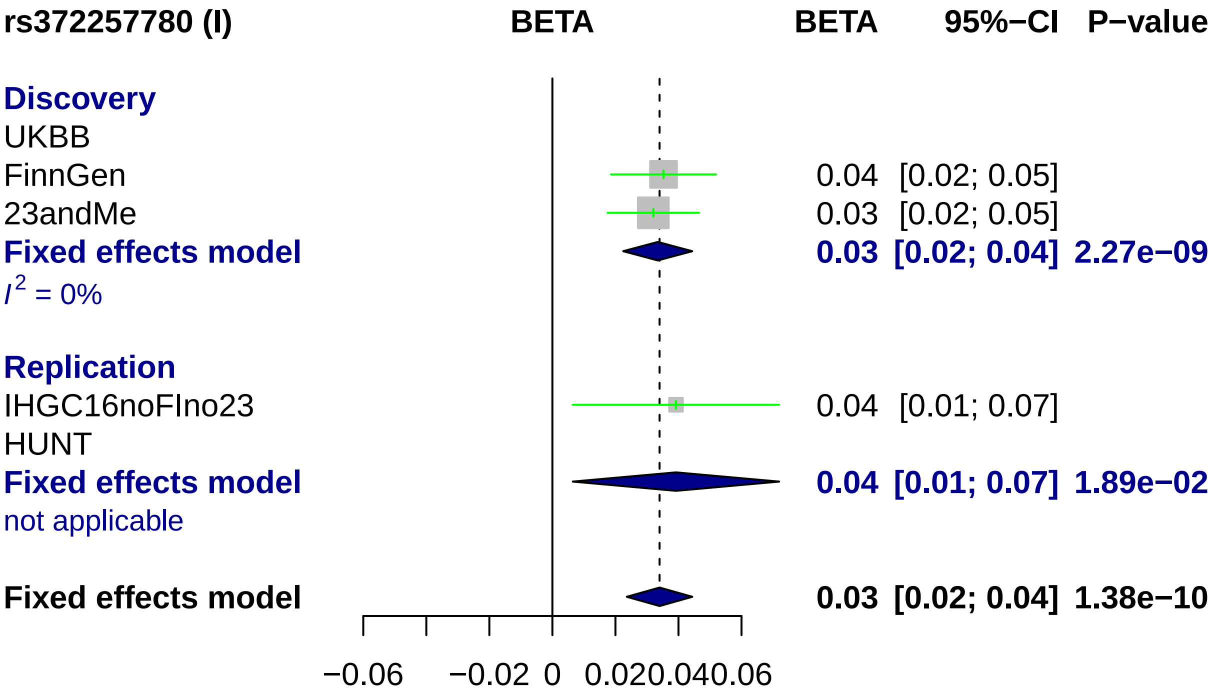
rs372257780 (I)
BETA
BETA
95%−CI
P−value
Discovery
UKBB
FinnGen
0.04 [0.02; 0.05]
23andMe
0.03 [0.02; 0.05]
Fixed effects model
0.03 [0.02; 0.04] 2.27e−09
I^2 = 0%
Replication
IHGC16noFIno23
0.04 [0.01; 0.07]
HUNT
Fixed effects model
0.04 [0.01; 0.07] 1.89e−02
not applicable
Fixed effects model
0.03 [0.02; 0.04] 1.38e−10
−0.06
−0.02
0
0.02
0.04
0.06

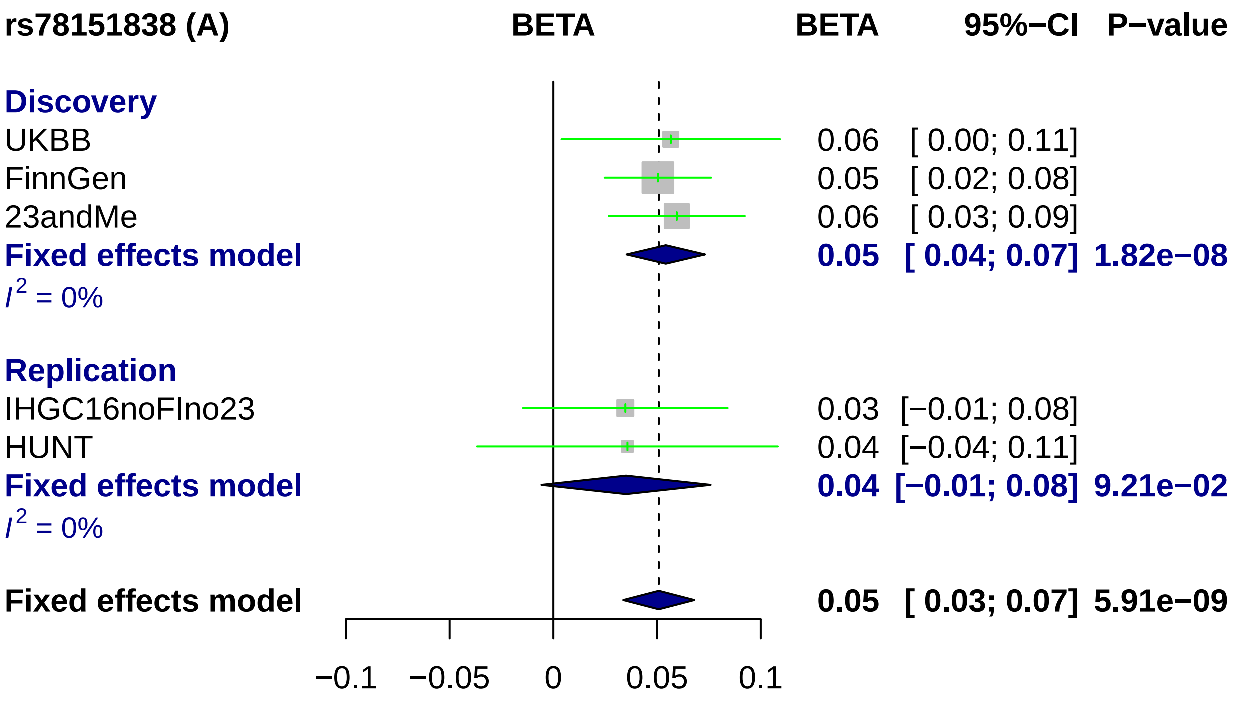
rs78151838 (A)
BETA
BETA
95%−CI
P−value
Discovery
UKBB
FinnGen
23andMe
Fixed effects model
I2 = 0%
Replication
IHGC16noFIno23
HUNT
Fixed effects model
I2 = 0%
Fixed effects model
0.06 [0.00; 0.11]
0.05 [0.02; 0.08]
0.06 [0.03; 0.09]
0.05 [0.04; 0.07] 1.82e−08
0.03 [−0.01; 0.08]
0.04 [−0.04; 0.11]
0.04 [−0.01; 0.08] 9.21e−02
0.05 [0.03; 0.07] 5.91e−09
−0.1
−0.05
0
0.05
0.1

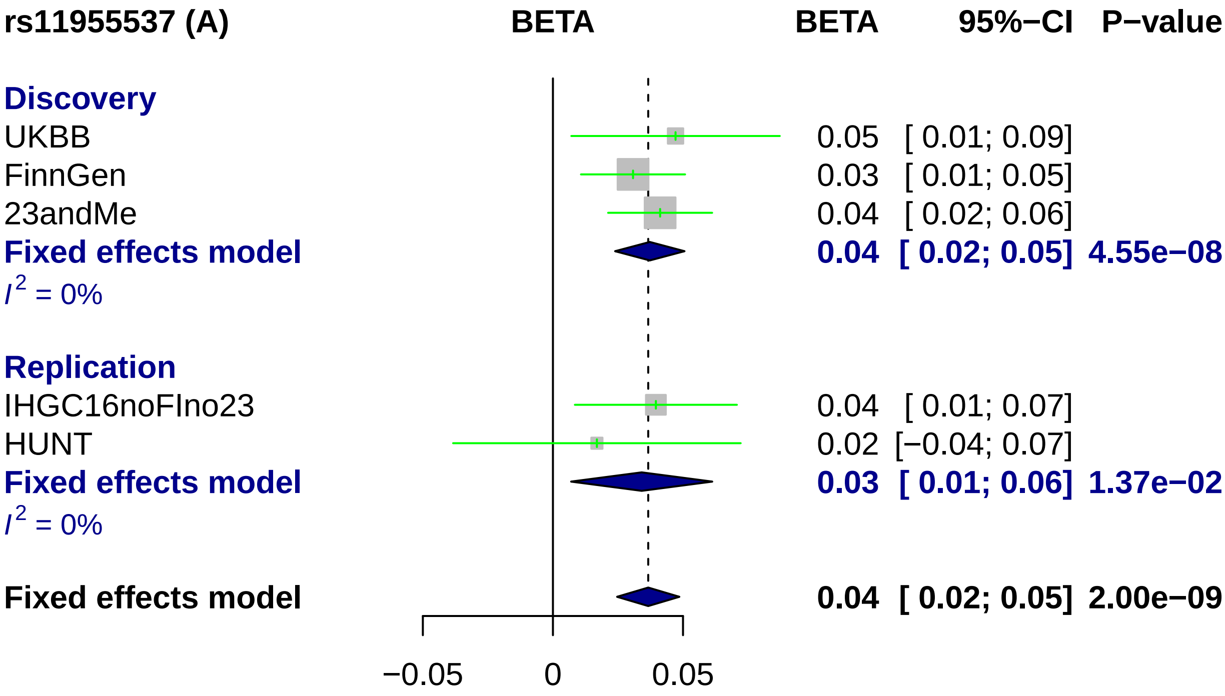
rs11955537 (A)
BETA
BETA
95%−CI
P−value
Discovery
UKBB 0.05 [0.01; 0.09]
FinnGen 0.03 [0.01; 0.05]
23andMe 0.04 [0.02; 0.06]
Fixed effects model 0.04 [0.02; 0.05] 4.55e−08
I^2 = 0%
Replication
IHGC16noFIno23 0.04 [0.01; 0.07]
HUNT 0.02 [−0.04; 0.07]
Fixed effects model 0.03 [0.01; 0.06] 1.37e−02
I^2 = 0%
Fixed effects model 0.04 [0.02; 0.05] 2.00e−09
−0.05
0
0.05

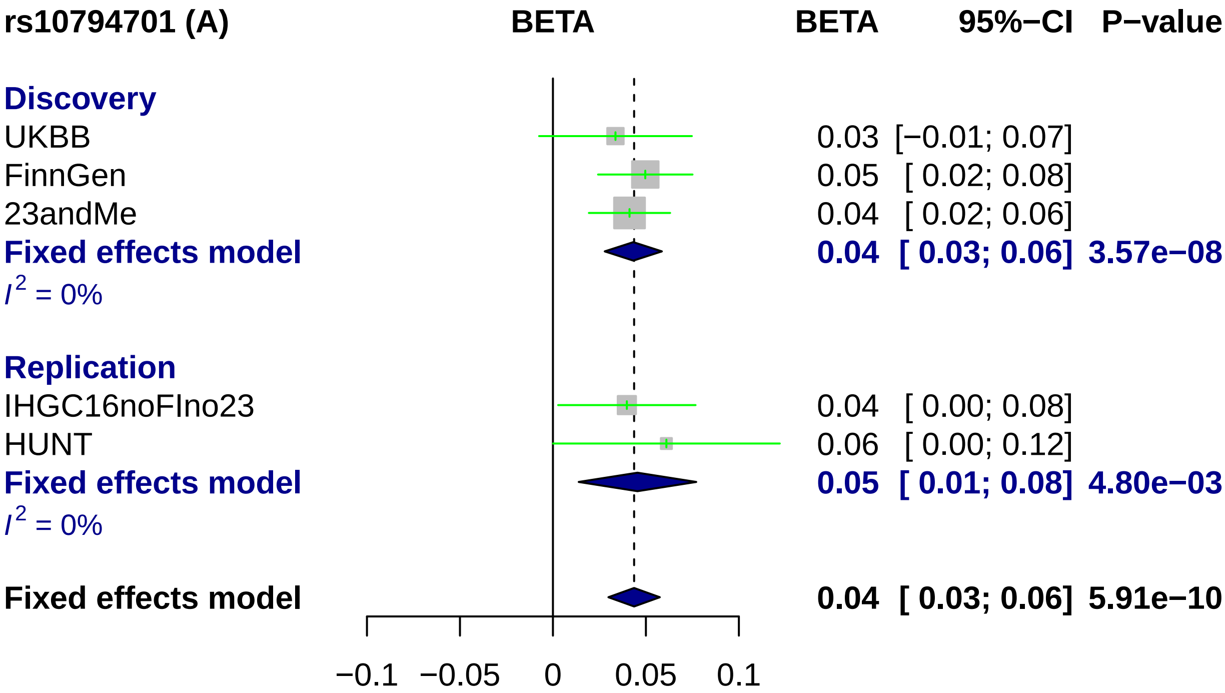
rs10794701 (A)
BETA
BETA
95%−CI
P−value
Discovery
UKBB
0.03
[−0.01; 0.07]
FinnGen
0.05
[0.02; 0.08]
23andMe
0.04
[0.02; 0.06]
Fixed effects model
0.04
[0.03; 0.06]
3.57e−08
I2 = 0%
Replication
IHGC16noFIno23
0.04
[0.00; 0.08]
HUNT
0.06
[0.00; 0.12]
Fixed effects model
0.05
[0.01; 0.08]
4.80e−03
I2 = 0%
Fixed effects model
0.04
[0.03; 0.06]
5.91e−10
−0.1
−0.05
0
0.05
0.1

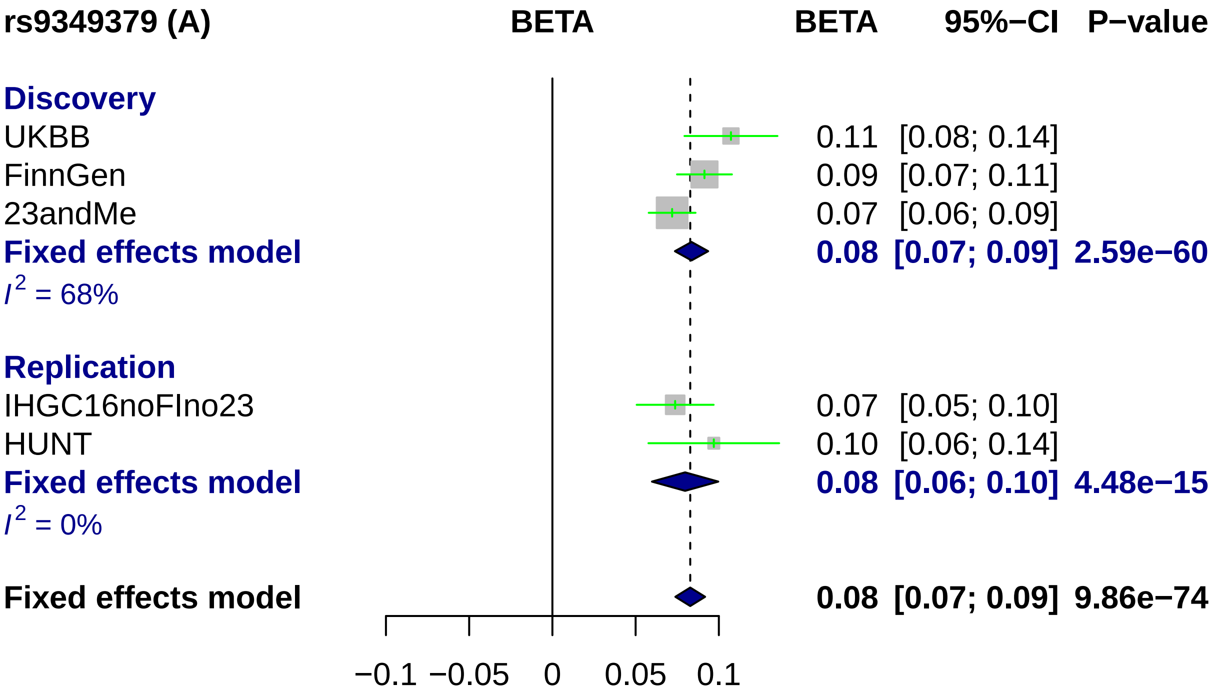
rs9349379 (A)
BETA
BETA
95%−CI
P−value
Discovery
UKBB
0.11
[0.08; 0.14]
FinnGen
0.09
[0.07; 0.11]
23andMe
0.07
[0.06; 0.09]
Fixed effects model
0.08
[0.07; 0.09]
2.59e−60
I^2 = 68%
Replication
IHGC16noFIno23
0.07
[0.05; 0.10]
HUNT
0.10
[0.06; 0.14]
Fixed effects model
0.08
[0.06; 0.10]
4.48e−15
I^2 = 0%
Fixed effects model
0.08
[0.07; 0.09]
9.86e−74
−0.1
−0.05
0
0.05
0.1

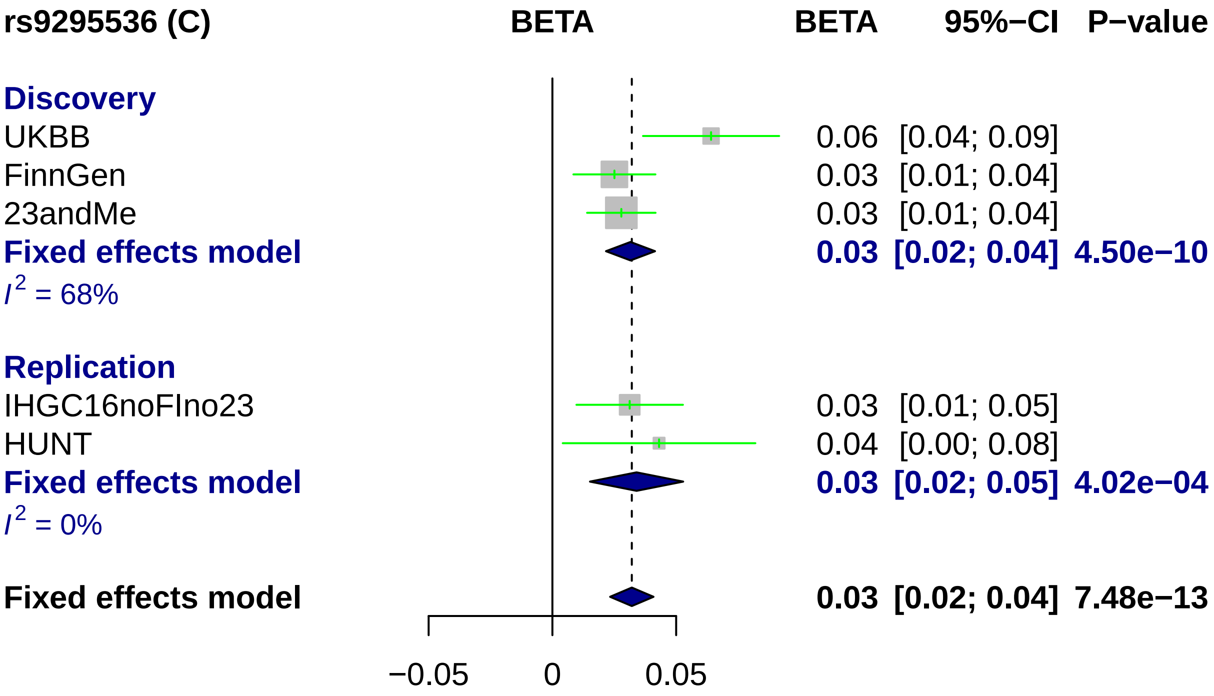
rs9295536 (C)
BETA
BETA
95%−CI
P−value
Discovery
UKBB
0.06 [0.04; 0.09]
FinnGen
0.03 [0.01; 0.04]
23andMe
0.03 [0.01; 0.04]
Fixed effects model
0.03 [0.02; 0.04] 4.50e−10
I2 = 68%
Replication
IHGC16noFIno23
0.03 [0.01; 0.05]
HUNT
0.04 [0.00; 0.08]
Fixed effects model
0.03 [0.02; 0.05] 4.02e−04
I2 = 0%
Fixed effects model
0.03 [0.02; 0.04] 7.48e−13
−0.05
0
0.05

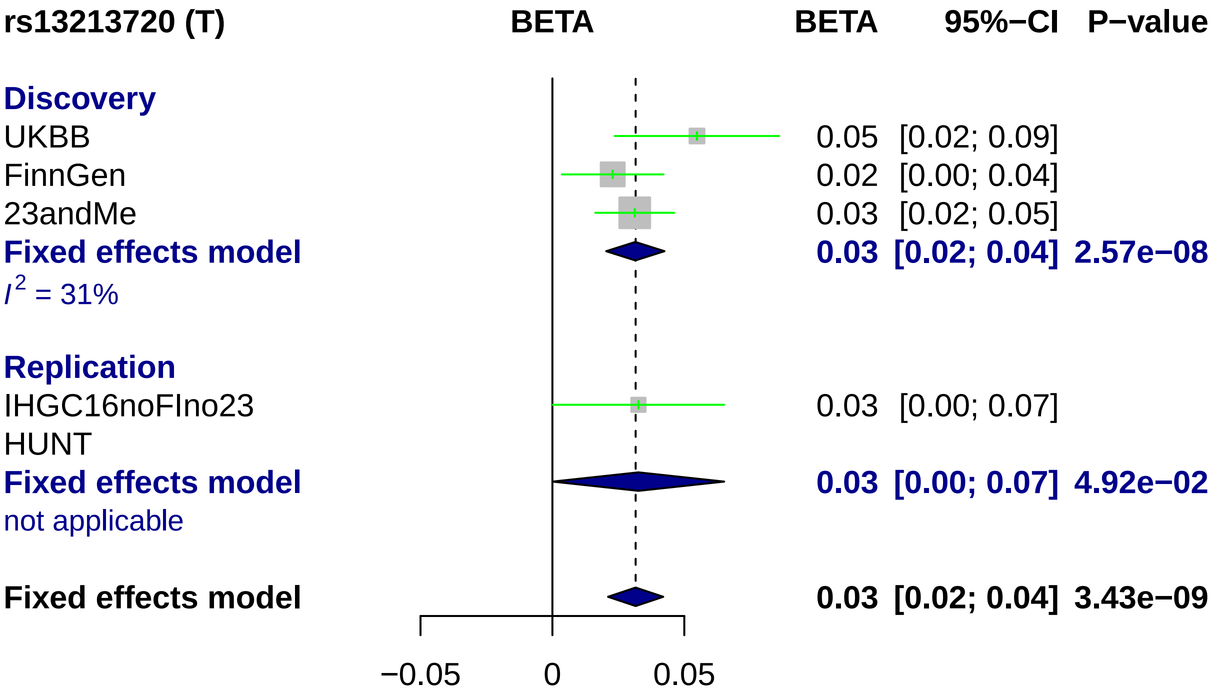
rs13213720 (T)
BETA
BETA
95%−CI
P−value
Discovery
UKBB
0.05
[0.02; 0.09]
FinnGen
0.02
[0.00; 0.04]
23andMe
0.03
[0.02; 0.05]
Fixed effects model
0.03
[0.02; 0.04]
2.57e−08
I^2 = 31%
Replication
IHGC16noFIno23
0.03
[0.00; 0.07]
HUNT
Fixed effects model
0.03
[0.00; 0.07]
4.92e−02
not applicable
Fixed effects model
0.03
[0.02; 0.04]
3.43e−09
−0.05
0
0.05

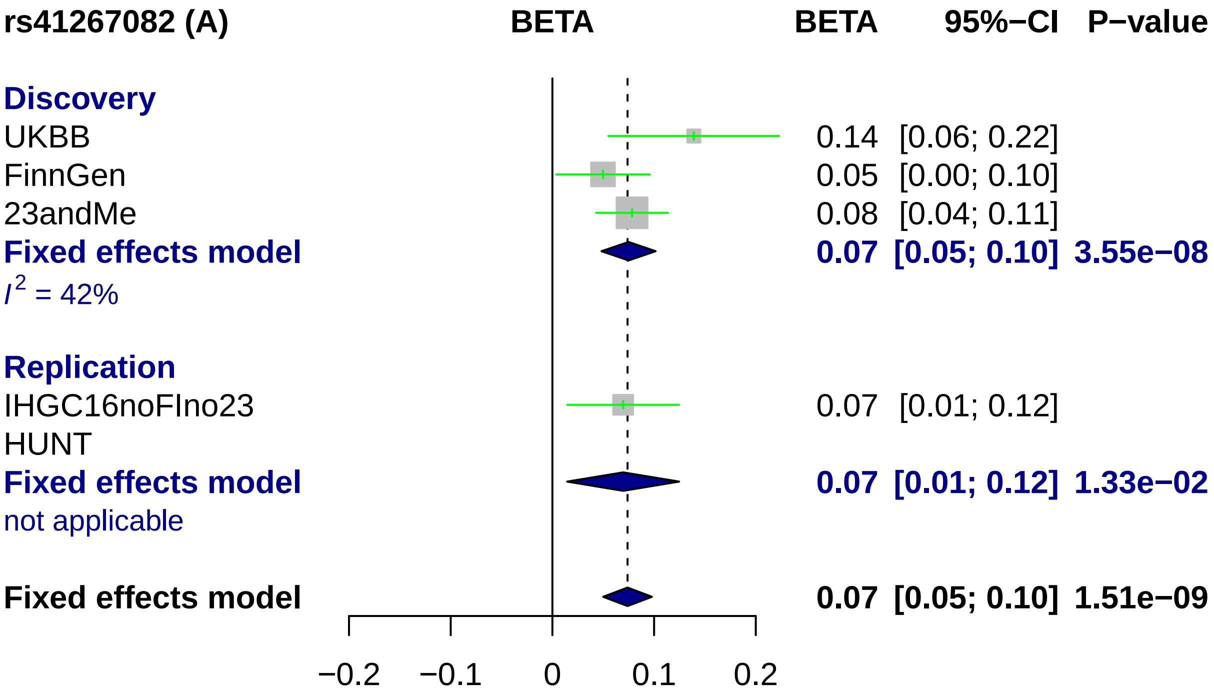
rs41267082 (A)
BETA
BETA
95%−CI
P−value
Discovery
UKBB 0.14 [0.06; 0.22]
FinnGen 0.05 [0.00; 0.10]
23andMe 0.08 [0.04; 0.11]
Fixed effects model 0.07 [0.05; 0.10] 3.55e−08
I^2 = 42%
Replication
IHGC16noFIno23 0.07 [0.01; 0.12]
HUNT
Fixed effects model 0.07 [0.01; 0.12] 1.33e−02
not applicable
Fixed effects model 0.07 [0.05; 0.10] 1.51e−09
−0.2
−0.1
0
0.1
0.2

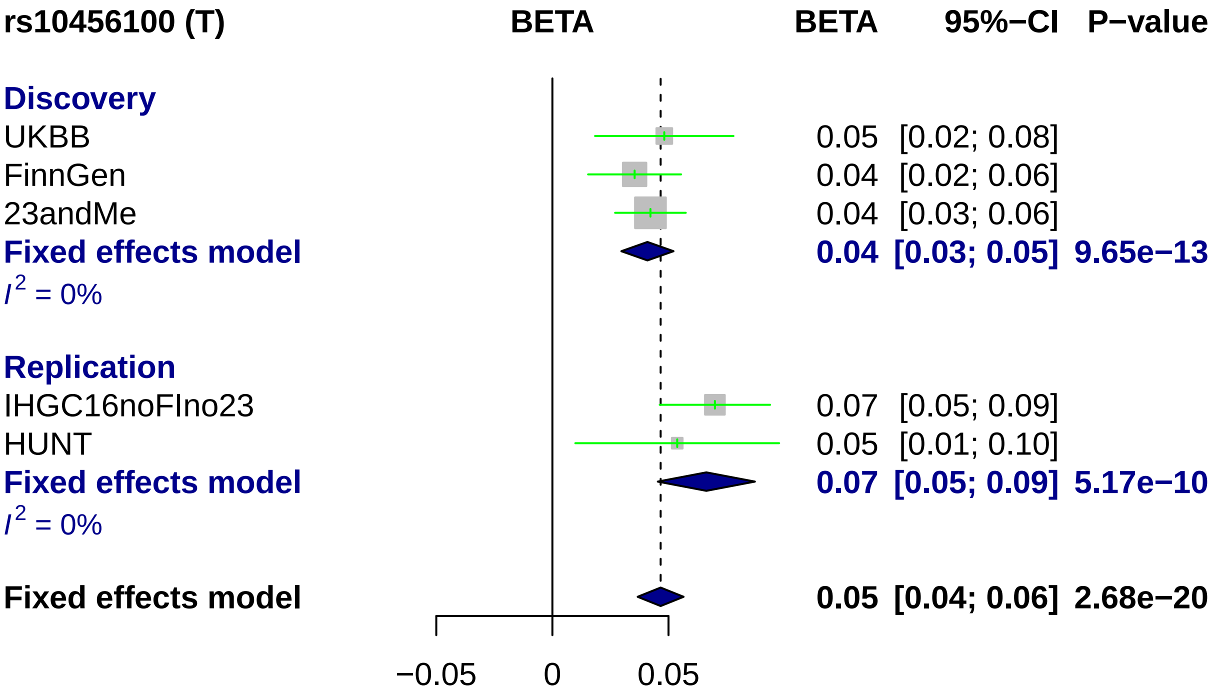
rs10456100 (T)
BETA
BETA
95%−CI
P−value
Discovery
UKBB
0.05 [0.02; 0.08]
FinnGen
0.04 [0.02; 0.06]
23andMe
0.04 [0.03; 0.06]
Fixed effects model
0.04 [0.03; 0.05] 9.65e−13
I2 = 0%
Replication
IHGC16noFIno23
0.07 [0.05; 0.09]
HUNT
0.05 [0.01; 0.10]
Fixed effects model
0.07 [0.05; 0.09] 5.17e−10
I2 = 0%
Fixed effects model
0.05 [0.04; 0.06] 2.68e−20
−0.05
0
0.05

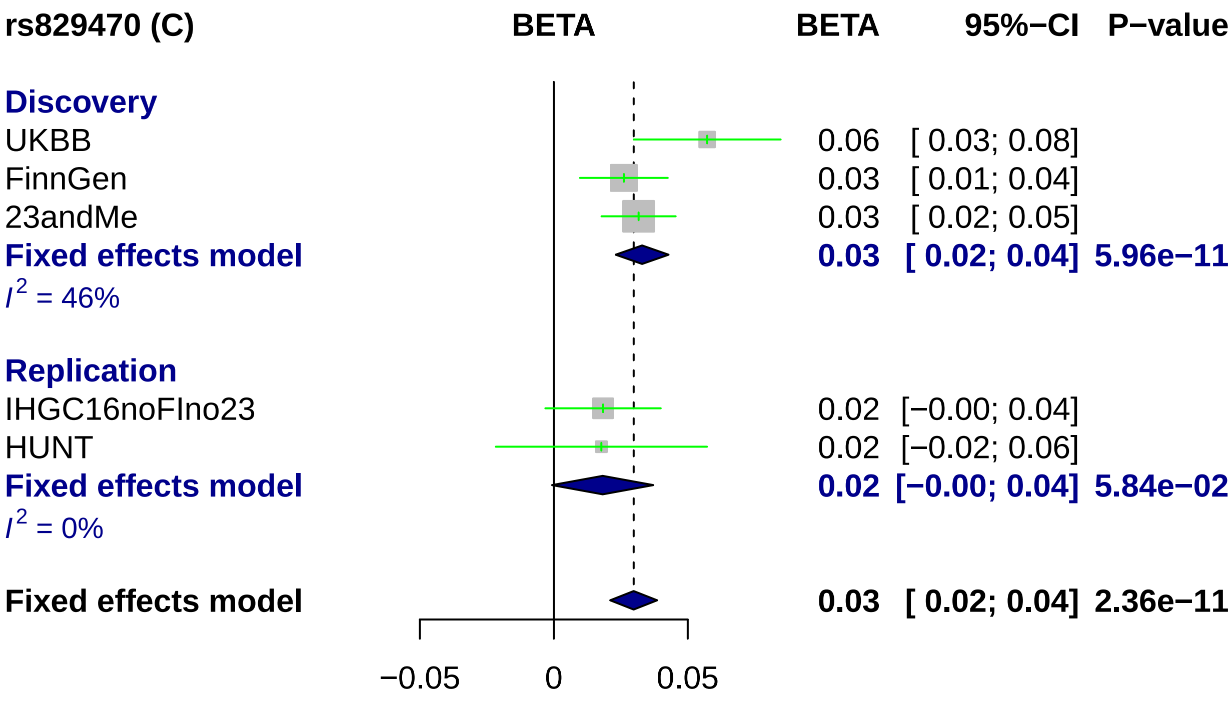
rs829470 (C)
BETA
BETA
95%−CI
P−value
Discovery
UKBB
0.06
[0.03; 0.08]
FinnGen
0.03
[0.01; 0.04]
23andMe
0.03
[0.02; 0.05]
Fixed effects model
0.03
[0.02; 0.04]
5.96e−11
I2 = 46%
Replication
IHGC16noFIno23
0.02
[−0.00; 0.04]
HUNT
0.02
[−0.02; 0.06]
Fixed effects model
0.02
[−0.00; 0.04]
5.84e−02
I2 = 0%
Fixed effects model
0.03
[0.02; 0.04]
2.36e−11
−0.05
0
0.05

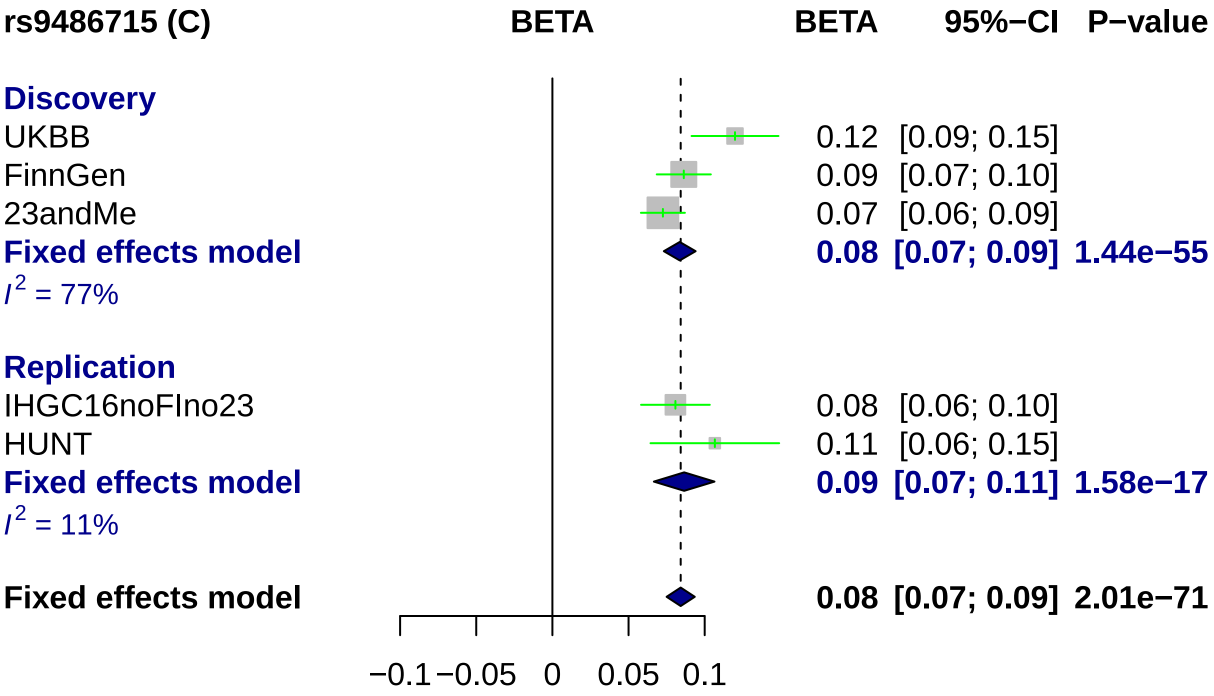
rs9486715 (C)
BETA
BETA
95%−CI
P−value
Discovery
UKBB
0.12 [0.09; 0.15]
FinnGen
0.09 [0.07; 0.10]
23andMe
0.07 [0.06; 0.09]
Fixed effects model
0.08 [0.07; 0.09] 1.44e−55
I2 = 77%
Replication
IHGC16noFIno23
0.08 [0.06; 0.10]
HUNT
0.11 [0.06; 0.15]
Fixed effects model
0.09 [0.07; 0.11] 1.58e−17
I2 = 11%
Fixed effects model
0.08 [0.07; 0.09] 2.01e−71
−0.1
−0.05
0
0.05
0.1

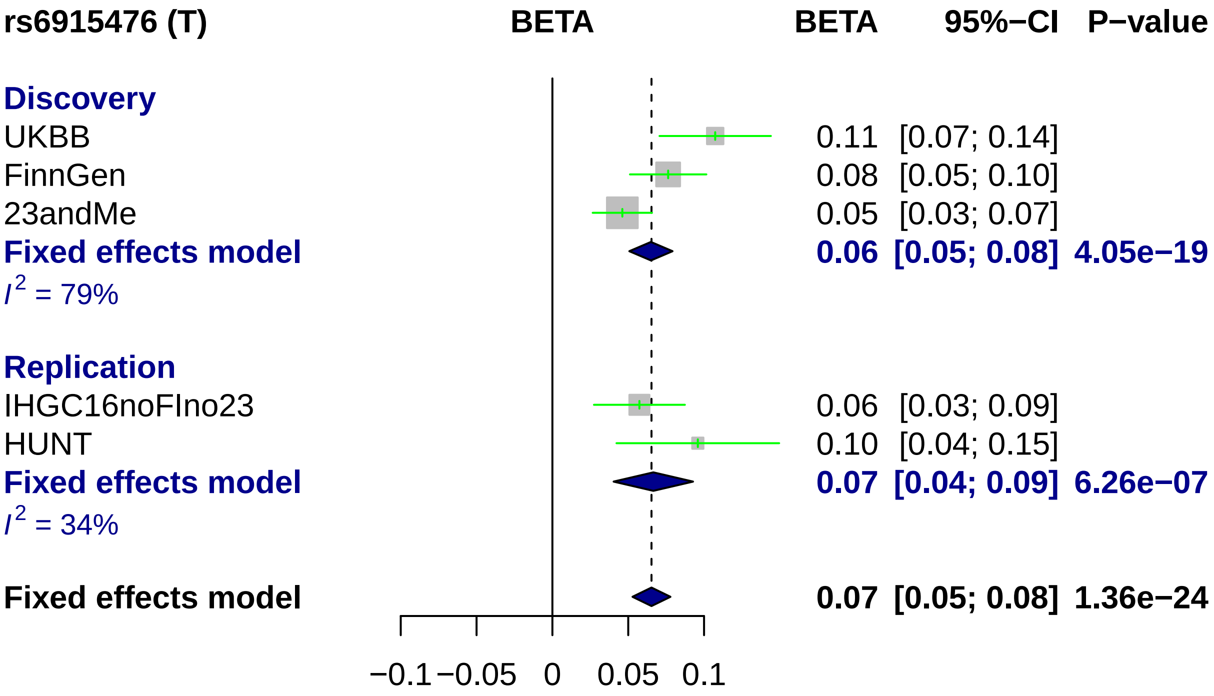
rs6915476 (T)
BETA
BETA
95%−CI
P−value
Discovery
UKBB
0.11 [0.07; 0.14]
FinnGen
0.08 [0.05; 0.10]
23andMe
0.05 [0.03; 0.07]
Fixed effects model
0.06 [0.05; 0.08] 4.05e−19
I2 = 79%
Replication
IHGC16noFIno23
0.06 [0.03; 0.09]
HUNT
0.10 [0.04; 0.15]
Fixed effects model
0.07 [0.04; 0.09] 6.26e−07
I2 = 34%
Fixed effects model
0.07 [0.05; 0.08] 1.36e−24
−0.1
−0.05
0
0.05
0.1

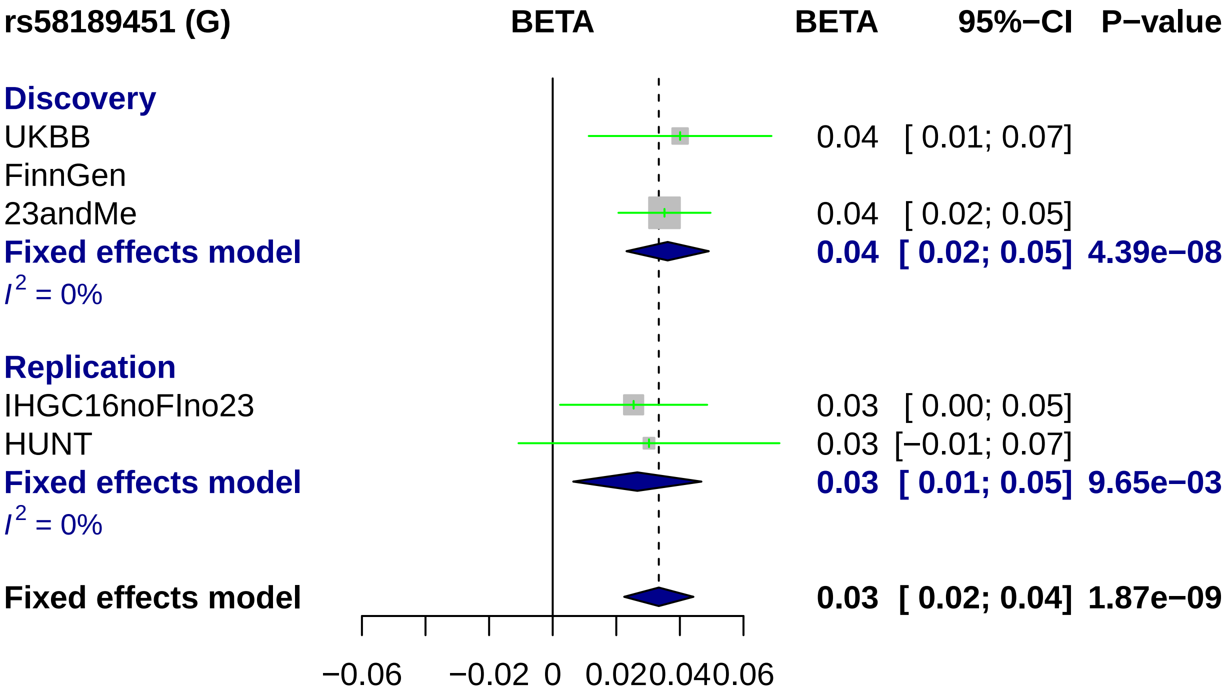

rs58189451 (G)
BETA
BETA
95%−CI
P−value
Discovery
UKBB
0.04
[0.01; 0.07]
FinnGen
23andMe
0.04
[0.02; 0.05]
Fixed effects model
0.04
[0.02; 0.05]
4.39e−08
I^2 = 0%
Replication
IHGC16noFIno23
0.03
[0.00; 0.05]
HUNT
0.03
[−0.01; 0.07]
Fixed effects model
0.03
[0.01; 0.05]
9.65e−03
I^2 = 0%
Fixed effects model
0.03
[0.02; 0.04]
1.87e−09
−0.06
−0.02
0
0.02
0.04
0.06

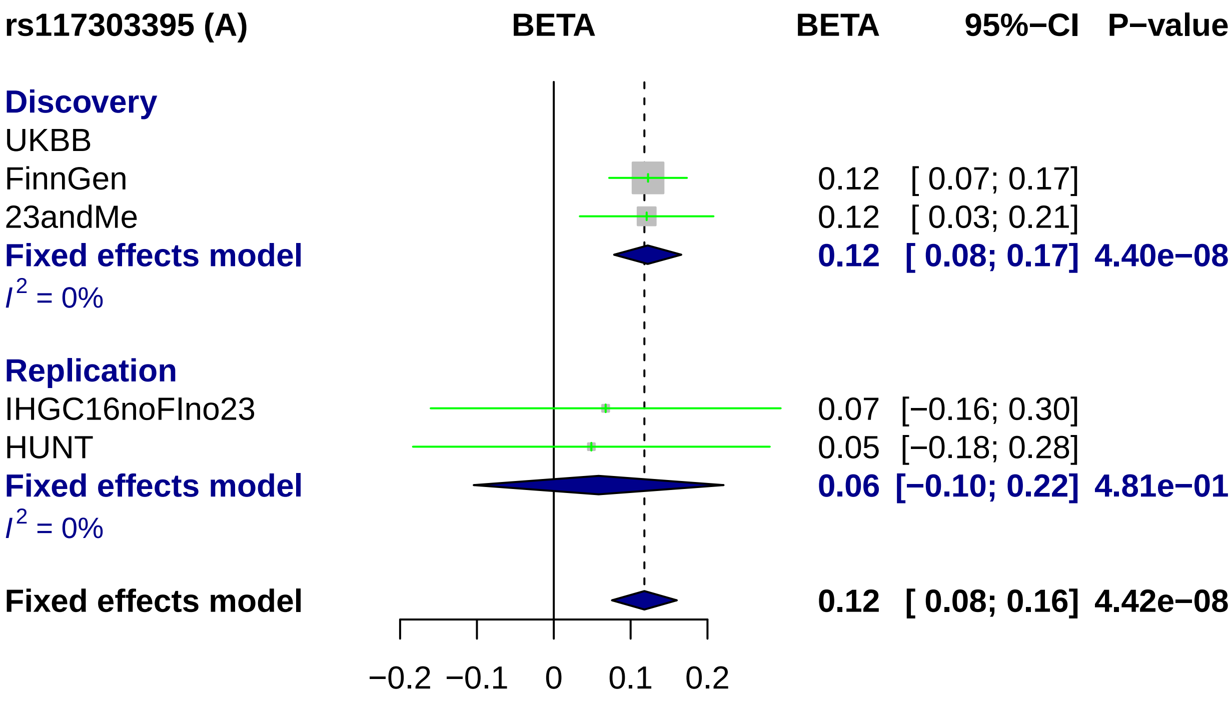
rs117303395 (A)
BETA
BETA
95%−CI
P−value
Discovery
UKBB
FinnGen 0.12 [0.07; 0.17]
23andMe 0.12 [0.03; 0.21]
Fixed effects model 0.12 [0.08; 0.17] 4.40e−08
I^2 = 0%
Replication
IHGC16noFIno23 0.07 [−0.16; 0.30]
HUNT 0.05 [−0.18; 0.28]
Fixed effects model 0.06 [−0.10; 0.22] 4.81e−01
I^2 = 0%
Fixed effects model 0.12 [0.08; 0.16] 4.42e−08
−0.2
−0.1
0
0.1
0.2

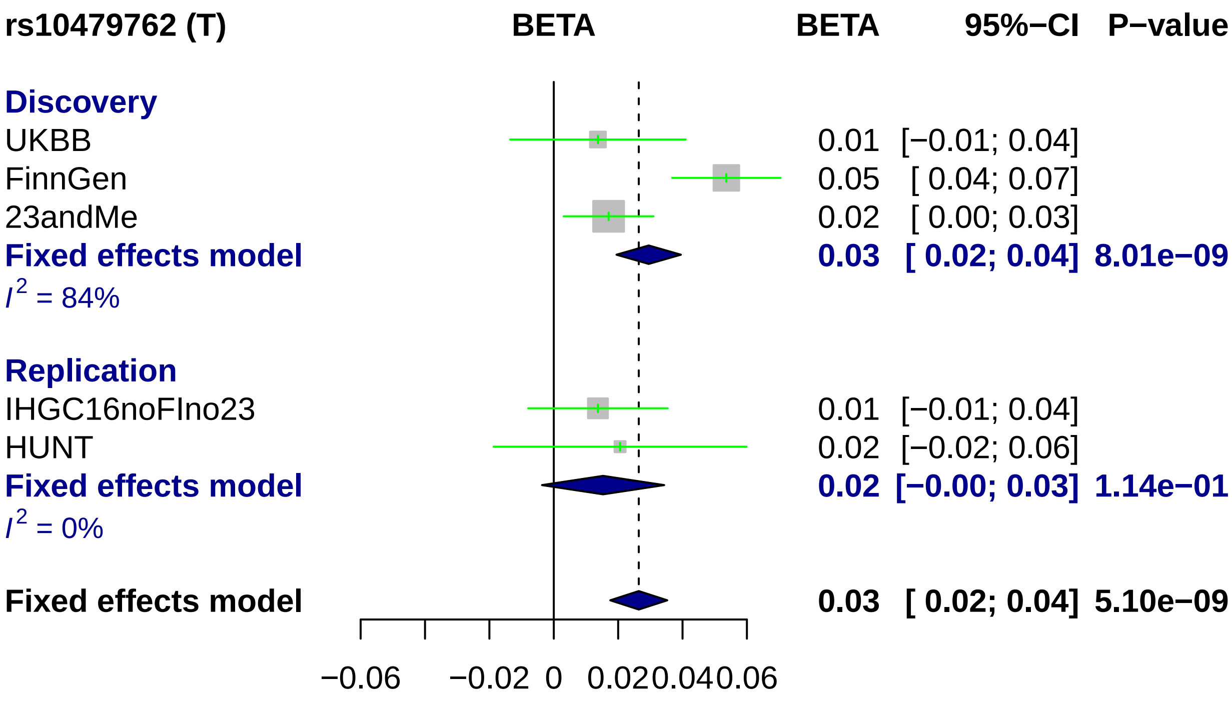
rs10479762 (T)
BETA
BETA
95%−CI
P−value
Discovery
UKBB
0.01
[−0.01; 0.04]
FinnGen
0.05
[0.04; 0.07]
23andMe
0.02
[0.00; 0.03]
Fixed effects model
0.03
[0.02; 0.04]
8.01e−09
I^2 = 84%
Replication
IHGC16noFIno23
0.01
[−0.01; 0.04]
HUNT
0.02
[−0.02; 0.06]
Fixed effects model
0.02
[−0.00; 0.03]
1.14e−01
I^2 = 0%
Fixed effects model
0.03
[0.02; 0.04]
5.10e−09
−0.06
−0.02
0
0.02
0.04
0.06

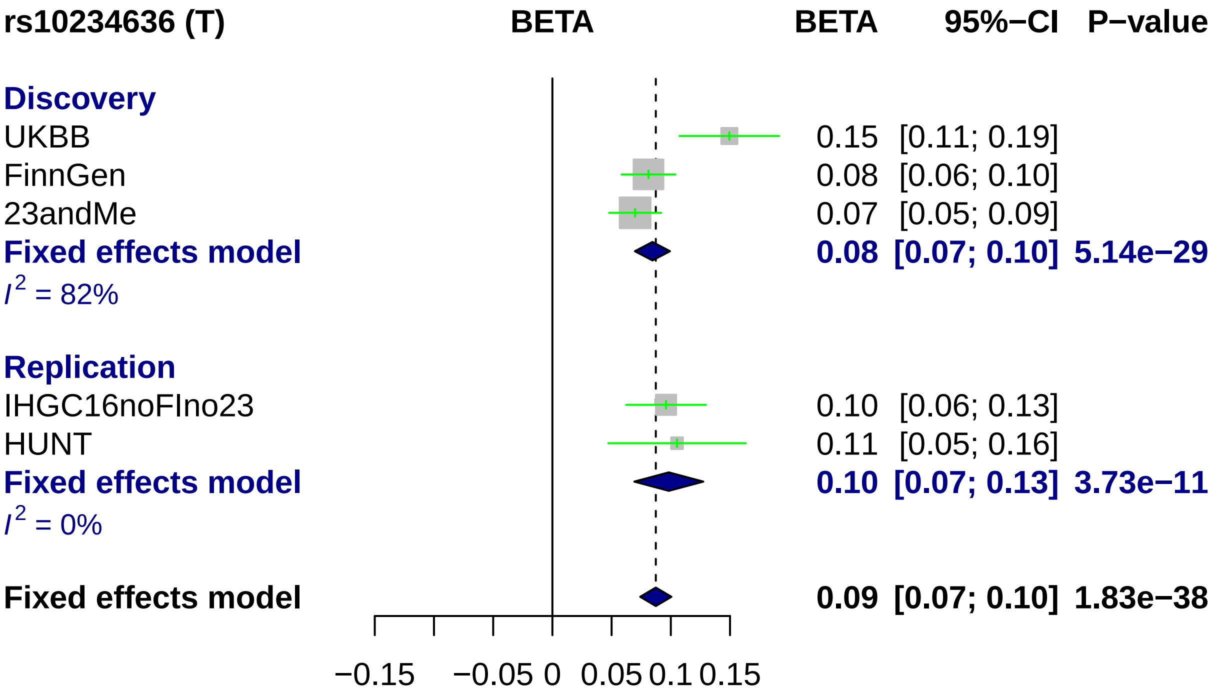
rs10234636 (T)
BETA
BETA
95%−CI
P−value
Discovery
UKBB
0.15 [0.11; 0.19]
FinnGen
0.08 [0.06; 0.10]
23andMe
0.07 [0.05; 0.09]
Fixed effects model
0.08 [0.07; 0.10] 5.14e−29
I^2 = 82%
Replication
IHGC16noFIno23
0.10 [0.06; 0.13]
HUNT
0.11 [0.05; 0.16]
Fixed effects model
0.10 [0.07; 0.13] 3.73e−11
I^2 = 0%
Fixed effects model
0.09 [0.07; 0.10] 1.83e−38
−0.15
−0.05
0
0.05
0.1
0.15

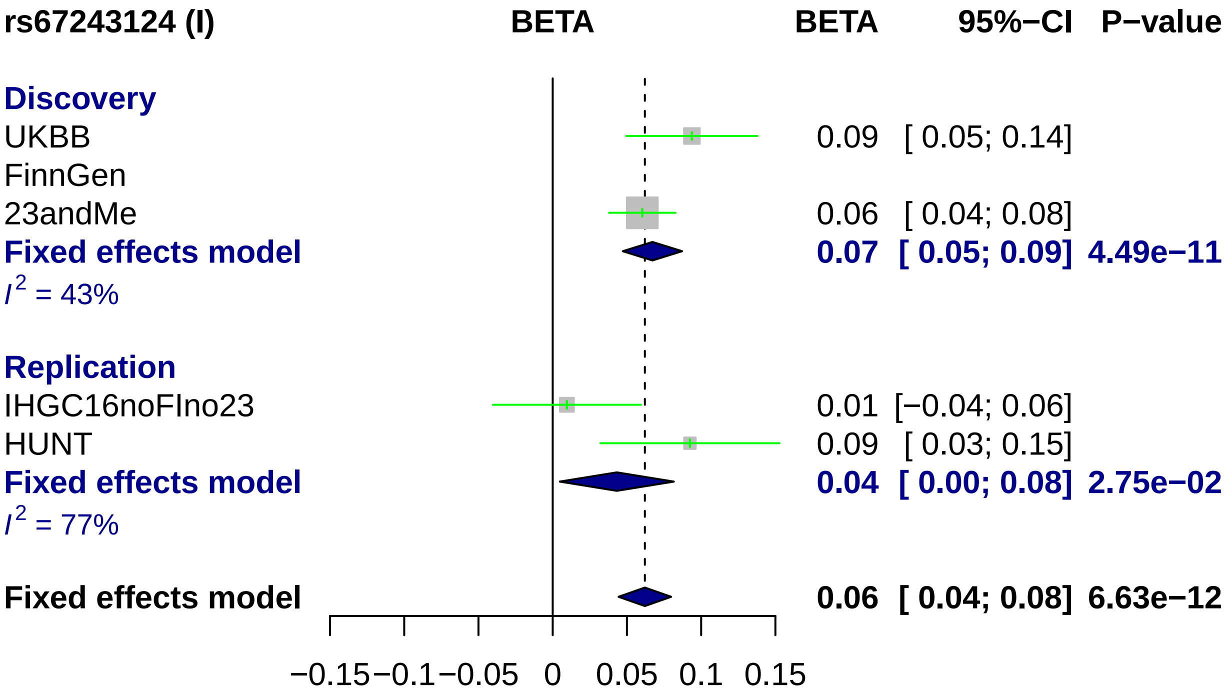
rs67243124 (I)
BETA
BETA
95%−CI
P−value
Discovery
UKBB
0.09 [0.05; 0.14]
FinnGen
23andMe
0.06 [0.04; 0.08]
Fixed effects model
0.07 [0.05; 0.09] 4.49e−11
I^2 = 43%
Replication
IHGC16noFIno23
0.01 [−0.04; 0.06]
HUNT
0.09 [0.03; 0.15]
Fixed effects model
0.04 [0.00; 0.08] 2.75e−02
I^2 = 77%
Fixed effects model
0.06 [0.04; 0.08] 6.63e−12
−0.15
−0.1
−0.05
0
0.05
0.1
0.15

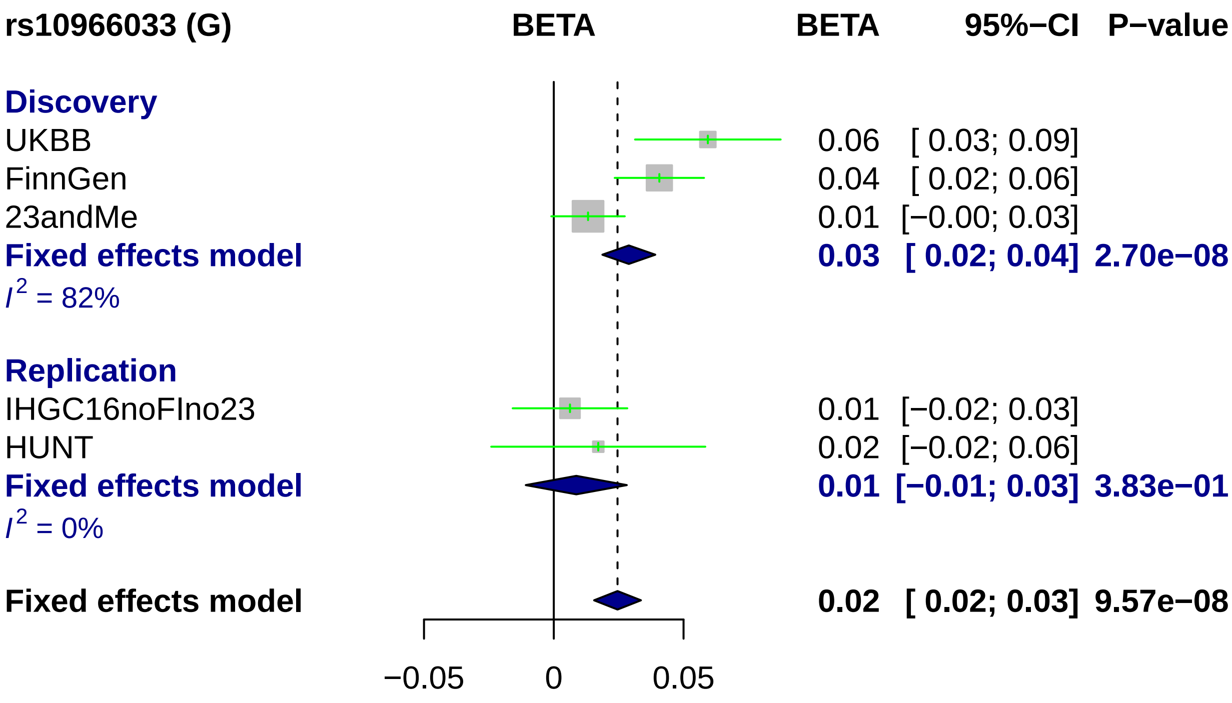

rs10966033 (G)
BETA
BETA
95%−CI
P−value
Discovery
UKBB
0.06
[0.03; 0.09]
FinnGen
0.04
[0.02; 0.06]
23andMe
0.01
[−0.00; 0.03]
Fixed effects model
0.03
[0.02; 0.04]
2.70e−08
I2 = 82%
Replication
IHGC16noFIno23
0.01
[−0.02; 0.03]
HUNT
0.02
[−0.02; 0.06]
Fixed effects model
0.01
[−0.01; 0.03]
3.83e−01
I2 = 0%
Fixed effects model
0.02
[0.02; 0.03]
9.57e−08
−0.05
0
0.05

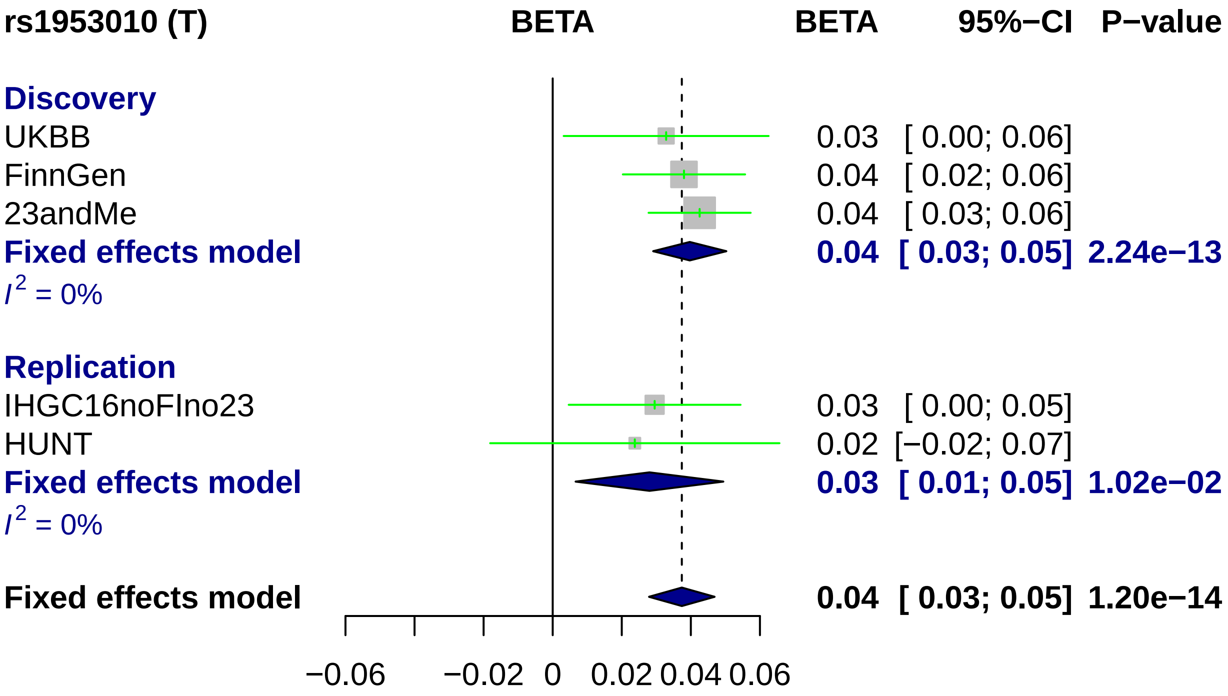
rs1953010 (T)
BETA
BETA
95%−CI
P−value
Discovery
UKBB
0.03 [0.00; 0.06]
FinnGen
0.04 [0.02; 0.06]
23andMe
0.04 [0.03; 0.06]
Fixed effects model
0.04 [0.03; 0.05] 2.24e−13
I2 = 0%
Replication
IHGC16noFIno23
0.03 [0.00; 0.05]
HUNT
0.02 [−0.02; 0.07]
Fixed effects model
0.03 [0.01; 0.05] 1.02e−02
I2 = 0%
Fixed effects model
0.04 [0.03; 0.05] 1.20e−14
−0.06
−0.02
0
0.02
0.04
0.06

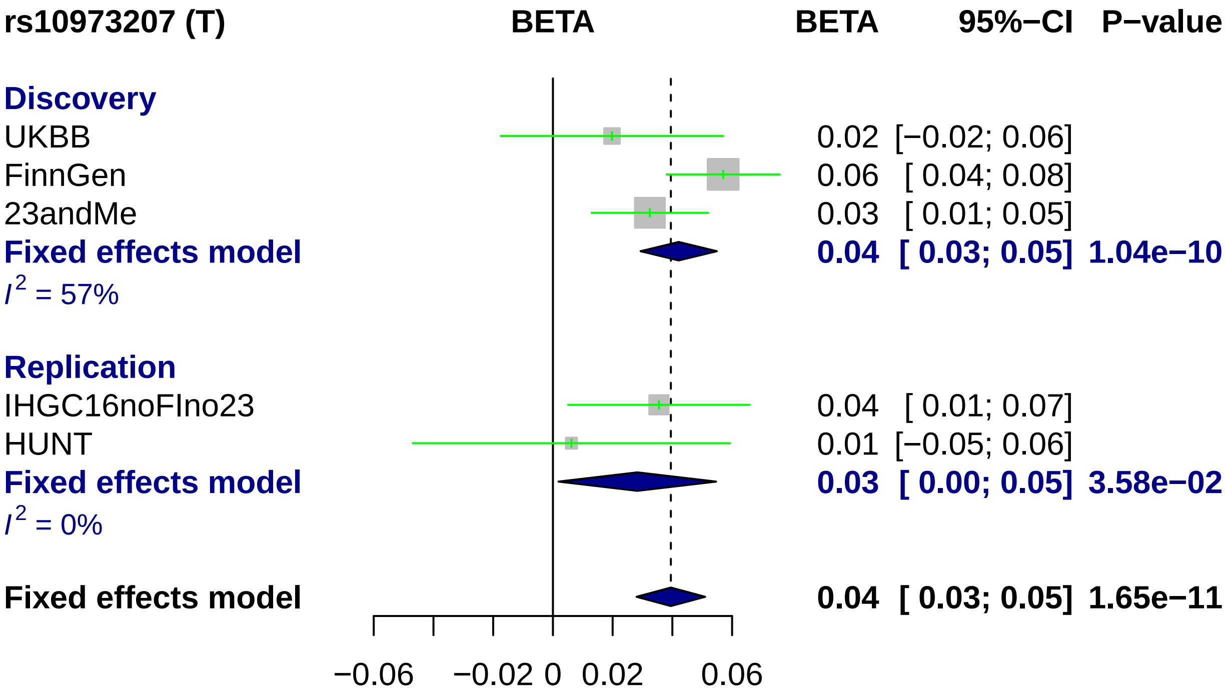
rs10973207 (T)
BETA
BETA
95%−CI
P−value
Discovery
UKBB
0.02 [−0.02; 0.06]
FinnGen
0.06 [0.04; 0.08]
23andMe
0.03 [0.01; 0.05]
Fixed effects model
0.04 [0.03; 0.05] 1.04e−10
I2 = 57%
Replication
IHGC16noFIno23
0.04 [0.01; 0.07]
HUNT
0.01 [−0.05; 0.06]
Fixed effects model
0.03 [0.00; 0.05] 3.58e−02
I2 = 0%
Fixed effects model
0.04 [0.03; 0.05] 1.65e−11
−0.06
−0.02
0
0.02
0.06

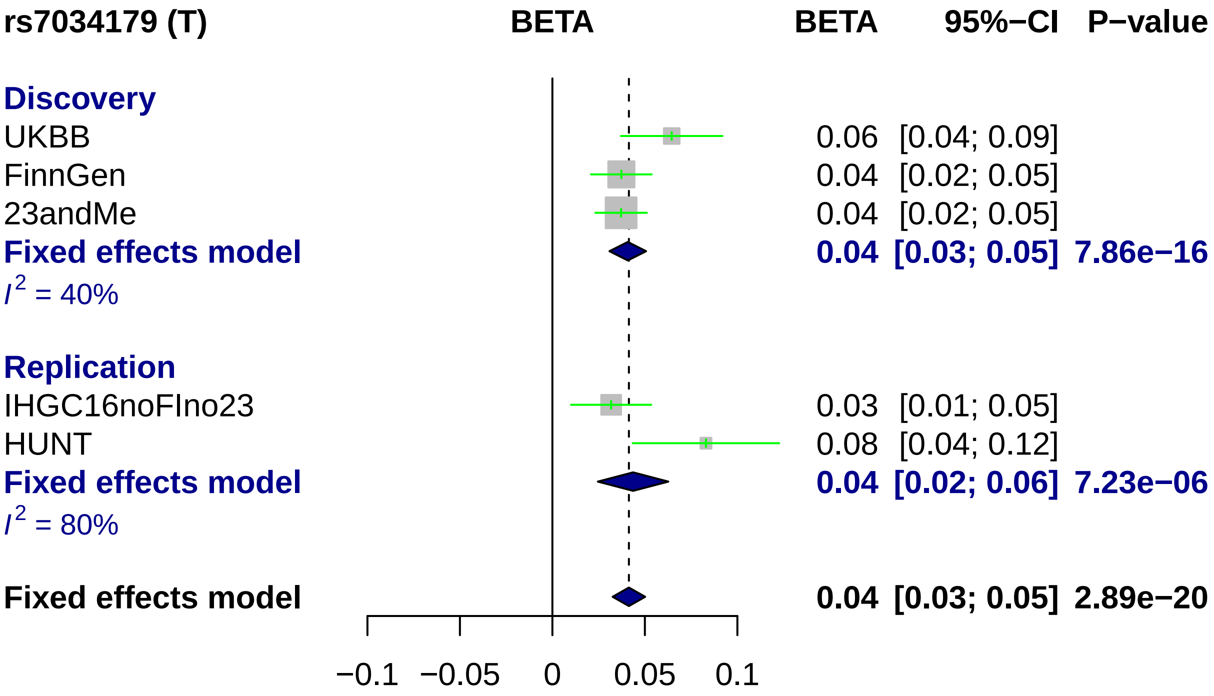
rs7034179 (T)
BETA
BETA 95%−CI P−value
Discovery
UKBB 0.06 [0.04; 0.09]
FinnGen 0.04 [0.02; 0.05]
23andMe 0.04 [0.02; 0.05]
Fixed effects model 0.04 [0.03; 0.05] 7.86e−16
I^2 = 40%
Replication
IHGC16noFIno23 0.03 [0.01; 0.05]
HUNT 0.08 [0.04; 0.12]
Fixed effects model 0.04 [0.02; 0.06] 7.23e−06
I^2 = 80%
Fixed effects model 0.04 [0.03; 0.05] 2.89e−20
−0.1
−0.05
0
0.05
0.1

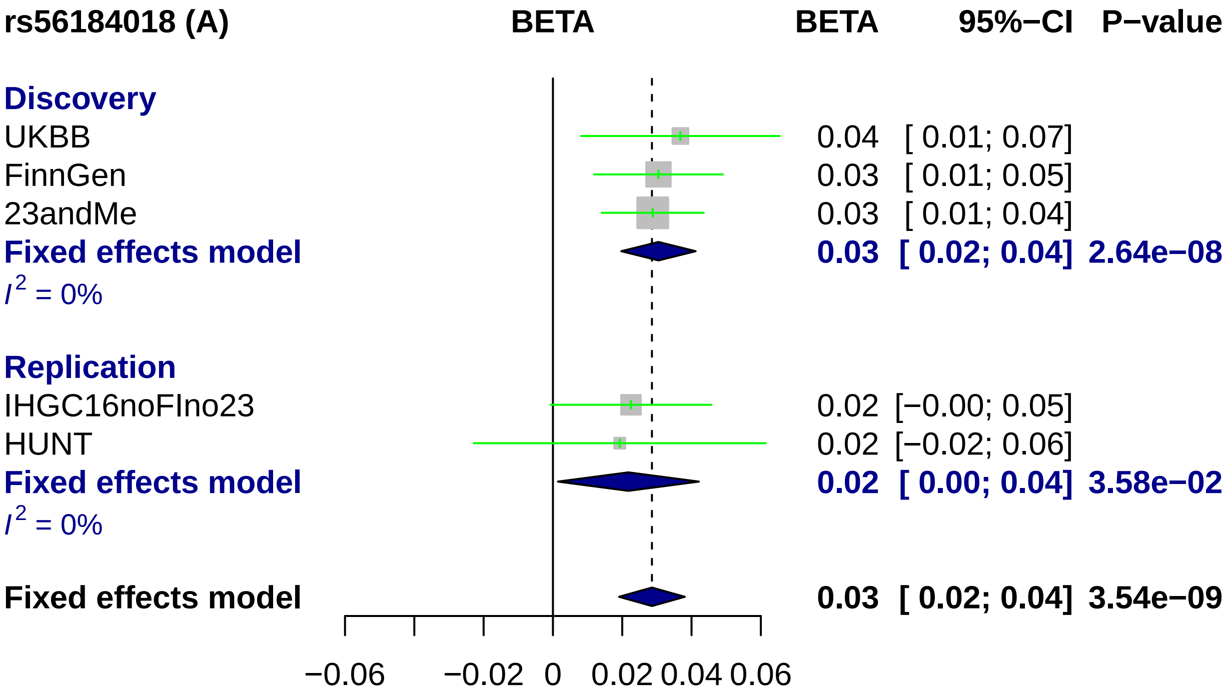
rs56184018 (A)
BETA
BETA
95%−CI
P−value
Discovery
UKBB 0.04 [0.01; 0.07]
FinnGen 0.03 [0.01; 0.05]
23andMe 0.03 [0.01; 0.04]
Fixed effects model 0.03 [0.02; 0.04] 2.64e−08
I^2 = 0%
Replication
IHGC16noFIno23 0.02 [−0.00; 0.05]
HUNT 0.02 [−0.02; 0.06]
Fixed effects model 0.02 [0.00; 0.04] 3.58e−02
I^2 = 0%
Fixed effects model 0.03 [0.02; 0.04] 3.54e−09
−0.06
−0.02
0
0.02
0.04
0.06

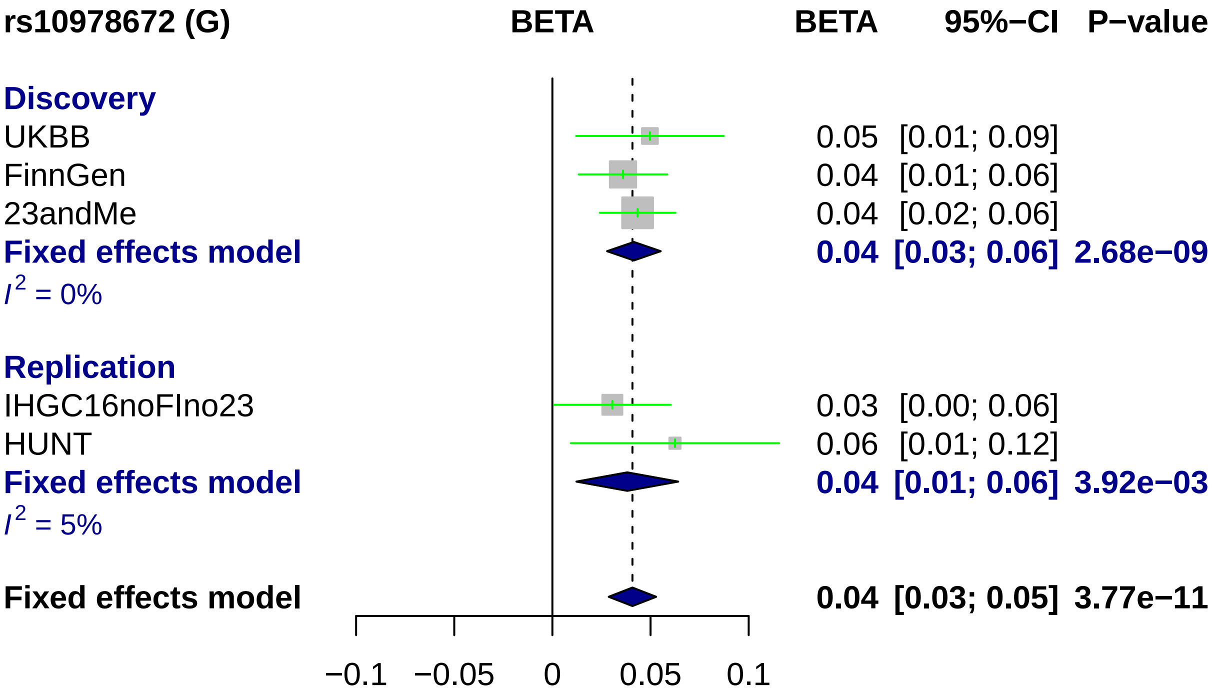
rs10978672 (G)
BETA
BETA
95%−CI
P−value
Discovery
UKBB
0.05 [0.01; 0.09]
FinnGen
0.04 [0.01; 0.06]
23andMe
0.04 [0.02; 0.06]
Fixed effects model
0.04 [0.03; 0.06] 2.68e−09
I2 = 0%
Replication
IHGC16noFIno23
0.03 [0.00; 0.06]
HUNT
0.06 [0.01; 0.12]
Fixed effects model
0.04 [0.01; 0.06] 3.92e−03
I2 = 5%
Fixed effects model
0.04 [0.03; 0.05] 3.77e−11
−0.1
−0.05
0
0.05
0.1

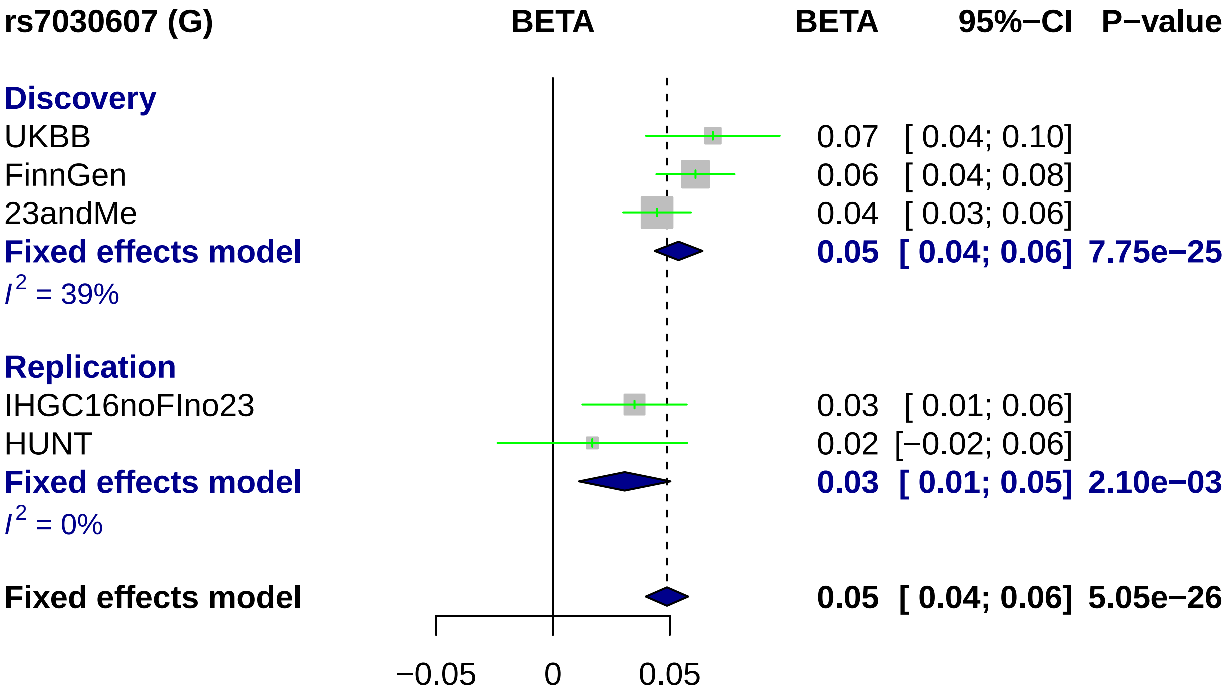
rs7030607 (G)
BETA
BETA
95%−CI
P−value
Discovery
UKBB
0.07 [0.04; 0.10]
FinnGen
0.06 [0.04; 0.08]
23andMe
0.04 [0.03; 0.06]
Fixed effects model
0.05 [0.04; 0.06] 7.75e−25
I2 = 39%
Replication
IHGC16noFIno23
0.03 [0.01; 0.06]
HUNT
0.02 [−0.02; 0.06]
Fixed effects model
0.03 [0.01; 0.05] 2.10e−03
I2 = 0%
Fixed effects model
0.05 [0.04; 0.06] 5.05e−26
−0.05
0
0.05

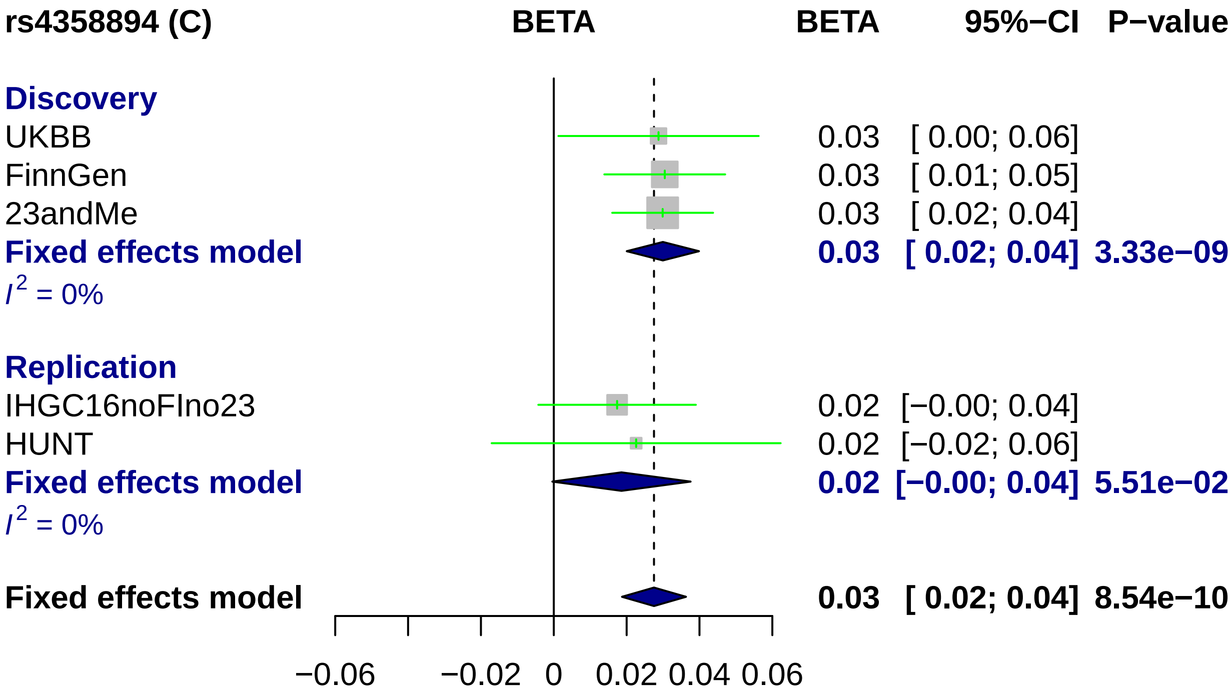
rs4358894 (C)
BETA
BETA
95%−CI
P−value
Discovery
UKBB
0.03
[0.00; 0.06]
FinnGen
0.03
[0.01; 0.05]
23andMe
0.03
[0.02; 0.04]
Fixed effects model
0.03
[0.02; 0.04]
3.33e−09
I^2 = 0%
Replication
IHGC16noFIno23
0.02
[−0.00; 0.04]
HUNT
0.02
[−0.02; 0.06]
Fixed effects model
0.02
[−0.00; 0.04]
5.51e−02
I^2 = 0%
Fixed effects model
0.03
[0.02; 0.04]
8.54e−10
−0.06
−0.02
0
0.02
0.04
0.06

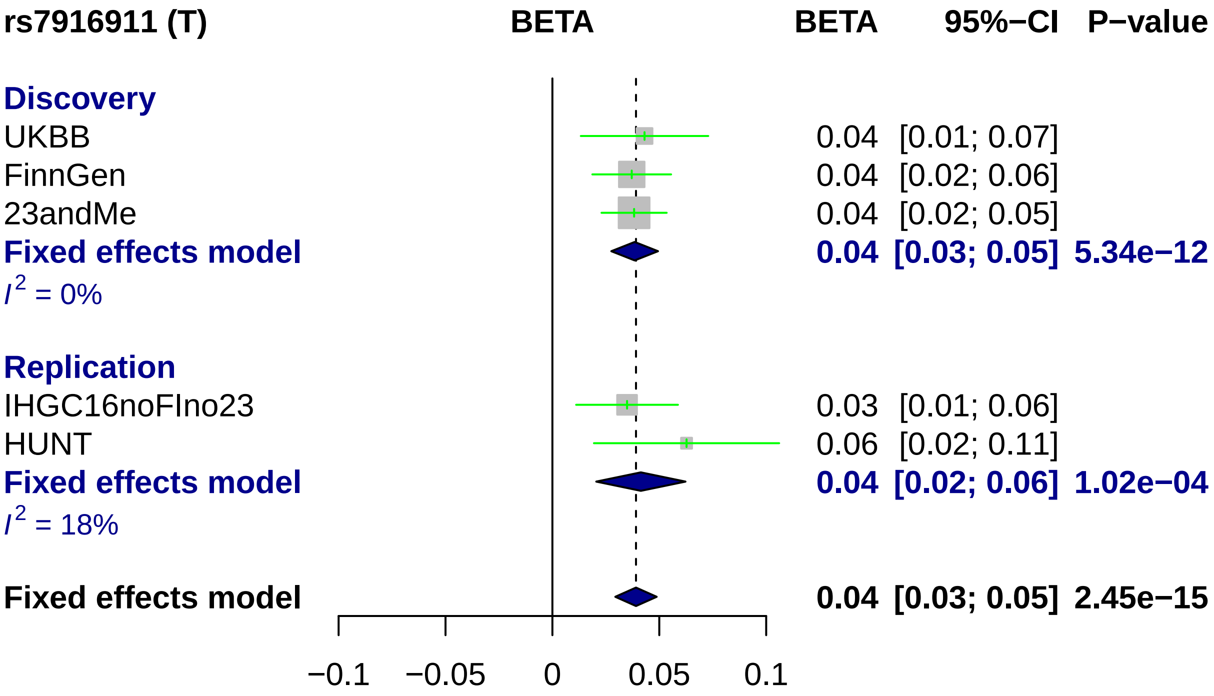

rs7916911 (T)
BETA
BETA
95%−CI
P−value
Discovery
UKBB
0.04 [0.01; 0.07]
FinnGen
0.04 [0.02; 0.06]
23andMe
0.04 [0.02; 0.05]
Fixed effects model
0.04 [0.03; 0.05] 5.34e−12
I2 = 0%
Replication
IHGC16noFIno23
0.03 [0.01; 0.06]
HUNT
0.06 [0.02; 0.11]
Fixed effects model
0.04 [0.02; 0.06] 1.02e−04
I2 = 18%
Fixed effects model
0.04 [0.03; 0.05] 2.45e−15
−0.1
−0.05
0
0.05
0.1

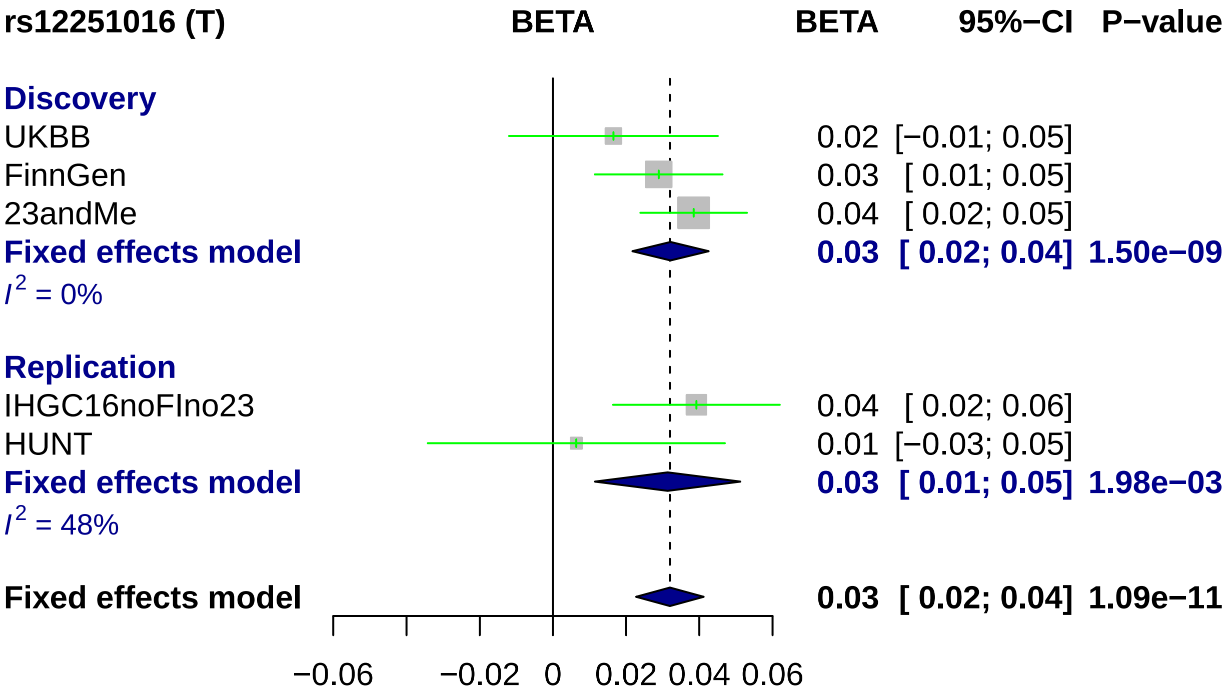
rs12251016 (T)
BETA
BETA
95%−CI
P−value
Discovery
UKBB
0.02 [−0.01; 0.05]
FinnGen
0.03 [0.01; 0.05]
23andMe
0.04 [0.02; 0.05]
Fixed effects model
0.03 [0.02; 0.04] 1.50e−09
I^2 = 0%
Replication
IHGC16noFIno23
0.04 [0.02; 0.06]
HUNT
0.01 [−0.03; 0.05]
Fixed effects model
0.03 [0.01; 0.05] 1.98e−03
I^2 = 48%
Fixed effects model
0.03 [0.02; 0.04] 1.09e−11
−0.06
−0.02
0
0.02
0.04
0.06

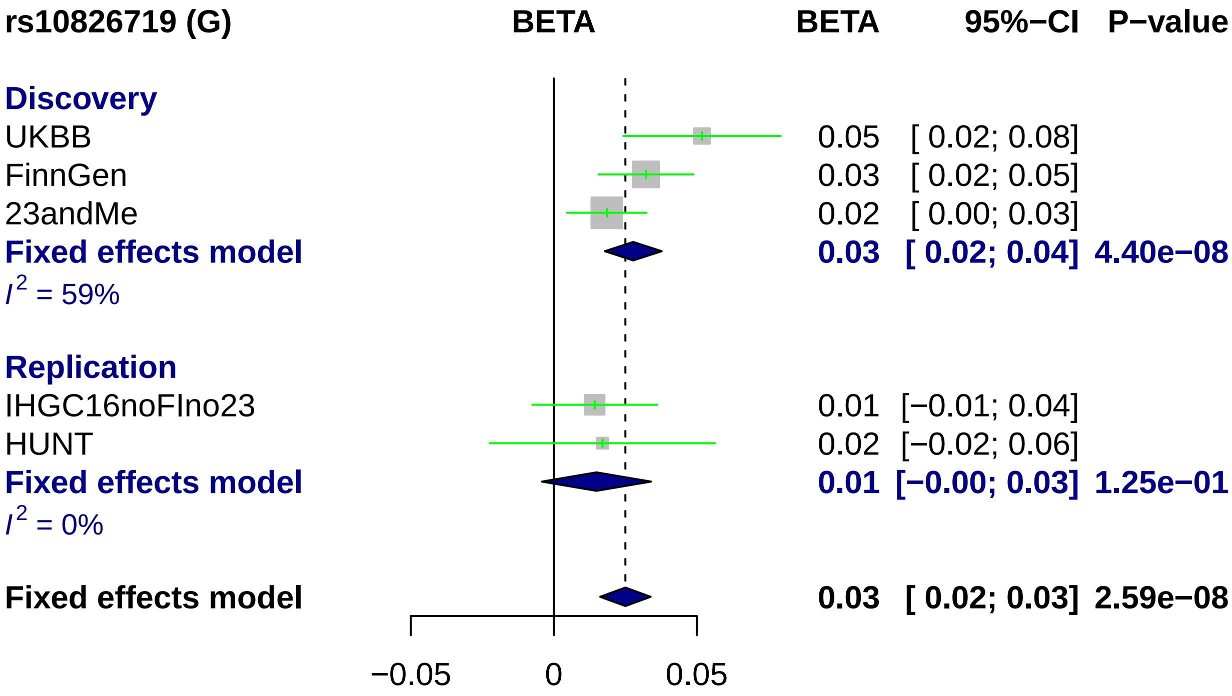
rs10826719 (G)
BETA
BETA
95%−CI
P−value
Discovery
UKBB
0.05 [0.02; 0.08]
FinnGen
0.03 [0.02; 0.05]
23andMe
0.02 [0.00; 0.03]
Fixed effects model
0.03 [0.02; 0.04] 4.40e−08
I2 = 59%
Replication
IHGC16noFIno23
0.01 [−0.01; 0.04]
HUNT
0.02 [−0.02; 0.06]
Fixed effects model
0.01 [−0.00; 0.03] 1.25e−01
I2 = 0%
Fixed effects model
0.03 [0.02; 0.03] 2.59e−08
−0.05
0
0.05

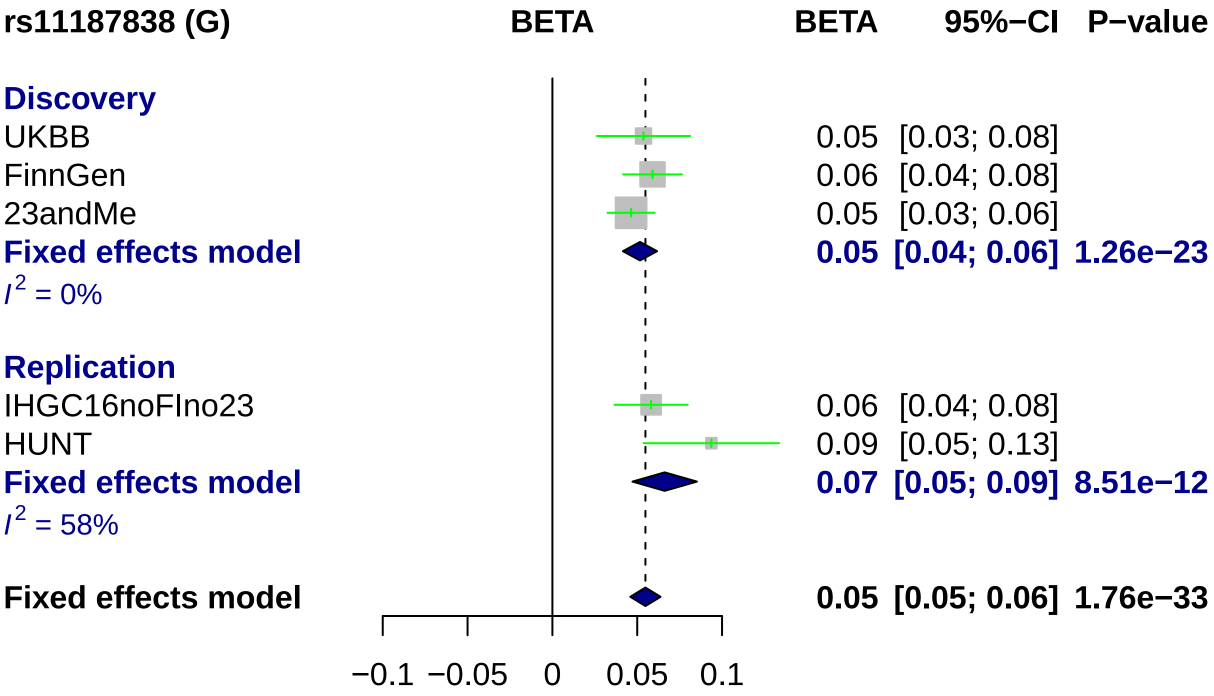

rs11187838 (G)
BETA
BETA
95%−CI
P−value
Discovery
UKBB
0.05 [0.03; 0.08]
FinnGen
0.06 [0.04; 0.08]
23andMe
0.05 [0.03; 0.06]
Fixed effects model
0.05 [0.04; 0.06] 1.26e−23
I2 = 0%
Replication
IHGC16noFIno23
0.06 [0.04; 0.08]
HUNT
0.09 [0.05; 0.13]
Fixed effects model
0.07 [0.05; 0.09] 8.51e−12
I2 = 58%
Fixed effects model
0.05 [0.05; 0.06] 1.76e−33
−0.1
−0.05
0
0.05
0.1

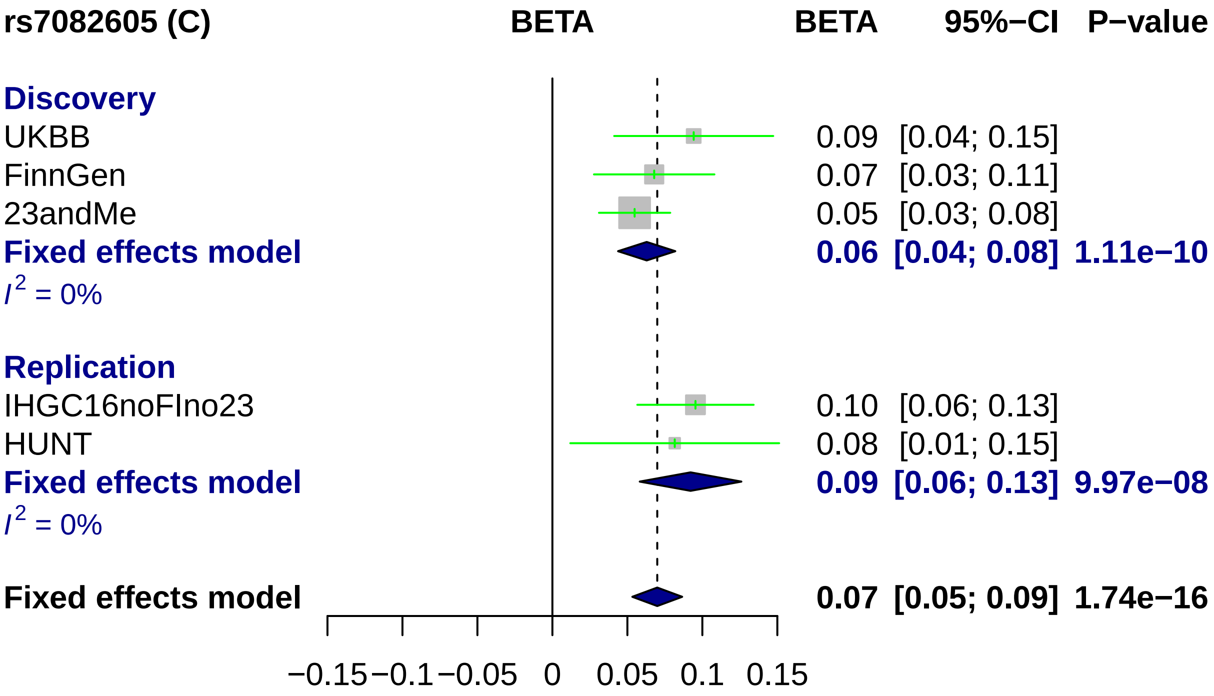
rs7082605 (C)
BETA
BETA
95%−CI
P−value
Discovery
UKBB
0.09 [0.04; 0.15]
FinnGen
0.07 [0.03; 0.11]
23andMe
0.05 [0.03; 0.08]
Fixed effects model
0.06 [0.04; 0.08] 1.11e−10
I2 = 0%
Replication
IHGC16noFIno23
0.10 [0.06; 0.13]
HUNT
0.08 [0.01; 0.15]
Fixed effects model
0.09 [0.06; 0.13] 9.97e−08
I2 = 0%
Fixed effects model
0.07 [0.05; 0.09] 1.74e−16
−0.15
−0.1
−0.05
0
0.05
0.1
0.15

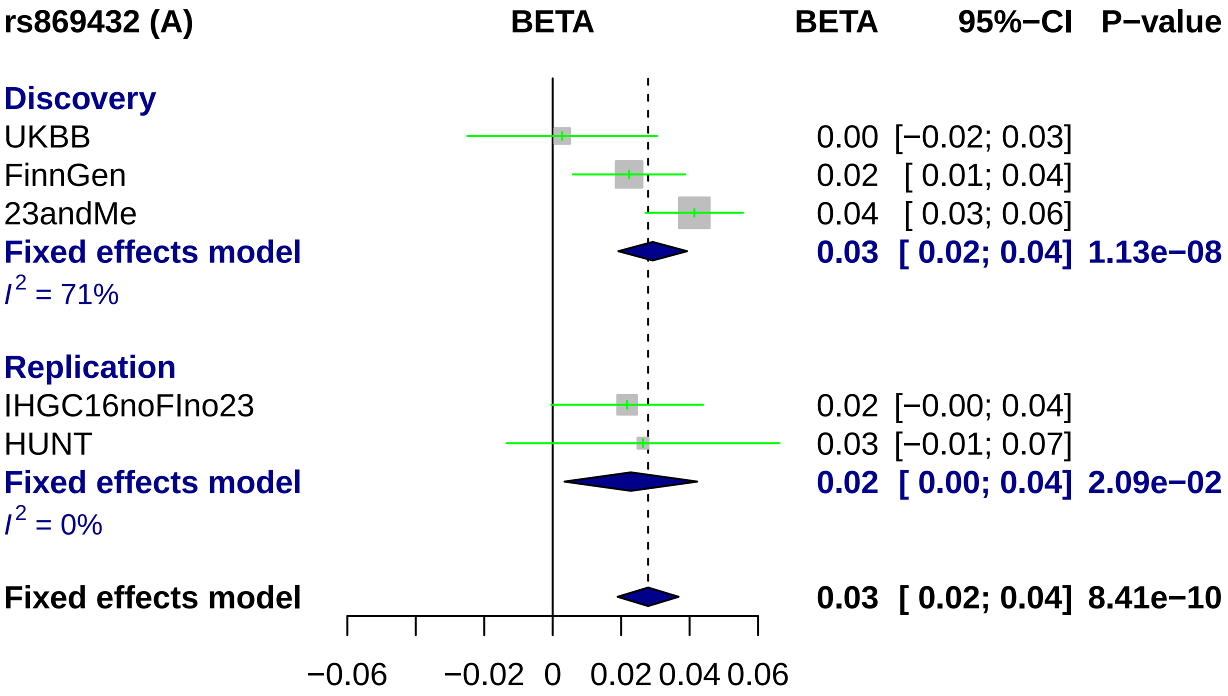

rs869432 (A)
BETA
BETA
95%−CI
P−value
Discovery
UKBB
0.00 [−0.02; 0.03]
FinnGen
0.02 [0.01; 0.04]
23andMe
0.04 [0.03; 0.06]
Fixed effects model
0.03 [0.02; 0.04] 1.13e−08
I^2 = 71%
Replication
IHGC16noFIno23
0.02 [−0.00; 0.04]
HUNT
0.03 [−0.01; 0.07]
Fixed effects model
0.02 [0.00; 0.04] 2.09e−02
I^2 = 0%
Fixed effects model
0.03 [0.02; 0.04] 8.41e−10
−0.06
−0.02
0
0.02
0.04
0.06

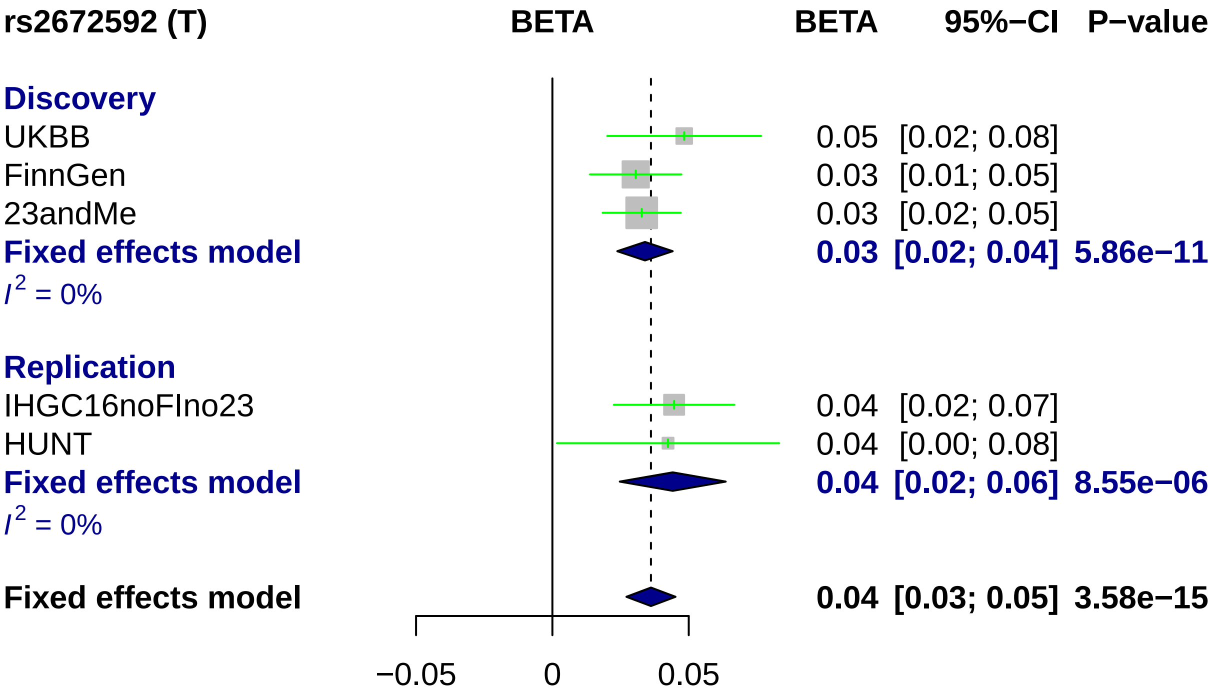
rs2672592 (T)
BETA
BETA
95%−CI
P−value
Discovery
UKBB
0.05 [0.02; 0.08]
FinnGen
0.03 [0.01; 0.05]
23andMe
0.03 [0.02; 0.05]
Fixed effects model
0.03 [0.02; 0.04] 5.86e−11
I2 = 0%
Replication
IHGC16noFIno23
0.04 [0.02; 0.07]
HUNT
0.04 [0.00; 0.08]
Fixed effects model
0.04 [0.02; 0.06] 8.55e−06
I2 = 0%
Fixed effects model
0.04 [0.03; 0.05] 3.58e−15
−0.05
0
0.05

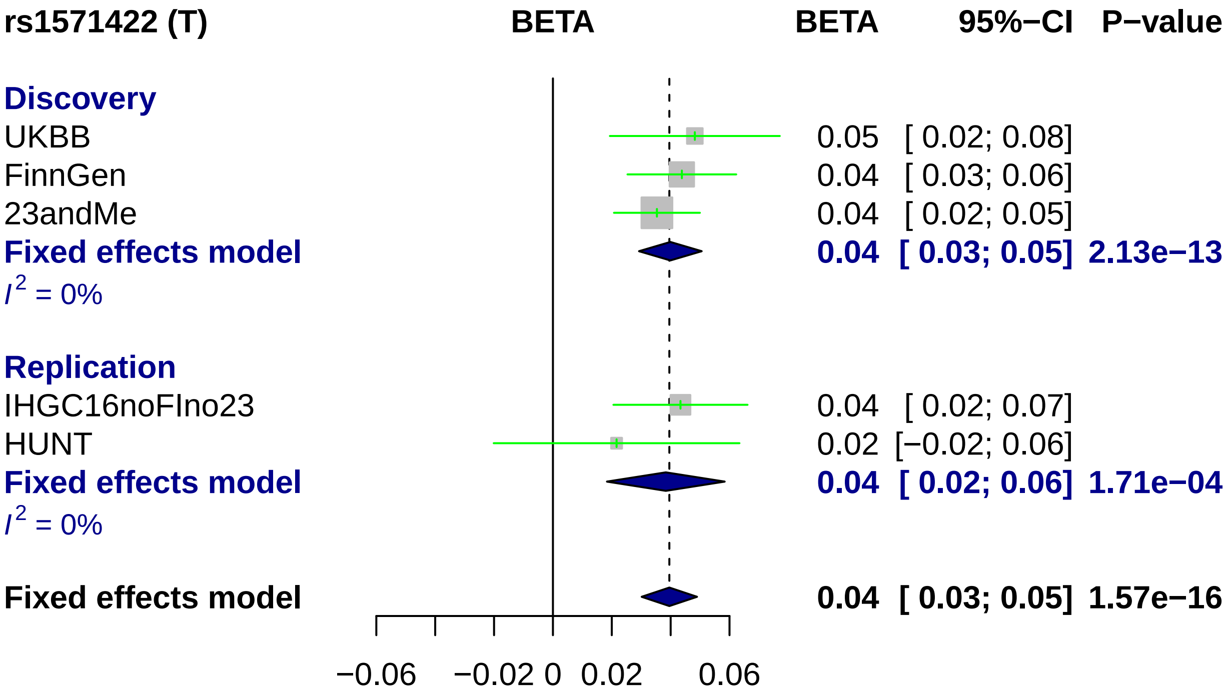
rs1571422 (T)
BETA
BETA
95%−CI
P−value
Discovery
UKBB
0.05
[0.02; 0.08]
FinnGen
0.04
[0.03; 0.06]
23andMe
0.04
[0.02; 0.05]
Fixed effects model
0.04
[0.03; 0.05]
2.13e−13
I^2 = 0%
Replication
IHGC16noFIno23
0.04
[0.02; 0.07]
HUNT
0.02
[−0.02; 0.06]
Fixed effects model
0.04
[0.02; 0.06]
1.71e−04
I^2 = 0%
Fixed effects model
0.04
[0.03; 0.05]
1.57e−16
−0.06
−0.02
0
0.02
0.06

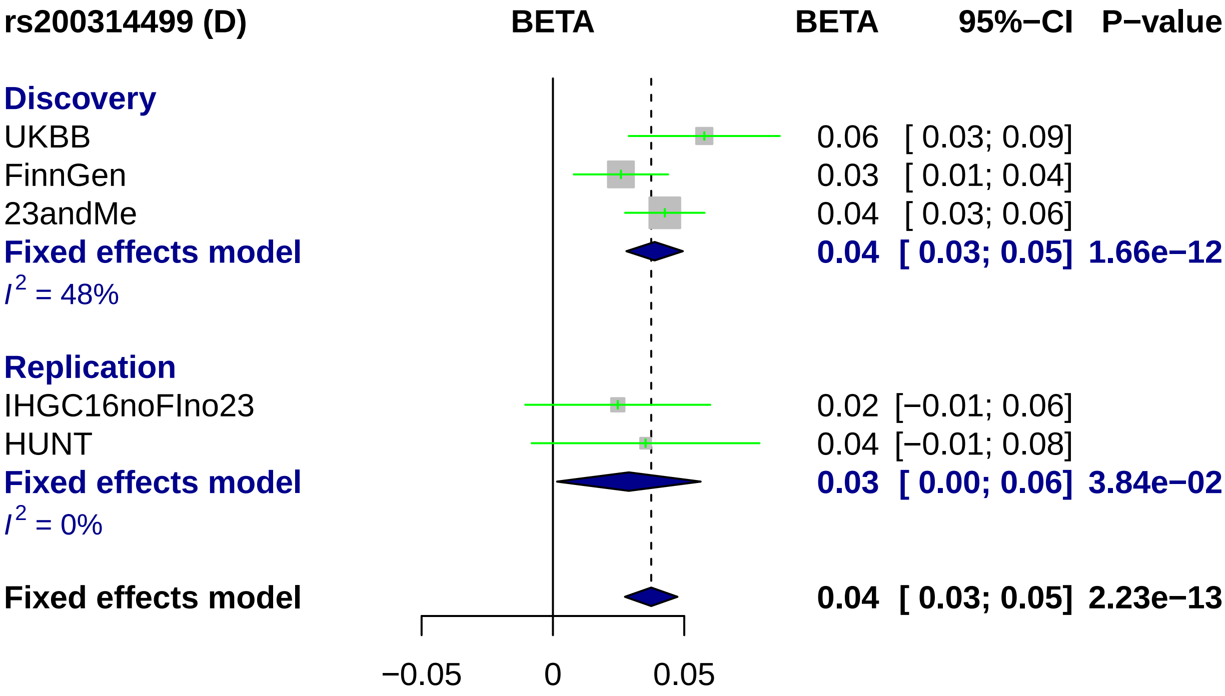
rs200314499 (D)
BETA
BETA
95%−CI
P−value
Discovery
UKBB
0.06 [0.03; 0.09]
FinnGen
0.03 [0.01; 0.04]
23andMe
0.04 [0.03; 0.06]
Fixed effects model
0.04 [0.03; 0.05] 1.66e−12
I² = 48%
Replication
IHGC16noFIno23
0.02 [−0.01; 0.06]
HUNT
0.04 [−0.01; 0.08]
Fixed effects model
0.03 [0.00; 0.06] 3.84e−02
I² = 0%
Fixed effects model
0.04 [0.03; 0.05] 2.23e−13
−0.05
0
0.05

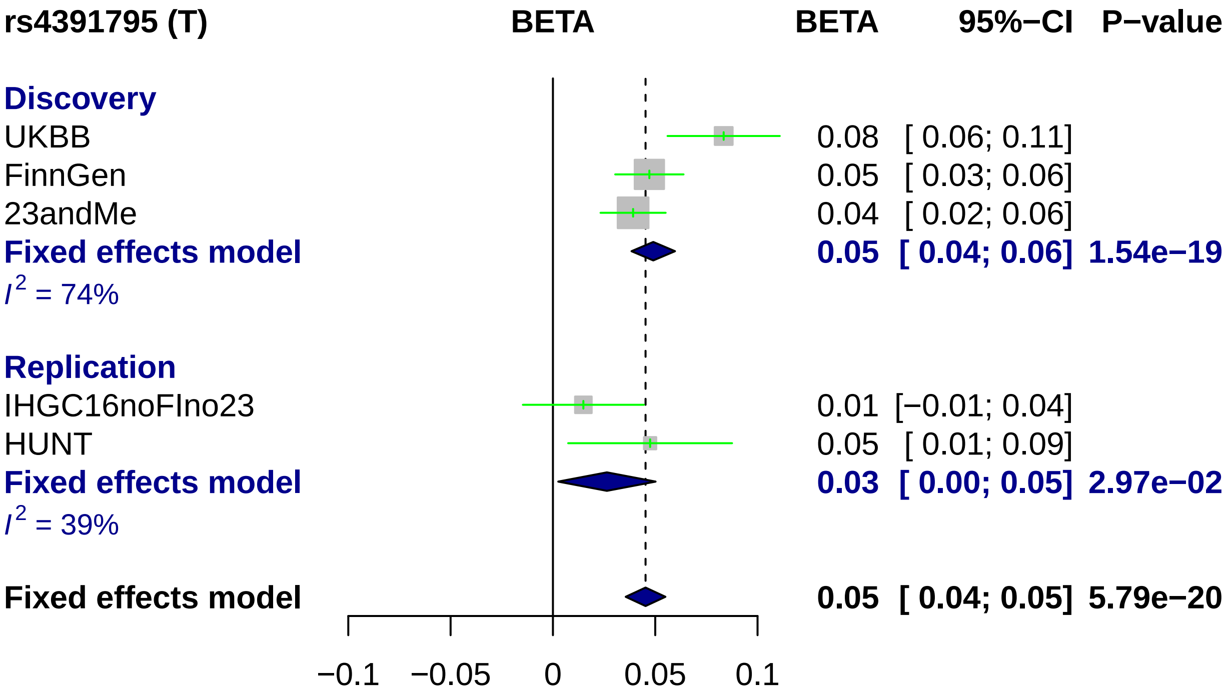
rs4391795 (T)
BETA
BETA
95%−CI
P−value
Discovery
UKBB
0.08
[0.06; 0.11]
FinnGen
0.05
[0.03; 0.06]
23andMe
0.04
[0.02; 0.06]
Fixed effects model
0.05
[0.04; 0.06]
1.54e−19
I2 = 74%
Replication
IHGC16noFIno23
0.01
[−0.01; 0.04]
HUNT
0.05
[0.01; 0.09]
Fixed effects model
0.03
[0.00; 0.05]
2.97e−02
I2 = 39%
Fixed effects model
0.05
[0.04; 0.05]
5.79e−20
−0.1
−0.05
0
0.05
0.1

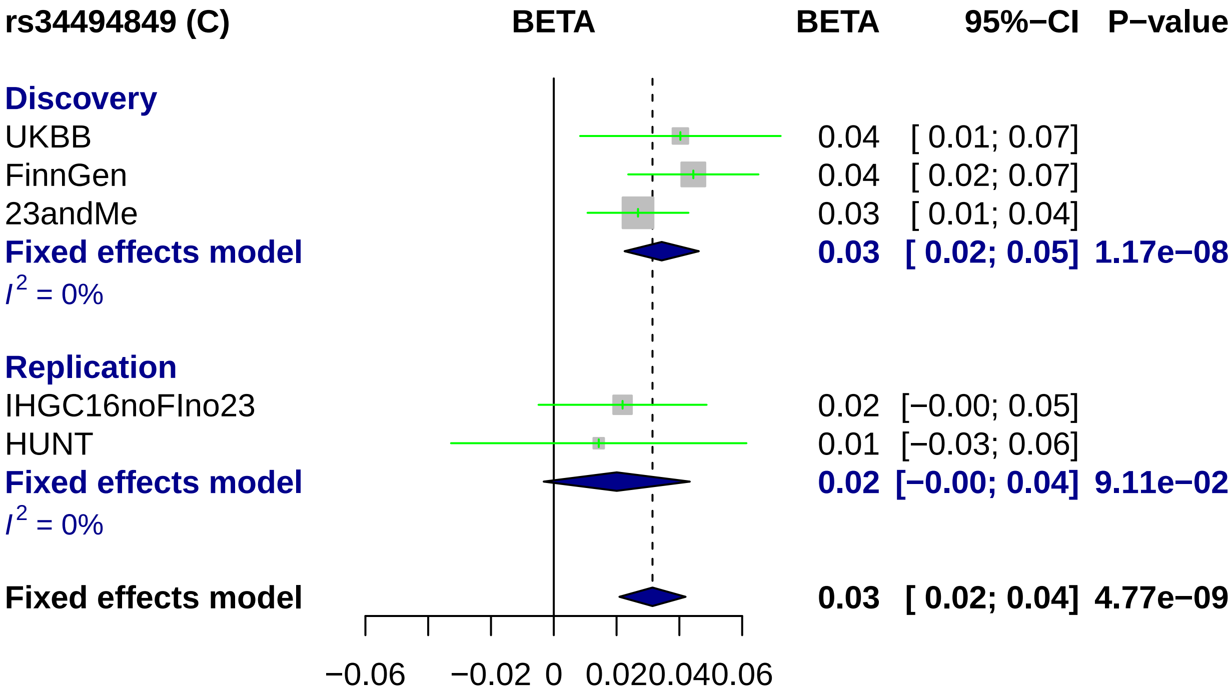
rs34494849 (C)
BETA
BETA
95%−CI
P−value
Discovery
UKBB
0.04 [0.01; 0.07]
FinnGen
0.04 [0.02; 0.07]
23andMe
0.03 [0.01; 0.04]
Fixed effects model
0.03 [0.02; 0.05] 1.17e−08
I2 = 0%
Replication
IHGC16noFIno23
0.02 [−0.00; 0.05]
HUNT
0.01 [−0.03; 0.06]
Fixed effects model
0.02 [−0.00; 0.04] 9.11e−02
I2 = 0%
Fixed effects model
0.03 [0.02; 0.04] 4.77e−09
−0.06
−0.02
0
0.02
0.04
0.06

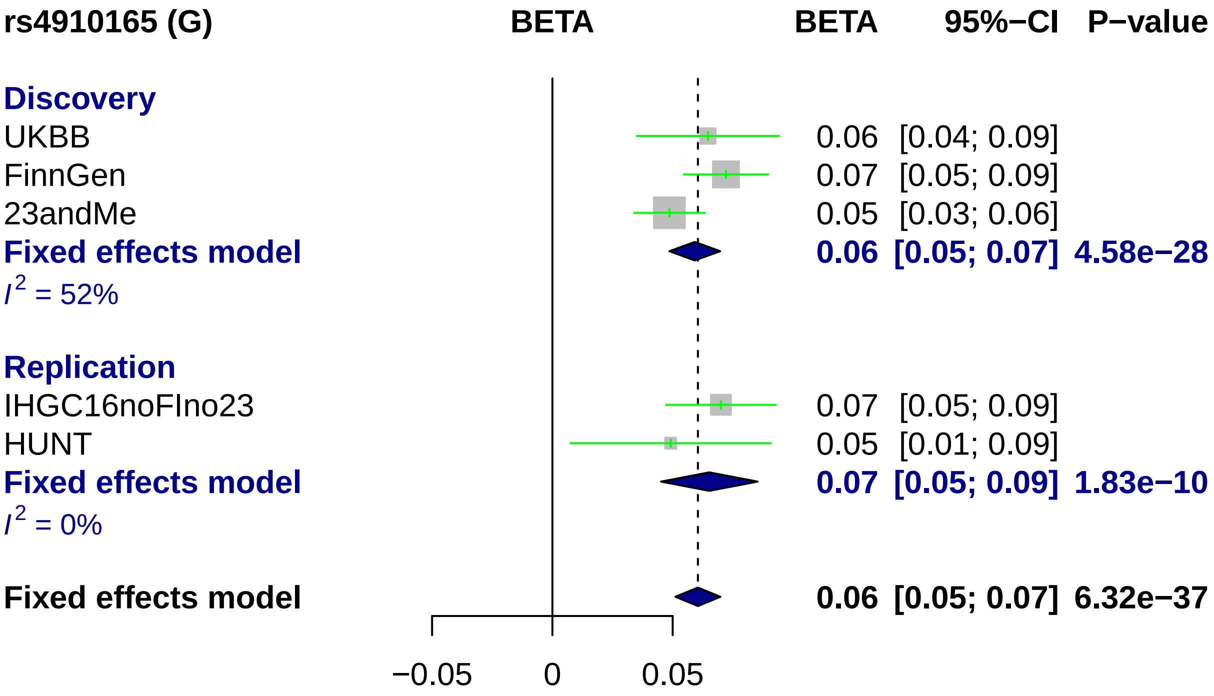

rs4910165 (G)
BETA
BETA
95%−CI
P−value
Discovery
UKBB
0.06 [0.04; 0.09]
FinnGen
0.07 [0.05; 0.09]
23andMe
0.05 [0.03; 0.06]
Fixed effects model
0.06 [0.05; 0.07] 4.58e−28
I2 = 52%
Replication
IHGC16noFIno23
0.07 [0.05; 0.09]
HUNT
0.05 [0.01; 0.09]
Fixed effects model
0.07 [0.05; 0.09] 1.83e−10
I2 = 0%
Fixed effects model
0.06 [0.05; 0.07] 6.32e−37
−0.05
0
0.05

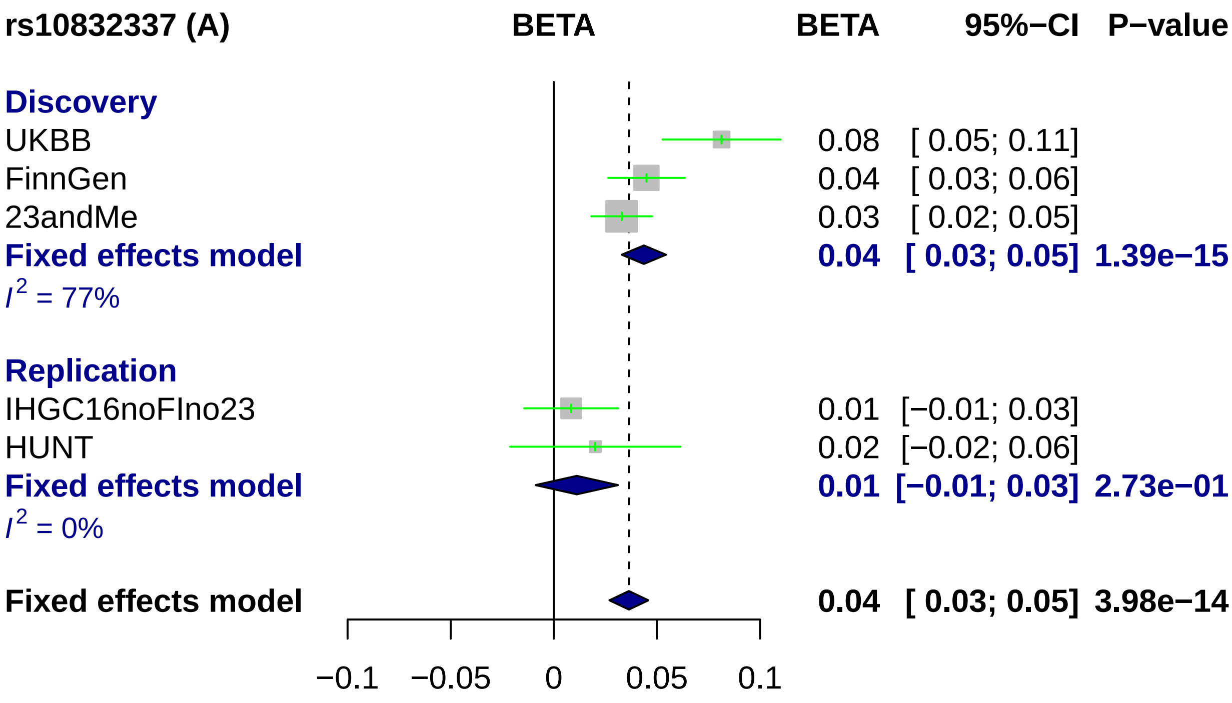
rs10832337 (A)
BETA
BETA
95%−CI
P−value
Discovery
UKBB
0.08
[0.05; 0.11]
FinnGen
0.04
[0.03; 0.06]
23andMe
0.03
[0.02; 0.05]
Fixed effects model
0.04
[0.03; 0.05]
1.39e−15
I2 = 77%
Replication
IHGC16noFIno23
0.01
[−0.01; 0.03]
HUNT
0.02
[−0.02; 0.06]
Fixed effects model
0.01
[−0.01; 0.03]
2.73e−01
I2 = 0%
Fixed effects model
0.04
[0.03; 0.05]
3.98e−14
−0.1
−0.05
0
0.05
0.1

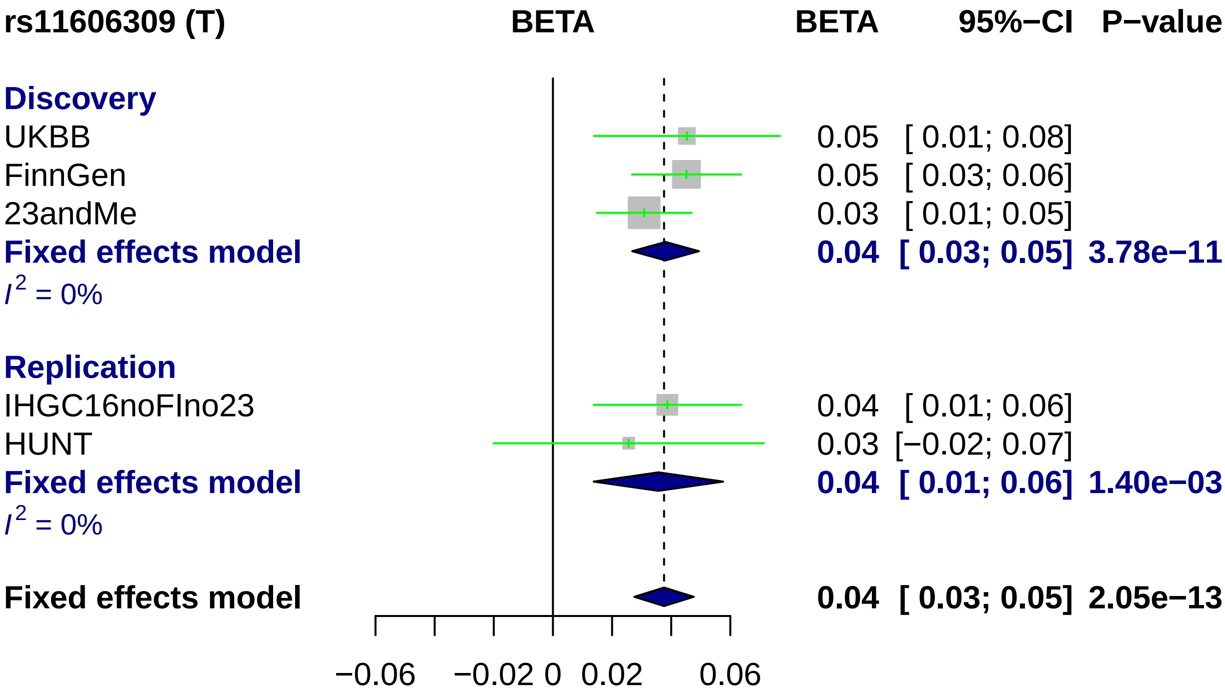
rs11606309 (T)
BETA
BETA
95%−CI
P−value
Discovery
UKBB
0.05
[0.01; 0.08]
FinnGen
0.05
[0.03; 0.06]
23andMe
0.03
[0.01; 0.05]
Fixed effects model
0.04
[0.03; 0.05]
3.78e−11
I2 = 0%
Replication
IHGC16noFIno23
0.04
[0.01; 0.06]
HUNT
0.03
[−0.02; 0.07]
Fixed effects model
0.04
[0.01; 0.06]
1.40e−03
I2 = 0%
Fixed effects model
0.04
[0.03; 0.05]
2.05e−13
−0.06
−0.02
0
0.02
0.06

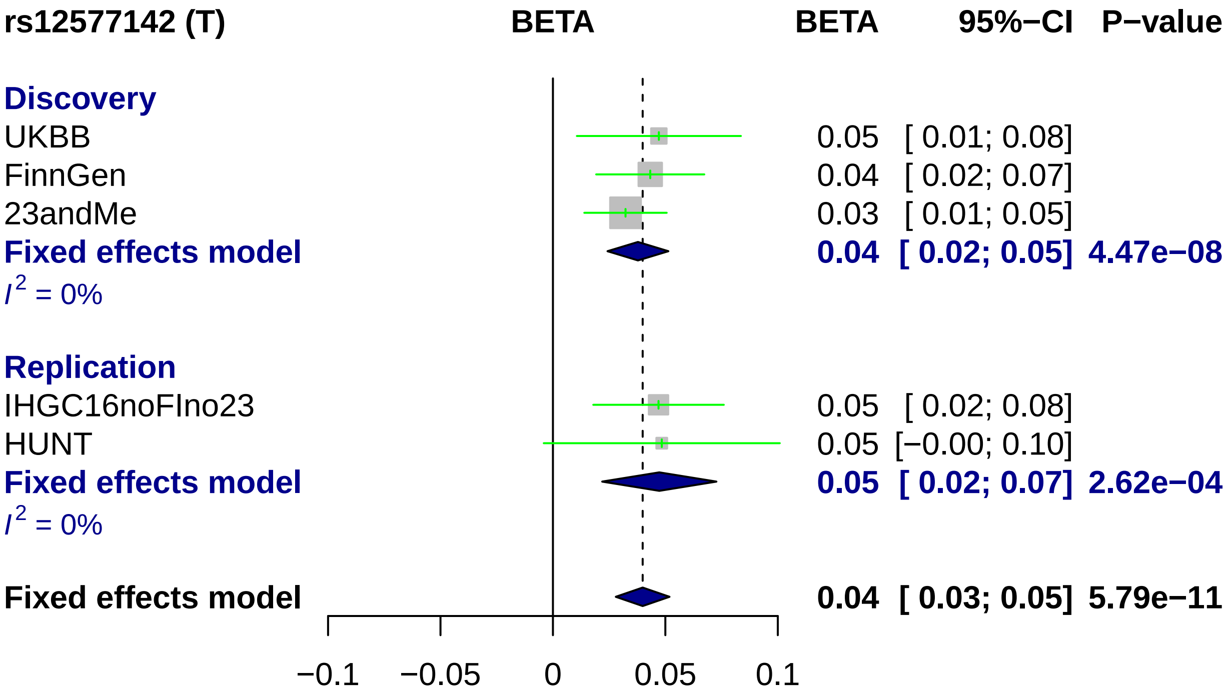
rs12577142 (T)
BETA
BETA
95%−CI
P−value
Discovery
UKBB 0.05 [0.01; 0.08]
FinnGen 0.04 [0.02; 0.07]
23andMe 0.03 [0.01; 0.05]
Fixed effects model 0.04 [0.02; 0.05] 4.47e−08
I^2 = 0%
Replication
IHGC16noFIno23 0.05 [0.02; 0.08]
HUNT 0.05 [−0.00; 0.10]
Fixed effects model 0.05 [0.02; 0.07] 2.62e−04
I^2 = 0%
Fixed effects model 0.04 [0.03; 0.05] 5.79e−11
−0.1
−0.05
0
0.05
0.1

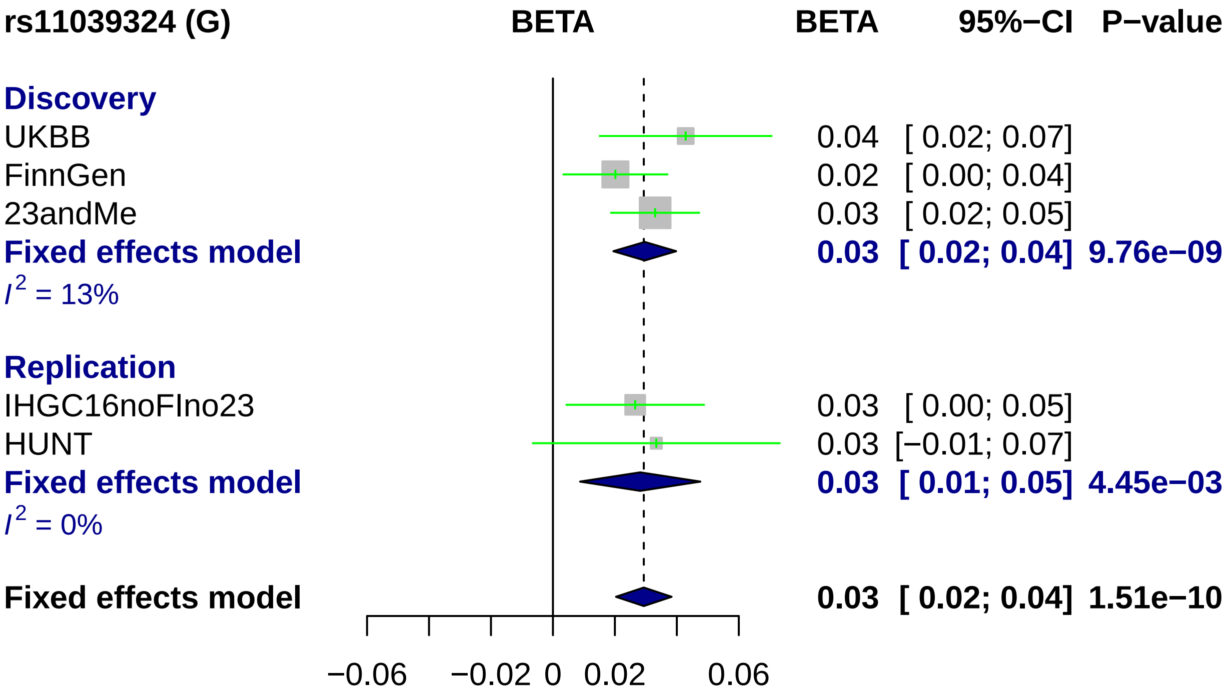
rs11039324 (G)
BETA
BETA
95%−CI
P−value
Discovery
UKBB
0.04 [0.02; 0.07]
FinnGen
0.02 [0.00; 0.04]
23andMe
0.03 [0.02; 0.05]
Fixed effects model
0.03 [0.02; 0.04] 9.76e−09
I2 = 13%
Replication
IHGC16noFIno23
0.03 [0.00; 0.05]
HUNT
0.03 [−0.01; 0.07]
Fixed effects model
0.03 [0.01; 0.05] 4.45e−03
I2 = 0%
Fixed effects model
0.03 [0.02; 0.04] 1.51e−10
−0.06
−0.02
0
0.02
0.06

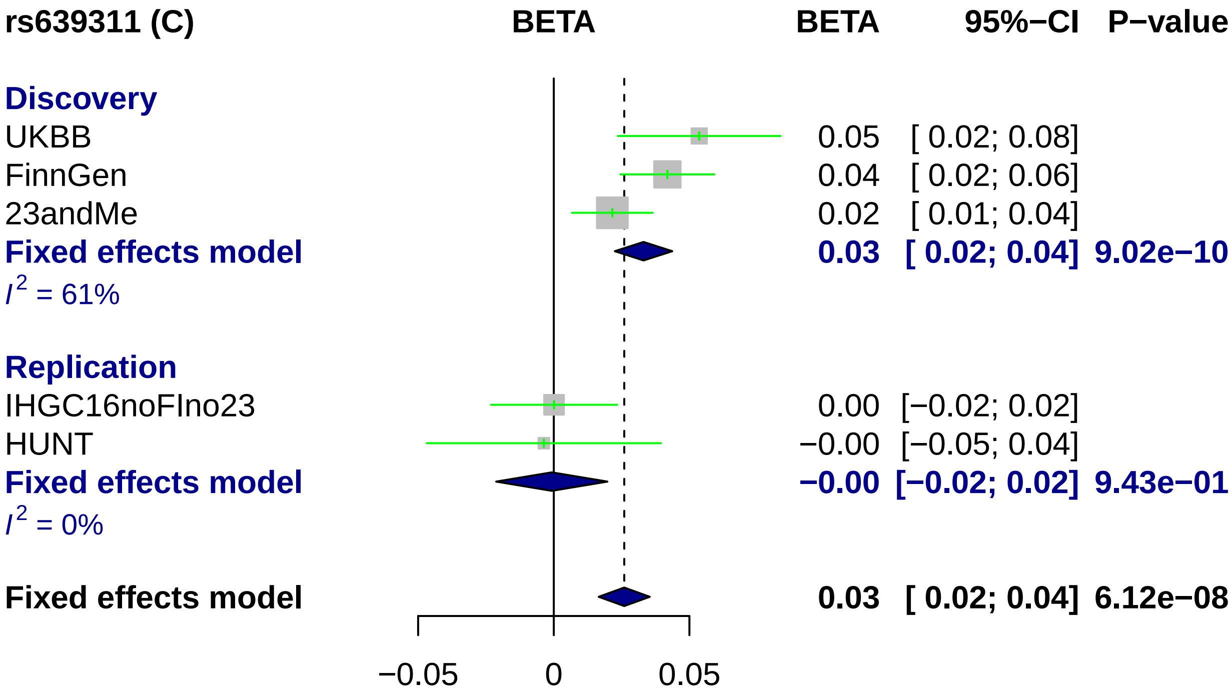
rs639311 (C)
BETA
BETA
95%−CI
P−value
Discovery
UKBB
0.05
[0.02; 0.08]
FinnGen
0.04
[0.02; 0.06]
23andMe
0.02
[0.01; 0.04]
Fixed effects model
0.03
[0.02; 0.04]
9.02e−10
I^2 = 61%
Replication
IHGC16noFIno23
0.00
[−0.02; 0.02]
HUNT
−0.00
[−0.05; 0.04]
Fixed effects model
−0.00
[−0.02; 0.02]
9.43e−01
I^2 = 0%
Fixed effects model
0.03
[0.02; 0.04]
6.12e−08
−0.05
0
0.05

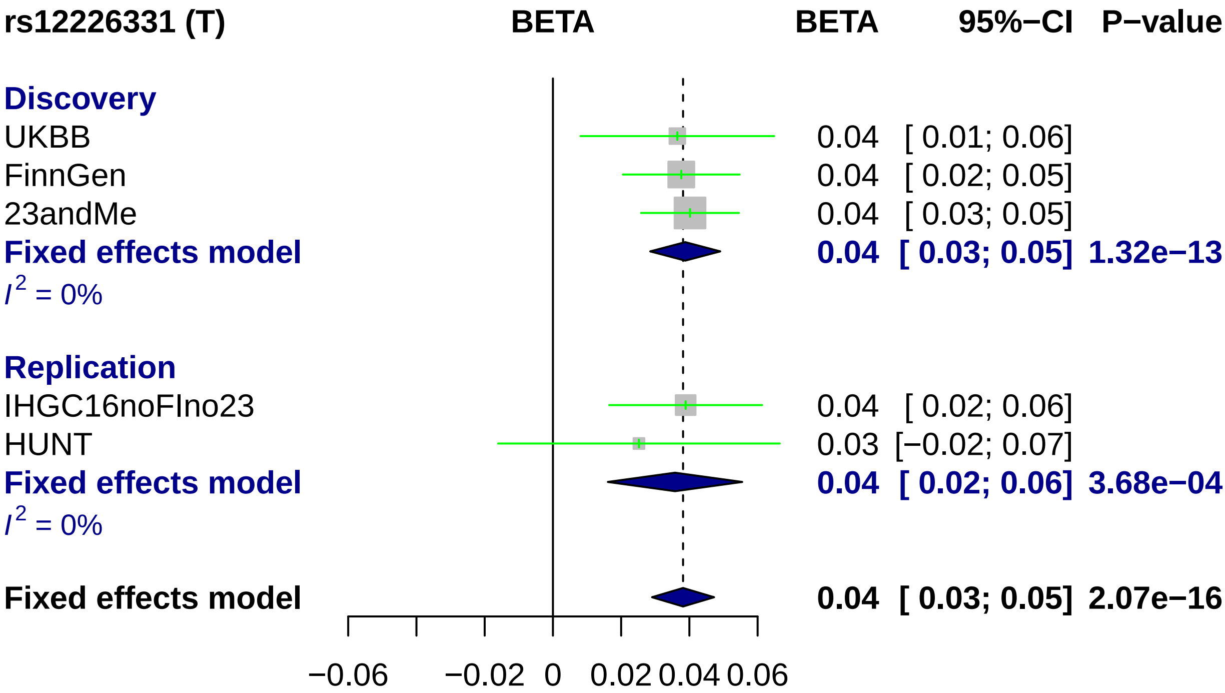

rs12226331 (T)
BETA
BETA
95%−CI
P−value
Discovery
UKBB
0.04 [0.01; 0.06]
FinnGen
0.04 [0.02; 0.05]
23andMe
0.04 [0.03; 0.05]
Fixed effects model
0.04 [0.03; 0.05] 1.32e−13
I2 = 0%
Replication
IHGC16noFIno23
0.04 [0.02; 0.06]
HUNT
0.03 [−0.02; 0.07]
Fixed effects model
0.04 [0.02; 0.06] 3.68e−04
I2 = 0%
Fixed effects model
0.04 [0.03; 0.05] 2.07e−16
−0.06
−0.02
0
0.02
0.04
0.06

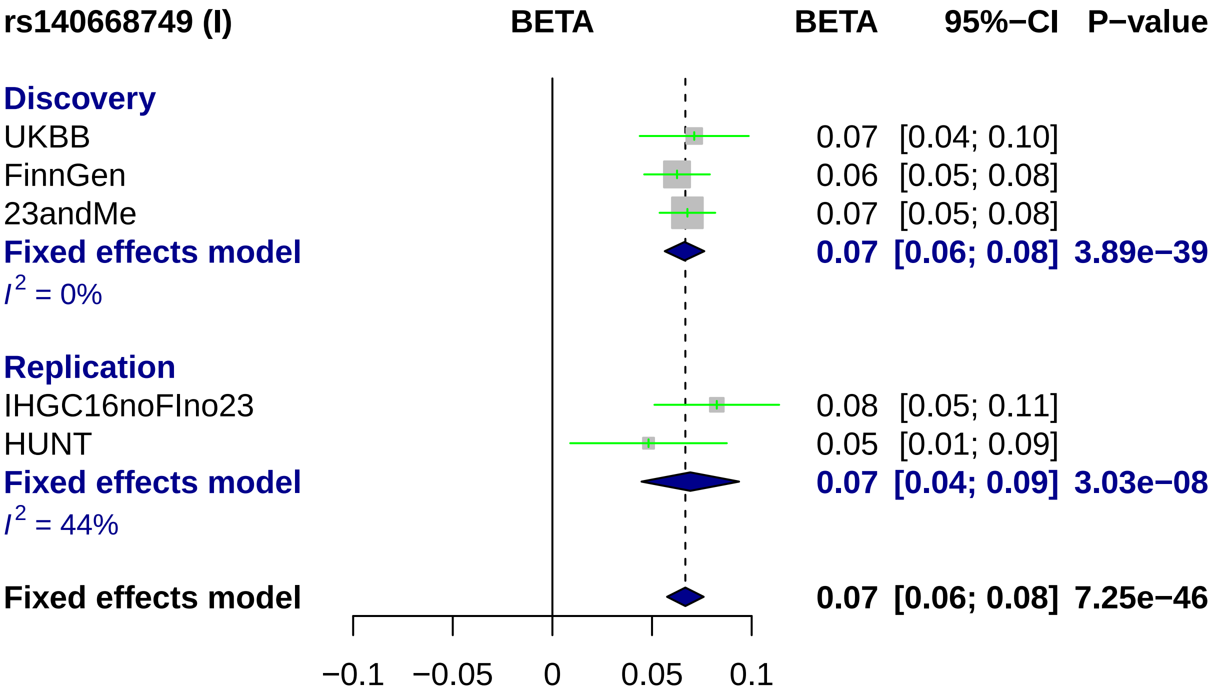
rs140668749 (I)
BETA
BETA
95%−CI
P−value
Discovery
UKBB
0.07
[0.04; 0.10]
FinnGen
0.06
[0.05; 0.08]
23andMe
0.07
[0.05; 0.08]
Fixed effects model
0.07
[0.06; 0.08]
3.89e−39
I2 = 0%
Replication
IHGC16noFIno23
0.08
[0.05; 0.11]
HUNT
0.05
[0.01; 0.09]
Fixed effects model
0.07
[0.04; 0.09]
3.03e−08
I2 = 44%
Fixed effects model
0.07
[0.06; 0.08]
7.25e−46
−0.1
−0.05
0
0.05
0.1

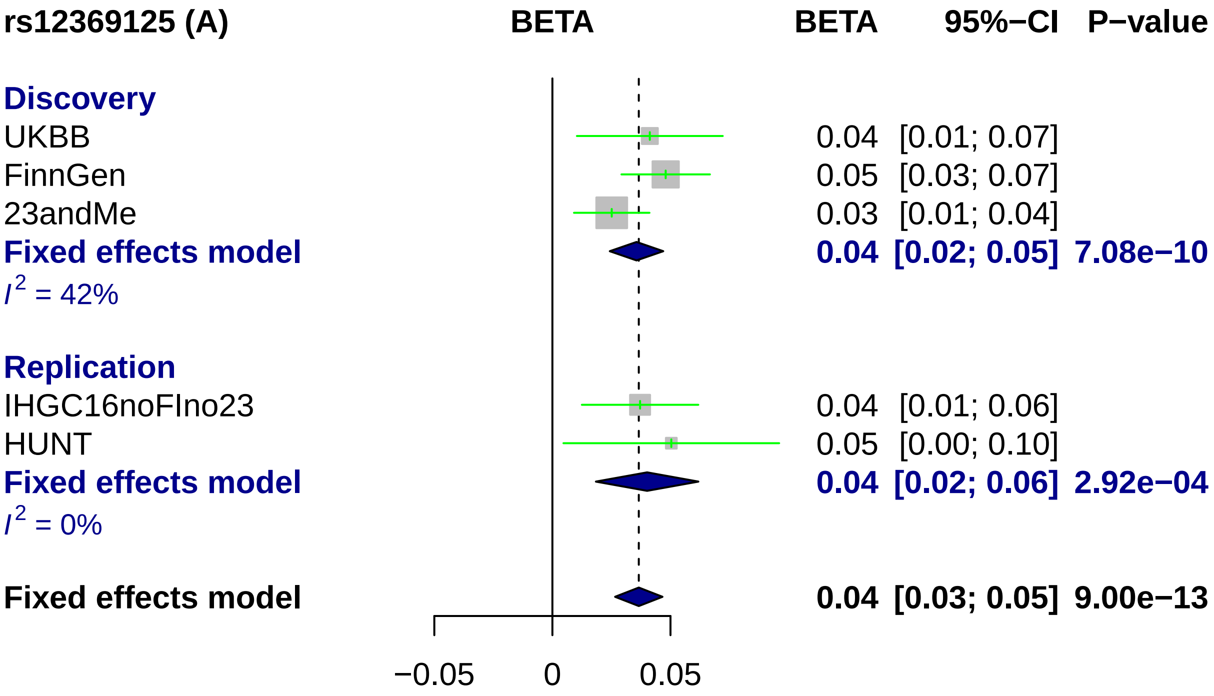
rs12369125 (A)
BETA
BETA
95%−CI
P−value
Discovery
UKBB
0.04 [0.01; 0.07]
FinnGen
0.05 [0.03; 0.07]
23andMe
0.03 [0.01; 0.04]
Fixed effects model
0.04 [0.02; 0.05] 7.08e−10
I2 = 42%
Replication
IHGC16noFIno23
0.04 [0.01; 0.06]
HUNT
0.05 [0.00; 0.10]
Fixed effects model
0.04 [0.02; 0.06] 2.92e−04
I2 = 0%
Fixed effects model
0.04 [0.03; 0.05] 9.00e−13
−0.05
0
0.05

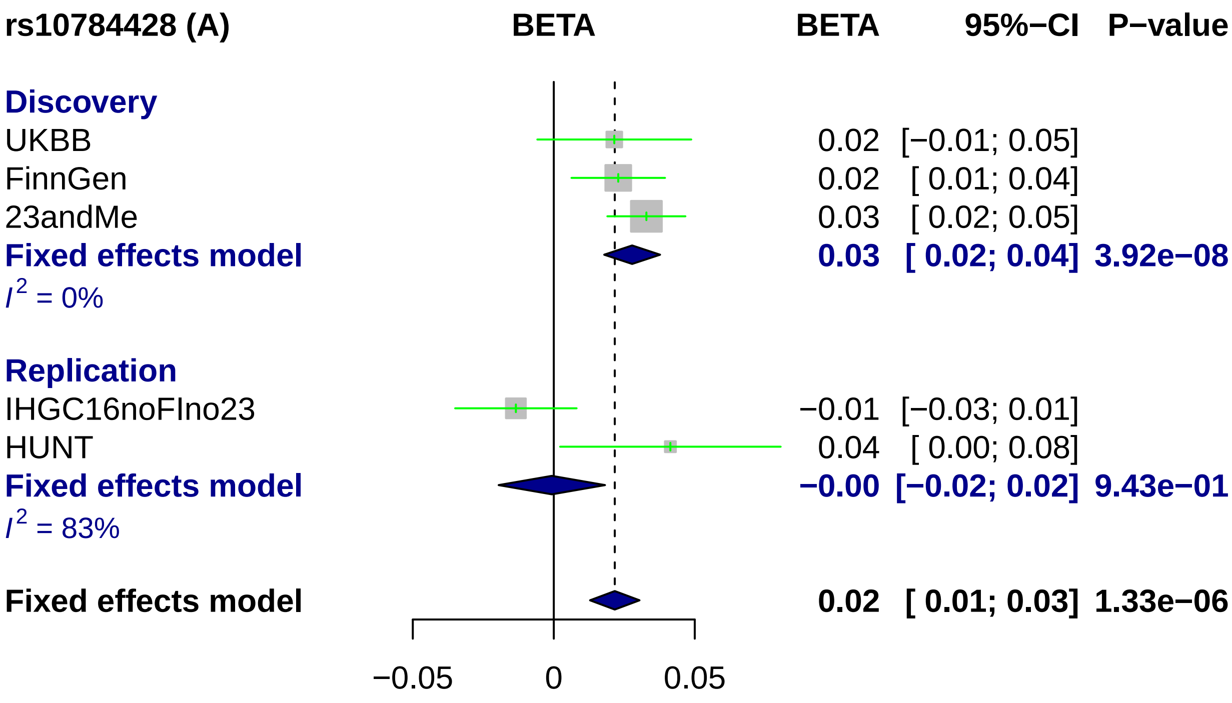
rs10784428 (A)
BETA
BETA
95%−CI
P−value
Discovery
UKBB
0.02 [−0.01; 0.05]
FinnGen
0.02 [0.01; 0.04]
23andMe
0.03 [0.02; 0.05]
Fixed effects model
0.03 [0.02; 0.04] 3.92e−08
I2 = 0%
Replication
IHGC16noFIno23
−0.01 [−0.03; 0.01]
HUNT
0.04 [0.00; 0.08]
Fixed effects model
−0.00 [−0.02; 0.02] 9.43e−01
I2 = 83%
Fixed effects model
0.02 [0.01; 0.03] 1.33e−06
−0.05
0
0.05

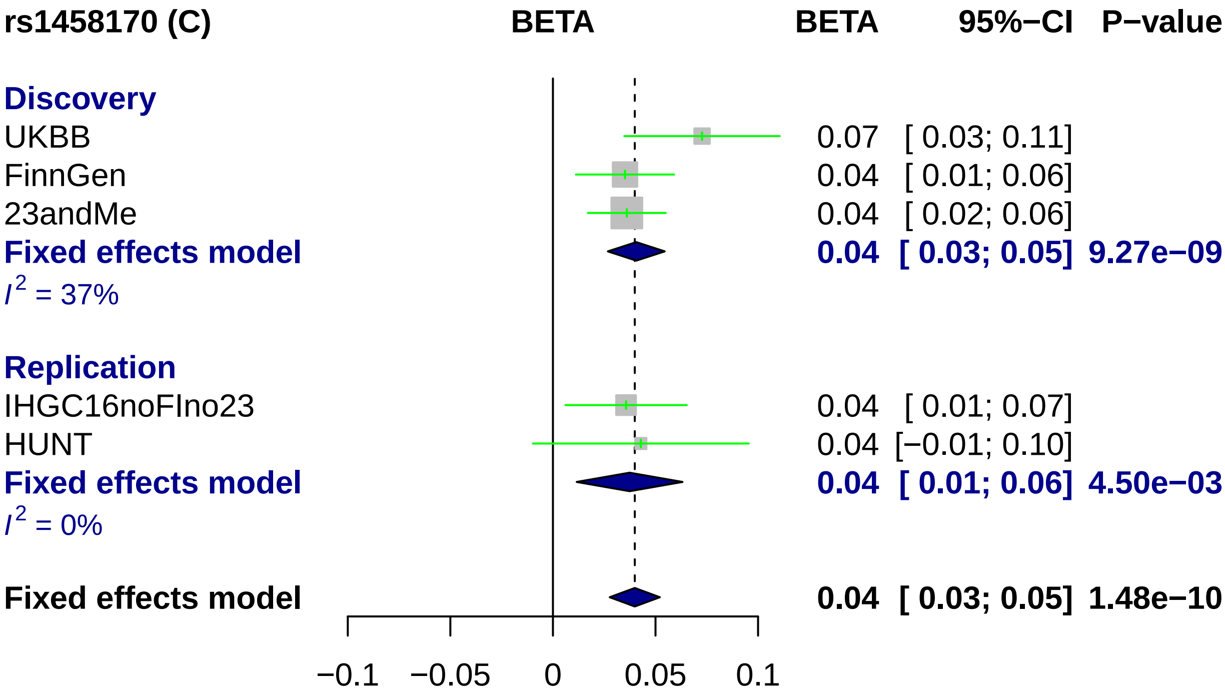
rs1458170 (C)
BETA
BETA
95%−CI
P−value
Discovery
UKBB
0.07
[0.03; 0.11]
FinnGen
0.04
[0.01; 0.06]
23andMe
0.04
[0.02; 0.06]
Fixed effects model
0.04
[0.03; 0.05]
9.27e−09
I2 = 37%
Replication
IHGC16noFIno23
0.04
[0.01; 0.07]
HUNT
0.04
[−0.01; 0.10]
Fixed effects model
0.04
[0.01; 0.06]
4.50e−03
I2 = 0%
Fixed effects model
0.04
[0.03; 0.05]
1.48e−10
−0.1
−0.05
0
0.05
0.1

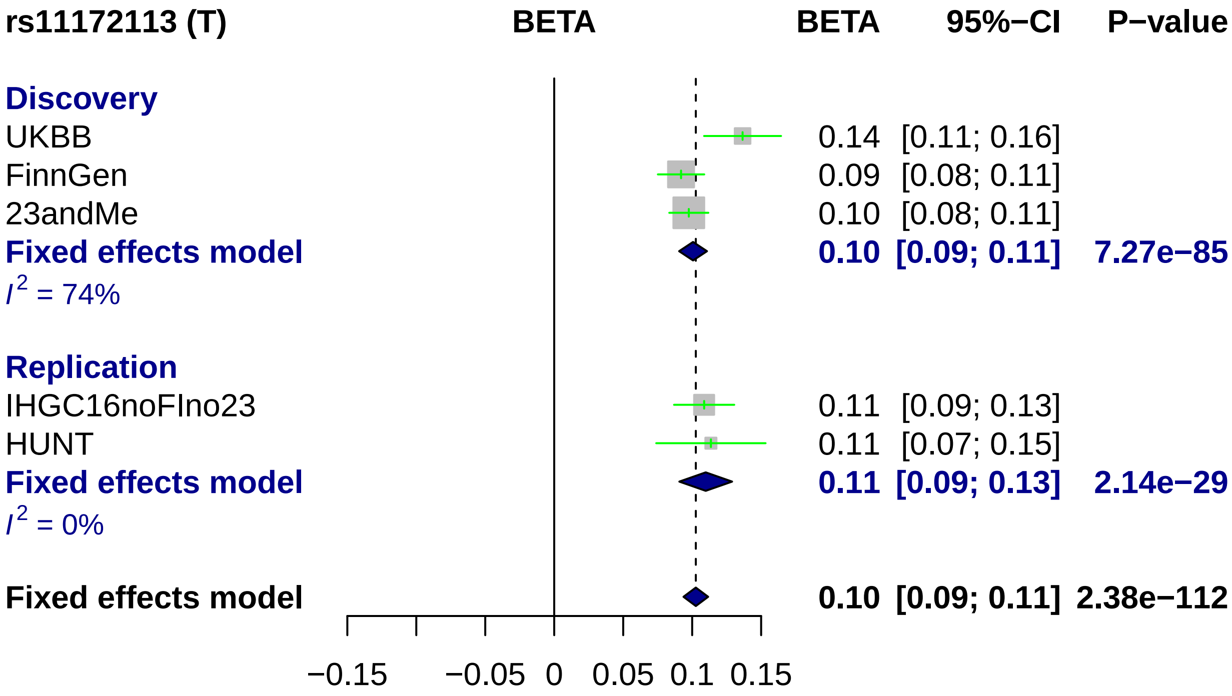

rs11172113 (T)
BETA
BETA
95%−CI
P−value
Discovery
UKBB
0.14 [0.11; 0.16]
FinnGen
0.09 [0.08; 0.11]
23andMe
0.10 [0.08; 0.11]
Fixed effects model
0.10 [0.09; 0.11] 7.27e−85
I^2 = 74%
Replication
IHGC16noFIno23
0.11 [0.09; 0.13]
HUNT
0.11 [0.07; 0.15]
Fixed effects model
0.11 [0.09; 0.13] 2.14e−29
I^2 = 0%
Fixed effects model
0.10 [0.09; 0.11] 2.38e−112
−0.15
−0.05
0
0.05
0.1
0.15

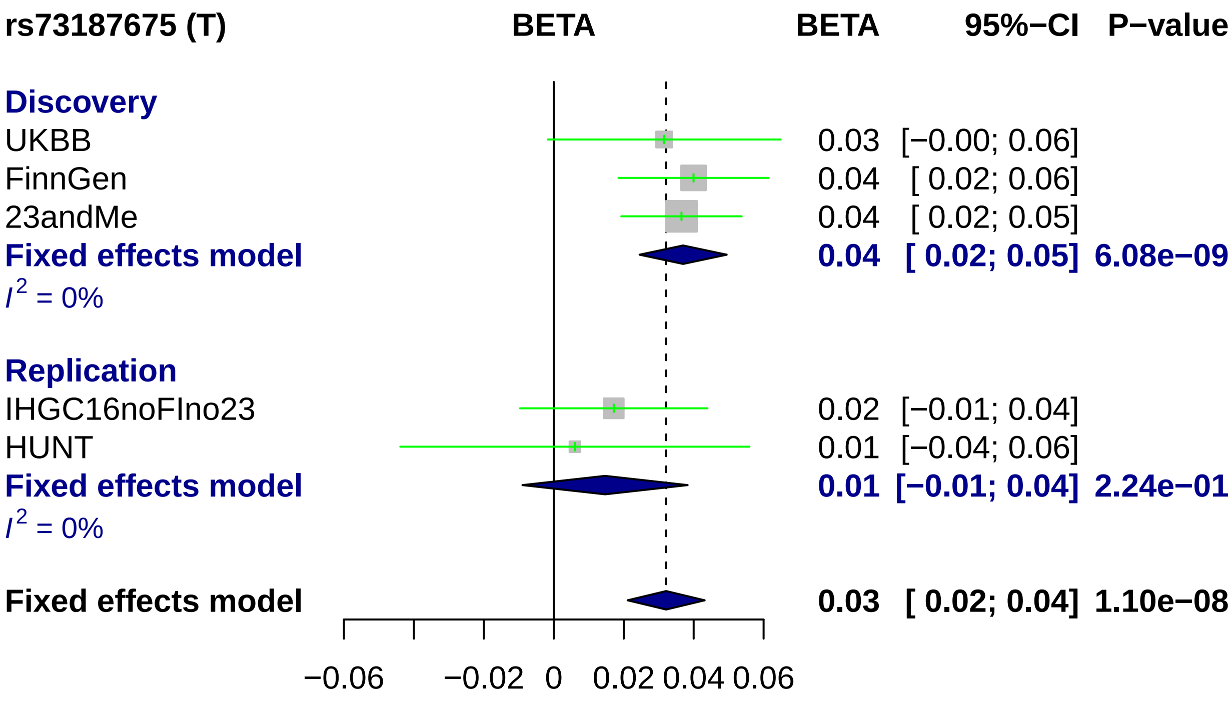

rs73187675 (T)
BETA
BETA
95%−CI
P−value
Discovery
UKBB
0.03 [−0.00; 0.06]
FinnGen
0.04 [0.02; 0.06]
23andMe
0.04 [0.02; 0.05]
Fixed effects model
0.04 [0.02; 0.05] 6.08e−09
I2 = 0%
Replication
IHGC16noFIno23
0.02 [−0.01; 0.04]
HUNT
0.01 [−0.04; 0.06]
Fixed effects model
0.01 [−0.01; 0.04] 2.24e−01
I2 = 0%
Fixed effects model
0.03 [0.02; 0.04] 1.10e−08
−0.06
−0.02
0
0.02
0.04
0.06

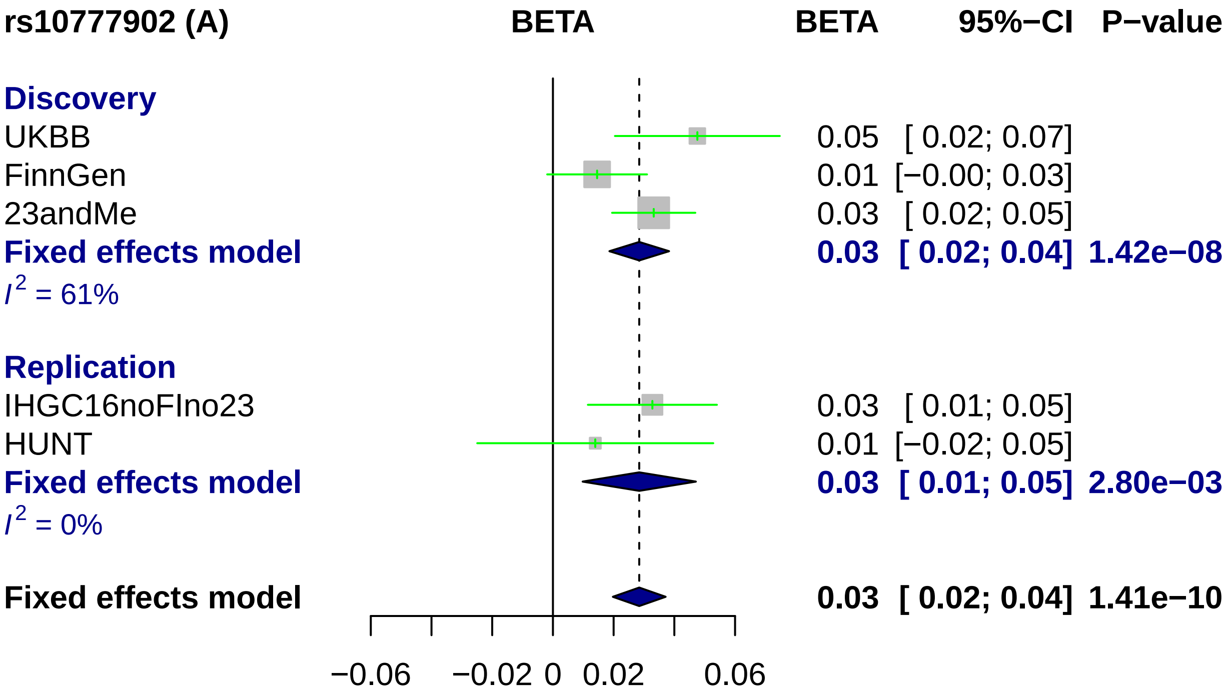
rs10777902 (A)
BETA
BETA
95%−CI
P−value
Discovery
UKBB
0.05 [0.02; 0.07]
FinnGen
0.01 [−0.00; 0.03]
23andMe
0.03 [0.02; 0.05]
Fixed effects model
0.03 [0.02; 0.04] 1.42e−08
I2 = 61%
Replication
IHGC16noFIno23
0.03 [0.01; 0.05]
HUNT
0.01 [−0.02; 0.05]
Fixed effects model
0.03 [0.01; 0.05] 2.80e−03
I2 = 0%
Fixed effects model
0.03 [0.02; 0.04] 1.41e−10
−0.06
−0.02
0
0.02
0.06

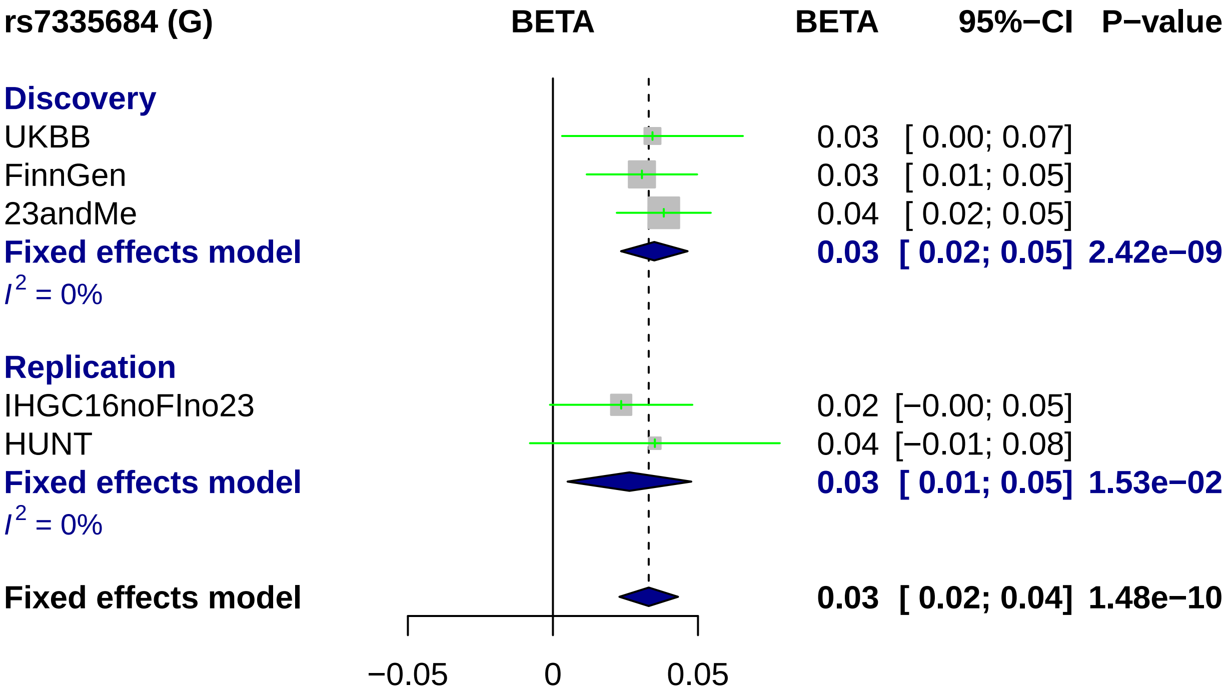
rs7335684 (G)
BETA
BETA
95%−CI
P−value
Discovery
UKBB
0.03 [0.00; 0.07]
FinnGen
0.03 [0.01; 0.05]
23andMe
0.04 [0.02; 0.05]
Fixed effects model
0.03 [0.02; 0.05] 2.42e−09
I2 = 0%
Replication
IHGC16noFIno23
0.02 [−0.00; 0.05]
HUNT
0.04 [−0.01; 0.08]
Fixed effects model
0.03 [0.01; 0.05] 1.53e−02
I2 = 0%
Fixed effects model
0.03 [0.02; 0.04] 1.48e−10
−0.05
0
0.05

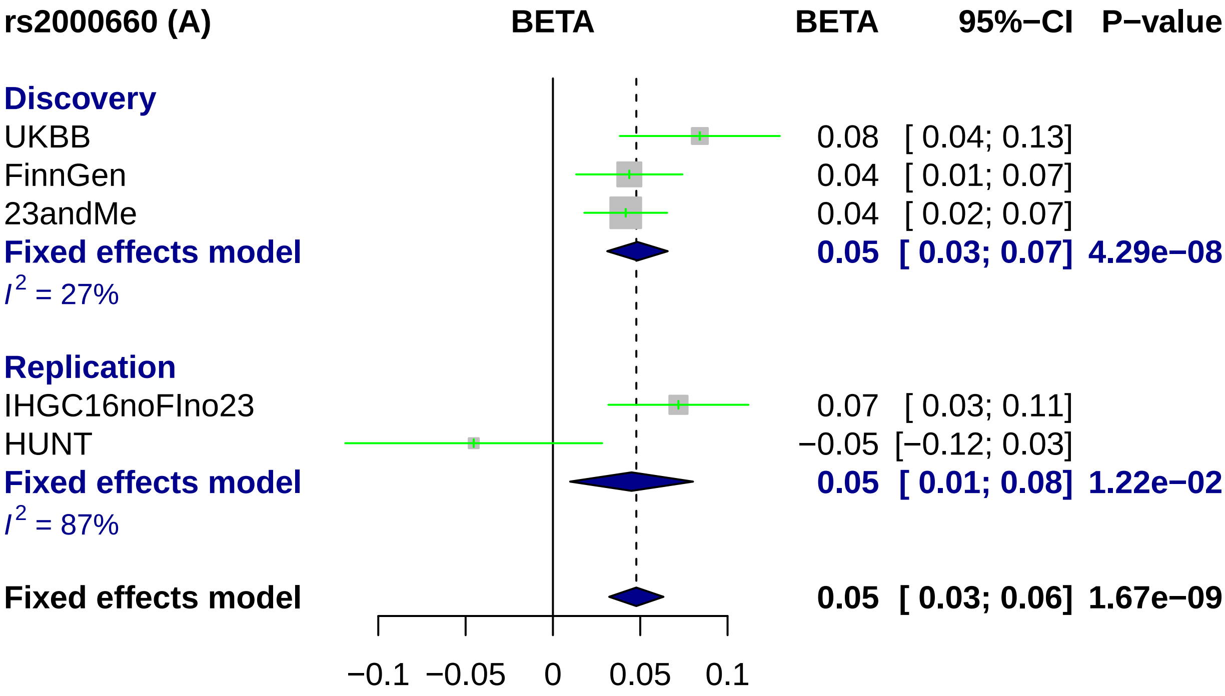
rs2000660 (A)
BETA
BETA
95%−CI
P−value
Discovery
UKBB
0.08
[0.04; 0.13]
FinnGen
0.04
[0.01; 0.07]
23andMe
0.04
[0.02; 0.07]
Fixed effects model
0.05
[0.03; 0.07]
4.29e−08
I2 = 27%
Replication
IHGC16noFIno23
0.07
[0.03; 0.11]
HUNT
−0.05
[−0.12; 0.03]
Fixed effects model
0.05
[0.01; 0.08]
1.22e−02
I2 = 87%
Fixed effects model
0.05
[0.03; 0.06]
1.67e−09
−0.1
−0.05
0
0.05
0.1

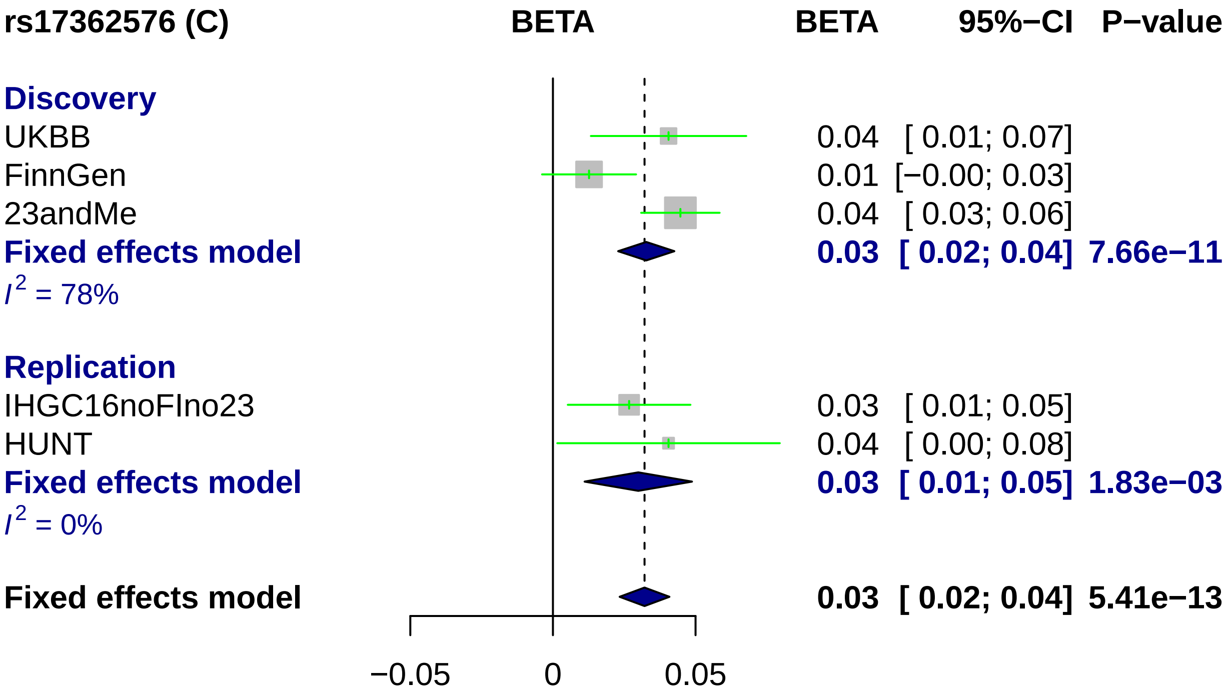
rs17362576 (C)
BETA
BETA
95%−CI
P−value
Discovery
UKBB
0.04 [0.01; 0.07]
FinnGen
0.01 [−0.00; 0.03]
23andMe
0.04 [0.03; 0.06]
Fixed effects model
0.03 [0.02; 0.04] 7.66e−11
I2 = 78%
Replication
IHGC16noFIno23
0.03 [0.01; 0.05]
HUNT
0.04 [0.00; 0.08]
Fixed effects model
0.03 [0.01; 0.05] 1.83e−03
I2 = 0%
Fixed effects model
0.03 [0.02; 0.04] 5.41e−13
−0.05
0
0.05

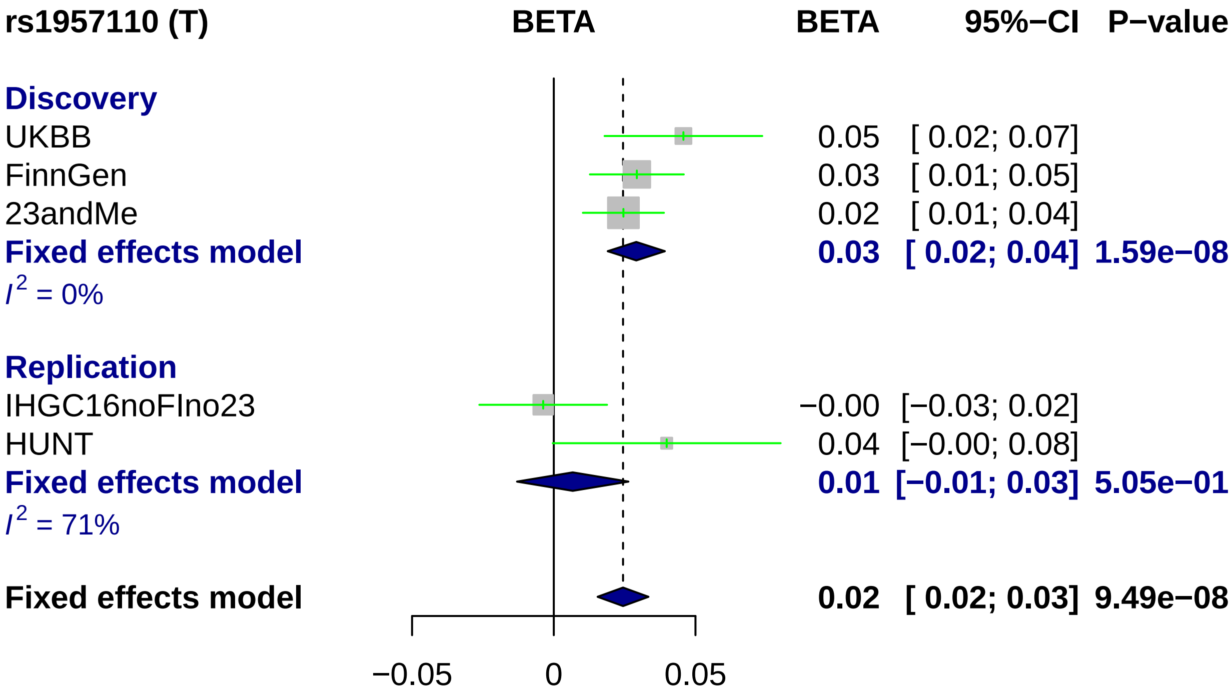
rs1957110 (T)
BETA
BETA
95%−CI
P−value
Discovery
UKBB
0.05
[0.02; 0.07]
FinnGen
0.03
[0.01; 0.05]
23andMe
0.02
[0.01; 0.04]
Fixed effects model
0.03
[0.02; 0.04]
1.59e−08
I^2 = 0%
Replication
IHGC16noFIno23
−0.00
[−0.03; 0.02]
HUNT
0.04
[−0.00; 0.08]
Fixed effects model
0.01
[−0.01; 0.03]
5.05e−01
I^2 = 71%
Fixed effects model
0.02
[0.02; 0.03]
9.49e−08
−0.05
0
0.05

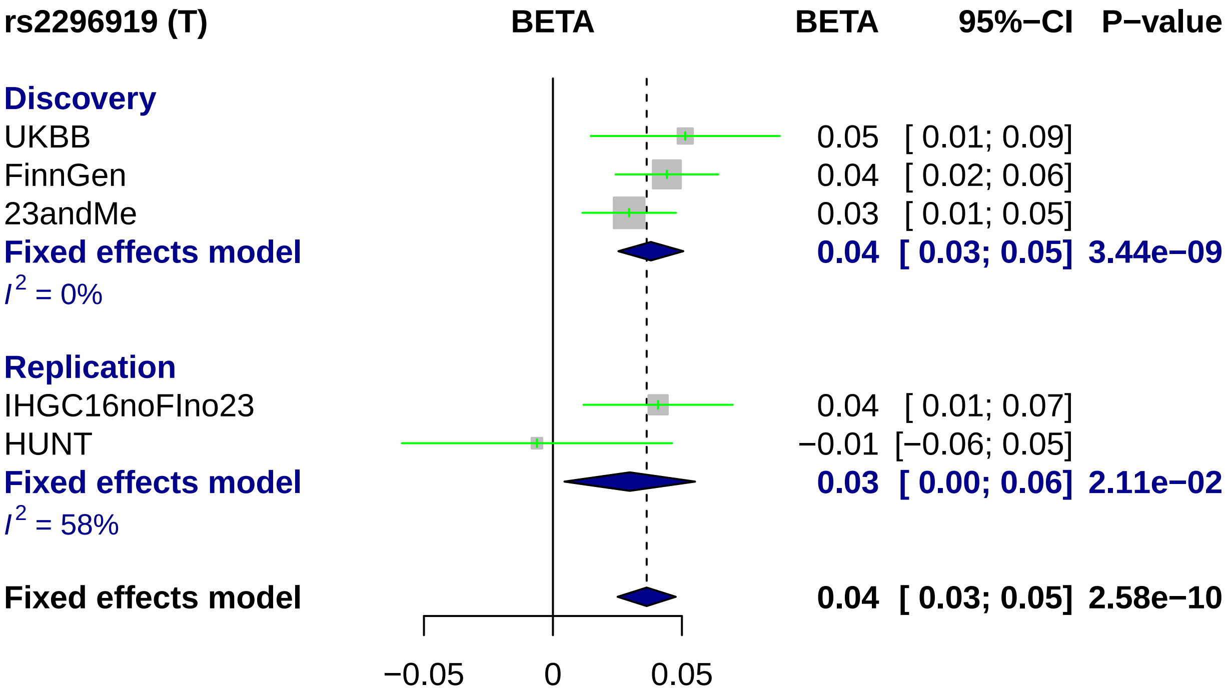
rs2296919 (T)
BETA
BETA 95%−CI P−value
Discovery
UKBB 0.05 [0.01; 0.09]
FinnGen 0.04 [0.02; 0.06]
23andMe 0.03 [0.01; 0.05]
Fixed effects model 0.04 [0.03; 0.05] 3.44e−09
I^2 = 0%
Replication
IHGC16noFIno23 0.04 [0.01; 0.07]
HUNT −0.01 [−0.06; 0.05]
Fixed effects model 0.03 [0.00; 0.06] 2.11e−02
I^2 = 58%
Fixed effects model 0.04 [0.03; 0.05] 2.58e−10
−0.05
0
0.05

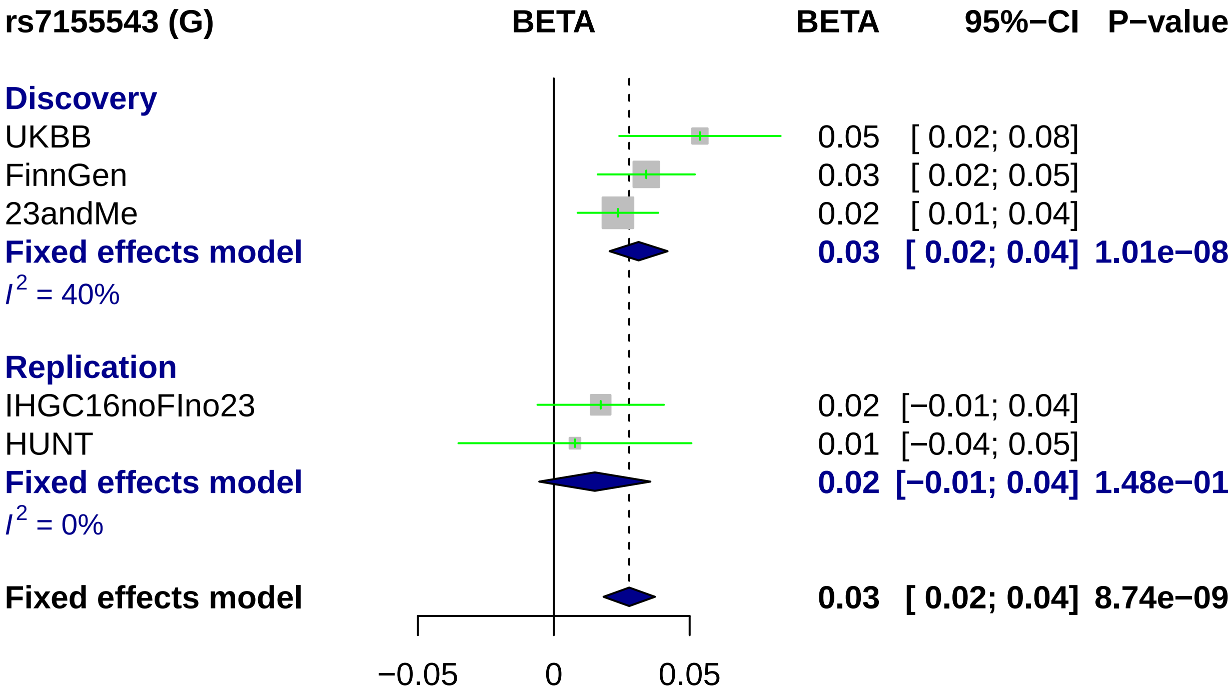
rs7155543 (G)
BETA
BETA
95%−CI
P−value
Discovery
UKBB
0.05 [0.02; 0.08]
FinnGen
0.03 [0.02; 0.05]
23andMe
0.02 [0.01; 0.04]
Fixed effects model
0.03 [0.02; 0.04] 1.01e−08
I2 = 40%
Replication
IHGC16noFIno23
0.02 [−0.01; 0.04]
HUNT
0.01 [−0.04; 0.05]
Fixed effects model
0.02 [−0.01; 0.04] 1.48e−01
I2 = 0%
Fixed effects model
0.03 [0.02; 0.04] 8.74e−09
−0.05
0
0.05

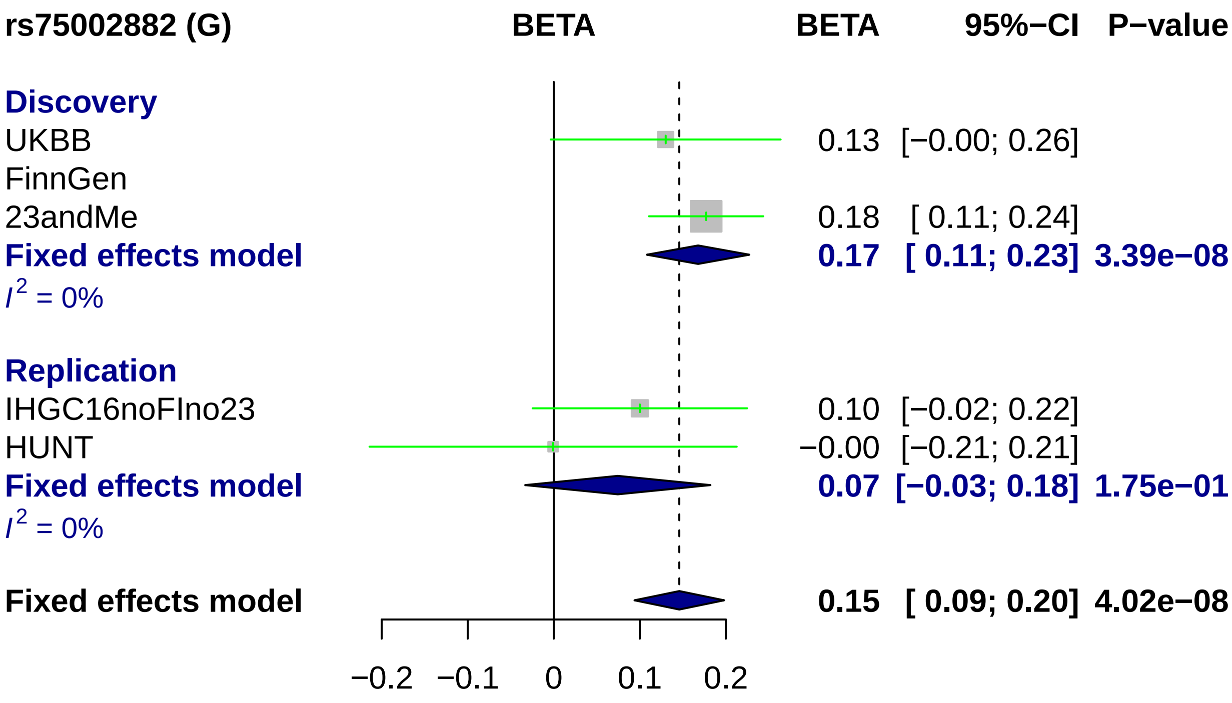

rs75002882 (G)
BETA
BETA
95%−CI
P−value
Discovery
UKBB
0.13 [−0.00; 0.26]
FinnGen
23andMe
0.18 [0.11; 0.24]
Fixed effects model
0.17 [0.11; 0.23] 3.39e−08
I² = 0%
Replication
IHGC16noFIno23
0.10 [−0.02; 0.22]
HUNT
−0.00 [−0.21; 0.21]
Fixed effects model
0.07 [−0.03; 0.18] 1.75e−01
I² = 0%
Fixed effects model
0.15 [0.09; 0.20] 4.02e−08
−0.2
−0.1
0
0.1
0.2

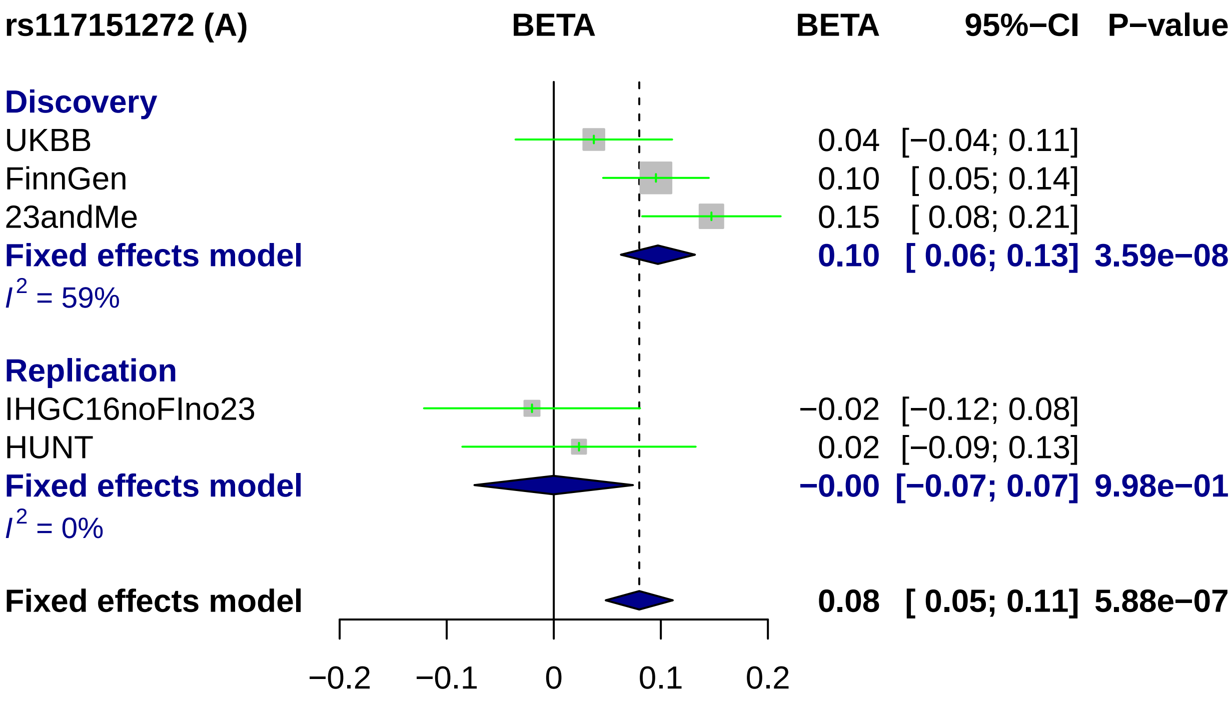

rs117151272 (A)
BETA
BETA
95%−CI
P−value
Discovery
UKBB
0.04
[−0.04; 0.11]
FinnGen
0.10
[0.05; 0.14]
23andMe
0.15
[0.08; 0.21]
Fixed effects model
0.10
[0.06; 0.13]
3.59e−08
I2 = 59%
Replication
IHGC16noFIno23
−0.02
[−0.12; 0.08]
HUNT
0.02
[−0.09; 0.13]
Fixed effects model
−0.00
[−0.07; 0.07]
9.98e−01
I2 = 0%
Fixed effects model
0.08
[0.05; 0.11]
5.88e−07
−0.2
−0.1
0
0.1
0.2

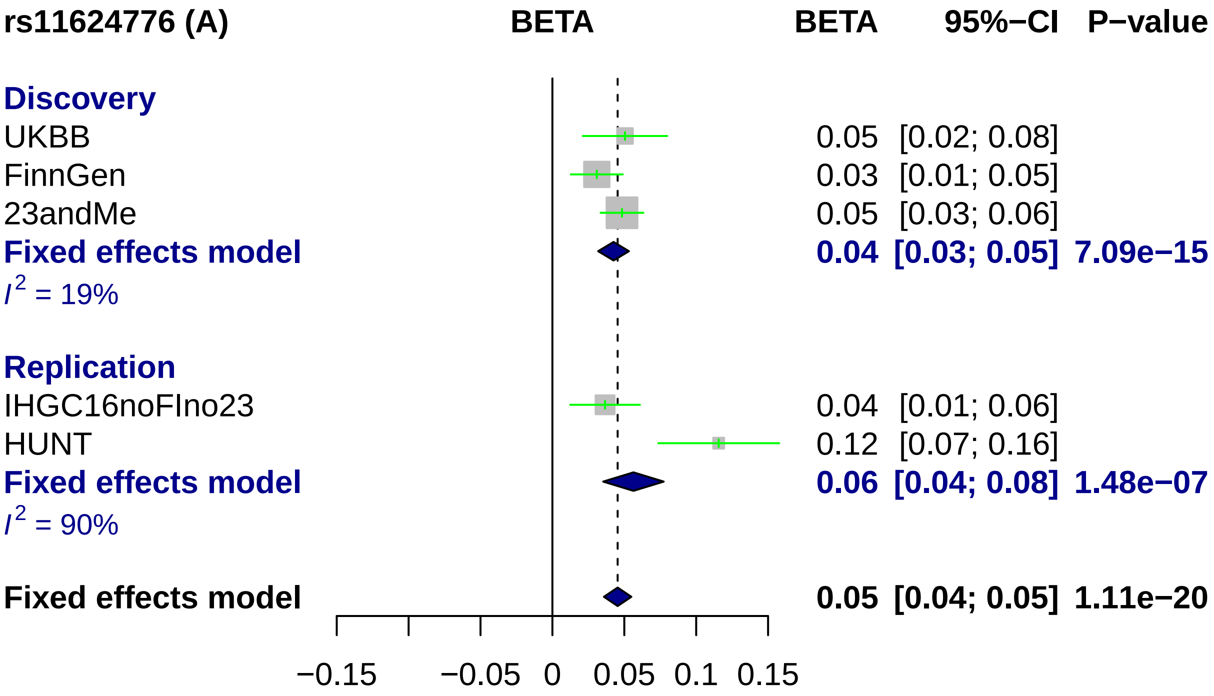
rs11624776 (A)
BETA
BETA 95%−CI P−value
Discovery
UKBB 0.05 [0.02; 0.08]
FinnGen 0.03 [0.01; 0.05]
23andMe 0.05 [0.03; 0.06]
Fixed effects model 0.04 [0.03; 0.05] 7.09e−15
I^2 = 19%
Replication
IHGC16noFIno23 0.04 [0.01; 0.06]
HUNT 0.12 [0.07; 0.16]
Fixed effects model 0.06 [0.04; 0.08] 1.48e−07
I^2 = 90%
Fixed effects model 0.05 [0.04; 0.05] 1.11e−20
−0.15 −0.05 0 0.05 0.1 0.15

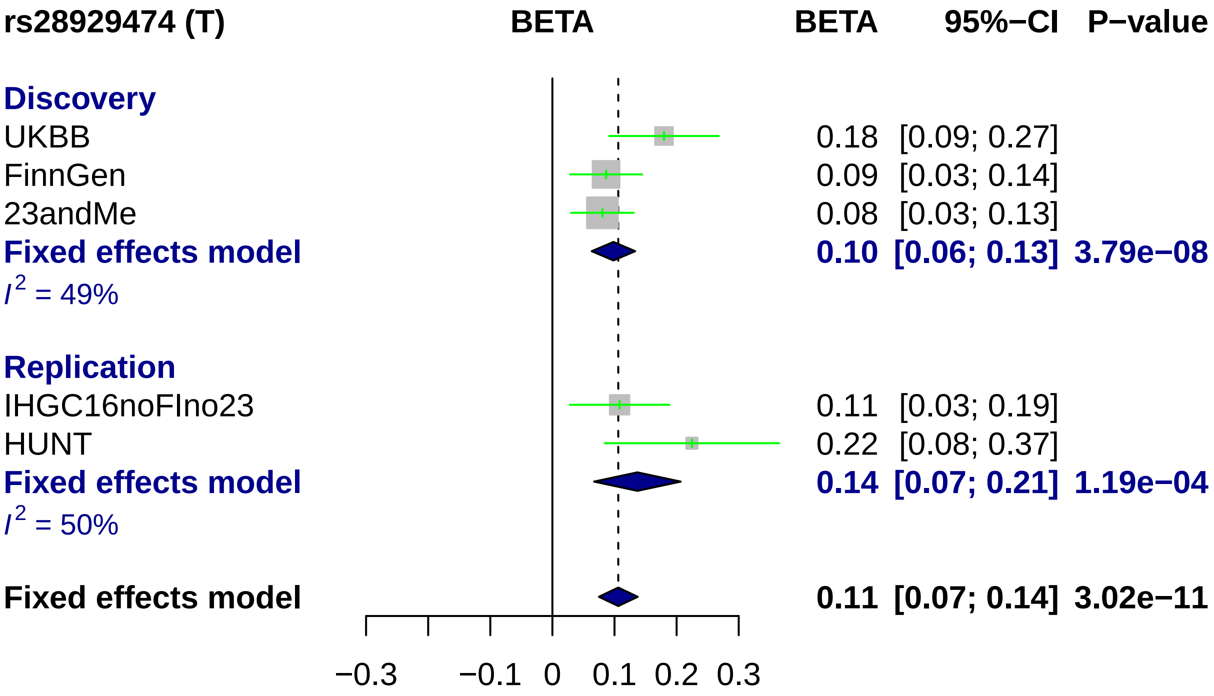
rs28929474 (T)
BETA
BETA
95%−CI
P−value
Discovery
UKBB
0.18 [0.09; 0.27]
FinnGen
0.09 [0.03; 0.14]
23andMe
0.08 [0.03; 0.13]
Fixed effects model
0.10 [0.06; 0.13] 3.79e−08
I^2 = 49%
Replication
IHGC16noFIno23
0.11 [0.03; 0.19]
HUNT
0.22 [0.08; 0.37]
Fixed effects model
0.14 [0.07; 0.21] 1.19e−04
I^2 = 50%
Fixed effects model
0.11 [0.07; 0.14] 3.02e−11
−0.3
−0.1
0
0.1
0.2
0.3

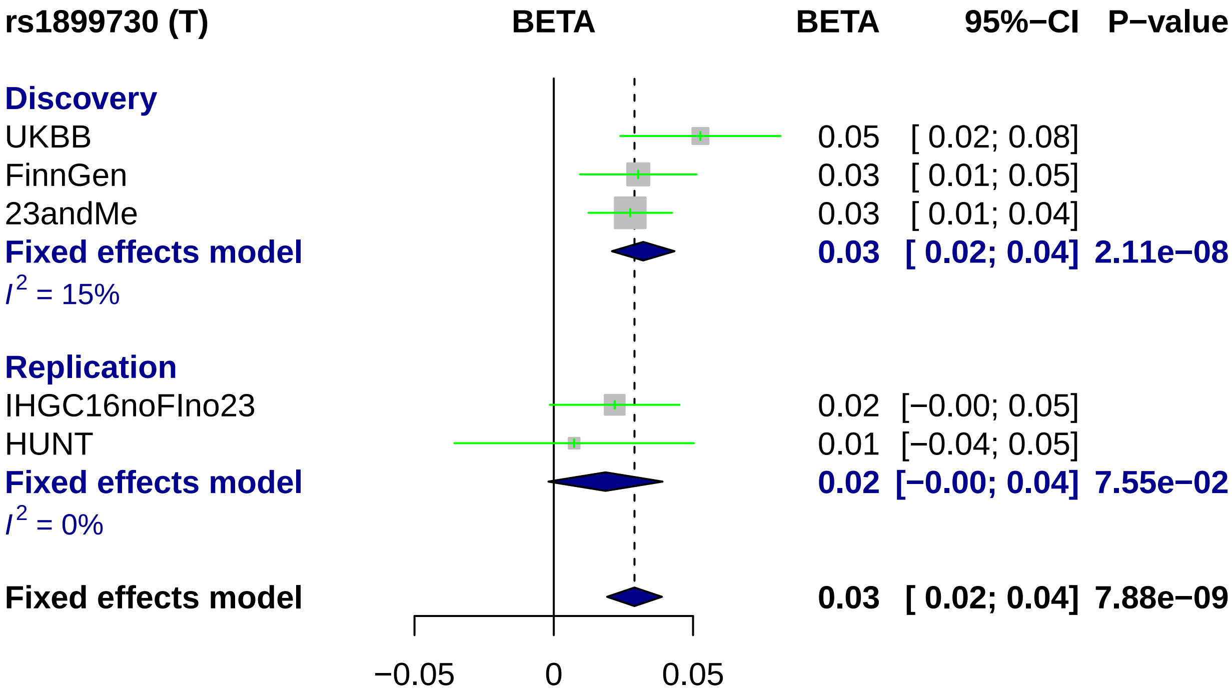
rs1899730 (T)
BETA
BETA
95%−CI
P−value
Discovery
UKBB
0.05 [0.02; 0.08]
FinnGen
0.03 [0.01; 0.05]
23andMe
0.03 [0.01; 0.04]
Fixed effects model
0.03 [0.02; 0.04] 2.11e−08
I2 = 15%
Replication
IHGC16noFIno23
0.02 [−0.00; 0.05]
HUNT
0.01 [−0.04; 0.05]
Fixed effects model
0.02 [−0.00; 0.04] 7.55e−02
I2 = 0%
Fixed effects model
0.03 [0.02; 0.04] 7.88e−09
−0.05
0
0.05

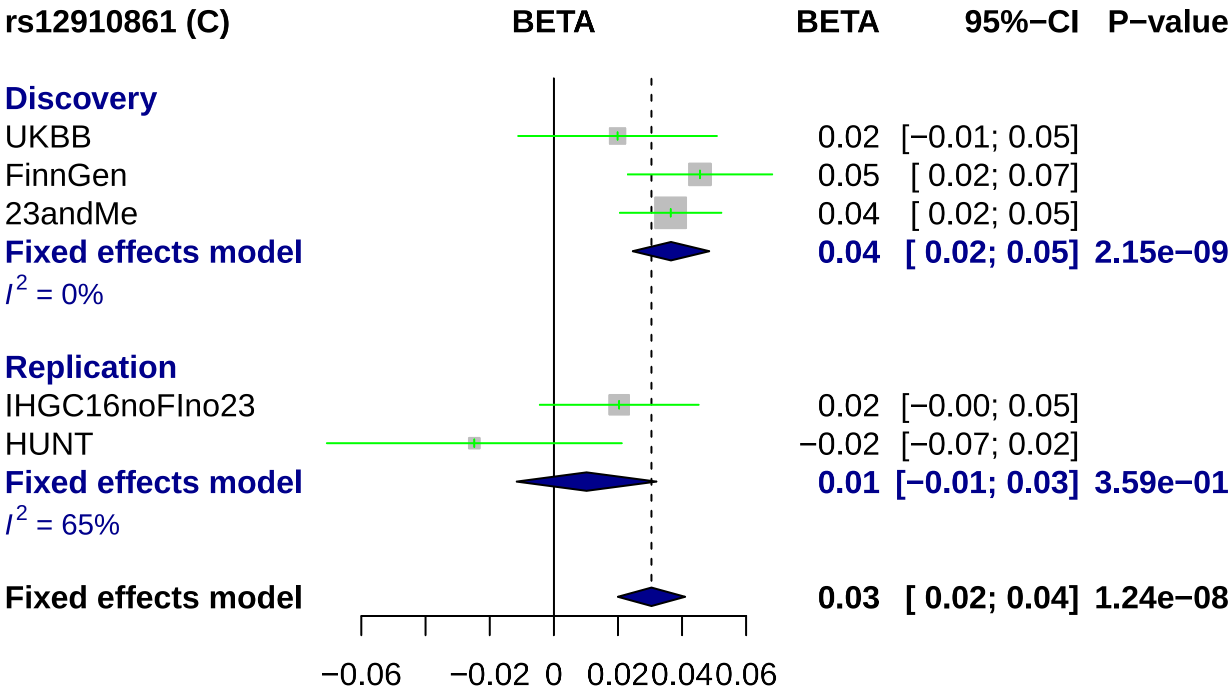
rs12910861 (C)
BETA
BETA
95%−CI
P−value
Discovery
UKBB
0.02 [−0.01; 0.05]
FinnGen
0.05 [0.02; 0.07]
23andMe
0.04 [0.02; 0.05]
Fixed effects model
0.04 [0.02; 0.05] 2.15e−09
I2 = 0%
Replication
IHGC16noFIno23
0.02 [−0.00; 0.05]
HUNT
−0.02 [−0.07; 0.02]
Fixed effects model
0.01 [−0.01; 0.03] 3.59e−01
I2 = 65%
Fixed effects model
0.03 [0.02; 0.04] 1.24e−08
−0.06
−0.02
0
0.02
0.04
0.06

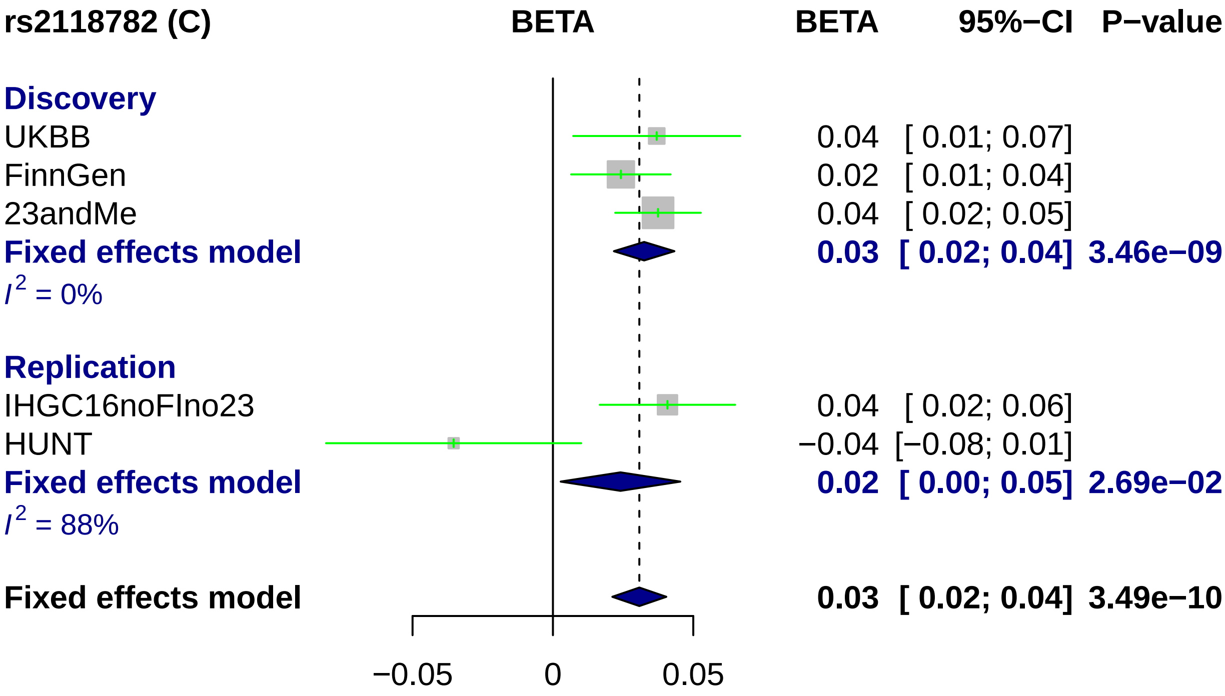
rs2118782 (C)
BETA
BETA
95%−CI
P−value
Discovery
UKBB
0.04 [0.01; 0.07]
FinnGen
0.02 [0.01; 0.04]
23andMe
0.04 [0.02; 0.05]
Fixed effects model
0.03 [0.02; 0.04] 3.46e−09
I2 = 0%
Replication
IHGC16noFIno23
0.04 [0.02; 0.06]
HUNT
−0.04 [−0.08; 0.01]
Fixed effects model
0.02 [0.00; 0.05] 2.69e−02
I2 = 88%
Fixed effects model
0.03 [0.02; 0.04] 3.49e−10
−0.05
0
0.05

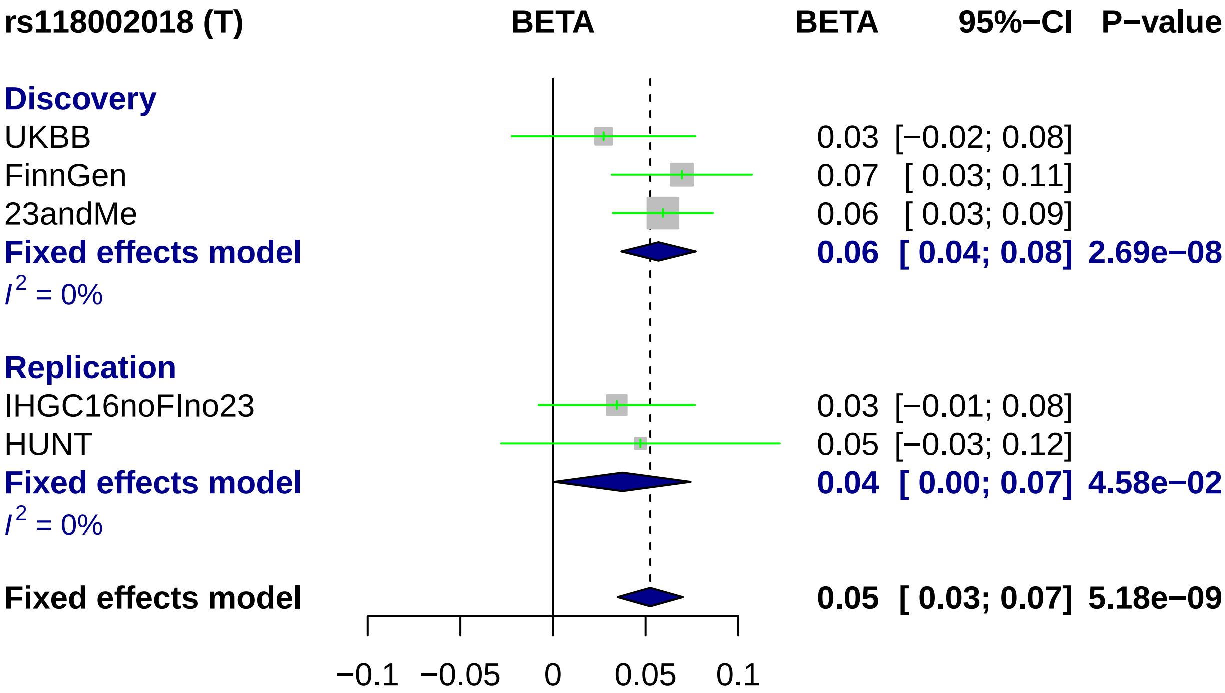
rs118002018 (T)
BETA
BETA 95%−CI P−value
Discovery
UKBB 0.03 [−0.02; 0.08]
FinnGen 0.07 [0.03; 0.11]
23andMe 0.06 [0.03; 0.09]
Fixed effects model 0.06 [0.04; 0.08] 2.69e−08
I 2 = 0%
Replication
IHGC16noFIno23 0.03 [−0.01; 0.08]
HUNT 0.05 [−0.03; 0.12]
Fixed effects model 0.04 [0.00; 0.07] 4.58e−02
I 2 = 0%
Fixed effects model 0.05 [0.03; 0.07] 5.18e−09
−0.1 −0.05 0 0.05 0.1

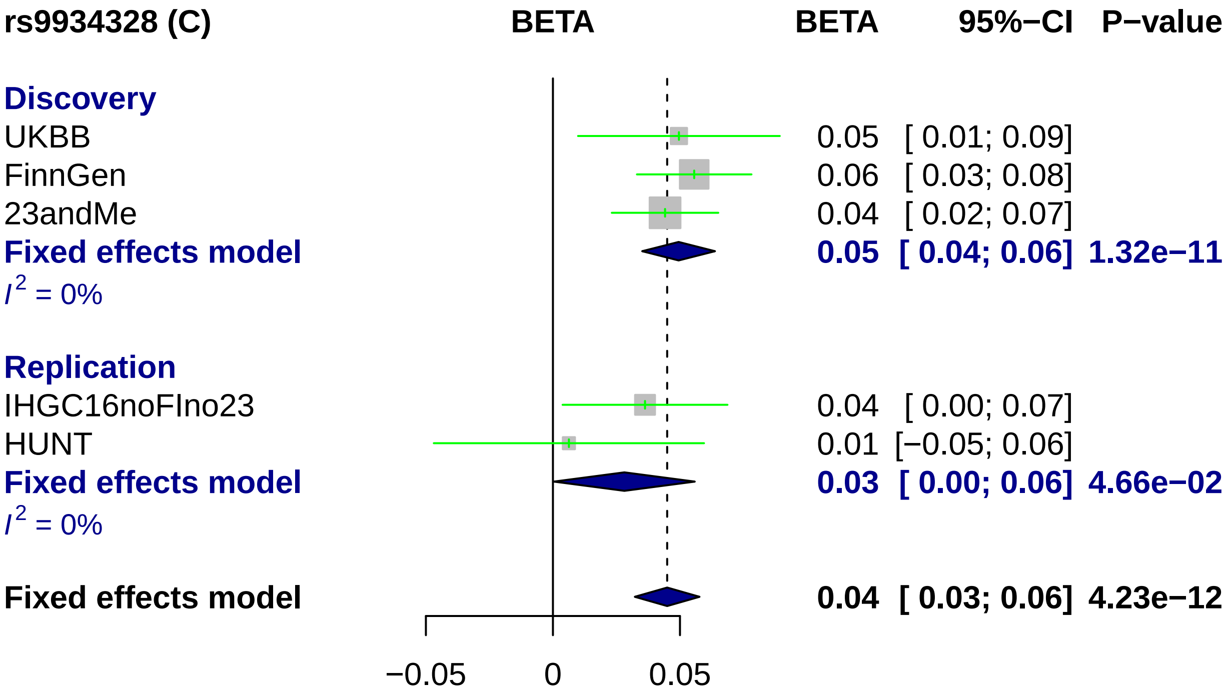
rs9934328 (C)
BETA
BETA
95%−CI
P−value
Discovery
UKBB
0.05 [0.01; 0.09]
FinnGen
0.06 [0.03; 0.08]
23andMe
0.04 [0.02; 0.07]
Fixed effects model
0.05 [0.04; 0.06] 1.32e−11
I2 = 0%
Replication
IHGC16noFIno23
0.04 [0.00; 0.07]
HUNT
0.01 [−0.05; 0.06]
Fixed effects model
0.03 [0.00; 0.06] 4.66e−02
I2 = 0%
Fixed effects model
0.04 [0.03; 0.06] 4.23e−12
−0.05
0
0.05

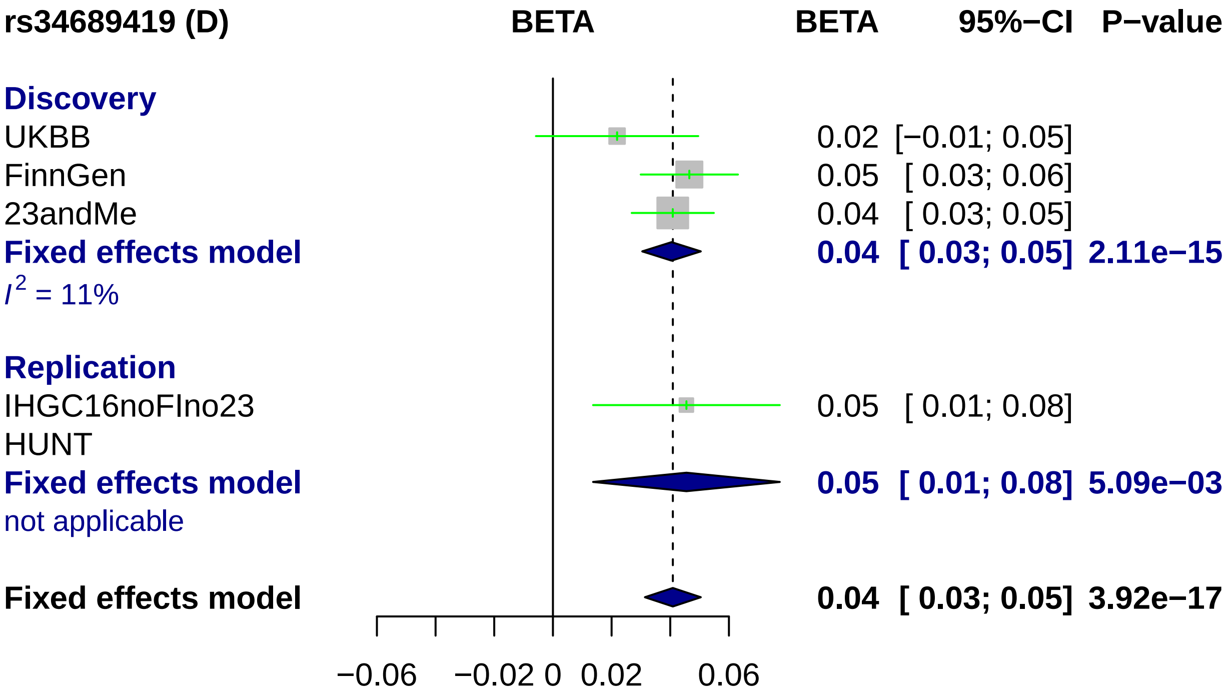
rs34689419 (D)
BETA
BETA
95%−CI
P−value
Discovery
UKBB
0.02 [−0.01; 0.05]
FinnGen
0.05 [0.03; 0.06]
23andMe
0.04 [0.03; 0.05]
Fixed effects model
0.04 [0.03; 0.05] 2.11e−15
I2 = 11%
Replication
IHGC16noFIno23
0.05 [0.01; 0.08]
HUNT
Fixed effects model
0.05 [0.01; 0.08] 5.09e−03
not applicable
Fixed effects model
0.04 [0.03; 0.05] 3.92e−17
−0.06
−0.02
0
0.02
0.06

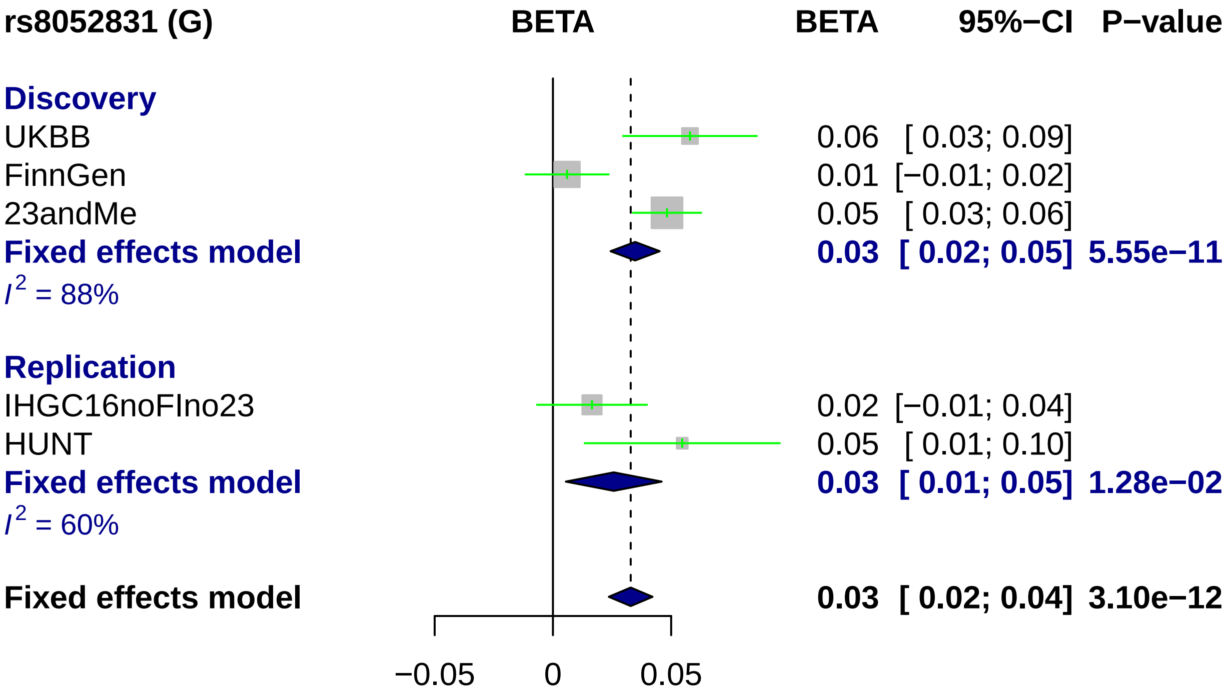
rs8052831 (G)
BETA
BETA
95%−CI
P−value
Discovery
UKBB
0.06
[0.03; 0.09]
FinnGen
0.01
[−0.01; 0.02]
23andMe
0.05
[0.03; 0.06]
Fixed effects model
0.03
[0.02; 0.05]
5.55e−11
I2 = 88%
Replication
IHGC16noFIno23
0.02
[−0.01; 0.04]
HUNT
0.05
[0.01; 0.10]
Fixed effects model
0.03
[0.01; 0.05]
1.28e−02
I2 = 60%
Fixed effects model
0.03
[0.02; 0.04]
3.10e−12
−0.05
0
0.05

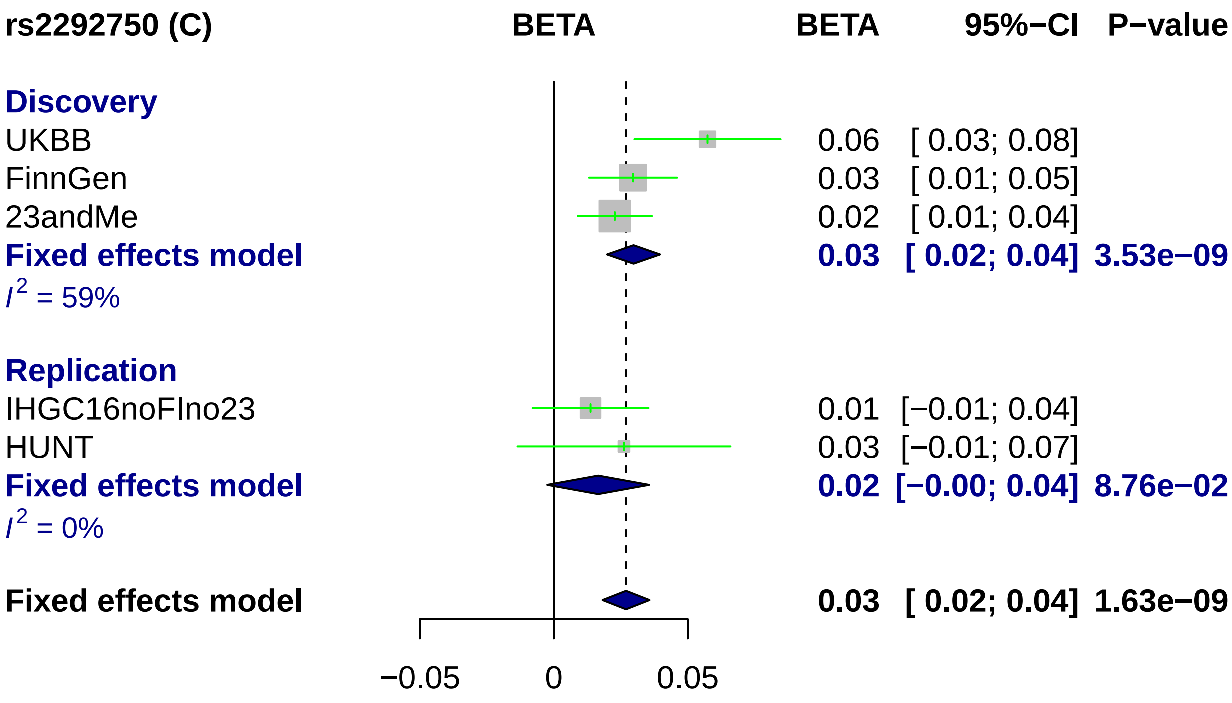
rs2292750 (C)
BETA
BETA
95%−CI
P−value
Discovery
UKBB
0.06
[0.03; 0.08]
FinnGen
0.03
[0.01; 0.05]
23andMe
0.02
[0.01; 0.04]
Fixed effects model
0.03
[0.02; 0.04]
3.53e−09
I2 = 59%
Replication
IHGC16noFIno23
0.01
[−0.01; 0.04]
HUNT
0.03
[−0.01; 0.07]
Fixed effects model
0.02
[−0.00; 0.04]
8.76e−02
I2 = 0%
Fixed effects model
0.03
[0.02; 0.04]
1.63e−09
−0.05
0
0.05

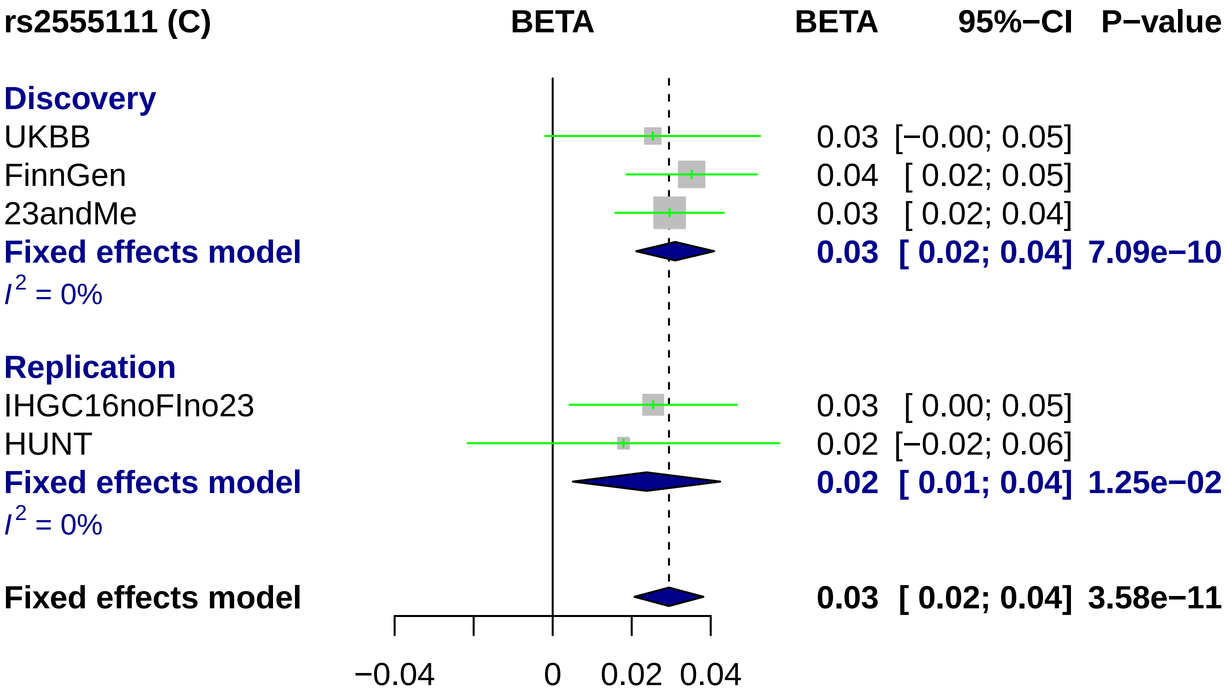
rs2555111 (C)
BETA
BETA
95%−CI
P−value
Discovery
UKBB
0.03 [−0.00; 0.05]
FinnGen
0.04 [0.02; 0.05]
23andMe
0.03 [0.02; 0.04]
Fixed effects model
0.03 [0.02; 0.04] 7.09e−10
I^2 = 0%
Replication
IHGC16noFIno23
0.03 [0.00; 0.05]
HUNT
0.02 [−0.02; 0.06]
Fixed effects model
0.02 [0.01; 0.04] 1.25e−02
I^2 = 0%
Fixed effects model
0.03 [0.02; 0.04] 3.58e−11
−0.04
0
0.02
0.04

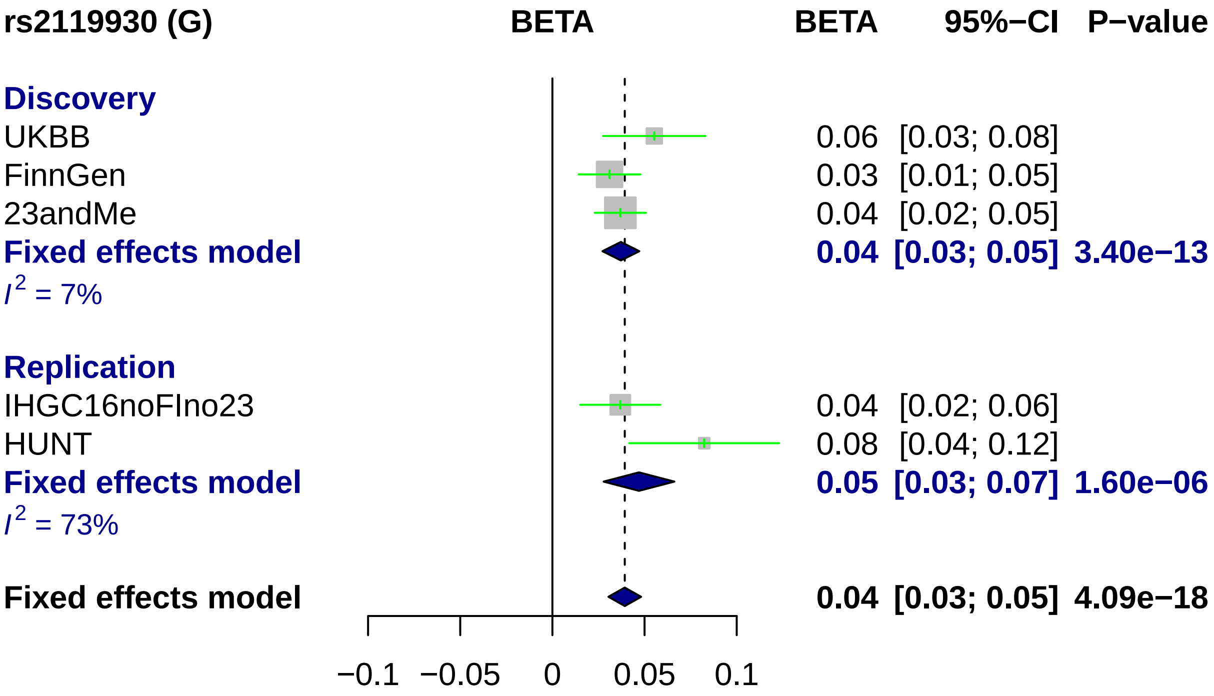
rs2119930 (G)
BETA
BETA
95%−CI
P−value
Discovery
UKBB
0.06 [0.03; 0.08]
FinnGen
0.03 [0.01; 0.05]
23andMe
0.04 [0.02; 0.05]
Fixed effects model
0.04 [0.03; 0.05] 3.40e−13
I2 = 7%
Replication
IHGC16noFIno23
0.04 [0.02; 0.06]
HUNT
0.08 [0.04; 0.12]
Fixed effects model
0.05 [0.03; 0.07] 1.60e−06
I2 = 73%
Fixed effects model
0.04 [0.03; 0.05] 4.09e−18
−0.1
−0.05
0
0.05
0.1

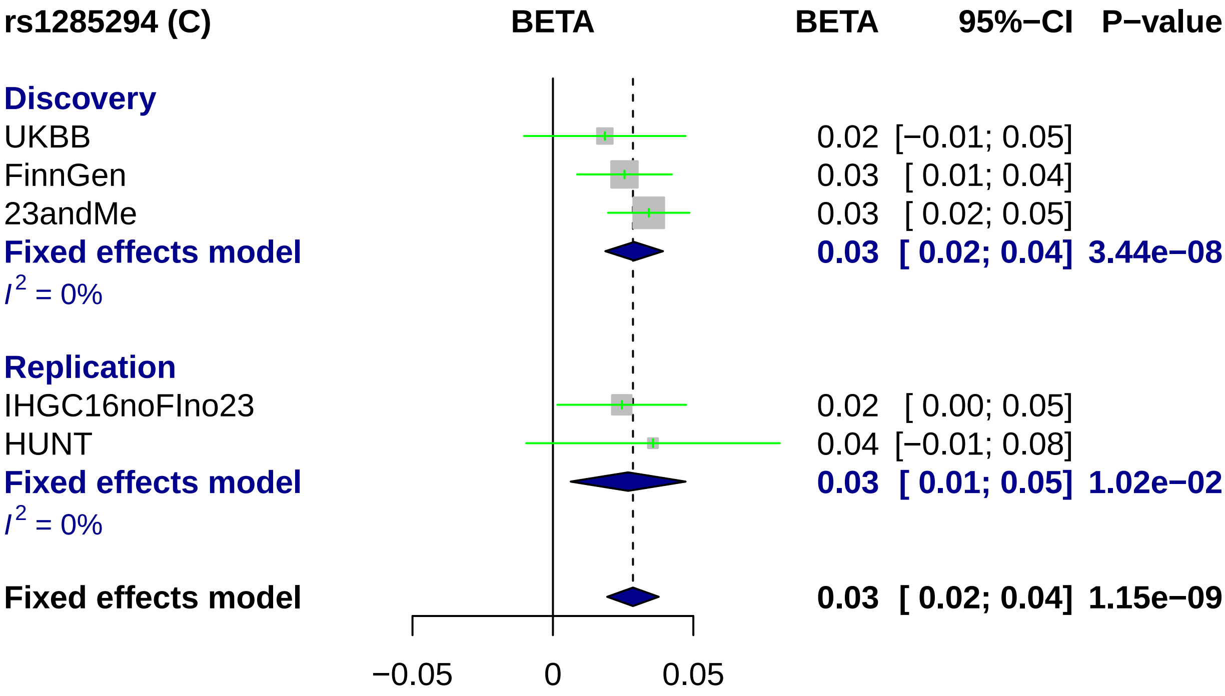
rs1285294 (C)
BETA
BETA
95%−CI
P−value
Discovery
UKBB
0.02 [−0.01; 0.05]
FinnGen
0.03 [0.01; 0.04]
23andMe
0.03 [0.02; 0.05]
Fixed effects model
0.03 [0.02; 0.04] 3.44e−08
I^2 = 0%
Replication
IHGC16noFIno23
0.02 [0.00; 0.05]
HUNT
0.04 [−0.01; 0.08]
Fixed effects model
0.03 [0.01; 0.05] 1.02e−02
I^2 = 0%
Fixed effects model
0.03 [0.02; 0.04] 1.15e−09
−0.05
0
0.05

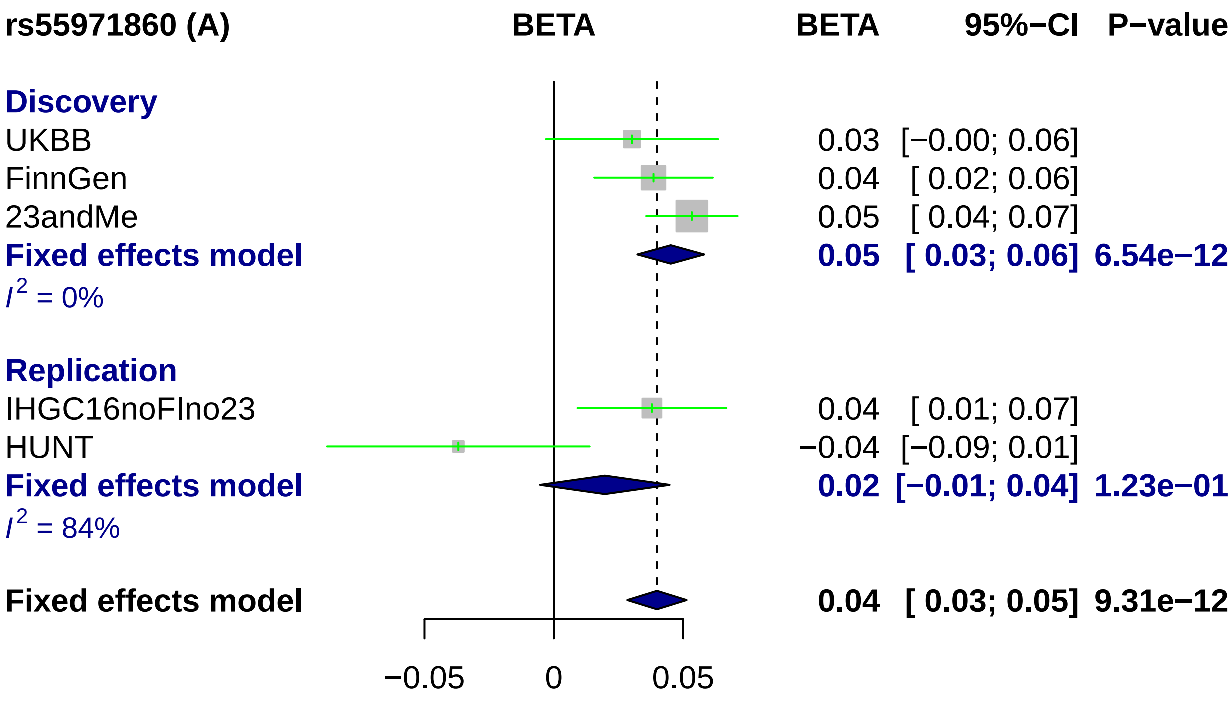

rs55971860 (A)
BETA
BETA
95%−CI
P−value
Discovery
UKBB
0.03 [−0.00; 0.06]
FinnGen
0.04 [0.02; 0.06]
23andMe
0.05 [0.04; 0.07]
Fixed effects model
0.05 [0.03; 0.06] 6.54e−12
I2 = 0%
Replication
IHGC16noFIno23
0.04 [0.01; 0.07]
HUNT
−0.04 [−0.09; 0.01]
Fixed effects model
0.02 [−0.01; 0.04] 1.23e−01
I2 = 84%
Fixed effects model
0.04 [0.03; 0.05] 9.31e−12
−0.05
0
0.05

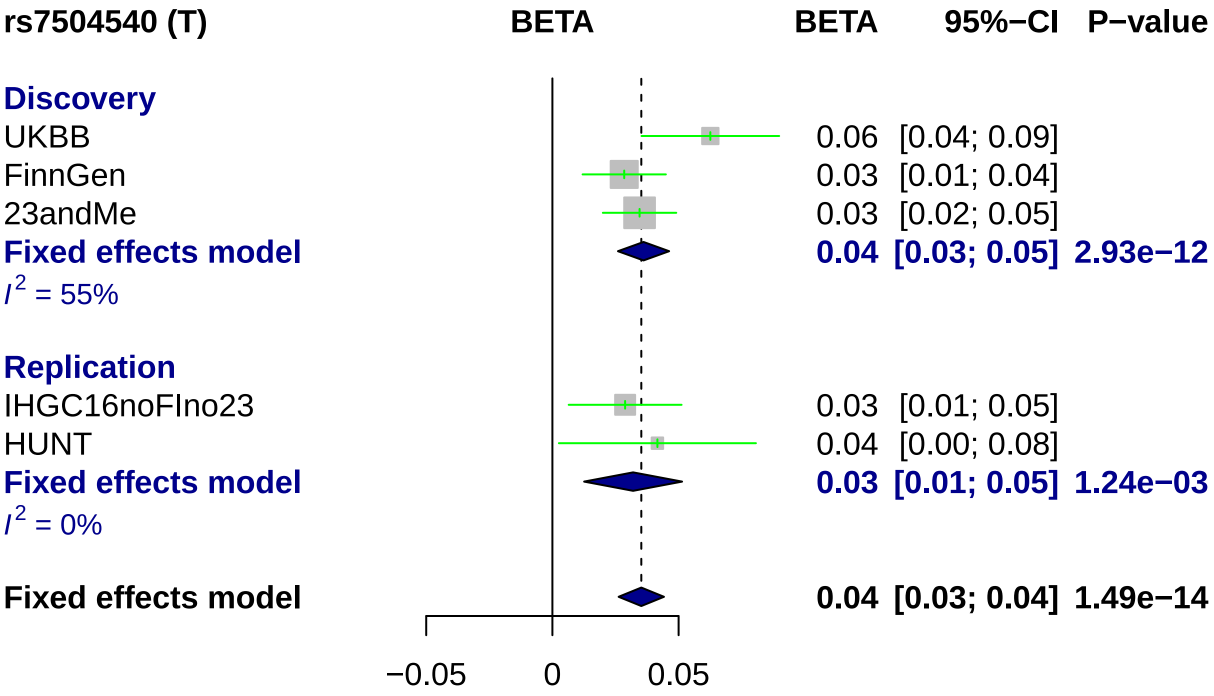
rs7504540 (T)
BETA
BETA
95%−CI
P−value
Discovery
UKBB
0.06 [0.04; 0.09]
FinnGen
0.03 [0.01; 0.04]
23andMe
0.03 [0.02; 0.05]
Fixed effects model
0.04 [0.03; 0.05] 2.93e−12
I2 = 55%
Replication
IHGC16noFIno23
0.03 [0.01; 0.05]
HUNT
0.04 [0.00; 0.08]
Fixed effects model
0.03 [0.01; 0.05] 1.24e−03
I2 = 0%
Fixed effects model
0.04 [0.03; 0.04] 1.49e−14
−0.05
0
0.05

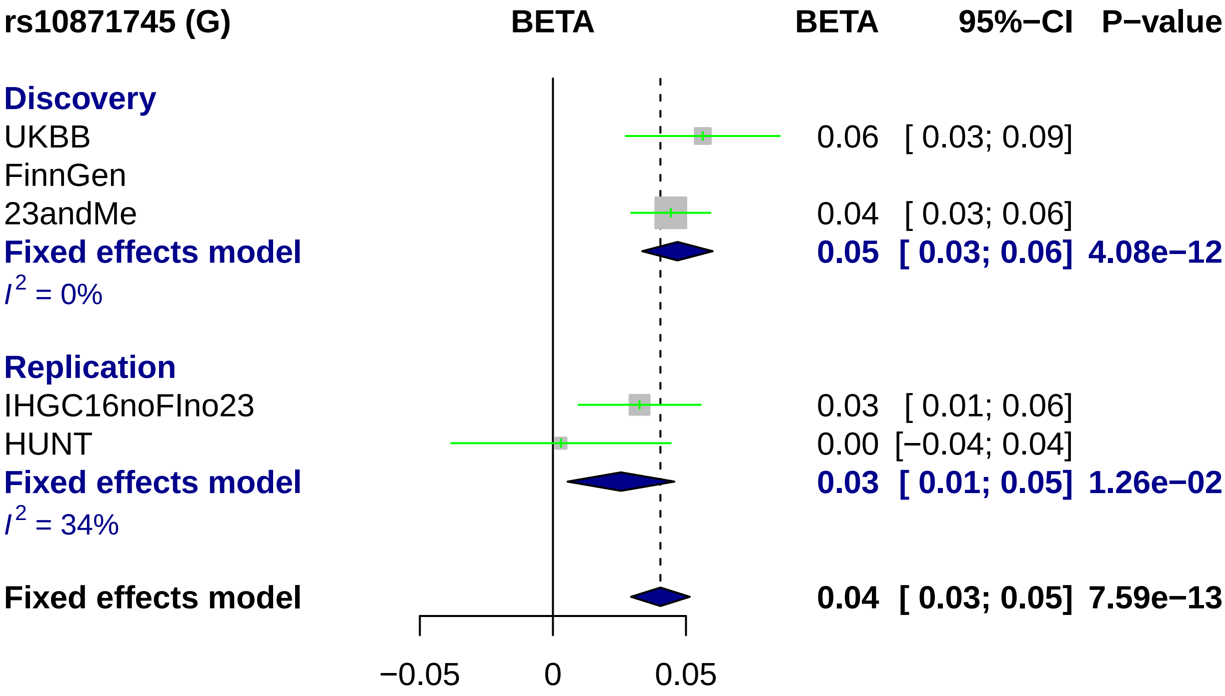
rs10871745 (G)
BETA
BETA
95%−CI
P−value
Discovery
UKBB
0.06 [0.03; 0.09]
FinnGen
23andMe
0.04 [0.03; 0.06]
Fixed effects model
0.05 [0.03; 0.06] 4.08e−12
I^2 = 0%
Replication
IHGC16noFIno23
0.03 [0.01; 0.06]
HUNT
0.00 [−0.04; 0.04]
Fixed effects model
0.03 [0.01; 0.05] 1.26e−02
I^2 = 34%
Fixed effects model
0.04 [0.03; 0.05] 7.59e−13
−0.05
0
0.05

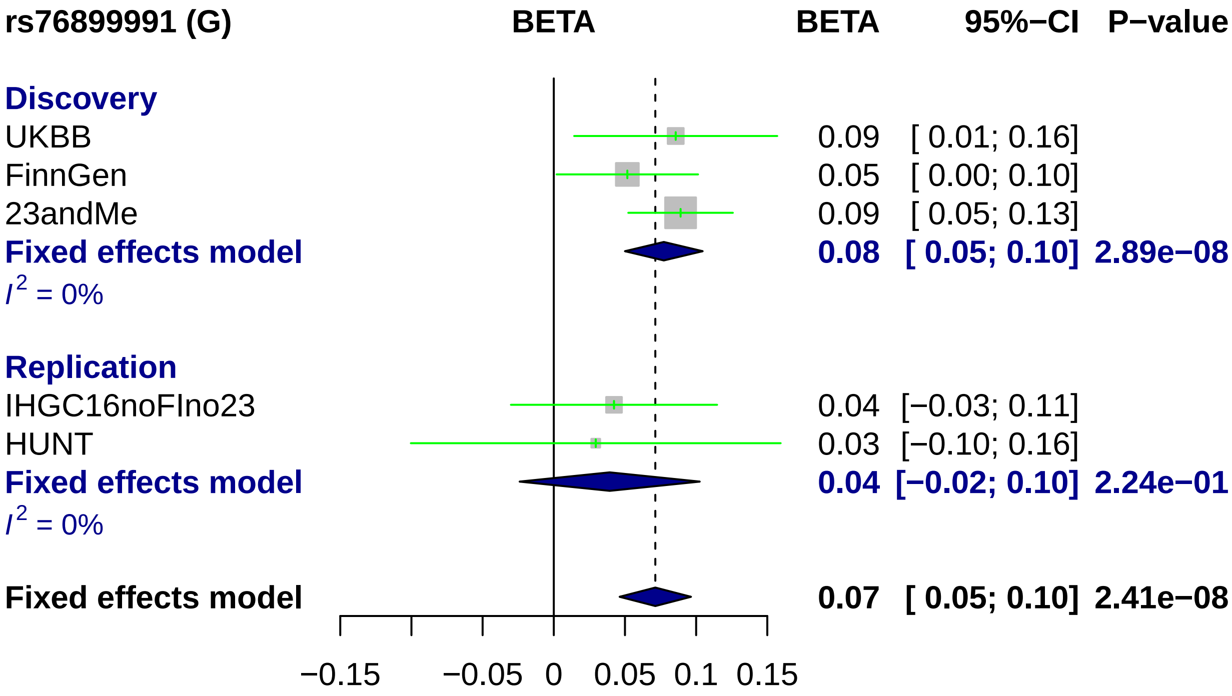

rs76899991 (G)
BETA
BETA
95%−CI
P−value
Discovery
UKBB
0.09
[0.01; 0.16]
FinnGen
0.05
[0.00; 0.10]
23andMe
0.09
[0.05; 0.13]
Fixed effects model
0.08
[0.05; 0.10]
2.89e−08
I2 = 0%
Replication
IHGC16noFIno23
0.04
[−0.03; 0.11]
HUNT
0.03
[−0.10; 0.16]
Fixed effects model
0.04
[−0.02; 0.10]
2.24e−01
I2 = 0%
Fixed effects model
0.07
[0.05; 0.10]
2.41e−08
−0.15
−0.05
0
0.05
0.1
0.15

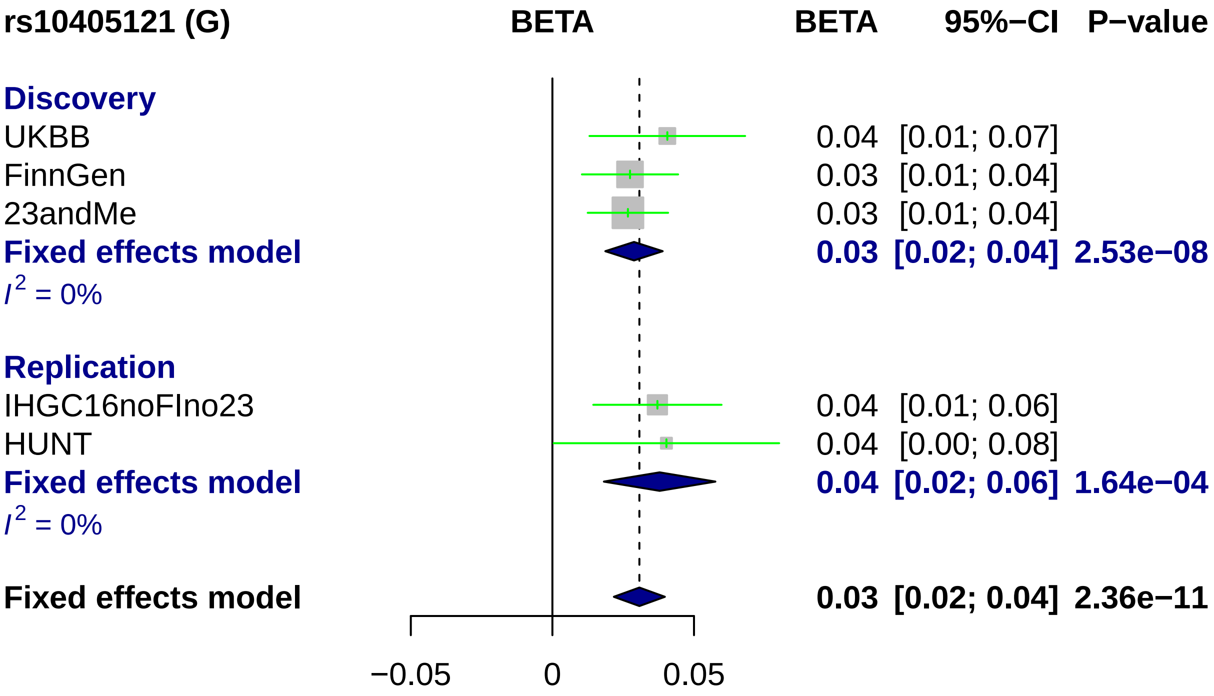

rs10405121 (G)
BETA
BETA
95%−CI
P−value
Discovery
UKBB
0.04 [0.01; 0.07]
FinnGen
0.03 [0.01; 0.04]
23andMe
0.03 [0.01; 0.04]
Fixed effects model
0.03 [0.02; 0.04] 2.53e−08
I2 = 0%
Replication
IHGC16noFIno23
0.04 [0.01; 0.06]
HUNT
0.04 [0.00; 0.08]
Fixed effects model
0.04 [0.02; 0.06] 1.64e−04
I2 = 0%
Fixed effects model
0.03 [0.02; 0.04] 2.36e−11
−0.05
0
0.05

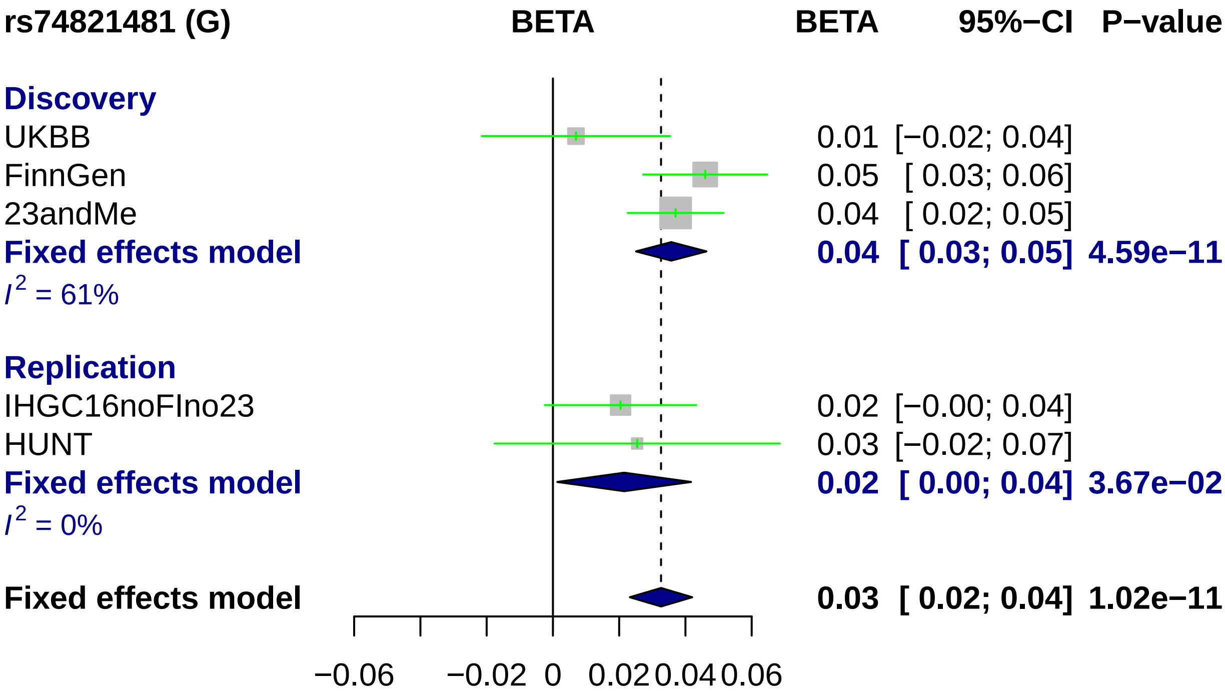
rs74821481 (G)
BETA
BETA
95%−CI
P−value
Discovery
UKBB
FinnGen
23andMe
Fixed effects model
I² = 61%
Replication
IHGC16noFIno23
HUNT
Fixed effects model
I² = 0%
Fixed effects model
0.01 [−0.02; 0.04]
0.05 [0.03; 0.06]
0.04 [0.02; 0.05]
0.04 [0.03; 0.05] 4.59e−11
0.02 [−0.00; 0.04]
0.03 [−0.02; 0.07]
0.02 [0.00; 0.04] 3.67e−02
0.03 [0.02; 0.04] 1.02e−11
−0.06
−0.02
0
0.02
0.04
0.06

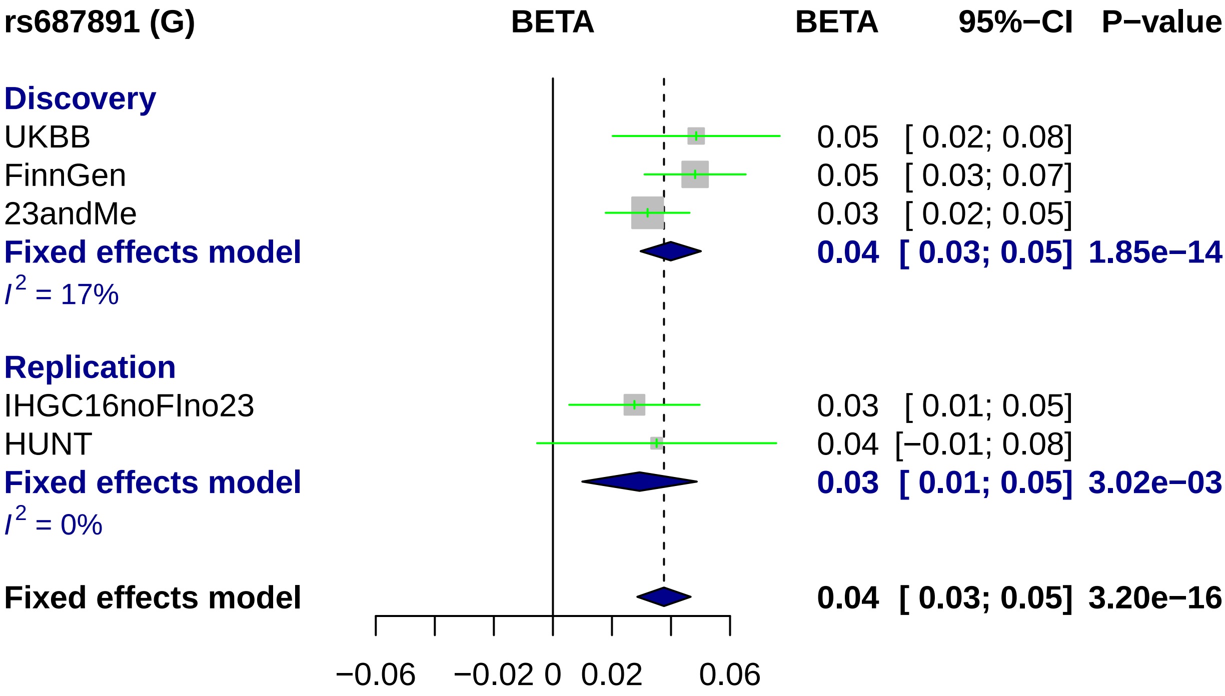
rs687891 (G)
BETA
BETA
95%−CI
P−value
Discovery
UKBB
0.05 [0.02; 0.08]
FinnGen
0.05 [0.03; 0.07]
23andMe
0.03 [0.02; 0.05]
Fixed effects model
0.04 [0.03; 0.05] 1.85e−14
I^2 = 17%
Replication
IHGC16noFIno23
0.03 [0.01; 0.05]
HUNT
0.04 [−0.01; 0.08]
Fixed effects model
0.03 [0.01; 0.05] 3.02e−03
I^2 = 0%
Fixed effects model
0.04 [0.03; 0.05] 3.20e−16
−0.06
−0.02
0
0.02
0.06

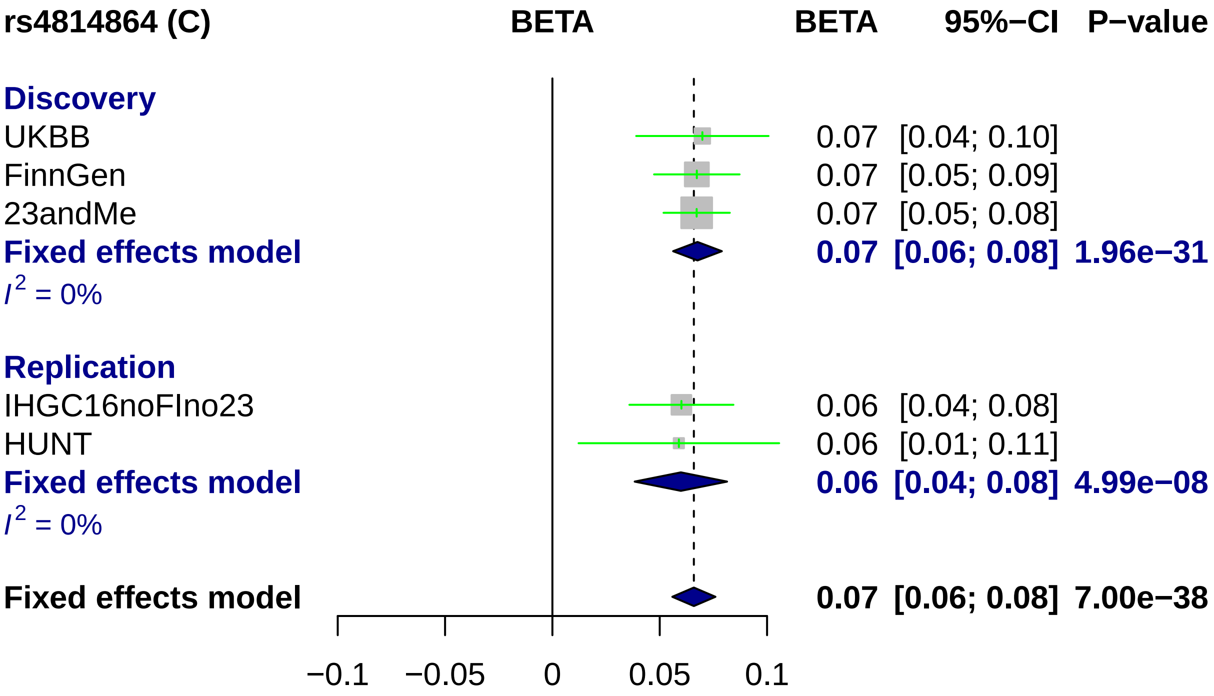

rs4814864 (C)
BETA
BETA
95%−CI
P−value
Discovery
UKBB
0.07 [0.04; 0.10]
FinnGen
0.07 [0.05; 0.09]
23andMe
0.07 [0.05; 0.08]
Fixed effects model
0.07 [0.06; 0.08] 1.96e−31
I^2 = 0%
Replication
IHGC16noFIno23
0.06 [0.04; 0.08]
HUNT
0.06 [0.01; 0.11]
Fixed effects model
0.06 [0.04; 0.08] 4.99e−08
I^2 = 0%
Fixed effects model
0.07 [0.06; 0.08] 7.00e−38
−0.1
−0.05
0
0.05
0.1

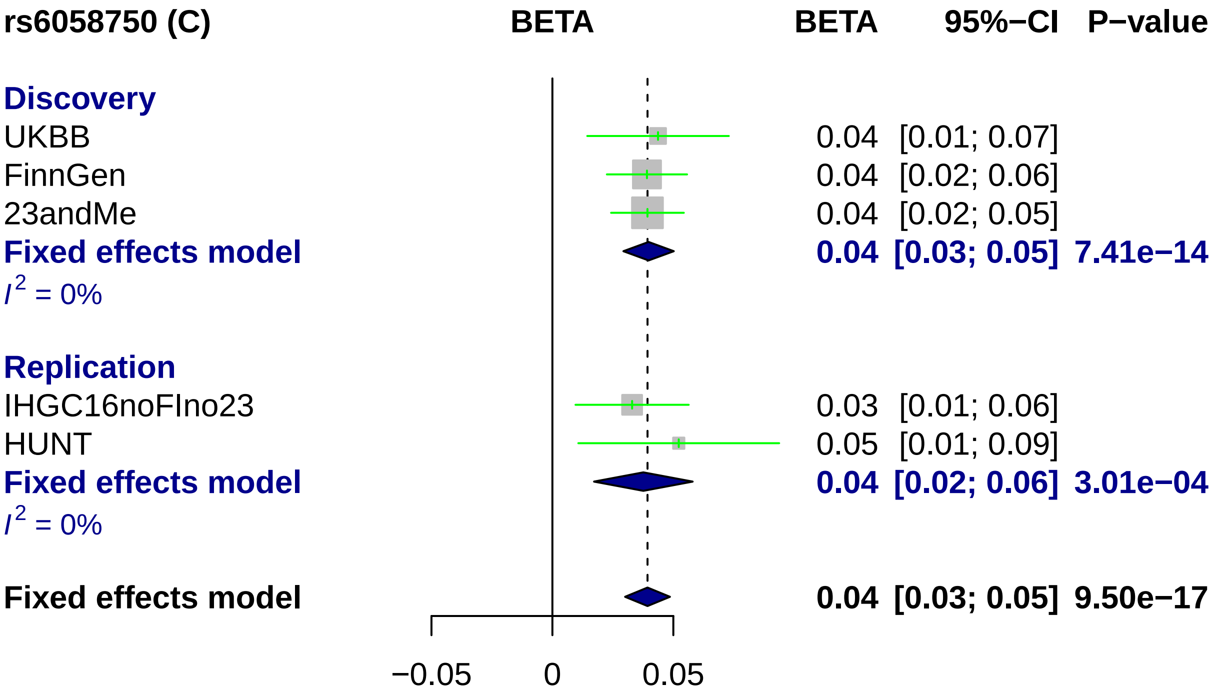
rs6058750 (C)
BETA
BETA
95%−CI
P−value
Discovery
UKBB
0.04 [0.01; 0.07]
FinnGen
0.04 [0.02; 0.06]
23andMe
0.04 [0.02; 0.05]
Fixed effects model
0.04 [0.03; 0.05] 7.41e−14
I2 = 0%
Replication
IHGC16noFIno23
0.03 [0.01; 0.06]
HUNT
0.05 [0.01; 0.09]
Fixed effects model
0.04 [0.02; 0.06] 3.01e−04
I2 = 0%
Fixed effects model
0.04 [0.03; 0.05] 9.50e−17
−0.05
0
0.05

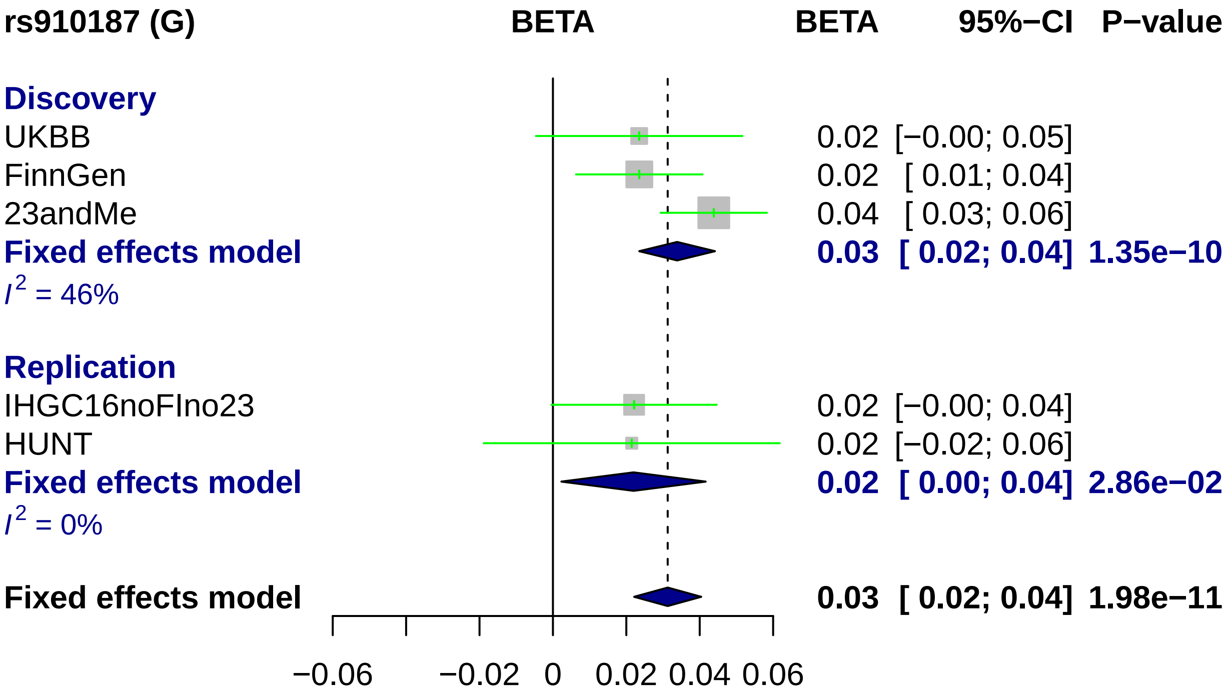
rs910187 (G)
BETA
BETA
95%−CI
P−value
Discovery
UKBB
0.02 [−0.00; 0.05]
FinnGen
0.02 [0.01; 0.04]
23andMe
0.04 [0.03; 0.06]
Fixed effects model
0.03 [0.02; 0.04] 1.35e−10
I2 = 46%
Replication
IHGC16noFIno23
0.02 [−0.00; 0.04]
HUNT
0.02 [−0.02; 0.06]
Fixed effects model
0.02 [0.00; 0.04] 2.86e−02
I2 = 0%
Fixed effects model
0.03 [0.02; 0.04] 1.98e−11
−0.06
−0.02
0
0.02
0.04
0.06

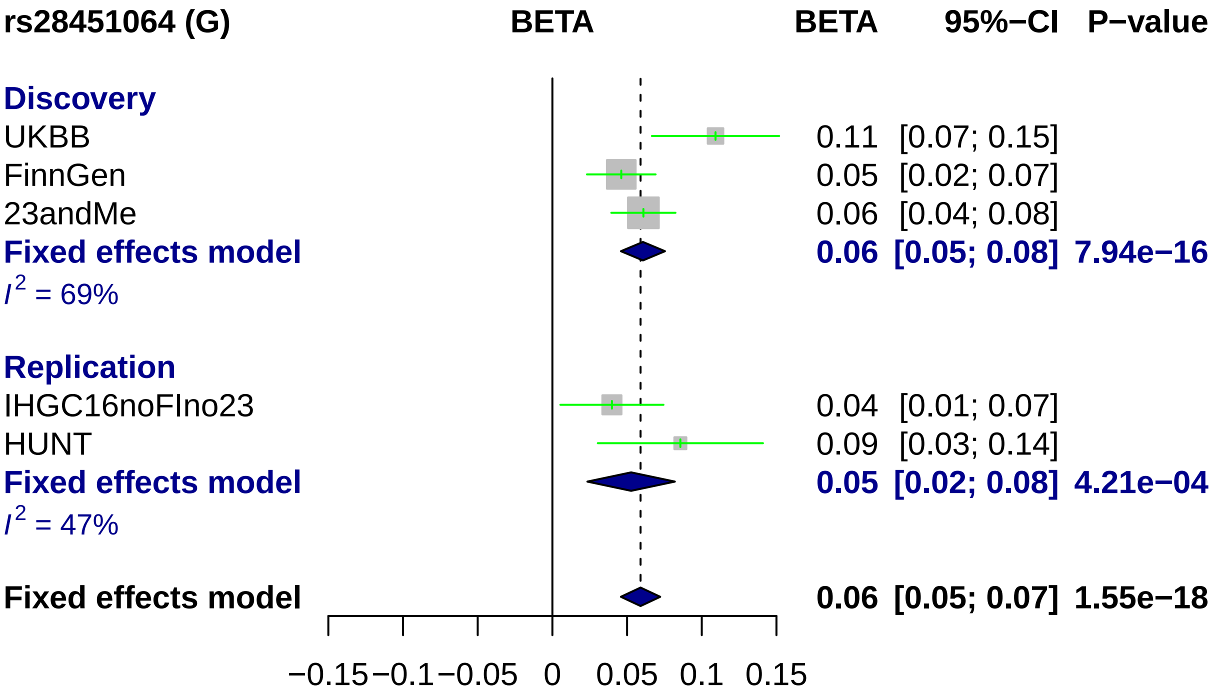
rs28451064 (G)
BETA
BETA
95%−CI
P−value
Discovery
UKBB
0.11 [0.07; 0.15]
FinnGen
0.05 [0.02; 0.07]
23andMe
0.06 [0.04; 0.08]
Fixed effects model
0.06 [0.05; 0.08] 7.94e−16
I^2 = 69%
Replication
IHGC16noFIno23
0.04 [0.01; 0.07]
HUNT
0.09 [0.03; 0.14]
Fixed effects model
0.05 [0.02; 0.08] 4.21e−04
I^2 = 47%
Fixed effects model
0.06 [0.05; 0.07] 1.55e−18
−0.15 −0.1 −0.05 0 0.05 0.1 0.15

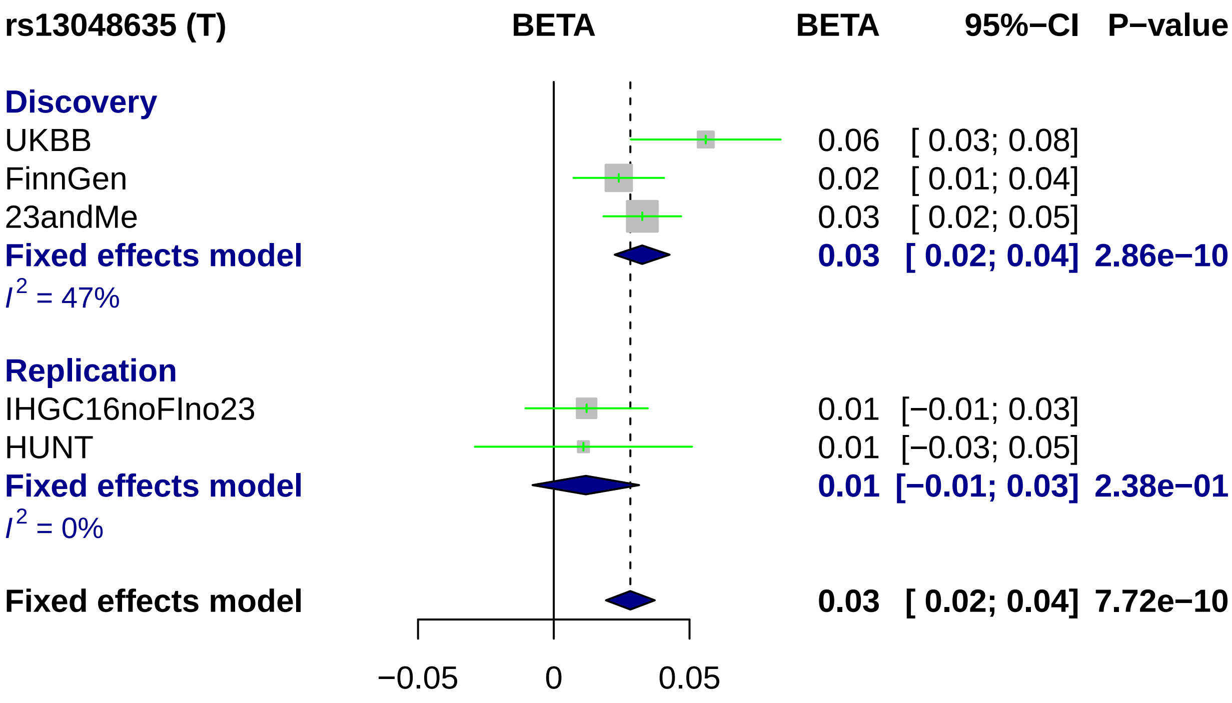

rs13048635 (T)
BETA
BETA
95%−CI
P−value
Discovery
UKBB
0.06 [0.03; 0.08]
FinnGen
0.02 [0.01; 0.04]
23andMe
0.03 [0.02; 0.05]
Fixed effects model
0.03 [0.02; 0.04] 2.86e−10
I^2 = 47%
Replication
IHGC16noFIno23
0.01 [−0.01; 0.03]
HUNT
0.01 [−0.03; 0.05]
Fixed effects model
0.01 [−0.01; 0.03] 2.38e−01
I^2 = 0%
Fixed effects model
0.03 [0.02; 0.04] 7.72e−10
−0.05
0
0.05

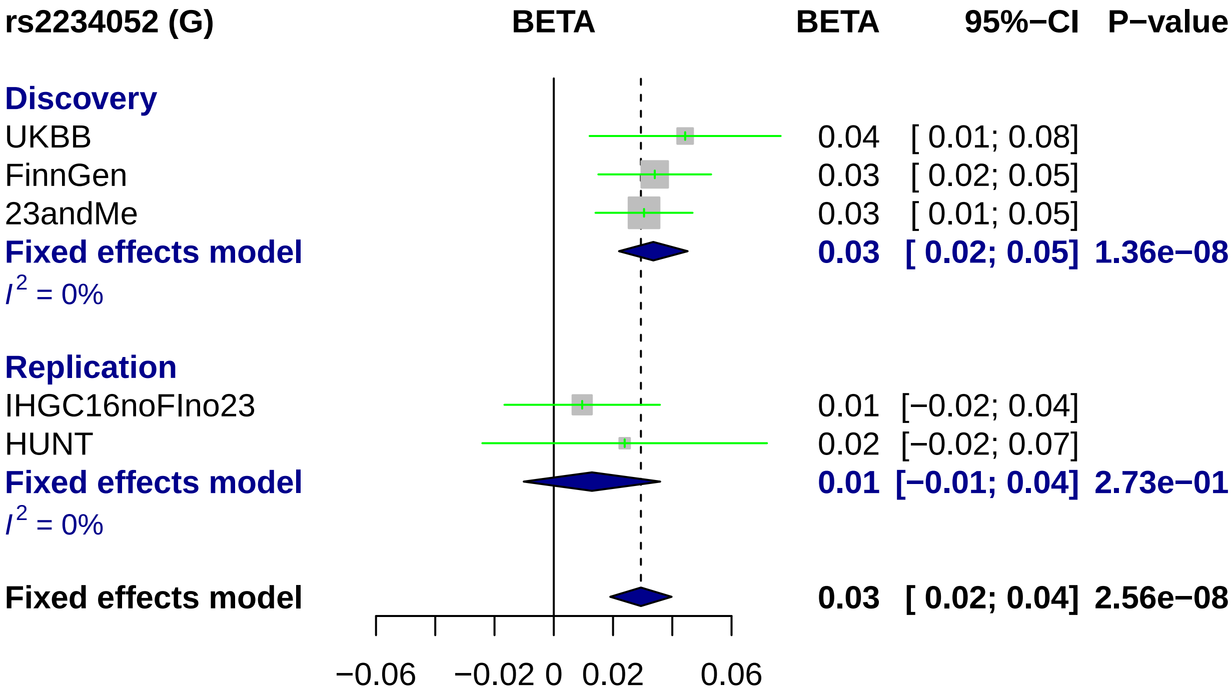

rs2234052 (G)
BETA
BETA
95%−CI
P−value
Discovery
UKBB
0.04
[0.01; 0.08]
FinnGen
0.03
[0.02; 0.05]
23andMe
0.03
[0.01; 0.05]
Fixed effects model
0.03
[0.02; 0.05]
1.36e−08
I2 = 0%
Replication
IHGC16noFIno23
0.01
[−0.02; 0.04]
HUNT
0.02
[−0.02; 0.07]
Fixed effects model
0.01
[−0.01; 0.04]
2.73e−01
I2 = 0%
Fixed effects model
0.03
[0.02; 0.04]
2.56e−08
−0.06
−0.02
0
0.02
0.06

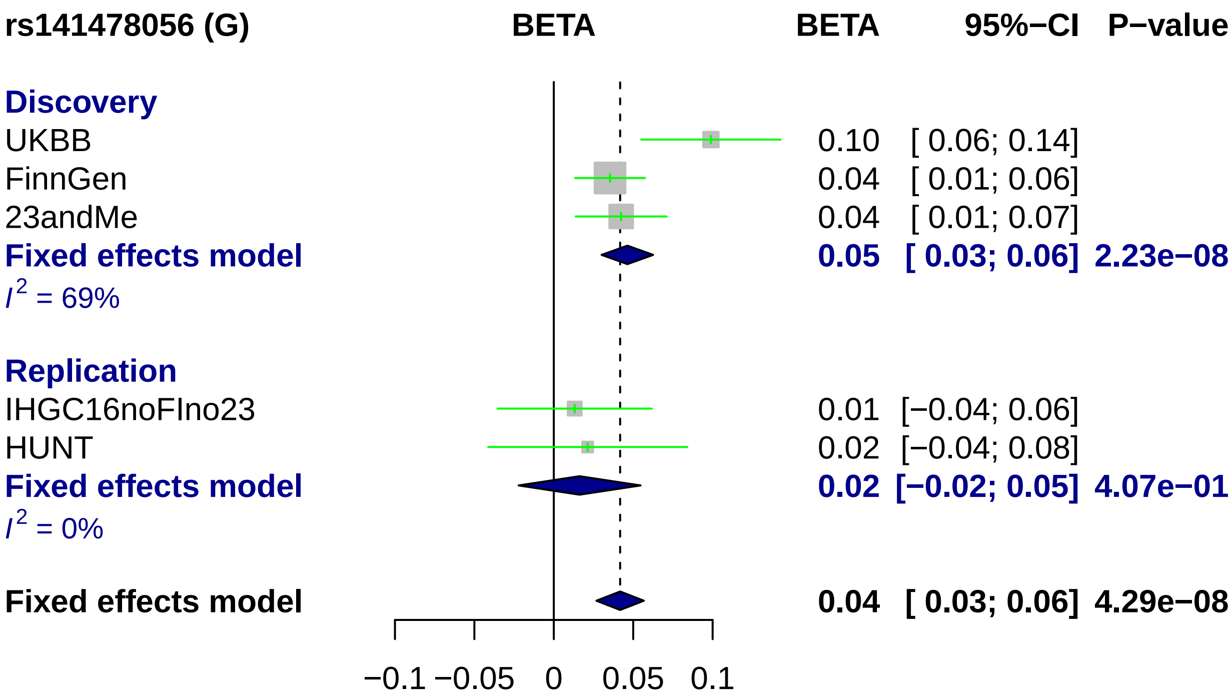
rs141478056 (G)
BETA
BETA
95%−CI
P−value
Discovery
UKBB
0.10
[0.06; 0.14]
FinnGen
0.04
[0.01; 0.06]
23andMe
0.04
[0.01; 0.07]
Fixed effects model
0.05
[0.03; 0.06]
2.23e−08
I2 = 69%
Replication
IHGC16noFIno23
0.01
[−0.04; 0.06]
HUNT
0.02
[−0.04; 0.08]
Fixed effects model
0.02
[−0.02; 0.05]
4.07e−01
I2 = 0%
Fixed effects model
0.04
[0.03; 0.06]
4.29e−08
−0.1
−0.05
0
0.05
0.1

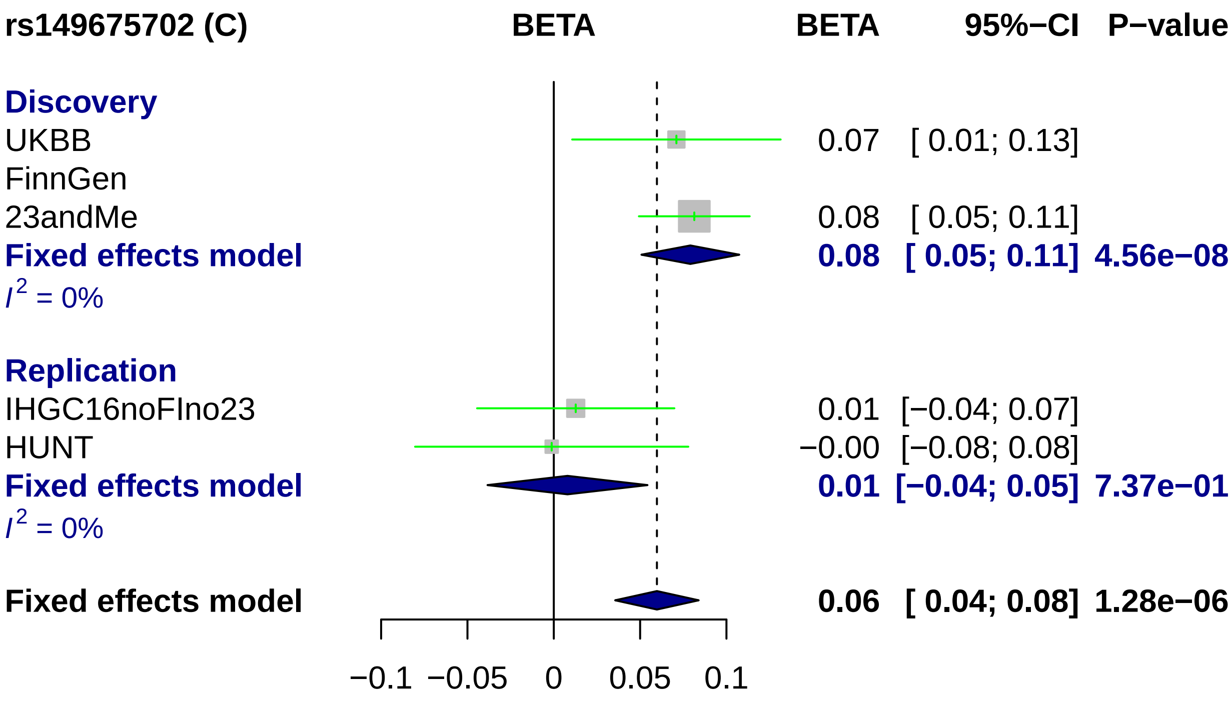
rs149675702 (C)
BETA
BETA
95%−CI
P−value
Discovery
UKBB
0.07 [0.01; 0.13]
FinnGen
23andMe
0.08 [0.05; 0.11]
Fixed effects model
0.08 [0.05; 0.11] 4.56e−08
I2 = 0%
Replication
IHGC16noFIno23
0.01 [−0.04; 0.07]
HUNT
−0.00 [−0.08; 0.08]
Fixed effects model
0.01 [−0.04; 0.05] 7.37e−01
I2 = 0%
Fixed effects model
0.06 [0.04; 0.08] 1.28e−06
−0.1
−0.05
0
0.05
0.1

Supplementary Figure 4. **LocusZoom-plots of the 122 LD-independent migraine risk loci identified from the meta-analysis (N = 967,534; 98,374 cases and 869,160 controls).** X-axis shows the chromosomal location, and Y-axis shows the strength of the association as two-sided $-\log_{10}$ *P*-value from the inverse-variance weighted fixed-effects meta-analysis. Black horizontal line corresponds to $P = 5 \times 10^{-8}$ and blue line shows the recombination rate. The squared correlation to the lead variant is shown by colors based on the combined UK Biobank and FinnGen data. Figure 4 is divided into 122 parts on the following pages.

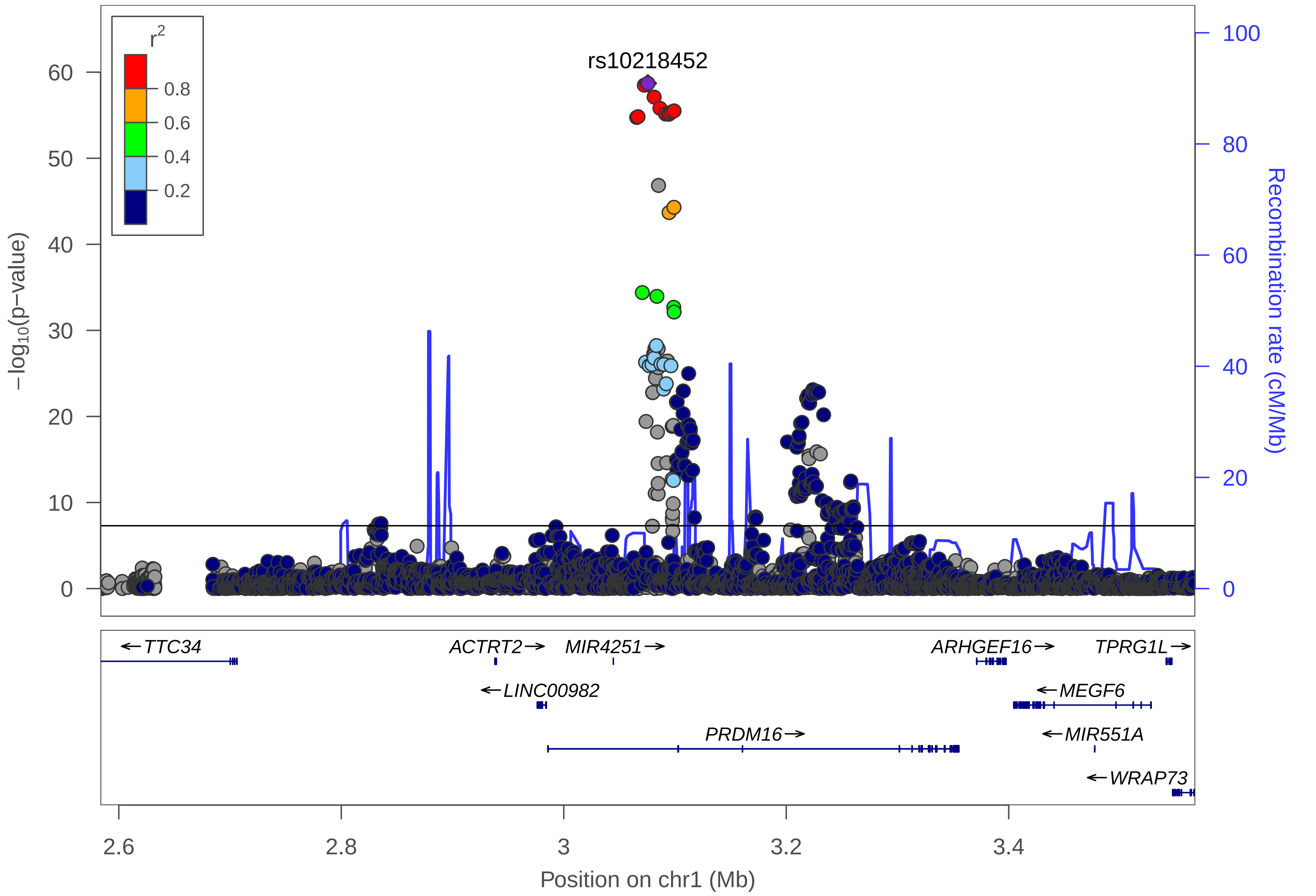

rs10218452
r2
0.8
0.6
0.4
0.2
60
50
40
30
20
10
0
−log10(p−value)
100
80
60
40
20
0
Recombination rate (cM/Mb)
←TTC34
ACTRT2→
MIR4251→
ARHGEF16→
TPRG1L→
←LINC00982
←MEGF6
PRDM16→
←MIR551A
←WRAP73
2.6
2.8
3
3.2
3.4
Position on chr1 (Mb)

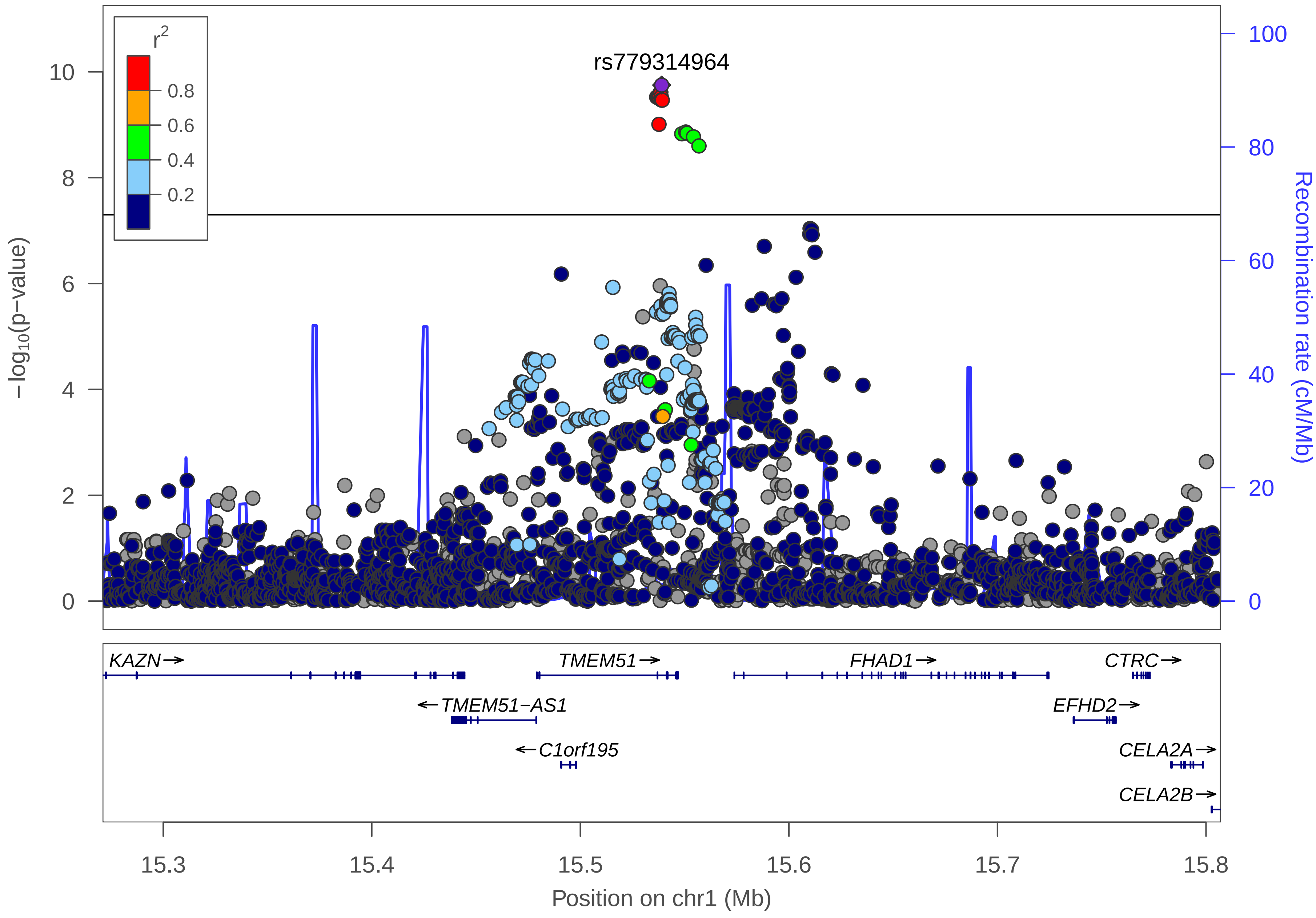

r2
0.8
0.6
0.4
0.2
rs779314964
10
8
6
4
2
0
−log10(p−value)
100
80
60
40
20
0
Recombination rate (cM/Mb)
KAZN→
TMEM51→
FHAD1→
CTRC→
←TMEM51−AS1
EFHD2→
←C1orf195
CELA2A→
CELA2B→
15.3
15.4
15.5
15.6
15.7
15.8
Position on chr1 (Mb)

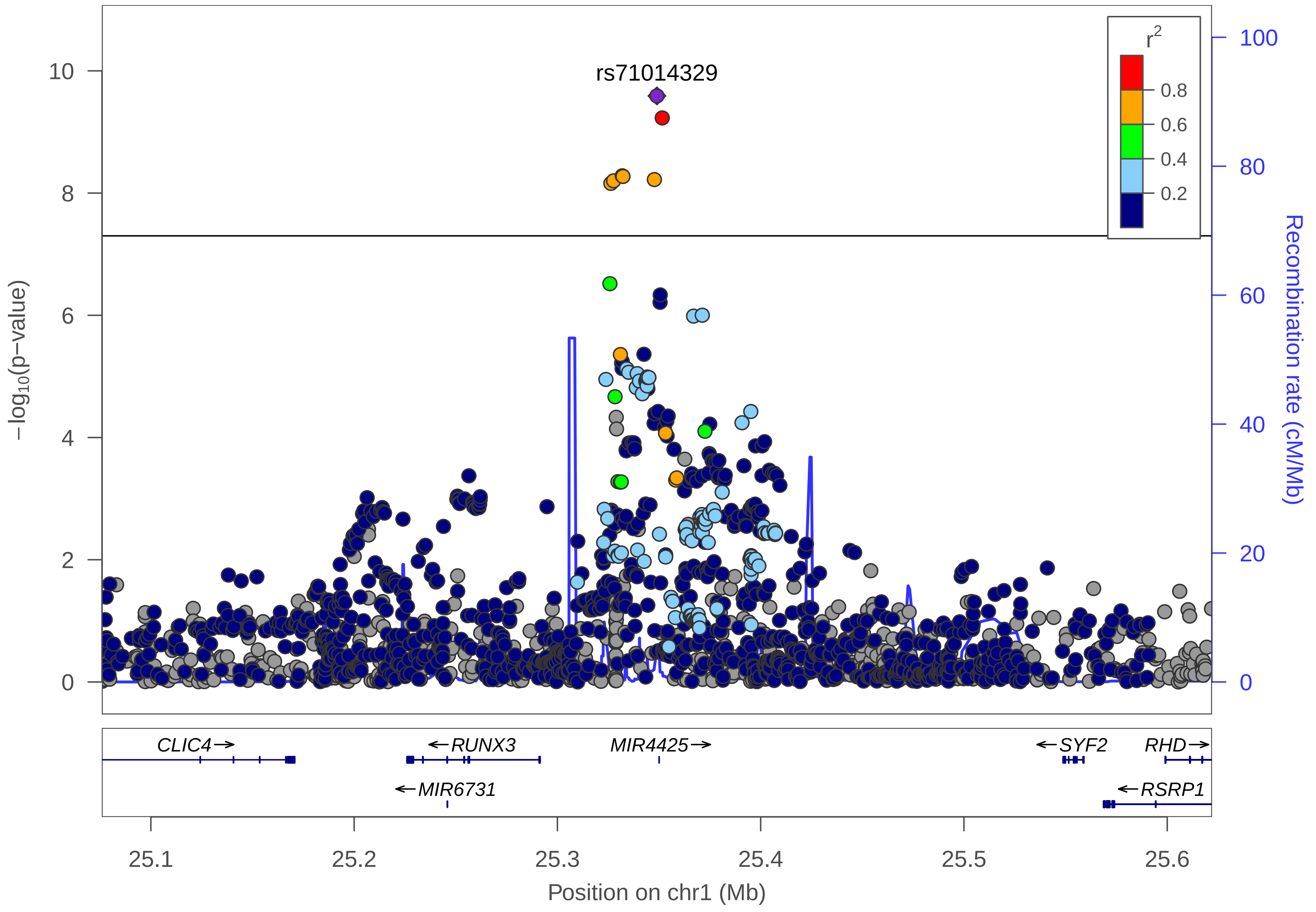

rs71014329
r2
0.8
0.6
0.4
0.2
−log10(p−value)
Recombination rate (cM/Mb)
CLIC4→
←RUNX3
MIR4425→
←SYF2
RHD→
←MIR6731
←RSRP1
25.1
25.2
25.3
25.4
25.5
25.6
Position on chr1 (Mb)

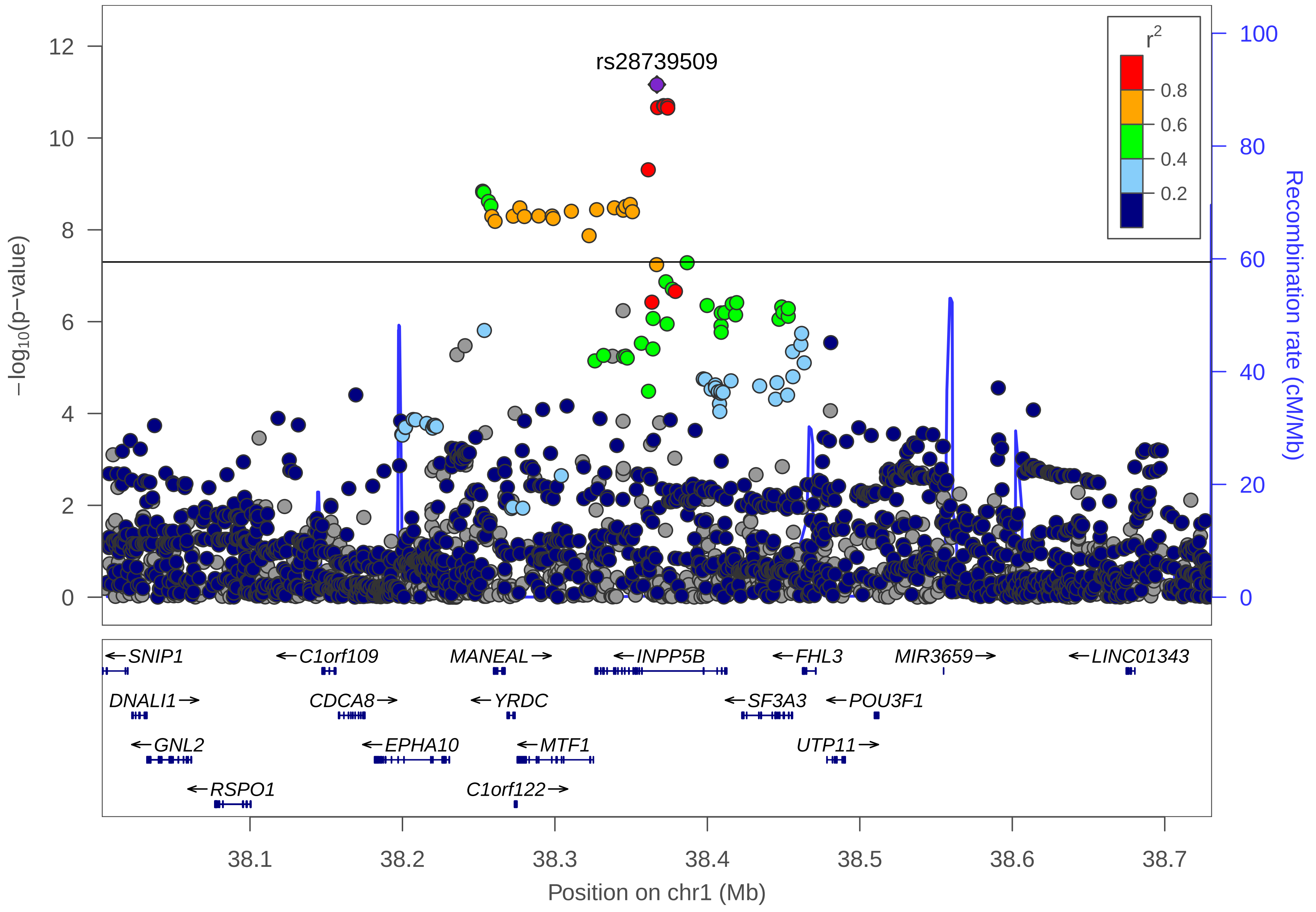
rs28739509
−log10(p−value)
Recombination rate (cM/Mb)
r2
0.8
0.6
0.4
0.2
12
10
8
6
4
2
0
100
80
60
40
20
0
←SNIP1
←C1orf109
MANEAL→
←INPP5B
←FHL3
MIR3659→
←LINC01343
DNALI1→
CDCA8→
←YRDC
←SF3A3
←POU3F1
←GNL2
←EPHA10
←MTF1
UTP11→
←RSPO1
C1orf122→
38.1
38.2
38.3
38.4
38.5
38.6
38.7
Position on chr1 (Mb)

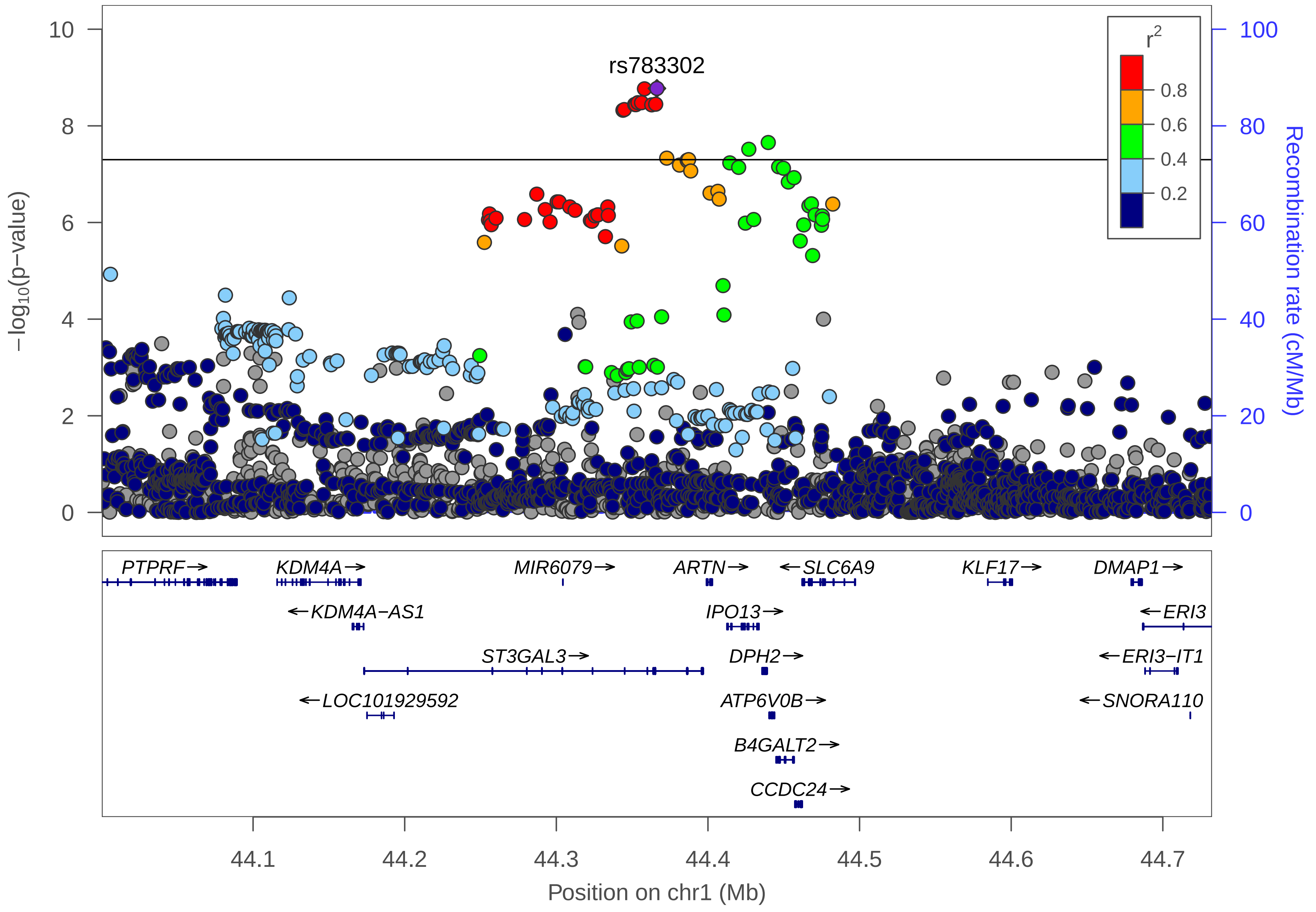
rs783302
r2
0.8
0.6
0.4
0.2
−log10(p−value)
Recombination rate (cM/Mb)
0
2
4
6
8
10
0
20
40
60
80
100
PTPRF→
KDM4A→
MIR6079→
ARTN→
←SLC6A9
KLF17→
DMAP1→
←KDM4A−AS1
IPO13→
←ERI3
ST3GAL3→
DPH2→
←ERI3−IT1
←LOC101929592
ATP6V0B→
←SNORA110
B4GALT2→
CCDC24→
44.1
44.2
44.3
44.4
44.5
44.6
44.7
Position on chr1 (Mb)

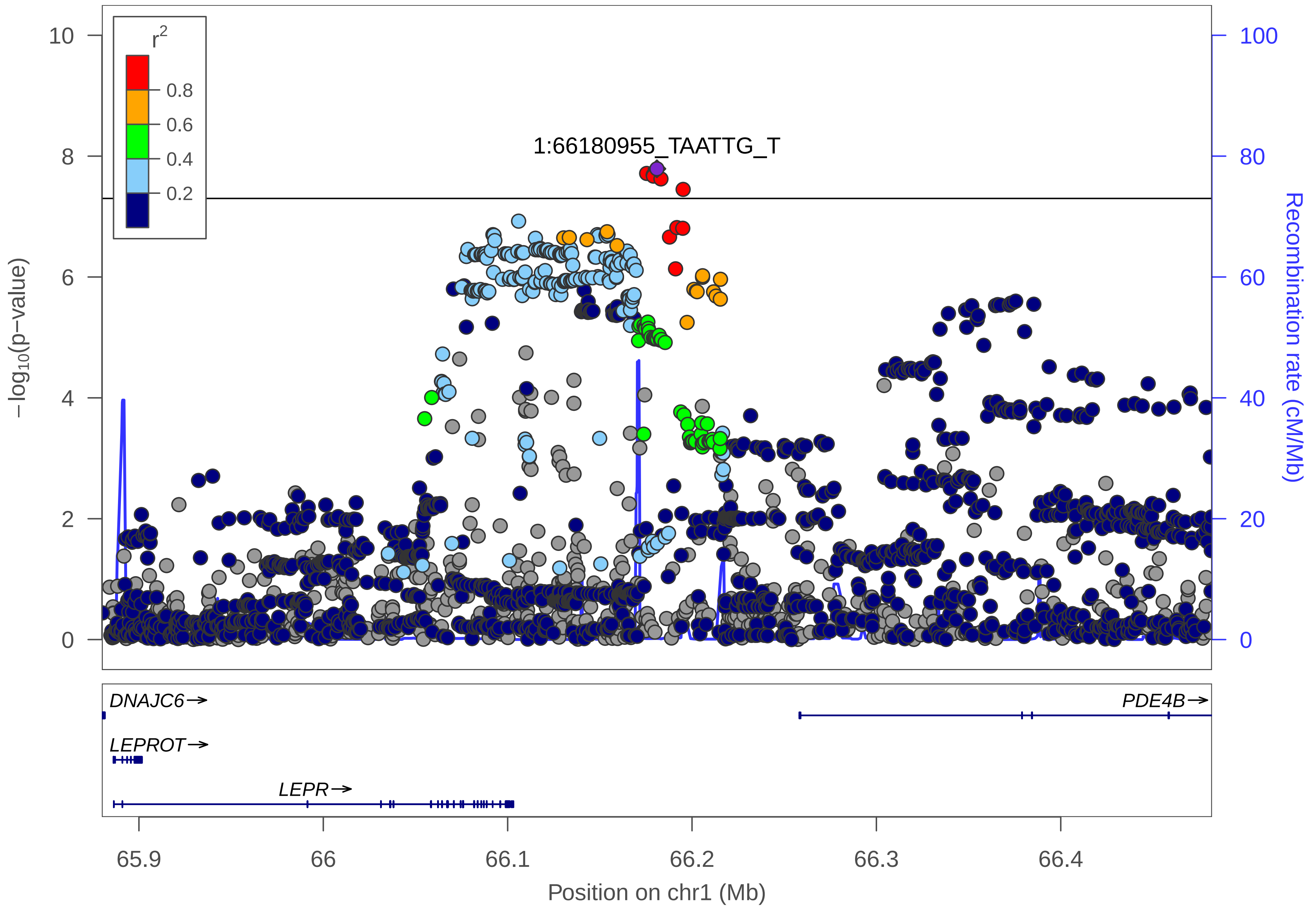

r2
0.8
0.6
0.4
0.2
10
8
6
4
2
0
−log10(p−value)
100
80
60
40
20
0
Recombination rate (cM/Mb)
1:66180955_TAATTG_T
DNAJC6→
LEPROT→
LEPR→
PDE4B→
65.9
66
66.1
66.2
66.3
66.4
Position on chr1 (Mb)

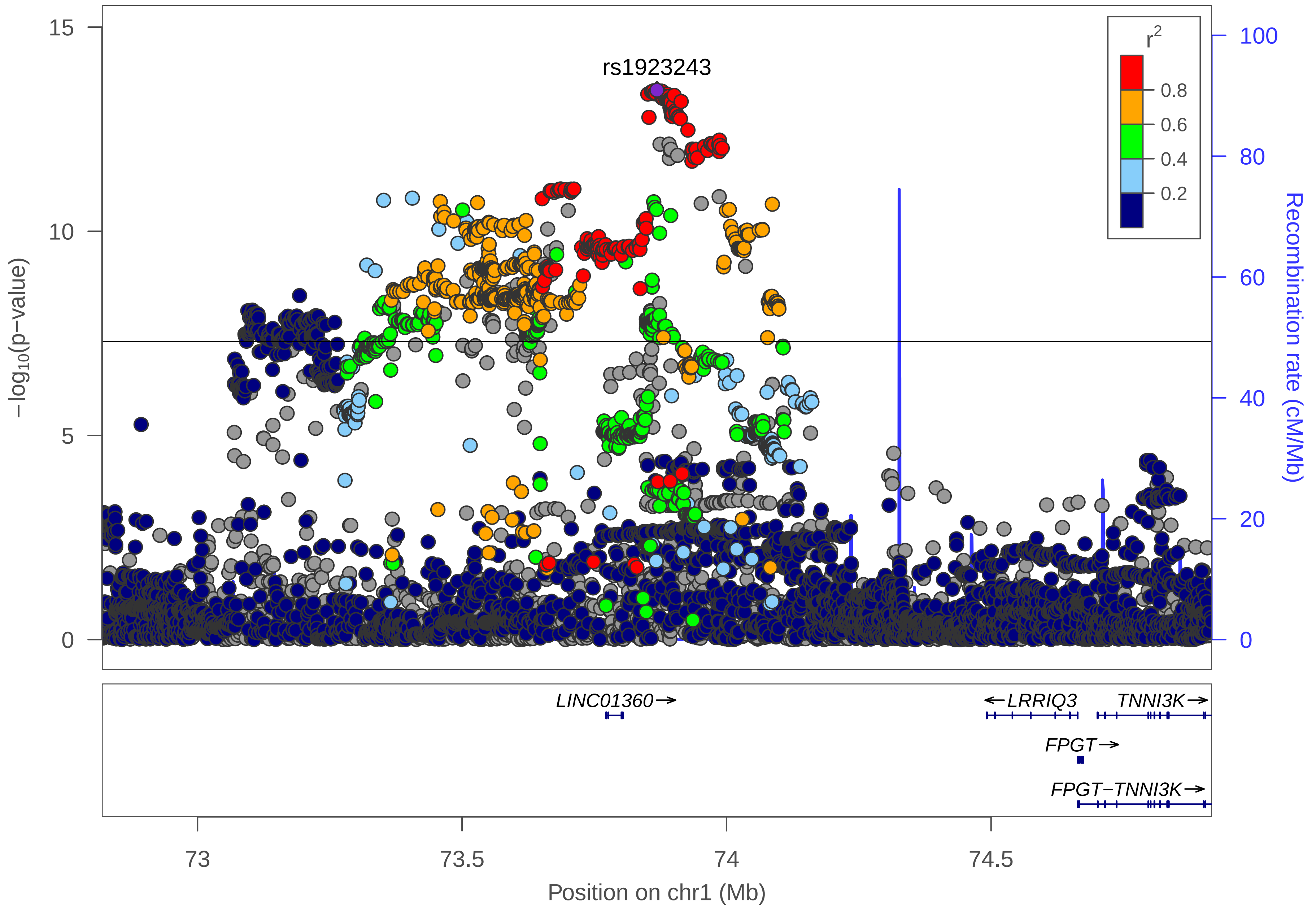
rs1923243
r^2
0.8
0.6
0.4
0.2
15
10
5
0
100
80
60
40
20
0
−log10(p−value)
Recombination rate (cM/Mb)
LINC01360→
←LRRIQ3
TNNI3K→
FPGT→
FPGT−TNNI3K→
73
73.5
74
74.5
Position on chr1 (Mb)

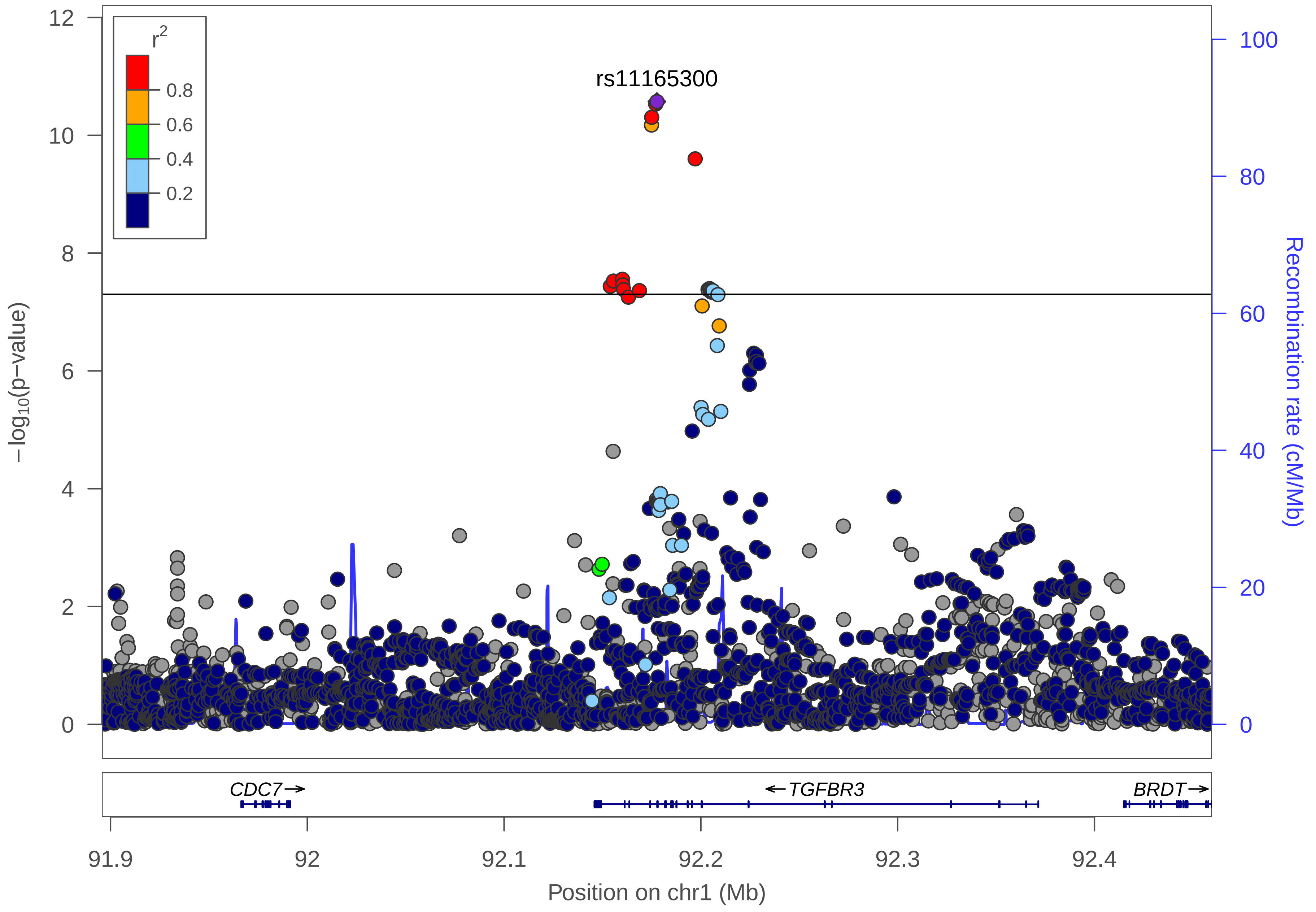
r2
0.8
0.6
0.4
0.2
rs11165300
12
10
8
6
4
2
0
−log10(p−value)
100
80
60
40
20
0
Recombination rate (cM/Mb)
CDC7→
←TGFBR3
BRDT→
91.9
92
92.1
92.2
92.3
92.4
Position on chr1 (Mb)

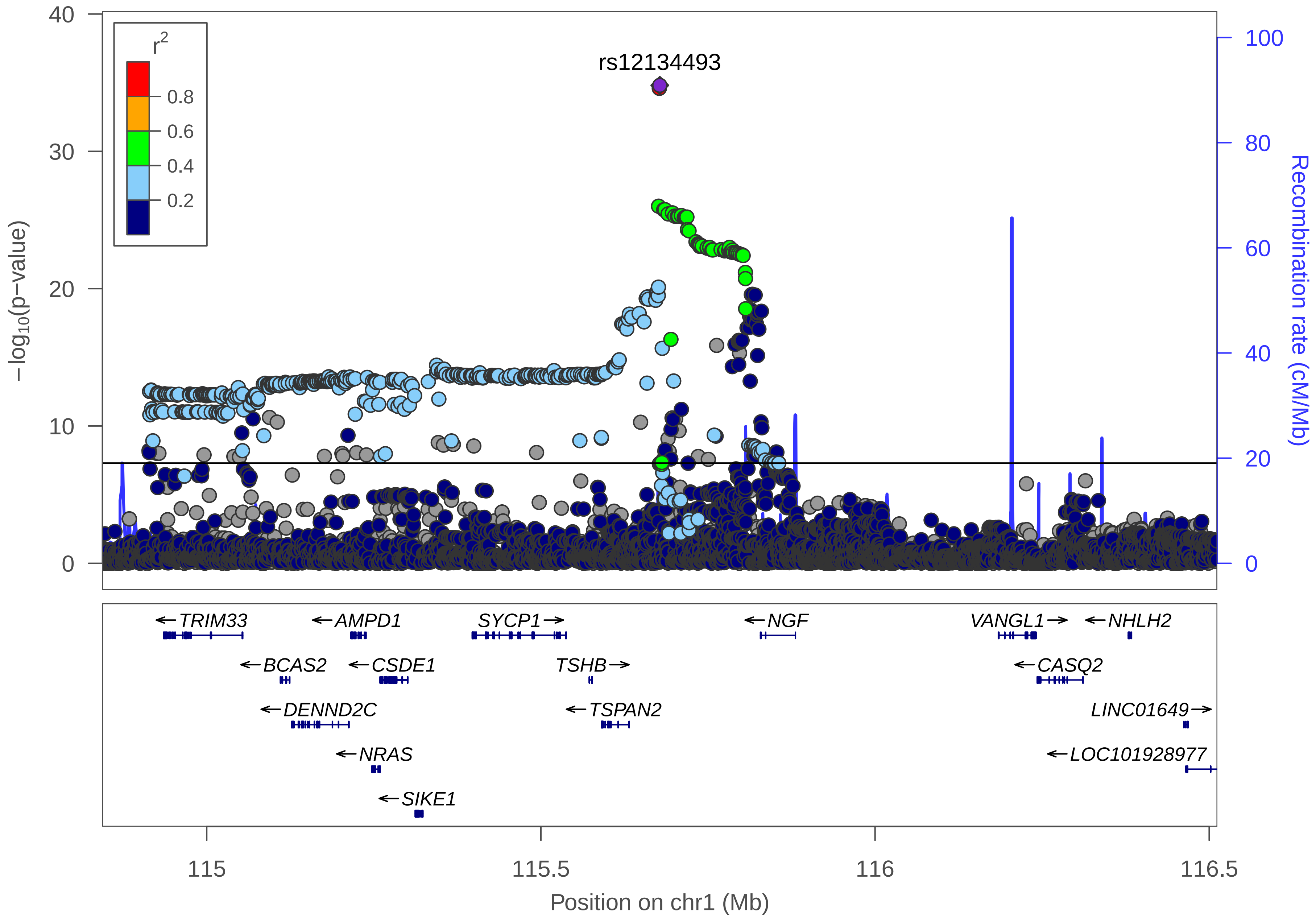
rs12134493
r2
0.8
0.6
0.4
0.2
−log10(p−value)
0
10
20
30
40
Recombination rate (cM/Mb)
0
20
40
60
80
100
TRIM33
AMPD1
SYCP1
NGF
VANGL1
NHLH2
BCAS2
CSDE1
TSHB
CASQ2
DENND2C
TSPAN2
LINC01649
NRAS
LOC101928977
SIKE1
115
115.5
116
116.5
Position on chr1 (Mb)

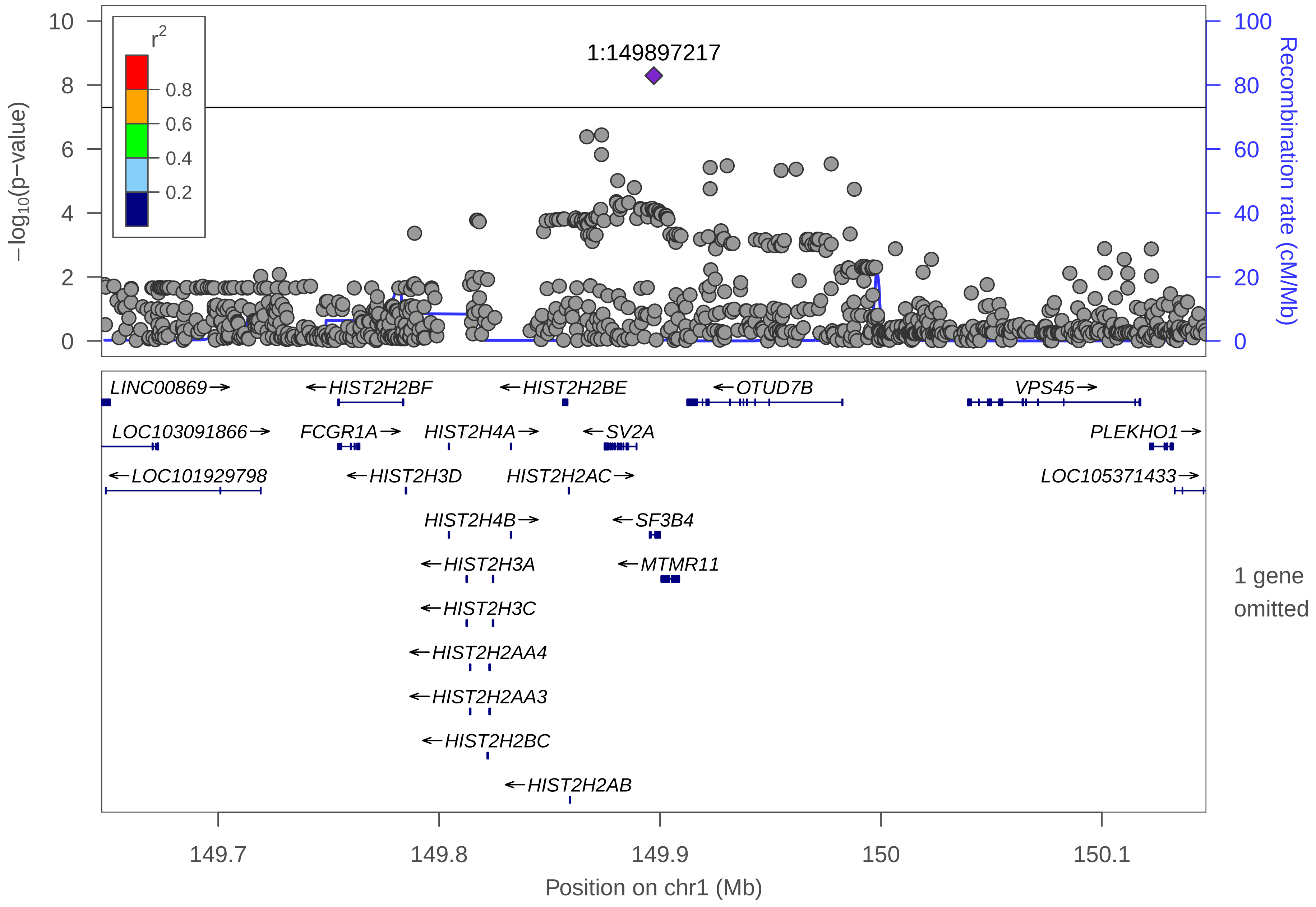
1:149897217
r2
0.8
0.6
0.4
0.2
−log10(p−value)
Recombination rate (cM/Mb)
LINC00869
HIST2H2BF
HIST2H2BE
OTUD7B
VPS45
LOC103091866
FCGR1A
HIST2H4A
SV2A
PLEKHO1
LOC101929798
HIST2H3D
HIST2H2AC
LOC105371433
HIST2H4B
SF3B4
HIST2H3A
MTMR11
HIST2H3C
HIST2H2AA4
HIST2H2AA3
HIST2H2BC
HIST2H2AB
1 gene omitted
149.7
149.8
149.9
150
150.1
Position on chr1 (Mb)

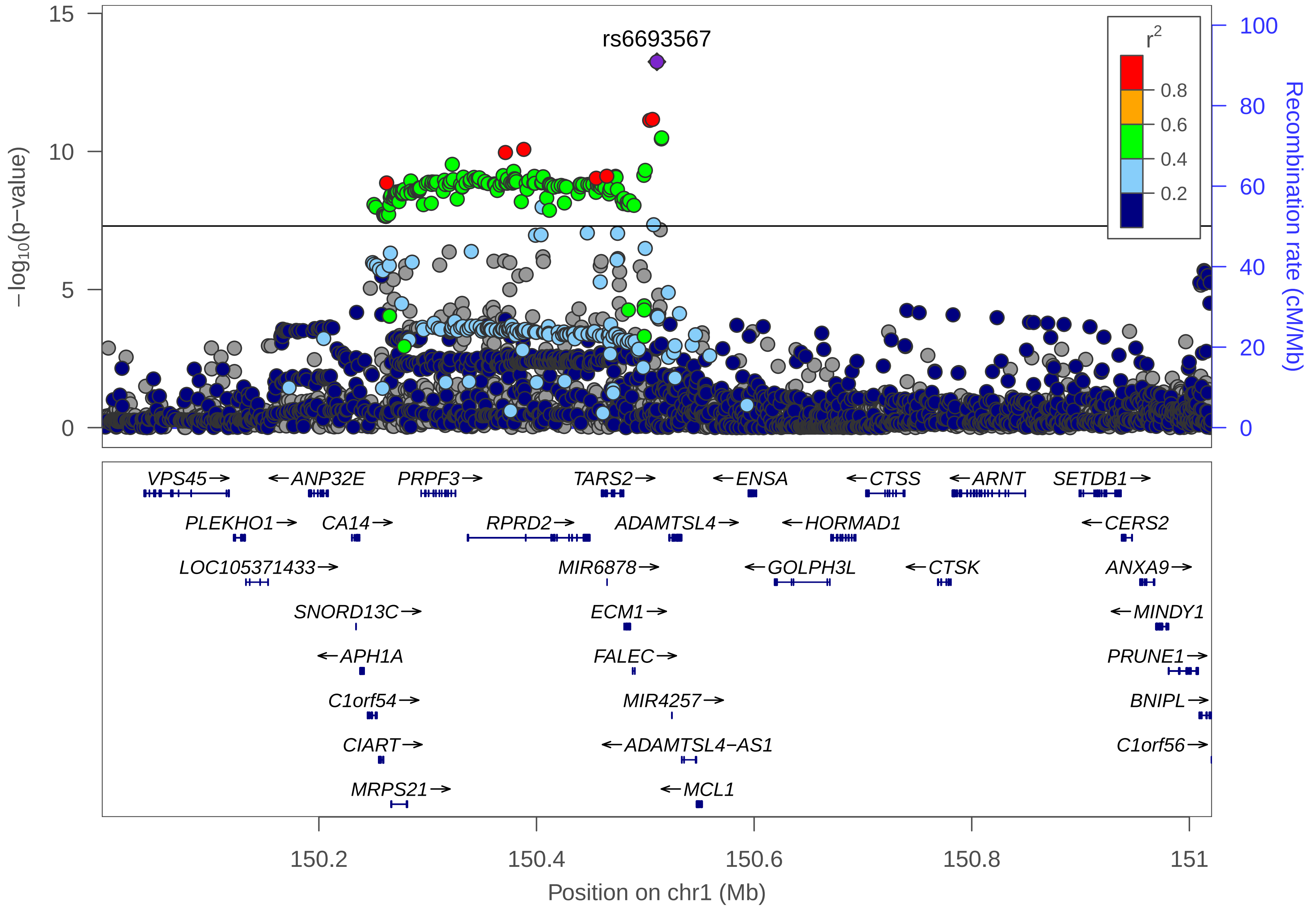

rs6693567
r2
0.8
0.6
0.4
0.2
−log10(p−value)
Recombination rate (cM/Mb)
VPS45→
←ANP32E
PRPF3→
TARS2→
←ENSA
←CTSS
←ARNT
SETDB1→
PLEKHO1→
CA14→
RPRD2→
ADAMTSL4→
←HORMAD1
←CERS2
LOC105371433→
MIR6878→
←GOLPH3L
←CTSK
ANXA9→
SNORD13C→
ECM1→
←MINDY1
←APH1A
FALEC→
PRUNE1→
C1orf54→
MIR4257→
BNIPL→
CIART→
←ADAMTSL4−AS1
C1orf56→
MRPS21→
←MCL1
150.2
150.4
150.6
150.8
151
Position on chr1 (Mb)

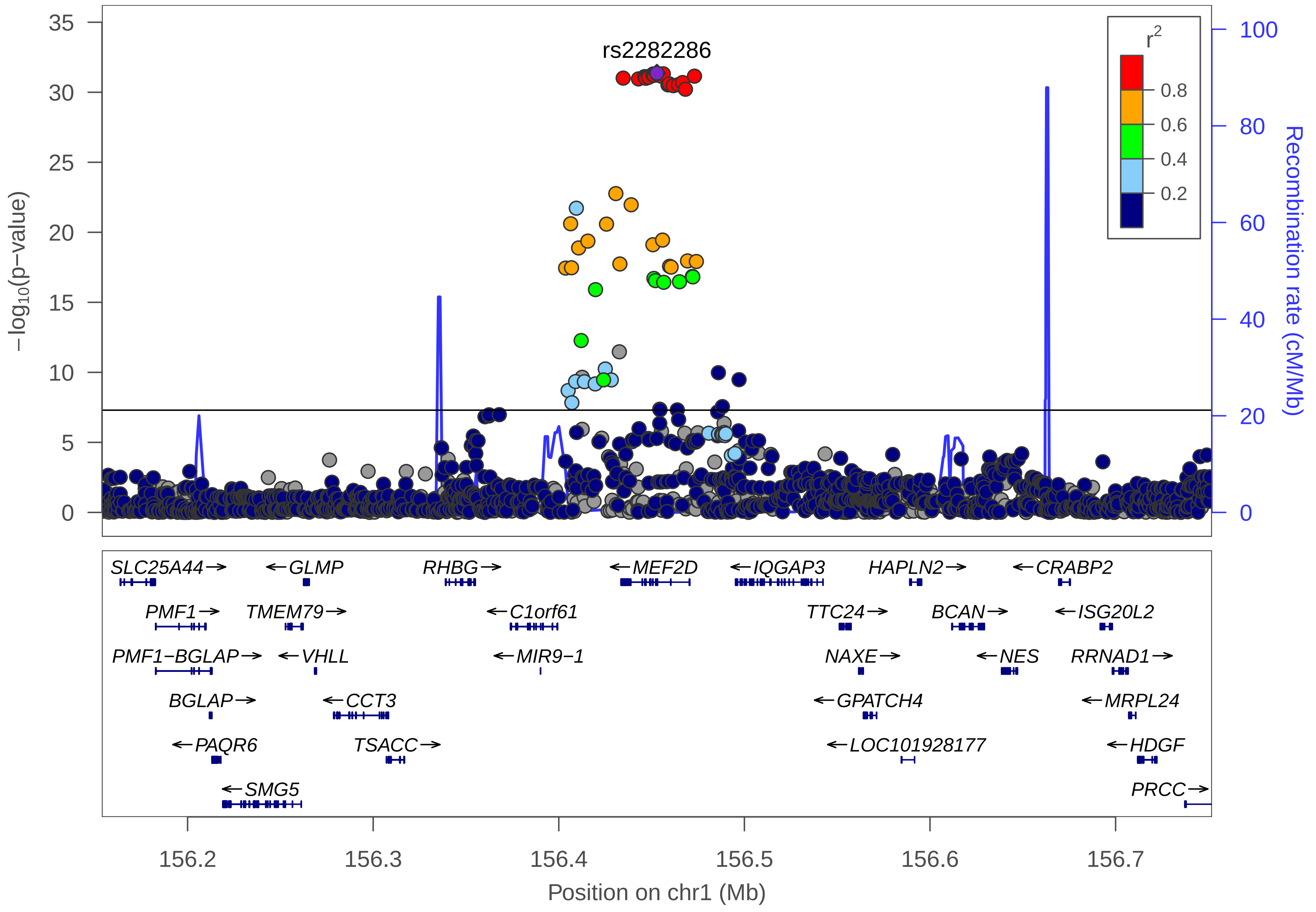

rs2282286
r2
0.8
0.6
0.4
0.2
−log10(p−value)
Recombination rate (cM/Mb)
35
30
25
20
15
10
5
0
100
80
60
40
20
0
SLC25A44→
←GLMP
RHBG→
←MEF2D
←IQGAP3
HAPLN2→
←CRABP2
PMF1→
TMEM79→
←C1orf61
TTC24→
BCAN→
←ISG20L2
PMF1−BGLAP→
←VHLL
←MIR9−1
NAXE→
←NES
RRNAD1→
BGLAP→
←CCT3
←GPATCH4
←MRPL24
←PAQR6
TSACC→
←LOC101928177
←HDGF
←SMG5
PRCC→
156.2
156.3
156.4
156.5
156.6
156.7
Position on chr1 (Mb)

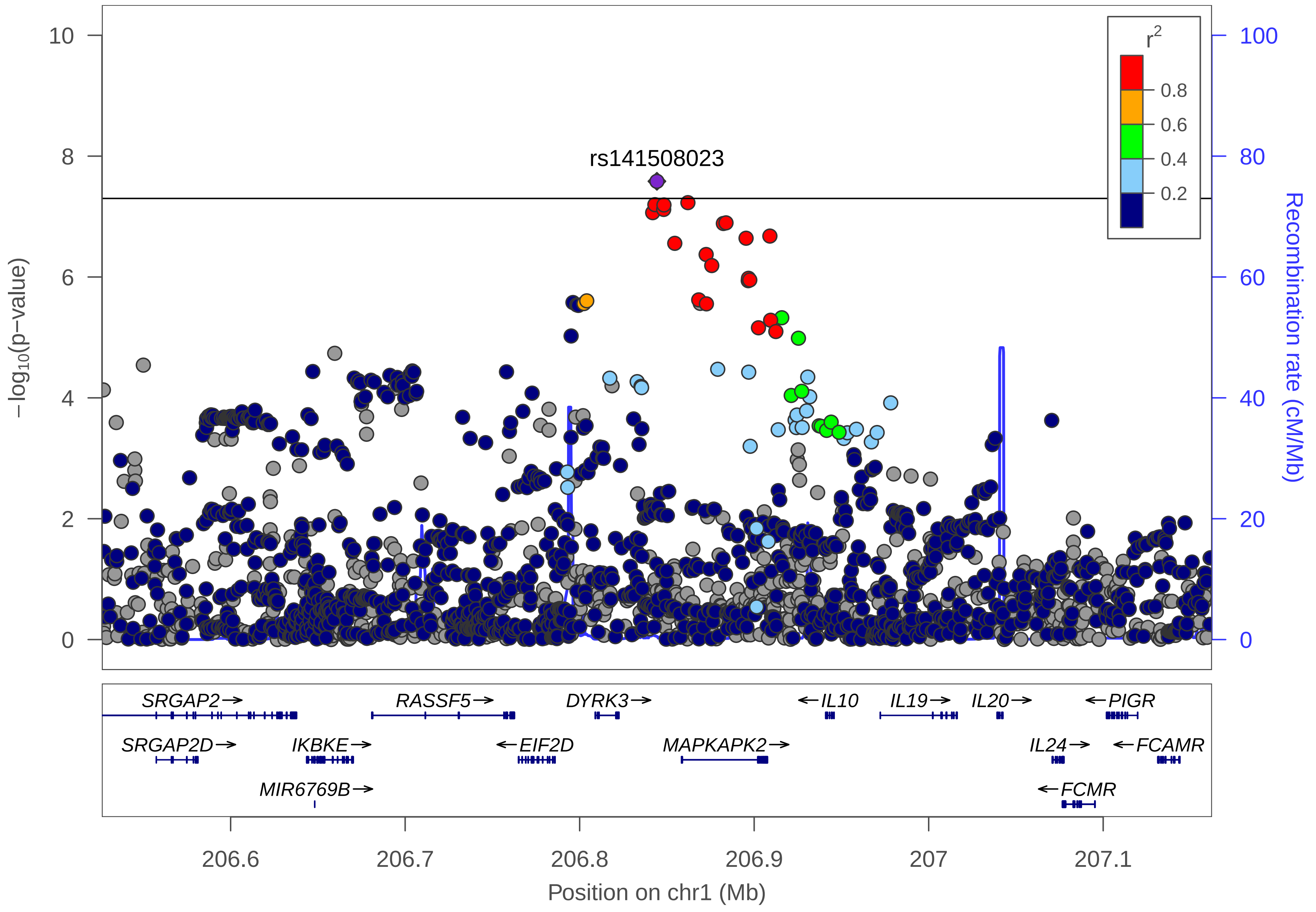
rs141508023
r2
0.8
0.6
0.4
0.2
−log10(p−value)
Recombination rate (cM/Mb)
0
2
4
6
8
10
0
20
40
60
80
100
SRGAP2→
RASSF5→
DYRK3→
←IL10
IL19→
IL20→
←PIGR
SRGAP2D→
IKBKE→
←EIF2D
MAPKAPK2→
IL24→
←FCAMR
MIR6769B→
←FCMR
206.6
206.7
206.8
206.9
207
207.1
Position on chr1 (Mb)

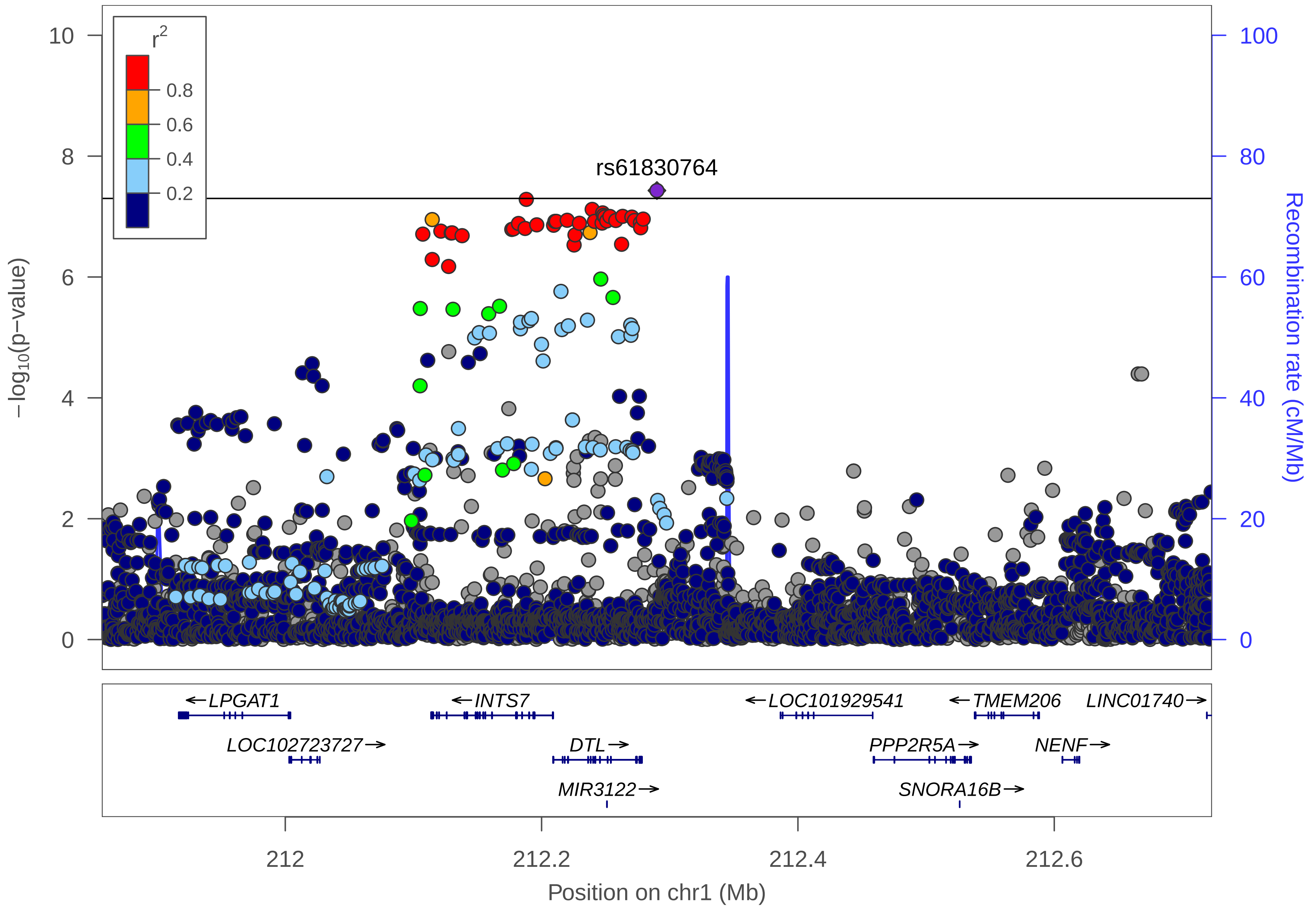

r2
0.8
0.6
0.4
0.2
rs61830764
−log10(p−value)
Recombination rate (cM/Mb)
10
8
6
4
2
0
100
80
60
40
20
0
LPGAT1
INTS7
LOC101929541
TMEM206
LINC01740
LOC102723727
DTL
PPP2R5A
NENF
MIR3122
SNORA16B
212
212.2
212.4
212.6
Position on chr1 (Mb)

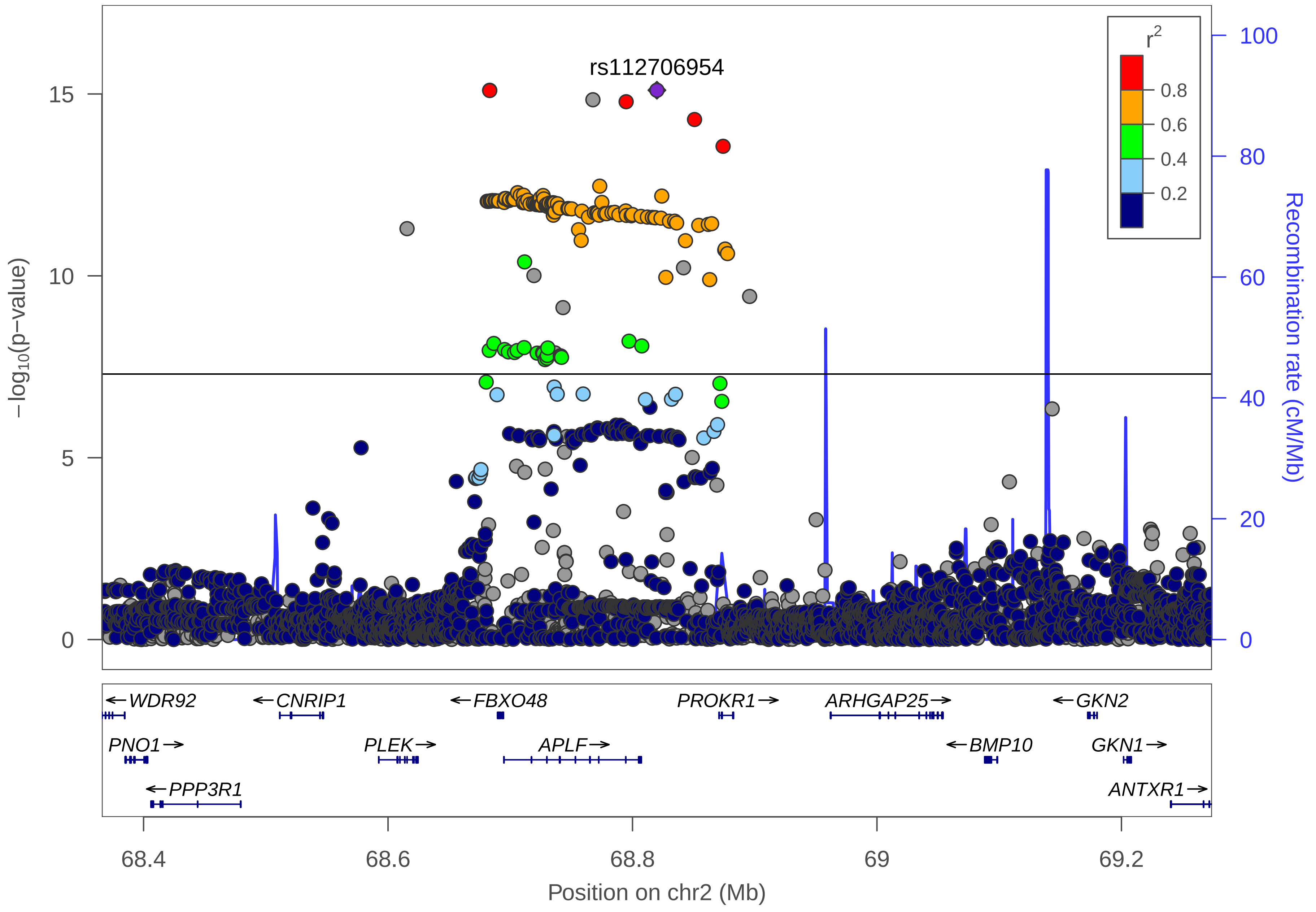

rs112706954
r2
0.8
0.6
0.4
0.2
−log10(p−value)
0
5
10
15
Recombination rate (cM/Mb)
0
20
40
60
80
100
WDR92
CNRIP1
FBXO48
PROKR1
ARHGAP25
GKN2
PNO1
PLEK
APLF
BMP10
GKN1
PPP3R1
ANTXR1
68.4
68.6
68.8
69
69.2
Position on chr2 (Mb)

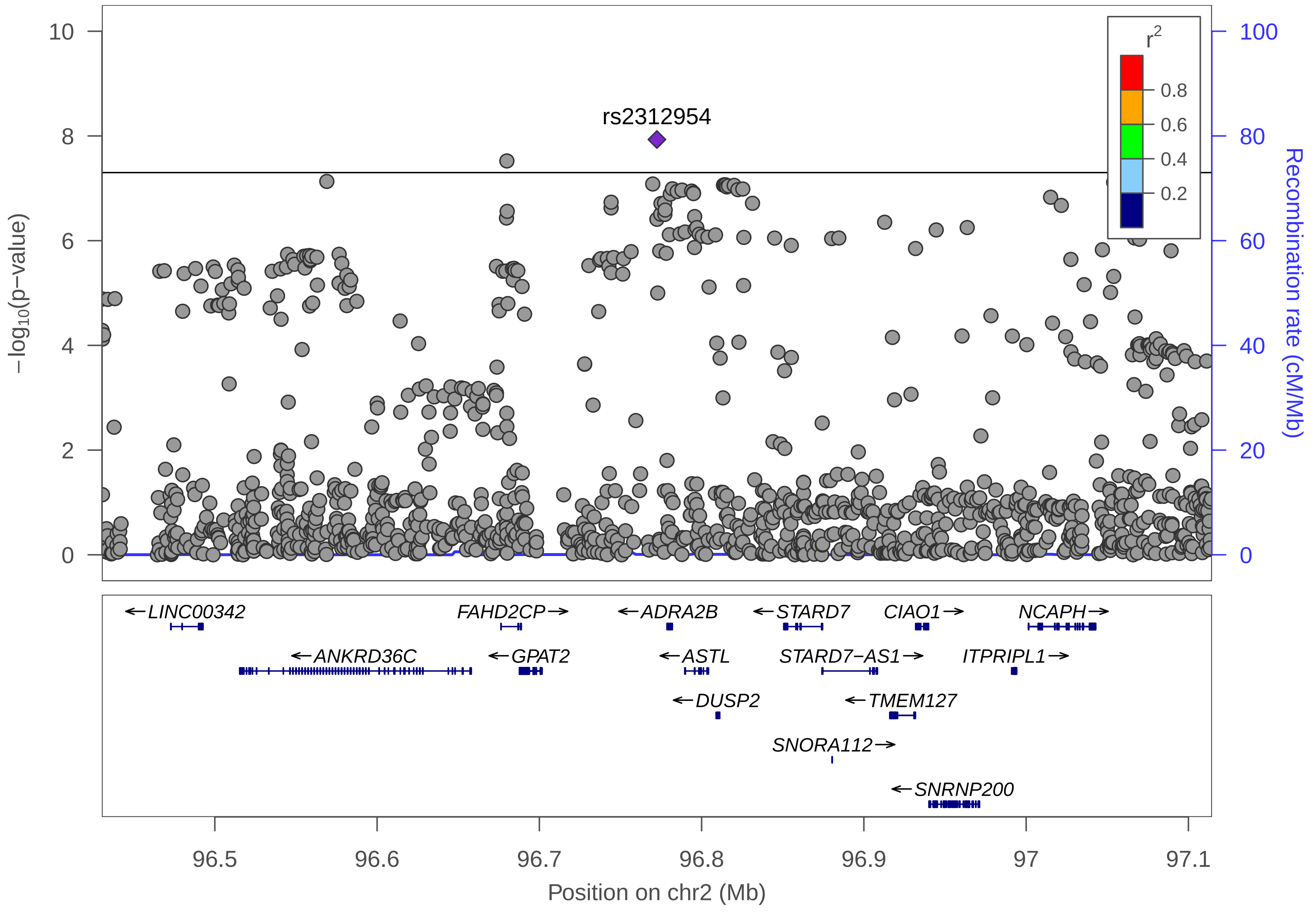

rs2312954
r2
0.8
0.6
0.4
0.2
−log10(p−value)
Recombination rate (cM/Mb)
0
2
4
6
8
10
0
20
40
60
80
100
←LINC00342
FAHD2CP→
←ADRA2B
←STARD7
CIAO1→
NCAPH→
←ANKRD36C
←GPAT2
←ASTL
STARD7−AS1→
ITPRIPL1→
←DUSP2
←TMEM127
SNORA112→
←SNRNP200
96.5
96.6
96.7
96.8
96.9
97
97.1
Position on chr2 (Mb)

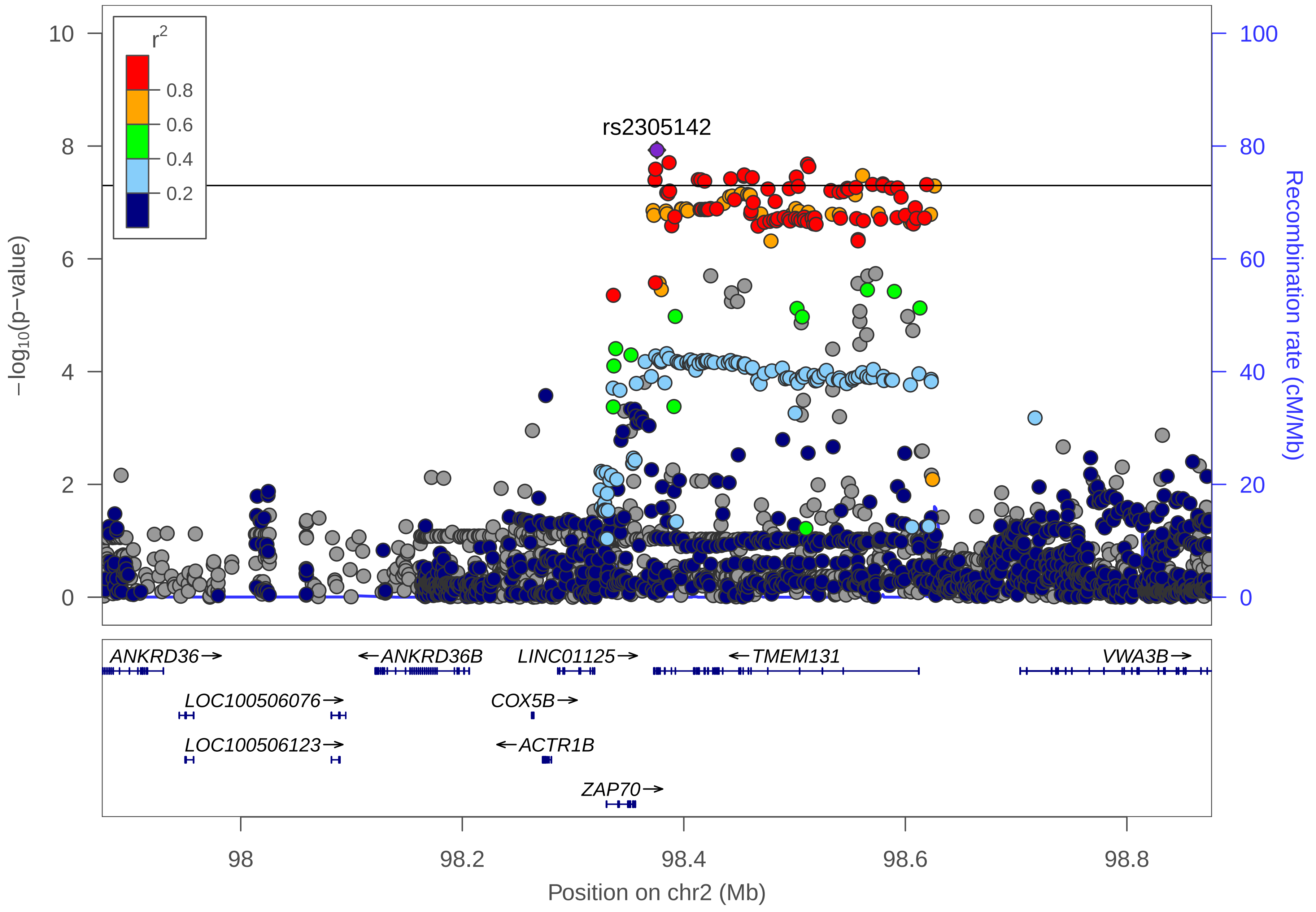

r2
0.8
0.6
0.4
0.2
rs2305142
−log10(p−value)
Recombination rate (cM/Mb)
10
8
6
4
2
0
100
80
60
40
20
0
ANKRD36→
←ANKRD36B
LINC01125→
←TMEM131
VWA3B→
LOC100506076→
COX5B→
LOC100506123→
←ACTR1B
ZAP70→
98
98.2
98.4
98.6
98.8
Position on chr2 (Mb)

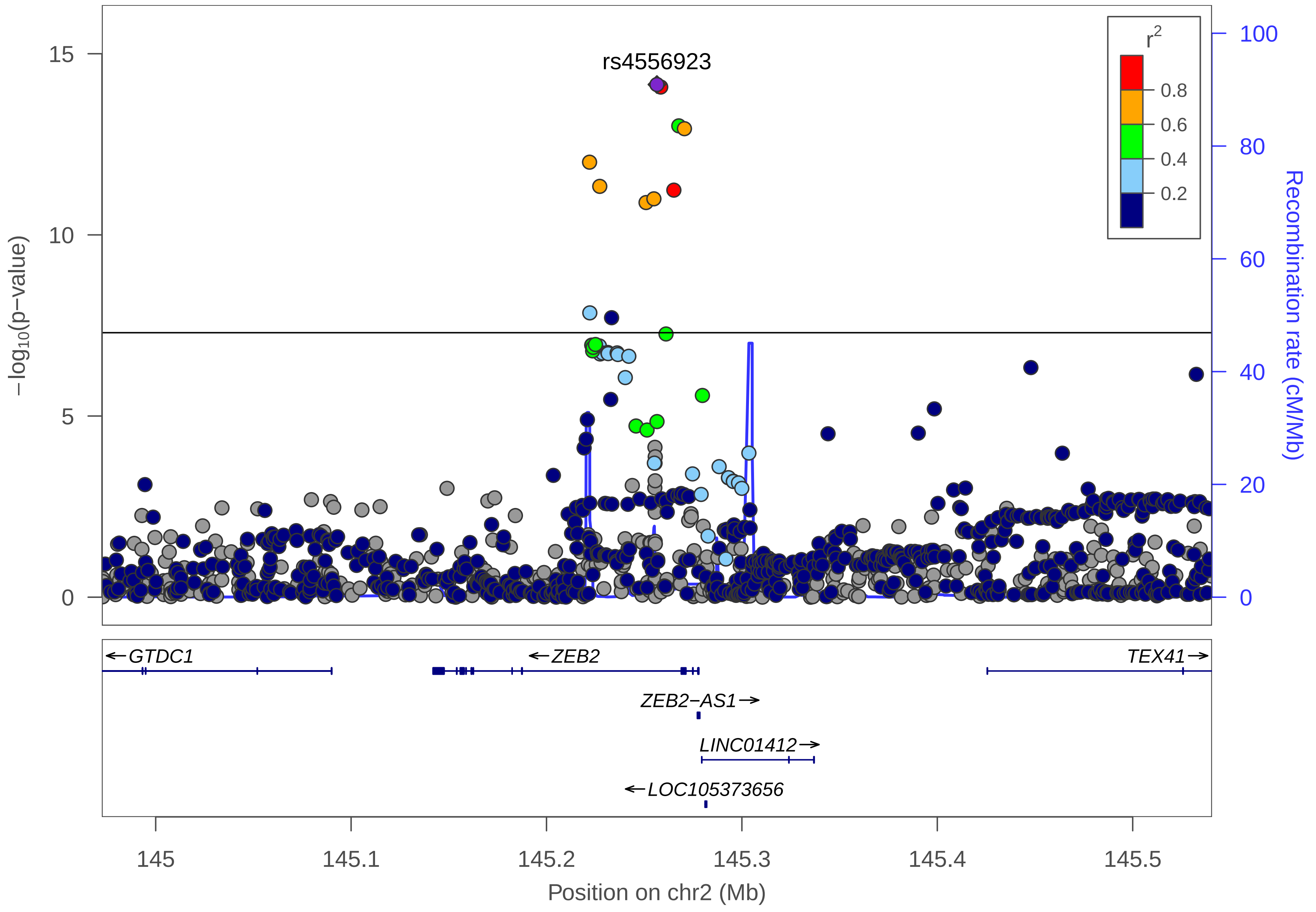

rs4556923
r2
0.8
0.6
0.4
0.2
−log10(p−value)
15
10
5
0
100
80
60
40
20
0
Recombination rate (cM/Mb)
←GTDC1
←ZEB2
TEX41→
ZEB2−AS1→
LINC01412→
←LOC105373656
145
145.1
145.2
145.3
145.4
145.5
Position on chr2 (Mb)

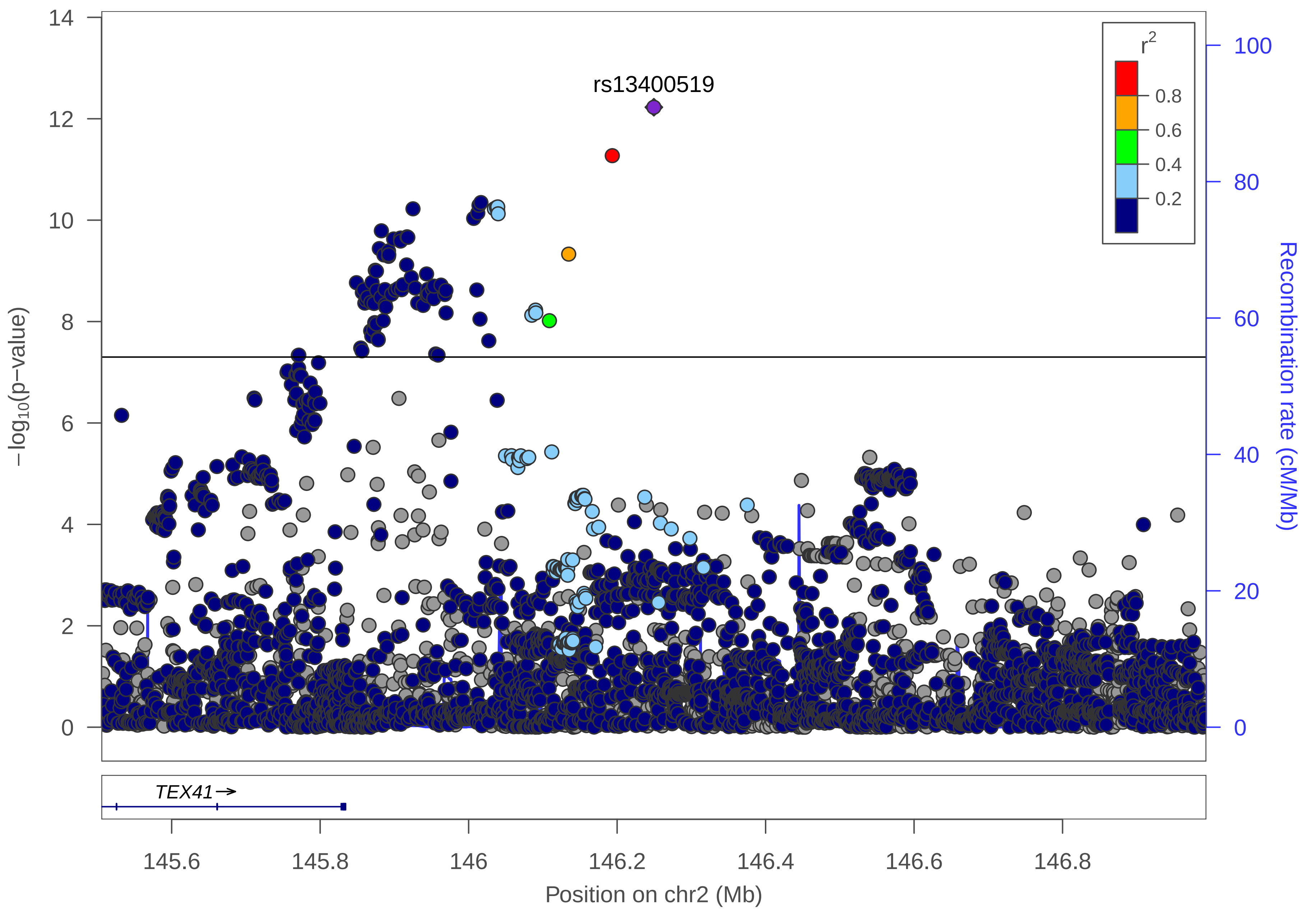
rs13400519
r2
0.8
0.6
0.4
0.2
−log10(p−value)
14
12
10
8
6
4
2
0
100
80
60
40
20
0
Recombination rate (cM/Mb)
TEX41
145.6
145.8
146
146.2
146.4
146.6
146.8
Position on chr2 (Mb)

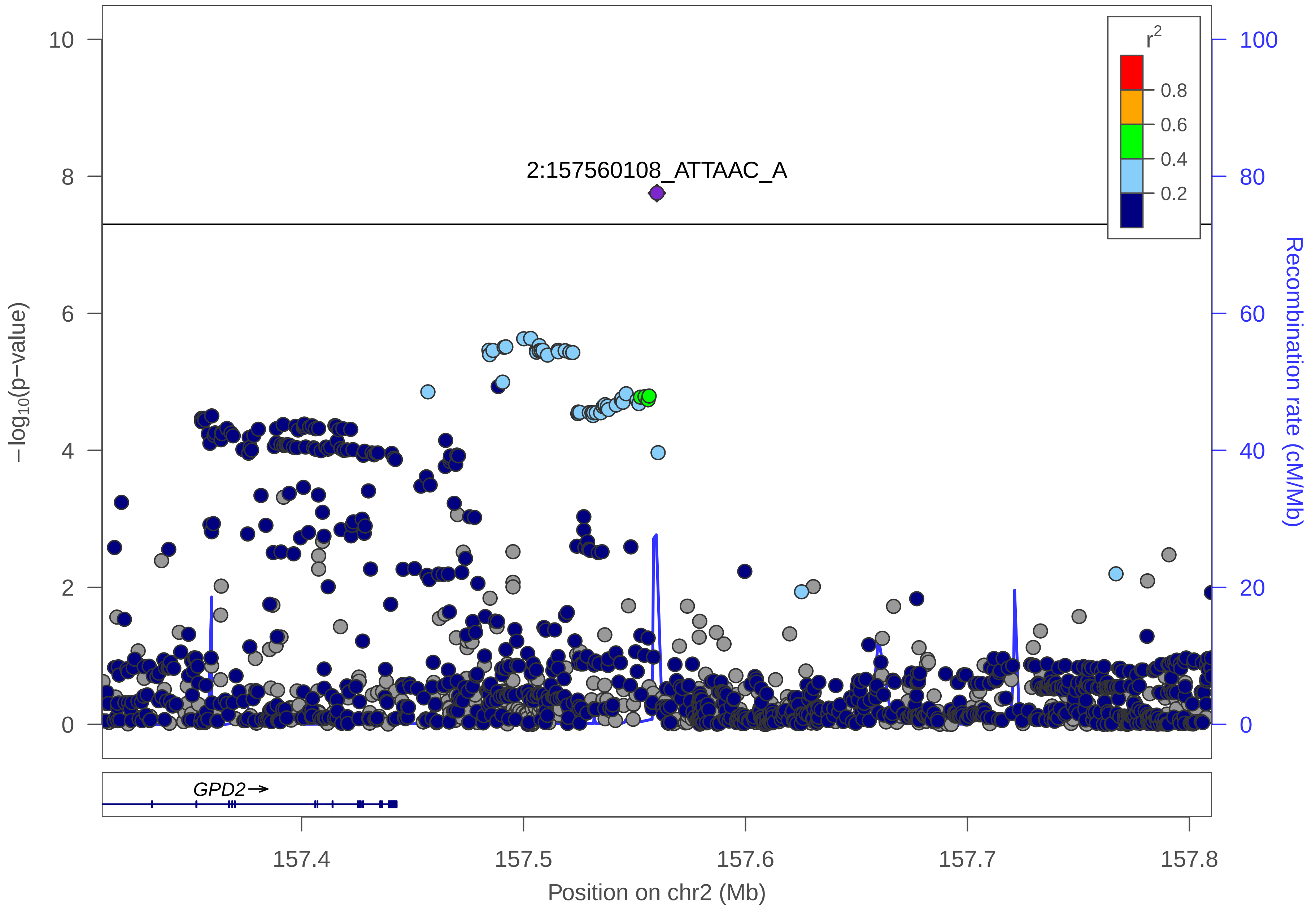

2:157560108_ATTAAC_A
r2
0.8
0.6
0.4
0.2
10
8
6
4
2
0
−log10(p−value)
100
80
60
40
20
0
Recombination rate (cM/Mb)
GPD2→
157.4
157.5
157.6
157.7
157.8
Position on chr2 (Mb)

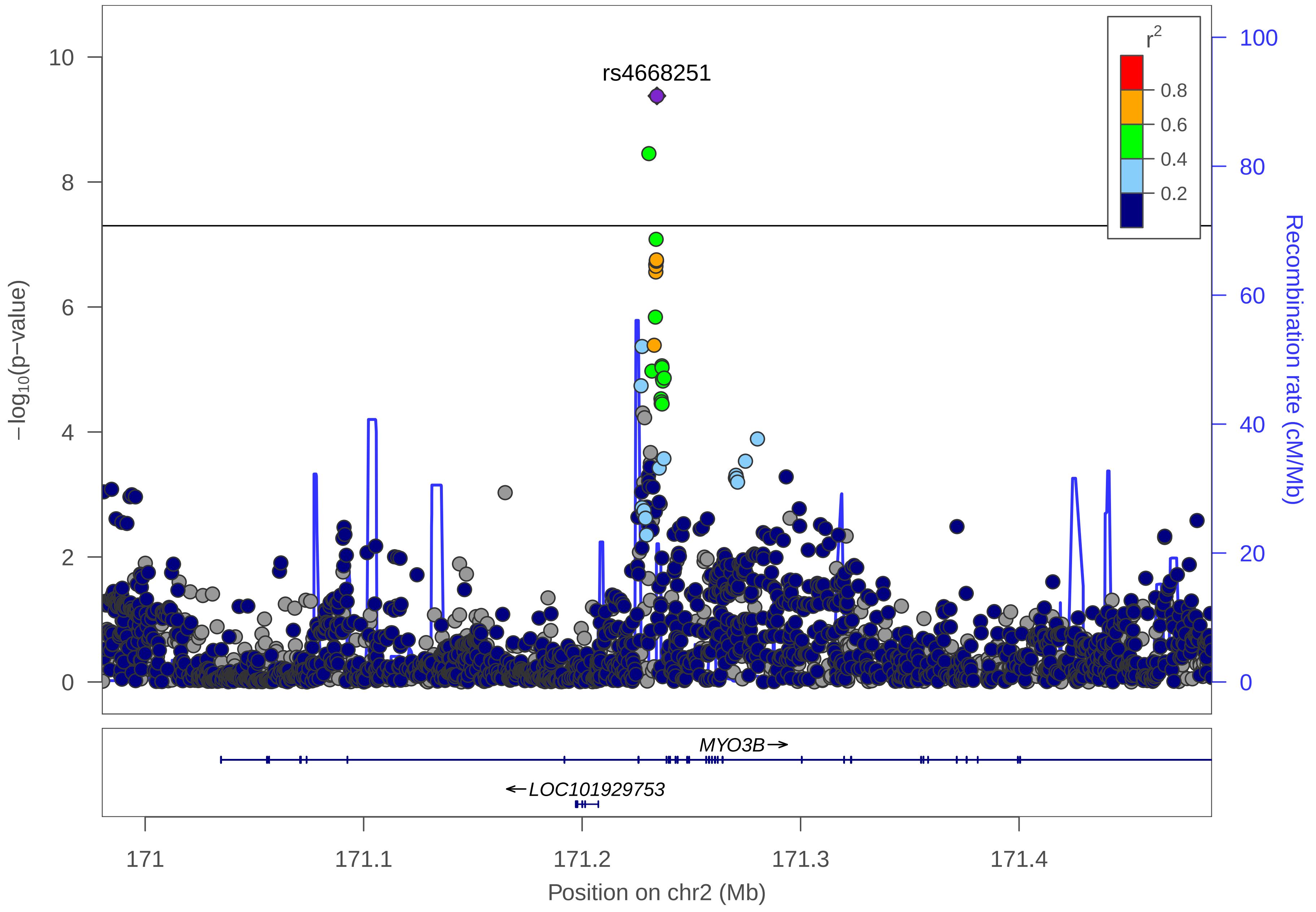

rs4668251
r2
0.8
0.6
0.4
0.2
−log10(p−value)
Recombination rate (cM/Mb)
10
8
6
4
2
0
100
80
60
40
20
0
MYO3B→
←LOC101929753
171
171.1
171.2
171.3
171.4
Position on chr2 (Mb)

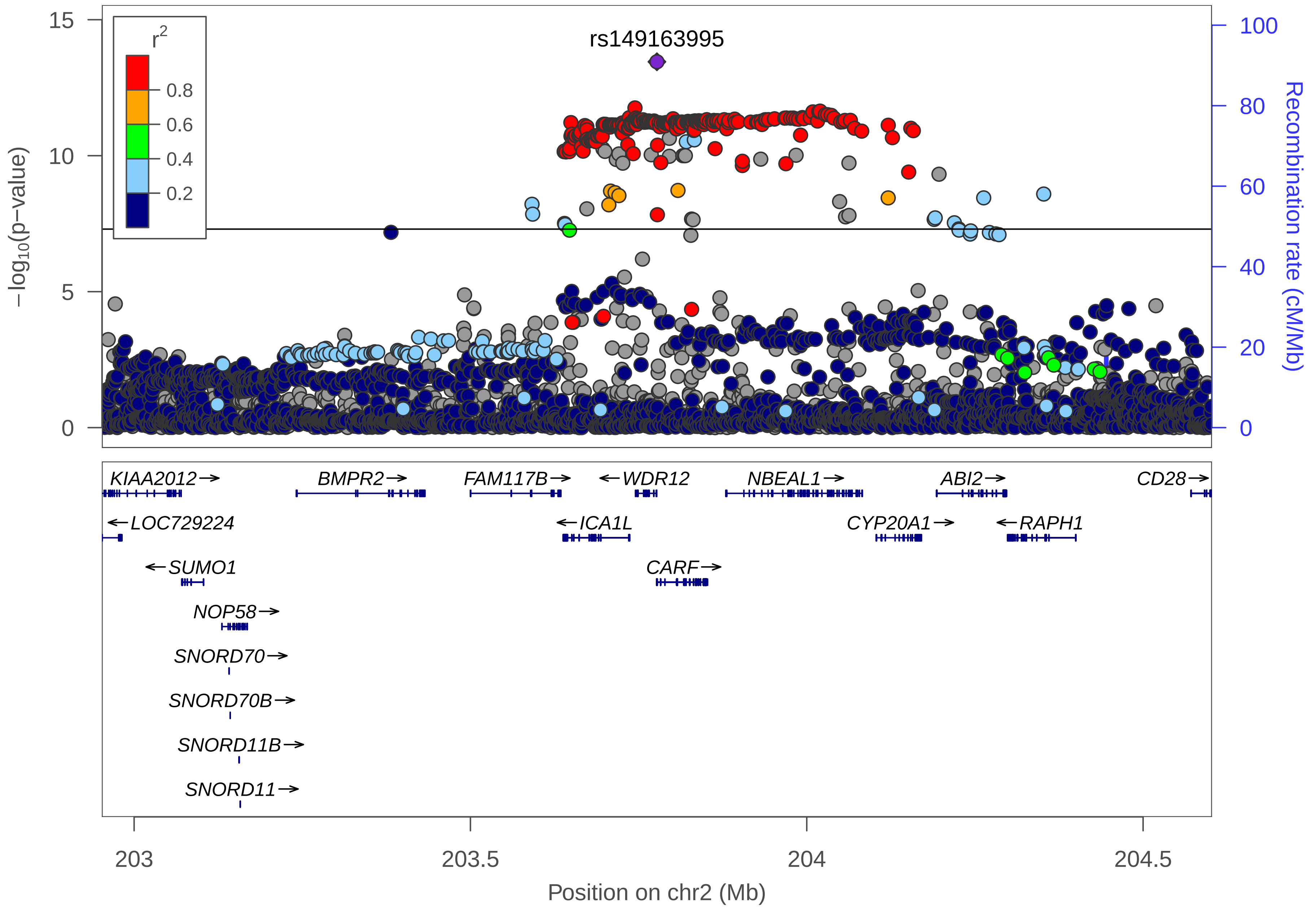

rs149163995
r2
0.8
0.6
0.4
0.2
15
10
5
0
−log10(p−value)
100
80
60
40
20
0
Recombination rate (cM/Mb)
KIAA2012→
BMPR2→
FAM117B→
←WDR12
NBEAL1→
ABI2→
CD28→
←LOC729224
←ICA1L
CYP20A1→
←RAPH1
←SUMO1
CARF→
NOP58→
SNORD70→
SNORD70B→
SNORD11B→
SNORD11→
203
203.5
204
204.5
Position on chr2 (Mb)

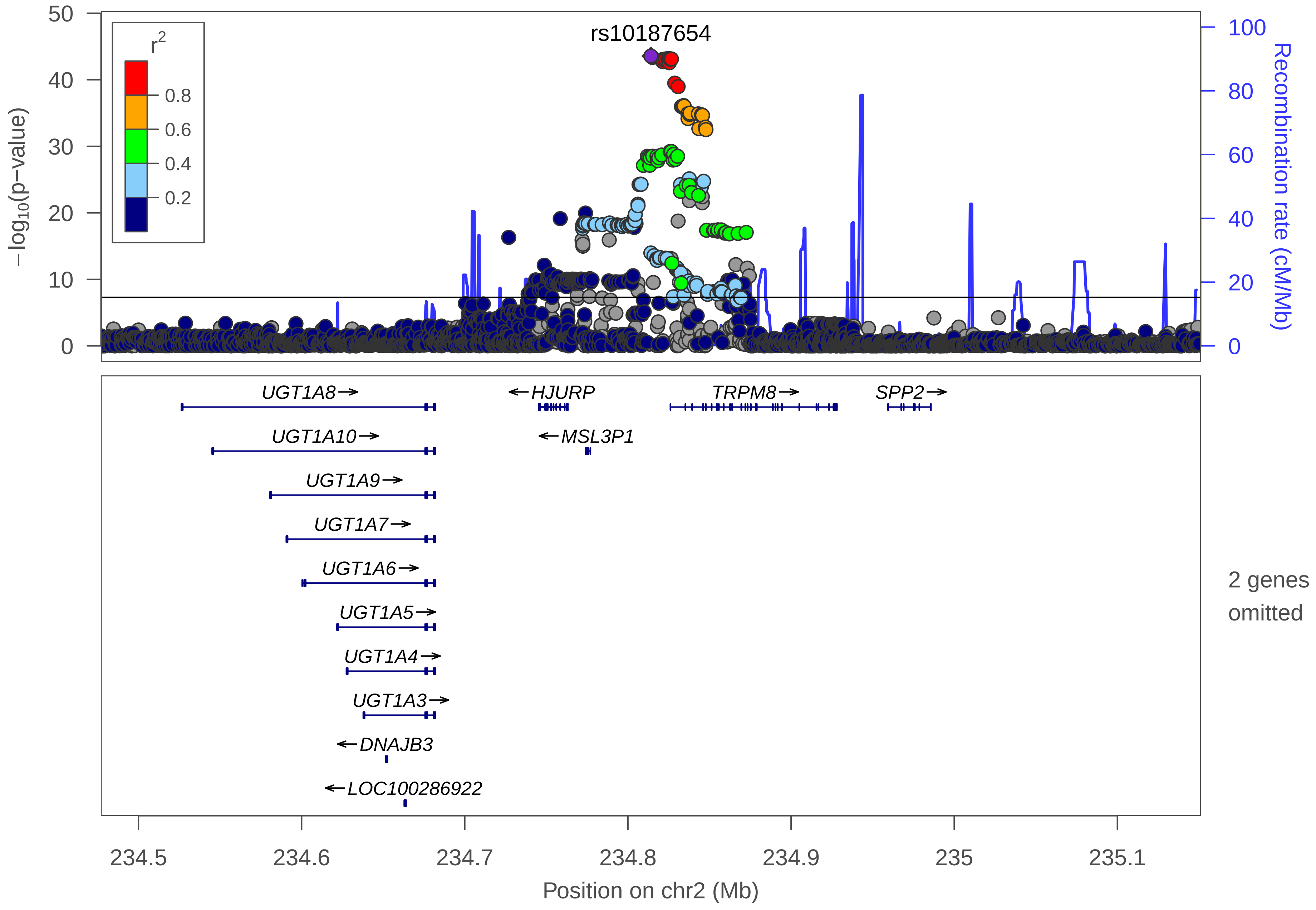

rs10187654
r2
0.8
0.6
0.4
0.2
−log10(p−value)
Recombination rate (cM/Mb)
UGT1A8→
UGT1A10→
UGT1A9→
UGT1A7→
UGT1A6→
UGT1A5→
UGT1A4→
UGT1A3→
←DNAJB3
←LOC100286922
←HJURP
←MSL3P1
TRPM8→
SPP2→
2 genes omitted
234.5
234.6
234.7
234.8
234.9
235
235.1
Position on chr2 (Mb)

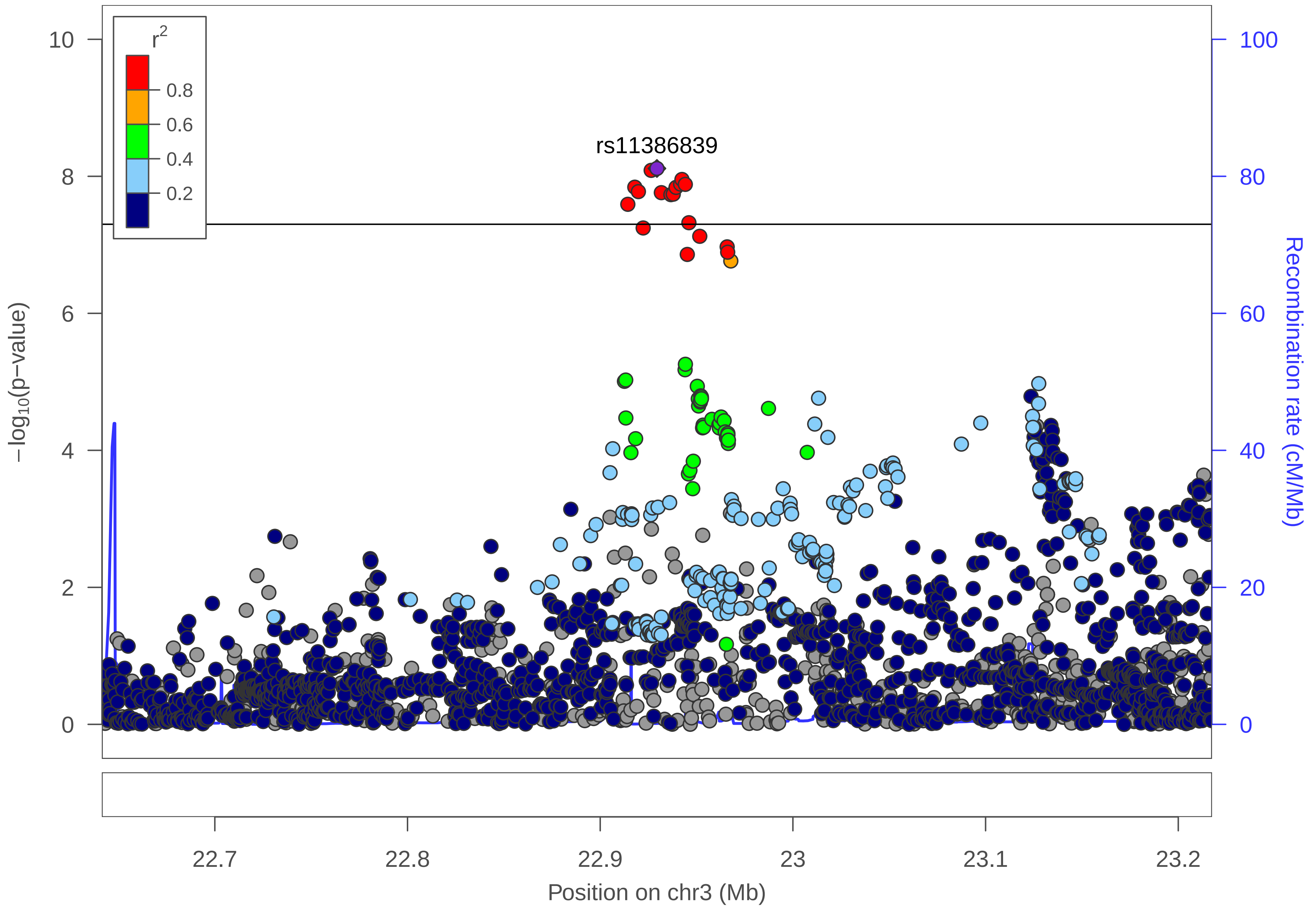

r2
0.8
0.6
0.4
0.2
rs11386839
10
8
6
4
2
0
−log10(p−value)
100
80
60
40
20
0
Recombination rate (cM/Mb)
22.7
22.8
22.9
23
23.1
23.2
Position on chr3 (Mb)

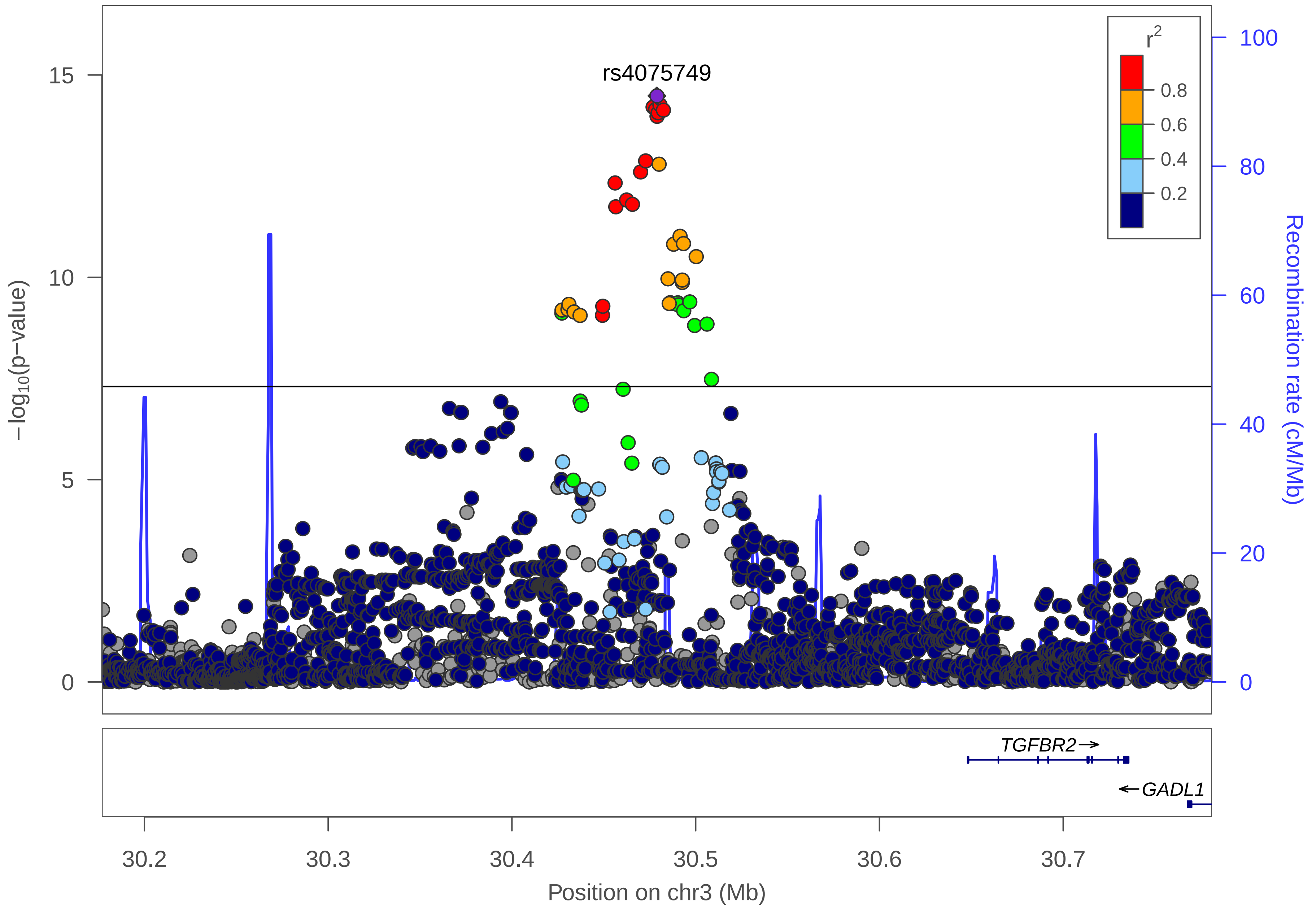
rs4075749
r2
0.8
0.6
0.4
0.2
15
10
5
0
100
80
60
40
20
0
−log10(p−value)
Recombination rate (cM/Mb)
TGFBR2→
←GADL1
30.2
30.3
30.4
30.5
30.6
30.7
Position on chr3 (Mb)

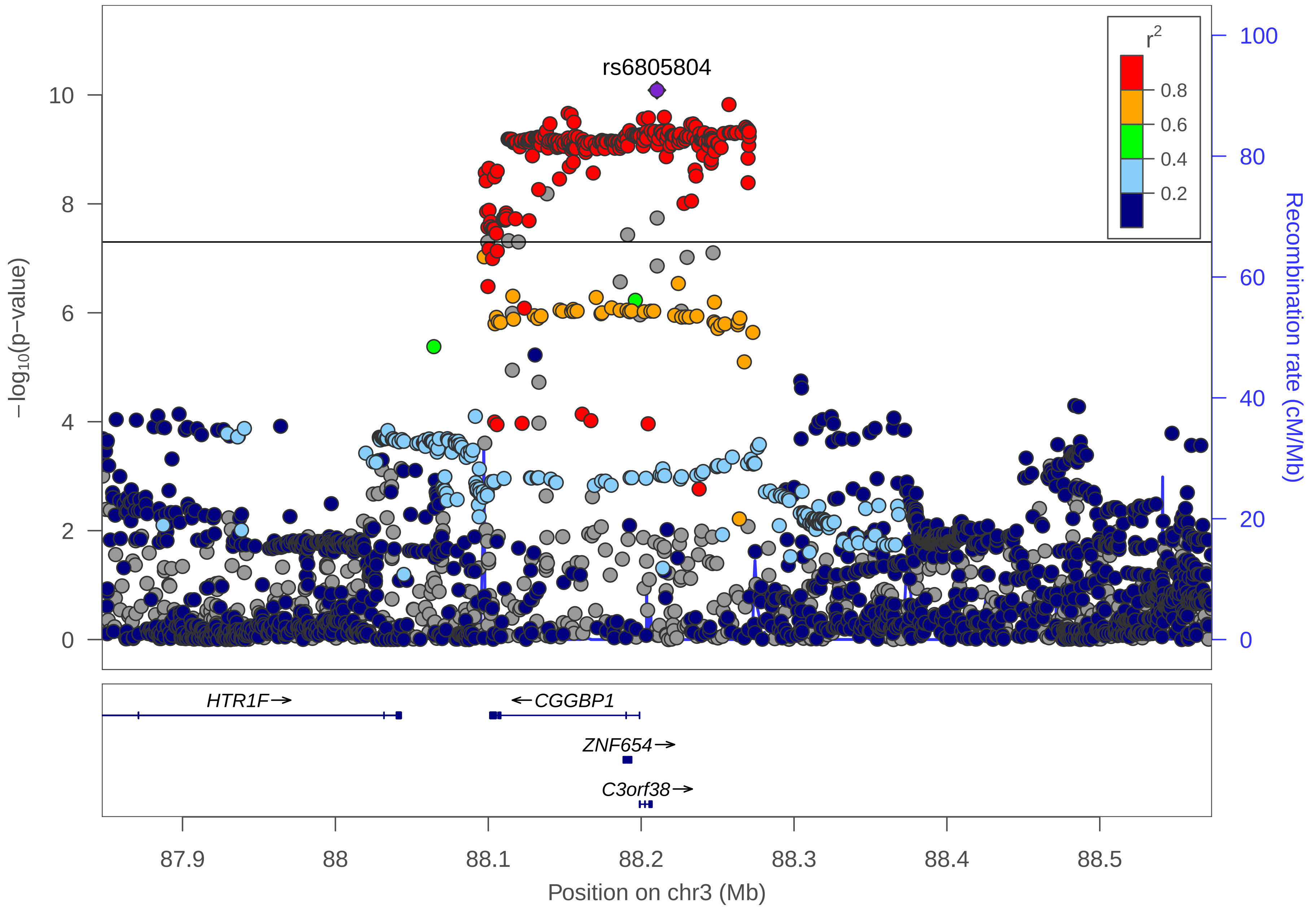
rs6805804
r2
0.8
0.6
0.4
0.2
10
8
6
4
2
0
100
80
60
40
20
0
−log10(p−value)
Recombination rate (cM/Mb)
HTR1F→
←CGGBP1
ZNF654→
C3orf38→
87.9
88
88.1
88.2
88.3
88.4
88.5
Position on chr3 (Mb)

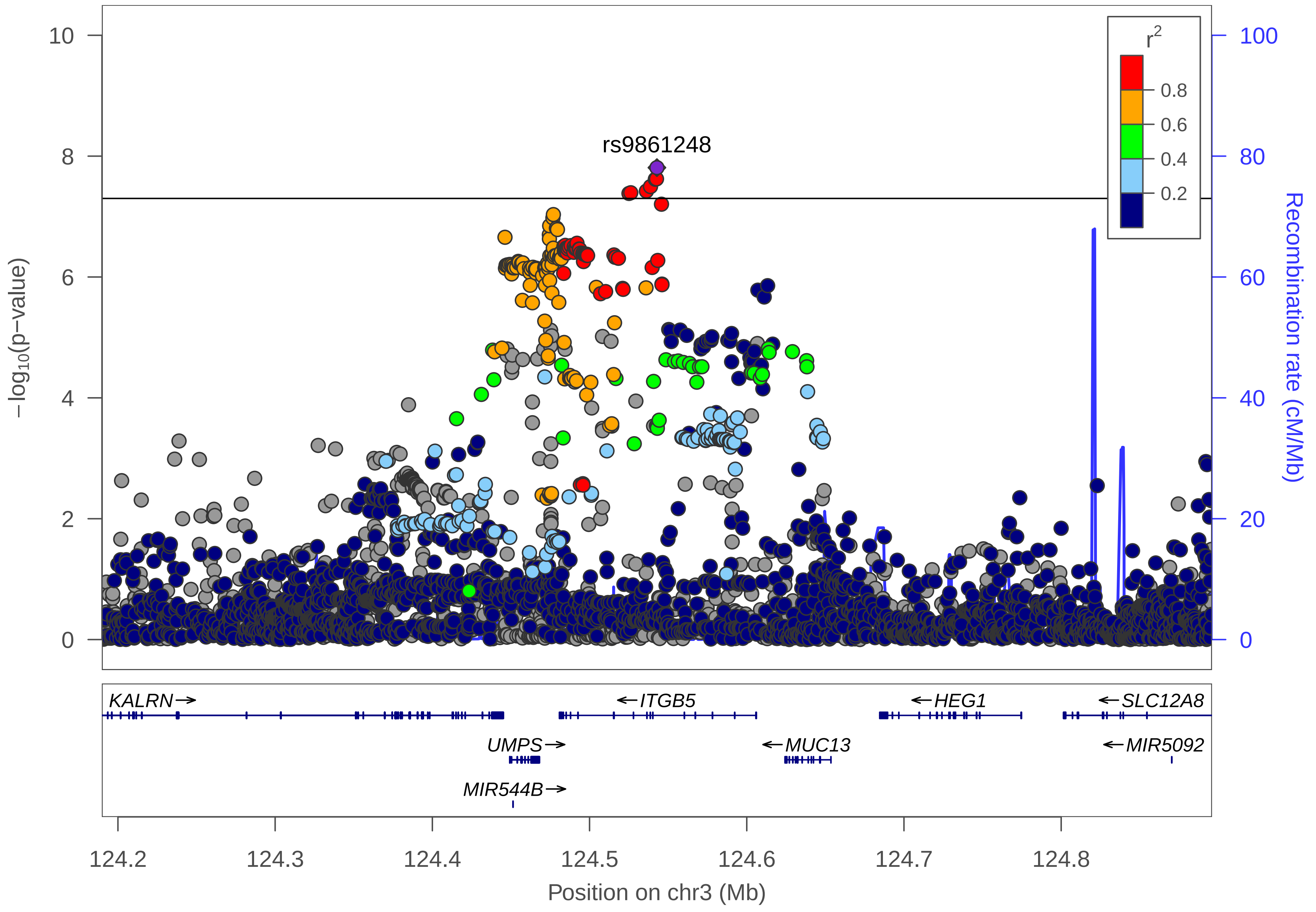

rs9861248
r2
0.8
0.6
0.4
0.2
−log10(p−value)
0
2
4
6
8
10
Recombination rate (cM/Mb)
0
20
40
60
80
100
KALRN→
←ITGB5
←HEG1
←SLC12A8
UMPS→
←MUC13
←MIR5092
MIR544B→
124.2
124.3
124.4
124.5
124.6
124.7
124.8
Position on chr3 (Mb)

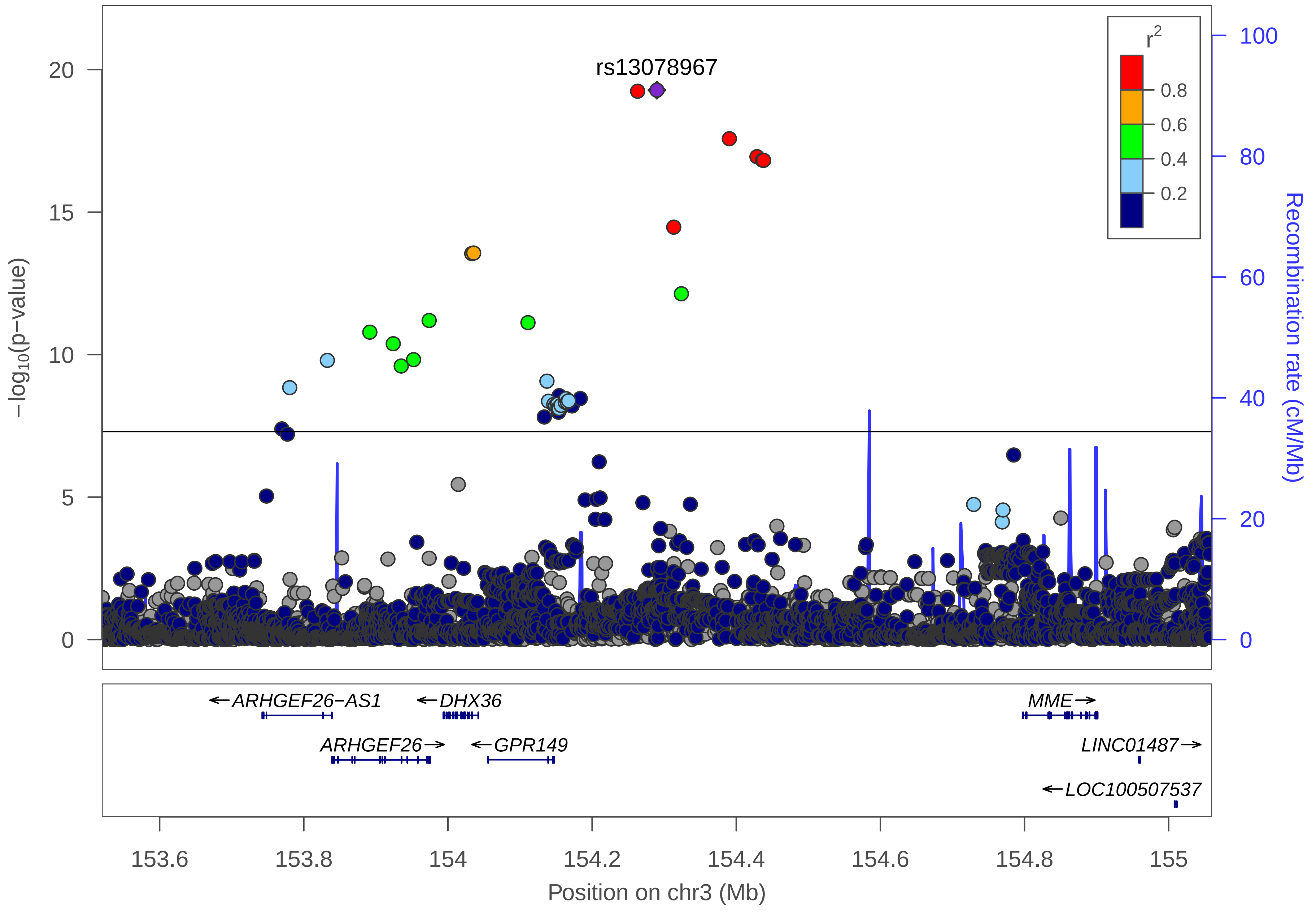

rs13078967
r²
0.8
0.6
0.4
0.2
−log10(p−value)
Recombination rate (cM/Mb)
ARHGEF26−AS1
DHX36
ARHGEF26
GPR149
MME
LINC01487
LOC100507537
Position on chr3 (Mb)

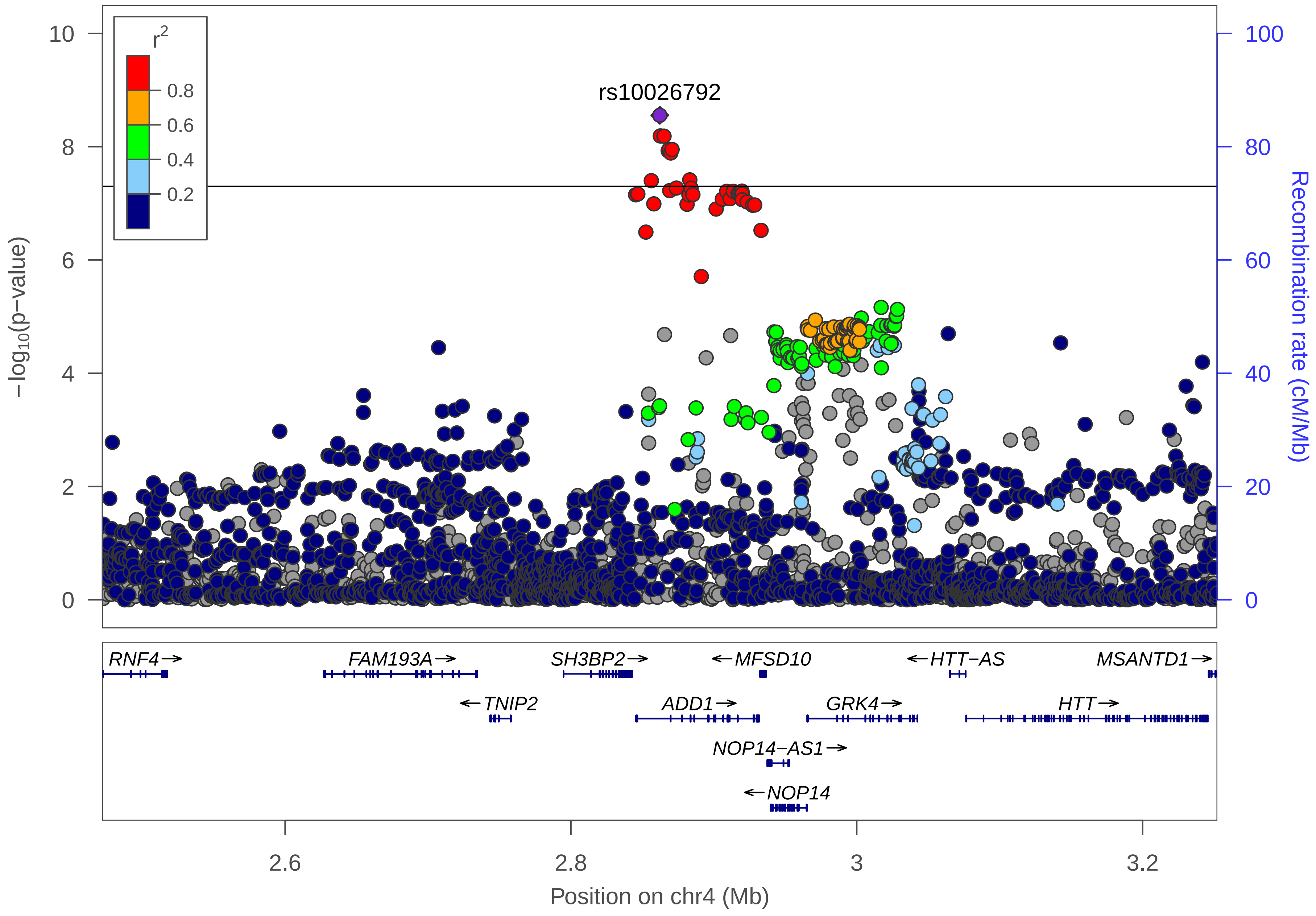

r2
0.8
0.6
0.4
0.2
rs10026792
−log10(p−value)
Recombination rate (cM/Mb)
10
8
6
4
2
0
100
80
60
40
20
0
RNF4→
FAM193A→
SH3BP2→
←MFSD10
←HTT−AS
MSANTD1→
←TNIP2
ADD1→
GRK4→
HTT→
NOP14−AS1→
←NOP14
2.6
2.8
3
3.2
Position on chr4 (Mb)

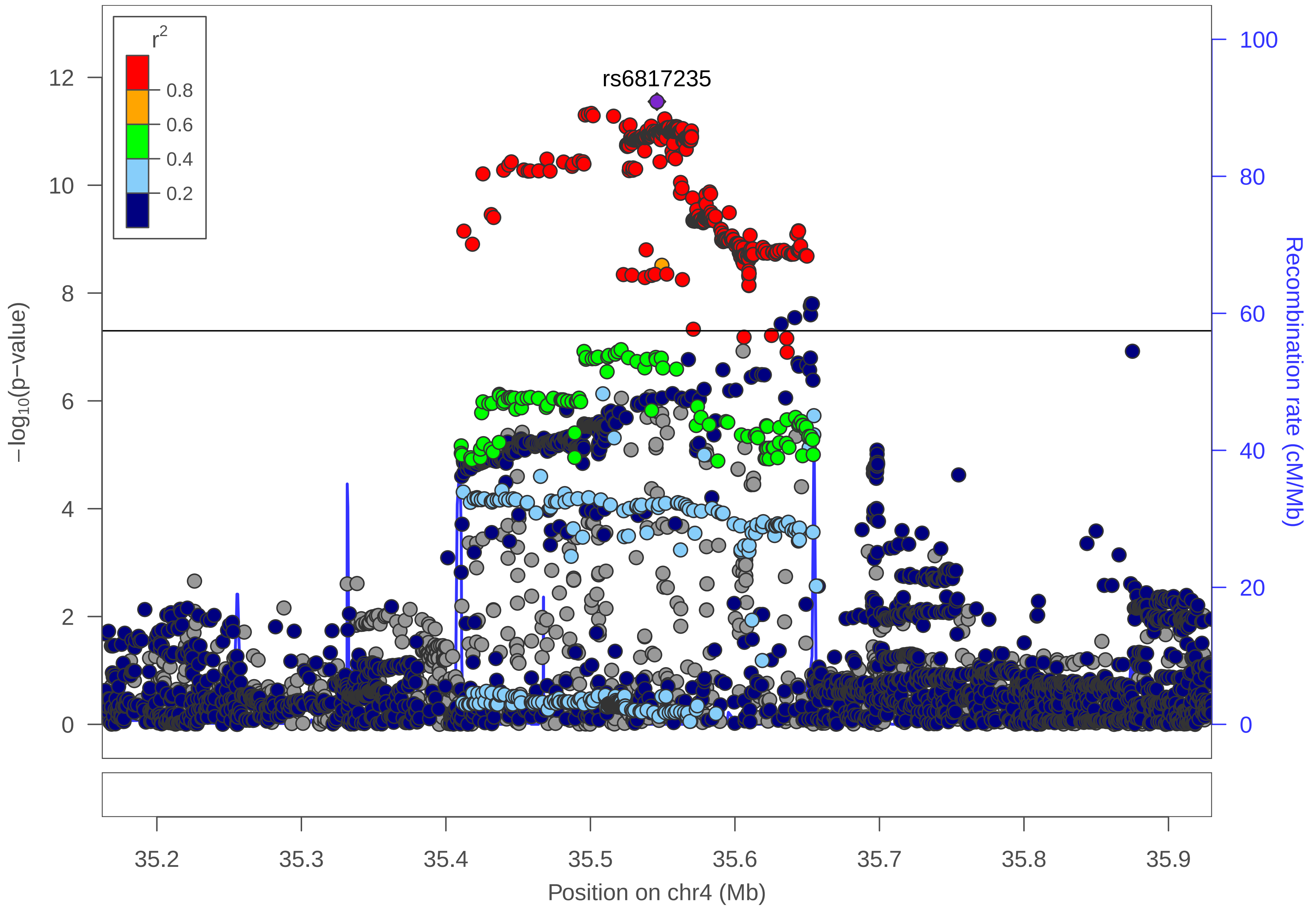

r2
0.8
0.6
0.4
0.2
rs6817235
−log10(p−value)
12
10
8
6
4
2
0
Recombination rate (cM/Mb)
100
80
60
40
20
0
35.2
35.3
35.4
35.5
35.6
35.7
35.8
35.9
Position on chr4 (Mb)

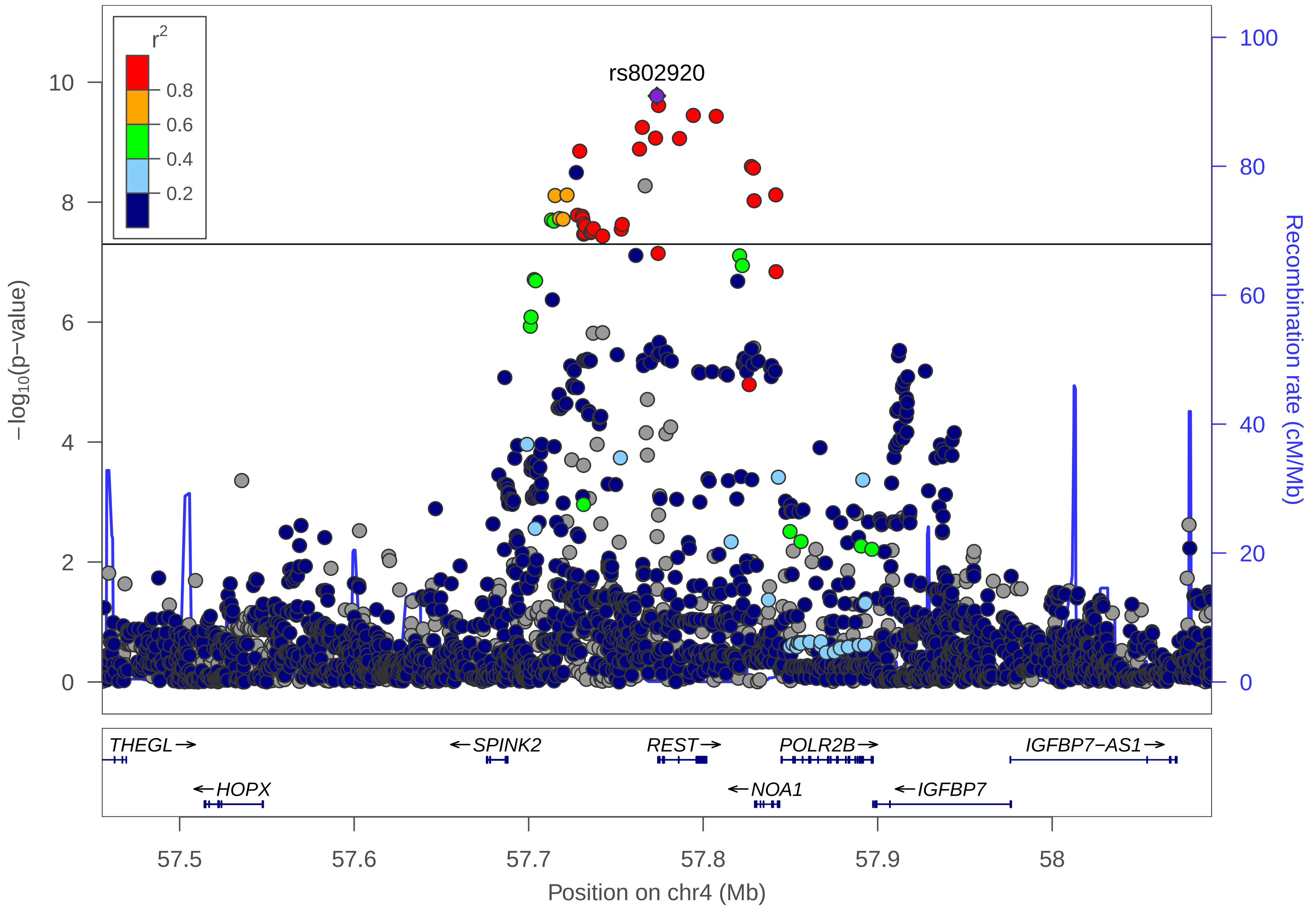

r2
0.8
0.6
0.4
0.2
rs802920
10
8
6
4
2
0
−log10(p−value)
100
80
60
40
20
0
Recombination rate (cM/Mb)
THEGL→
←SPINK2
REST→
POLR2B→
IGFBP7−AS1→
←HOPX
←NOA1
←IGFBP7
57.5
57.6
57.7
57.8
57.9
58
Position on chr4 (Mb)

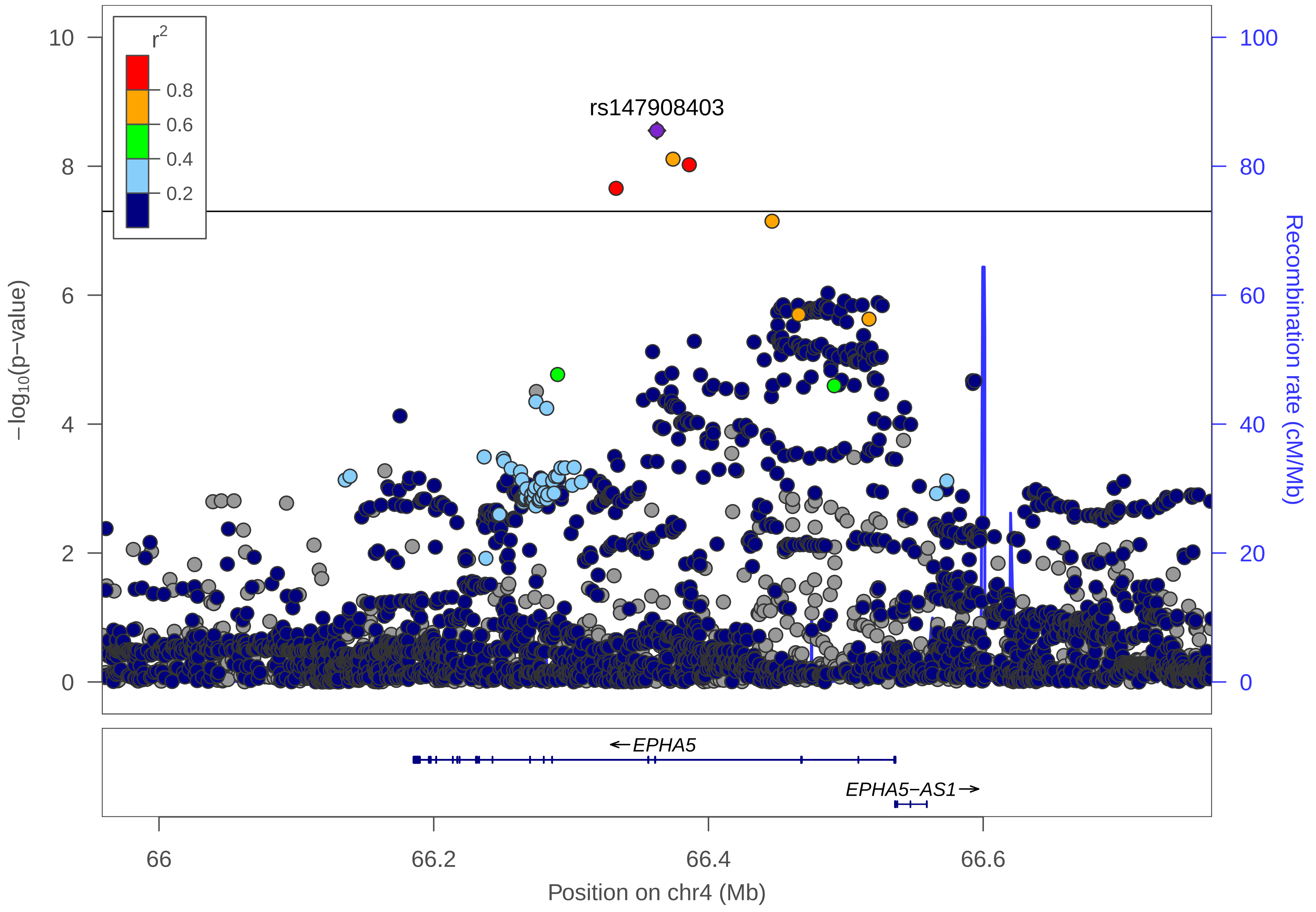

rs147908403
r^2
0.8
0.6
0.4
0.2
$-\log_{10}$(p−value)
Recombination rate (cM/Mb)
←EPHA5
EPHA5−AS1→
66
66.2
66.4
66.6
Position on chr4 (Mb)

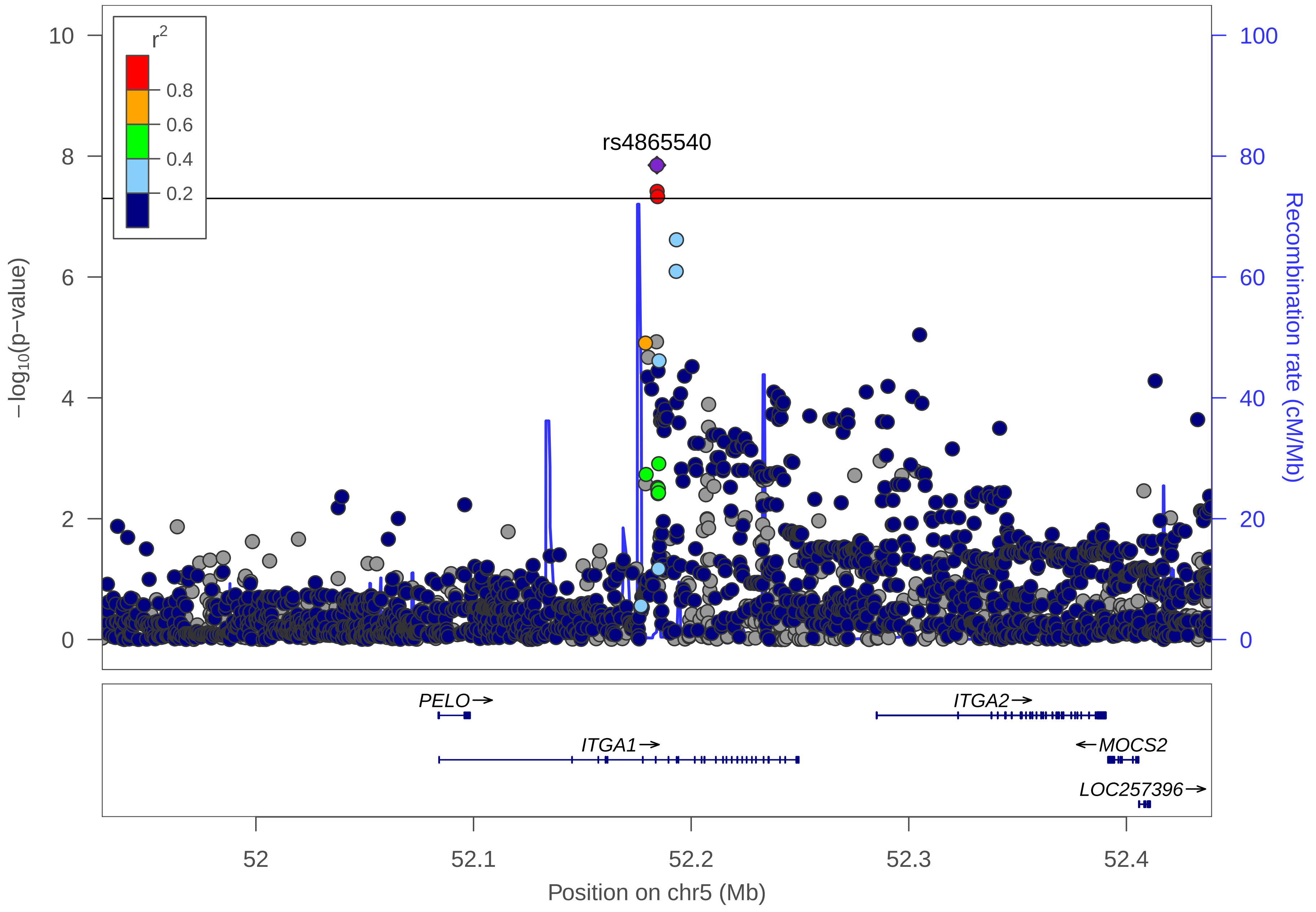
rs4865540
r2
0.8
0.6
0.4
0.2
10
8
6
4
2
0
−log10(p−value)
100
80
60
40
20
0
Recombination rate (cM/Mb)
PELO→
ITGA2→
ITGA1→
←MOCS2
LOC257396→
52
52.1
52.2
52.3
52.4
Position on chr5 (Mb)

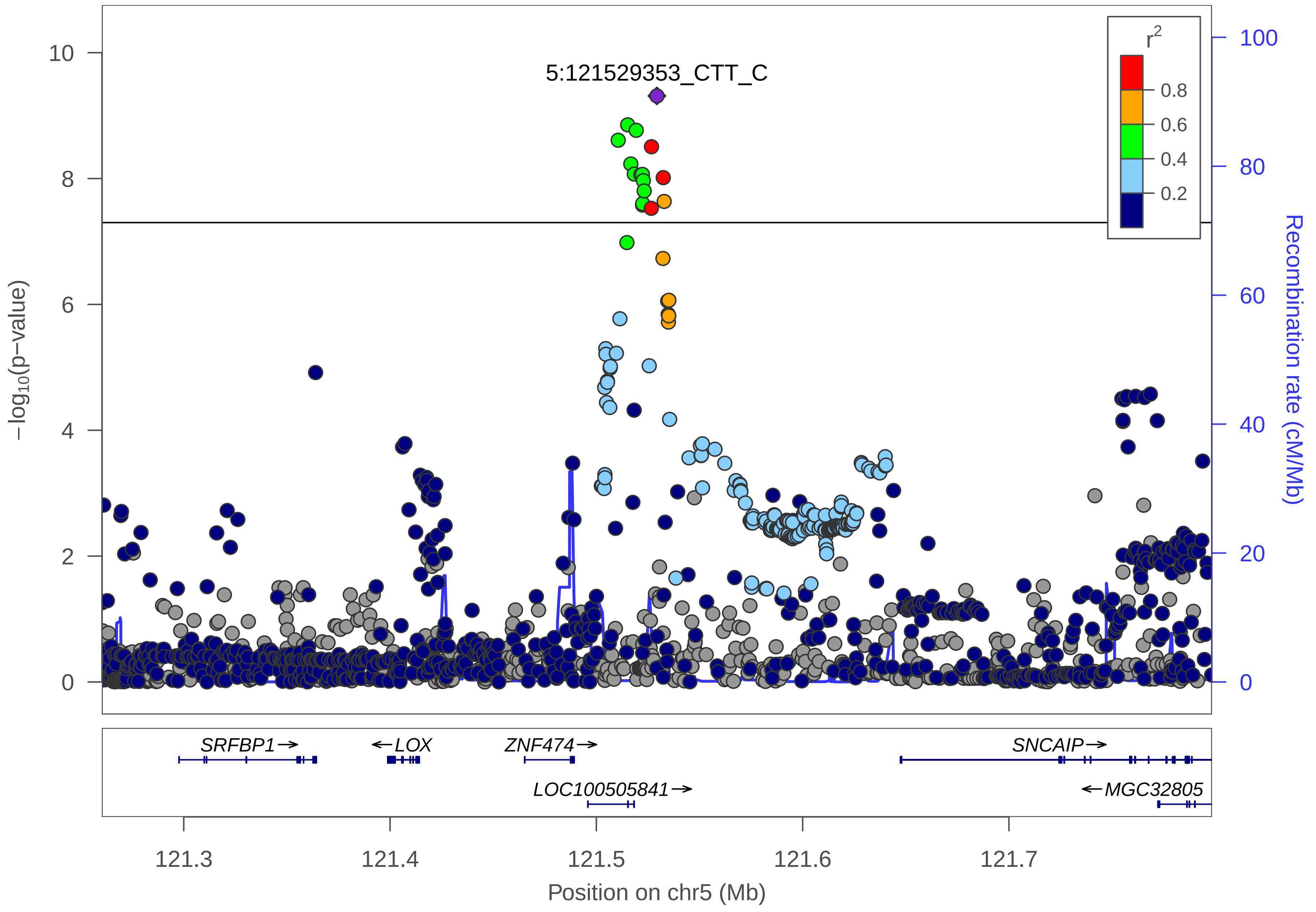

5:121529353_CTT_C
r2
0.8
0.6
0.4
0.2
10
8
6
4
2
0
−log10(p−value)
100
80
60
40
20
0
Recombination rate (cM/Mb)
SRFBP1→
←LOX
ZNF474→
SNCAIP→
LOC100505841→
←MGC32805
121.3
121.4
121.5
121.6
121.7
Position on chr5 (Mb)

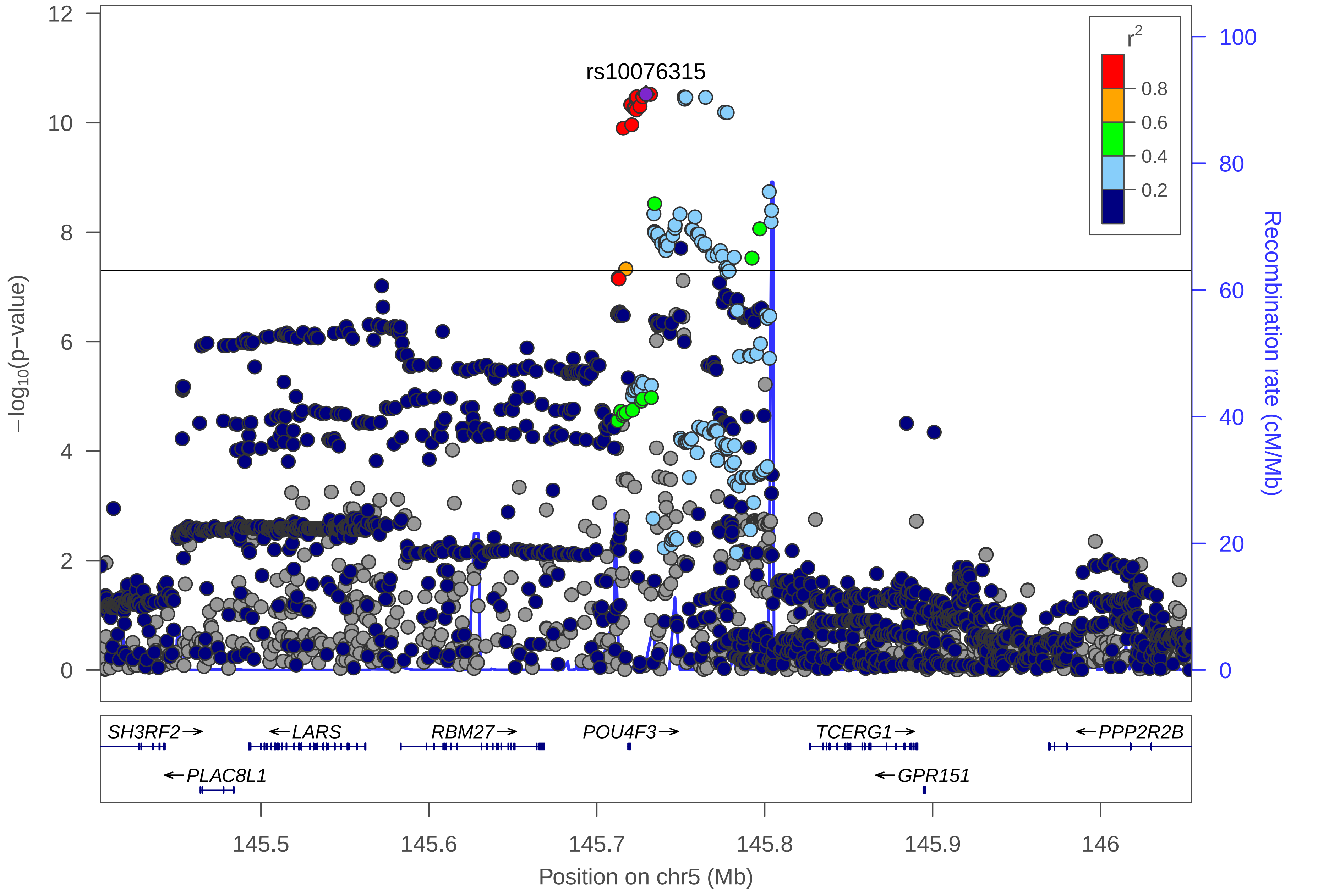

rs10076315
r²
0.8
0.6
0.4
0.2
−log10(p−value)
Recombination rate (cM/Mb)
12
10
8
6
4
2
0
100
80
60
40
20
0
SH3RF2→
←LARS
RBM27→
POU4F3→
TCERG1→
←PPP2R2B
←PLAC8L1
←GPR151
145.5
145.6
145.7
145.8
145.9
146
Position on chr5 (Mb)

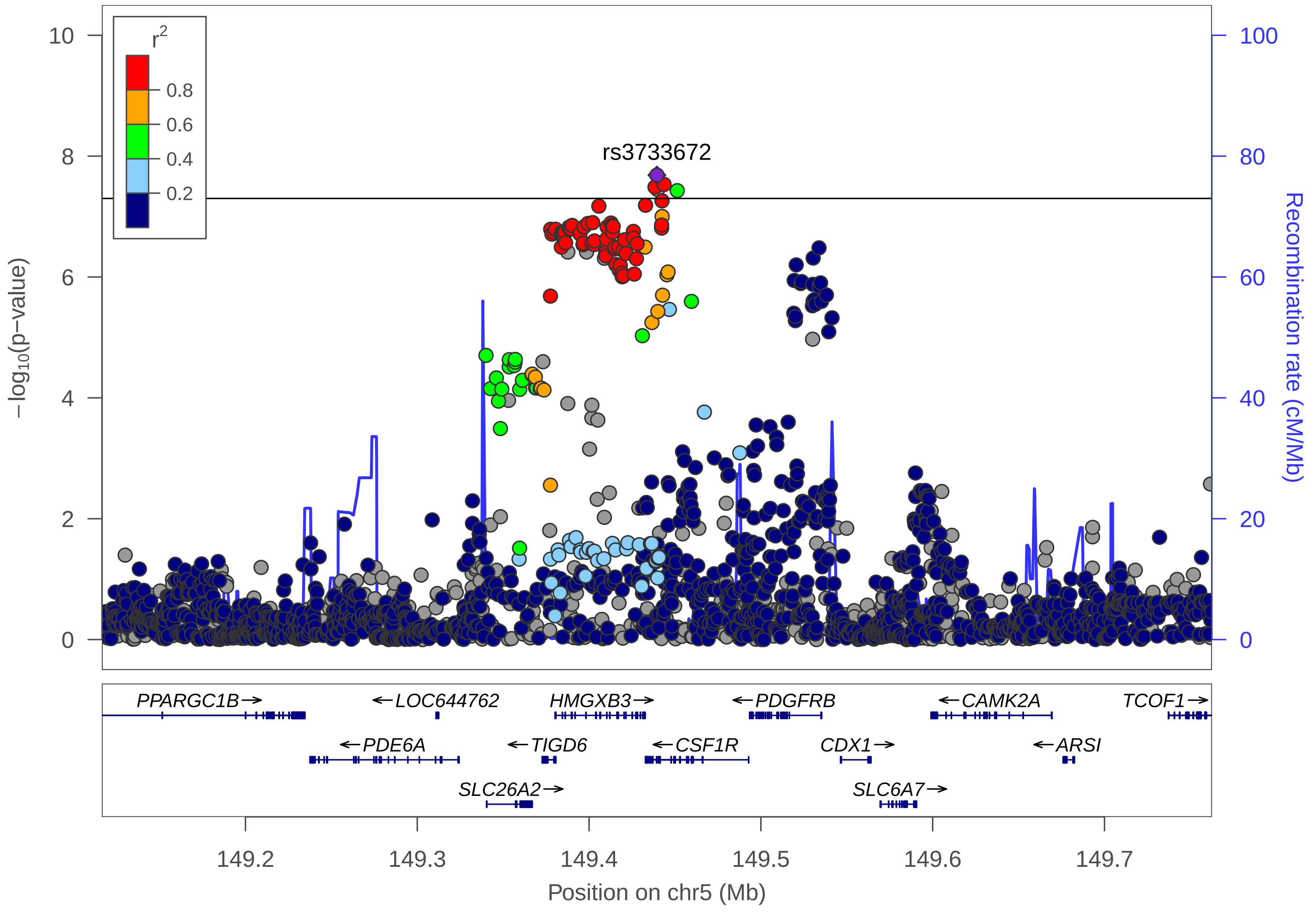

r2
0.8
0.6
0.4
0.2
rs3733672
−log10(p−value)
Recombination rate (cM/Mb)
10
8
6
4
2
0
100
80
60
40
20
0
PPARGC1B→
←LOC644762
HMGXB3→
←PDGFRB
←CAMK2A
TCOF1→
←PDE6A
←TIGD6
←CSF1R
CDX1→
←ARSI
SLC26A2→
SLC6A7→
149.2
149.3
149.4
149.5
149.6
149.7
Position on chr5 (Mb)

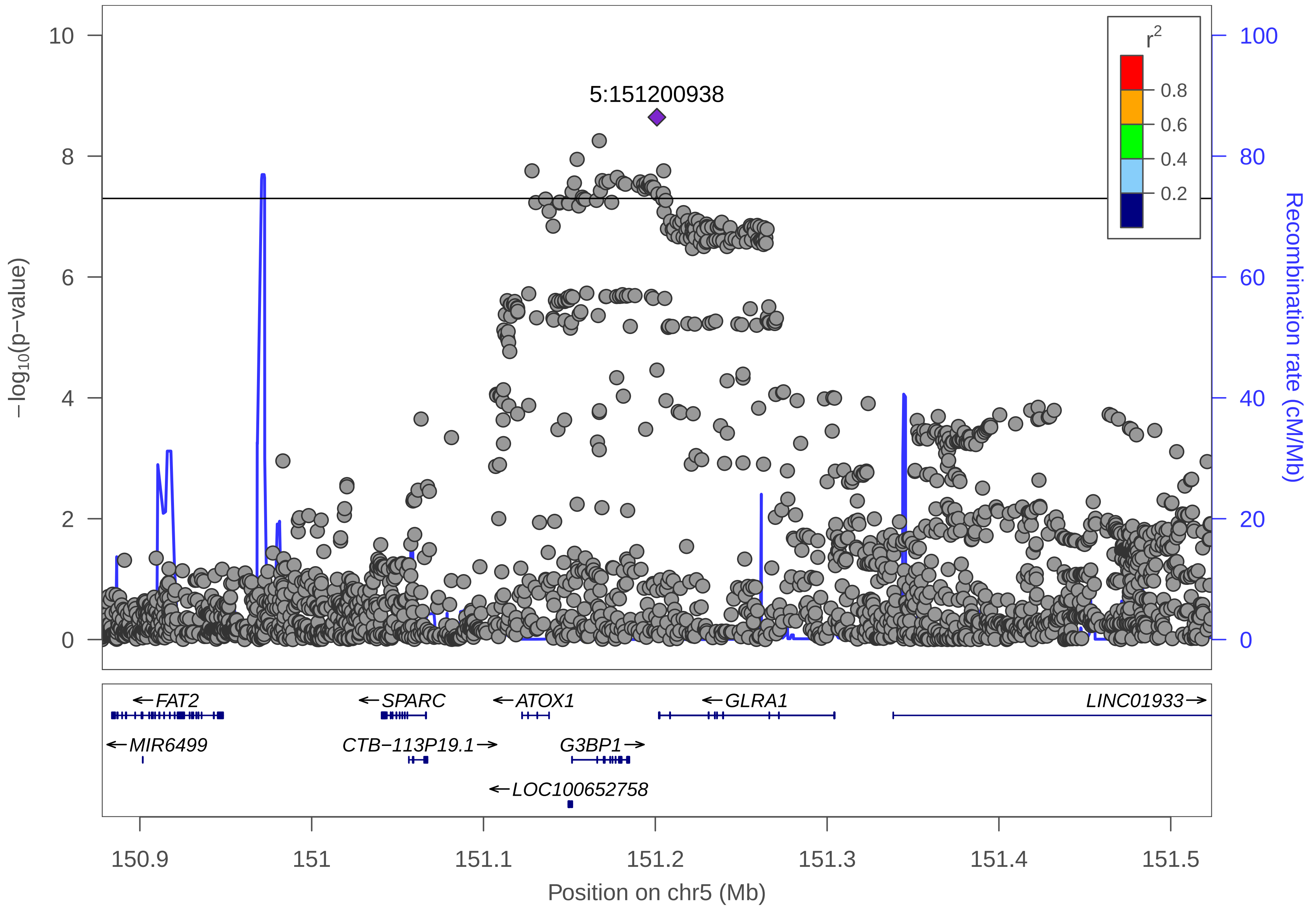
5:151200938
r²
0.8
0.6
0.4
0.2
−log10(p−value)
0
2
4
6
8
10
Recombination rate (cM/Mb)
0
20
40
60
80
100
←FAT2
←MIR6499
←SPARC
CTB−113P19.1→
←ATOX1
G3BP1→
←LOC100652758
←GLRA1
LINC01933→
150.9
151
151.1
151.2
151.3
151.4
151.5
Position on chr5 (Mb)

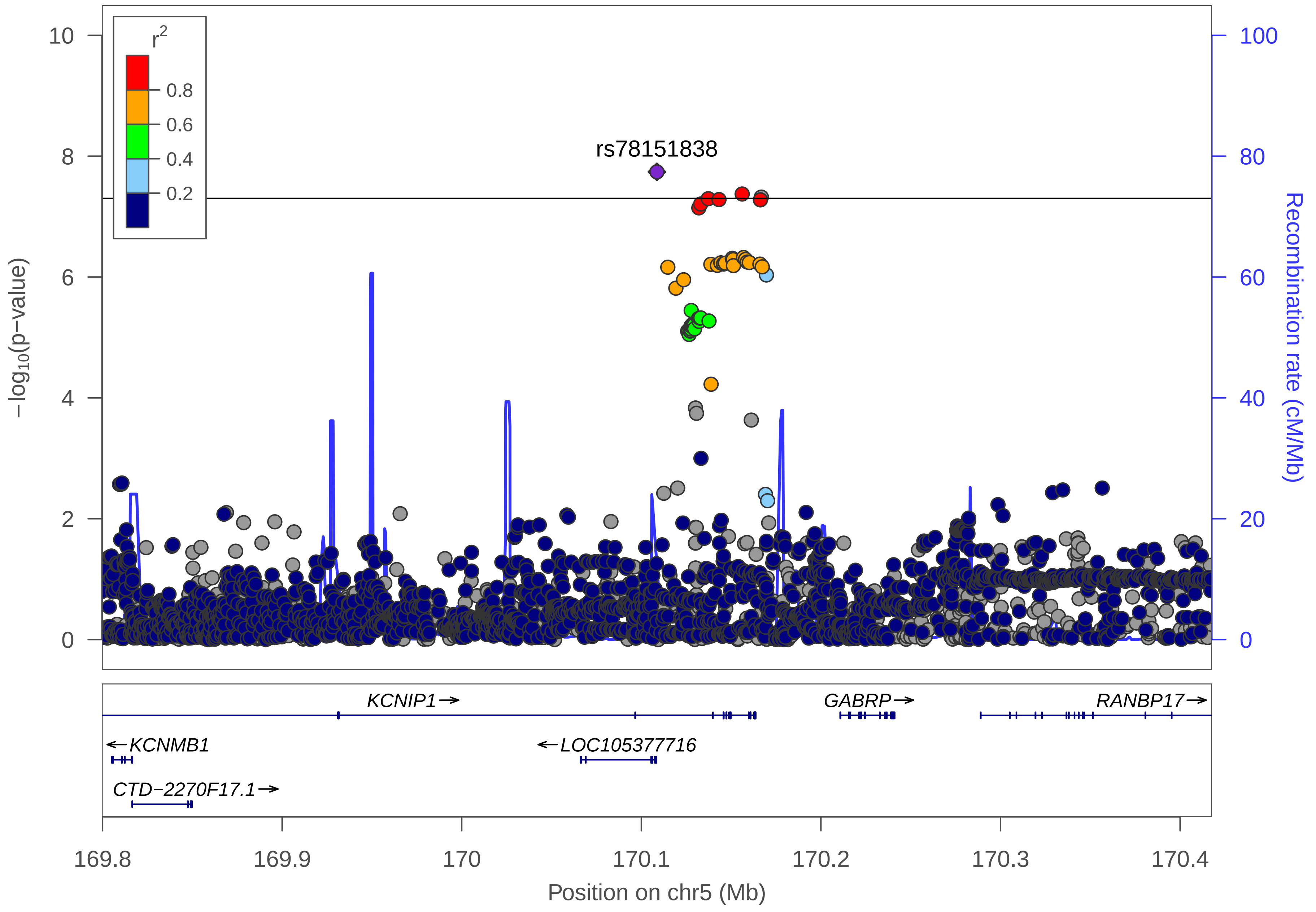

r2
0.8
0.6
0.4
0.2
rs78151838
−log10(p−value)
Recombination rate (cM/Mb)
KCNIP1→
GABRP→
RANBP17→
←KCNMB1
←LOC105377716
CTD−2270F17.1→
169.8
169.9
170
170.1
170.2
170.3
170.4
Position on chr5 (Mb)

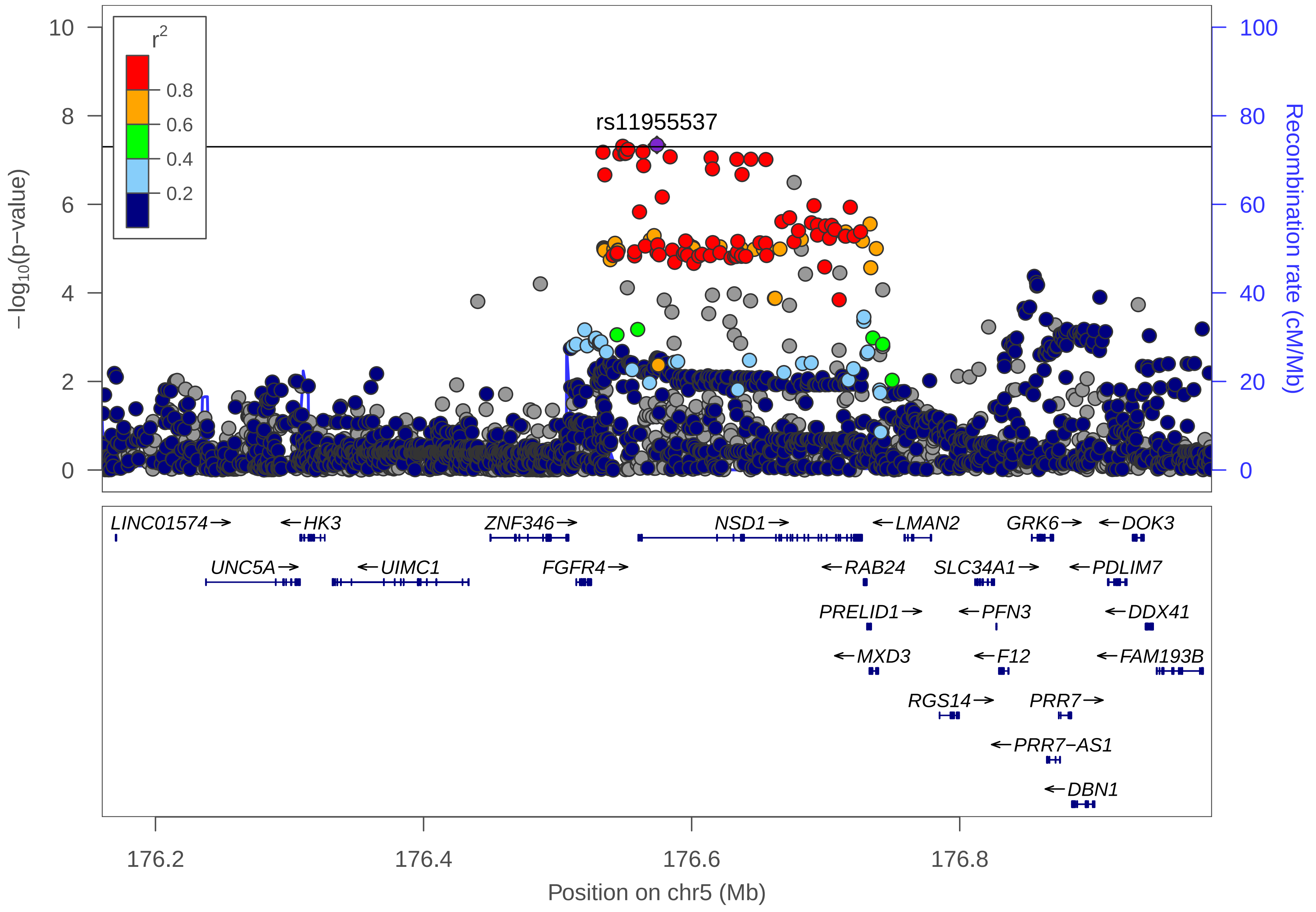

rs11955537
r2
0.8
0.6
0.4
0.2
−log10(p−value)
Recombination rate (cM/Mb)
0
2
4
6
8
10
0
20
40
60
80
100
LINC01574→
←HK3
ZNF346→
NSD1→
←LMAN2
GRK6→
←DOK3
UNC5A→
←UIMC1
FGFR4→
←RAB24
SLC34A1→
←PDLIM7
PRELID1→
←PFN3
←DDX41
←MXD3
←F12
←FAM193B
RGS14→
PRR7→
←PRR7−AS1
←DBN1
176.2
176.4
176.6
176.8
Position on chr5 (Mb)

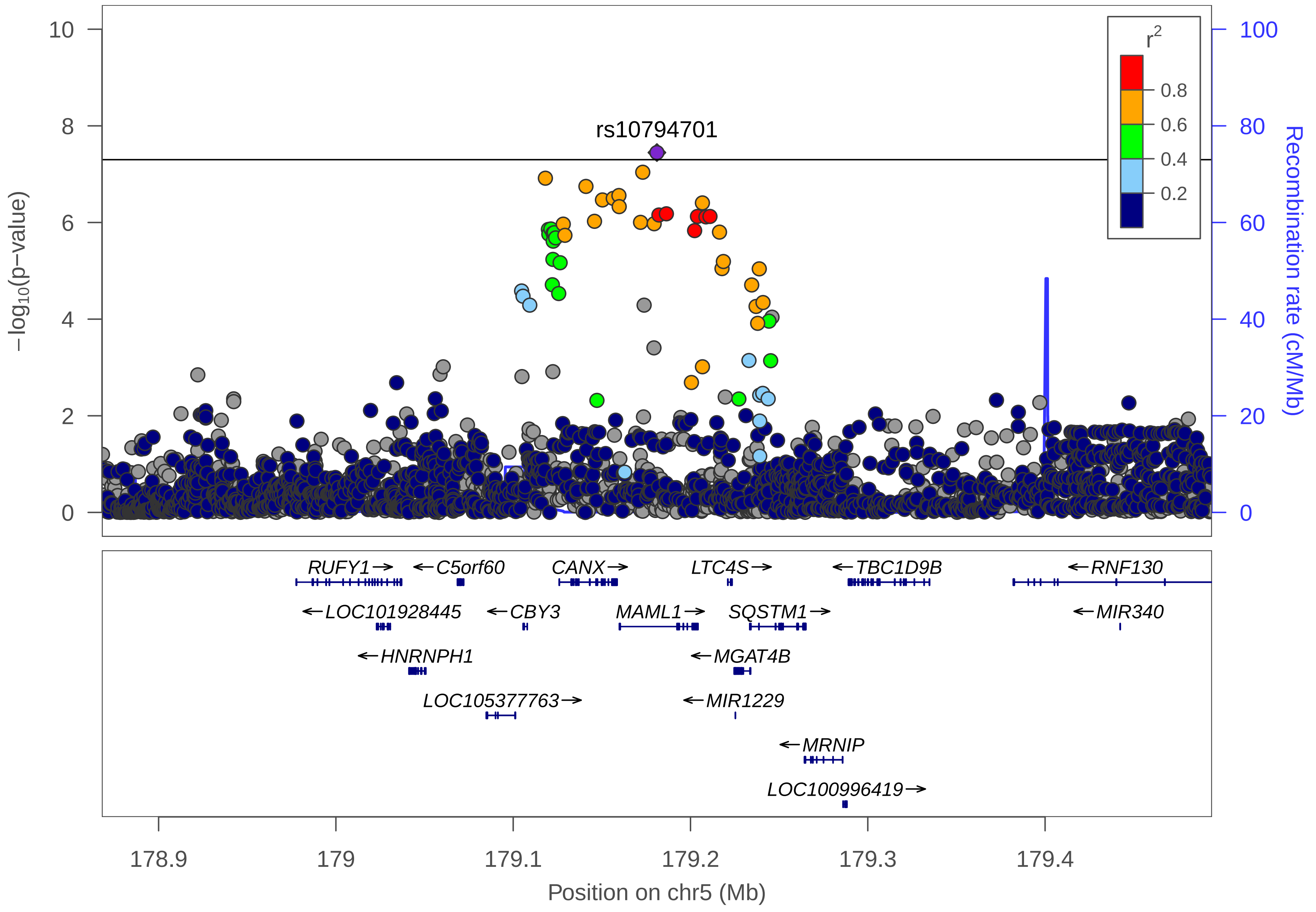

rs10794701
r2
0.8
0.6
0.4
0.2
−log10(p−value)
Recombination rate (cM/Mb)
0
2
4
6
8
10
0
20
40
60
80
100
RUFY1→
←C5orf60
CANX→
LTC4S→
←TBC1D9B
←RNF130
←LOC101928445
←CBY3
MAML1→
SQSTM1→
←MIR340
←HNRNPH1
←MGAT4B
LOC105377763→
←MIR1229
←MRNIP
LOC100996419→
178.9
179
179.1
179.2
179.3
179.4
Position on chr5 (Mb)

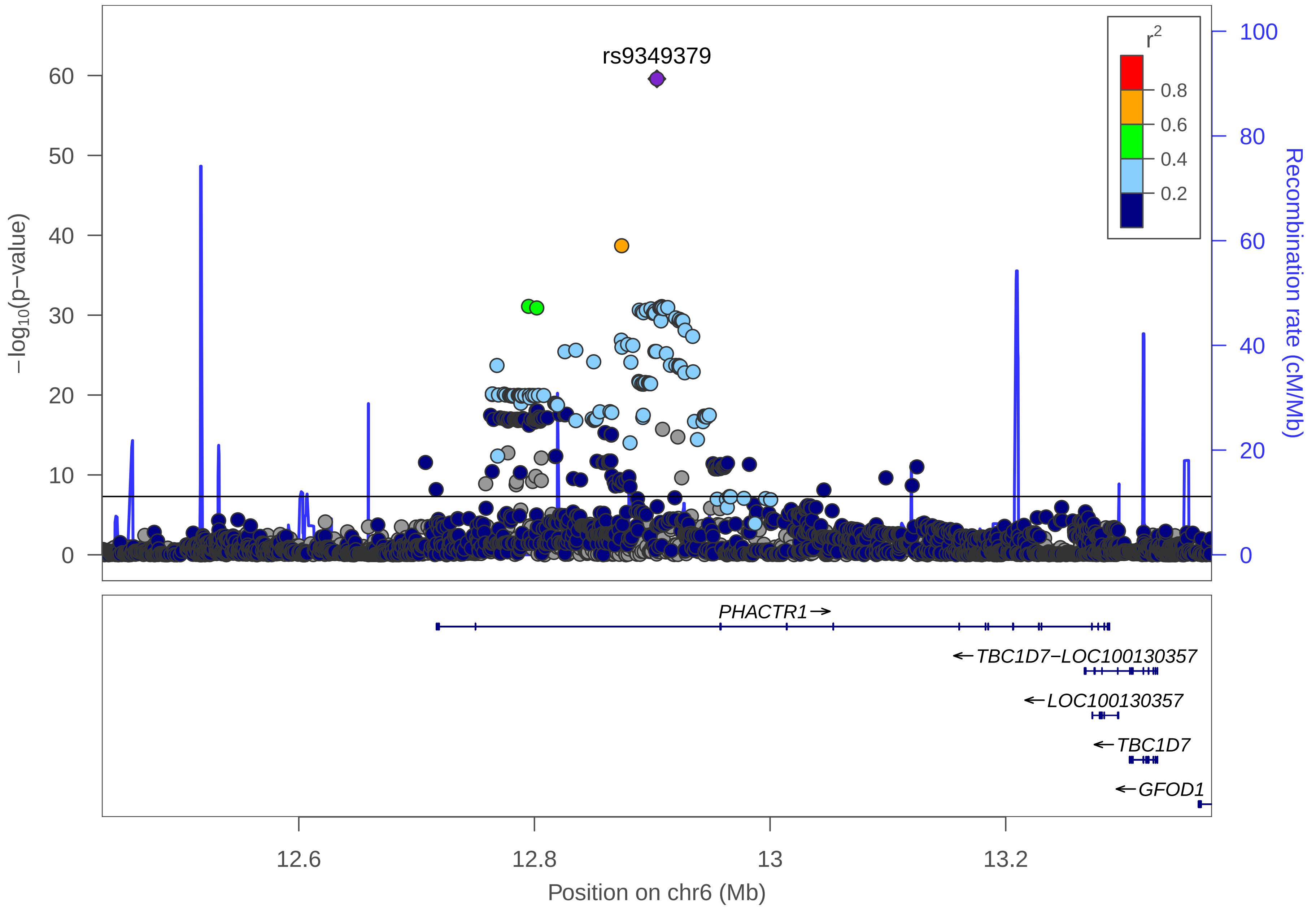

rs9349379
r²
0.8
0.6
0.4
0.2
−log10(p−value)
Recombination rate (cM/Mb)
0
10
20
30
40
50
60
0
20
40
60
80
100
PHACTR1→
←TBC1D7−LOC100130357
←LOC100130357
←TBC1D7
←GFOD1
12.6
12.8
13
13.2
Position on chr6 (Mb)

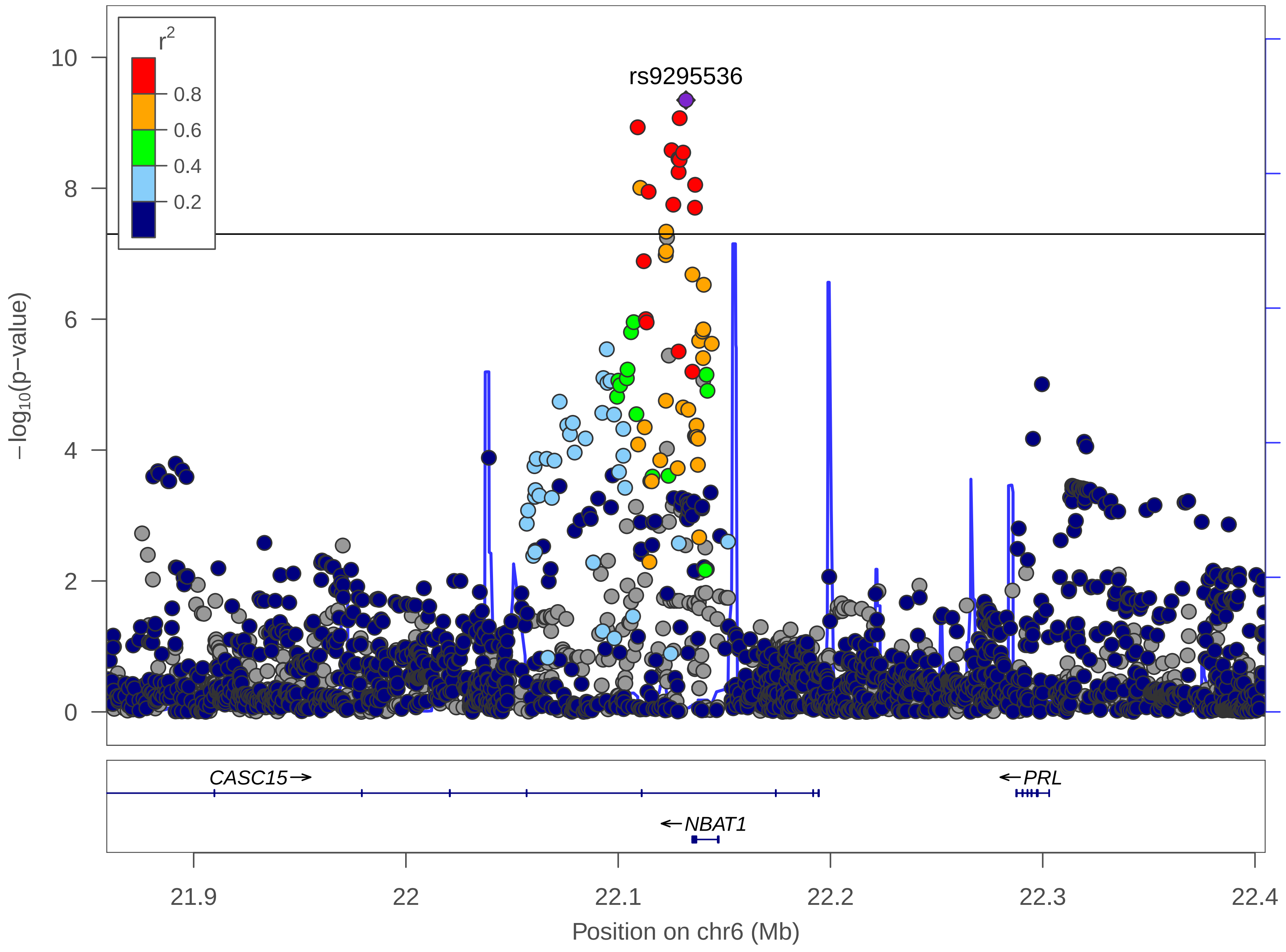

r2
0.8
0.6
0.4
0.2
rs9295536
10
8
6
4
2
0
−log10(p−value)
100
80
60
40
20
0
Recombination rate (cM/Mb)
CASC15→
←PRL
←NBAT1
21.9
22
22.1
22.2
22.3
22.4
Position on chr6 (Mb)

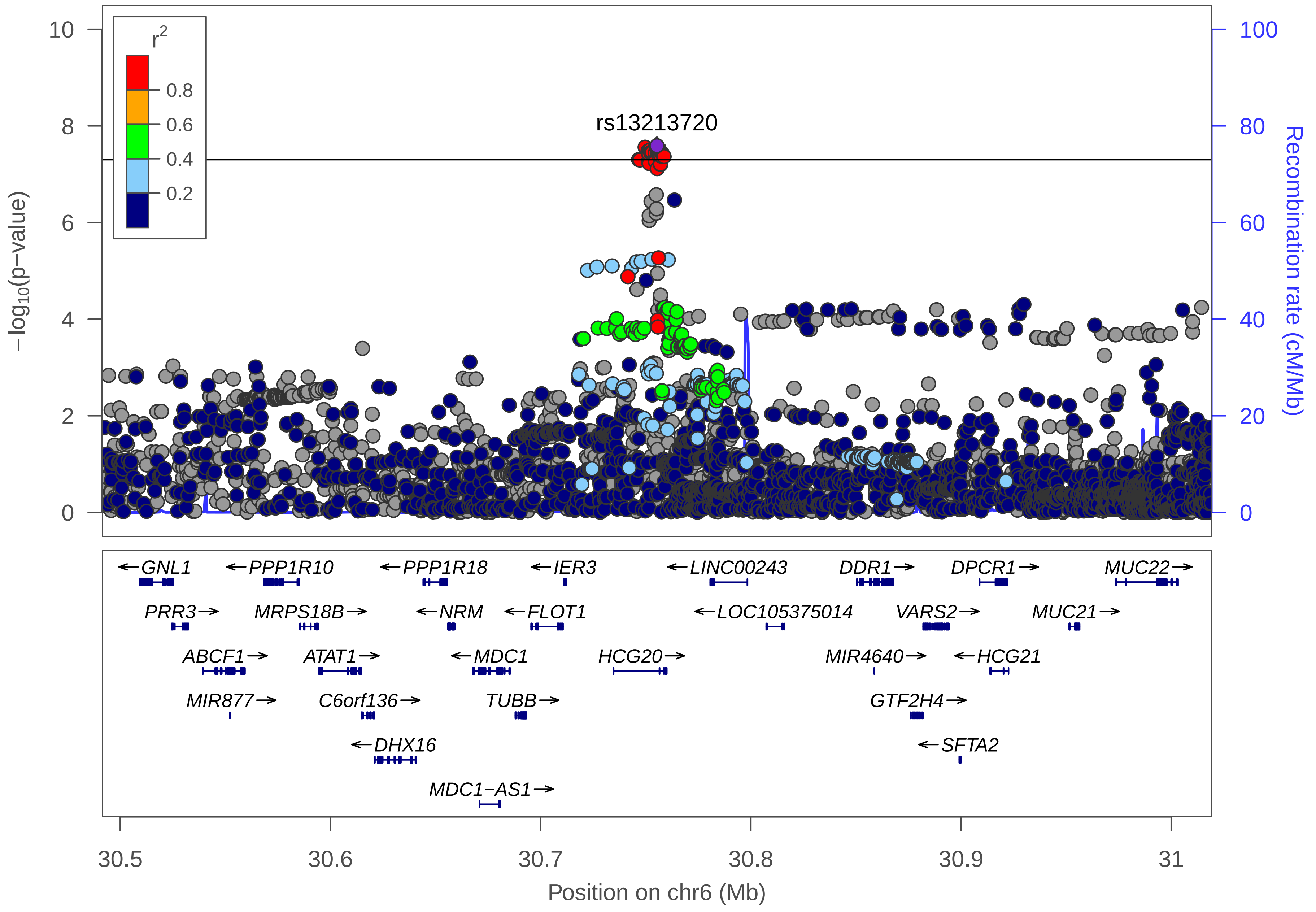
r2
0.8
0.6
0.4
0.2
rs13213720
−log10(p−value)
Recombination rate (cM/Mb)
10
8
6
4
2
0
100
80
60
40
20
0
←GNL1
←PPP1R10
←PPP1R18
←IER3
←LINC00243
DDR1→
DPCR1→
MUC22→
PRR3→
MRPS18B→
←NRM
←FLOT1
←LOC105375014
VARS2→
MUC21→
ABCF1→
ATAT1→
←MDC1
HCG20→
MIR4640→
←HCG21
MIR877→
C6orf136→
TUBB→
GTF2H4→
←DHX16
←SFTA2
MDC1−AS1→
30.5
30.6
30.7
30.8
30.9
31
Position on chr6 (Mb)

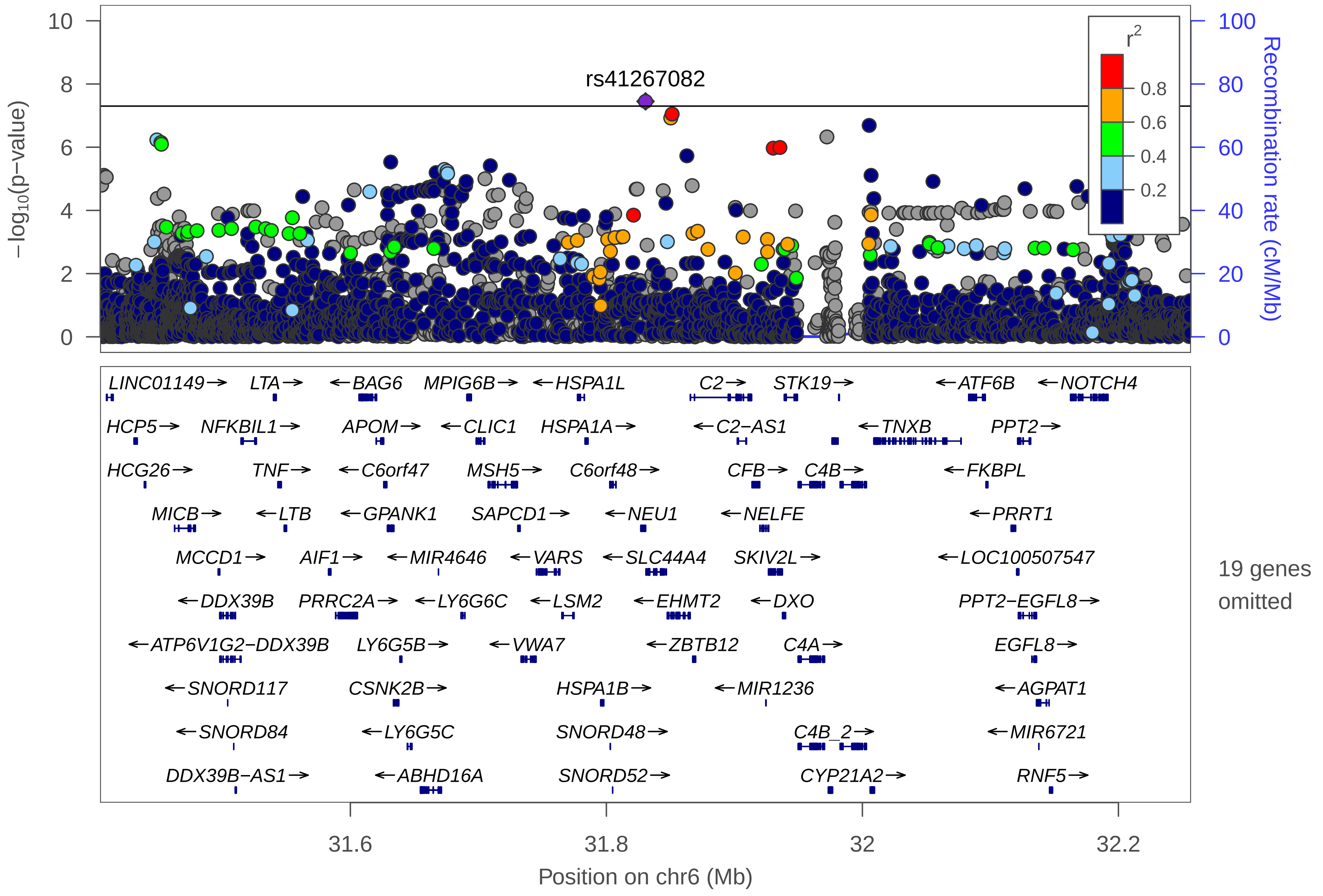

rs41267082
r2
0.8
0.6
0.4
0.2
-log10(p-value)
Recombination rate (cM/Mb)
LINC01149→ LTA→ ←BAG6 MPIG6B→ ←HSPA1L C2→ STK19→ ←ATF6B ←NOTCH4
HCP5→ NFKBIL1→ APOM→ ←CLIC1 HSPA1A→ ←C2-AS1 ←TNXB PPT2→
HCG26→ TNF→ ←C6orf47 MSH5→ C6orf48→ CFB→ C4B→ ←FKBPL
MICB→ ←LTB ←GPANK1 SAPCD1→ ←NEU1 ←NELFE ←PRRT1
MCCD1→ AIF1→ ←MIR4646 ←VARS ←SLC44A4 SKIV2L→ ←LOC100507547
←DDX39B PRRC2A→ ←LY6G6C ←LSM2 ←EHMT2 ←DXO PPT2-EGFL8→
←ATP6V1G2-DDX39B LY6G5B→ ←VWA7 ←ZBTB12 C4A→ EGFL8→
←SNORD117 CSNK2B→ HSPA1B→ ←MIR1236 ←AGPAT1
←SNORD84 ←LY6G5C SNORD48→ C4B_2→ ←MIR6721
DDX39B-AS1→ ←ABHD16A SNORD52→ CYP21A2→ RNF5→
19 genes omitted
31.6
31.8
32
32.2
Position on chr6 (Mb)

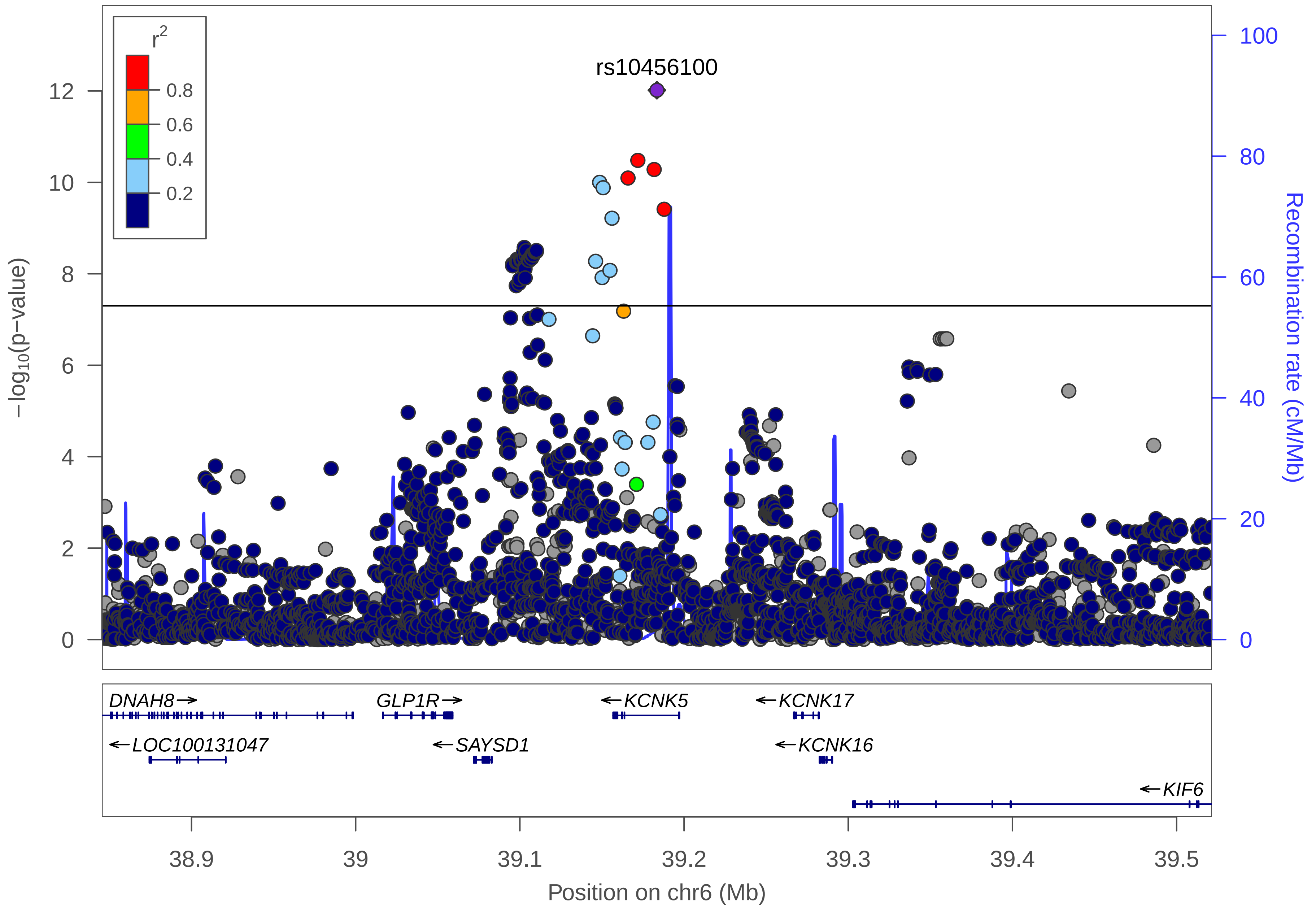
r2
0.8
0.6
0.4
0.2
rs10456100
−log10(p−value)
Recombination rate (cM/Mb)
0
2
4
6
8
10
12
20
40
60
80
100
DNAH8
GLP1R
KCNK5
KCNK17
LOC100131047
SAYSD1
KCNK16
KIF6
38.9
39
39.1
39.2
39.3
39.4
39.5
Position on chr6 (Mb)

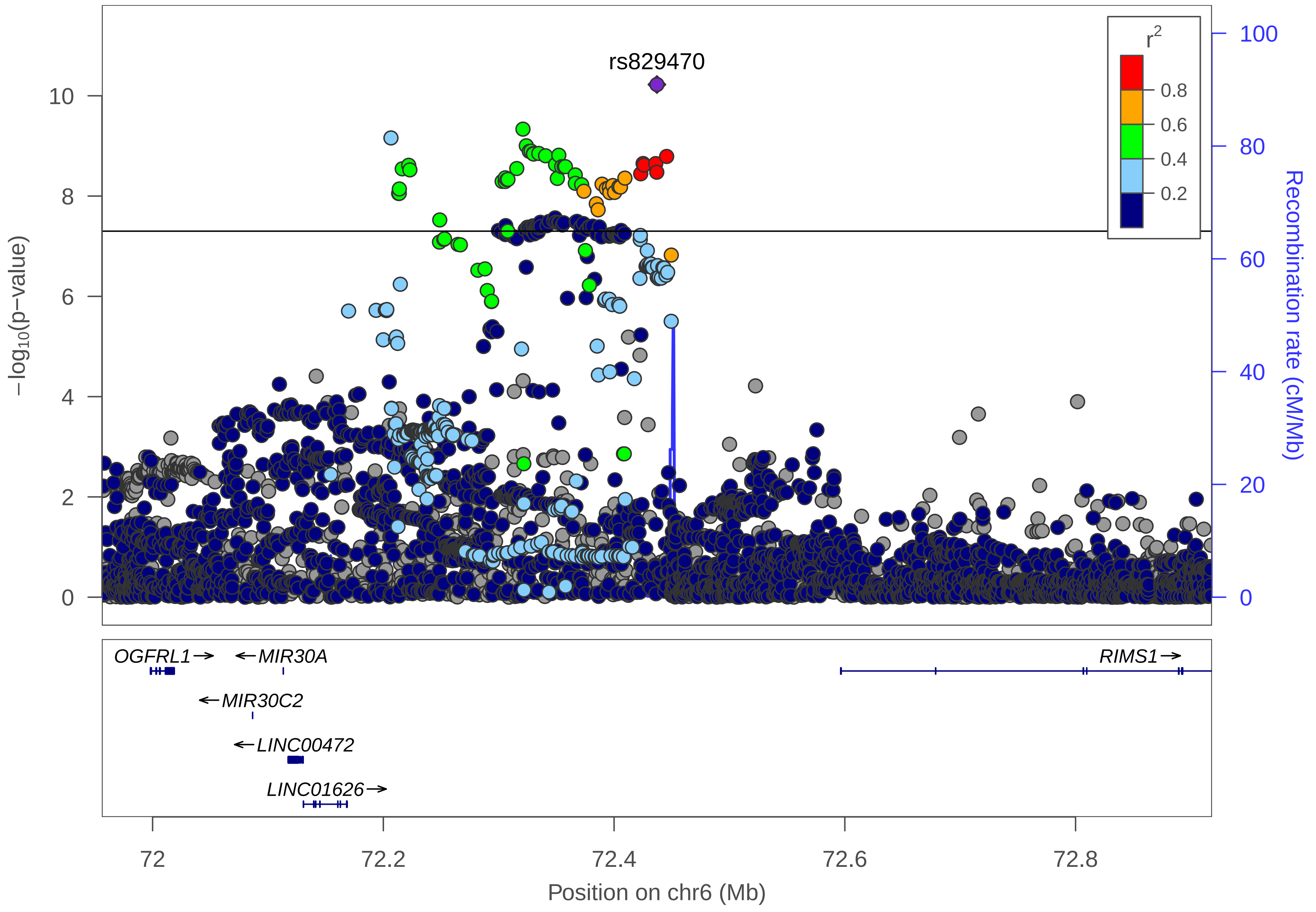

rs829470
r2
0.8
0.6
0.4
0.2
10
8
6
4
2
0
100
80
60
40
20
0
−log10(p−value)
Recombination rate (cM/Mb)
OGFRL1→
←MIR30A
←MIR30C2
←LINC00472
LINC01626→
RIMS1→
72
72.2
72.4
72.6
72.8
Position on chr6 (Mb)

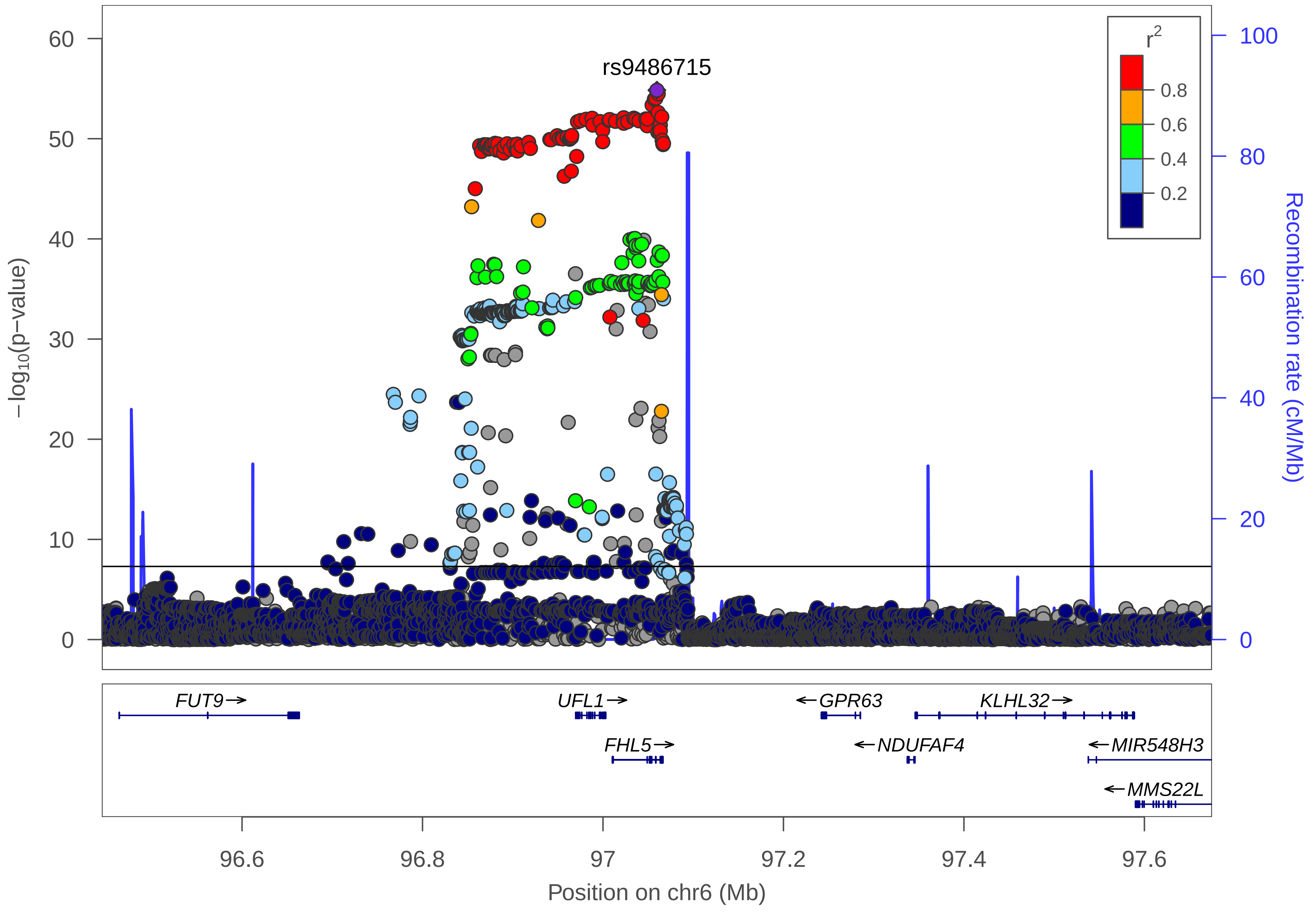
rs9486715
r2
0.8
0.6
0.4
0.2
−log10(p−value)
0
10
20
30
40
50
60
Recombination rate (cM/Mb)
0
20
40
60
80
100
FUT9→
UFL1→
←GPR63
KLHL32→
FHL5→
←NDUFAF4
←MIR548H3
←MMS22L
96.6
96.8
97
97.2
97.4
97.6
Position on chr6 (Mb)

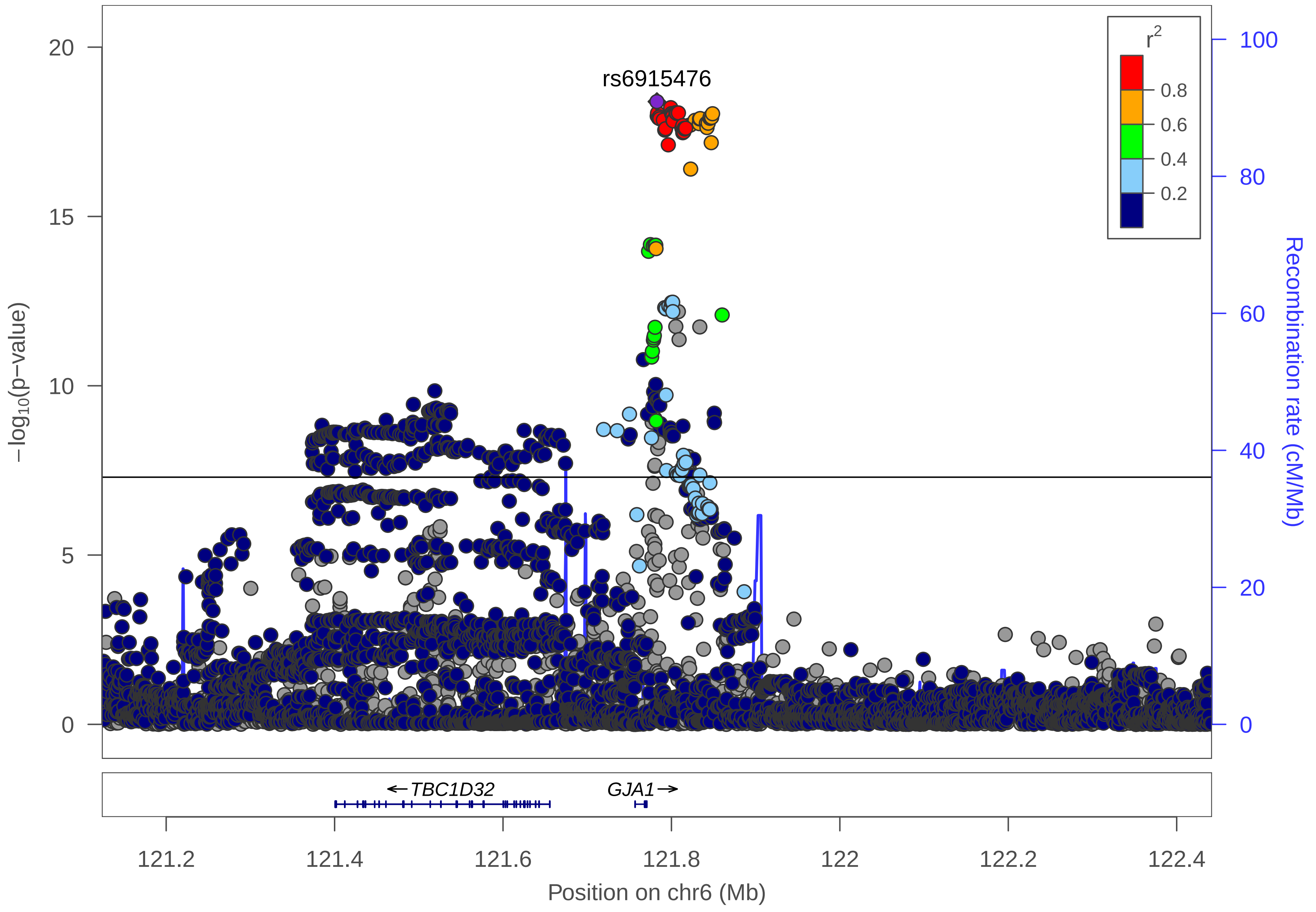

rs6915476
r^2
0.8
0.6
0.4
0.2
20
15
10
5
0
−log10(p−value)
100
80
60
40
20
0
Recombination rate (cM/Mb)
←TBC1D32
GJA1→
121.2
121.4
121.6
121.8
122
122.2
122.4
Position on chr6 (Mb)

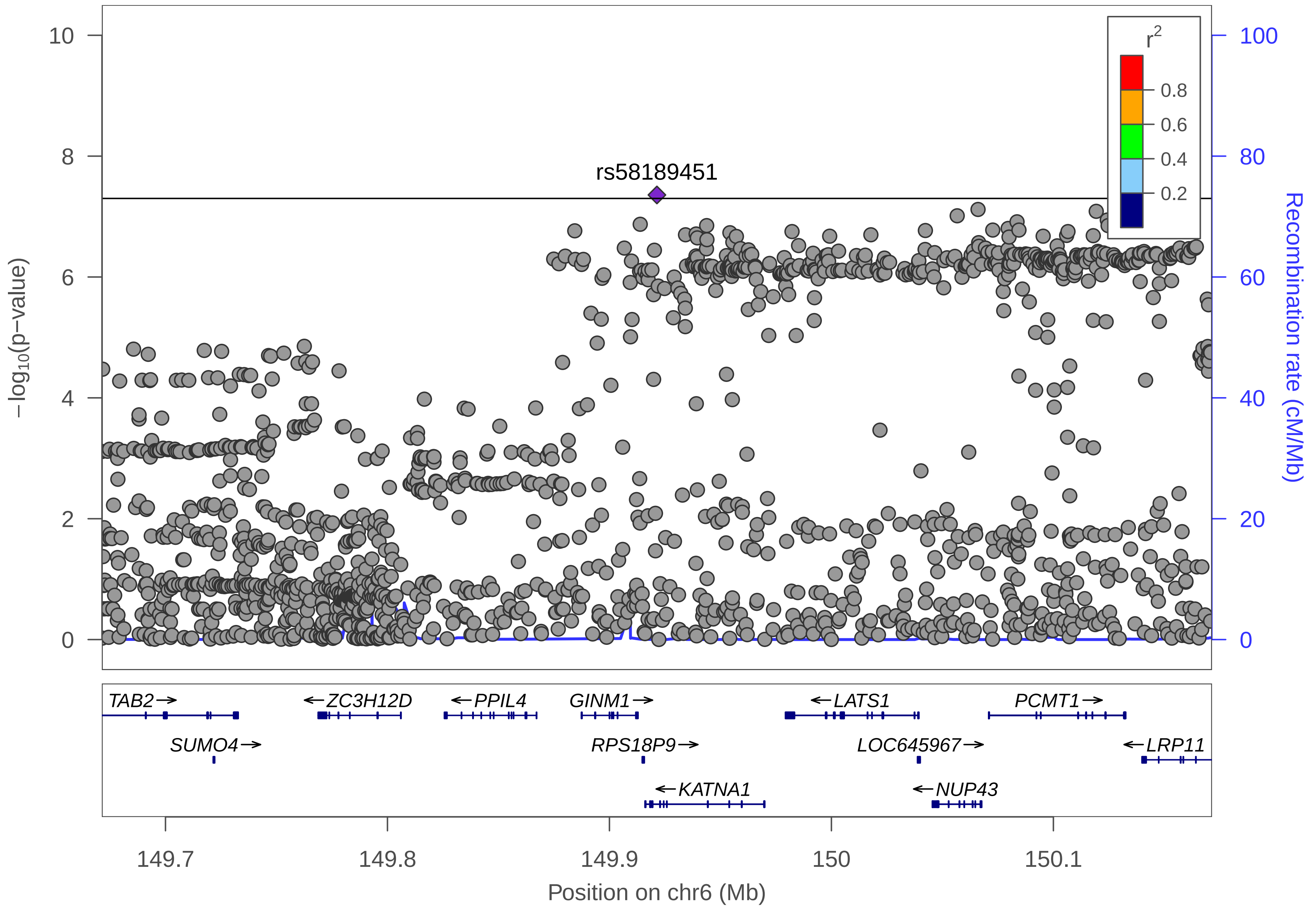

rs58189451
r2
0.8
0.6
0.4
0.2
−log10(p−value)
Recombination rate (cM/Mb)
10
8
6
4
2
0
100
80
60
40
20
0
TAB2→
←ZC3H12D
←PPIL4
GINM1→
←LATS1
PCMT1→
SUMO4→
RPS18P9→
LOC645967→
←LRP11
←KATNA1
←NUP43
149.7
149.8
149.9
150
150.1
Position on chr6 (Mb)

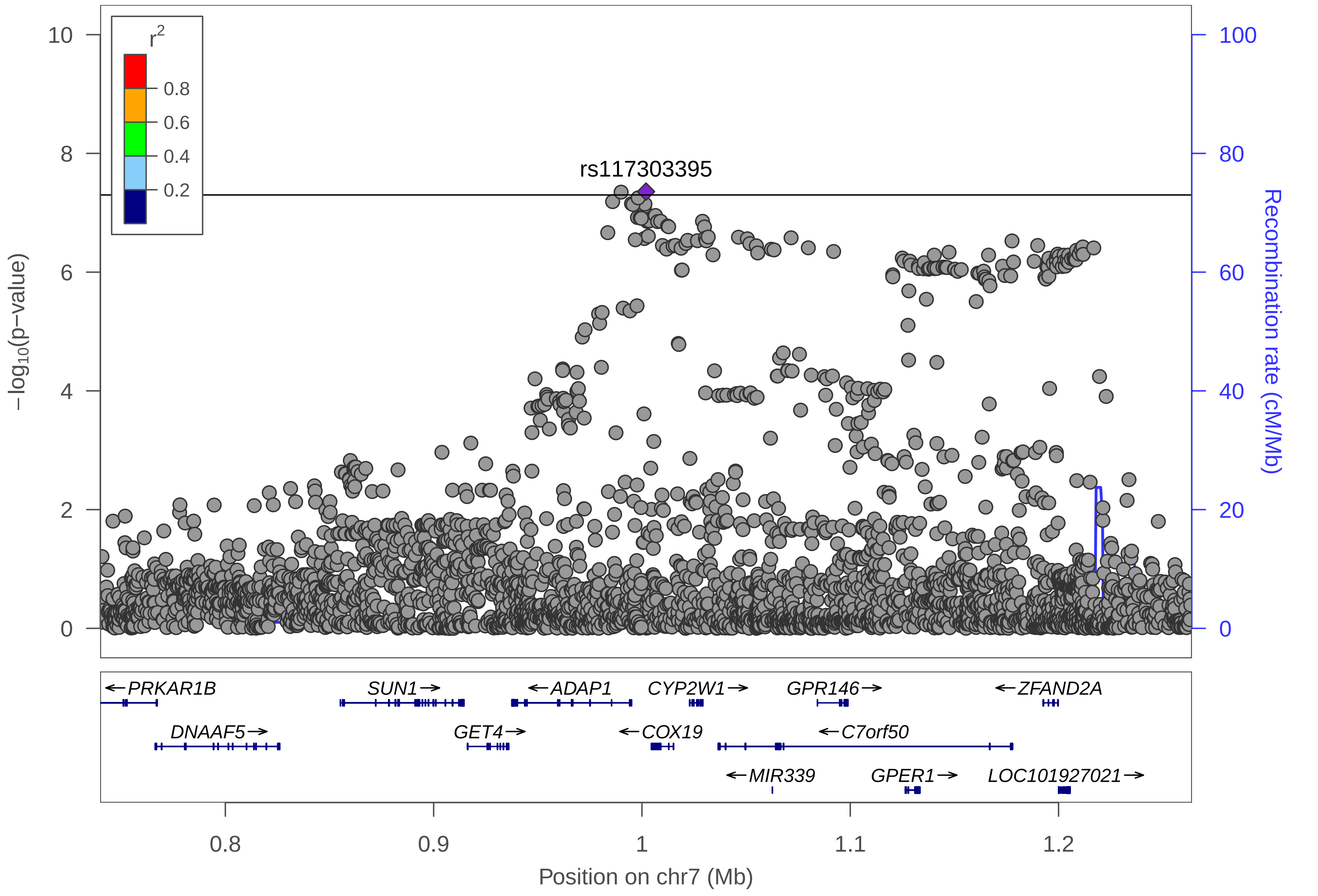

r2
0.8
0.6
0.4
0.2
rs117303395
−log10(p−value)
Recombination rate (cM/Mb)
10
8
6
4
2
0
100
80
60
40
20
0
PRKAR1B
SUN1
ADAP1
CYP2W1
GPR146
ZFAND2A
DNAAF5
GET4
COX19
C7orf50
MIR339
GPER1
LOC101927021
0.8
0.9
1
1.1
1.2
Position on chr7 (Mb)

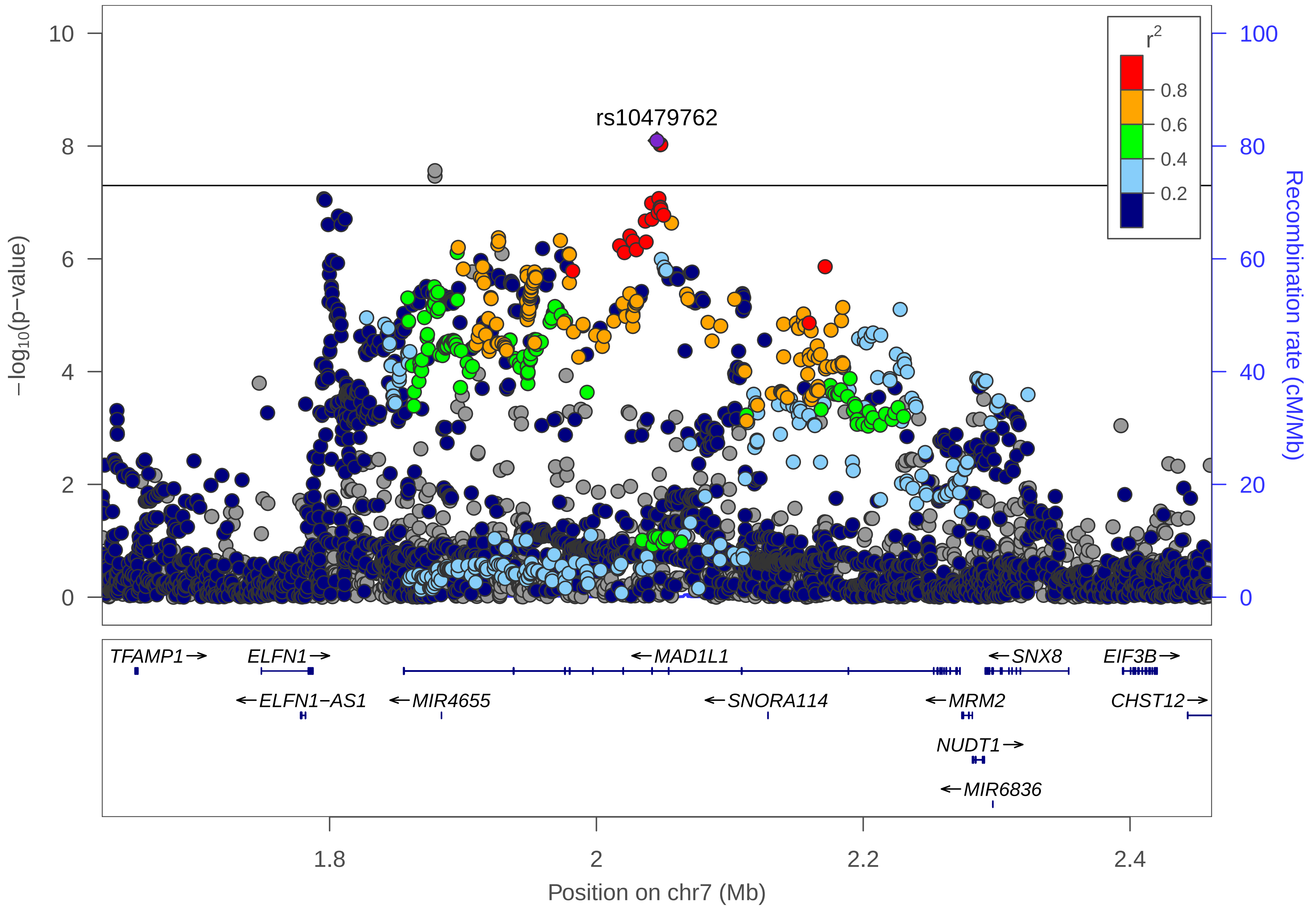

rs10479762
r2
0.8
0.6
0.4
0.2
−log10(p−value)
Recombination rate (cM/Mb)
10
8
6
4
2
0
100
80
60
40
20
0
TFAMP1→
ELFN1→
←MAD1L1
←SNX8
EIF3B→
←ELFN1−AS1
←MIR4655
←SNORA114
←MRM2
CHST12→
NUDT1→
←MIR6836
1.8
2
2.2
2.4
Position on chr7 (Mb)

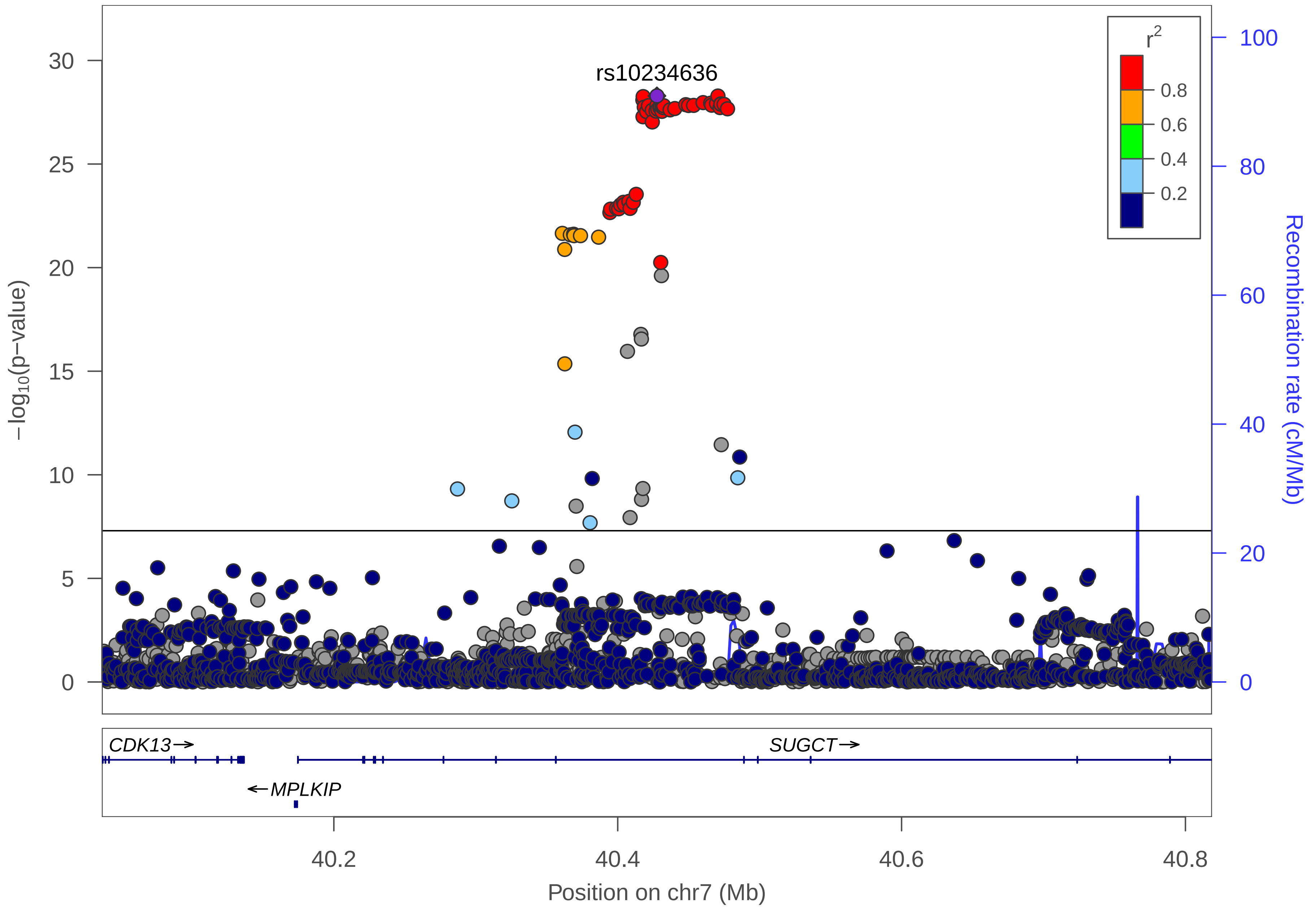
rs10234636
r²
0.8
0.6
0.4
0.2
−log10(p−value)
Recombination rate (cM/Mb)
CDK13→
SUGCT→
←MPLKIP
Position on chr7 (Mb)
40.2
40.4
40.6
40.8
0
5
10
15
20
25
30
20
40
60
80
100

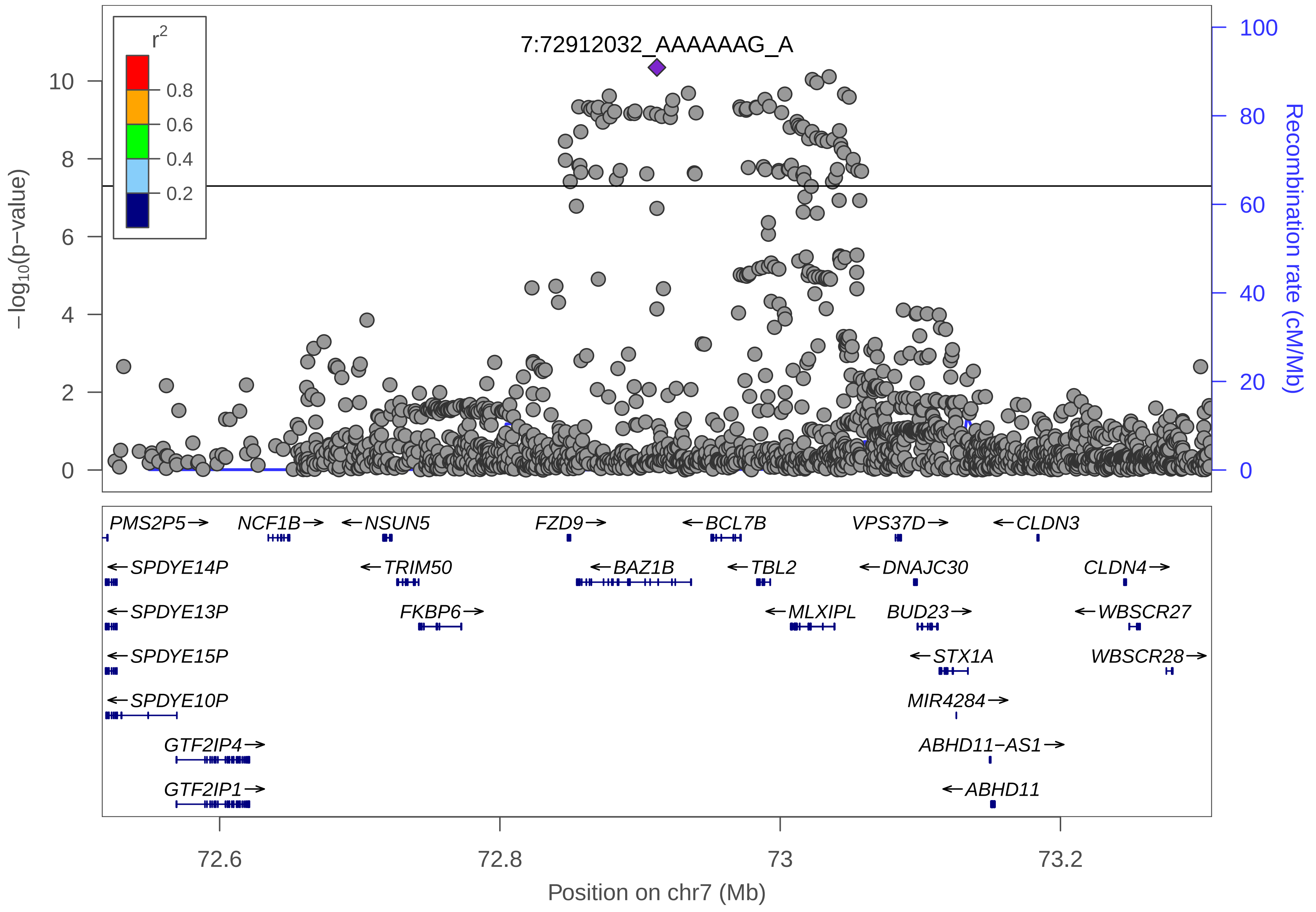

r2
0.8
0.6
0.4
0.2
7:72912032_AAAAAAG_A
−log10(p−value)
Recombination rate (cM/Mb)
0
2
4
6
8
10
0
20
40
60
80
100
PMS2P5→
NCF1B→
←NSUN5
FZD9→
←BCL7B
VPS37D→
←CLDN3
←SPDYE14P
←TRIM50
←BAZ1B
←TBL2
←DNAJC30
CLDN4→
←SPDYE13P
FKBP6→
←MLXIPL
BUD23→
←WBSCR27
←SPDYE15P
←STX1A
WBSCR28→
←SPDYE10P
MIR4284→
GTF2IP4→
ABHD11−AS1→
GTF2IP1→
←ABHD11
72.6
72.8
73
73.2
Position on chr7 (Mb)

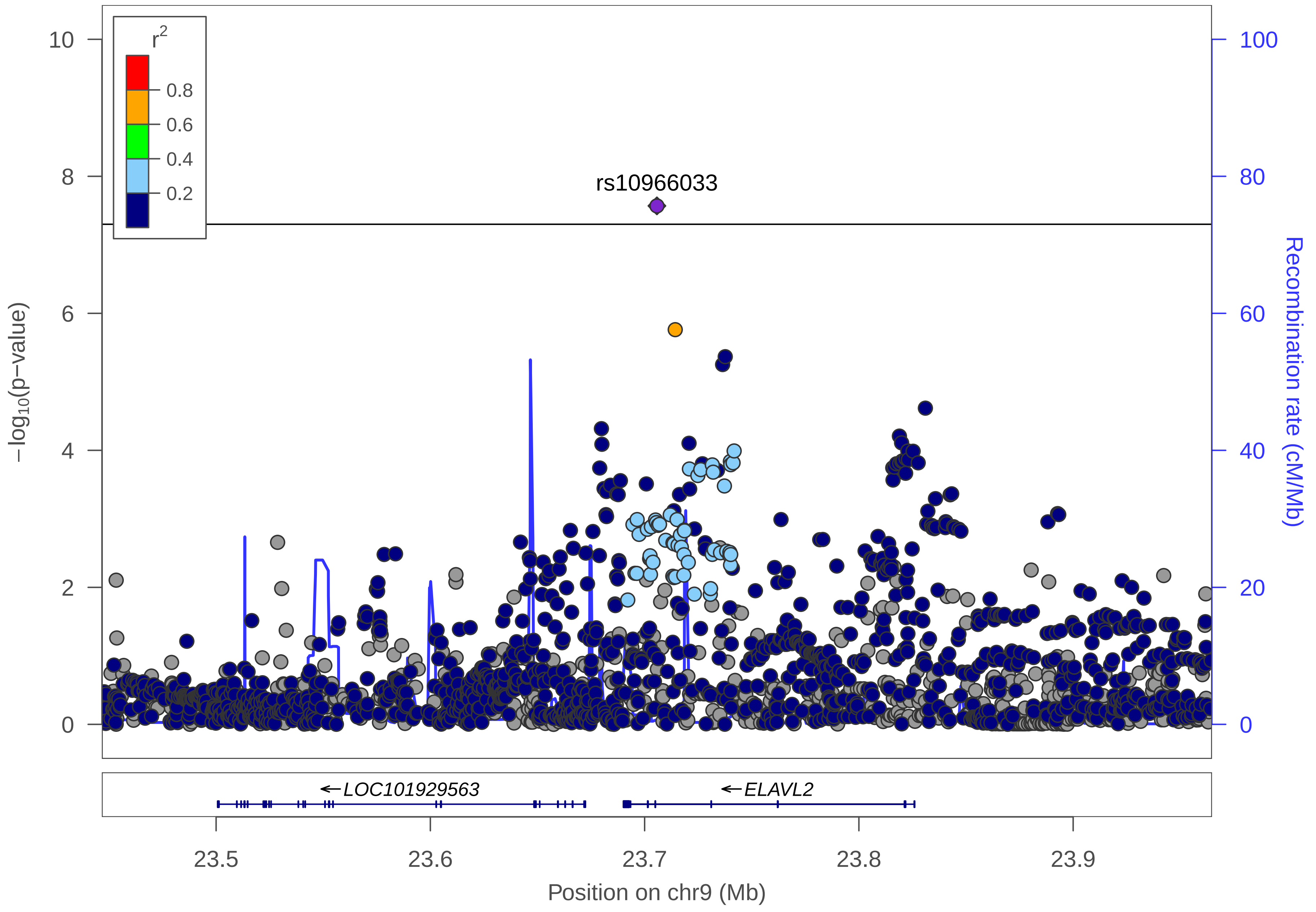

r2
0.8
0.6
0.4
0.2
rs10966033
10
8
6
4
2
0
−log10(p−value)
100
80
60
40
20
0
Recombination rate (cM/Mb)
←LOC101929563
←ELAVL2
23.5
23.6
23.7
23.8
23.9
Position on chr9 (Mb)

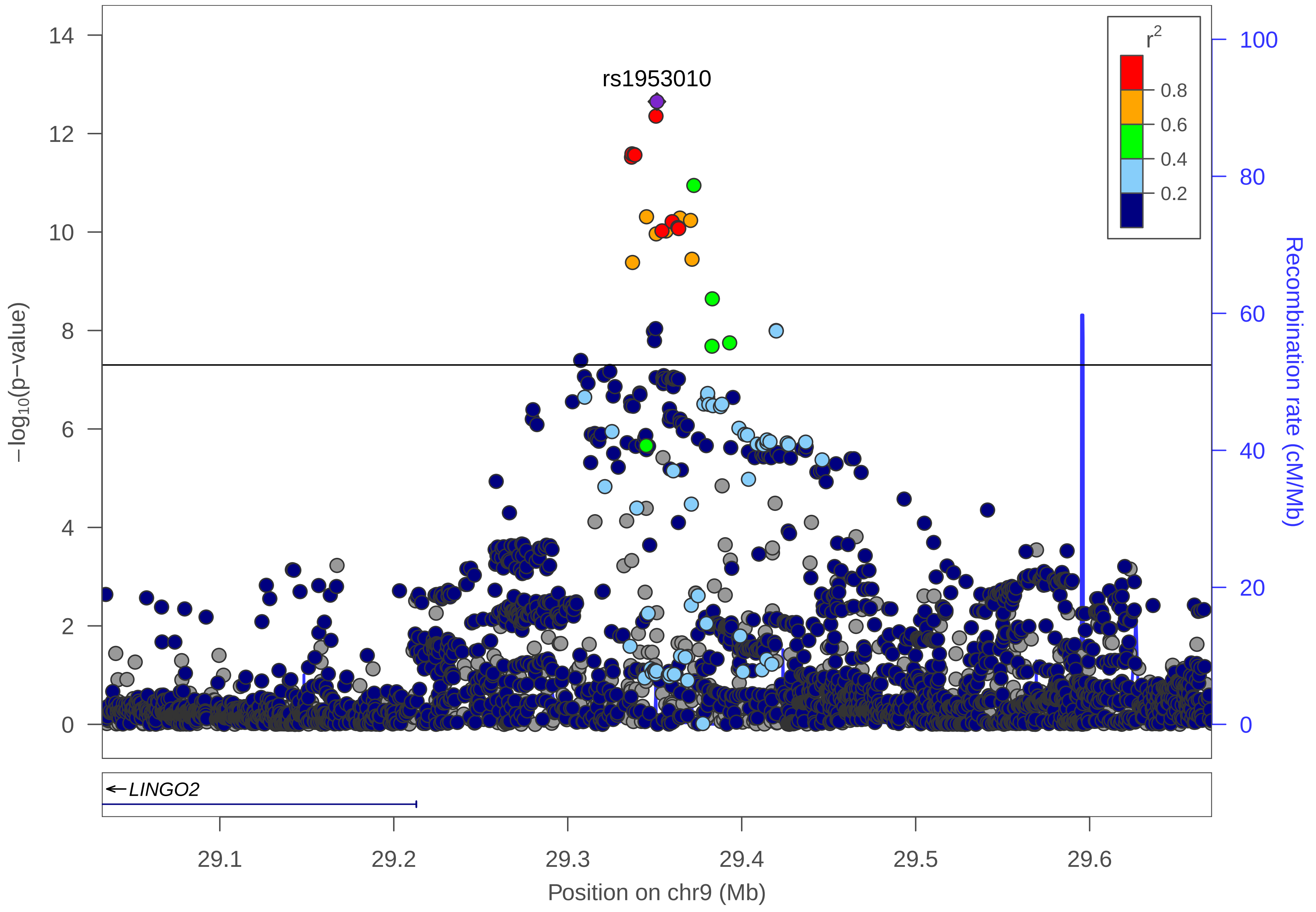

rs1953010
r2
0.8
0.6
0.4
0.2
−log10(p−value)
Recombination rate (cM/Mb)
LINGO2
Position on chr9 (Mb)
29.1
29.2
29.3
29.4
29.5
29.6

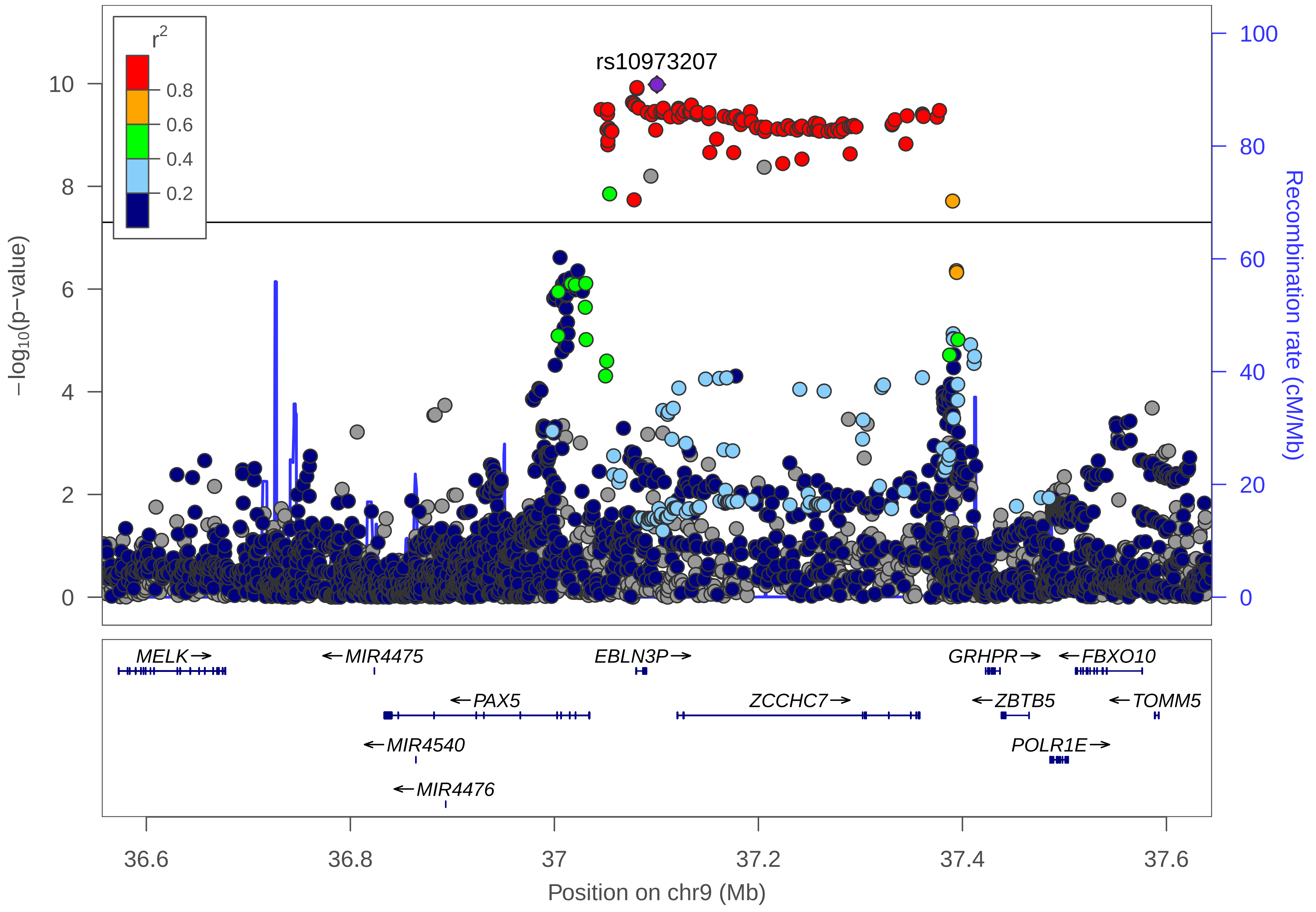

r2
0.8
0.6
0.4
0.2
rs10973207
10
8
6
4
2
0
−log10(p−value)
100
80
60
40
20
0
Recombination rate (cM/Mb)
MELK→
←MIR4475
EBLN3P→
GRHPR→
←FBXO10
←PAX5
ZCCHC7→
←ZBTB5
←TOMM5
←MIR4540
POLR1E→
←MIR4476
36.6
36.8
37
37.2
37.4
37.6
Position on chr9 (Mb)

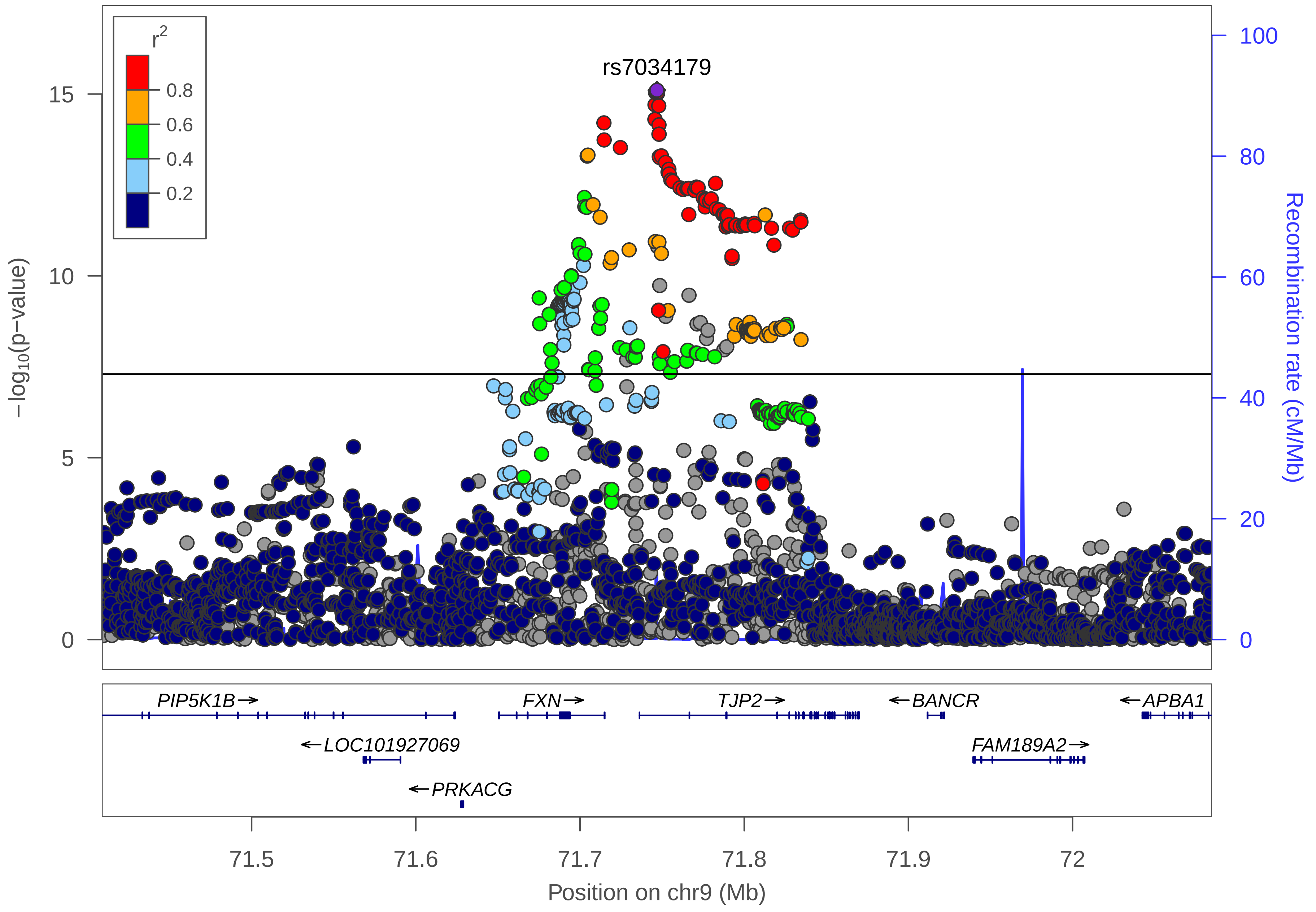
r2
0.8
0.6
0.4
0.2
rs7034179
15
10
5
0
−log10(p−value)
100
80
60
40
20
0
Recombination rate (cM/Mb)
PIP5K1B→
FXN→
TJP2→
←BANCR
←APBA1
←LOC101927069
FAM189A2→
←PRKACG
71.5
71.6
71.7
71.8
71.9
72
Position on chr9 (Mb)

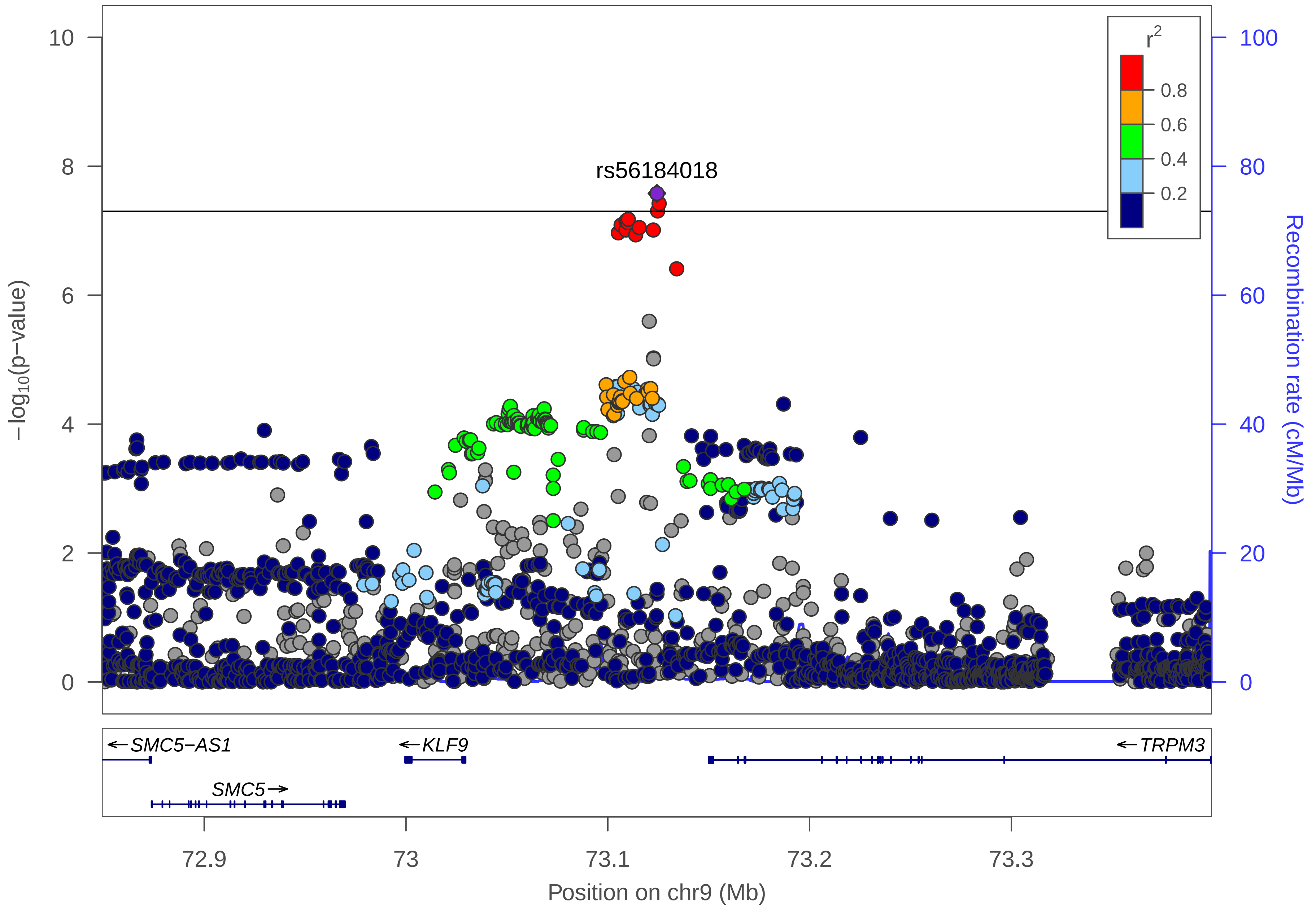
rs56184018
r2
0.8
0.6
0.4
0.2
−log10(p−value)
Recombination rate (cM/Mb)
10
8
6
4
2
0
100
80
60
40
20
0
←SMC5−AS1
←KLF9
←TRPM3
SMC5→
72.9
73
73.1
73.2
73.3
Position on chr9 (Mb)

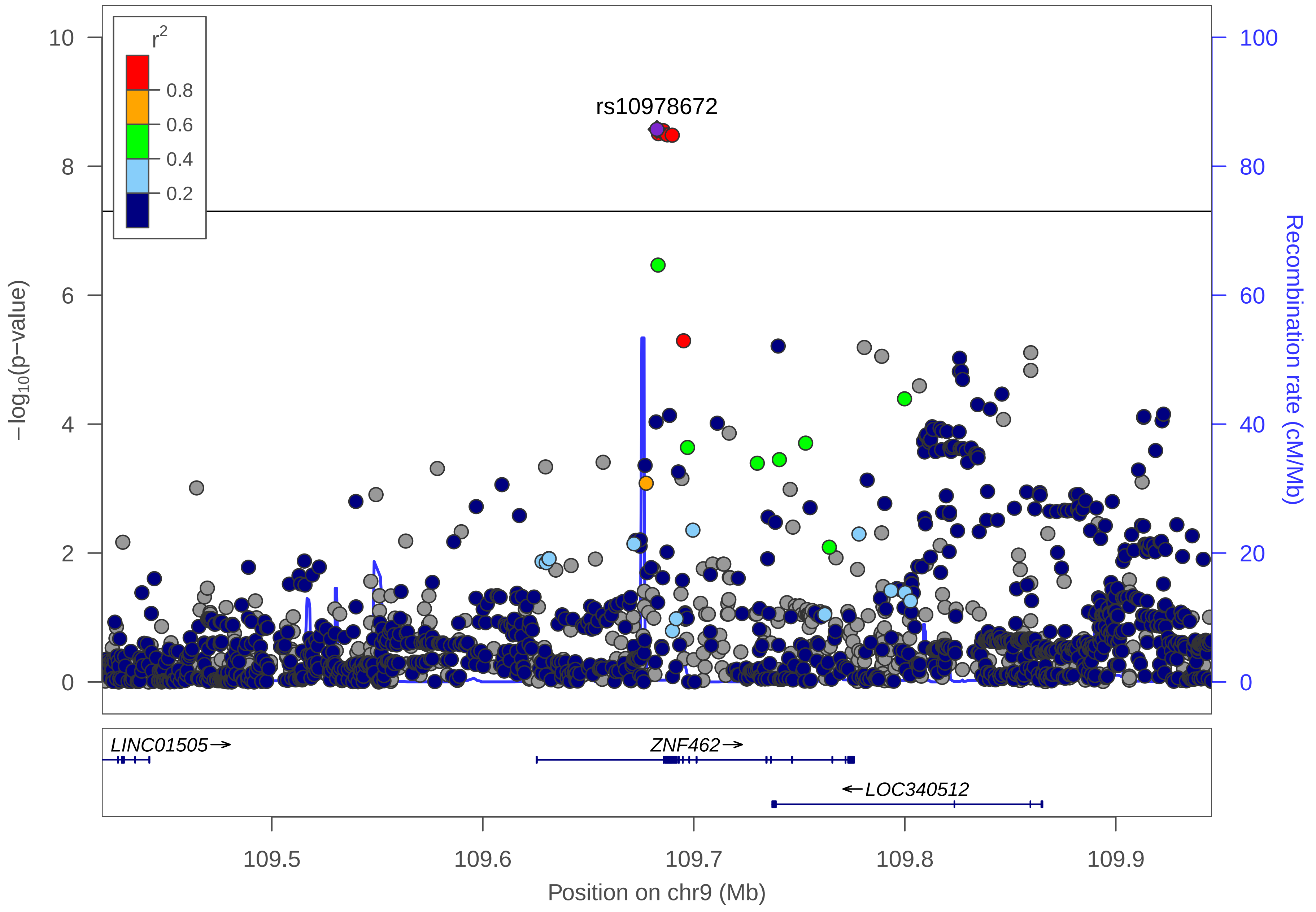

r2
0.8
0.6
0.4
0.2
rs10978672
10
8
6
4
2
0
−log10(p−value)
100
80
60
40
20
0
Recombination rate (cM/Mb)
LINC01505→
ZNF462→
←LOC340512
109.5
109.6
109.7
109.8
109.9
Position on chr9 (Mb)

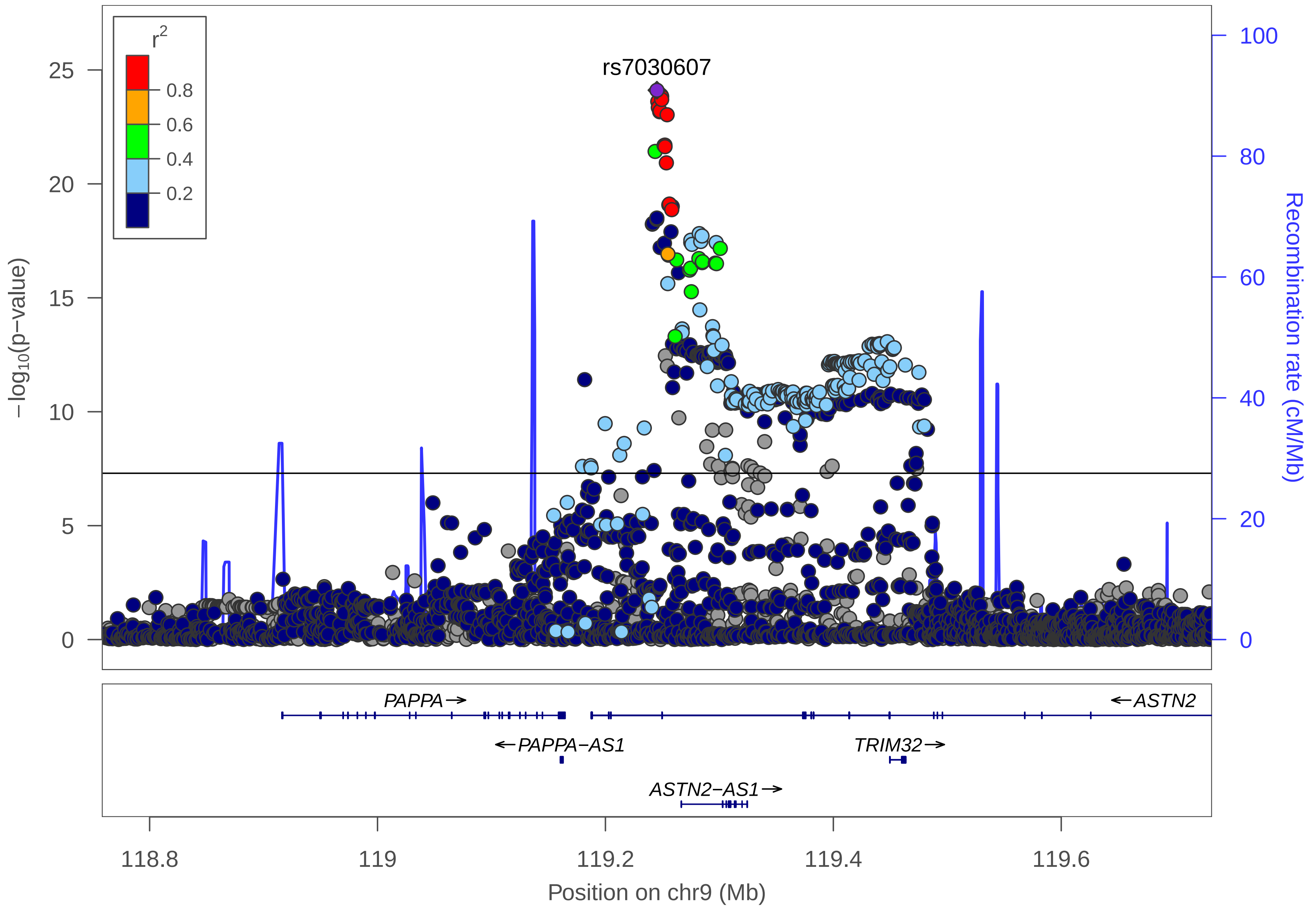

r2
0.8
0.6
0.4
0.2
rs7030607
25
20
15
10
5
0
−log10(p−value)
100
80
60
40
20
0
Recombination rate (cM/Mb)
PAPPA→
←ASTN2
←PAPPA−AS1
TRIM32→
ASTN2−AS1→
118.8
119
119.2
119.4
119.6
Position on chr9 (Mb)

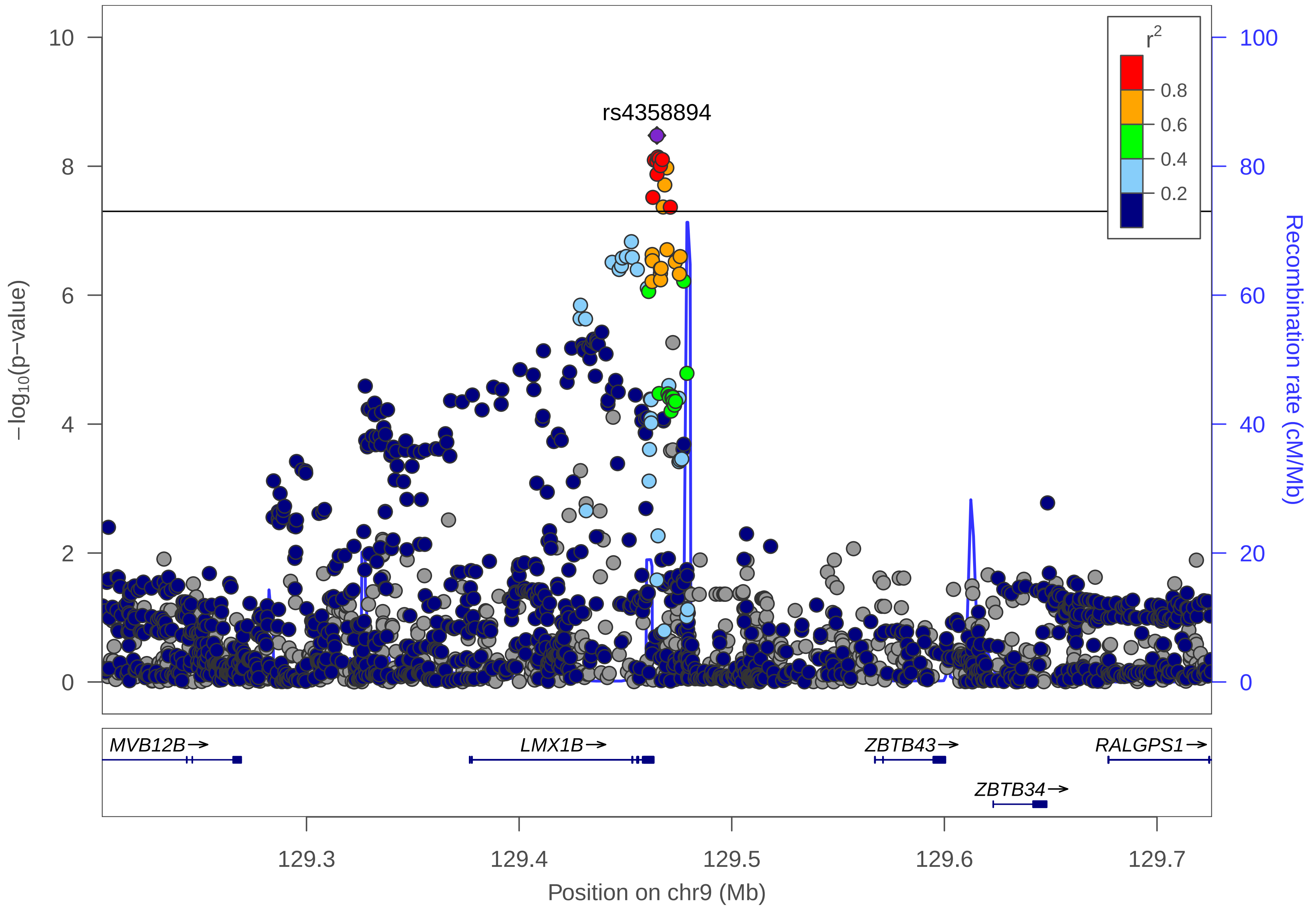

rs4358894
r2
0.8
0.6
0.4
0.2
10
8
6
4
2
0
100
80
60
40
20
0
−log10(p−value)
Recombination rate (cM/Mb)
MVB12B→
LMX1B→
ZBTB43→
RALGPS1→
ZBTB34→
129.3
129.4
129.5
129.6
129.7
Position on chr9 (Mb)

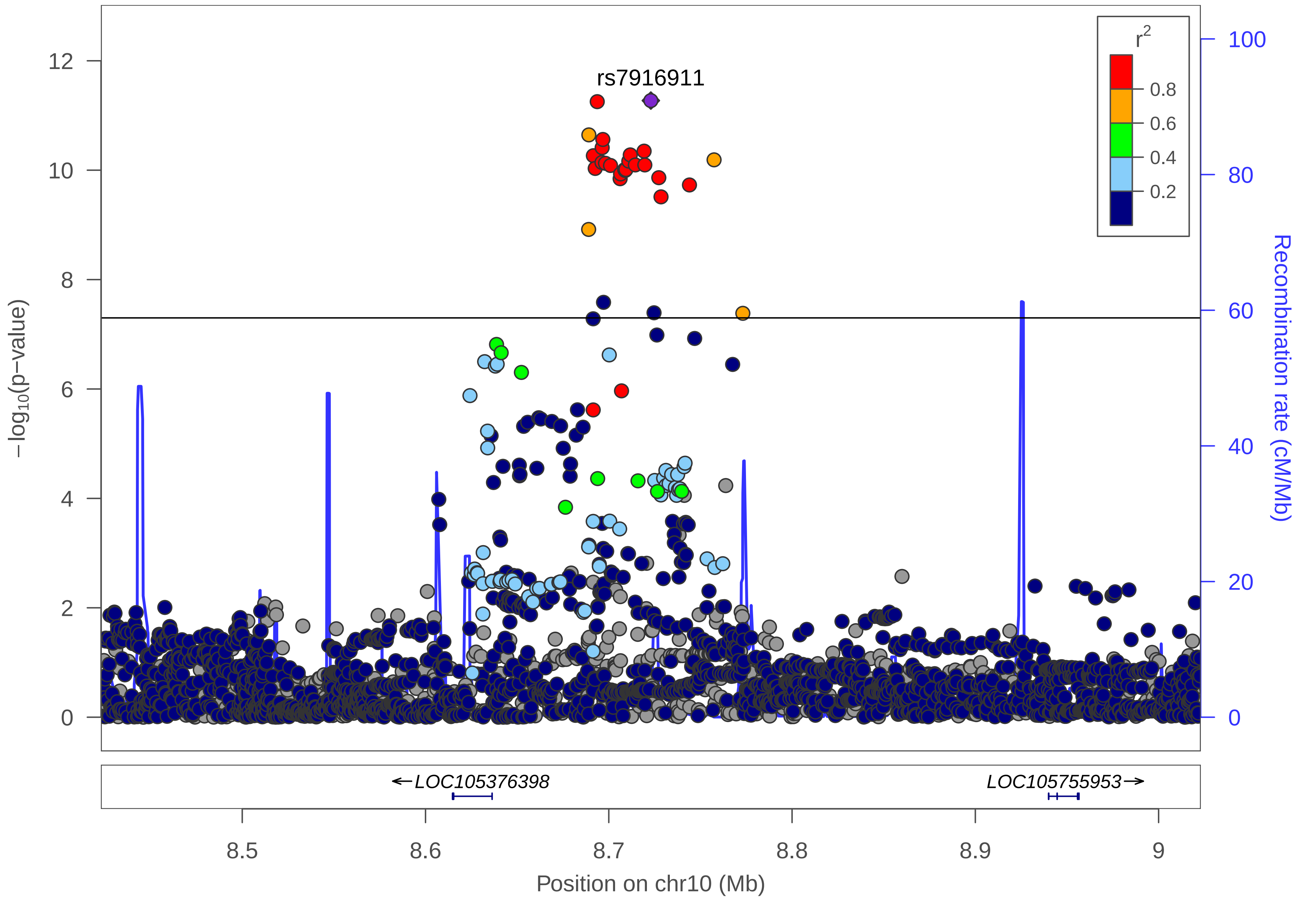
rs7916911
r2
0.8
0.6
0.4
0.2
12
10
8
6
4
2
0
−log10(p−value)
100
80
60
40
20
0
Recombination rate (cM/Mb)
LOC105376398
LOC105755953
8.5
8.6
8.7
8.8
8.9
9
Position on chr10 (Mb)

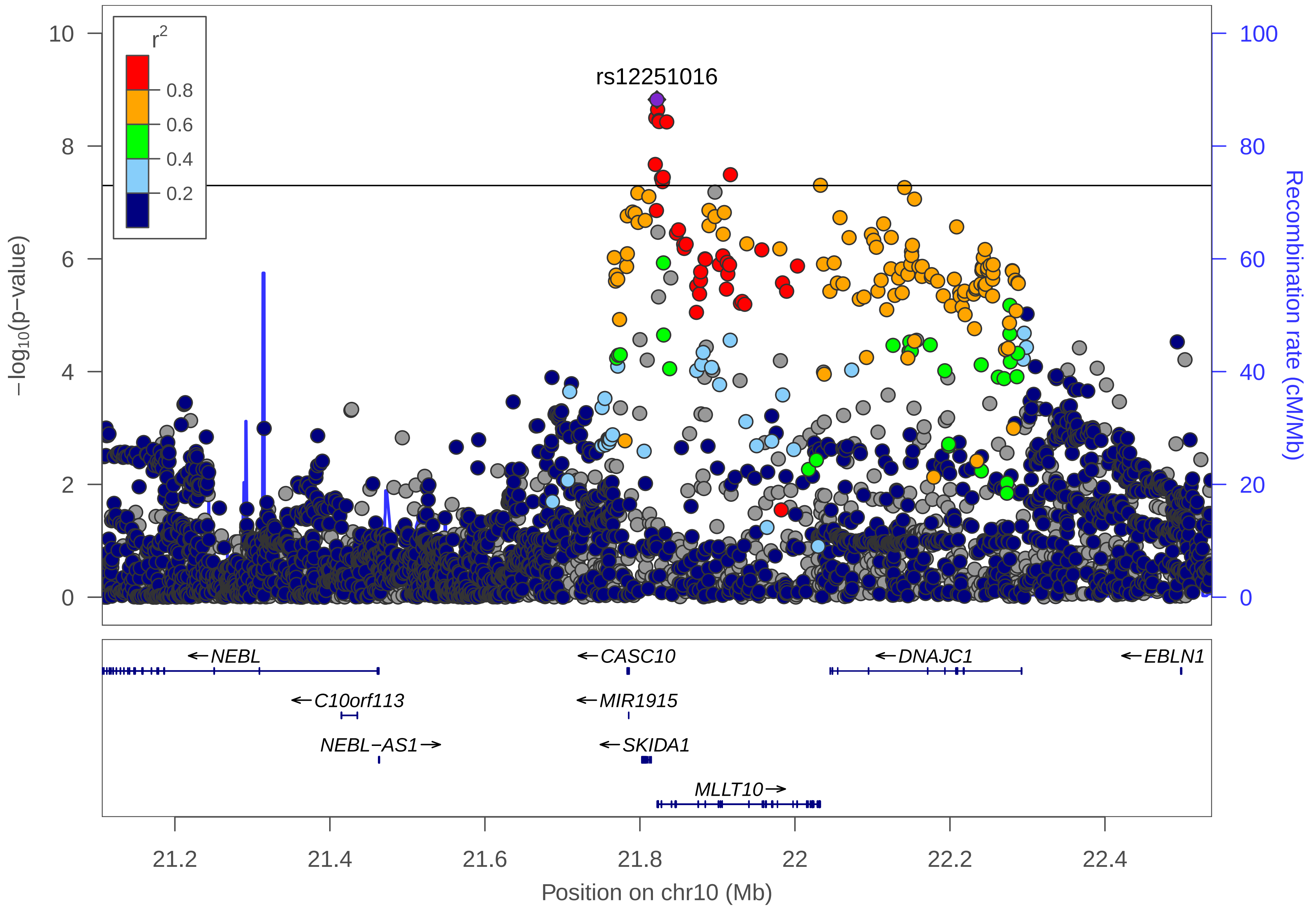

r2
0.8
0.6
0.4
0.2
rs12251016
10
8
6
4
2
0
−log10(p−value)
100
80
60
40
20
0
Recombination rate (cM/Mb)
←NEBL
←C10orf113
NEBL−AS1→
←CASC10
←MIR1915
←SKIDA1
MLLT10→
←DNAJC1
←EBLN1
21.2
21.4
21.6
21.8
22
22.2
22.4
Position on chr10 (Mb)

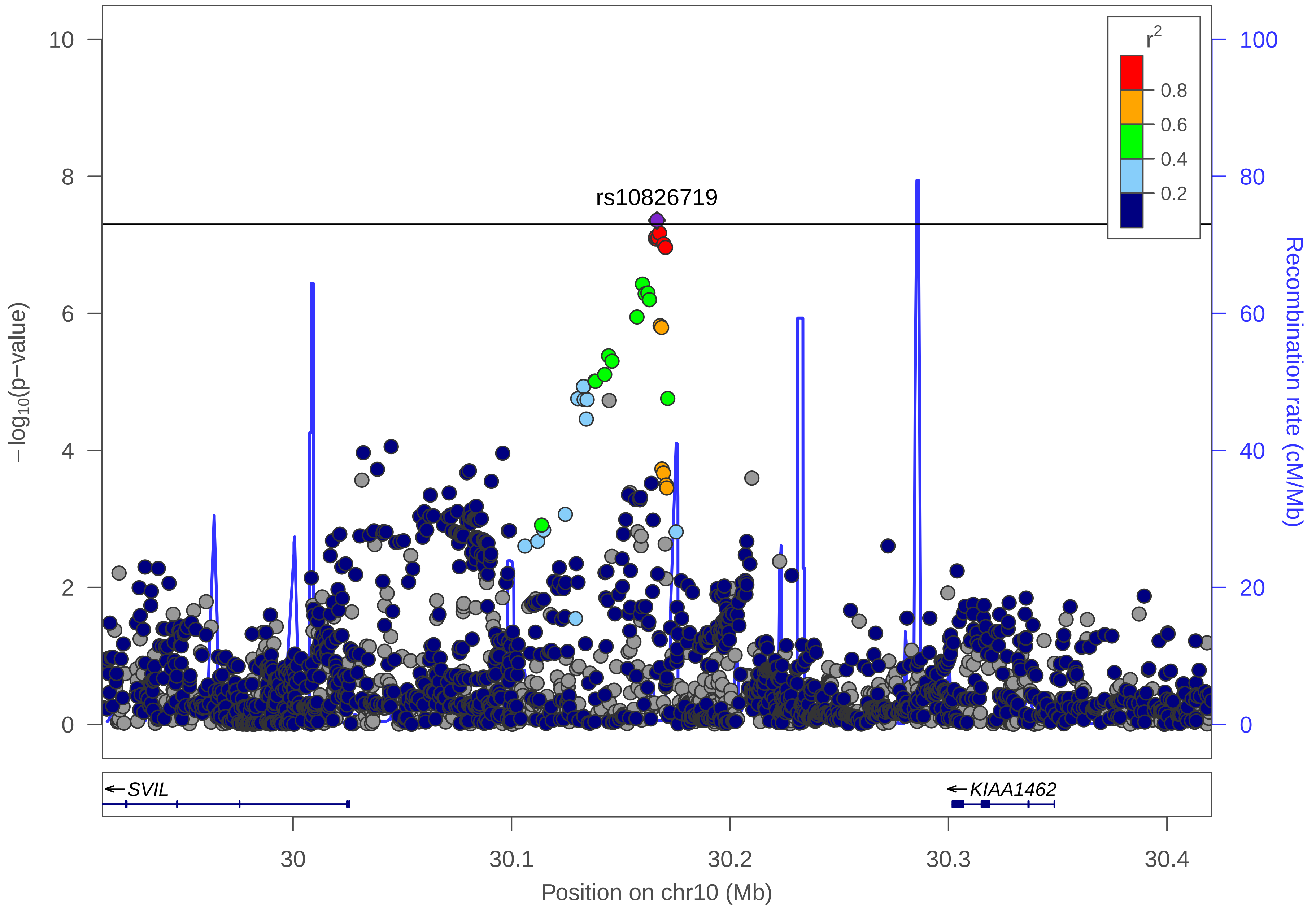

r2
0.8
0.6
0.4
0.2
rs10826719
10
8
6
4
2
0
−log10(p−value)
100
80
60
40
20
0
Recombination rate (cM/Mb)
SVIL
KIAA1462
30
30.1
30.2
30.3
30.4
Position on chr10 (Mb)

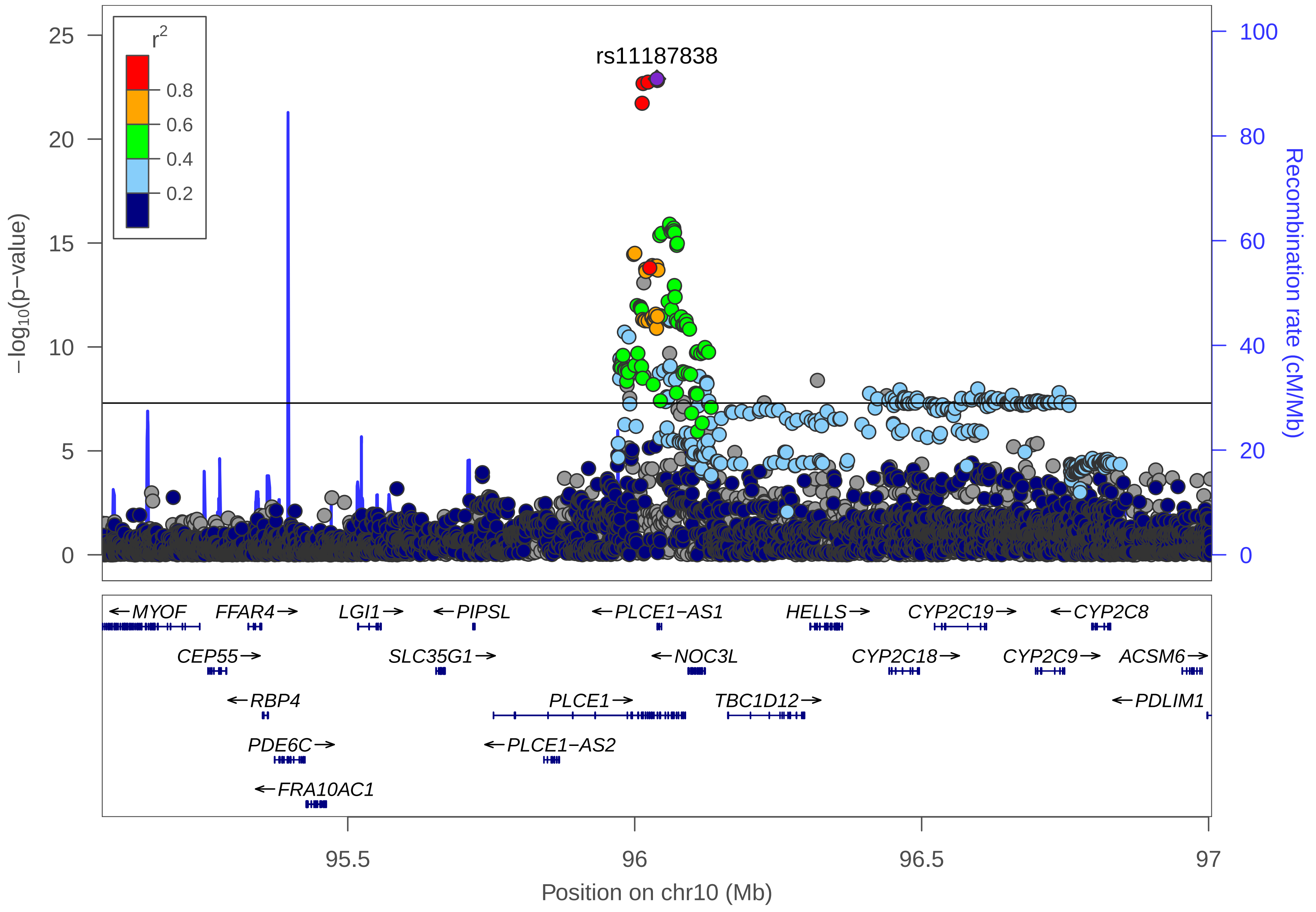

rs11187838
r2
0.8
0.6
0.4
0.2
−log10(p−value)
Recombination rate (cM/Mb)
25
20
15
10
5
0
100
80
60
40
20
0
←MYOF
FFAR4→
LGI1→
←PIPSL
←PLCE1−AS1
HELLS→
CYP2C19→
←CYP2C8
CEP55→
SLC35G1→
←NOC3L
CYP2C18→
CYP2C9→
ACSM6→
←RBP4
PLCE1→
TBC1D12→
←PDLIM1
PDE6C→
←PLCE1−AS2
←FRA10AC1
95.5
96
96.5
97
Position on chr10 (Mb)

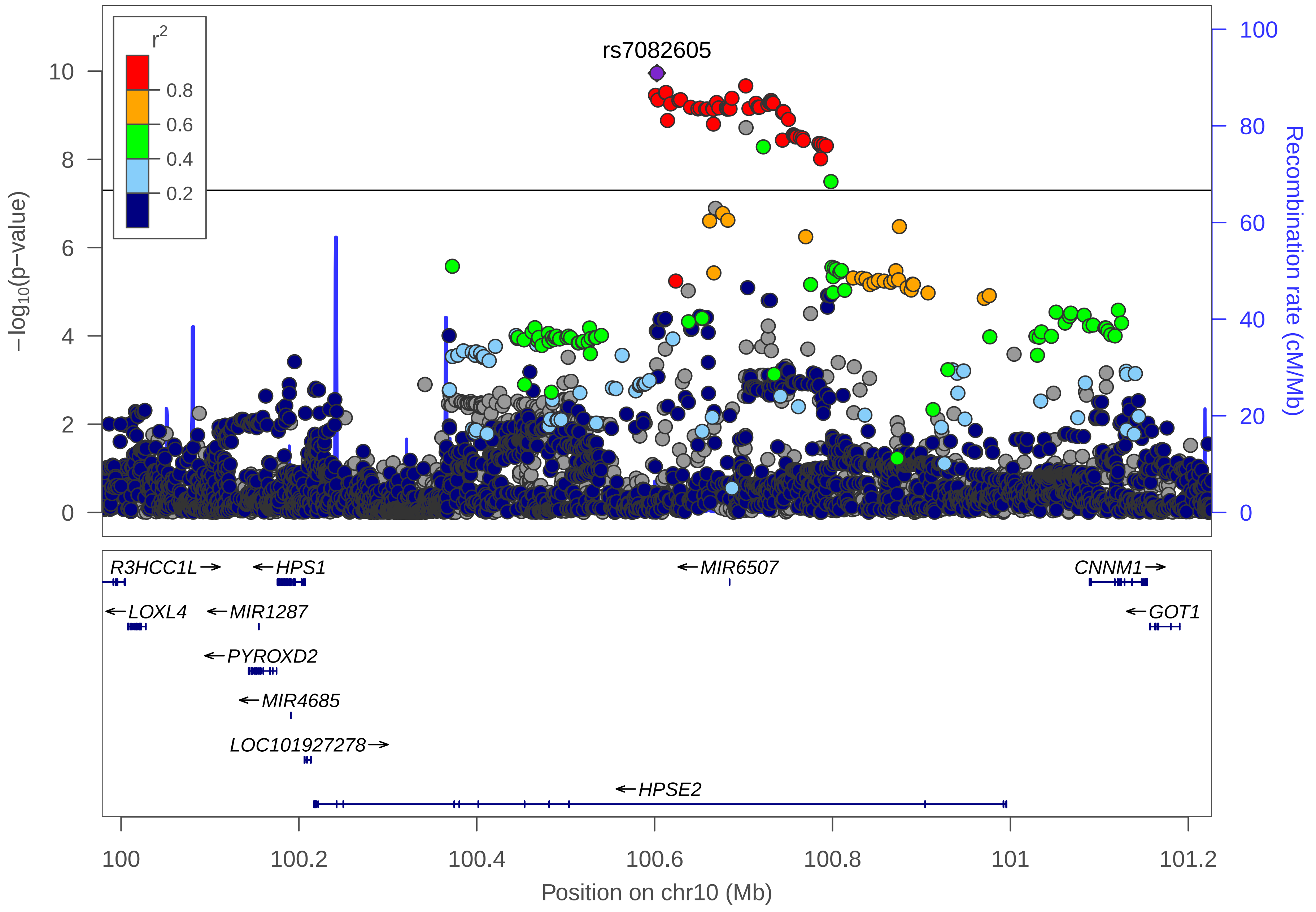
rs7082605
r2
0.8
0.6
0.4
0.2
−log10(p−value)
Recombination rate (cM/Mb)
R3HCC1L→
←HPS1
←MIR6507
CNNM1→
←LOXL4
←MIR1287
←GOT1
←PYROXD2
←MIR4685
LOC101927278→
←HPSE2
Position on chr10 (Mb)
100
100.2
100.4
100.6
100.8
101
101.2

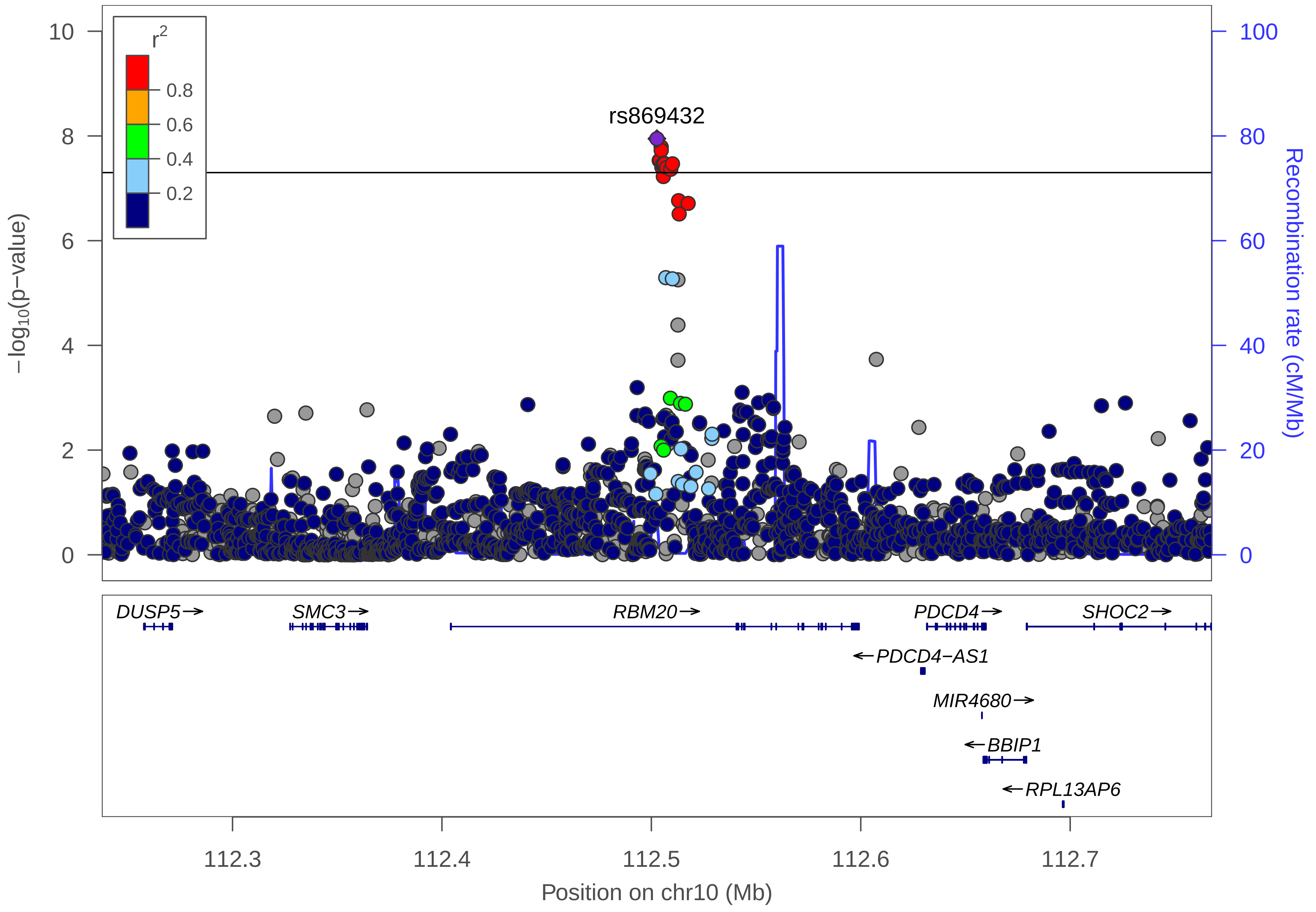

r2
0.8
0.6
0.4
0.2
rs869432
−log10(p−value)
Recombination rate (cM/Mb)
10
8
6
4
2
0
100
80
60
40
20
0
DUSP5→
SMC3→
RBM20→
PDCD4→
SHOC2→
←PDCD4−AS1
MIR4680→
←BBIP1
←RPL13AP6
112.3
112.4
112.5
112.6
112.7
Position on chr10 (Mb)

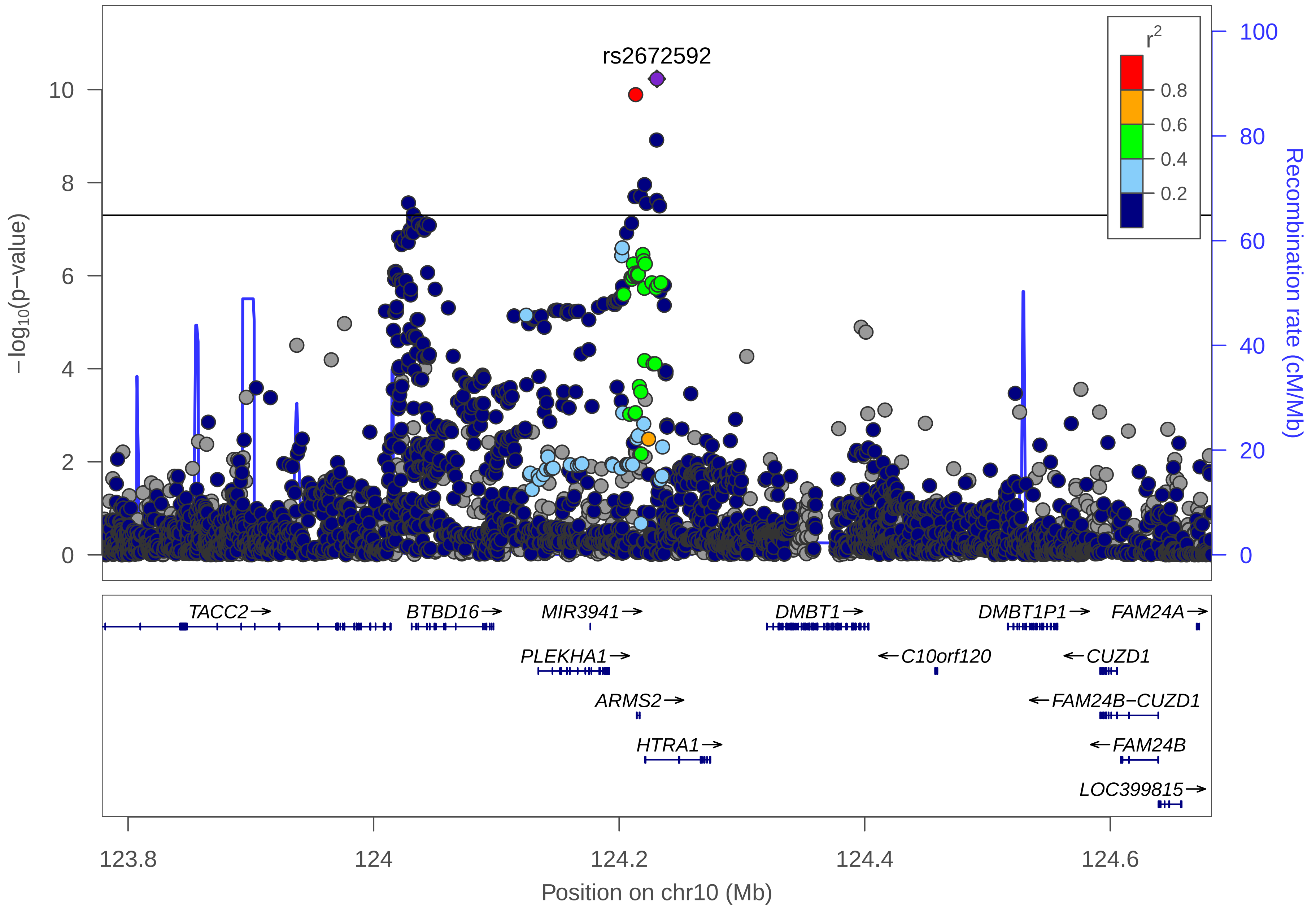
rs2672592
r²
0.8
0.6
0.4
0.2
−log10(p−value)
Recombination rate (cM/Mb)
TACC2→
BTBD16→
MIR3941→
DMBT1→
DMBT1P1→
FAM24A→
PLEKHA1→
←C10orf120
←CUZD1
ARMS2→
←FAM24B−CUZD1
HTRA1→
←FAM24B
LOC399815→
123.8
124
124.2
124.4
124.6
Position on chr10 (Mb)

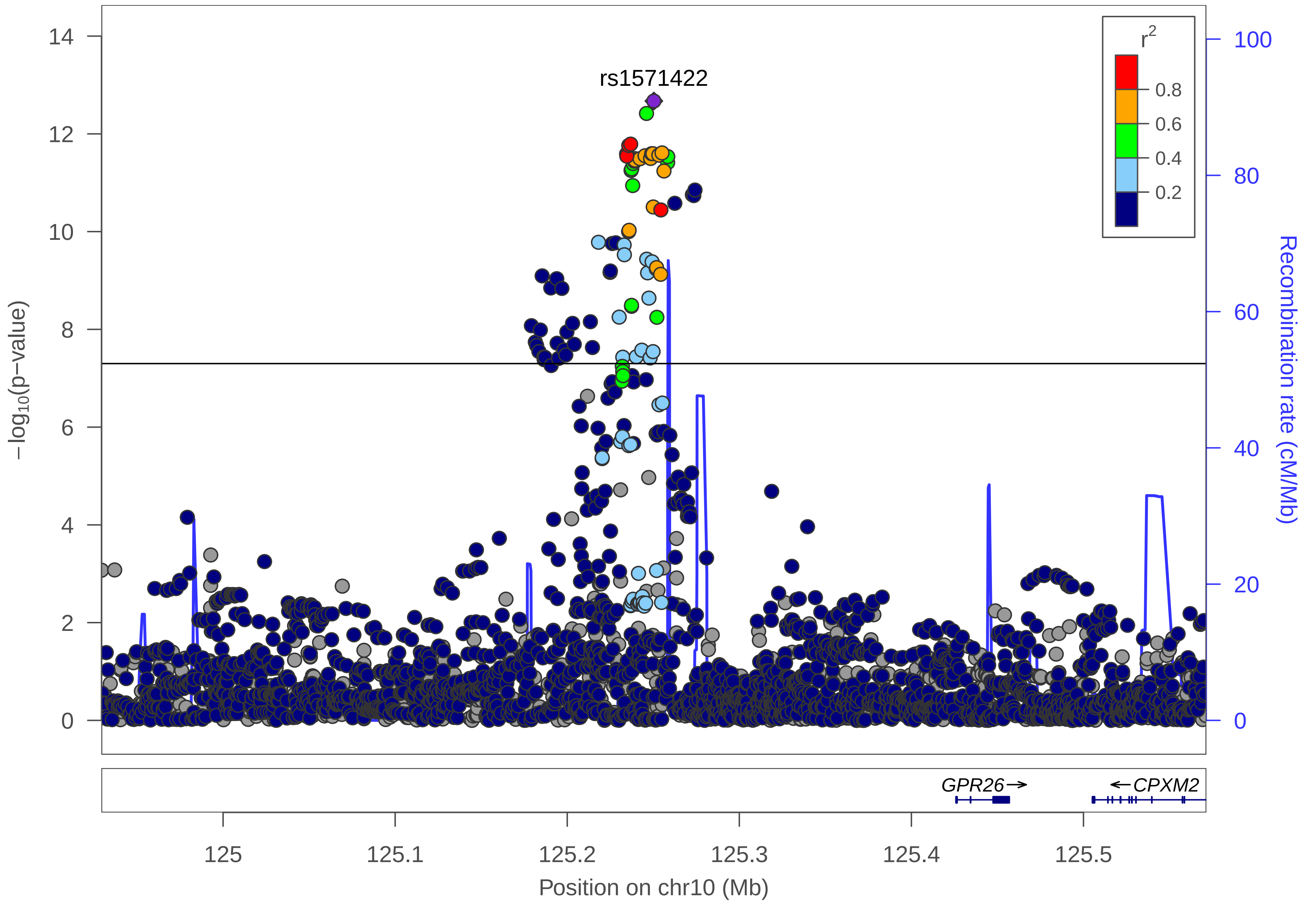

rs1571422
r2
0.8
0.6
0.4
0.2
−log10(p−value)
Recombination rate (cM/Mb)
14
12
10
8
6
4
2
0
100
80
60
40
20
0
GPR26→
←CPXM2
125
125.1
125.2
125.3
125.4
125.5
Position on chr10 (Mb)

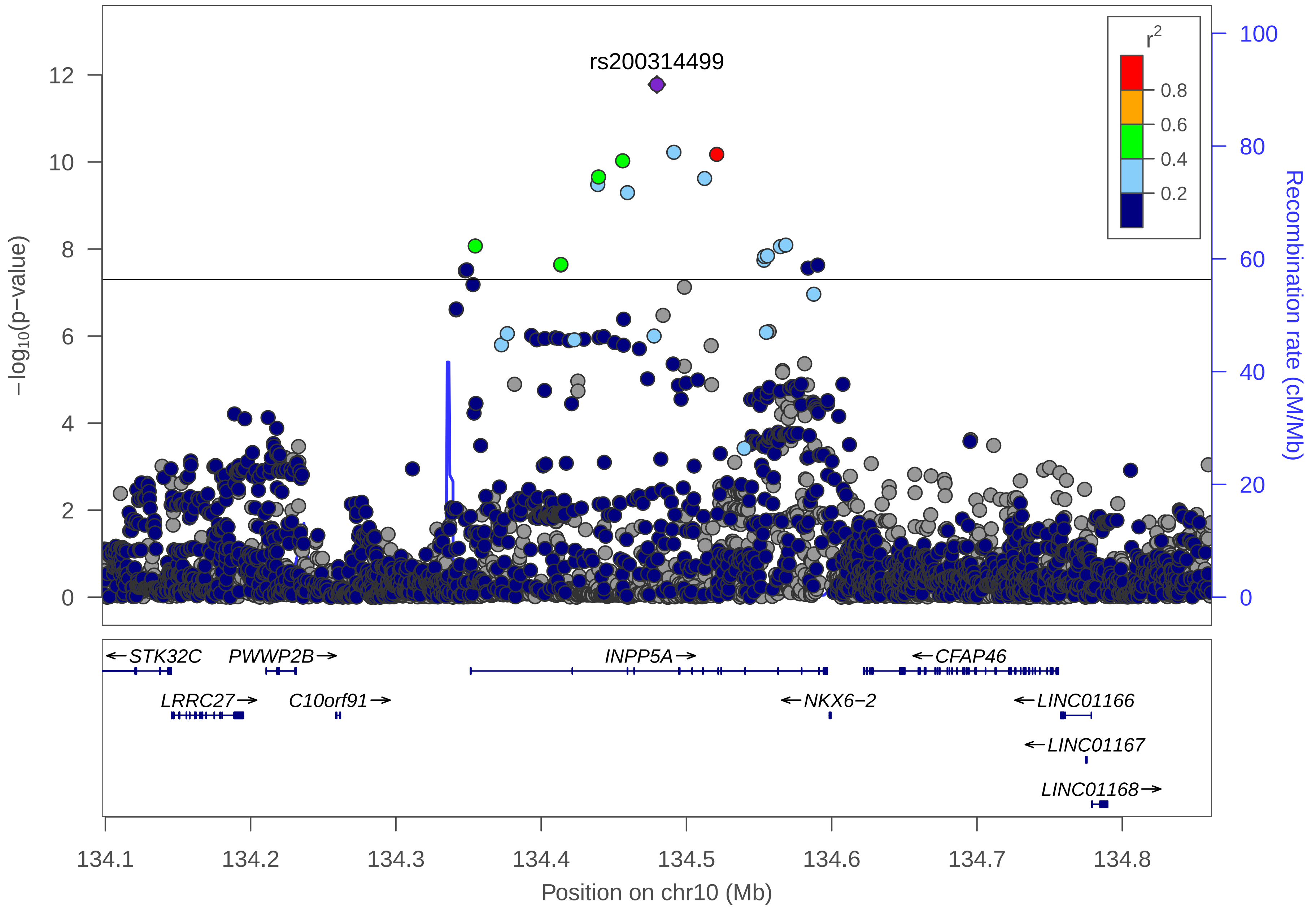
rs200314499
r2
0.8
0.6
0.4
0.2
−log10(p−value)
Recombination rate (cM/Mb)
0
2
4
6
8
10
12
0
20
40
60
80
100
STK32C
PWWP2B
INPP5A
CFAP46
LRRC27
C10orf91
NKX6−2
LINC01166
LINC01167
LINC01168
134.1
134.2
134.3
134.4
134.5
134.6
134.7
134.8
Position on chr10 (Mb)

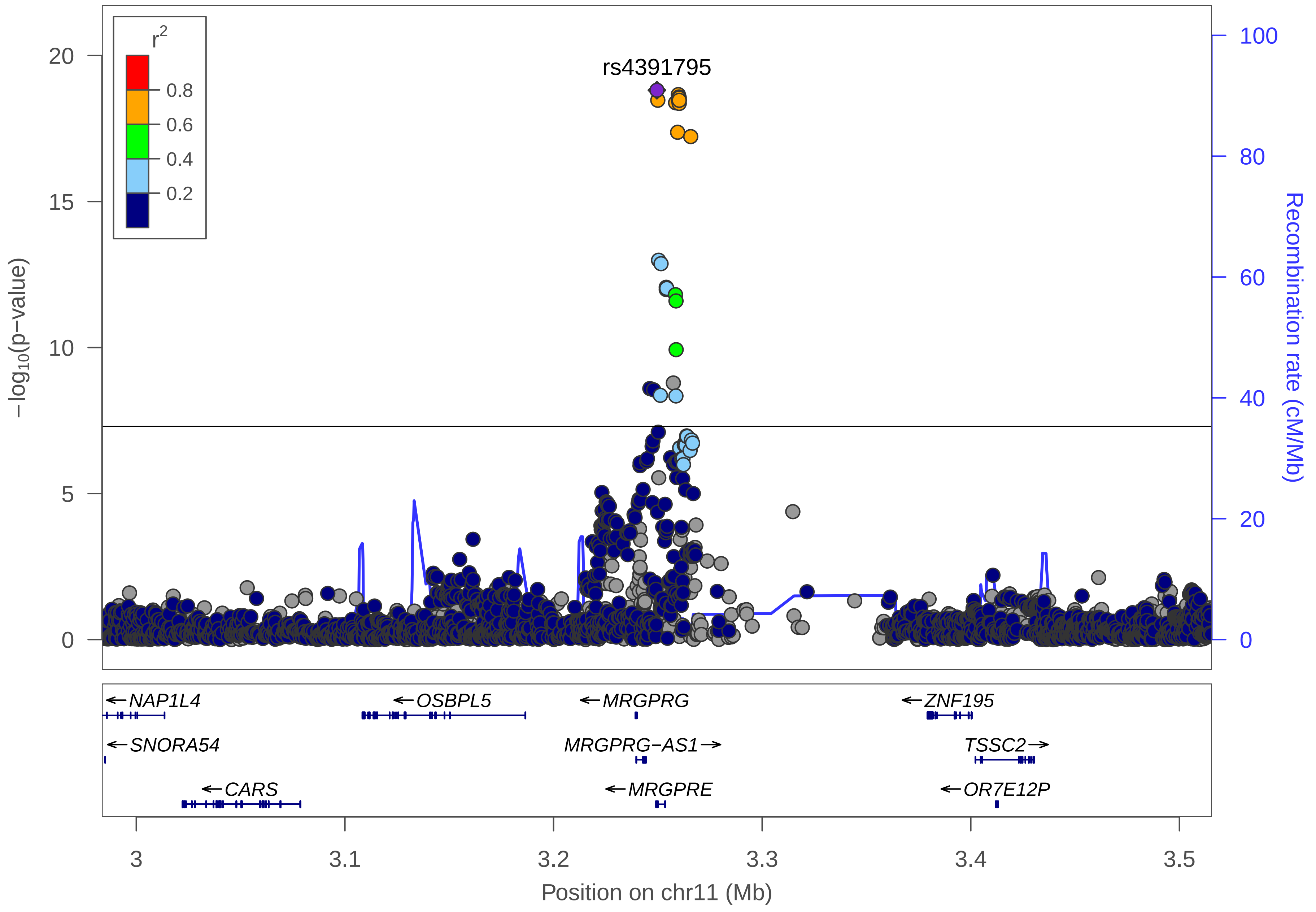
r2
0.8
0.6
0.4
0.2
rs4391795
20
15
10
5
0
−log10(p−value)
100
80
60
40
20
0
Recombination rate (cM/Mb)
←NAP1L4
←SNORA54
←CARS
←OSBPL5
←MRGPRG
MRGPRG−AS1→
←MRGPRE
←ZNF195
TSSC2→
←OR7E12P
3
3.1
3.2
3.3
3.4
3.5
Position on chr11 (Mb)

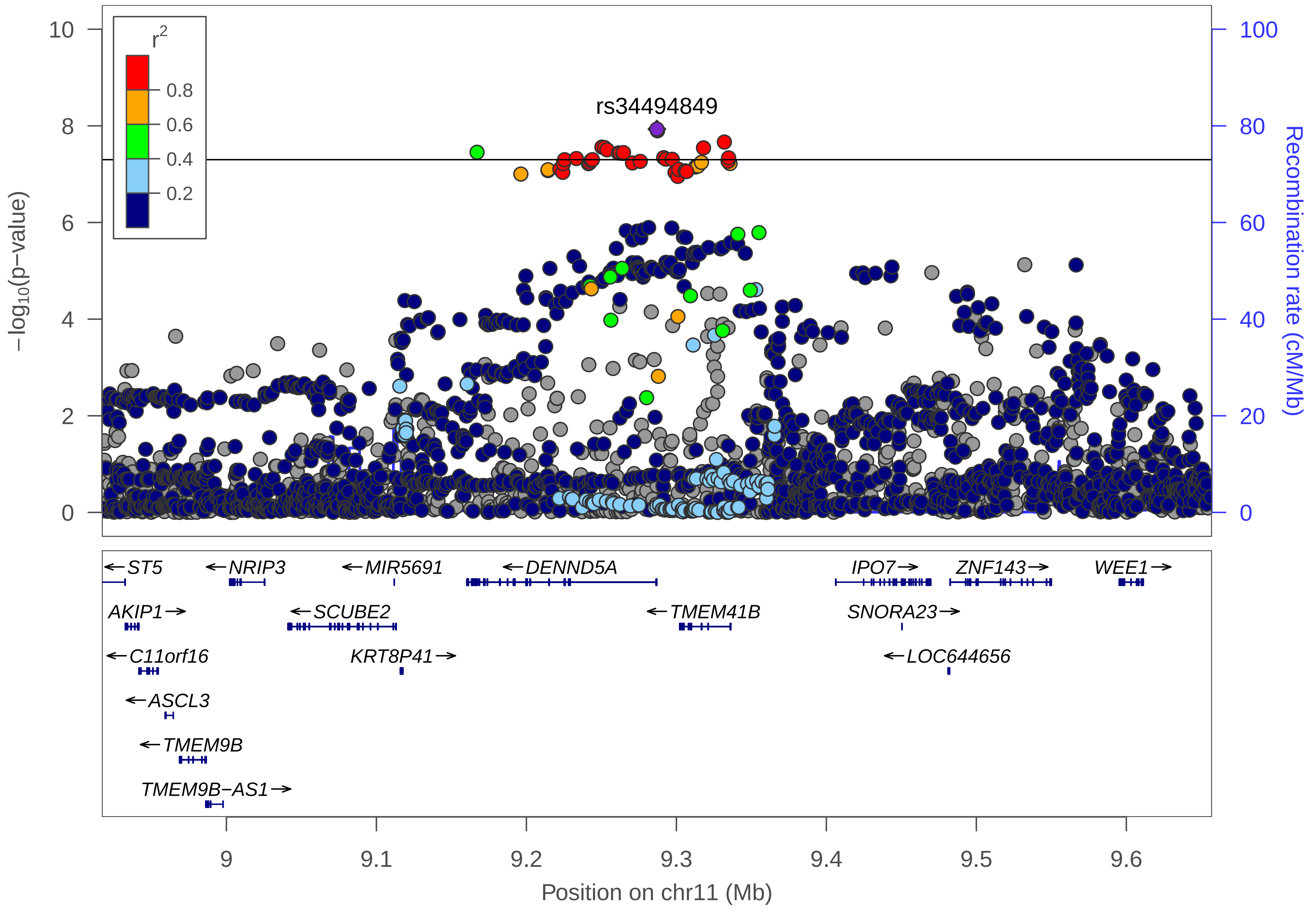

r2
0.8
0.6
0.4
0.2
rs34494849
−log10(p−value)
Recombination rate (cM/Mb)
0
2
4
6
8
10
0
20
40
60
80
100
ST5
NRIP3
MIR5691
DENND5A
IPO7
ZNF143
WEE1
AKIP1
SCUBE2
TMEM41B
SNORA23
C11orf16
KRT8P41
LOC644656
ASCL3
TMEM9B
TMEM9B−AS1
9
9.1
9.2
9.3
9.4
9.5
9.6
Position on chr11 (Mb)

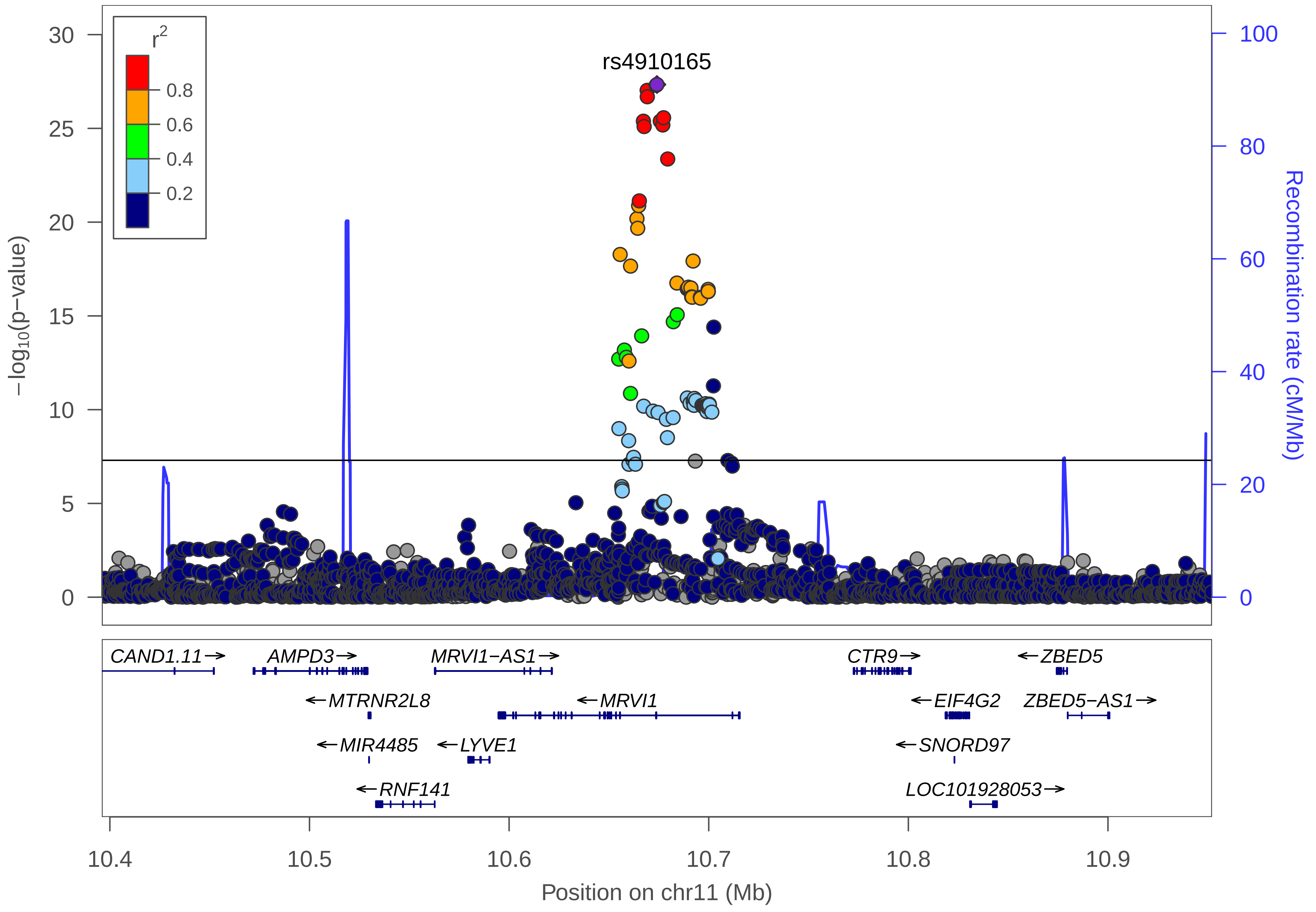

r2
0.8
0.6
0.4
0.2
rs4910165
−log10(p−value)
Recombination rate (cM/Mb)
30
25
20
15
10
5
0
100
80
60
40
20
0
CAND1.11→
AMPD3→
MRVI1−AS1→
CTR9→
←ZBED5
←MTRNR2L8
←MRVI1
←EIF4G2
ZBED5−AS1→
←MIR4485
←LYVE1
←SNORD97
←RNF141
LOC101928053→
10.4
10.5
10.6
10.7
10.8
10.9
Position on chr11 (Mb)

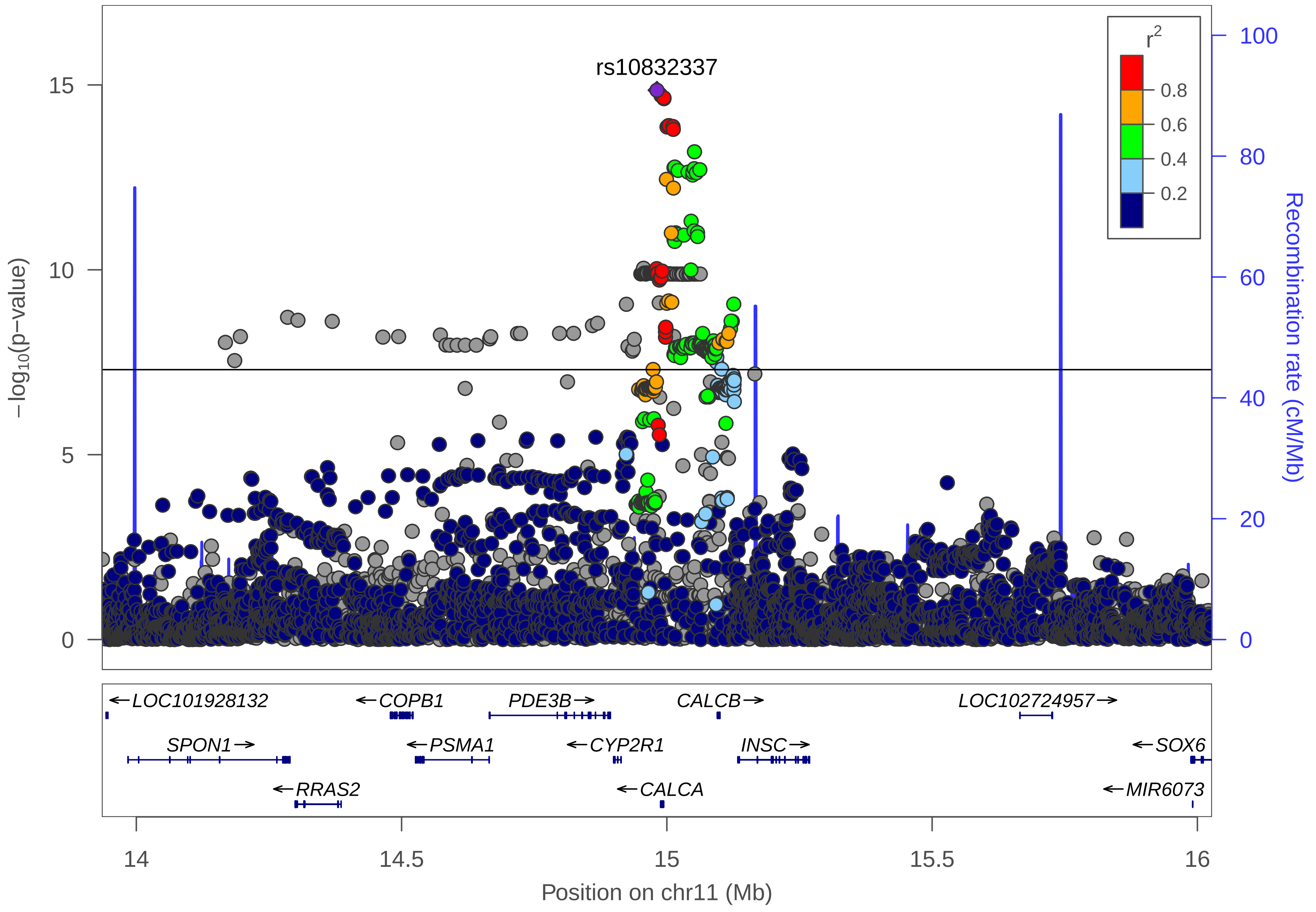

rs10832337
r2
0.8
0.6
0.4
0.2
15
10
5
0
−log10(p−value)
100
80
60
40
20
0
Recombination rate (cM/Mb)
←LOC101928132
←COPB1
PDE3B→
CALCB→
LOC102724957→
SPON1→
←PSMA1
←CYP2R1
INSC→
←SOX6
←RRAS2
←CALCA
←MIR6073
14
14.5
15
15.5
16
Position on chr11 (Mb)

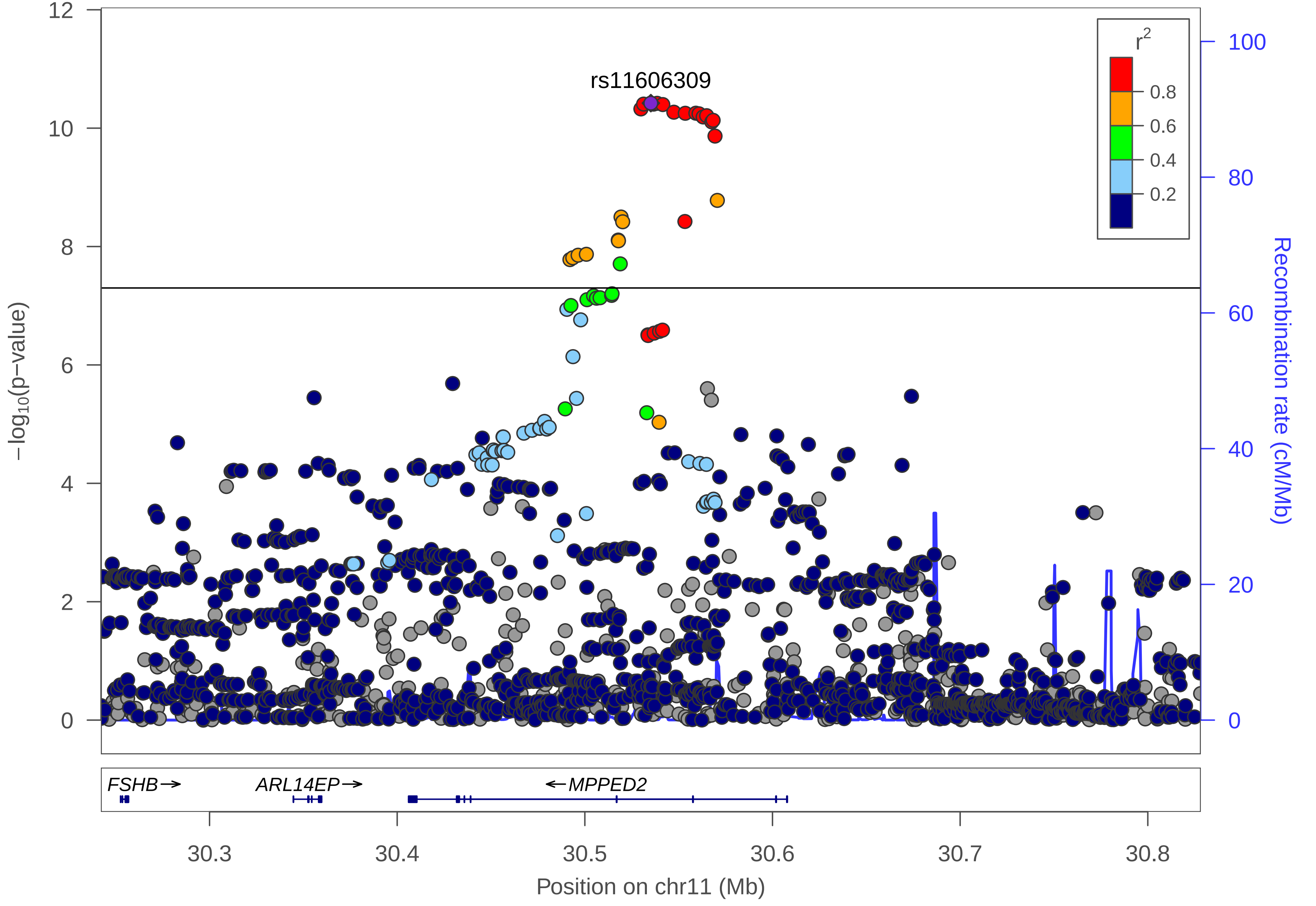

rs11606309
r2
0.8
0.6
0.4
0.2
12
10
8
6
4
2
0
100
80
60
40
20
0
−log10(p−value)
Recombination rate (cM/Mb)
FSHB→
ARL14EP→
←MPPED2
30.3
30.4
30.5
30.6
30.7
30.8
Position on chr11 (Mb)

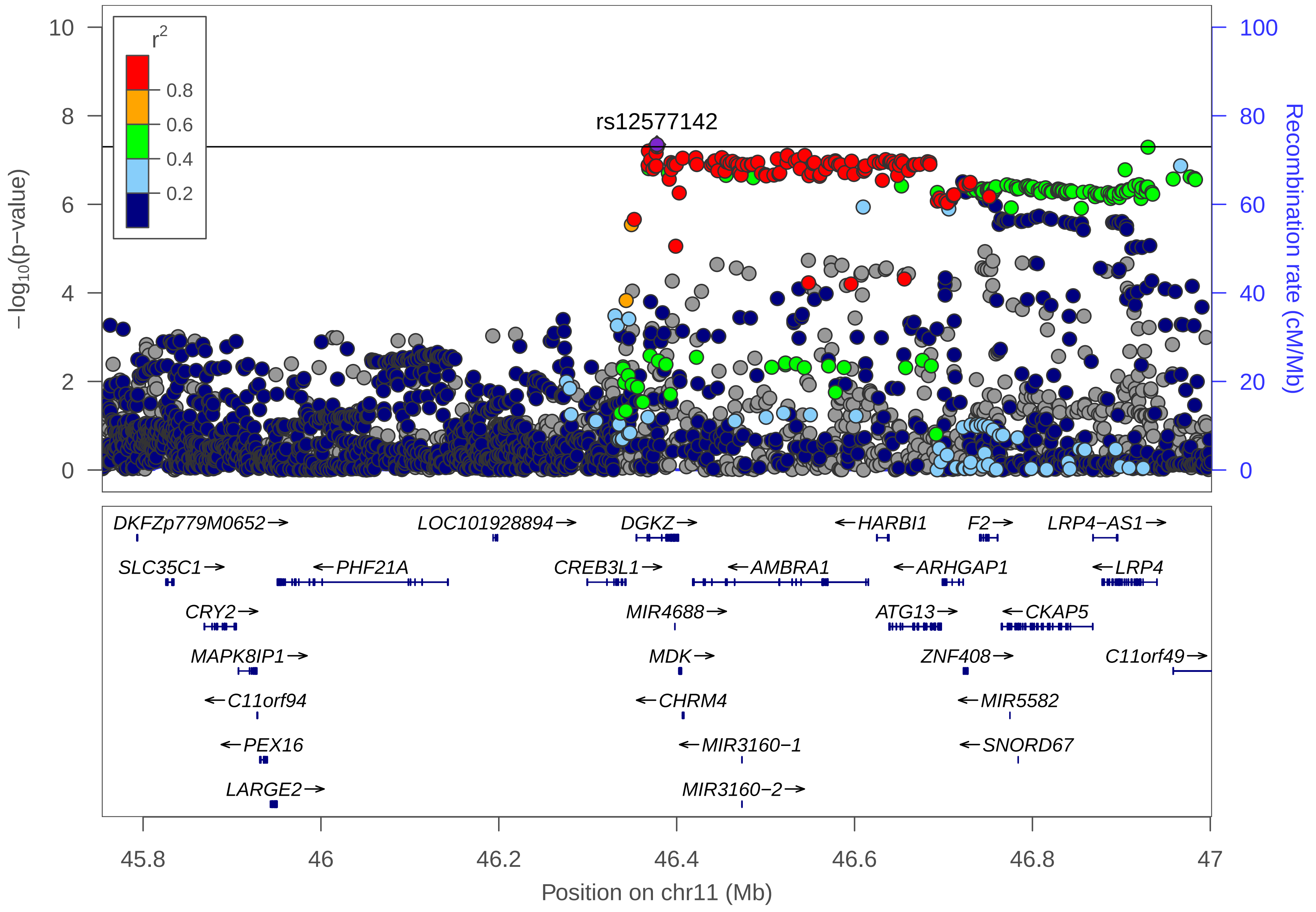

r2
0.8
0.6
0.4
0.2
rs12577142
−log10(p−value)
Recombination rate (cM/Mb)
0 2 4 6 8 10
0 20 40 60 80 100
DKFZp779M0652→
LOC101928894→
DGKZ→
←HARBI1
F2→
LRP4−AS1→
SLC35C1→
←PHF21A
CREB3L1→
←AMBRA1
←ARHGAP1
←LRP4
CRY2→
MIR4688→
ATG13→
←CKAP5
MAPK8IP1→
MDK→
ZNF408→
C11orf49→
←C11orf94
←CHRM4
←MIR5582
←PEX16
←MIR3160−1
←SNORD67
LARGE2→
MIR3160−2→
45.8 46 46.2 46.4 46.6 46.8 47
Position on chr11 (Mb)

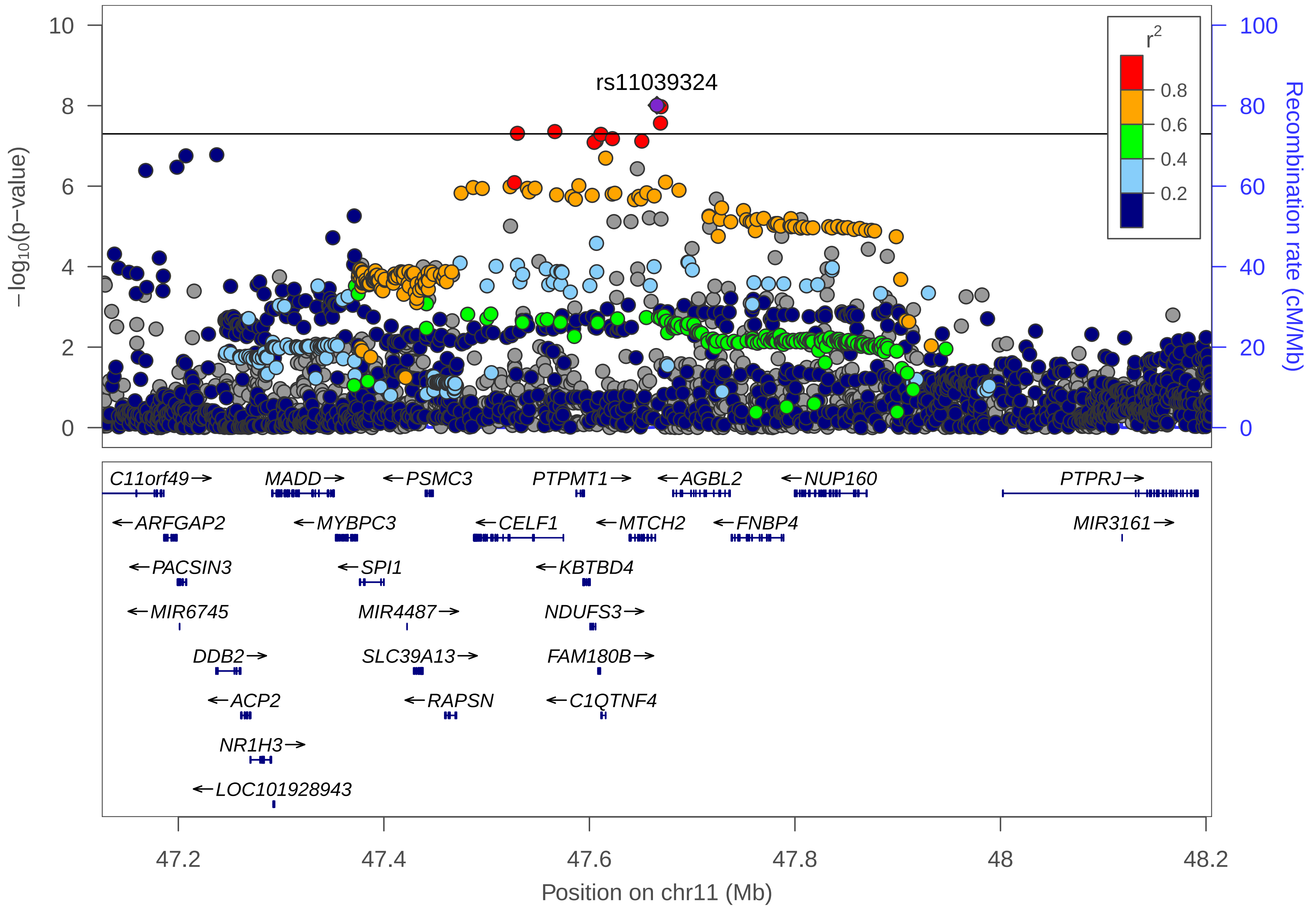

rs11039324
r²
0.8
0.6
0.4
0.2
−log10(p−value)
Recombination rate (cM/Mb)
10
8
6
4
2
0
100
80
60
40
20
0
C11orf49→
MADD→
←PSMC3
PTPMT1→
←AGBL2
←NUP160
PTPRJ→
←ARFGAP2
←MYBPC3
←CELF1
←MTCH2
←FNBP4
MIR3161→
←PACSIN3
←SPI1
←KBTBD4
←MIR6745
MIR4487→
NDUFS3→
DDB2→
SLC39A13→
FAM180B→
←ACP2
←RAPSN
←C1QTNF4
NR1H3→
←LOC101928943
47.2
47.4
47.6
47.8
48
48.2
Position on chr11 (Mb)

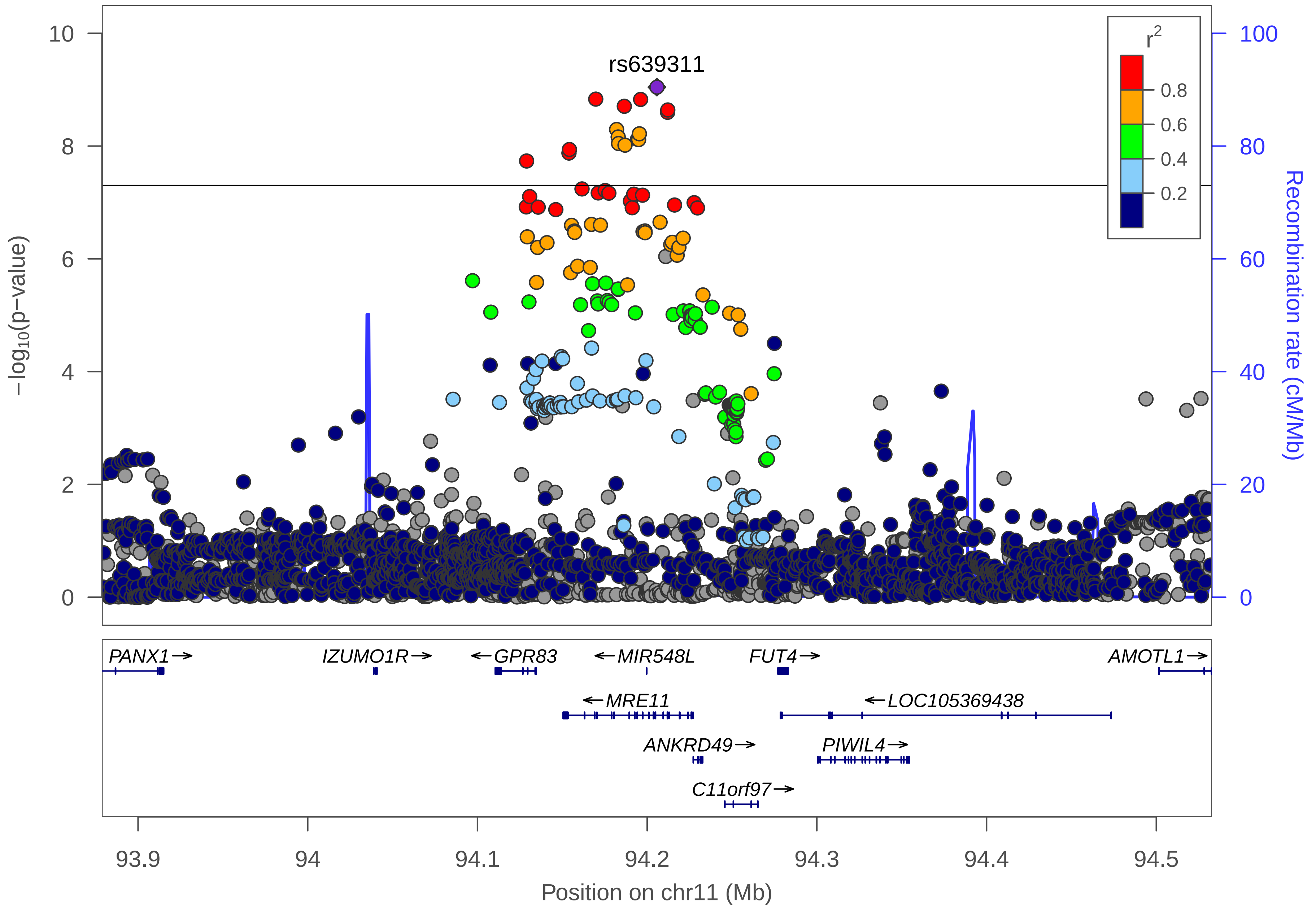
rs639311
r2
0.8
0.6
0.4
0.2
−log10(p−value)
Recombination rate (cM/Mb)
PANX1→
IZUMO1R→
←GPR83
←MIR548L
FUT4→
AMOTL1→
←MRE11
←LOC105369438
ANKRD49→
PIWIL4→
C11orf97→
93.9
94
94.1
94.2
94.3
94.4
94.5
Position on chr11 (Mb)

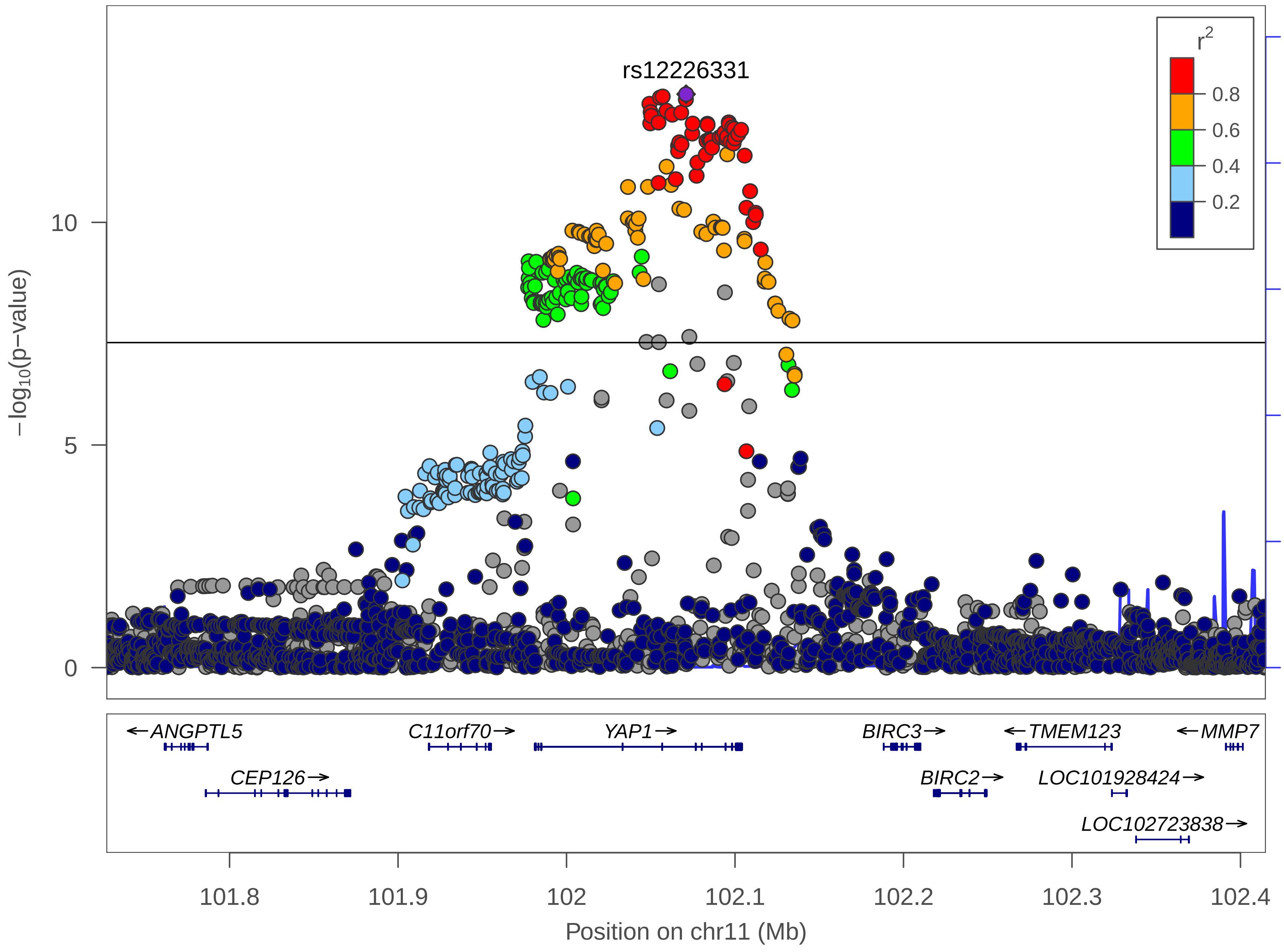

rs12226331
r2
0.8
0.6
0.4
0.2
−log10(p−value)
10
5
0
100
80
60
40
20
0
Recombination rate (cM/Mb)
←ANGPTL5
C11orf70→
YAP1→
BIRC3→
←TMEM123
←MMP7
CEP126→
BIRC2→
LOC101928424→
LOC102723838→
101.8
101.9
102
102.1
102.2
102.3
102.4
Position on chr11 (Mb)

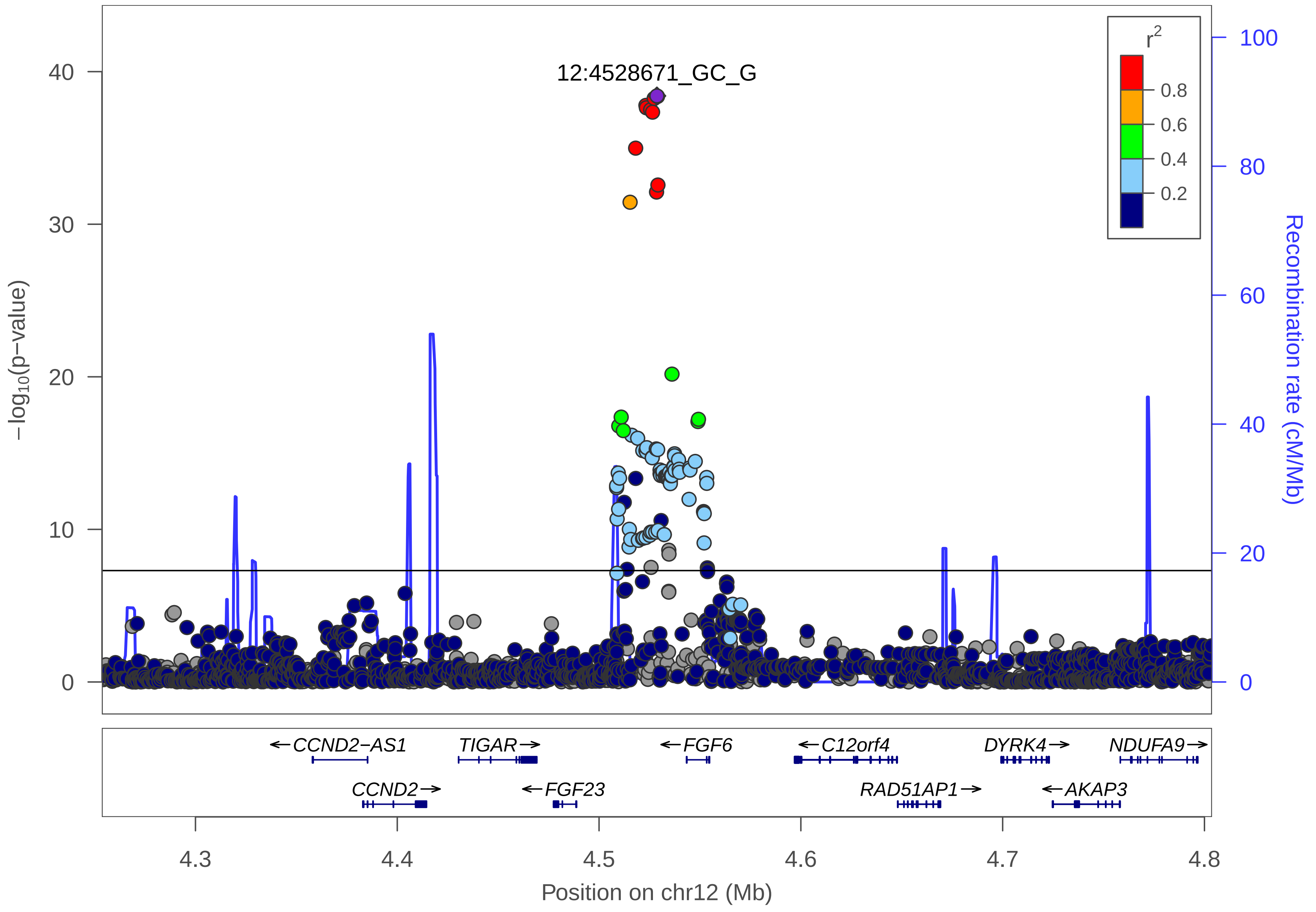

12:4528671_GC_G
r2
0.8
0.6
0.4
0.2
−log10(p−value)
40
30
20
10
0
100
80
60
40
20
0
Recombination rate (cM/Mb)
←CCND2−AS1
TIGAR→
←FGF6
←C12orf4
DYRK4→
NDUFA9→
CCND2→
←FGF23
RAD51AP1→
←AKAP3
4.3
4.4
4.5
4.6
4.7
4.8
Position on chr12 (Mb)

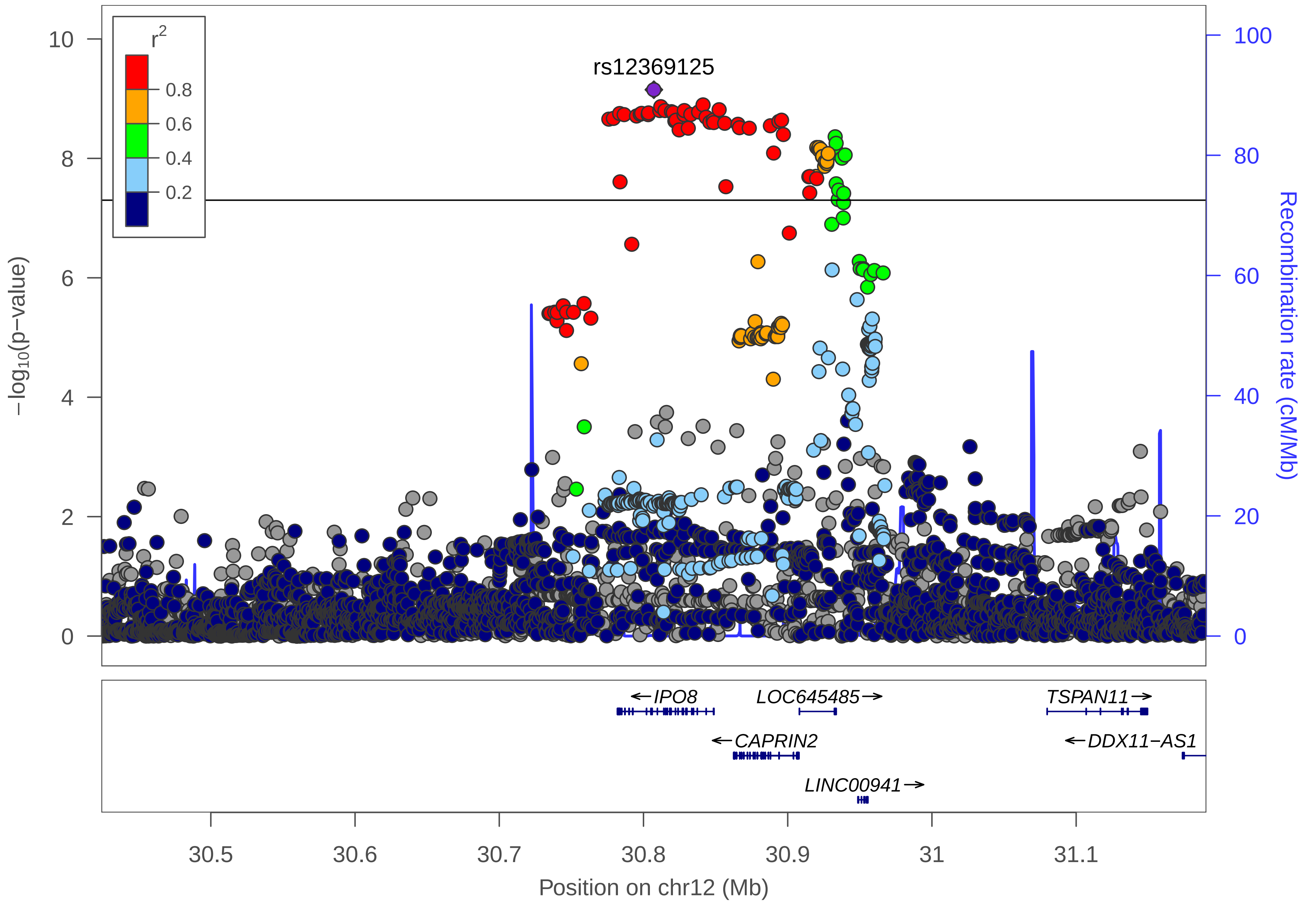
r²
0.8
0.6
0.4
0.2
rs12369125
10
8
6
4
2
0
−log10(p−value)
100
80
60
40
20
0
Recombination rate (cM/Mb)
←IPO8
LOC645485→
TSPAN11→
←CAPRIN2
←DDX11−AS1
LINC00941→
30.5
30.6
30.7
30.8
30.9
31
31.1
Position on chr12 (Mb)

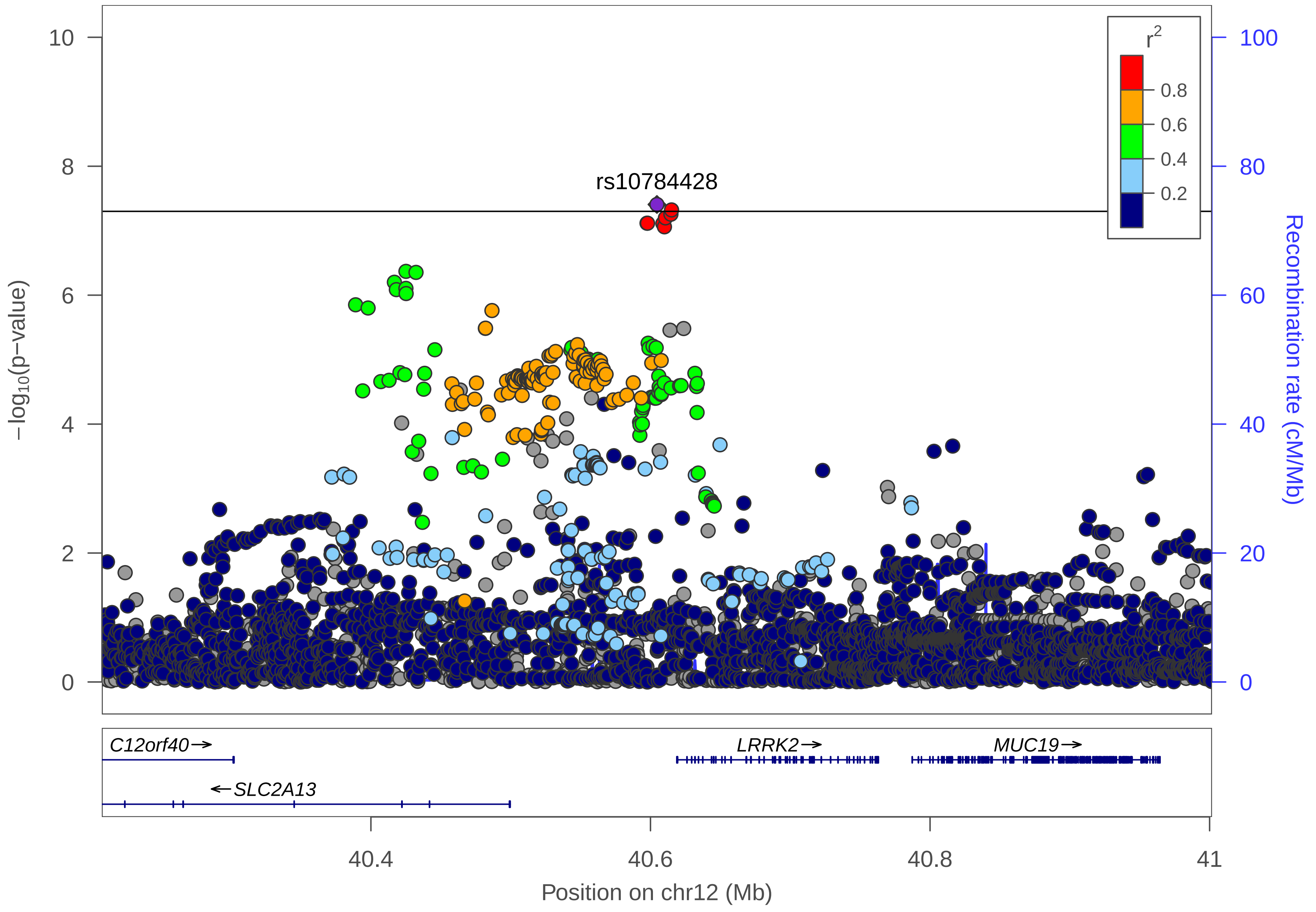
rs10784428
r2
0.8
0.6
0.4
0.2
10
8
6
4
2
0
100
80
60
40
20
0
−log10(p−value)
Recombination rate (cM/Mb)
C12orf40→
LRRK2→
MUC19→
←SLC2A13
40.4
40.6
40.8
41
Position on chr12 (Mb)

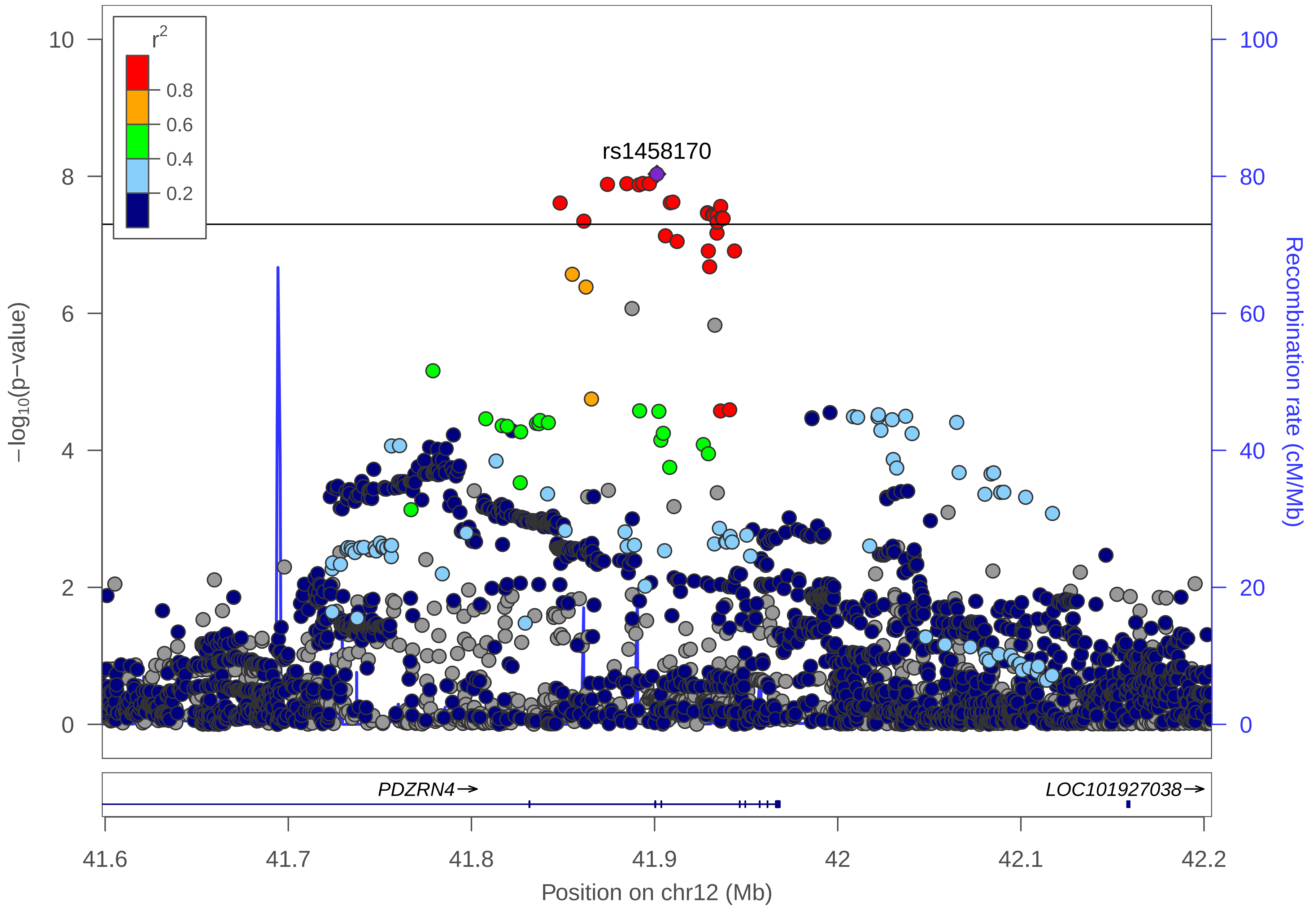

r2
0.8
0.6
0.4
0.2
rs1458170
10
8
6
4
2
0
−log10(p−value)
100
80
60
40
20
0
Recombination rate (cM/Mb)
PDZRN4→
LOC101927038→
41.6
41.7
41.8
41.9
42
42.1
42.2
Position on chr12 (Mb)

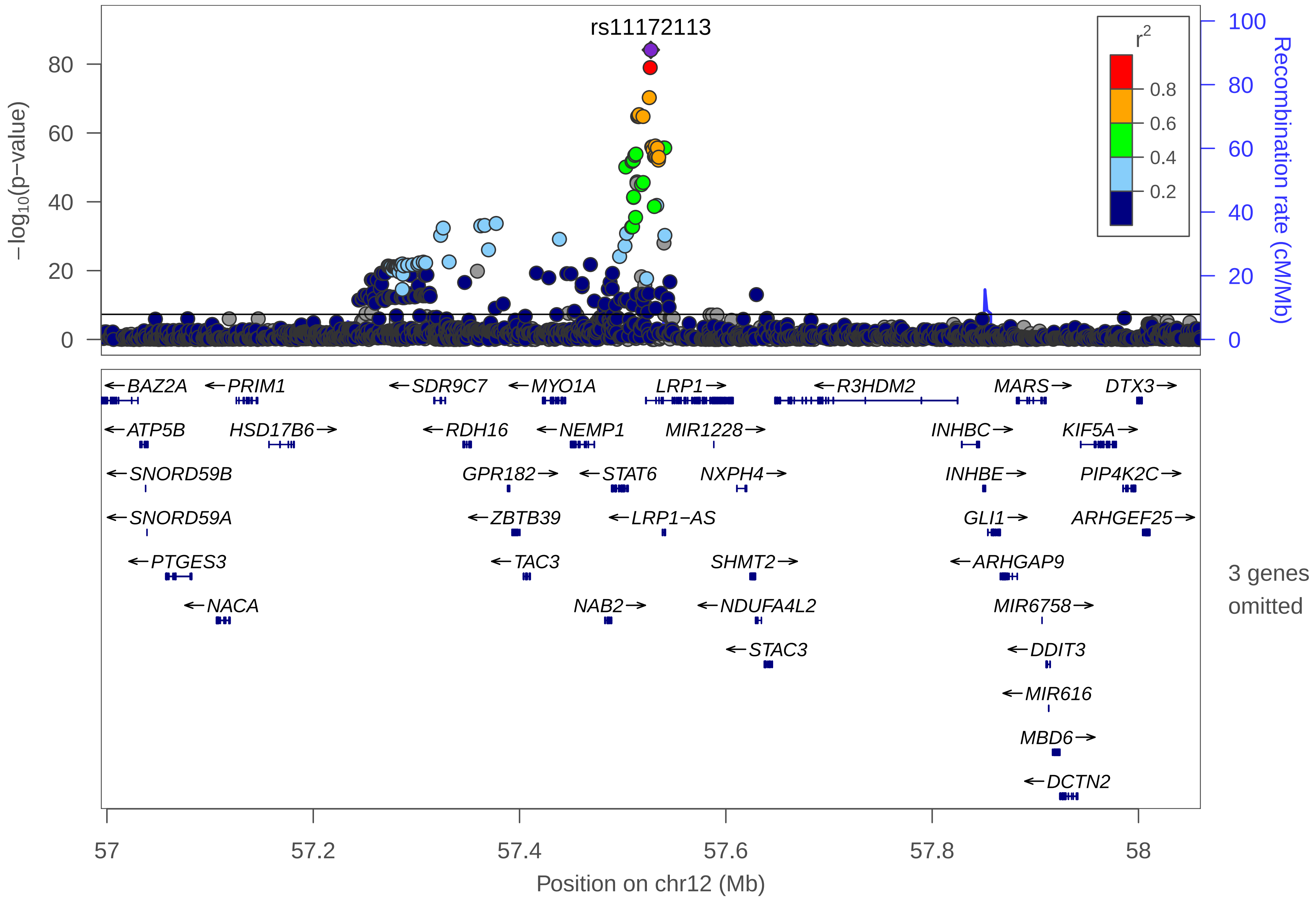

rs11172113
r2
0.8
0.6
0.4
0.2
−log10(p−value)
80
60
40
20
0
Recombination rate (cM/Mb)
100
80
60
40
20
0
←BAZ2A
←PRIM1
←SDR9C7
←MYO1A
LRP1→
←R3HDM2
MARS→
DTX3→
←ATP5B
HSD17B6→
←RDH16
←NEMP1
MIR1228→
INHBC→
KIF5A→
←SNORD59B
GPR182→
←STAT6
NXPH4→
INHBE→
PIP4K2C→
←SNORD59A
←ZBTB39
←LRP1−AS
GLI1→
ARHGEF25→
←PTGES3
←TAC3
SHMT2→
←ARHGAP9
←NACA
NAB2→
←NDUFA4L2
MIR6758→
←STAC3
←DDIT3
←MIR616
MBD6→
←DCTN2
3 genes omitted
57
57.2
57.4
57.6
57.8
58
Position on chr12 (Mb)

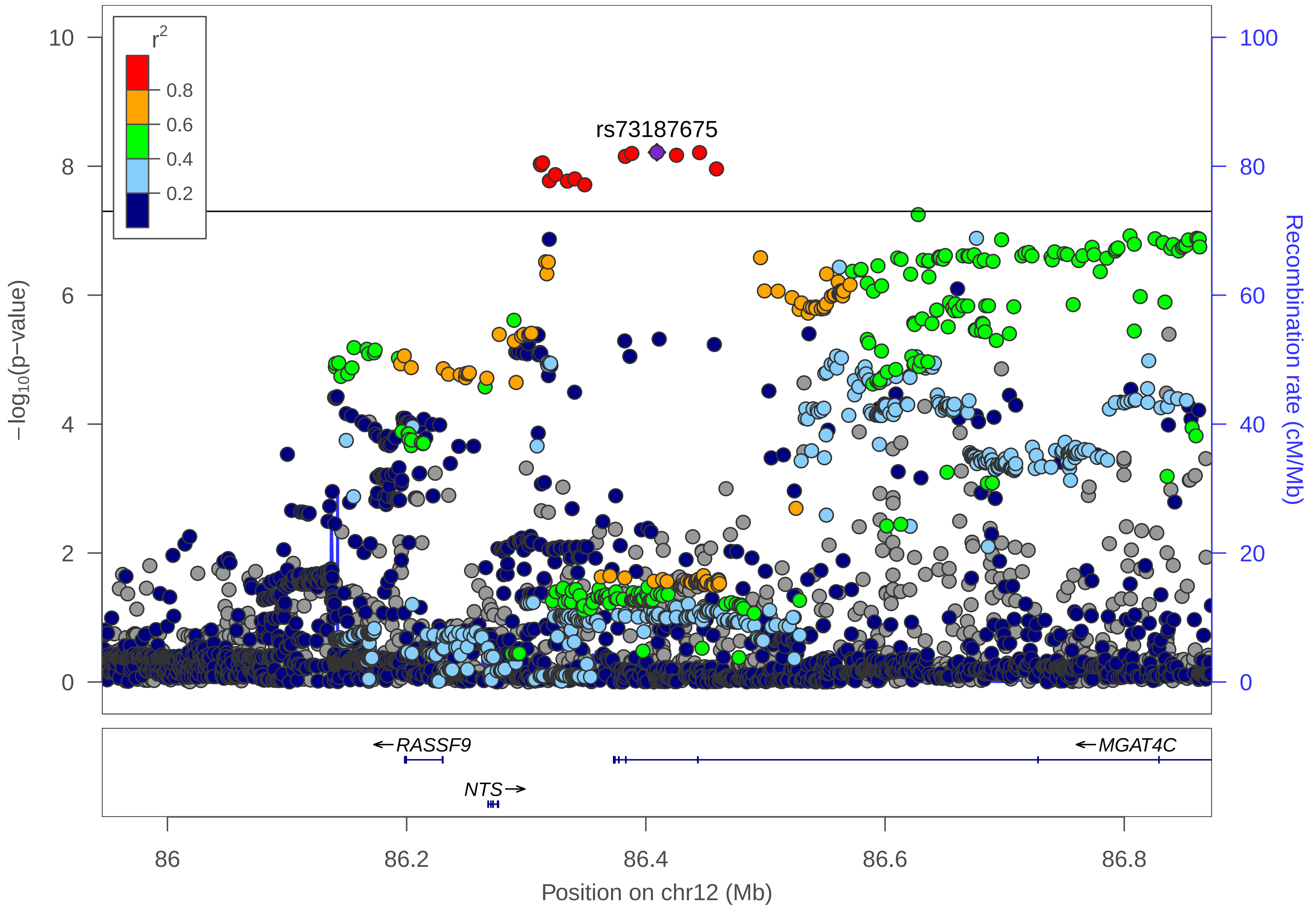

r2
0.8
0.6
0.4
0.2
rs73187675
−log10(p−value)
10
8
6
4
2
0
Recombination rate (cM/Mb)
100
80
60
40
20
0
←RASSF9
NTS→
←MGAT4C
86
86.2
86.4
86.6
86.8
Position on chr12 (Mb)

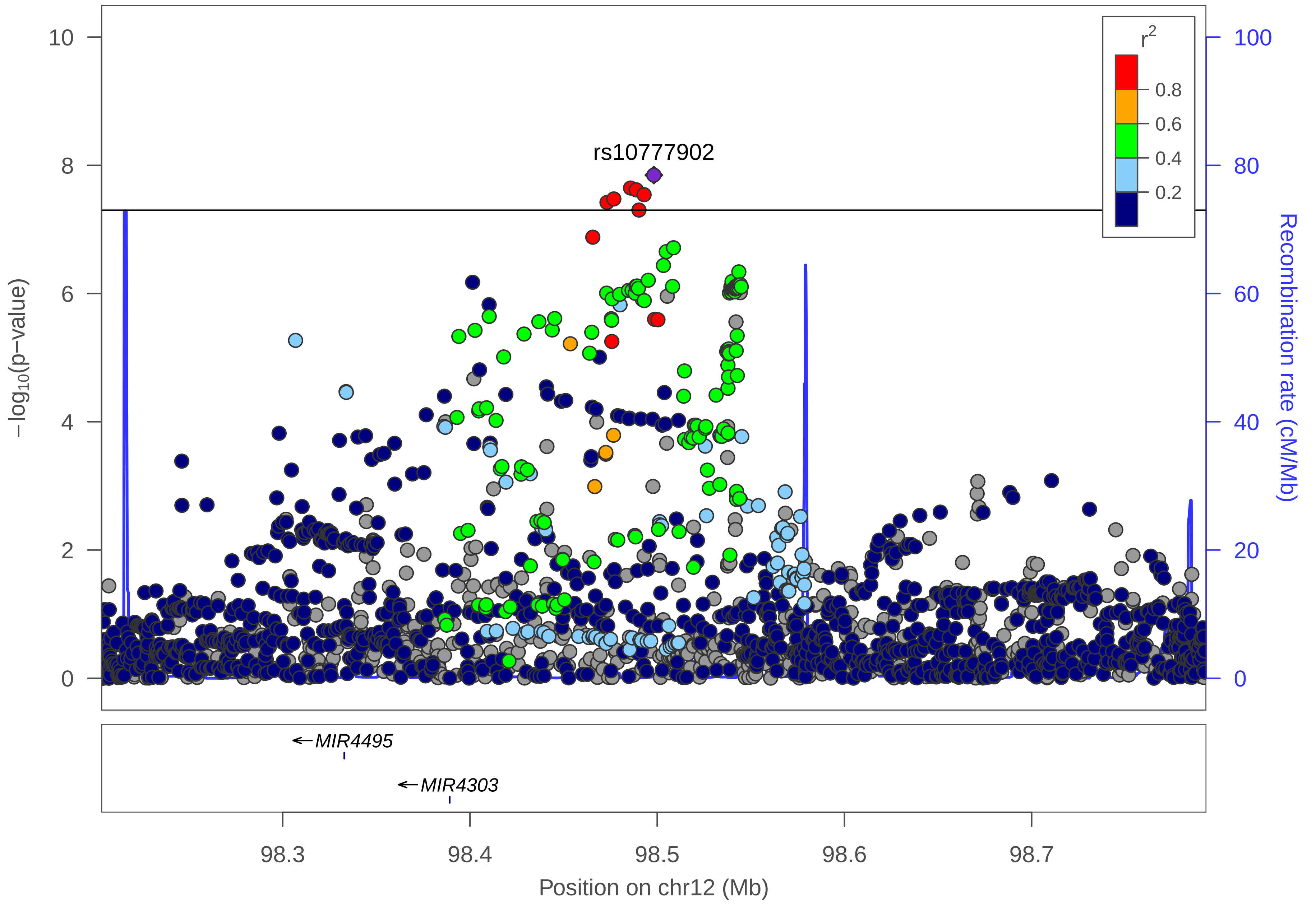

rs10777902
r^2
0.8
0.6
0.4
0.2
10
8
6
4
2
0
100
80
60
40
20
0
−log10(p−value)
Recombination rate (cM/Mb)
MIR4495
MIR4303
98.3
98.4
98.5
98.6
98.7
Position on chr12 (Mb)

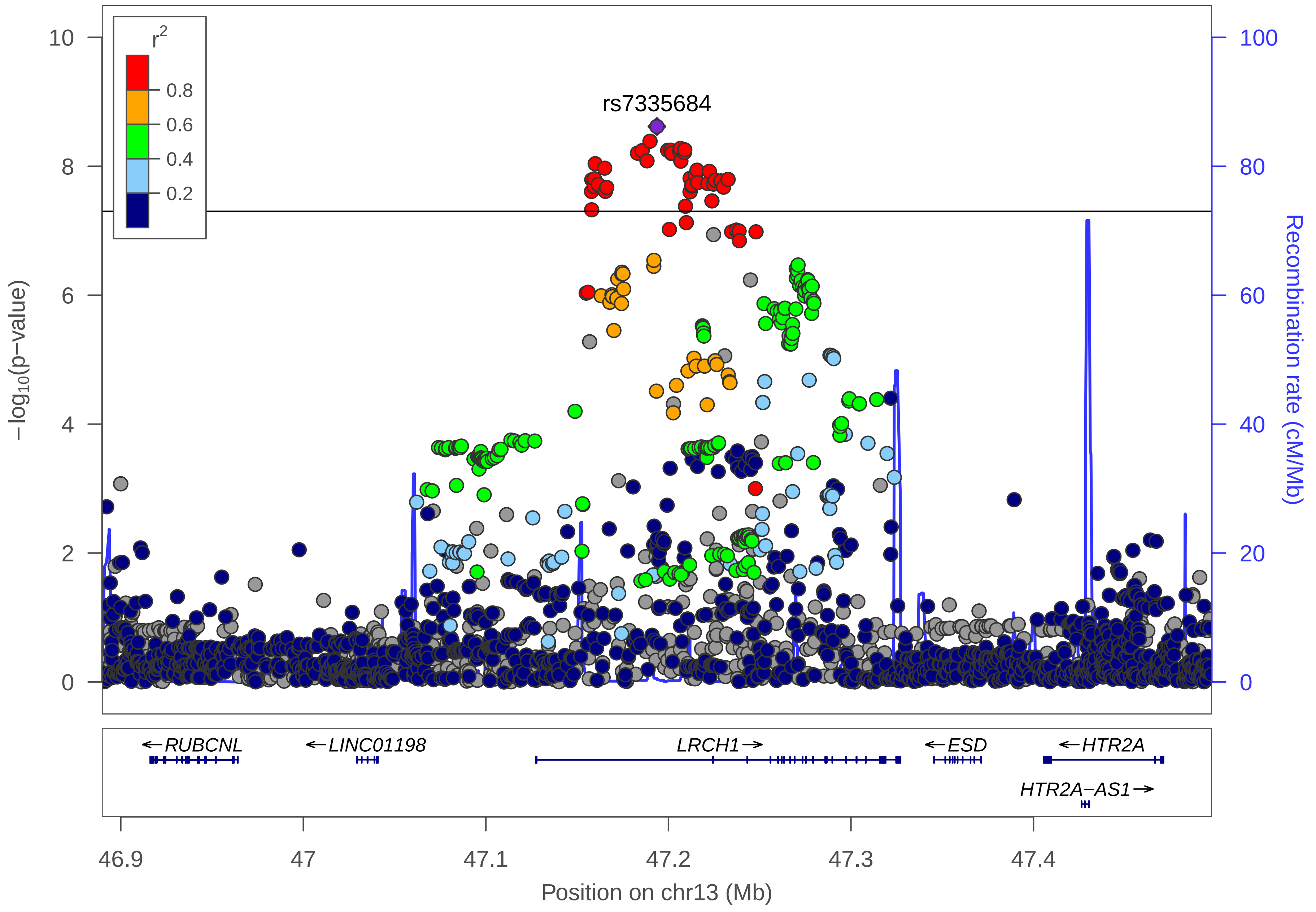

r2
0.8
0.6
0.4
0.2
rs7335684
−log10(p−value)
Recombination rate (cM/Mb)
0
2
4
6
8
10
0
20
40
60
80
100
←RUBCNL
←LINC01198
LRCH1→
←ESD
←HTR2A
HTR2A−AS1→
46.9
47
47.1
47.2
47.3
47.4
Position on chr13 (Mb)

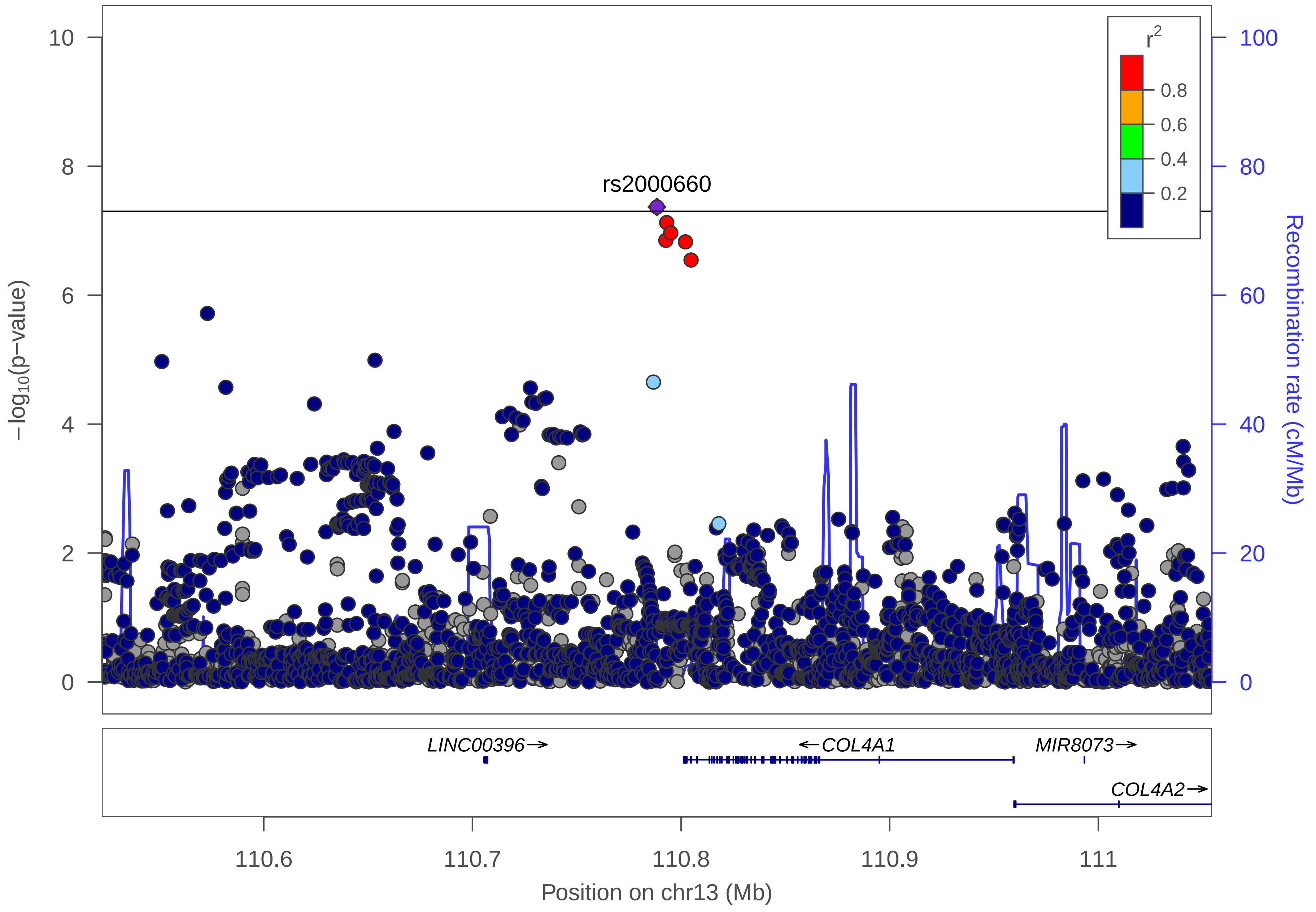

rs2000660
−log10(p−value)
Recombination rate (cM/Mb)
r2
0.8
0.6
0.4
0.2
10
8
6
4
2
0
100
80
60
40
20
0
LINC00396→
←COL4A1
MIR8073→
COL4A2→
110.6
110.7
110.8
110.9
111
Position on chr13 (Mb)

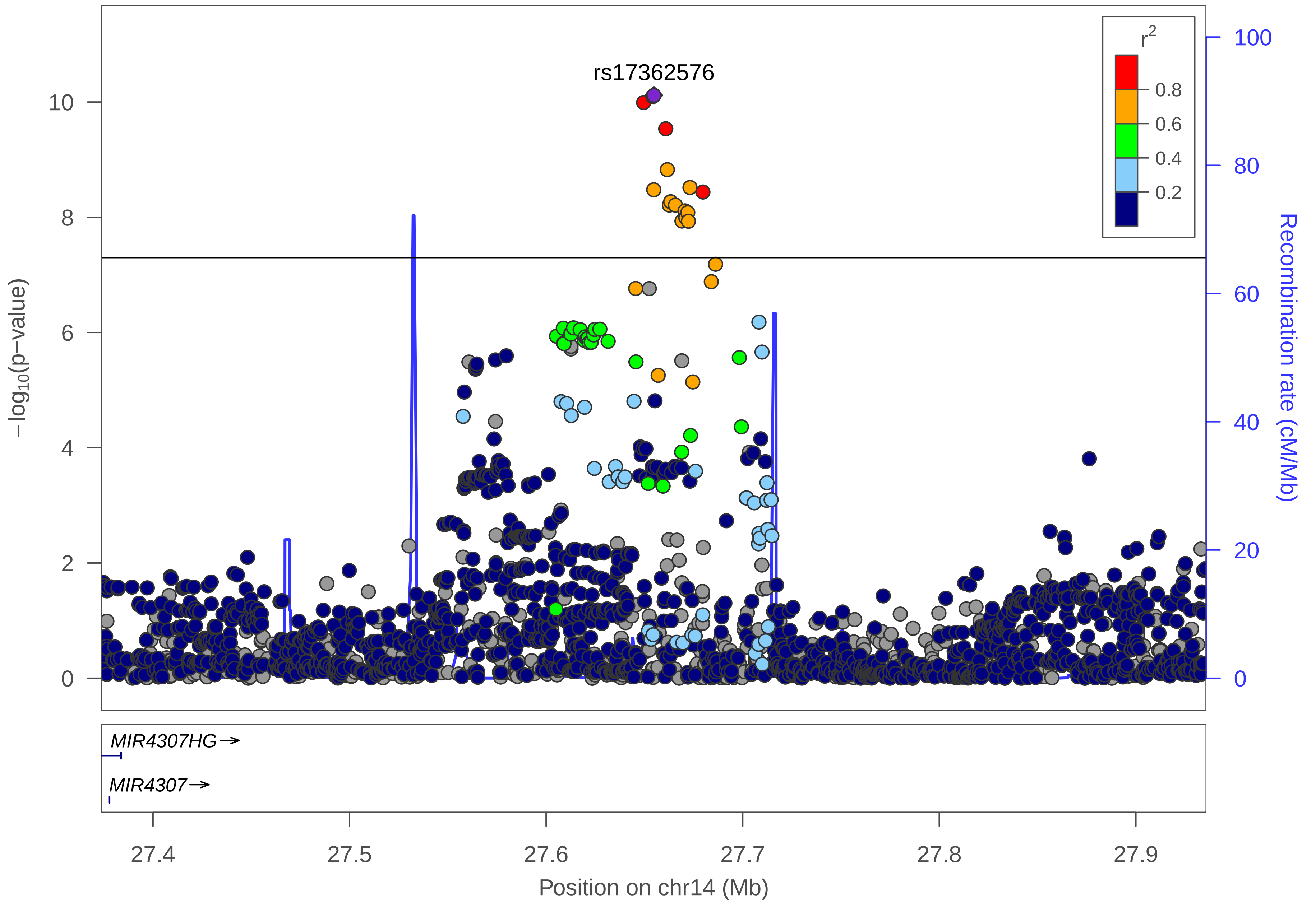
rs17362576
r2
0.8
0.6
0.4
0.2
100
80
60
40
20
0
Recombination rate (cM/Mb)
−log10(p−value)
10
8
6
4
2
0
MIR4307HG→
MIR4307→
27.4
27.5
27.6
27.7
27.8
27.9
Position on chr14 (Mb)

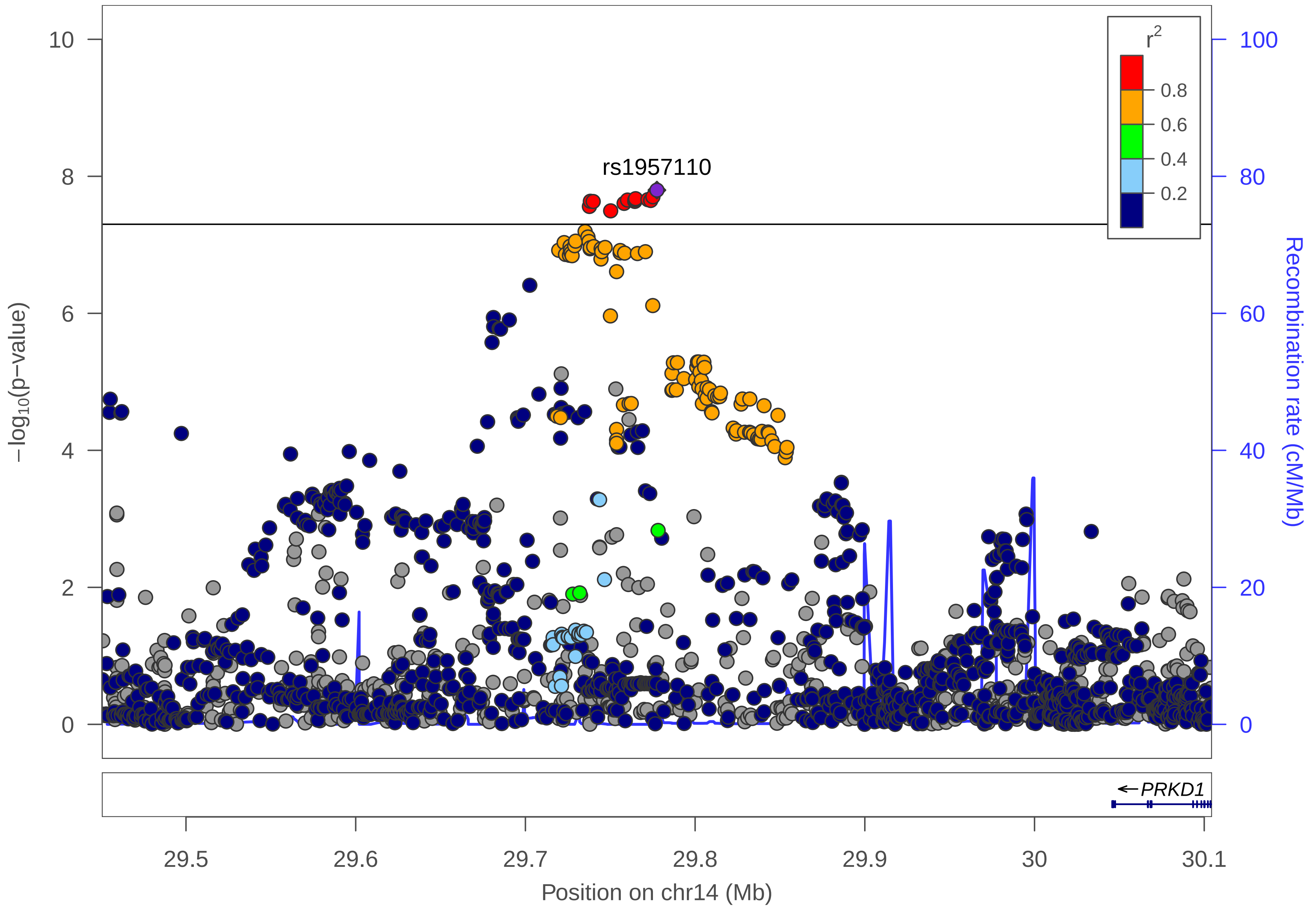
rs1957110
r2
0.8
0.6
0.4
0.2
10
8
6
4
2
0
100
80
60
40
20
0
−log10(p−value)
Recombination rate (cM/Mb)
PRKD1
29.5
29.6
29.7
29.8
29.9
30
30.1
Position on chr14 (Mb)

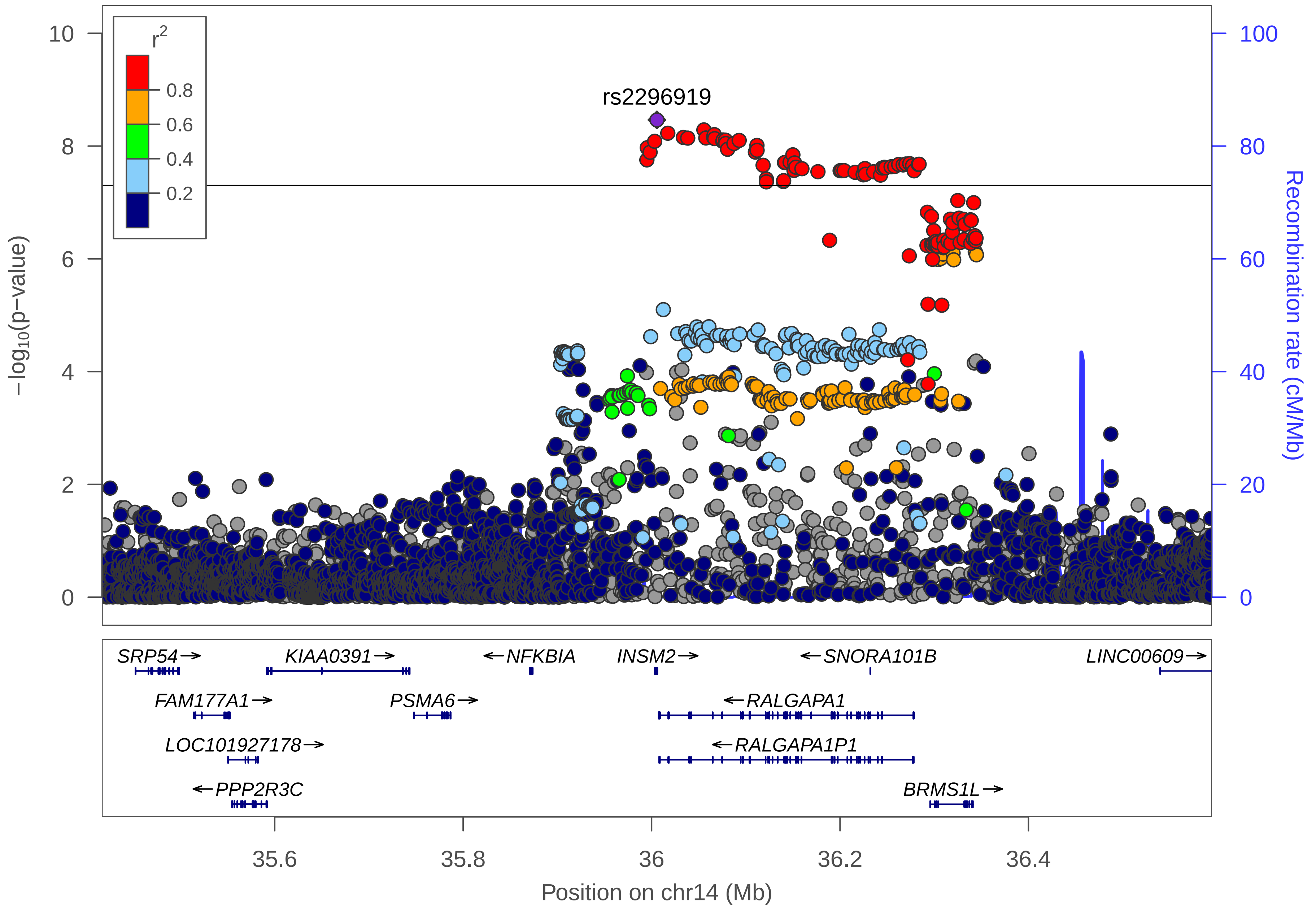
r2
0.8
0.6
0.4
0.2
rs2296919
−log10(p−value)
Recombination rate (cM/Mb)
0
2
4
6
8
10
0
20
40
60
80
100
SRP54→
KIAA0391→
←NFKBIA
INSM2→
←SNORA101B
LINC00609→
FAM177A1→
PSMA6→
←RALGAPA1
LOC101927178→
←RALGAPA1P1
←PPP2R3C
BRMS1L→
35.6
35.8
36
36.2
36.4
Position on chr14 (Mb)

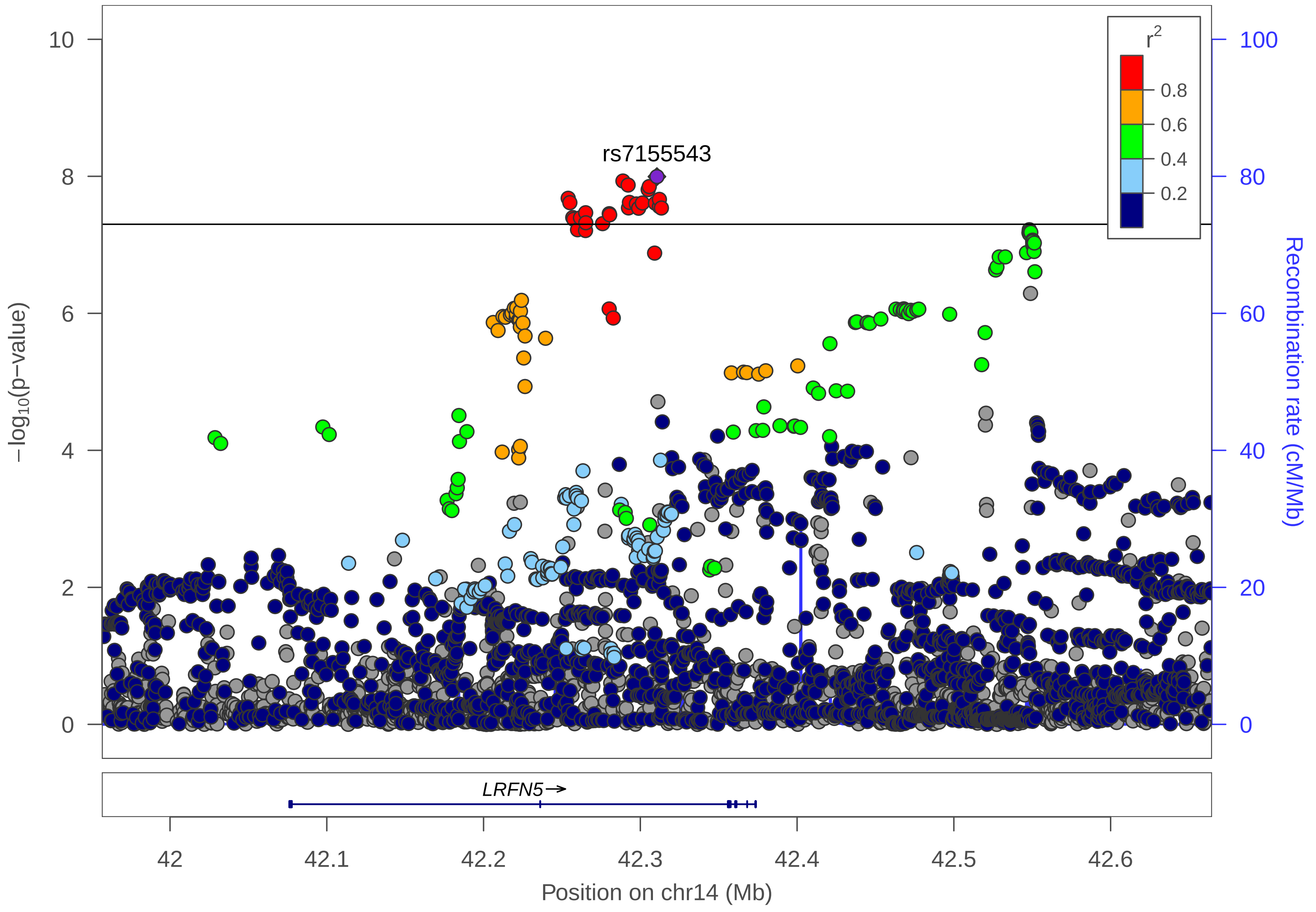

rs7155543
r²
0.8
0.6
0.4
0.2
−log10(p−value)
Recombination rate (cM/Mb)
10
8
6
4
2
0
100
80
60
40
20
0
LRFN5→
42
42.1
42.2
42.3
42.4
42.5
42.6
Position on chr14 (Mb)

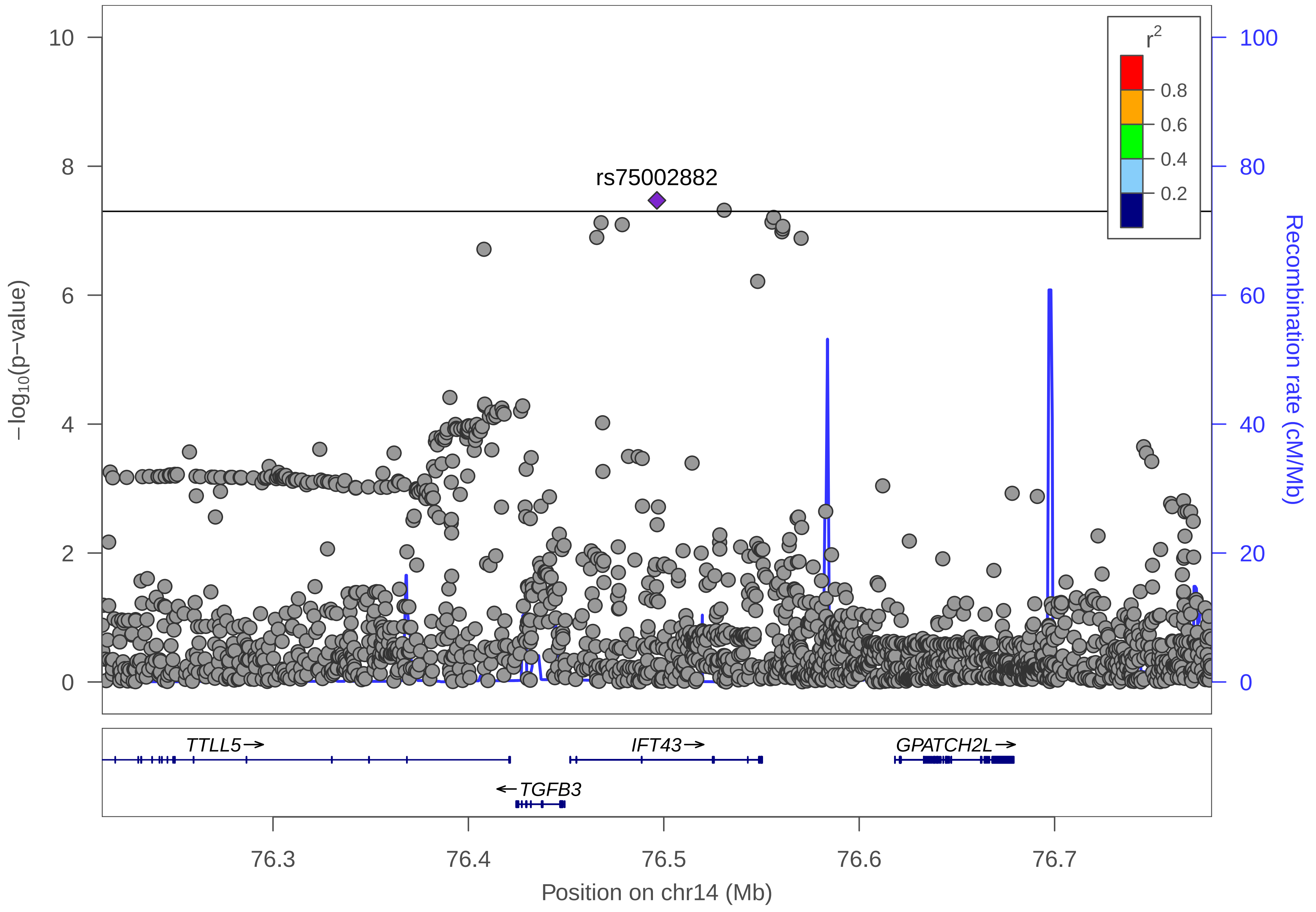

rs75002882
r2
0.8
0.6
0.4
0.2
10
8
6
4
2
0
100
80
60
40
20
0
−log10(p−value)
Recombination rate (cM/Mb)
TTLL5→
IFT43→
GPATCH2L→
←TGFB3
76.3
76.4
76.5
76.6
76.7
Position on chr14 (Mb)

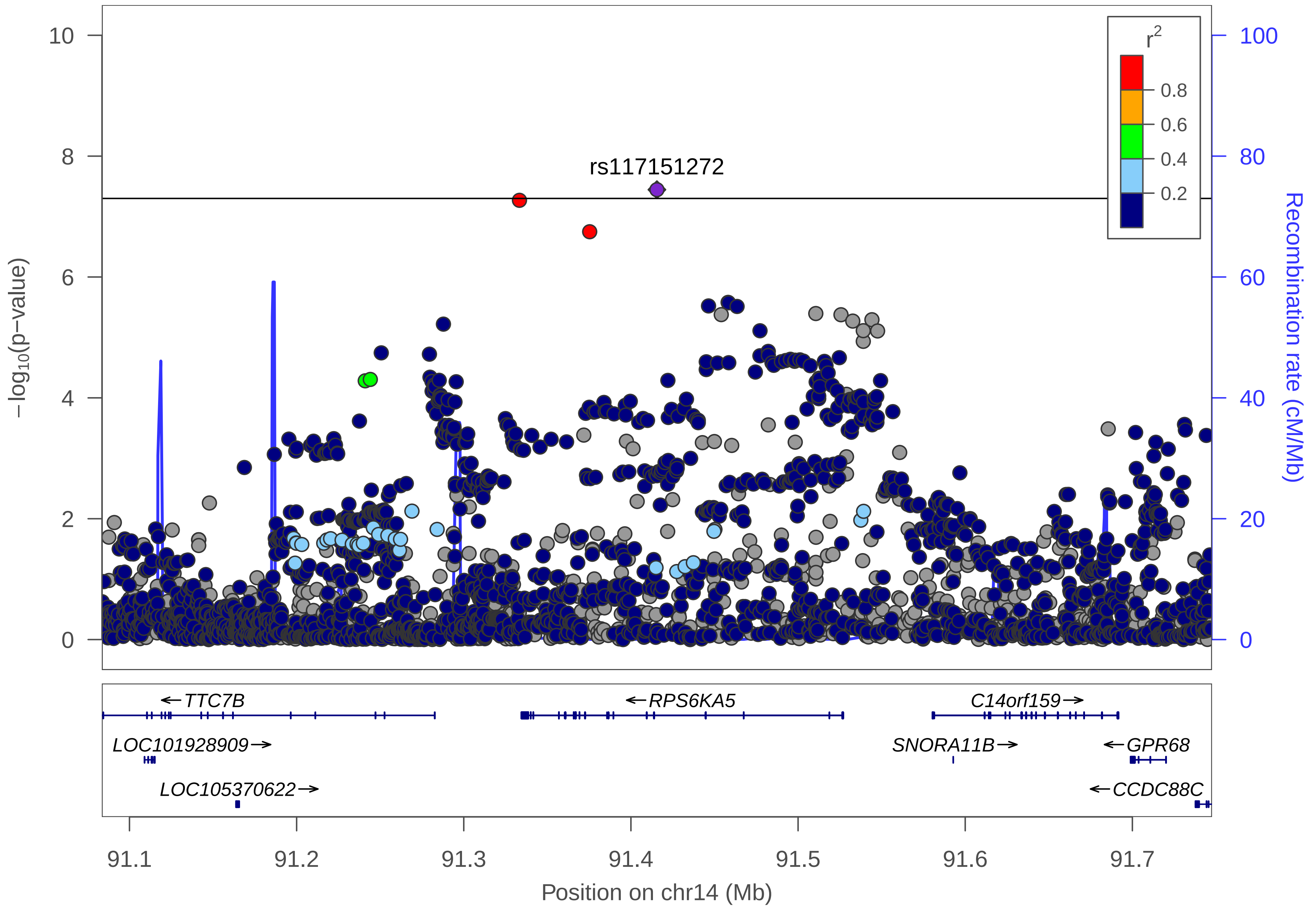

rs117151272
r2
0.8
0.6
0.4
0.2
−log10(p−value)
10
8
6
4
2
0
Recombination rate (cM/Mb)
100
80
60
40
20
0
←TTC7B
←RPS6KA5
C14orf159→
LOC101928909→
SNORA11B→
←GPR68
LOC105370622→
←CCDC88C
91.1
91.2
91.3
91.4
91.5
91.6
91.7
Position on chr14 (Mb)

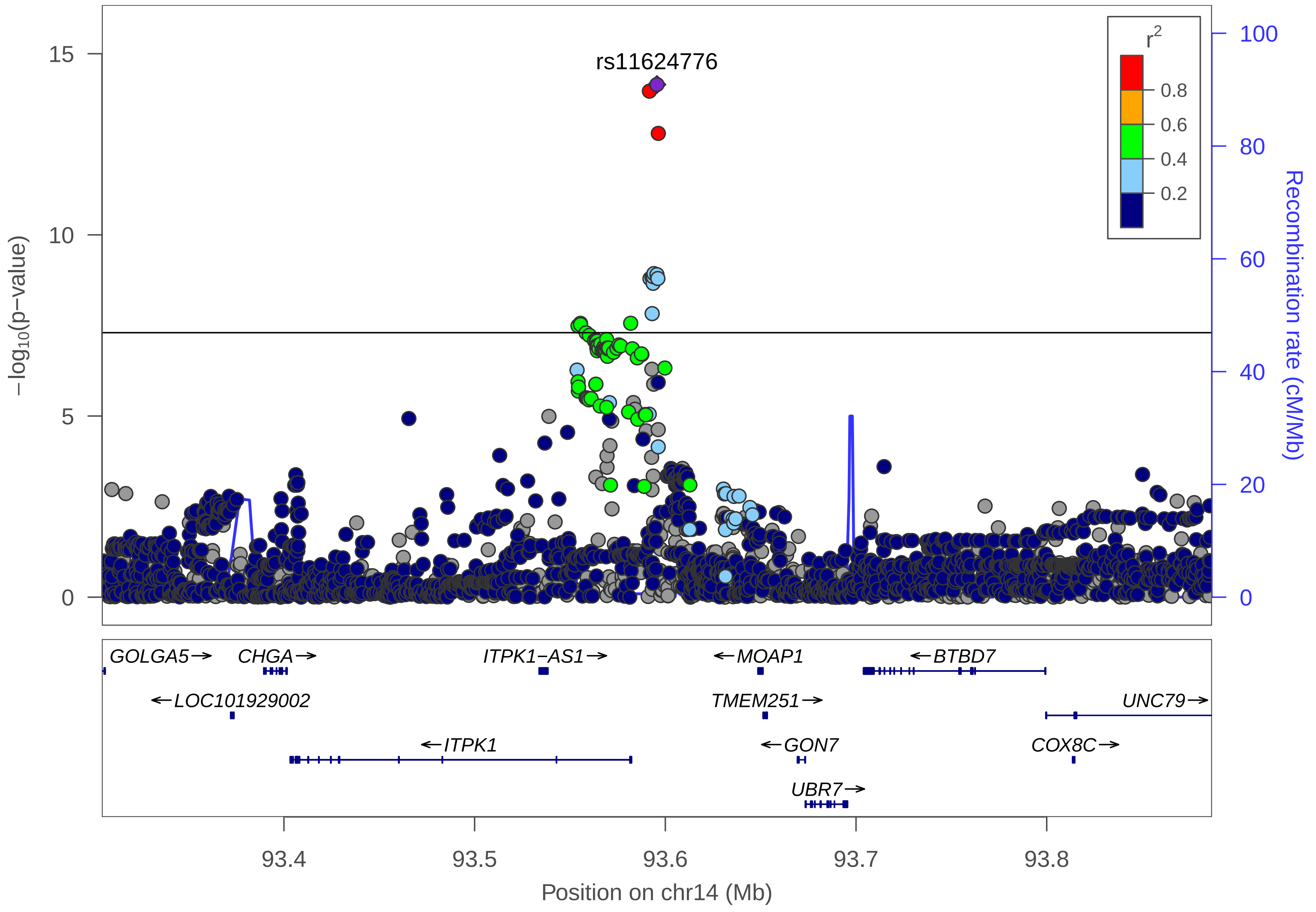
rs11624776
r2
0.8
0.6
0.4
0.2
−log10(p−value)
15
10
5
0
100
80
60
40
20
0
Recombination rate (cM/Mb)
GOLGA5→
CHGA→
ITPK1−AS1→
←MOAP1
←BTBD7
←LOC101929002
TMEM251→
UNC79→
←ITPK1
←GON7
COX8C→
UBR7→
93.4
93.5
93.6
93.7
93.8
Position on chr14 (Mb)

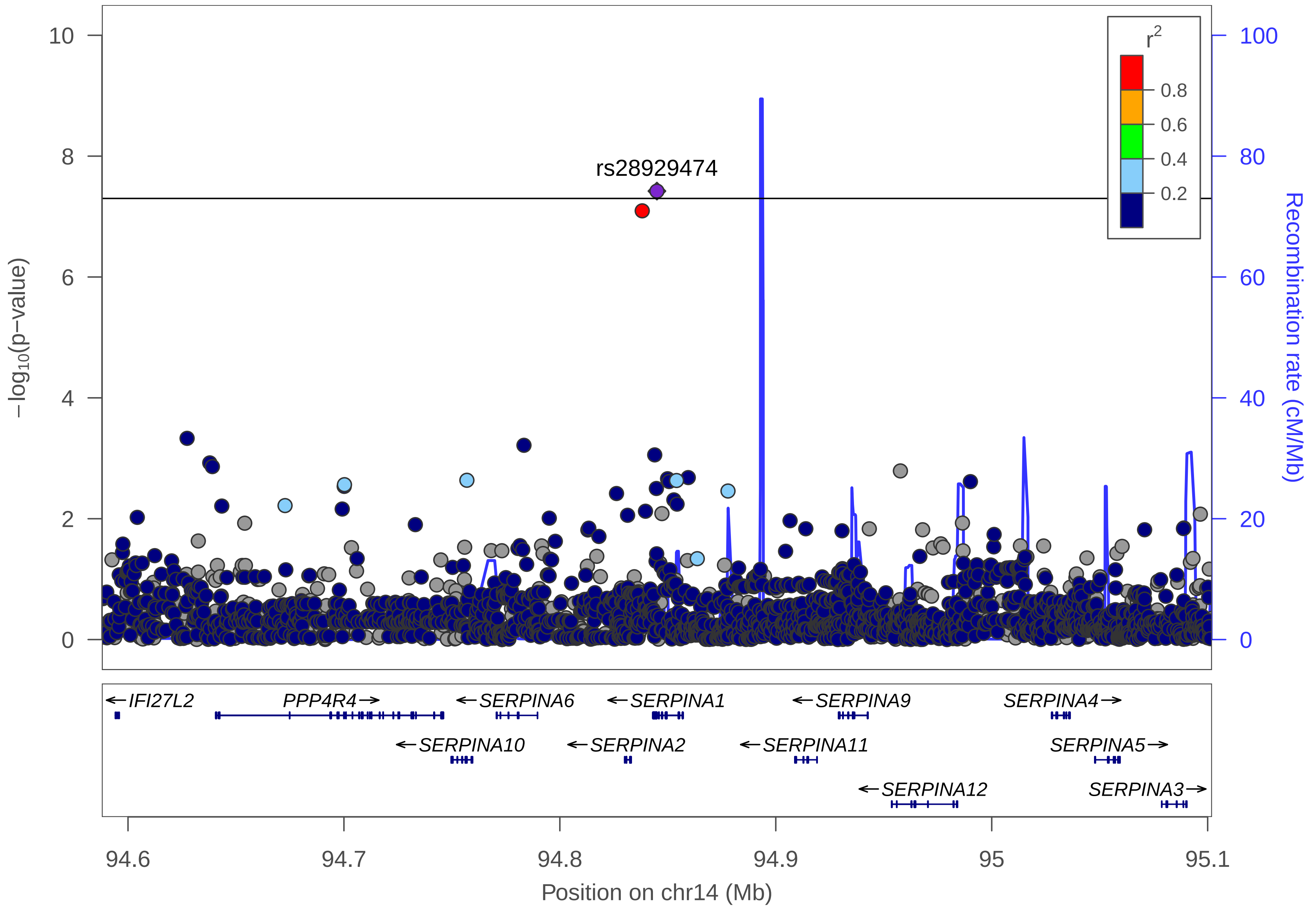
rs28929474
−log10(p−value)
Recombination rate (cM/Mb)
r2
0.8
0.6
0.4
0.2
0
2
4
6
8
10
0
20
40
60
80
100
IFI27L2
PPP4R4
SERPINA6
SERPINA1
SERPINA9
SERPINA4
SERPINA10
SERPINA2
SERPINA11
SERPINA5
SERPINA12
SERPINA3
94.6
94.7
94.8
94.9
95
95.1
Position on chr14 (Mb)

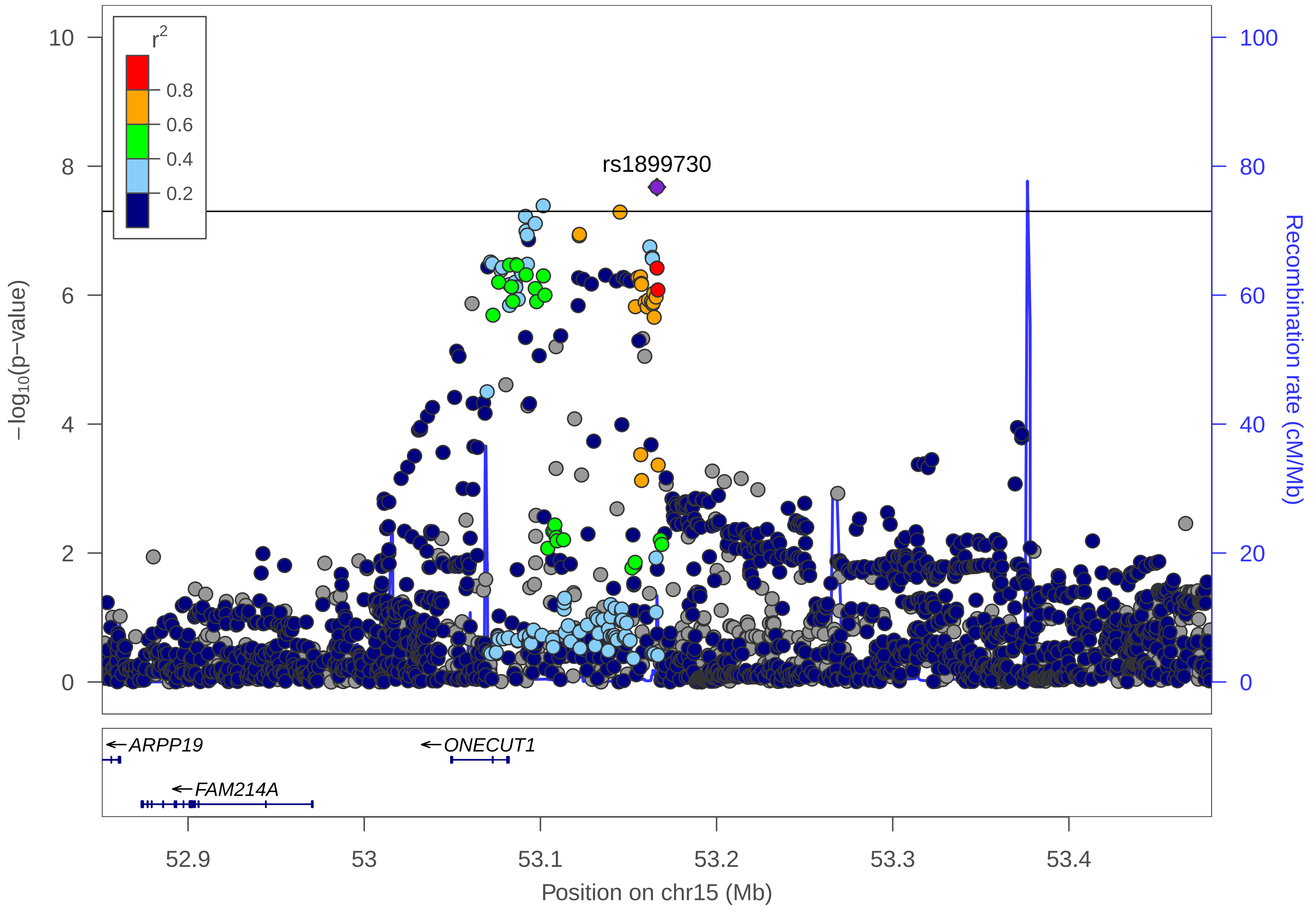

r2
0.8
0.6
0.4
0.2
rs1899730
10
8
6
4
2
0
100
80
60
40
20
0
−log10(p−value)
Recombination rate (cM/Mb)
ARPP19
ONECUT1
FAM214A
52.9
53
53.1
53.2
53.3
53.4
Position on chr15 (Mb)

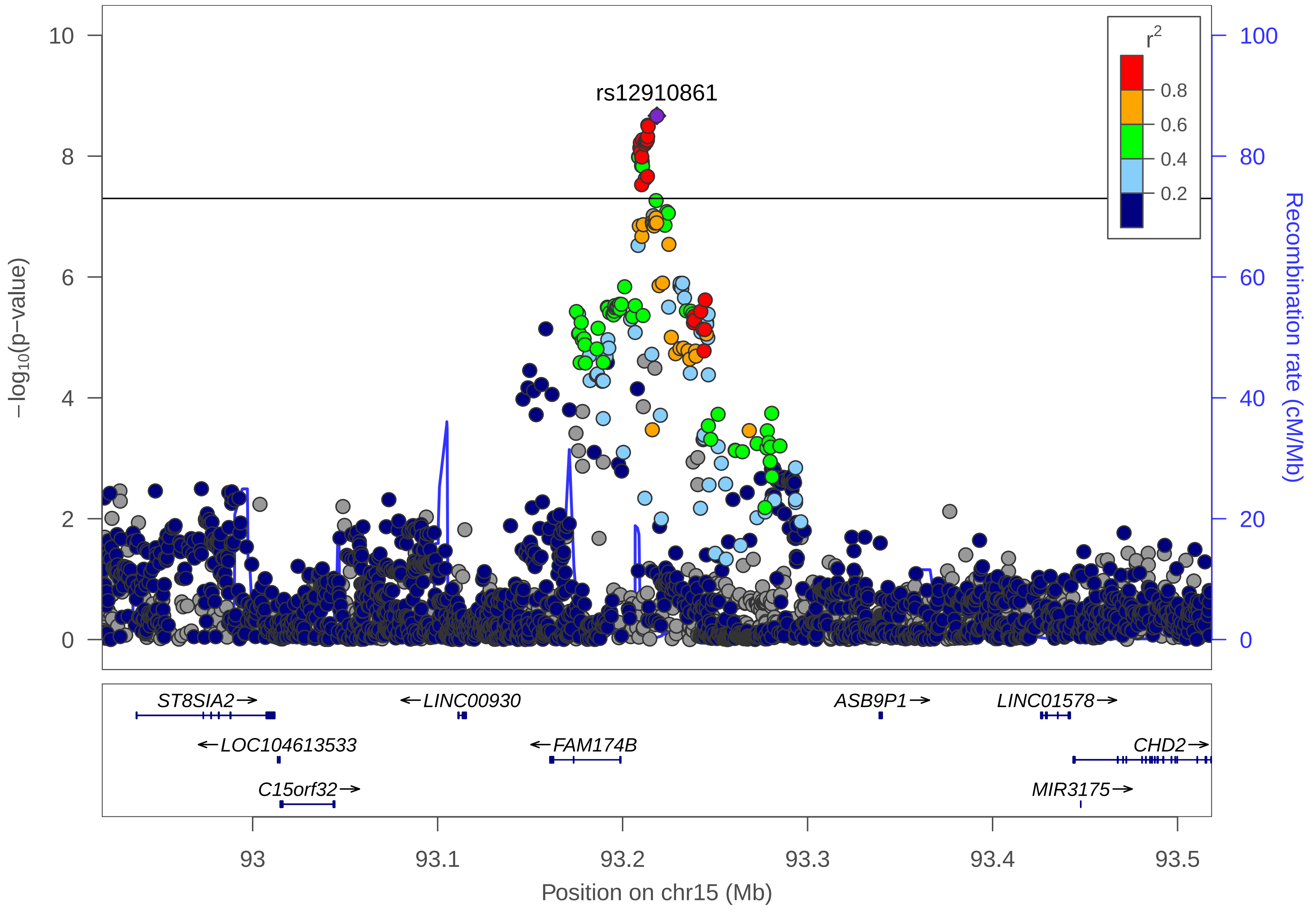
rs12910861
r2
0.8
0.6
0.4
0.2
−log10(p−value)
Recombination rate (cM/Mb)
ST8SIA2→
←LINC00930
ASB9P1→
LINC01578→
←LOC104613533
←FAM174B
CHD2→
C15orf32→
MIR3175→
Position on chr15 (Mb)

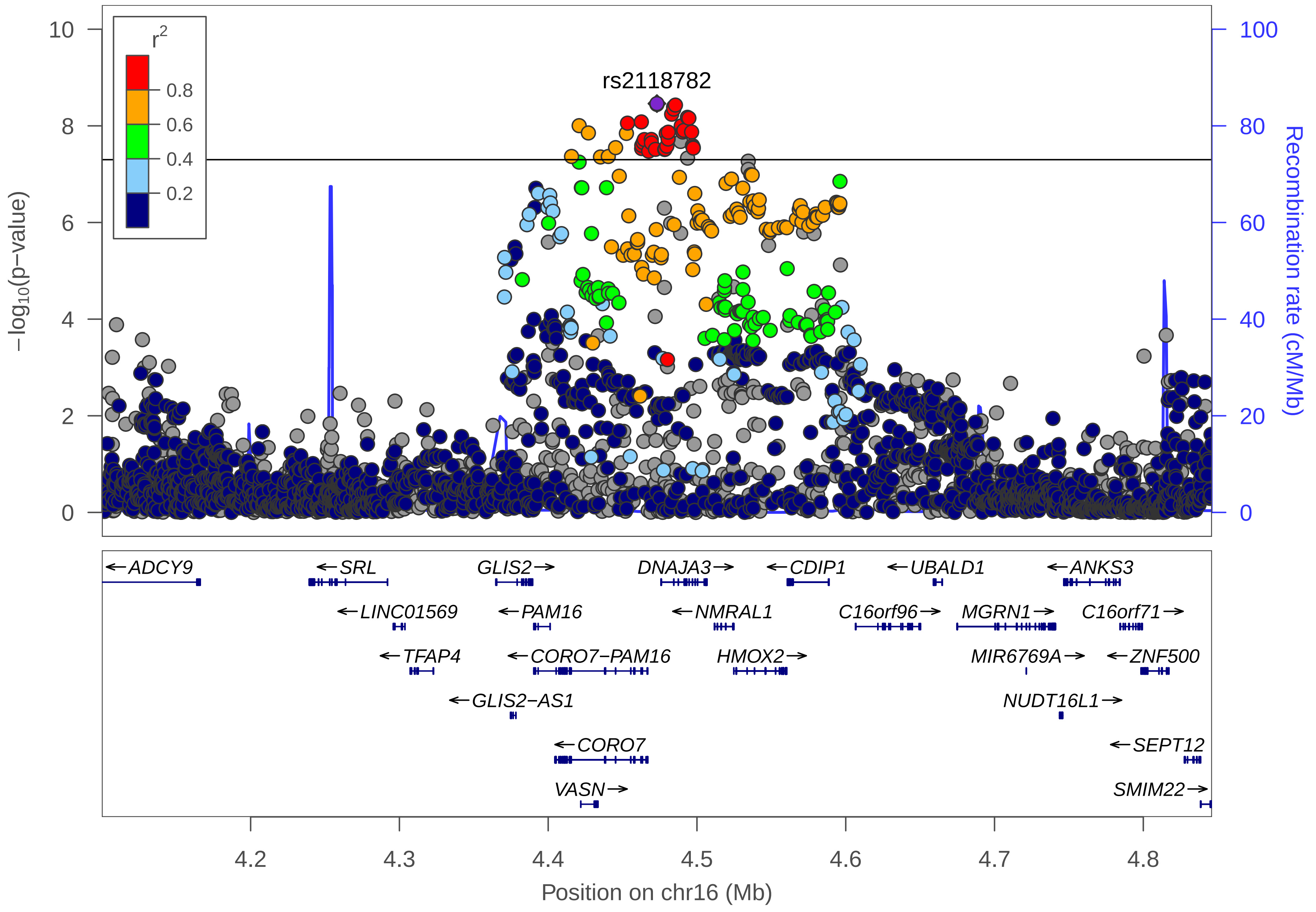
rs2118782
r2
0.8
0.6
0.4
0.2
10
8
6
4
2
0
−log10(p−value)
100
80
60
40
20
0
Recombination rate (cM/Mb)
ADCY9
SRL
GLIS2
DNAJA3
CDIP1
UBALD1
ANKS3
LINC01569
PAM16
NMRAL1
C16orf96
MGRN1
C16orf71
TFAP4
CORO7−PAM16
HMOX2
MIR6769A
ZNF500
GLIS2−AS1
NUDT16L1
CORO7
SEPT12
VASN
SMIM22
4.2
4.3
4.4
4.5
4.6
4.7
4.8
Position on chr16 (Mb)

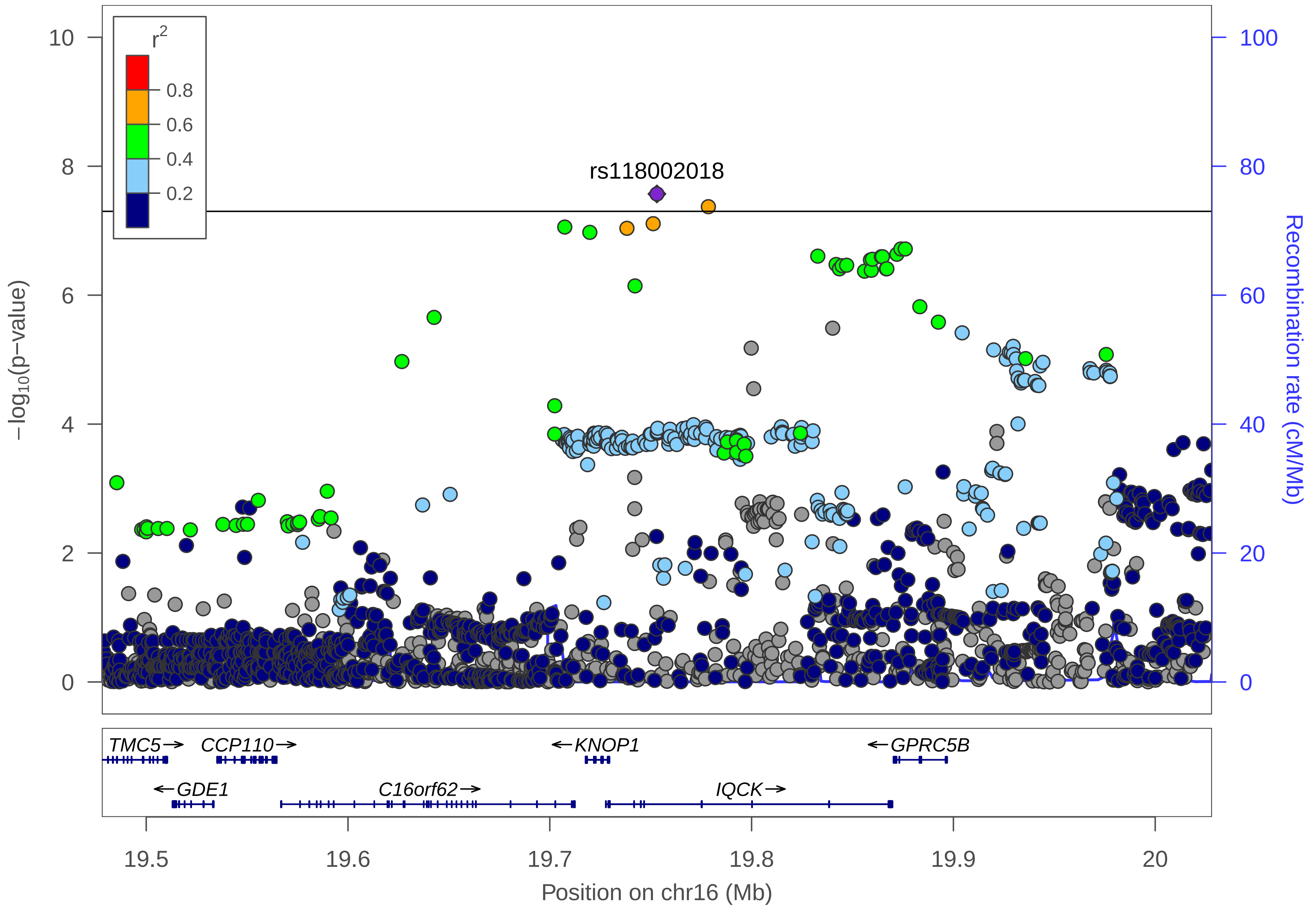

r²
0.8
0.6
0.4
0.2
rs118002018
−log10(p−value)
Recombination rate (cM/Mb)
10
8
6
4
2
0
100
80
60
40
20
0
TMC5→
CCP110→
←KNOP1
←GPRC5B
←GDE1
C16orf62→
IQCK→
19.5
19.6
19.7
19.8
19.9
20
Position on chr16 (Mb)

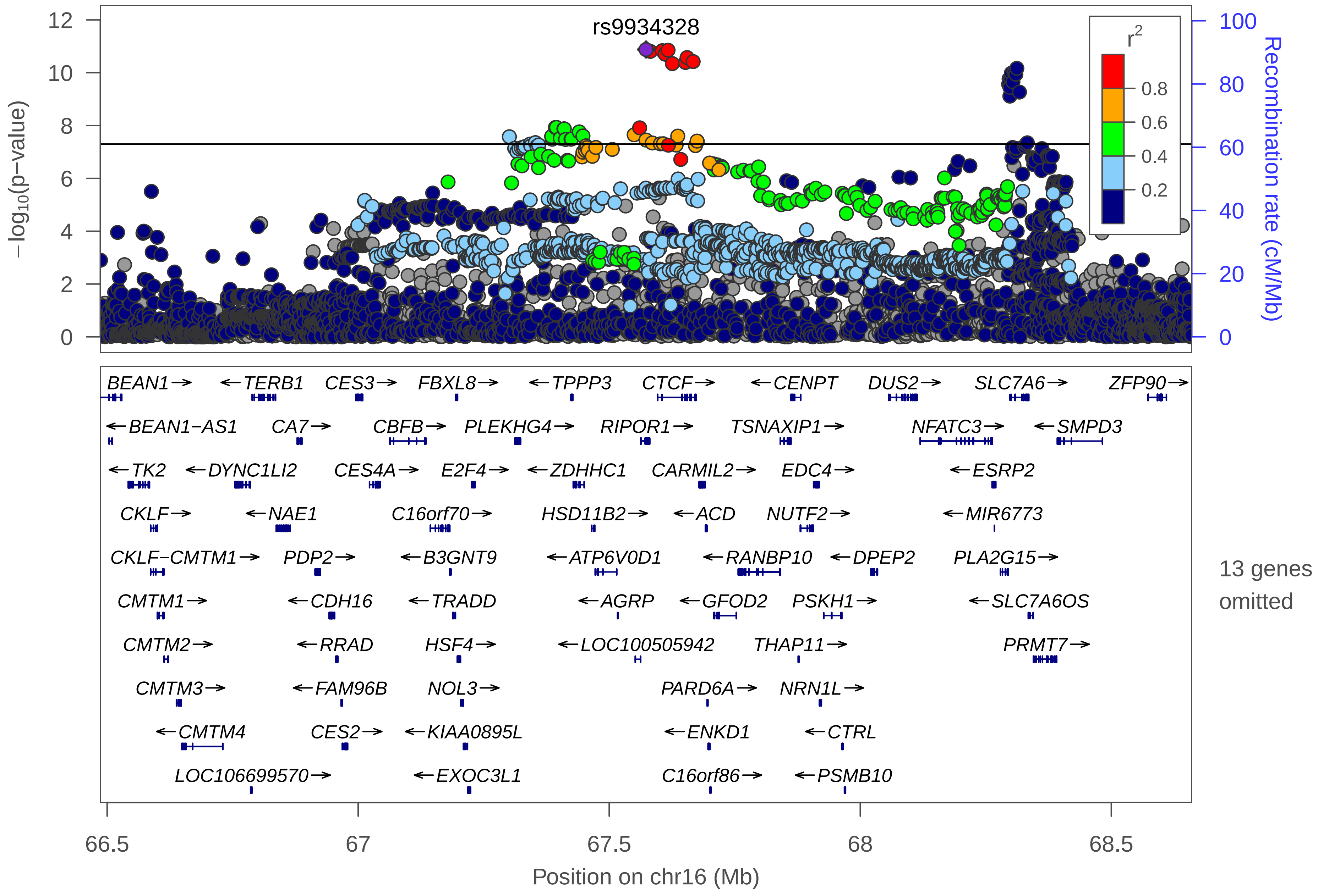

rs9934328
r2
0.8
0.6
0.4
0.2
−log10(p−value)
Recombination rate (cM/Mb)
BEAN1→ ←TERB1 CES3→ FBXL8→ ←TPPP3 CTCF→ ←CENPT DUS2→ SLC7A6→ ZFP90→
←BEAN1−AS1 CA7→ CBFB→ PLEKHG4→ RIPOR1→ TSNAXIP1→ NFATC3→ ←SMPD3
←TK2 ←DYNC1LI2 CES4A→ E2F4→ ←ZDHHC1 CARMIL2→ EDC4→ ←ESRP2
CKLF→ ←NAE1 C16orf70→ HSD11B2→ ←ACD NUTF2→ ←MIR6773
CKLF−CMTM1→ PDP2→ ←B3GNT9 ←ATP6V0D1 ←RANBP10 ←DPEP2 PLA2G15→
CMTM1→ ←CDH16 ←TRADD ←AGRP ←GFOD2 PSKH1→ ←SLC7A6OS
CMTM2→ ←RRAD HSF4→ ←LOC100505942 THAP11→ PRMT7→
CMTM3→ ←FAM96B NOL3→ PARD6A→ NRN1L→
←CMTM4 CES2→ ←KIAA0895L ←ENKD1 ←CTRL
LOC106699570→ ←EXOC3L1 C16orf86→ ←PSMB10
13 genes omitted
66.5
67
67.5
68
68.5
Position on chr16 (Mb)

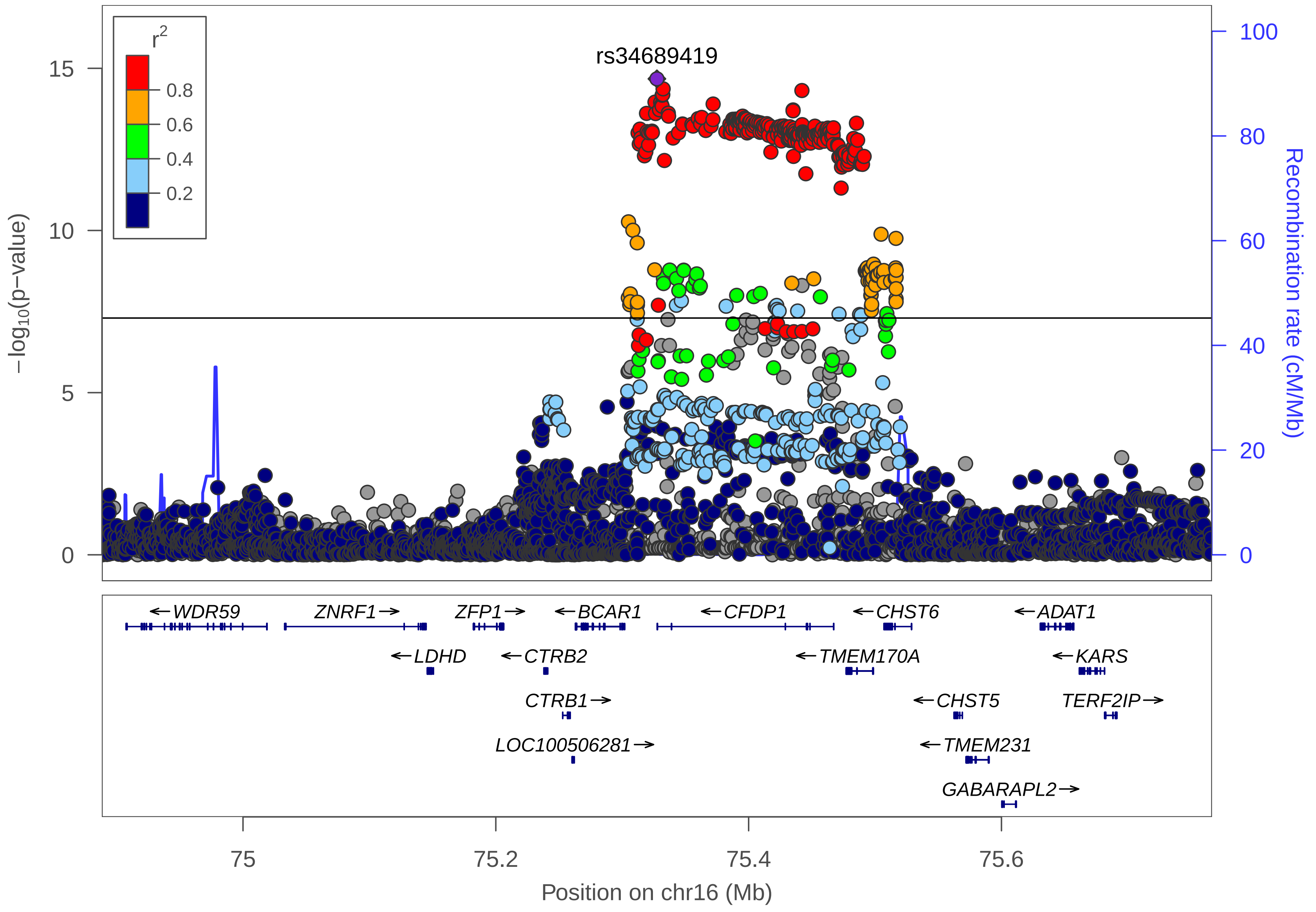

rs34689419
r2
0.8
0.6
0.4
0.2
−log10(p−value)
Recombination rate (cM/Mb)
WDR59
ZNRF1
ZFP1
BCAR1
CFDP1
CHST6
ADAT1
LDHD
CTRB2
TMEM170A
KARS
CTRB1
CHST5
TERF2IP
LOC100506281
TMEM231
GABARAPL2
75
75.2
75.4
75.6
Position on chr16 (Mb)

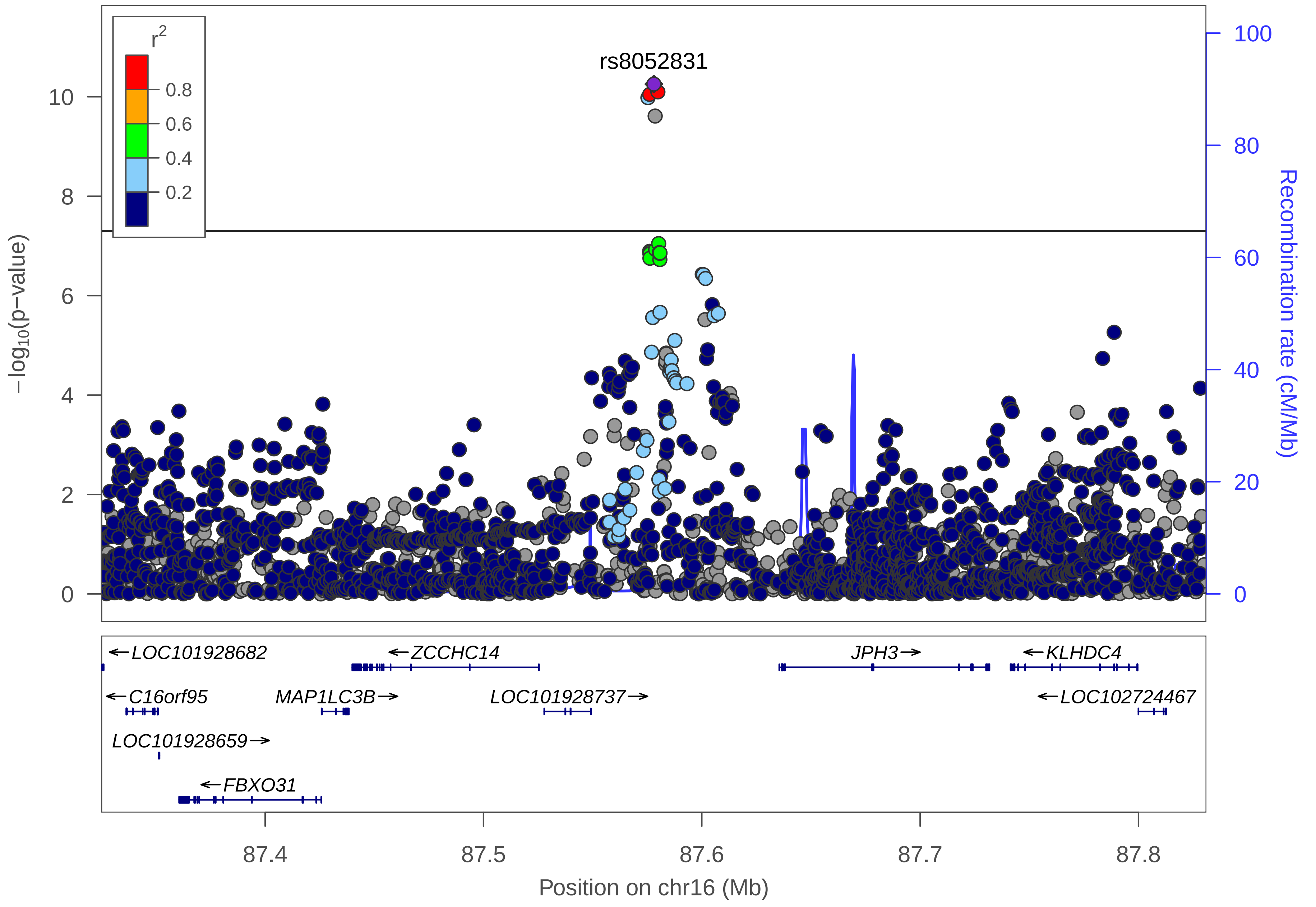

rs8052831
r2
0.8
0.6
0.4
0.2
−log10(p−value)
Recombination rate (cM/Mb)
←LOC101928682
←ZCCHC14
JPH3→
←KLHDC4
←C16orf95
MAP1LC3B→
LOC101928737→
←LOC102724467
LOC101928659→
←FBXO31
87.4
87.5
87.6
87.7
87.8
Position on chr16 (Mb)

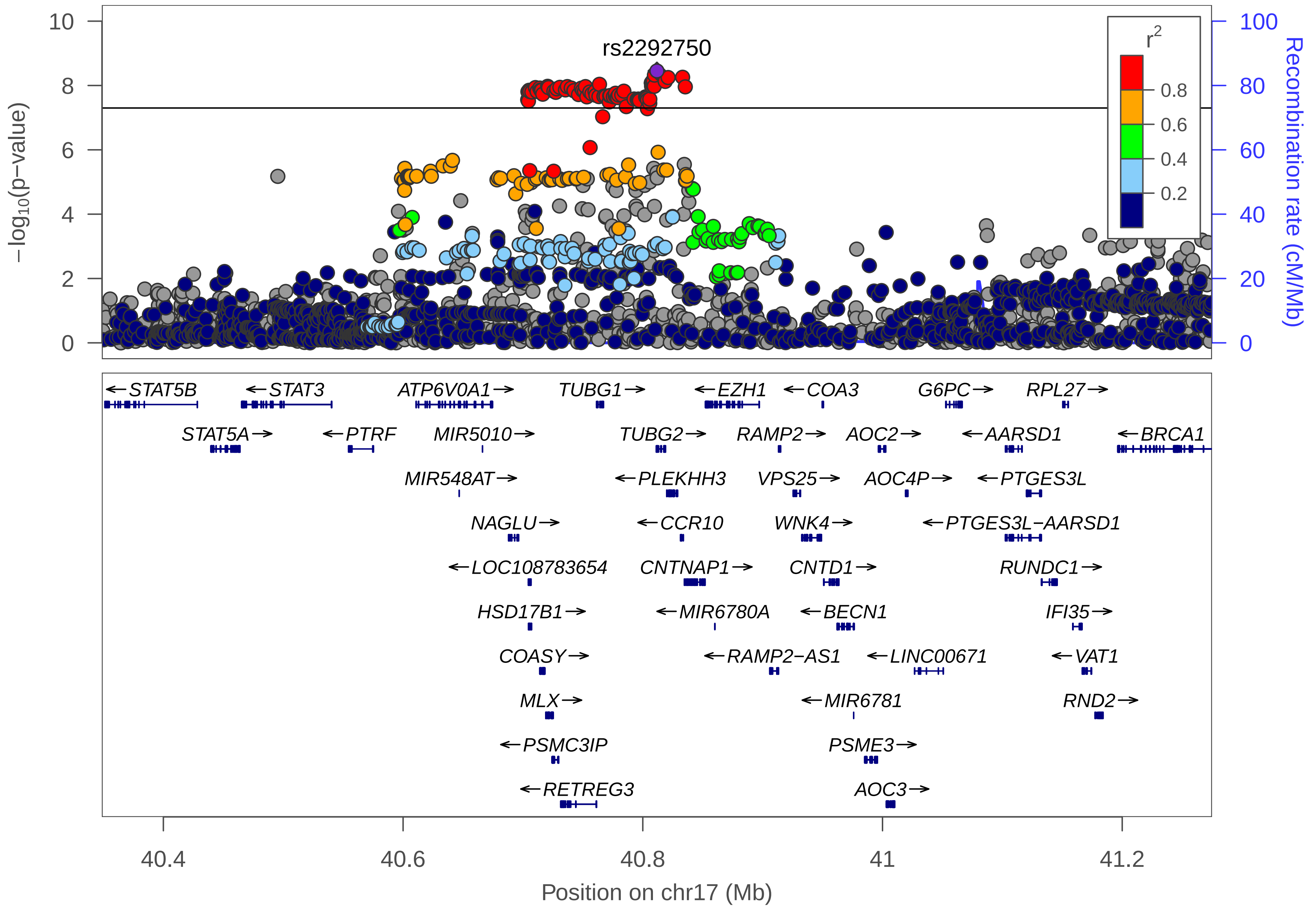
rs2292750
r2
0.8
0.6
0.4
0.2
10
8
6
4
2
0
100
80
60
40
20
0
−log10(p−value)
Recombination rate (cM/Mb)
←STAT5B
←STAT3
ATP6V0A1→
TUBG1→
←EZH1
←COA3
G6PC→
RPL27→
STAT5A→
←PTRF
MIR5010→
TUBG2→
RAMP2→
AOC2→
←AARSD1
←BRCA1
MIR548AT→
←PLEKHH3
VPS25→
AOC4P→
←PTGES3L
NAGLU→
←CCR10
WNK4→
←PTGES3L−AARSD1
←LOC108783654
CNTNAP1→
CNTD1→
RUNDC1→
HSD17B1→
←MIR6780A
←BECN1
IFI35→
COASY→
←RAMP2−AS1
←LINC00671
←VAT1
MLX→
←MIR6781
RND2→
←PSMC3IP
PSME3→
←RETREG3
AOC3→
40.4
40.6
40.8
41
41.2
Position on chr17 (Mb)

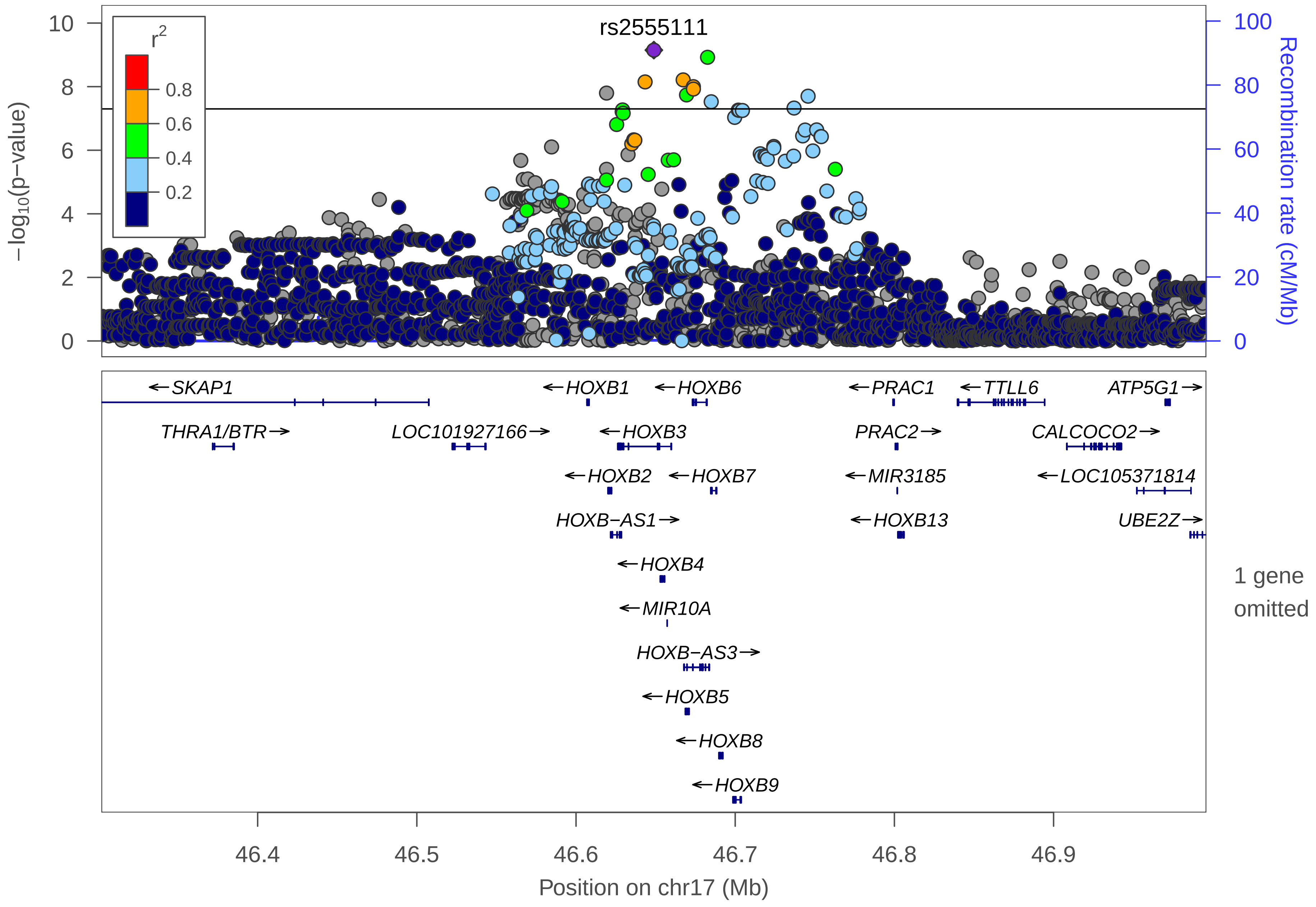
rs2555111
r2
0.8
0.6
0.4
0.2
10
8
6
4
2
0
−log10(p−value)
100
80
60
40
20
0
Recombination rate (cM/Mb)
SKAP1
THRA1/BTR
LOC101927166
HOXB1
HOXB6
HOXB3
HOXB2
HOXB7
HOXB−AS1
HOXB4
MIR10A
HOXB−AS3
HOXB5
HOXB8
HOXB9
PRAC1
PRAC2
MIR3185
HOXB13
TTLL6
CALCOCO2
LOC105371814
ATP5G1
UBE2Z
1 gene omitted
46.4
46.5
46.6
46.7
46.8
46.9
Position on chr17 (Mb)

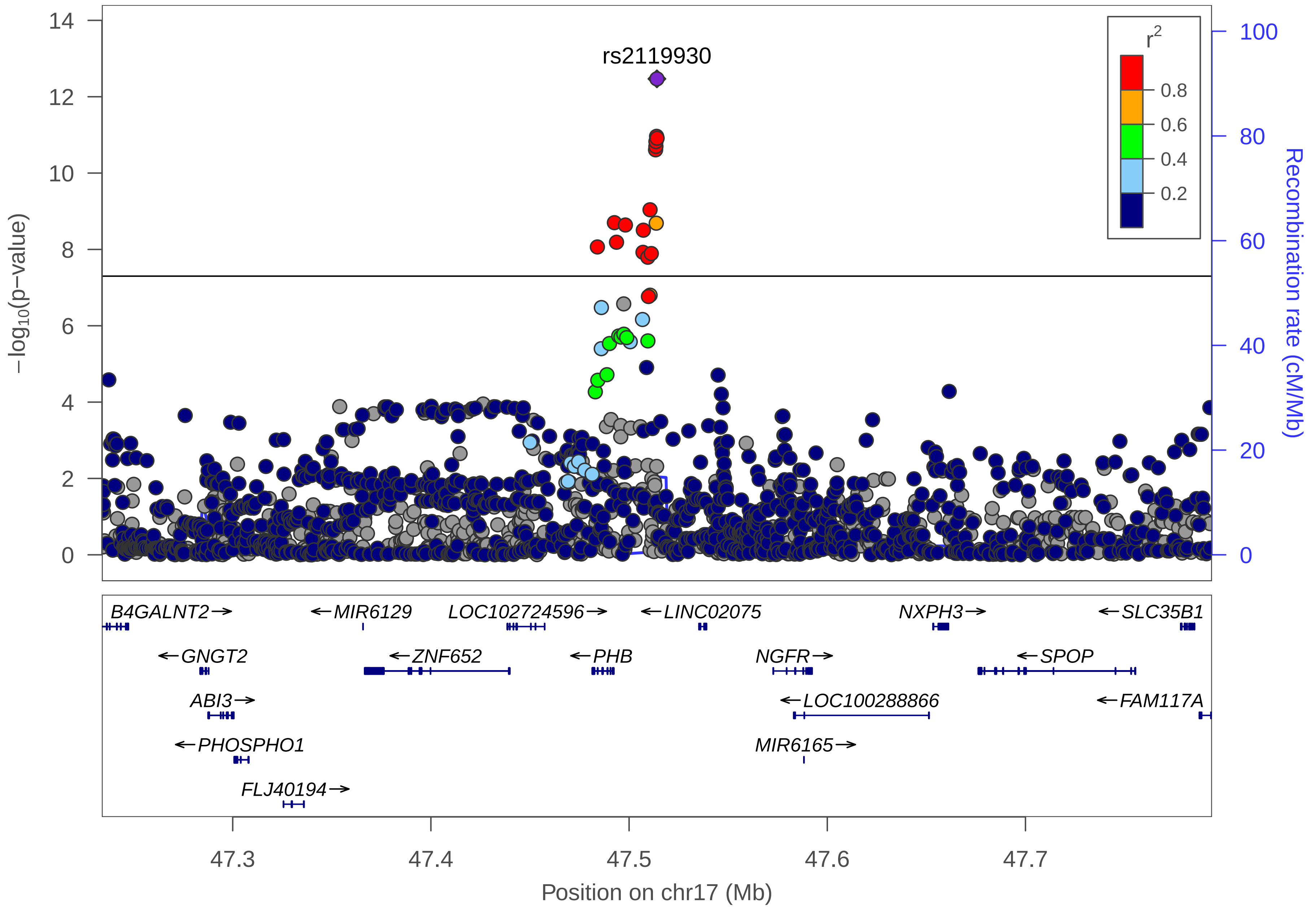

rs2119930
r²
0.8
0.6
0.4
0.2
−log10(p−value)
Recombination rate (cM/Mb)
0
2
4
6
8
10
12
14
0
20
40
60
80
100
B4GALNT2→
←MIR6129
LOC102724596→
←LINC02075
NXPH3→
←SLC35B1
←GNGT2
←ZNF652
←PHB
NGFR→
←SPOP
ABI3→
←LOC100288866
←FAM117A
←PHOSPHO1
MIR6165→
FLJ40194→
47.3
47.4
47.5
47.6
47.7
Position on chr17 (Mb)

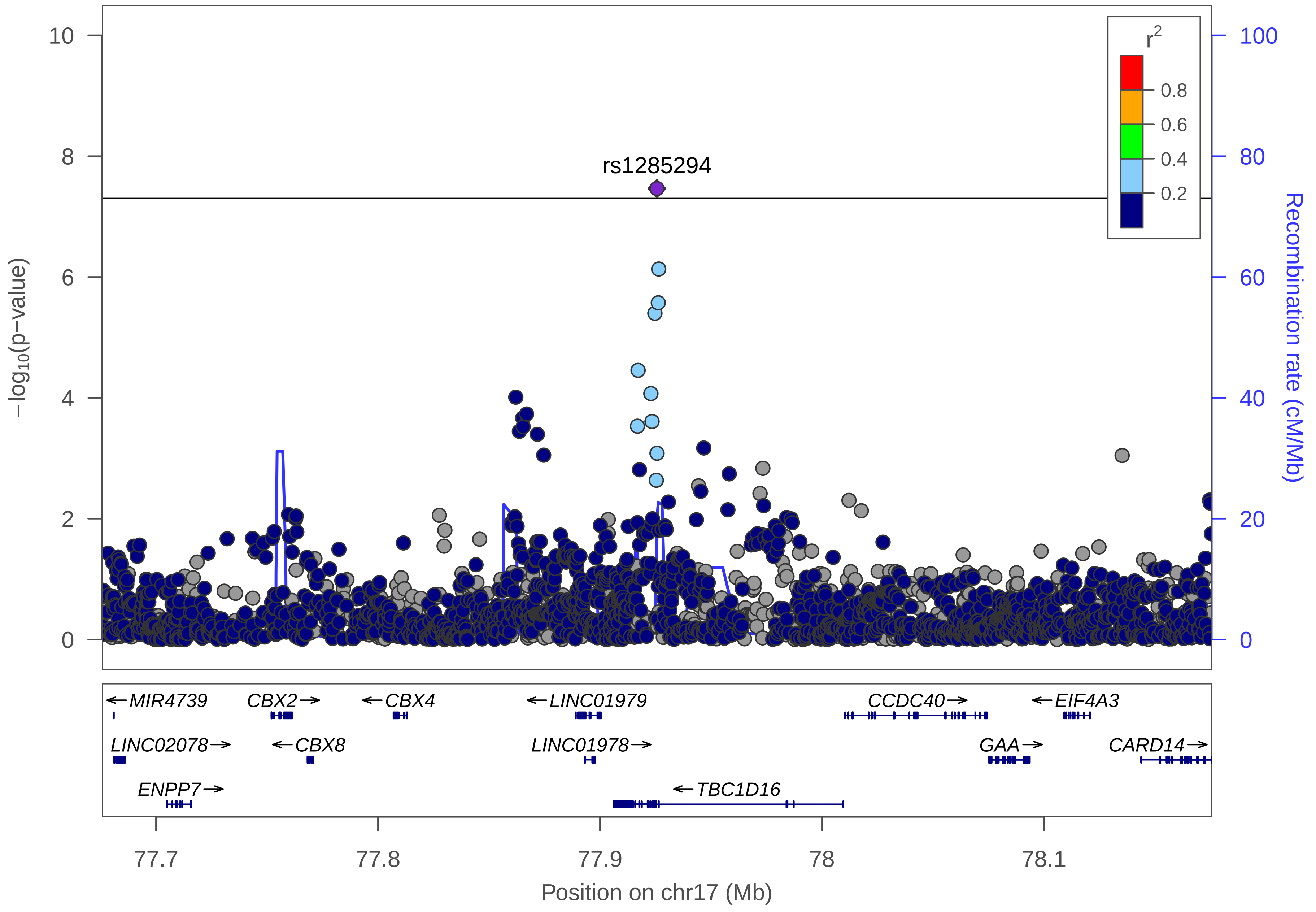

rs1285294
r2
0.8
0.6
0.4
0.2
−log10(p−value)
Recombination rate (cM/Mb)
10
8
6
4
2
0
100
80
60
40
20
0
MIR4739
CBX2
CBX4
LINC01979
CCDC40
EIF4A3
LINC02078
CBX8
LINC01978
GAA
CARD14
ENPP7
TBC1D16
77.7
77.8
77.9
78
78.1
Position on chr17 (Mb)

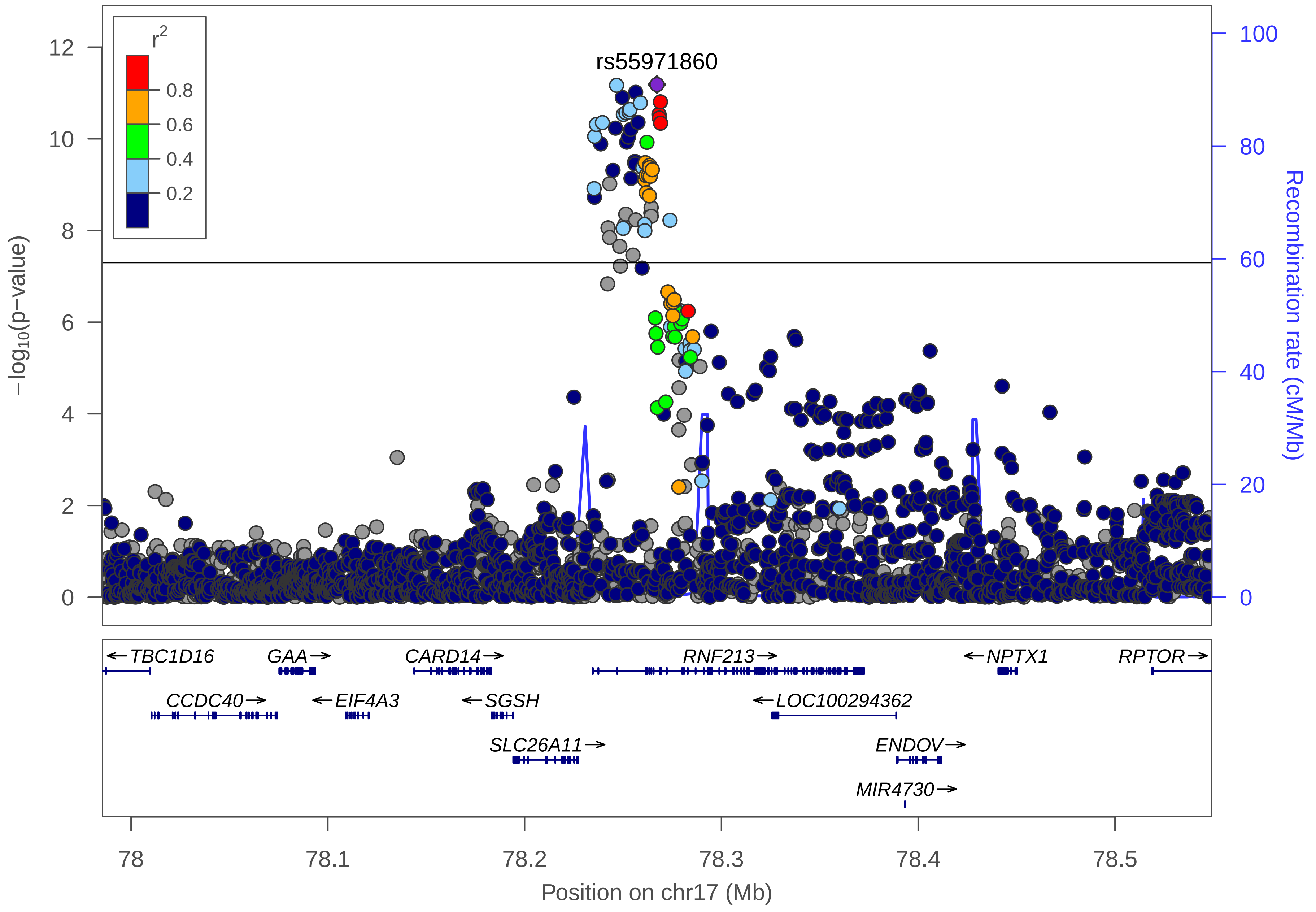

r2
0.8
0.6
0.4
0.2
rs55971860
12
10
8
6
4
2
0
−log10(p−value)
100
80
60
40
20
0
Recombination rate (cM/Mb)
←TBC1D16
GAA→
CARD14→
RNF213→
←NPTX1
RPTOR→
CCDC40→
←EIF4A3
←SGSH
←LOC100294362
SLC26A11→
ENDOV→
MIR4730→
78
78.1
78.2
78.3
78.4
78.5
Position on chr17 (Mb)

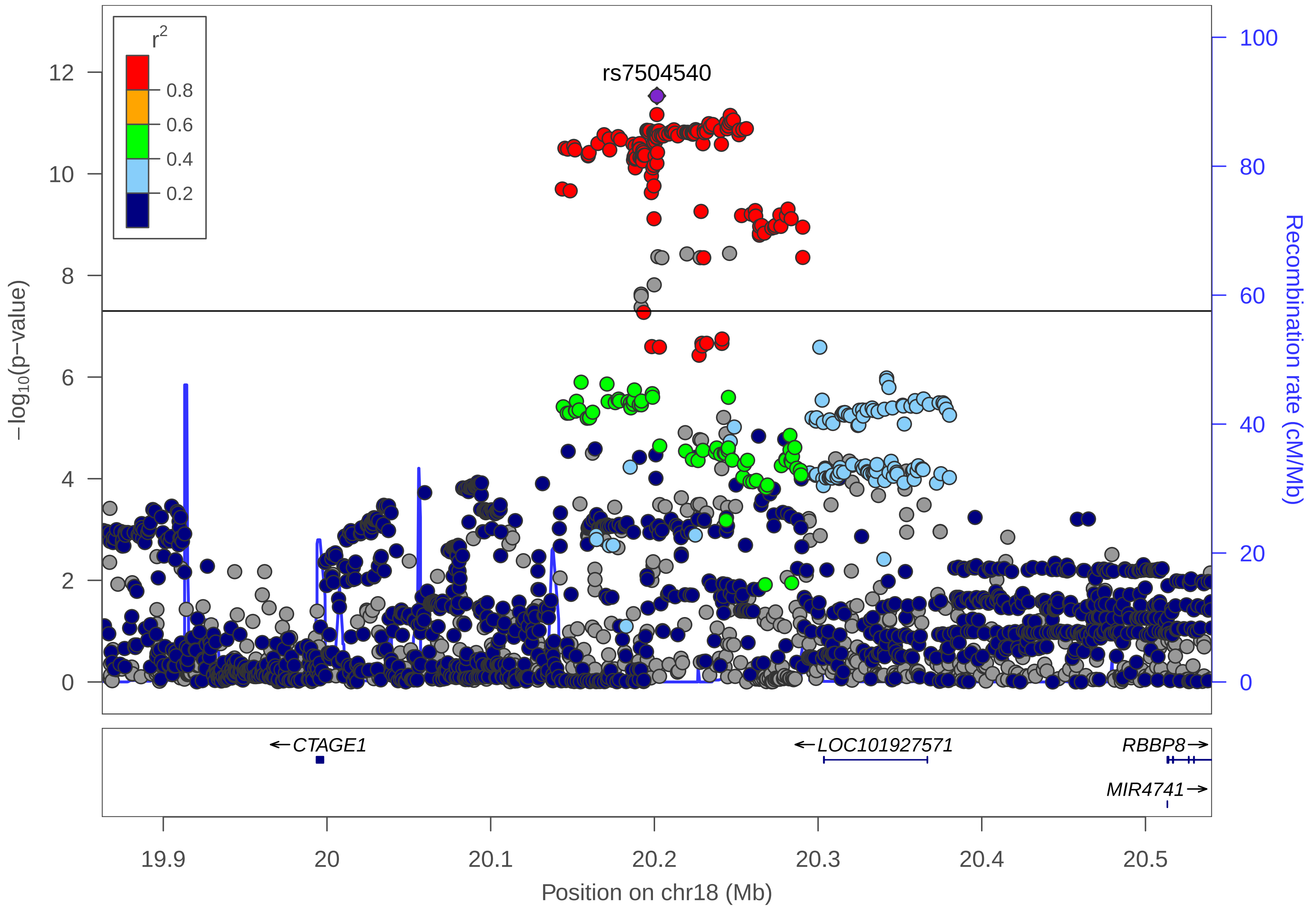

r2
0.8
0.6
0.4
0.2
rs7504540
12
10
8
6
4
2
0
−log10(p−value)
100
80
60
40
20
0
Recombination rate (cM/Mb)
←CTAGE1
←LOC101927571
RBBP8→
MIR4741→
19.9
20
20.1
20.2
20.3
20.4
20.5
Position on chr18 (Mb)

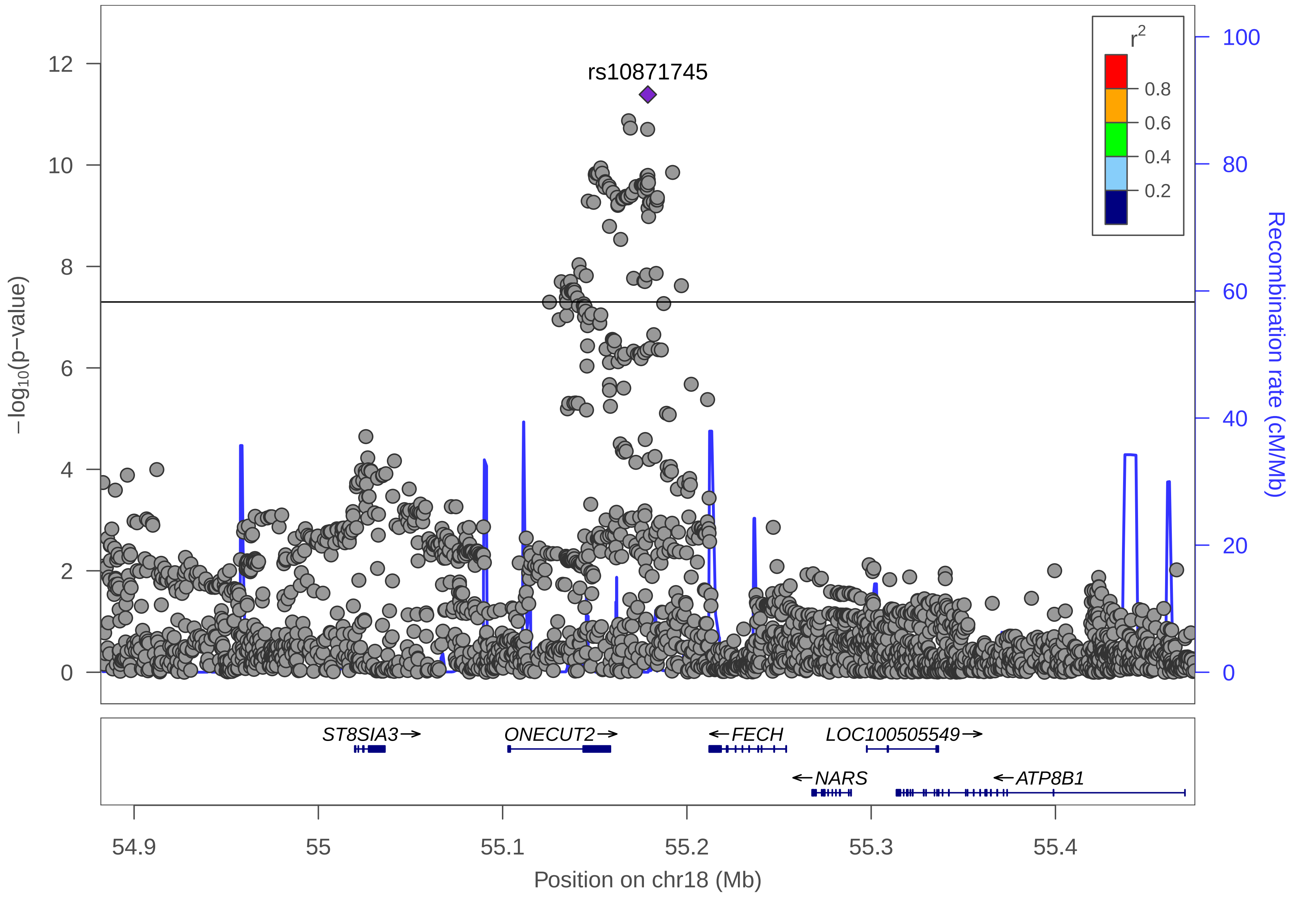

rs10871745
r2
0.8
0.6
0.4
0.2
−log10(p−value)
Recombination rate (cM/Mb)
0
2
4
6
8
10
12
0
20
40
60
80
100
ST8SIA3→
ONCUT2→
←FECH
LOC100505549→
←NARS
←ATP8B1
54.9
55
55.1
55.2
55.3
55.4
Position on chr18 (Mb)

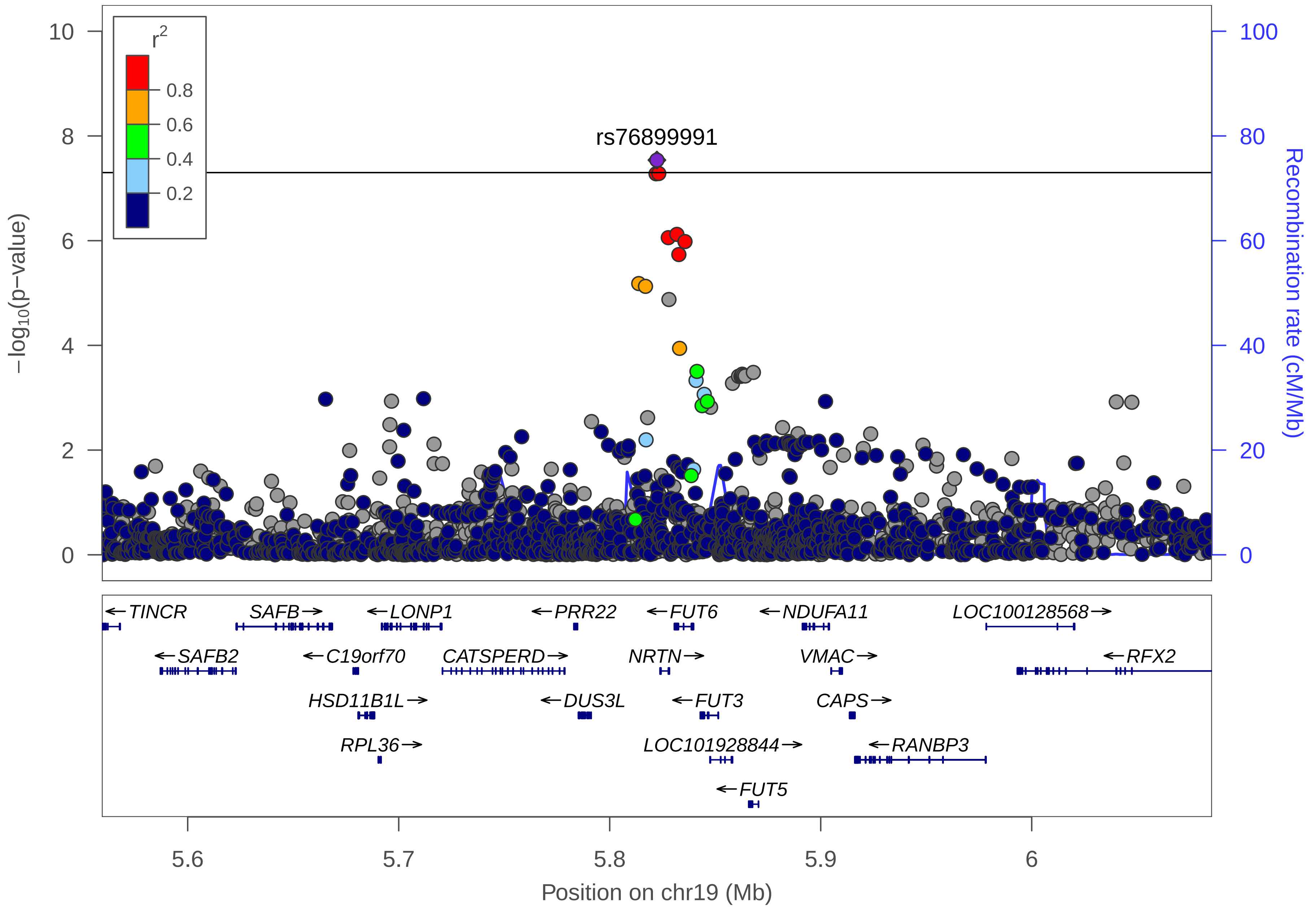

r2
0.8
0.6
0.4
0.2
rs76899991
10
8
6
4
2
0
−log10(p−value)
100
80
60
40
20
0
Recombination rate (cM/Mb)
← TINCR
SAFB →
← LONP1
← PRR22
← FUT6
← NDUFA11
LOC100128568 →
← SAFB2
← C19orf70
CATSPERD →
NRTN →
VMAC →
← RFX2
HSD11B1L →
← DUS3L
← FUT3
CAPS →
RPL36 →
LOC101928844 →
← RANBP3
← FUT5
5.6
5.7
5.8
5.9
6
Position on chr19 (Mb)

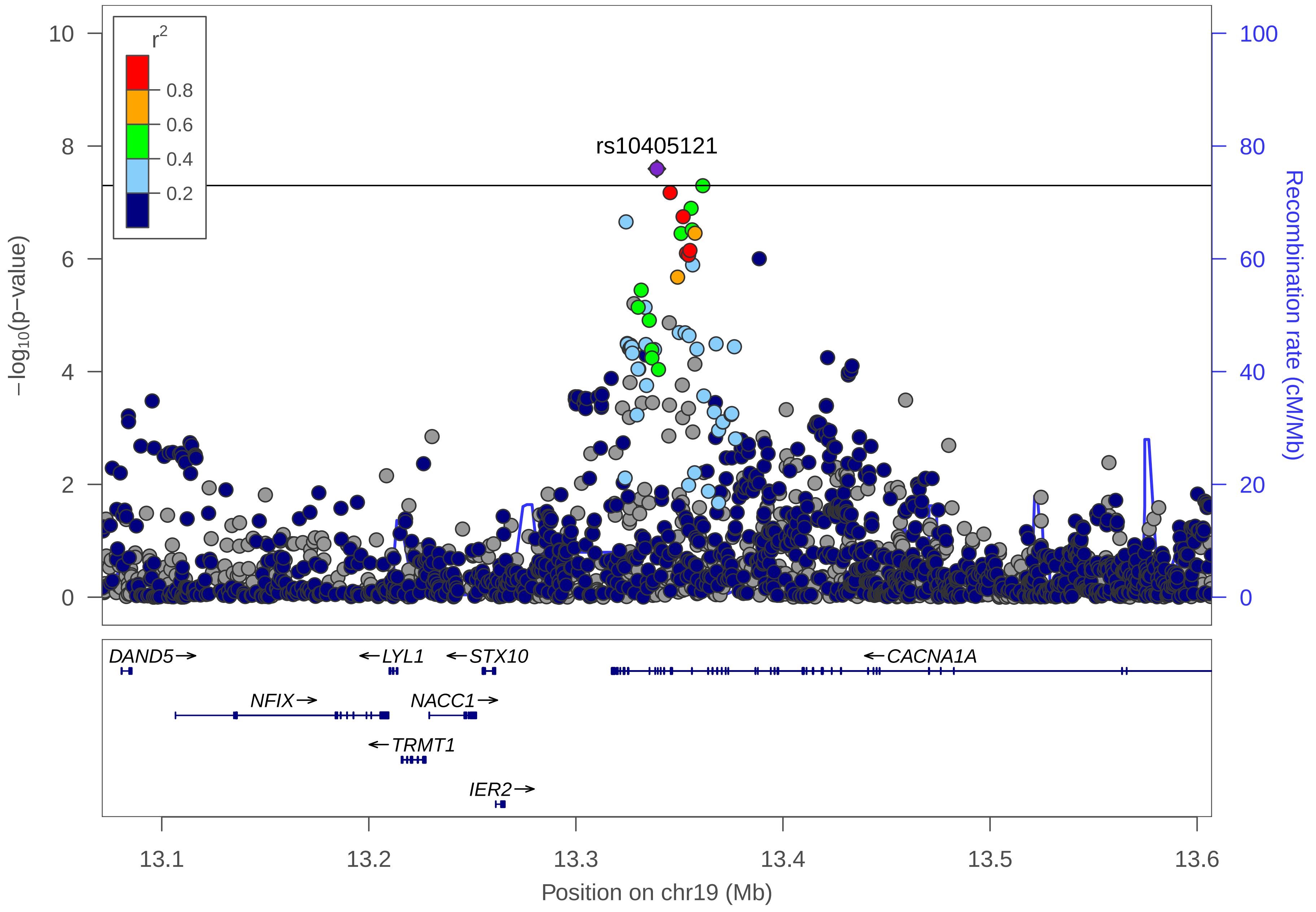

r2
0.8
0.6
0.4
0.2
rs10405121
−log10(p−value)
Recombination rate (cM/Mb)
10
8
6
4
2
0
100
80
60
40
20
0
DAND5
LYL1
STX10
CACNA1A
NFIX
NACC1
TRMT1
IER2
13.1
13.2
13.3
13.4
13.5
13.6
Position on chr19 (Mb)

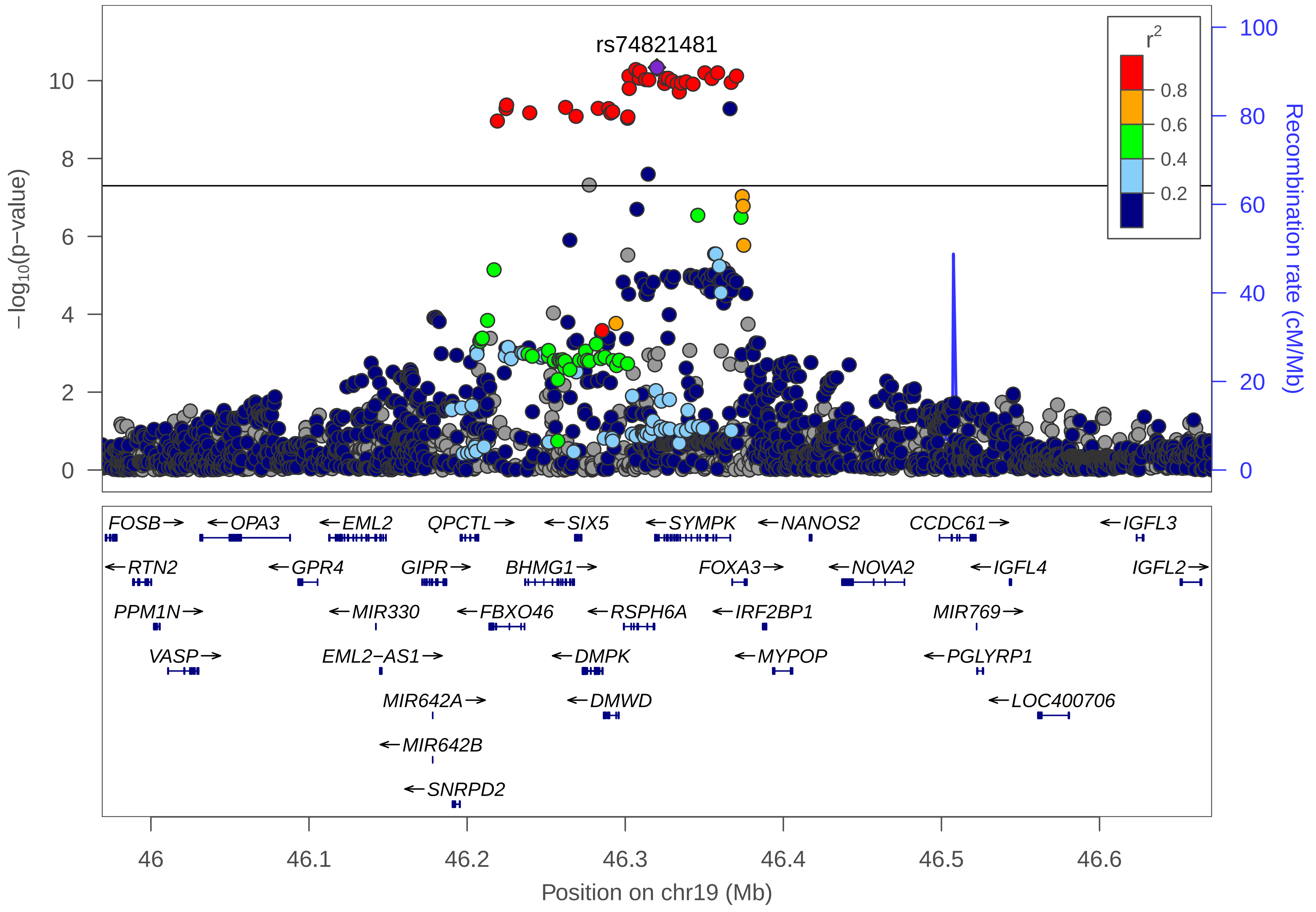

rs74821481
r2
0.8
0.6
0.4
0.2
−log10(p−value)
Recombination rate (cM/Mb)
0
2
4
6
8
10
0
20
40
60
80
100
FOSB→
←OPA3
←EML2
QPCTL→
←SIX5
←SYMPK
←NANOS2
CCDC61→
←IGFL3
←RTN2
←GPR4
GIPR→
BHMG1→
FOXA3→
←NOVA2
←IGFL4
IGFL2→
PPM1N→
←MIR330
←FBXO46
←RSPH6A
←IRF2BP1
MIR769→
VASP→
EML2−AS1→
←DMPK
←MYPOP
←PGLYRP1
MIR642A→
←DMWD
←LOC400706
←MIR642B
←SNRPD2
46
46.1
46.2
46.3
46.4
46.5
46.6
Position on chr19 (Mb)

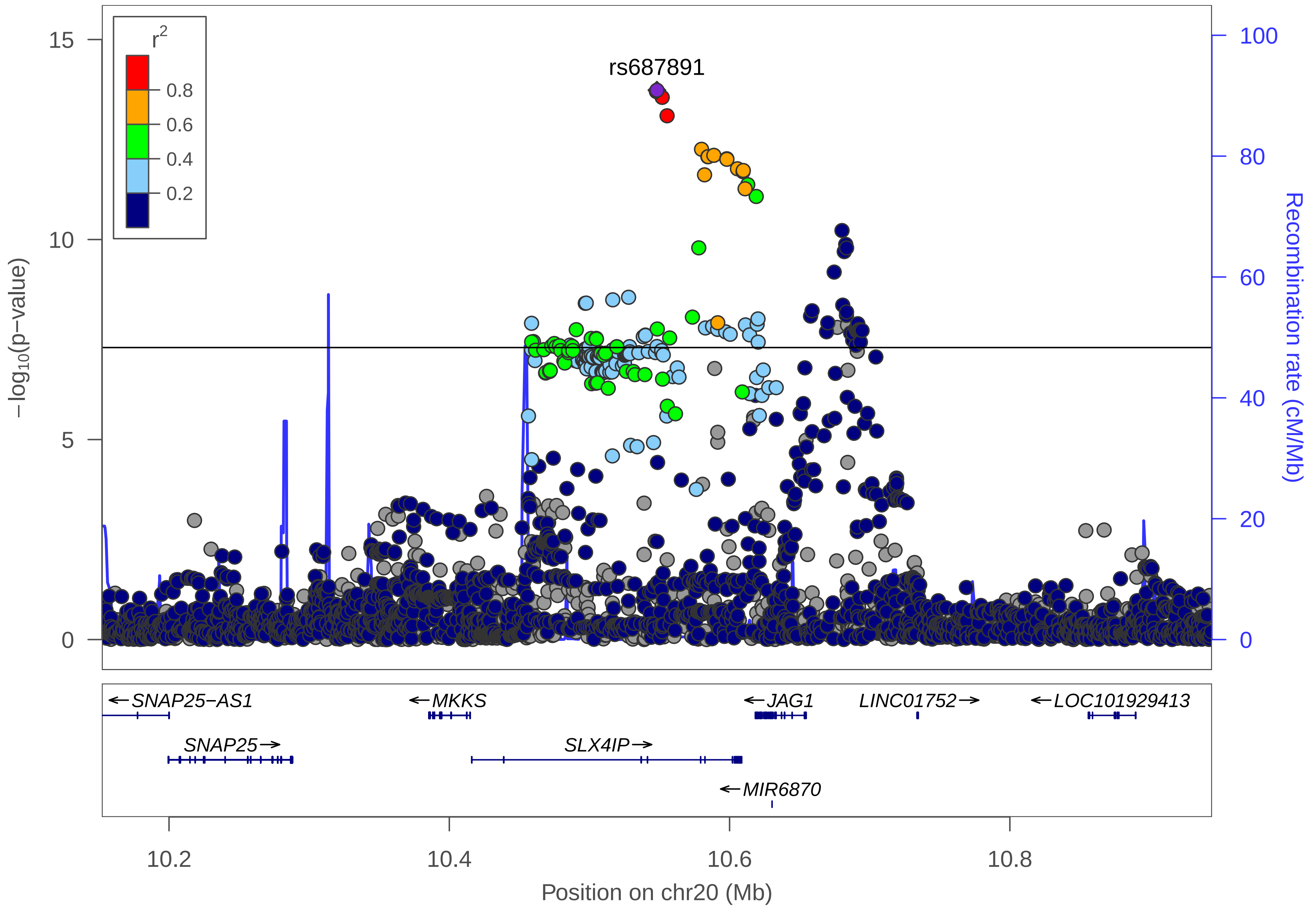

r2
0.8
0.6
0.4
0.2
rs687891
−log10(p−value)
Recombination rate (cM/Mb)
15
10
5
0
100
80
60
40
20
0
←SNAP25−AS1
←MKKS
←JAG1
LINC01752→
←LOC101929413
SNAP25→
SLX4IP→
←MIR6870
10.2
10.4
10.6
10.8
Position on chr20 (Mb)

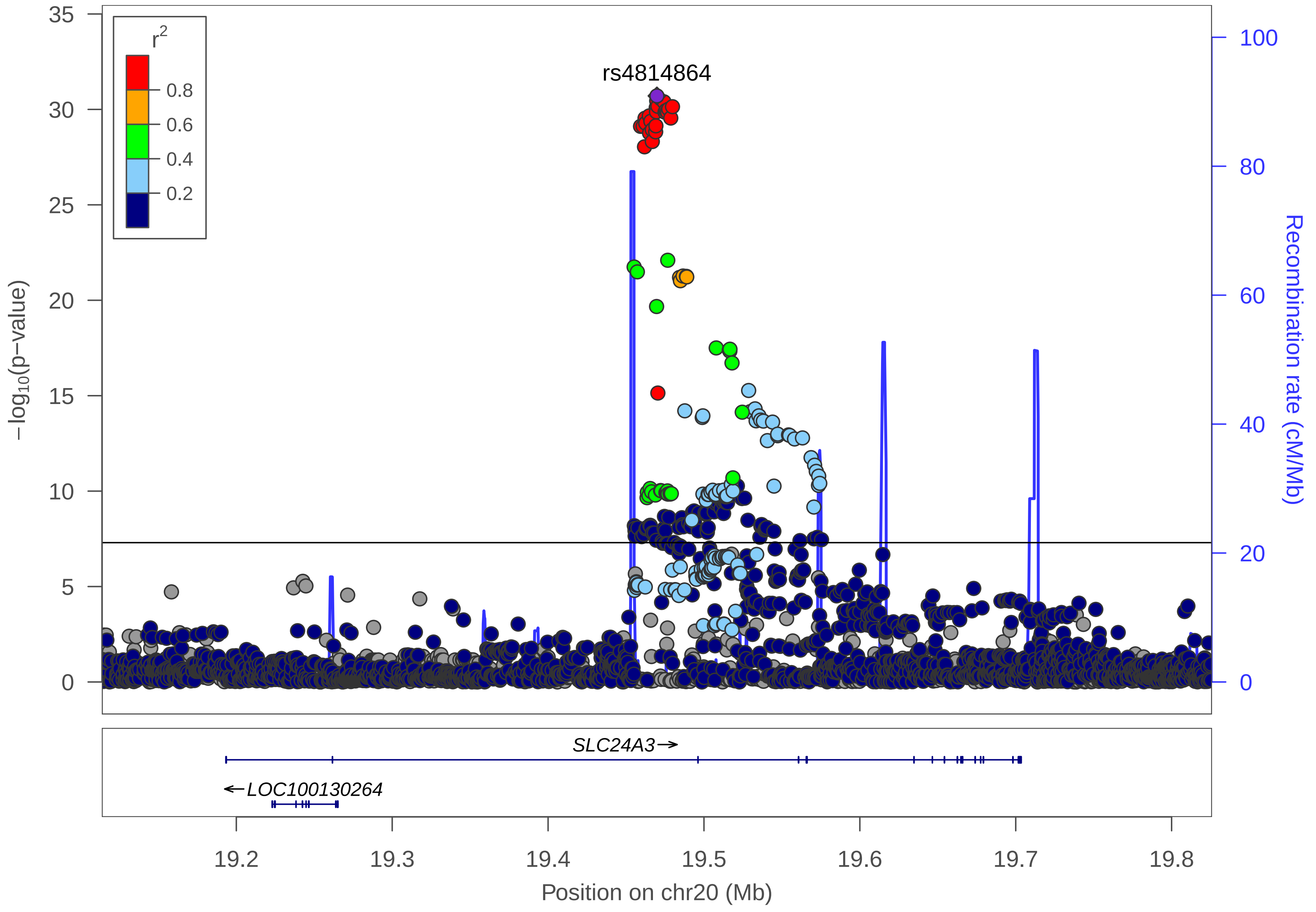

r2
0.8
0.6
0.4
0.2
rs4814864
−log10(p−value)
Recombination rate (cM/Mb)
35
30
25
20
15
10
5
0
100
80
60
40
20
0
SLC24A3→
←LOC100130264
19.2
19.3
19.4
19.5
19.6
19.7
19.8
Position on chr20 (Mb)

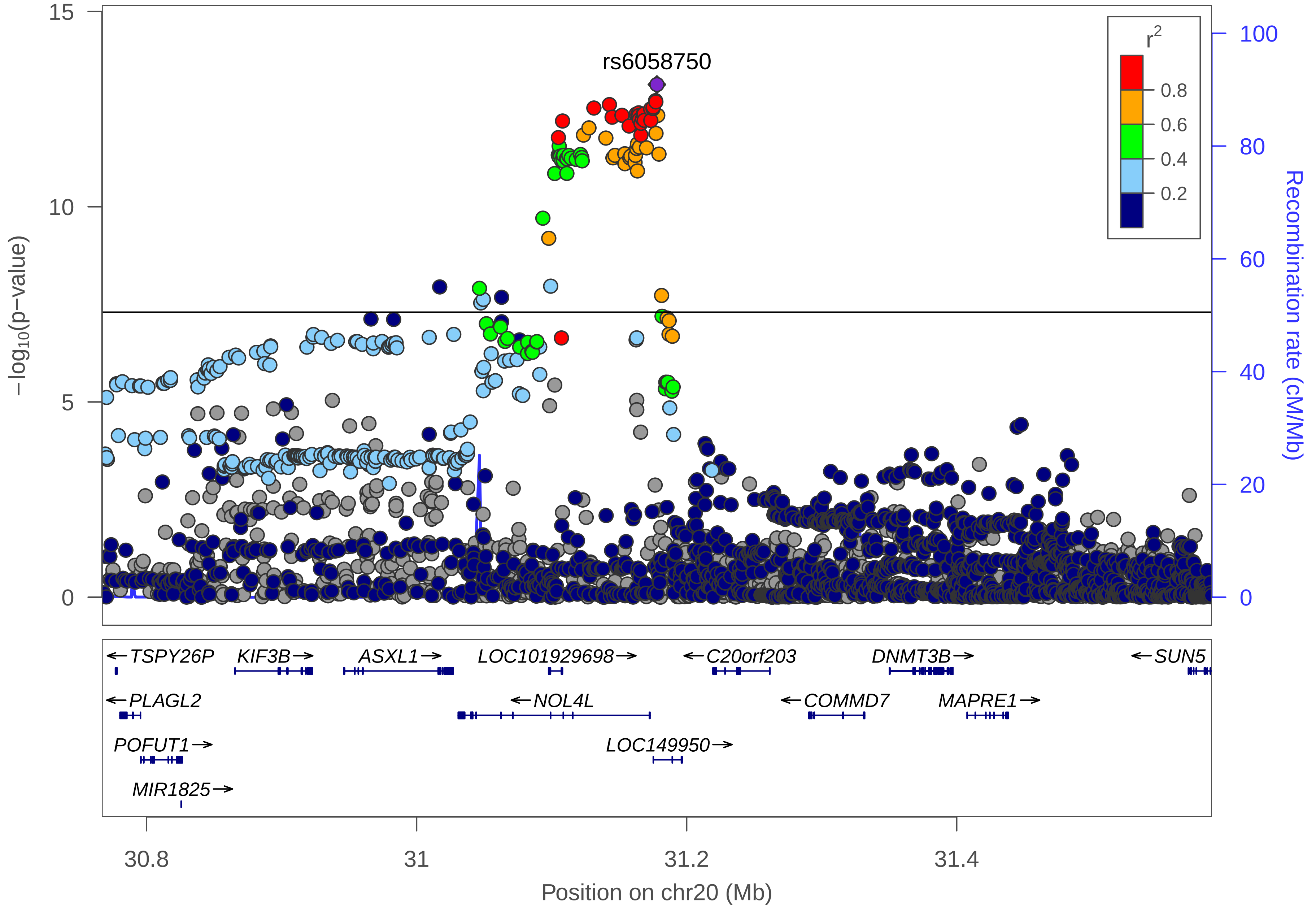

rs6058750
r2
0.8
0.6
0.4
0.2
−log10(p−value)
Recombination rate (cM/Mb)
15
10
5
0
100
80
60
40
20
0
←TSPY26P
KIF3B→
ASXL1→
LOC101929698→
←C20orf203
DNMT3B→
←SUN5
←PLAGL2
←NOL4L
←COMMD7
MAPRE1→
POFUT1→
LOC149950→
MIR1825→
30.8
31
31.2
31.4
Position on chr20 (Mb)

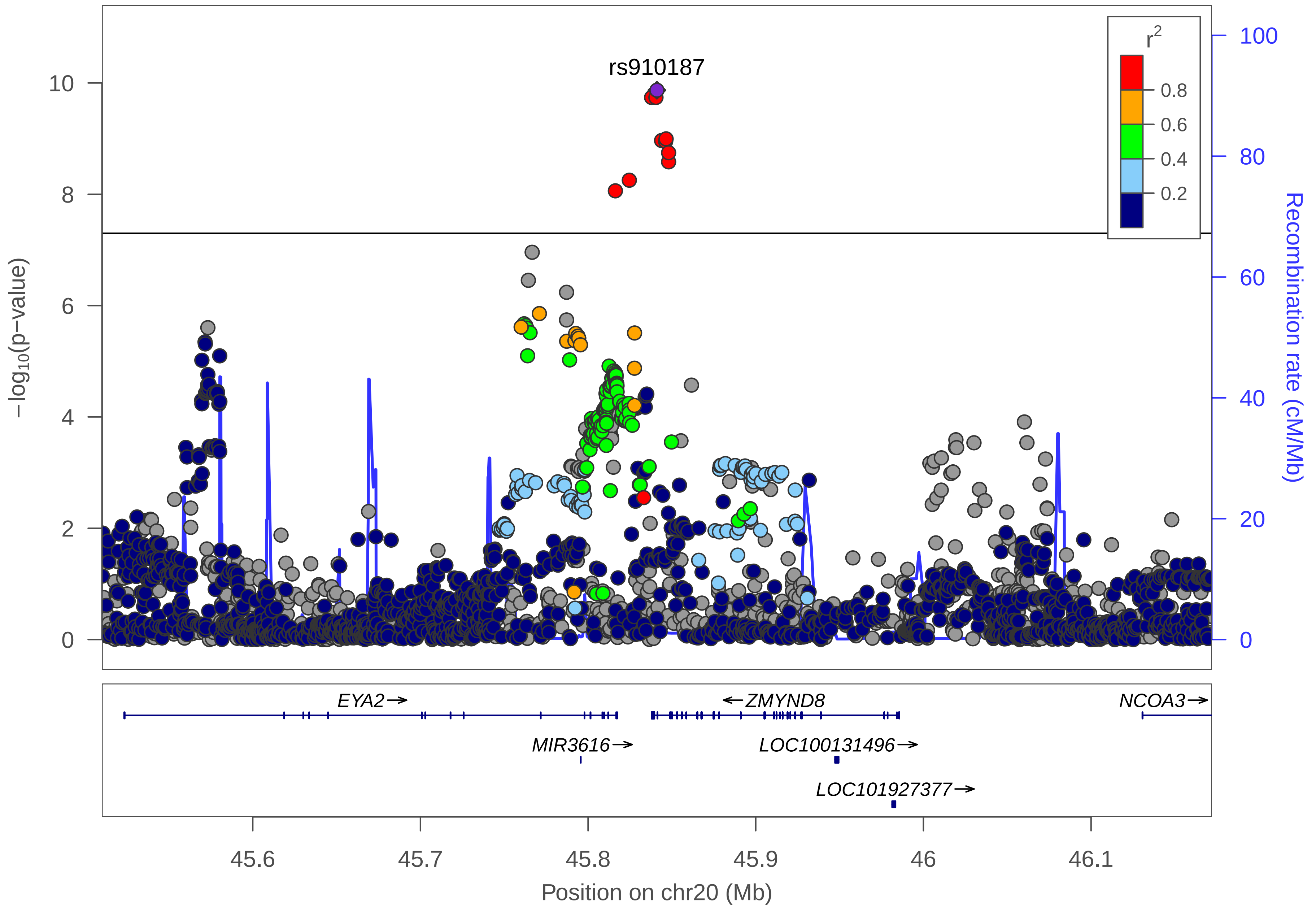
rs910187
r^2
0.8
0.6
0.4
0.2
−log10(p−value)
Recombination rate (cM/Mb)
10
8
6
4
2
0
100
80
60
40
20
0
EYA2→
←ZMYND8
NCOA3→
MIR3616→
LOC100131496→
LOC101927377→
45.6
45.7
45.8
45.9
46
46.1
Position on chr20 (Mb)

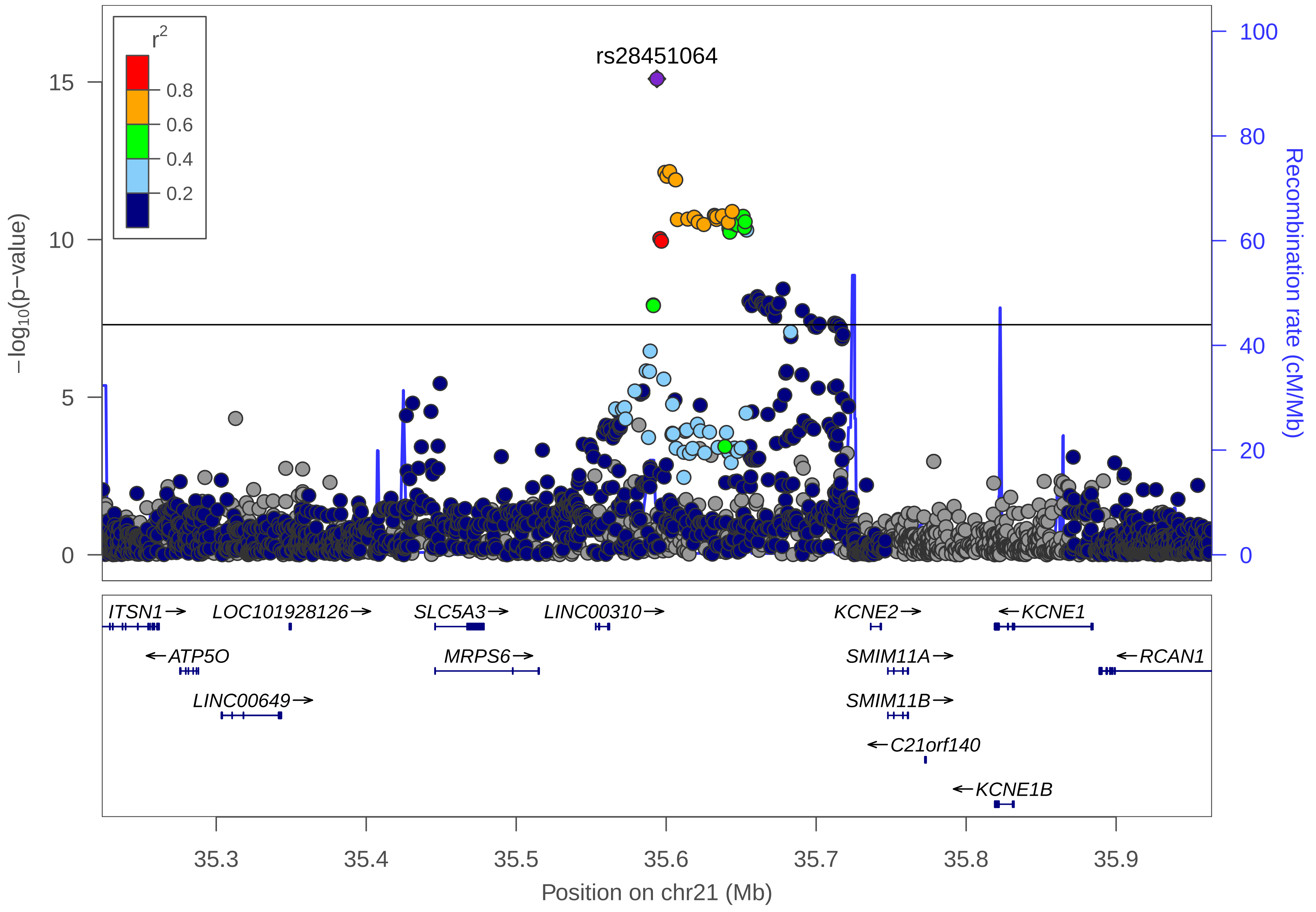

r2
0.8
0.6
0.4
0.2
rs28451064
15
10
5
0
−log10(p−value)
100
80
60
40
20
0
Recombination rate (cM/Mb)
ITSN1→
LOC101928126→
SLC5A3→
LINC00310→
KCNE2→
←KCNE1
←ATP5O
MRPS6→
SMIM11A→
←RCAN1
LINC00649→
SMIM11B→
←C21orf140
←KCNE1B
35.3
35.4
35.5
35.6
35.7
35.8
35.9
Position on chr21 (Mb)

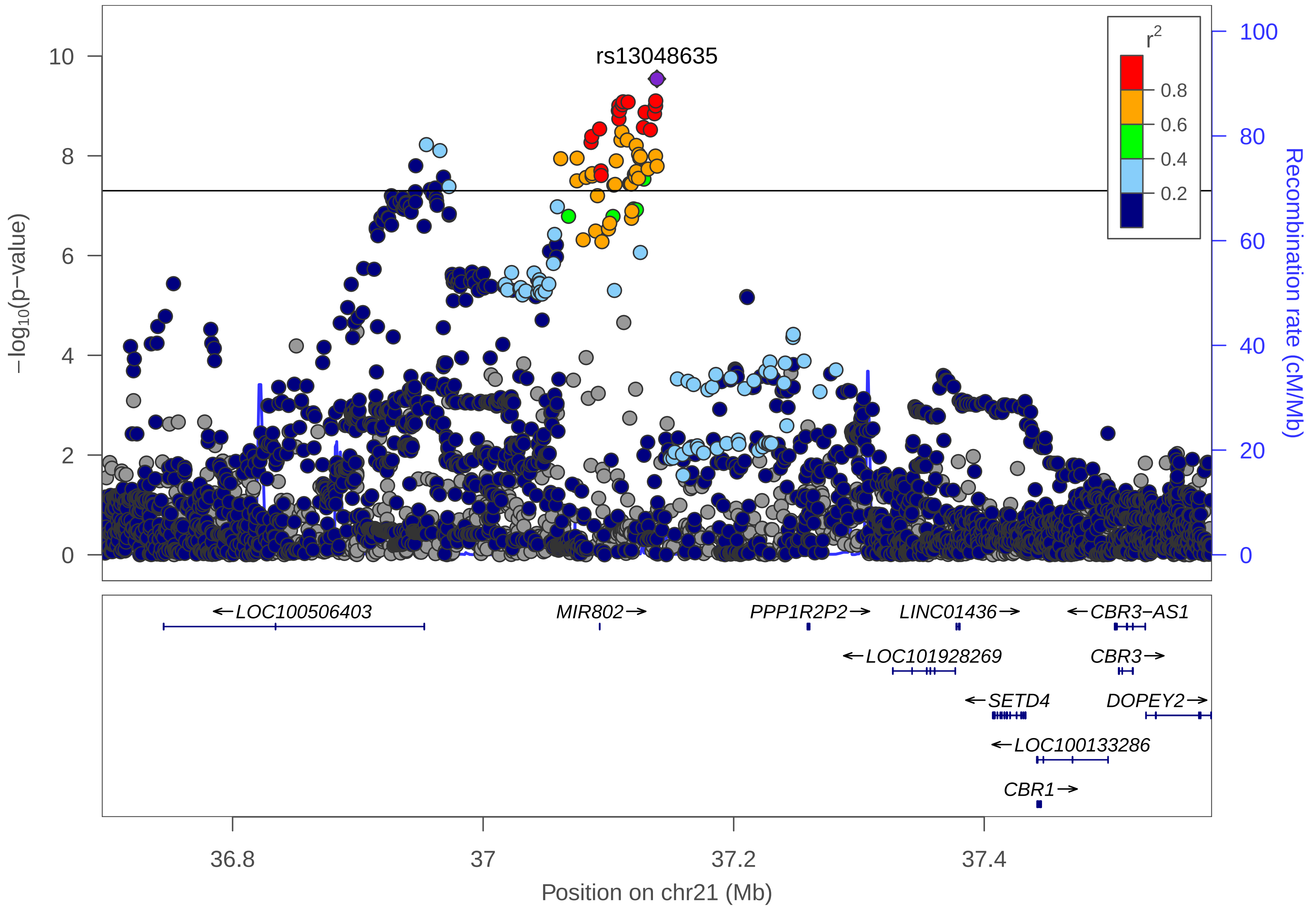

rs13048635
r2
0.8
0.6
0.4
0.2
−log10(p−value)
Recombination rate (cM/Mb)
LOC100506403
MIR802
PPP1R2P2
LINC01436
CBR3−AS1
LOC101928269
CBR3
SETD4
DOPEY2
LOC100133286
CBR1
36.8
37
37.2
37.4
Position on chr21 (Mb)

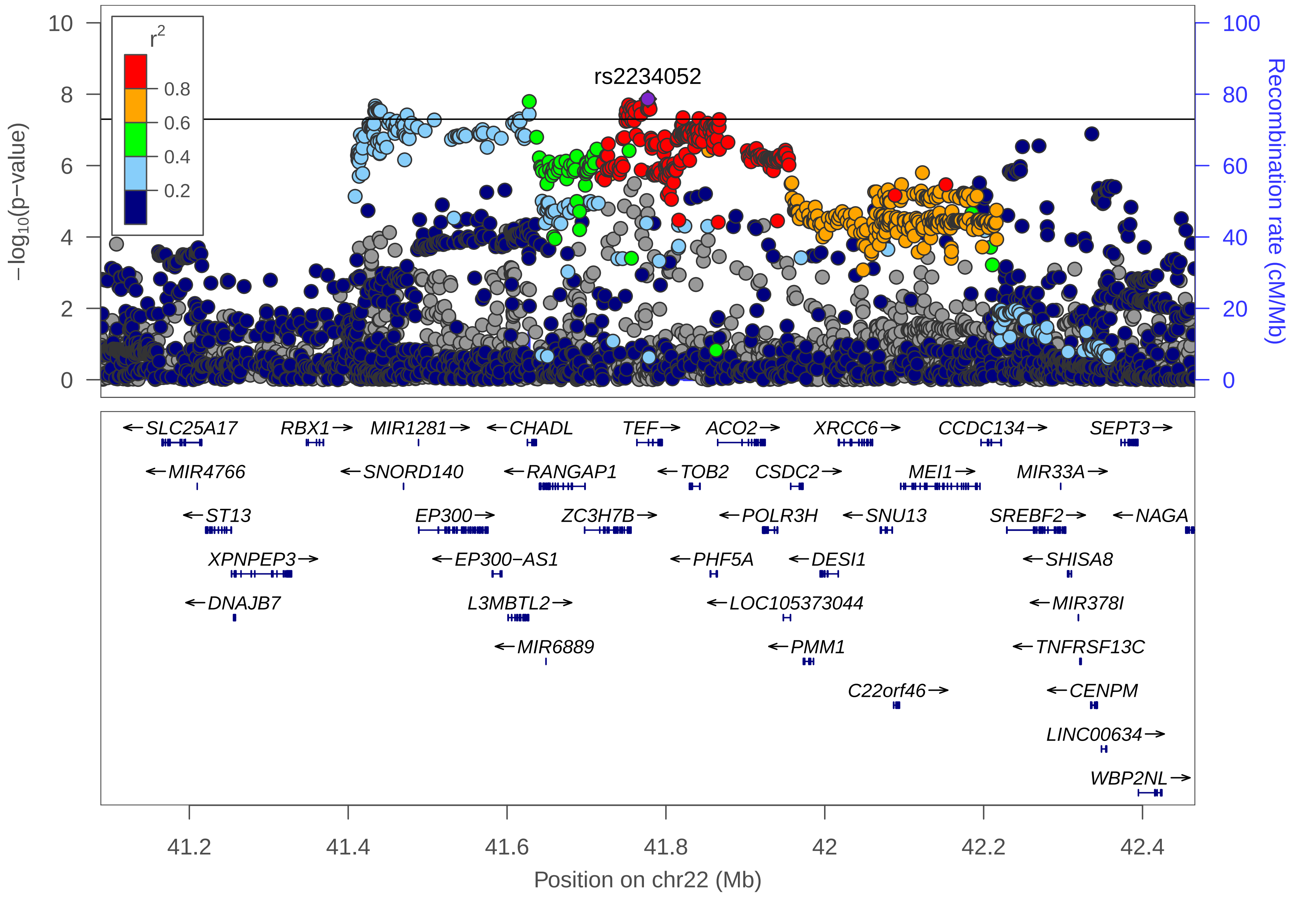

rs2234052
r2
0.8
0.6
0.4
0.2
−log10(p−value)
Recombination rate (cM/Mb)
0
2
4
6
8
10
0
20
40
60
80
100
SLC25A17
MIR4766
ST13
XPNPEP3
DNAJB7
RBX1
MIR1281
SNORD140
EP300
EP300−AS1
L3MBTL2
MIR6889
CHADL
RANGAP1
ZC3H7B
TEF
TOB2
PHF5A
ACO2
POLR3H
LOC105373044
CSDC2
DESI1
PMM1
XRCC6
SNU13
C22orf46
MEI1
CCDC134
SREBF2
SHISA8
MIR378I
TNFRSF13C
CENPM
LINC00634
WBP2NL
MIR33A
SEPT3
NAGA
41.2
41.4
41.6
41.8
42
42.2
42.4
Position on chr22 (Mb)

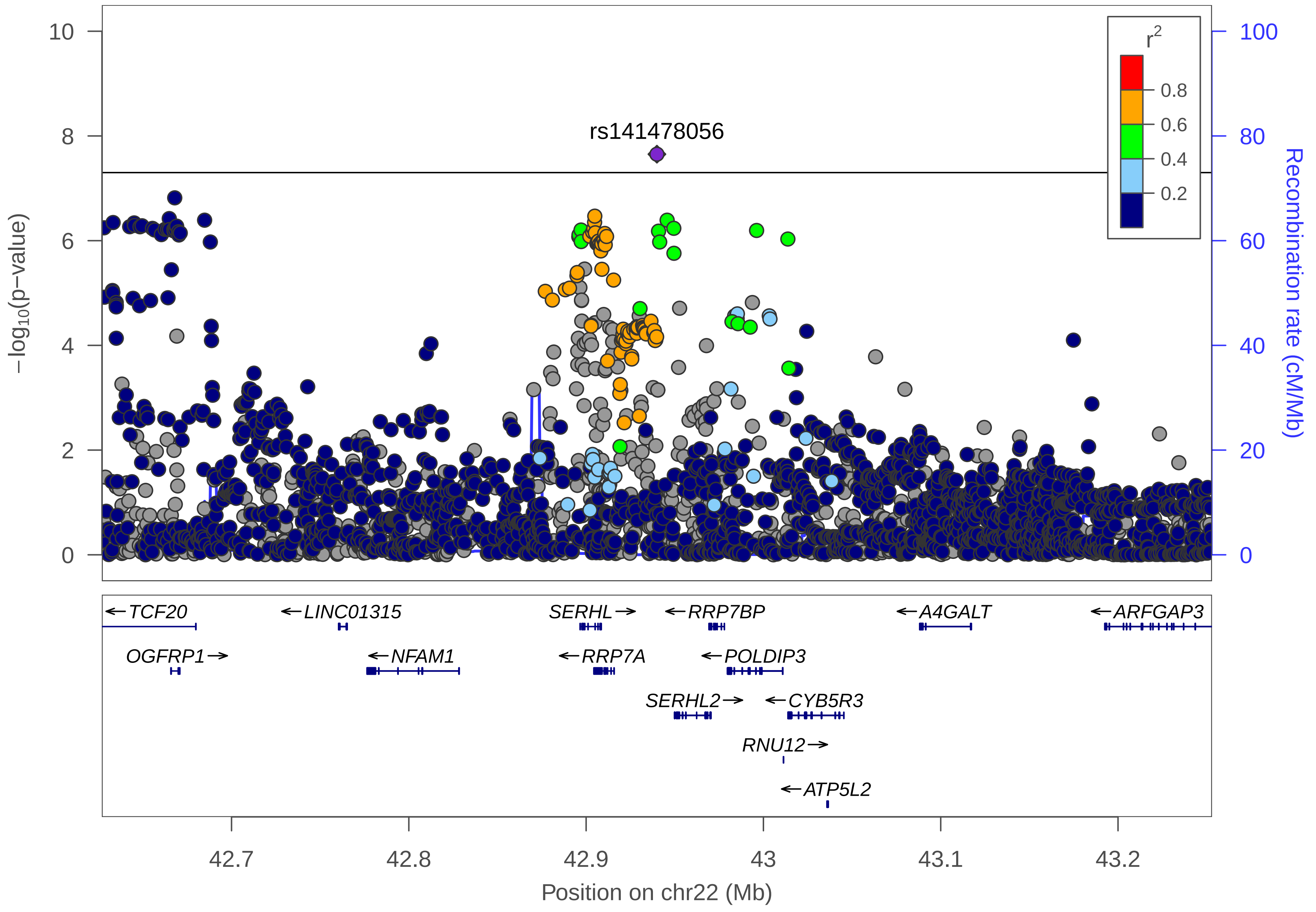

rs141478056
r2
0.8
0.6
0.4
0.2
10
8
6
4
2
0
100
80
60
40
20
0
−log10(p−value)
Recombination rate (cM/Mb)
TCF20
LINC01315
SERHL
RRP7BP
A4GALT
ARFGAP3
OGFRP1
NFAM1
RRP7A
POLDIP3
SERHL2
CYB5R3
RNU12
ATP5L2
42.7
42.8
42.9
43
43.1
43.2
Position on chr22 (Mb)

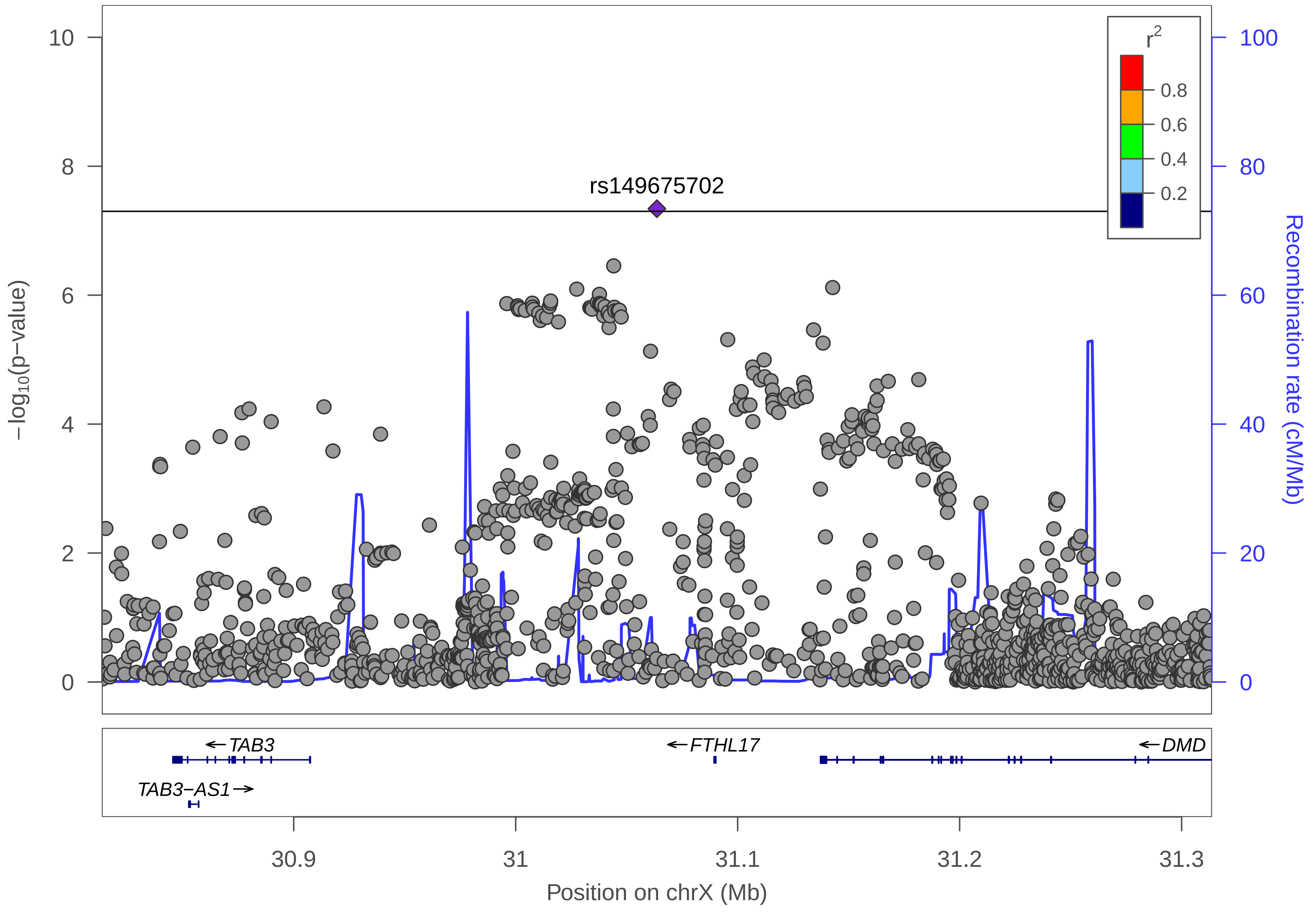

rs149675702
r2
0.8
0.6
0.4
0.2
−log10(p−value)
Recombination rate (cM/Mb)
10
8
6
4
2
0
100
80
60
40
20
0
TAB3
FTHL17
DMD
TAB3−AS1
30.9
31
31.1
31.2
31.3
Position on chrX (Mb)

Supplementary Figure 5.

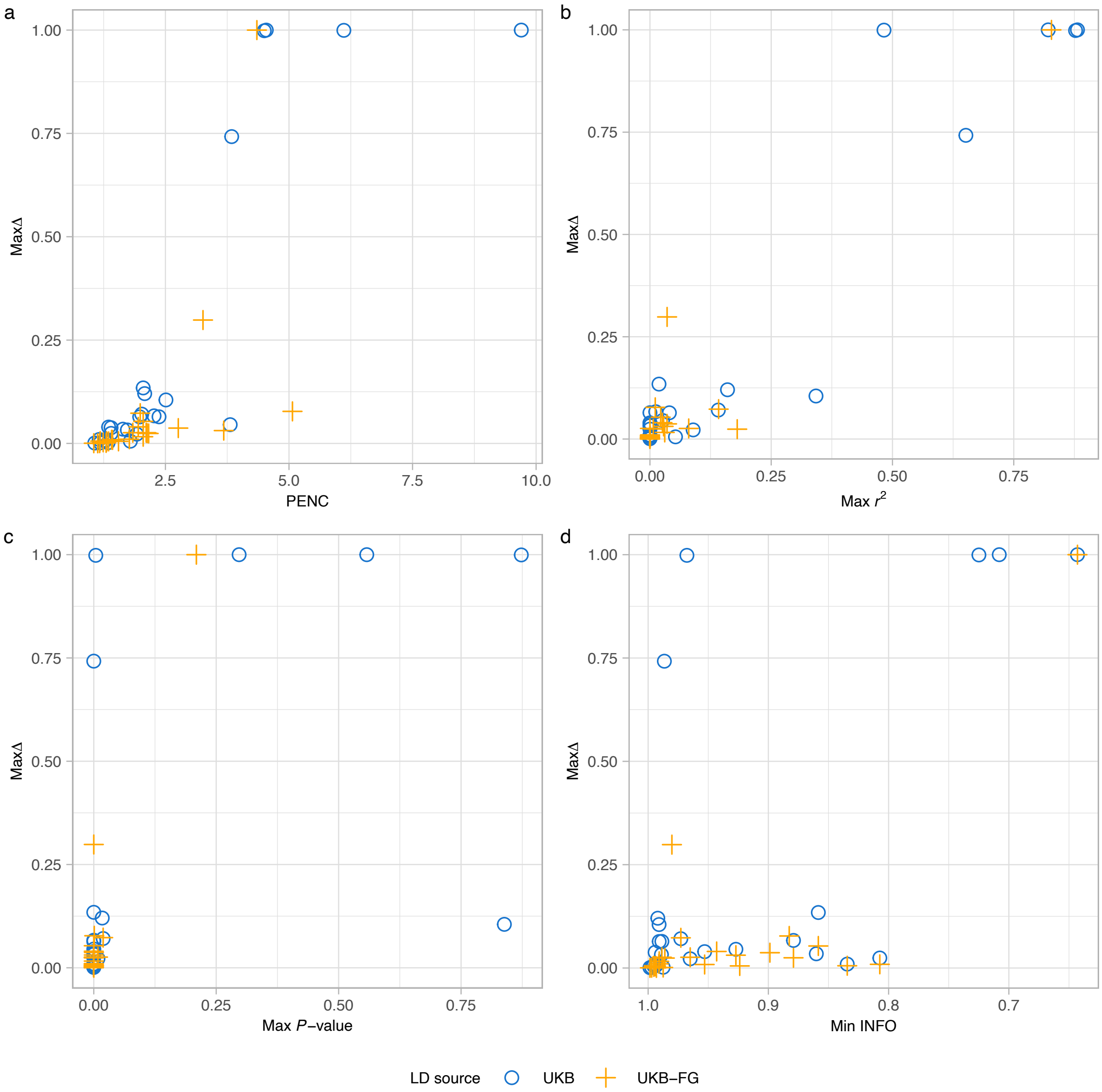

Supplementary Figure 5. Comparison of two reference LD panels (UK Biobank =UKB; N_{eff}= 42,135, and UK Biobank-FinnGen=UKB-FG; N_{eff}= 165,867) in fine-mapping of 26 migraine risk loci with in-sample LD available. Y-axis shows the maxΔ which is a maximum difference of a variant-specific posterior inclusion probabilities between the reference LD and in-sample LD from fine-mapping. X-axis shows a) posterior expectation of the number of causal variants (PENC) from FINEMAP, or, from the top variant(s) of the credible set(s), b) the maximum pairwise squared correlation, c) the maximum marginal P-value from the inverse-variance weighted fixed-effect meta-analysis, and d) the minimum INFO-value. The results using UKB LD reference panel are shown by blue circles and using combined UKB-FG reference panel by orange crosses.

Supplementary Figure 6.

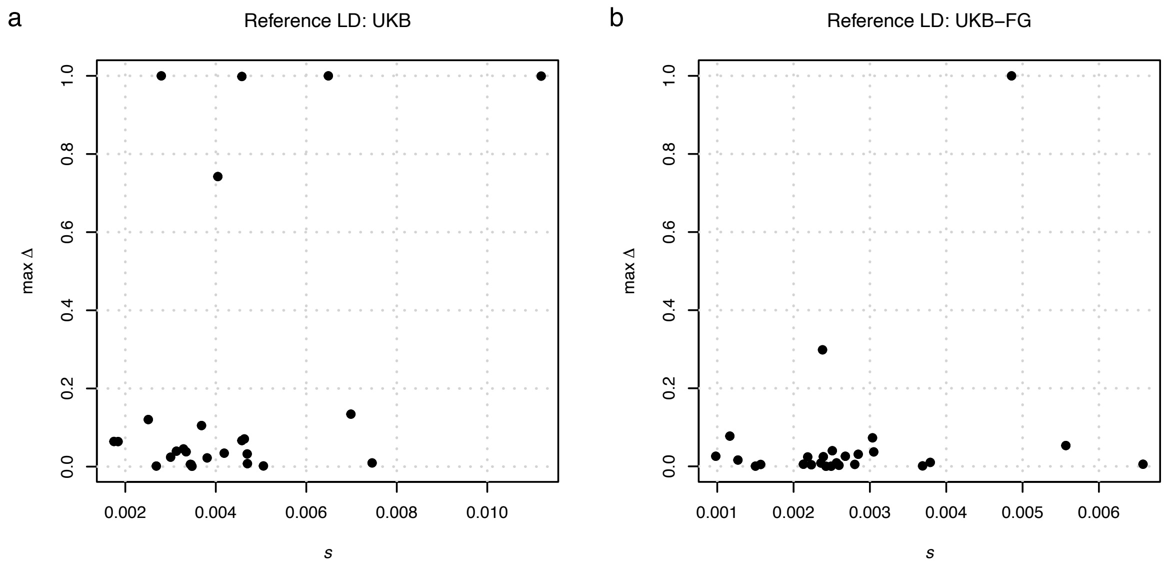

Supplementary Figure 6. **Quality of the reference LD fine-mapping as a function of regularization parameter '*s*'.** MaxΔ on y-axis is the maximum difference of the variant-wise posterior inclusion probabilities (PIPs) between the accurate fine-mapping using the in-sample LD and the approximate fine-mapping using the LD reference panel. Results with maxΔ > 0.1 are considered low quality. The regularization parameter '*s*' on x-axis is computed with the estimate_s_rss() function from the susieR package. Results are shown for the 26 regions where the in-sample LD was available. On left, results for the UK biobank LD reference (UKB, N_{eff}=42,135) and on right for UKB + FinnGen LD reference (UKB-FG, N_{eff}=165,867). If we choose a threshold value for '*s*' that filters out both low quality results (maxΔ > 0.1) with UKB-FG LD, we will also filter out 13 high quality regions (maxΔ < 0.1).
