# Supplementary material for: Fine-mapping a genome-wide meta-analysis of 98,374 migraine cases identifies 181 sets of candidate causal variants
Source: Nat Commun. 2026 Jan 12;17:355. doi: 10.1038/s41467-025-64880-3 (PMC12796328; doi:10.1038/s41467-025-64880-3)
Supplement: Supplementary file 2 — Description of Additional Supplementary Files [file 41467_2025_64880_MOESM2_ESM.pdf]

Description of Additional Supplementary Files

File Name: Supplementary Data 1.

Description: Lead variants of the 122 LD-independent (r2<0.1) genome-wide significant (P<5e-8) migraine risk loci identified in an inverse-variance weighted fixed-effects meta-analysis with 98,374 migraine cases and 869,160 controls.

File Name: Supplementary Data 2.

Description: Replication results of the 122 LD-independent (r2<0.1) lead variants using an external meta-analysis data of HUNT and IHGC16 without Finns and 23andMe data including 34,807 cases and 193,475 controls.

File Name: Supplementary Data 3a.

Description: Fine-mapping results of the 102 fine-map regions by FINEMAP using either insample LD (26 regions) or UKB-FG reference LD (76 regions).

File Name: Supplementary Data 3b.

Description: Lead variants filtered out from the meta-analysis due to QC protocol, and top variants of the credible sets of the corresponding fine-map regions.

File Name: Supplementary Data 4.

Description: Variants that have PIP > 0.1 in the credible sets of the 102 fine-map regions, and their overlap of significant cis-eQTLs in 49 tissues of GTEx v8 data.

File Name: Supplementary Data 5.

Description: Credible set variants that are predicted to have high or moderate impact on gene transcript by VEP, and their overlap of significant cis-eQTLs in 49 tissues of GTEx v8. data.

File Name: Supplementary Data 6.

Description: Phenome-wide association analysis of the 181 credible set top variants using 2,399 phenotypes from FinnGen R10 and a P-value threshold of 1e-5.

File Name: Supplementary Data 7.

Description: Protein QTL associations of the variants that have PIP > 0.1 in the credible sets of the 102 fine-map regions in UKB-PPP data.

File Name: Supplementary Data 8.

Description: Targeted phenome-wide association scan with 159 functional variants among the credible sets from fine-mapping 102 migraine fine-map regions within neurological and cardiovascular endpoints of FinnGen R10 with a P-value threshold of 1e-4.

File Name: Supplementary Data 9.

Description: Targeted phenome-wide association analysis with 307 variants that have posterior inclusion probability (PIP) > 0.1 among the credible sets from fine-mapping 102 migraine finemap regions within neurological and cardiovascular endpoints of FinnGen R10 with a P-value threshold of 1e-4.

File Name: Supplementary Data 10.

Description: List of FinnGen members.

File Name: Supplementary Data 11.

Description: List of International Headache Genetics Consortium (IHGC) members.

File Name: Supplementary Data 12.

Description: List of HUNT All-in Headache members.
