# Supplementary material for: Fine-mapping a genome-wide meta-analysis of 98,374 migraine cases identifies 181 sets of candidate causal variants
Source: Nat Commun. 2026 Jan 12;17:355. doi: 10.1038/s41467-025-64880-3 (PMC12796328; doi:10.1038/s41467-025-64880-3)
Supplement: Supplementary file 4 — Reporting Summary [file 41467_2025_64880_MOESM4_ESM.pdf]

nature portfolio

Corresponding author(s): Heidi Hautakangas, Matti Pirinen

Last updated by author(s): Sep 15, 2025

Reporting Summary

Nature Portfolio wishes to improve the reproducibility of the work that we publish. This form provides structure for consistency and transparency in reporting. For further information on Nature Portfolio policies, see our Editorial Policies and the Editorial Policy Checklist.

Statistics

For all statistical analyses, confirm that the following items are present in the figure legend, table legend, main text, or Methods section.

n/a	Confirmed	
☐	☒	The exact sample size (*n*) for each experimental group/condition, given as a discrete number and unit of measurement
☒	☐	A statement on whether measurements were taken from distinct samples or whether the same sample was measured repeatedly
☐	☒	The statistical test(s) used AND whether they are one- or two-sided<br>*Only common tests should be described solely by name; describe more complex techniques in the Methods section.*
☐	☒	A description of all covariates tested
☐	☒	A description of any assumptions or corrections, such as tests of normality and adjustment for multiple comparisons
☐	☒	A full description of the statistical parameters including central tendency (e.g. means) or other basic estimates (e.g. regression coefficient) AND variation (e.g. standard deviation) or associated estimates of uncertainty (e.g. confidence intervals)
☐	☒	For null hypothesis testing, the test statistic (e.g. *F*, *t*, *r*) with confidence intervals, effect sizes, degrees of freedom and *P* value noted<br>*Give P values as exact values whenever suitable.*
☐	☒	For Bayesian analysis, information on the choice of priors and Markov chain Monte Carlo settings
☒	☐	For hierarchical and complex designs, identification of the appropriate level for tests and full reporting of outcomes
☐	☒	Estimates of effect sizes (e.g. Cohen's *d*, Pearson's *r*), indicating how they were calculated

Our web collection on statistics for biologists contains articles on many of the points above.

Software and code

Policy information about availability of computer code

Data collection	No software was used for data collection.
Data analysis	Following software versions were used to conduct analyses:<br>R-3.5.2<br>FINEMAP v1.4<br>LDStore v2.0<br>LD Score regression v1.0.0<br>GWAMA v2.1<br>LiftOver (downloaded January 2021)<br>VEP (https://grch37.ensembl.org/Homo_sapiens/Tools/VEP)

For manuscripts utilizing custom algorithms or software that are central to the research but not yet described in published literature, software must be made available to editors and reviewers. We strongly encourage code deposition in a community repository (e.g. GitHub). See the Nature Portfolio guidelines for submitting code & software for further information.

Data

Policy information about availability of data

All manuscripts must include a data availability statement. This statement should provide the following information, where applicable:

- Accession codes, unique identifiers, or web links for publicly available datasets
- A description of any restrictions on data availability
- For clinical datasets or third party data, please ensure that the statement adheres to our policy

The GWAS summary statistics for UK Biobank are publicly available in GWAS Catalog under accession code GCST90671940. The access to the UK biobank data can be applied through https://www.ukbiobank.ac.uk/
The GWAS summary statistics for FinnGen R8 are publicly available through https://www.finngen.fi/en/access_results. The Finnish biobank data can be accessed through the Fingenious® services (https://site.fingenious.fi/en/) managed by FINBB. Finnish Health register data can be applied from Findata (https://findata.fi/en/data/).
The GWAS summary statistics for the 23andMe data set will be made available through 23andMe to qualified researchers under an agreement with 23andMe that protects the privacy of the 23andMe participants. Please visit https://research.23andme.com/collaborate/#publication for more information and to apply to access the data. Fine-mapping results are provided in Supplementary Tables S3a, S4 and S5. PheWAS results can be browsed at https://hhautakangas.github.io/phewas_migraine_tables.html.

Research involving human participants, their data, or biological material

Policy information about studies with human participants or human data. See also policy information about sex, gender (identity/presentation), and sexual orientation and race, ethnicity and racism.

Reporting on sex and gender	In our study, only genetic sex information was utilized, not gender information. We used data from three existing GWAS data, where genetic sex had been used as a covariate. We were not able to perform any sex-specific analyses, because the GWAS summary data available to us did not include sex-specific GWAS results.
Reporting on race, ethnicity, or other socially relevant groupings	We used existing GWAS summary data that all included participants with European genetic ancestry. The genetic ancestry groups were labeled as "Finnish" for samples from FinnGen, "White British" following the annotation given by the UK biobank data resource and "European" for the European ancestry samples from 23andMe. In each GWAS, genetic ancestry had been assessed by using Principal component analysis. Possible effects from population stratification had been controlled in each GWAS by using genetic principal components (PCs) as covariates (first 5 PCs in 23andMe, and first 10 PCs in the UK Biobank and the FinnGen).
Population characteristics	Overall migraine sample prevalence of the meta-analysis was 10.1%; 23andMe 19% (of which 70.0% females), UK Biobank 3% (of which 77% females), and FinnGen 10% (of which 84% females).Mean age of the FinnGen cohort was 54.1 years old, and of the UK Biobank migraine GWAS 56.9 years old. Information of mean age of 23andMe was not available, age bins are available in the supplementary table of Pickrell et al. Nature Genetics 2016.
Recruitment	Participants had been recruited through population-based biobank studies (UK Biobank and FinnGen), and a direct-to-consumer study (23andMe). FinnGen includes also prospective and retrospective epidemiological and disease-based cohorts. Migraine phenotype is self-reported in the UK Biobank and 23andMe GWAS, whereas in FinnGen migraine is defined based on medication purchase data. In both cases, it is possible that there are some migraine cases among the controls. This can bias allele frequency differences between the cases and controls by attenuating the true effect sizes, and therefore decreasing the statistical power of the study. Null variants should not be biased.<br>In addition, since FinnGen migraine phenotype is based on triptan purchases that are contraindicated in patients with cardiovascular diseases, it is possible that it represent a specific subset of migraine patients. Overrepresentation of migraineurs without any cardiovascular diseases could lead some FinnGen PheWAS associations where migraine risk alleles seem to have protective effect on cardiovascular phenotypes. However, it does not bias fine-mapping results.
Ethics oversight	All participating studies were approved by local research ethics committees and written informed consent was obtained from all study participants.<br>A human subjects protocol of 23andMe study had been reviewed and approved by Ethical & Independent Review Services, a private institutional review board (http://www.eandireview.com).<br>UK Biobank received ethical approval from the North West Multi-centre Research Ethics Committee (MREC).<br>The FinnGen study protocol has been approved by the Coordinating Ethics Committee of the Hospital District of Helsinki and Uusimaa (HUS) (Nr HUS/990/2017).

Note that full information on the approval of the study protocol must also be provided in the manuscript.

Field-specific reporting

Please select the one below that is the best fit for your research. If you are not sure, read the appropriate sections before making your selection.

☒ Life sciences ☐ Behavioural & social sciences ☐ Ecological, evolutionary & environmental sciences

For a reference copy of the document with all sections, see nature.com/documents/nr-reporting-summary-flat.pdf

Life sciences study design

All studies must disclose on these points even when the disclosure is negative.

Sample size	Our study sample size was not predetermined. Our meta-analysis study sample size (98,374 migraine cases and 869,160 controls) was determined by the availability of linkage disequilibrium (LD) information. For the fine-mapping analyses, we had full in-sample LD available for 26 regions, and for rest of the regions we had partial in-sample LD available from the FinnGen and the UK Biobank studies.
Data exclusions	From each GWAS studies included in the meta-analysis standard QC protocols for variant and individual exclusions had been used. Further details are provided in the Methods section. For a meta-analysis, we additionally excluded the SNPs with an effect allele frequency (EAF) discrepancy of >0.30 and indels with an EAF discrepancy of >0.20 compared to UK Biobank from each study. For fine-mapping, we included only autosomal SNPs that were available in all three GWAS studies included in the meta-analysis, other variants were excluded. This strict QC criteria was used because variation in the sample sizes of different variants can bias fine-mapping results.
Replication	For a replication of the 35 new migraine risk loci, we meta-analyzed an external International Headache Genetics Consortium (IHGC) migraine meta-analysis data from 2016 excluding 23andMe and Finnish data and the HUNT All-in Headache migraine GWAS, with 34,807 migraine cases and 193,475 controls. Of the 35 lead variants of the risk loci, 17 replicated with a $P < 0.05$, and 32/35 were consistent in effect direction ($P = 2.1 \times 10{-}7$ from one-sided binomial test).
Randomization	We did not study experimental groups.
Blinding	We did not study experimental groups.

Reporting for specific materials, systems and methods

We require information from authors about some types of materials, experimental systems and methods used in many studies. Here, indicate whether each material, system or method listed is relevant to your study. If you are not sure if a list item applies to your research, read the appropriate section before selecting a response.

Materials & experimental systems

n/a	Involved in the study	
☒	☐	Antibodies
☒	☐	Eukaryotic cell lines
☒	☐	Palaeontology and archaeology
☒	☐	Animals and other organisms
☒	☐	Clinical data
☒	☐	Dual use research of concern
☒	☐	Plants

Methods

n/a	Involved in the study	
☒	☐	ChIP-seq
☒	☐	Flow cytometry
☒	☐	MRI-based neuroimaging

Plants

Seed stocks	We did not study plants.
Novel plant genotypes	We did not study plants.
Authentication	We did not study plants.
